# Supplementary material for: A model species for agricultural pest genomics: the genome of the Colorado potato beetle, Leptinotarsa decemlineata (Coleoptera: Chrysomelidae)
Source: Sci Rep. 2018 Jan 31;8:1931. doi: 10.1038/s41598-018-20154-1 (PMC5792627; doi:10.1038/s41598-018-20154-1)
Supplement: Supplementary file 1 — Supplementary Materials [file 41598_2018_20154_MOESM1_ESM.pdf]

Supplementary Material

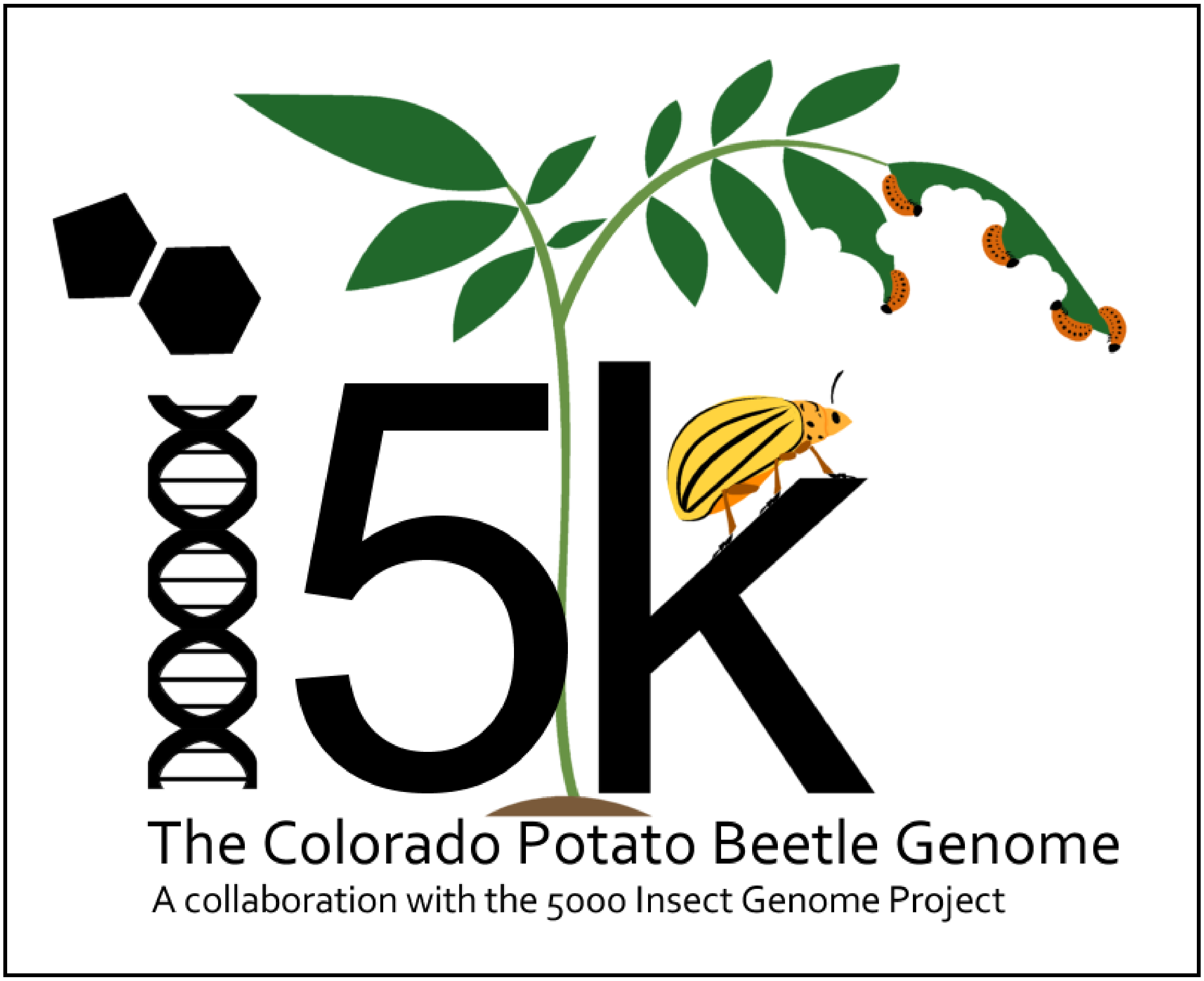

©

A model species for agricultural pest genomics: the genome of the Colorado potato beetle, *Leptinotarsa decemlineata* (Coleoptera: Chrysomelidae)

Sean D. Schoville, Yolanda H. Chen, Martin N. Andersson, Joshua B. Benoit, Anita Bhandari, Julia H. Bowsher, Kristian Brevik, Kaat Cappelle, Mei-Ju M. Chen, Anna K. Childers, Christopher Childers, Olivier Christiaens, Justin Clements, Elise M. Didion, Elena N. Elpidina, Patamarerk Engsontia, Markus Friedrich, Inmaculada García-Robles, Richard A. Gibbs, Chandan Goswami, Alessandro Grapputo, Kristina Gruden, Marcin Grynberg, Bernard Henrissat, Emily C. Jennings, Jeffery W. Jones, Megha Kalsi, Sher A. Khan, Abhishek Kumar, Fei Li, Vincent Lombard, Xingzhou Ma, Alexander Martynov, Nicholas J. Miller, Robert F. Mitchell, Monica Munoz-Torres, Anna Muszewska, Brenda Oppert, Subba Reddy Palli, Kristen A. Panfilio, Yannick Pauchet, Lindsey C. Perkin, Marko Petek, Monica F. Poelchau, Éric Record, Joseph P. Rinehart, Hugh M. Robertson, Andrew J. Rosendale, Victor M. Ruiz-Arroyo, Guy Smagghe, Zsofia Szendrei, Gregg W.C. Thomas, Alex S. Torson, Iris M. Vargas Jentzsch, Matthew T. Weirauch, Ashley D. Yates, George D. Yocum, June-Sun Yoon, Stephen Richards

---

© Illustration designed by Robert Mitchell.

Contents

Supplementary Methods: 5

Manual Gene Annotation and Classification 5

Homeodomain Transcription Factor Gene Clusters (Hox, Iro-C) 5

MicroRNA Annotation 5

Sensory Ecology 6

Plant Digestion 7

Supplementary Results 8

Genome Assembly, Assessment and Annotation 8

Genome Assembly 8

Assembly and Synteny Assessment: Homeodomain Transcription Factor Gene Clusters 8

Non-coding RNA Annotation 11

Sensory Ecology 12

Odorant Binding Proteins 12

Odorant Receptors 15

Gustatory Receptors 17

Ionotropic Receptors 20

Diapause and Environmental Stress 22

Predator Defense 24

Bt Receptors 24

Supplementary References Cited 25

Supplementary Tables 36

Table 1S. Positional information for the annotated homeobox genes 36

Table 2S. Interpro classification of rapidly changing gene families in the Leptinotarsa decemlineata lineage. 36

Table 3S. Gene ontology classification of rapidly changing gene families in the Leptinotarsa decemlineata lineage. 46

Table 4S. Transcription factors with predicted motifs. 58

Table 5S. Significantly differentially expressed genes, after Bonferroni correction, in mid-gut versus whole larval tissues of Leptinotarsa decemlineata. 67

Table 6S. Significantly differentially expressed genes, after Bonferroni correction, in an adult male and female Leptinotarsa decemlineata. 137

Table 7S. Significantly differentially expressed genes, after Bonferroni correction, in an adult male and larval Leptinotarsa decemlineata. 164

Table 8S. Significantly differentially expressed genes, after Bonferroni correction, in an adult female and larval Leptinotarsa decemlineata. 230

Table 9S. Transposable elements found in the genome of Lepinotarsa decemlineata. 286

Table 10S. List of genes in Leptinotarsa decemlineata genome with transposable elements in flanking regions. 287

Table 11S. Average pairwise genetic divergence (FST) estimated for Leptinotarsa decemlineata populations. 289

Table 12S. Demographic parameters estimated from the allele frequency spectrum for Leptinotarsa decemlineata populations. 290

Table 13S. The genes encoding odorant binding proteins (OBP) in Leptinotarsa decemlineata. 291

Table 14S. Details of the genes encoding odorant receptors (ORs) in Leptinotarsa decemlineata. 293

Table 15S. Details on the L. decemlineata gustatory receptors (GR) gene family. 295

Table 16S. Details on the L. decemlineata ionotropic receptor (IR) gene family. 301

Table 17S. Gene annotations related to diapause and environmental stress in Leptinotarsa. decemlineata. 303

Table 18S. Cysteine peptidases of Leptinotarsa decemlineata. 305

Table 19S. Serine peptidases of Leptinotarsa decemlineata 306

Table 20S. Summary of the number of Leptinotarsa decemlineata protein models assigned to families of the CAZy database and comparison to other insects. 307

Table 21S. Carbohydrate active enzymes and gene family assignments in the Leptinotarsa decemlineata genome. 309

Table 22S. Clusters of genes encoding cuticle proteins in the genome of Leptinotarsa decemlineata. 321

Table 23S. Genes associated with RNA interference in Leptinotarsa decemlineata. 321

Table 24S. Genes associated with venom in Leptinotarsa decemlineata. 322

Table 25S. The Cadherin genes, putative Bt receptors, annotated in Leptinotarsa decemlineata. 325

Supplementary Figures 326

Figure 1S. Organization of the Hox (A) and Iro-C clusters (B). 326

Figure 2S. Organization, synteny, and phylogeny of non-canonical homeodomain genes on Scaffold 63. 326

Figure 3S. Expression support for non-canonical homeodomain genes on Scaffold 63. 327

Figure 4S. Screen for possible contamination using BlobTools. 328

Figure 5S. Approximate maximum-likelihood phylogeny of odorant binding proteins (OBP). 329

Figure 6S. Unrooted phylogram illustrating the OR gene families of Leptinotarsa decemlineata. 330

Figure 7S. Approximate maximum-likelihood phylogeny of the gustatory receptor proteins (GRs)... 334

Figure 8S. Approximate maximum-likelihood phylogeny of the ionotropic receptor (IR) proteins. ... 335
Figure 9S. Phylogenetic tree of cys-loop ligand-gated ion channels in Leptinotarsa decemlineata. 336
Figure 10S. Representation of the exon structure of GABAR1 and the alternative versions of exon 3 and 6. 336
Figure 11S. Phylogenetic tree of cuticle proteins from Leptinotarsa decemlineata. 337
Figure 12S. Phylogenetic tree of cytosolic GST proteins from Leptinotarsa decemlineata. 338

Mention of trade names or commercial products in this publication is solely for the purpose of providing specific information and does not imply recommendation or endorsement by the U.S. Department of Agriculture. USDA is an equal opportunity provider and employer.

Supplementary Methods:

Manual Gene Annotation and Classification

Homeodomain Transcription Factor Gene Clusters (Hox, Iro-C)

Hox genes were annotated in the *Leptinotarsa decemlineata* genome by performing tBLASTn [1] searches on the *L. decemlineata* genomic scaffolds, using orthologous protein sequences from *Tribolium castaneum* (available in NCBI: official gene set Tcas3.0) and *Anoplophora glabripennis* [2]. Provisional models were refined, and potential gene duplications were identified, via iterative and reciprocal BLAST and by manual inspection and correction of protein alignments generated with ClustalW2 (www.ebi.ac.uk/Tools/msa/clustalw2) [3], using RNAseq expression evidence when available. Splice site information for *T. castaneum* was obtained for the OGS gene models as accessed from Assembly 4.0 in the genome browser of the Stanke group, University of Greifswald (http://bioinf.637 uni-greifswald.de/gb2/gbrowse/tcas4/).

MicroRNA Annotation

MicroRNAs (miRNA) were annotated by mapping all known mature arthropod miRNA sequences available on miRBase (http://www.mirbase.org/) [4] to the masked genome of *L. decemlineata* using Bowtie2 [5]. The putative precursor miRNAs (pre-miRNA) were extracted from the *L. decemlineata* genome based on the location of mature miRNAs in other arthropod species. If the mature miRNA was located at the 3'-end of precursor in other species, then we extracted the putative precursor 70 bp upstream of the mapped region. If the mature miRNA was located at 5'-end of precursor in other species, then we extracted the putative precursor 70 bp downstream of the mapped region. The extracted sequences were regarded as putative miRNAs if they satisfied following criteria: 1) the secondary structures of all putative pre-miRNAs were predicted by using RNAfold. Only those sequences with stem-loop structures and free energy lower than -18 kcal/mol were kept. 2) Stem-loop structures were tested using Triplet-svm-

predictor and the MapMi score, and sequences that did not pass the Triplet-svm-predictor test or had MapMi scores lower than 20 were removed. 3) The remaining sequences were blasted (BLASTn) against pre-miRNA sequences of reference arthropods, using a cut-off of e-value of 1e-5. The putative miRNAs were clustered using a single-linkage clustering algorithm to determine the miRNA family (precursor miRNAs are available in **Supplementary Dataset 1**). All the miRNA families were compared with the *T. castaneum* miRNAs [6]. If a *T. castaneum* miRNA family was not found by the abovementioned method, we searched the *L. decemlineata* genome again. If the mature and precursor miRNA sequences were found in the genome, we treated them as putative miRNAs.

Sensory Ecology

Chemosensory genes were identified by employing methods used in the *T. castaneum* genome project [6], relative to representative protein sequences from three outgroup species, *T. castaneum*, *A. glabripennis* and *D. melanogaster*, using tBLASTn searches. The four major gene families involved in chemoreception are the Odorant Binding Proteins (OBPs), Odorant Receptors (ORs), Gustatory Receptors (GRs), and Ionotropic Receptors (IRs). Provisional models were refined by manual inspection and correction, and fragmented and full-length genes were identified (protein sequences are included in the **Supplementary Supplementary Dataset 3**). Automated alignments of amino-acid translations were made for each group of genes using MUSCLE [7] or ClustalX [3] using default parameters, manually adjusted, and the alignments were trimmed using the GappyOut option in TrimAl v1.4 [8]. Maximum-likelihood phylogenies for each gene family were estimated using FastTree 2.1 [9] or PHYML v3.0 [10], and figures were prepared using FigTree v1.4.2 (http://tree.bio.ed.ac.uk/software/figtree/).

Plant Digestion

Peptidase genes were named according to the genus and species (e.g. *L. decemlineata*, Ld), peptidase similarity (Cat), and were noted as “species specific undefined” (ss.u), or predicted classical (B, L, F, O) and specific insect (I, Ll, Lc) peptidase types. To investigate the evolutionary relatedness of predicted cysteine peptidases, a phylogenetic analysis of predicted sequences greater than 200 amino acids and the reference human cathepsins L1, L2, B, C, F, H, K, O, S, W, Z, and human tubulointerstitial nephritis antigen-like (TINAL) protein was made. Clustal Omega [11] was used to develop the initial alignment. The evolutionary history was predicted using the Maximum Likelihood method based on the Poisson correction model [12] in MEGA6 [13]. The initial tree for the heuristic search was obtained by applying the best of Neighbor-Joining [14] and BioNJ [15] algorithms to a matrix of pairwise distances estimated using a JTT model, and then selecting the topology with superior log likelihood value. Bootstrap analysis of 200 repetitions were used to assess support for the phylogenetic tree.

Serine proteases (trypsins and chymotrypsins) were identified using *blastx* and assigned to gene families using MEROPS web service [16]. Similarly, *blastp* [17] was used to identify carbohydrate-active enzymes in the *L. decemlineata* genome using the carbohydrate-active enzymes database (CAZy, www.cazy.org) [18]. CAZy family inclusion was based on actual sequence conservation (percentage identity over the length of an entire CAZy domain). Briefly, proteins that gave more than 50% identity over the entire domain length of an entry in CAZy were directly assigned to the same family. Proteins with 15-50% identity to a protein already in CAZy were all manually inspected, aligned, and conserved features such as catalytic residues were searched. The variable modular structure of CAZy genes was integrated by performing alignments with isolated functional domains [19].

Supplementary Results

Genome Assembly, Assessment and Annotation

Genome Assembly

Using the kdr1 voltage-gated sodium channel (Vssc) gene (HQ589156) as a sex-linked marker [20], the *L. decemlineata* X-chromosome is identified as a single large scaffold in the ALLPATHS assembly (Scaffold# 362, 653.5 kb). In contrast, the mitochondrial genome is fragmented across several small scaffolds (Scaffold# 23662: 6.1 kb, Scaffold# 22044: 2.5 kb, Scaffold#23314: 1.6 kb) and is partially embedded (or is present as a numt) in one large scaffold (Scaffold# 6875: 2.3 kb).

Assembly and Synteny Assessment: Homeodomain Transcription Factor Gene Clusters

The Hox and Iroquois Complex (Iro-C) gene clusters encode highly conserved homeodomain transcription factors with essential roles in development. The Hox cluster is conserved across the Bilateria [21], and the Iro-C is found throughout the Insecta [22-24]. Annotation of the genes in these clusters provides an indicator of draft genome quality and an opportunity to assess synteny among closely related species. In *L. decemlineata*, we could construct complete gene models for all expected orthologs, with partial cluster assembly. Of note are (1) the high similarity in gene structure and cluster size with the Asian long-horned beetle, *A. glabripennis*, in contrast to the red flour beetle, *T. castaneum*, (2) a lineage-specific Hox gene duplication, (3) unexpected linkage to a homeobox gene of a different class, and (4) identification of two novel, linked homeobox genes.

We were able to find and annotate complete gene models for all ten Hox cluster genes, split across four different scaffolds (**Supplementary Figure 1SA**, **Supplementary Table 1S**). All linked Hox genes occurred in the expected order and with the expected, shared transcriptional orientation, suggesting that the current draft assembly is correct but simply

incomplete. Assuming direct concatenation of these four scaffolds, the Hox cluster would span a region of 3.7 Mb, similar to the estimated 3.5 Mb Hox cluster of *A. glabripennis* [2]. However, as the *L. decemlineata* genome is only about half the size, this reflects disproportionately large intergenic distances, as individual gene loci are not larger (see below). This may be due to possible local misassemblies with wrongly included sequence on these scaffolds. In the much smaller genome of *T. castaneum*, the Hox cluster's relative size is comparable to that seen in *A. glabripennis* (Tribolium Hox cluster of 0.7 Mb in a 160 Mb genome [6,25]).

For the small Iroquois Complex, while *L. decemlineata* has single, clear orthologs of both *iroquois* (*iro*) and *mirror* (*mirr*), here they are not linked in the current assembly (**Supplementary Figure 1SB**). Direct end-to-end concatenation of the relevant scaffolds would give a cluster size of 258 Kb, which at 74% of the *A. glabripennis* Iro-C cluster size is rather more proportional to genome size. As would be expected for an Iro-C cluster, the concatenated reconstruction in *L. decemlineata* includes no predicted genes between *iro* and *mirr*, and the two genes would have the same transcriptional orientation.

Leptinotarsa decemlineata Hox gene loci tend to be the same size or smaller than in *A. glabripennis*, encoding proteins of comparable size and on average only 10% longer than in *T. castaneum*. In all three beetle species, Hox protein sequence similarities were over 70%, and intron splice sites were largely conserved. Minor exceptions to these trends are the functionally diverged Hox genes *zerknüllt* (*zen*) and *fushi tarazu* (*ftz*), which might be expected to exhibit higher levels of sequence divergence [26-28]. These genes' loci are 10 and 6-fold larger, respectively, than in *Anoplophora*. Additionally, the protein sequence conservation across all three beetles was around 50% for *ftz*, and under 40% for *zen*.

In contrast, both *iro* and *mirr* are very strongly conserved between *L. decemlineata* and *Anoplophora*, with identical splice boundaries and gene structures, virtually identical protein sequences (93% and 88% identity, respectively), and even substantial stretches of nucleotide identity in both the 3' and 5' UTRs of both genes. The *L. decemlineata* genes are strongly expressed in adult female and 2nd instar larval RNAseq samples.

As would be expected for highly conserved genes, we only found evidence for single orthologs of all Hox and Iro-C genes except *zen*. Here, we found a tandem duplication resulting in a second gene we have termed *zen-like* (**Supplementary Figures 1SA**), as it is more divergent compared to the paralogue named *zen* and compared to orthologs from a variety of other insect species. However, *Ldec-zen* and *Ldec-zen-like* are likely a lineage specific duplication: the high level of sequence similarity between the paralogues results in them appearing as sister genes in Zen protein phylogenies (data not shown), making them distinct from the tandem duplications known from *T. castaneum* or other insects [28].

Surprisingly, we identified three additional homeobox genes 3' to *labial* (*Hox1*) on Scaffold 63 (**Supplementary Figure 1SA**). One of these is the ortholog of *intermediate neuroblasts defective* (*ind*), an embryonic patterning gene conserved across insects but not linked to the Hox cluster in *D. melanogaster, T. castaneum,* or *A. glabripennis.* Local synteny between *L. decemlineata* and *A. glabripennis* is conserved, as orthologs of a specific “uncharacterized protein” gene are linked to *ind* in both species (**Supplementary Figures 1S**).

Farther away along the scaffold (**Supplementary Figures 1SA**), we also identified a general Antennapedia-class (ANTP-class) homeobox gene with no clear ortholog in other arthropods, and which is most similar to the Hox gene *labial* **(Supplementary Figures 2S**). Although apparently unique, like the lineage-specific *zen-like*, this ANTP-class gene seems to be

real insofar as both have strong RNAseq expression support from multiple adult female libraries (**Supplementary Figure 3S**).

Finally, nested within the gene locus of the aforementioned *uncharacterized protein* (**Supplementary Figure 2SA**) was a curious automatic prediction for a gene that would encode eight tandem homeodomain DNA binding domains – an unprecedented situation. These homeodomains, along with their flanking protein sequence regions, belong to the Antennapedia- and anterior Hox gene class, but are highly redundant and more similar to one another than to other anterior Hox genes on this scaffold (**Supplementary Figure 2SC**). Although this gene model has canonical splice sites and encodes a large open reading frame (three exons each encoding 450, 539, or 964 amino acids), the unprecedented presence of multiple homeoboxes in a single gene, coupled to a lack of expression support from any of seven RNAseq libraries (**Supplementary Figure 3SB**), and the lack of an ortholog in *A. glabripennis* (syntenic or otherwise) casts doubt on whether this is an accurate gene model.

Non-coding RNA Annotation

We identified 85 putative miRNA loci representing 61 miRNA families in *L. decemlineata* (**Supplementary Dataset 2)**. One Coleoptera-specific miRNA, miR-3849, was found. This miRNA was shared by both *T. castaneum* and *L. decemlineata*, but is not known from other insect lineages. Interestingly, miR-3849 was located in the first intron of the *wingless* gene in both species, suggesting that this miRNA might have an important role in the development of coleopteran insects.

Sensory Ecology

Odorant Binding Proteins

Odorant binding proteins (OBP) are small soluble proteins that are highly abundant in the insect sensillar lymph. They are believed to solubilize hydrophobic odor molecules and transport them to the receptors that are housed in the membrane of olfactory sensory neuron dendrites [29-31]. OBPs have in some cases also been shown to influence odor responses, thus they might act as a pre-receptor filter, shaping the response specificity of olfactory sensory neurons (e.g. [32,33]). OBPs are characterized and classified into different groups based on the presence of conserved cysteine residues. Proteins of the Classic type have six conserved cysteines, Plus-C proteins have 12 cysteines and one characteristic proline, whereas Minus-C proteins usually have four cysteines [31]. Additionally, the Dimer class comprises large proteins with the Minus-C class motif repeated [29].

In total, 59 OBP genes were identified in the *L. decemlineata* genome assembly (**Table 2**, protein sequences are included in the **Supplementary Dataset 3**). This number is larger than the number of OBP genes in the genomes of *A. glabripennis* (52) [2] and *T. castaneum* (49) [6], but within the range of OBPs identified in other, non-coleopteran insect species [31]. A previous antennal transcriptome analysis only revealed 26 OBP-encoding transcripts in *L. decemlineata* [34]. All of the OBPs reported in this study could be re-identified in the genome, with overall consistent protein sequence prediction (see **Supplementary Table 13S** for discrepancies). Numbers to our OBP genes were given based on their positioning on genomic scaffolds and groupings in the tree (**Supplementary Figure 5S**). While our numbers do not correspond to the numbers in Liu *et al.* [34], we reference their classification along with genomic location and additional details of the OBP genes (**Supplementary Table 13S)**.

Of the 59 *L. decemlineata* OBP gene models, 57 encode full-length proteins and 17 of these were completed using available genomic and transcriptomic raw reads due to the N-terminus, C-terminus and/or internal regions of the genes being located in assembly gaps (suffix "FIX" added to gene name). One model remains partial (OBP38CTE) because its C-terminus could not be recovered from the raw reads (suffix "CTE" for C-terminus missing). Another gene (OBP10PSE) was characterized as a pseudogene since it appears to lack a first coding exon. Three of the genes (OBP44FJ, OBP55FJ, OBP56FJ) had exons assembled on multiple scaffolds, which for each gene could be merged into one model based on support from the raw reads (suffix "FJ" for FIX + JOIN). The automated gene prediction pipeline predicted only 16 of the 59 OBP genes correctly. Hence, 27 of the automated models had to be manually corrected, whereas 16 models had been missed entirely and thus were added manually. All of these gene models, where possible, are now annotated in the Apollo browser so that they will be available with the final protein release, but unassembled regions are not available therein.

A large proportion of OBP genes were present as tandemly duplicated arrays, with the largest tandem array comprising 10 OBP genes (LdecOBP45-54) over a 154 kb region of scaffold 160 (see **Supplementary Table 13S** for details). The majority of the OBP genes contained either one intron (23 genes) or five introns (26 genes). All genes with only one intron encoded the short signal peptide on the first exon, and the rest of the protein on the second exon. Of the 26 genes with five introns, 22 showed conserved exon/intron structure with introns 1, 2, 3, and 5 splicing in phase 0, and intron 4 in phase 1.

The 59 LdecOBPs were aligned with all OBPs from *A. glabripennis* and *T. castaneum* as well as a selected subset of OBPs from *D. melanogaster* using MUSCLE followed by limited manual correction. Uninformative regions of the alignment were removed with trimAL with the

similarity threshold set to 0, gap threshold set to 0.7, and 25% conserved positions allowed. A phylogeny of OBPs was constructed using FastTree 2.1 under default settings. This analysis reveals that two large OBP expansions (LdecOBP2-LdecOBP11 and LdecOBP37-59) are present in *L. decemlineata*. These OBP expansions are possibly related to its specialization on plants within Solanaceae (**Supplementary Figure 5S**). The other LdecOBPs are scattered across the tree, with the majority clustering with orthologs from the other two beetle species, and sometimes also with related proteins from *D. melanogaster*. Like other beetle species [2,31,35], *L. decemlineata* has one member of the Plus-C OBP class (LdecOBP29), which appears to be conserved across Coleoptera. In contrast, as many as 43 of the LdecOBPs (LdecOBP1-LdecOBP16, LdecOBP18-LdecOBP21 and LdecOBP37-LdecOBP59) belong to the Minus-C class of OBPs. All of these proteins contain four conserved cysteine residues. Interestingly, the Minus-C OBPs clustered in two sub-families in the tree, suggesting two independent losses of cysteines. One of the subfamilies contains LdecOBP1-LdecOBP16 and LdecOBP18-21 together with several of the Minus-C OBPs from *A. glabripennis* and *T. castaneum*. The other sub-family is comprised of the species-specific radiation of LdecOBP37-LdecOBP59, which is rooted by a smaller radiation of Minus-C OBPs from *A. glabripennis* (AglaOBP33-AglaOBP38). The absence of TcasOBPs in this clade suggests a more recent origin as compared to the other more species-diverse Minus-C clade. In turn, the entire clade of Minus-C proteins from *L. decemlineata* and *A. glabripennis* is rooted by a group of Classic OBPs with members from *T. castaneum* (TcasOBP19-21), *L. decemlineata* (LdecOBP36) and *D. melanogaster* (DmelOBP83a), supporting the idea of an independent emergence of this Minus-C protein clade [31]. We also identified one member of the Dimer class of OBPs (LdecOBP17), which to our knowledge is the first member of this class identified in Coleoptera. Also there is likely an

independent origin of a Dimer OBP in *L. decemlineata*, since it is part of a large array on a single scaffold along with the Minus-C OBPs12-19, all of which are somewhat related to each other. In addition, LdecOBP17 is not related to the Dimer OBPs in *D. melanogaster* (which therefore were left out from the tree). LdecOBP17 had the four-cysteine motif of Minus-C OBPs repeated, and grouped in the species-diverse clade of Minus-C OBPs. The remaining 14 LdecOBPs (LdecOBP22-LdecOBP28 and LdecOBP30-LdecOBP36) were of the Classic type.

In conclusion, the size of the genomic OBP repertoire in *L. decemlineata* is similar (or even slightly larger) to the size in other insect species. In contrast, the OR, GR, and IR families all appear reduced in *L. decemlineata* in relation to other beetle species (**Table 2**). Assuming that the reduction of the other chemosensory gene families (i.e. the receptors) reflects the host specialization of *L. decemlineata*, this result suggests that the OBP family in *L. decemlineata* has evolved differently in terms of repertoire size change in response to ecological adaptation. This result might not be surprising since the evolution of these gene families can be highly dynamic [31,36]. The absence of a repertoire size reduction among the OBPs could be due to the multiple physiological roles of these proteins [30].

Odorant Receptors

Odorant receptors are an insect-specific radiation of receptors present in the membranes of odorant receptor neurons in the insect sensillum [33], and are believed to be the primary mechanism for detecting and transducing olfactory signals (but see ionotropic receptors, e.g., [37]). To date, the odorant receptors of insects exist as heteromers containing two members: an olfactory receptor co-receptor (Orco) and an odorant receptor protein (OR), with the latter presenting the specific binding site for odorants [33]. While Orco is highly conserved and present as a single copy in winged insects [38], the ORs of insects evolve rapidly and show little

to no homology across insect orders. ORs initially appeared to cluster in seven major clades within the Coleoptera (Groups 1-7; [39,40]) but this organization is becoming less clear as more beetle receptors are described. Genes encoding putative odorant receptors were identified with iterating BLAST searches as described above, and models were built using Geneious v6.1.6 (Biomatters Ltd.). We also included the 36 ORs identified from Liu et al. [34] in our search; 25 were present in the genome, 10 proved to be redundant models, and one (OR28) could not be identified in the genome and showed no homology to known ORs. ORs have been renumbered in this manuscript to account for these discrepancies and to better organize the receptors by relatedness (see **Supplemental Table 14S** for correspondence of numbers).

We identified 75 OR genes in *L. decemlineata*, with four additional pseudogenes and a single Orco. Most OR genes included 4-6 introns, which often were quite large; complete genes frequently spanned over 30kb. Thus, many exons were situated in unsequenced regions of the genome and are missing, and only 30 full-length models were annotated (see **Supplemental Table 14S,** protein sequences are included in the **Supplementary Dataset 3)**. Three additional models (LdecOR23, 25, 54) were completed by comparison to data from Liu et al. [34]. The remaining models are missing N-terminal, internal, or C-terminal exons and are respectively denoted with NTE, INT, or CTE suffixes. When multiple suffixes were necessary, each was reduced to the single initial letters (e.g., NTE-INT becomes NI).

The complete set of LdecORs was aligned to ORs of *A. glabripennis* and *T. castaneum*, and analyzed as described above, but with untrimmed sequences. The phylogeny revealed that LdecORs could broadly be classified into the same broad groups defined by cerambycids and tenebrionids (**Supplemental Figure 6S**). We noted fewer and smaller radiations in *L. decemlineata* relative to the other two beetle species, which resulted in fewer overall ORs (*A.*

glabripennis: 121+11PSE, *T. castaneum*: 262+79PSE). However, as this is only the third genome-wide analysis of odorant receptors in the Coleoptera, the significance of this reduction is unclear.

Most LdecORs placed alongside radiations from the sister chrysomeloid *A. glabripennis*, with a notable exception of LdecOR52-55, which placed within a Group 3 radiation of *T. castaneum* ORs. No homologs were found in *L. decemlineata* for Group 4, Group 6, or the isolated TcasOR71-72. In fact, the placement of LdecORs offers further support to the hypothesis that Groups 5 and 6 are expansions restricted to the Tenebrionoidea, and might be merged into a broader family that includes Group 4 and a novel chrysomeloid expansion. This clade would be defined by the conserved outgroup of AglaOR101-103 and TcasOR275 [40], and here, by LdecOR56. Some small clades of receptors that were identified from previous beetle genomes [39,40] were also present in *L. decemlineata* by way of LdecOR45 ("Group 3A") and LdecOR77-79 (an as-yet unnamed clade identified in *A. glabripennis*). No ORs have been functionally characterized from beetles save for three pheromone receptors in the cerambycid *Megacyllene caryae* [41], so the significance of these relationships remains unknown.

Gustatory Receptors

Insect gustatory receptors (GRs) are seven transmembrane proteins which form ligand-activated ion channels [42]. They have a major role in gustation particularly for sweet and bitter taste and additional roles as receptors for CO2, pheromones, DEET, some amino acids and fatty acids and also thermal receptors (for a review, see [43]). A similar method used to identify *T. castaneum* Gr genes [6] was employed, except that the GR sequences from *T. castaneum, A. glabripennis* and *D. melanogaster* were used for the tBlastn search using Legacy BLAST available at the i5k Workspace@NAL website and the annotation was done on Apollo.

We identified 90 candidate Gr genes and three additional pseudogenes in the *L. decemlineata* genome (**Table 2**, protein sequences are included in the **Supplementary Dataset 3**). They seem to encode a total of 144 GRs via alternative splicing. Among them, 99 are potentially full-length receptors (378 amino acids in average) and 45 are partial proteins (200 amino acids minimum) which were limited by gaps in genome assembly and/or end of DNA scaffolds (**Supplementary Table 15S)**. The number of *L. decemlineata* Gr genes is fewer than those of *T. castaneum* (245 TcasGrs) and *A. glabripennis* (234 AglaGrs) which are the only two beetle species that the entire Gr gene repertoires were reported [2,6].

To understand evolutionary relationships of *L. decemlineat*a Gr genes to other insects, and to gain more insight into their putative functions, we built a phylogenetic tree from protein sequences of 147 LdecGRs, 245 TcasGRs, 234 AglaGRs and 39 DmelGRs [2,6,44] using FastTree 2.1. The phylogenetic tree reveals 4 major gene lineages including putative CO2 receptors, sugar receptors, fructose receptors and bitter receptors and also reveals many large lineage specific gene expansions (**Supplementary Figure 7S**). There are three putative carbon dioxide receptors (LdecGr1-3) which show simple 1:1 orthologous relationships with those of *T. castaneum* and *A. glabripennis*. These proteins show high sequence homology with *Drosophila* CO2 receptors (DmelGR21a and 63a) suggesting their conserved role in CO2 sensing [45,46]. Six candidate sugar receptor genes were identified (LdecGr4-9), which are fewer than those of *T. castaneum* (16 genes; TcasGr4-19) and *A. glabripennis* (10 genes, AglaGr4-13). Roles of DmelGR5a, 64a, and 64f in sensing sugar (e.g. sucrose, maltose and trehalose) have been demonstrated [47-49]. This clade is one of the oldest branches of chemoreceptor genes as their orthologs have been identified in all insects, and can be related to a crustacean ancestor [50,51], suggesting the important role for the perception of carbohydrate food sources.

We identified only one candidate fructose receptor (LdecGr10NI) which has a single ortholog in *D. melanogaster* (DmelGr43a) and B. mori (BmorGr9) [42,52]. However, this clade is expanded in *T. castaneum* (10 genes, TcasGr20-28 and TcasGr183) and *A. glabripennis* (3 genes, AglaGr14-16) which may suggest a higher requirement for sensing fructose in the two beetles although we could not exclude the possibility that additional genes were not discovered due to gaps in *L. decemlineata* genome assembly.

Other highly diverse GRs are generally classified as bitter receptors [44,53]. More than 90% of LdecGr models (138/147) are in this category and most of them have one phase 0 intron suggesting the common origin. Functions of some receptors in this clade were demonstrated in *D. melanogaster* e.g. DmelGR8a for sensing L-canavanine [54], DmelGR93a, 33a and 66a for sensing caffeine [55] and DmelGR68a and 39a for sensing female produced sex pheromone [56]. We could not identify clear orthologous genes of these receptors in L. decemlineata and other beetles suggesting that flies and beetles have independently evolved different receptors for sensing these tastants. Large lineage specific expansion in the beetle bitter receptor clades was observed e.g a clade of LdecGr44-53 and a clade of AglaGr17-56 which encode 33 and 48 putative proteins via alternative splicing, respectively. This presumably reflects gustatory adaptation for different ecological niches, e.g., feeding behavior on the *Solanum* plants of *L. decemlineata*.

Another interesting aspect for the beetle Gr genes is that some loci encode multiple proteins by alternative splicing. For example, LdecGr48 locus has 13 potential long N-terminal exons, all alternatively-spliced into a shared C-terminus exon and, in *T. castaneum*, TcasGr124 locus potentially encode 24 intact proteins [6]. None of *T. castaneum* and *L. decemlineata* odorant receptor genes are alternatively spliced [39], which could partly be explained by the

strict gene regulation control of insect Or genes (only one or few Or genes per neuron) [57-59]. Multiple gustatory receptors in the same neuron may contribute to the discrimination of tastants with similar structures.

Ionotropic Receptors

The IR family of chemoreceptors is a variant lineage of the ancient ionotropic glutamate recepotors involved in both olfaction and gustation [60]. It consists of 27 genes (**Table 16S** and **Figure 8S**). Like other insect genomes, *L. decemlineata* contains two highly conserved receptors (Ir8a and 25a) that are closely related to the original ionotropic glutamate receptors (see [51,61]), and which serve as co-receptors with most of the other IRs [60]. Ir76b is similarly conserved, but like other insects is much shorter at the N-terminus, and apparently serves as a co-receptor for IRs sensing salt, amines, and amino acids (e.g. [62]). Another group of receptors (Ir21a, 40a, 68a, and 93a) are present as conserved single orthologs, as is the case for most other insects, and this set have recently been implicated in sensing temperature and humidity (e.g. [63,64]). The Ir41 and 75 lineages consist of multiple genes in most insects including beetles, but in *L. decemlineata* there is a single ortholog of Ir41a, which is involved in sensing polyamines in *D. melanogaster* [65], whereas the Ir75 clade has siz genes involved in sensing various acids in *D. melanogaster* [66,67]. Finally, there is a set of highly divergent genes, only one of which has a clear relationship with the *D. melanogaster* IRs, Ir100a, a receptor of unknown function. Most are intronless, and those that have introns have idiosyncratically located introns in different phases, so likely were gained relatively recently (**Table 16S**). This pattern of mostly intronless genes is typical for these divergent IRs in other insects.

Following an approach initiated with the termite *Zootermopsis nevadensis* [51], and applied to several other insects including *A. glabripennis* and *T. castaneum* [2], the conserved

IRs are named for their *D. melanogaster* orthologs, the Ir41 and 75 lineages are named with suffices a and a-f, while the divergent genes, except Ir100a, are numbered from 101 to avoid any confusion with the *D. melanogaster* IR names which only go to 100a. As discussed briefly in McKenna et al. [2], the *T. castaneum* IRs are far more numerous than reported by Croset et al. [61], with 53 divergent IRs added to their gene set for a new total of 80 genes, eight of which are clearly pseudogenes, and the complete set of these proteins with this modified naming system is included along with the *L. decmlineata* IR proteins (provided in **Supplementary Dataset 3**).

The conserved *L. decmlineata* IR genes were particularly difficult to model. The fractured nature of the assembly, combined with the fact that they are generally large genes spanning up to 100kb and have many short exons, caused problems ranging from missing or truncated exons in gaps, to genes split across scaffolds, including two instances of exons on different scaffolds being interdigitated with one another. By focusing on conserved sequence and RNAseq support, fairly complete models were constructed for most IR genes (provided in **Supplementary Dataset 3**). However, many of these IR genes cannot be properly modeled in Apollo and hence are only partially available at the i5k WorkSpace@NAL. Thus, four of the 14 conserved genes required repair of the assembly, commonly using raw RNAseq reads (the RNAseq reads mapped in Apollo are not useful when an exon is missing or a gene is split across scaffolds or otherwise misassembled), as well as raw genome reads (**Table 16S**). Two genes still have internal exons missing, while the N-terminus remains unclear for three genes. Official models existed for at least parts of most of the conserved genes, but the divergent genes, despite commonly being intronless, were usually only represented in the AUGUSTUS gene set. Twelve entirely new gene models were created (**Table 16S**).

This repertoire of 14 conserved and 13 divergent IRs is smaller than the other sequenced beetles, *T. castaneum* having a set of 16 conserved and 64 divergent IRs, while *A. glabripennis* has a set of 16 conserved and 56 divergent IRs [2]. The major differences are in the numbers of divergent lineages. In stark contrast with both *T. castaneum* and *A. glabripennis*, there is just one convincing lineage-specific duplication amongst these divergent IRs (LdecIr104/105), compared with multiple large lineage-specific expansions of up to 15 and 20 genes in *T. castaneum* and *A. glabripennis*, respectively (**Figure 8S**). Furthermore, this is the only tandemly-duplicated pair of genes in this family (**Table 16S**). In addition, it appears that several divergent IR lineages have been lost from *L. decemlineata*. These divergent IRs in the beetles are related to two clades of IRs in *D. melanogaster*, the Ir7/11a clade of genes expressed in larval and adult gustatory organs [61] and the much larger Ir20a clade also implicated in gustation rather than olfaction [60,68,69], but branch support levels are low within this set of divergent IRs. Ligands are not known for any of these divergent IRs in *D. melanogaster*, but this considerable potential gustatory receptor contraction in *L. decemlineata* is in line with the contraction of the GR family, and is presumably related to the host specialization of this beetle.

Diapause and Environmental Stress

To survive reoccurring environmental stress such as cold or dry seasons, insects employ a neuroendocrine controlled state of diapause [70], a form of dormancy characterized by developmental arrest, decreased metabolism [71], and increased resistance to environmental stress including increased cold hardiness and desiccation resistance [72,73]. Key components of the genetic mechanisms underlying diapause and environmental stress in other species were found in *L. decemlineata* genome assembly (**Table 17S**).

Diapause associated genes include the insulin signaling pathway, with homology to the insulin receptor, forkhead box protein O (FOXO) and insulin-like peptides. The full genome sequencing explained previously observed differences in regulation of two similar mRNA transcripts associated with diapause development (AF532863.1 named diapause-associated transcript-2 (DAT-2) [74] and FG591177.1 [75]). There are in fact four DAT-2 mRNA transcripts that represent distinct isoforms of the same gene locus, sharing one exon, but differing over the remainder of the alignment. DAT-2 does not align with proteins in NCBI's non-redundant database outside of beetles, demonstrating that this protein is lineage specific. Following the DAT-2 gene, we found a tandem cluster of four DAT-2-like genes. The architecture of this gene family may have been influenced by the location of a transposable element located within the intron of DAT-2 [76].

The heat shock protein complex includes 13 members of the small hsp family and 30 genes containing the DnaJ domain (possible hsp40 family) in addition to the hsp90 and hsp70 families. Notably, genes homologous with the cold-tolerance genes Frost [77,78] and antifreeze proteins [79,80] appear to be absent from *L. decemlineata*. Aquaporins (AQPs) are critical in the movement of water and other small solutes across cell membranes [81]. Nine putative aquaporin genes were identified in *L. decemlineata*, similar to the number present in other beetles, but only three of these genes were full length. The unorthodox AQP, called AQP6, is not present in the gene predictions, although it is known from all other insect genomes examined to date, including the red flour beetle, *Tribolium castaneum* [6].

We also annotated genes underlying insect antioxidant responses and autophagy genes. Thirty-six putative proteins in eight families (omitting the glutathione-S-transferase family) related to antioxidant capacity were identified within the *L. decemlineata* genome. Compared to

the *Bombyx mori* genome [82], this number is reduced due to the significant expansion of the heme-binding peroxidase subfamily in *B. mori*. However, there is no apparent reduction in comparison to *Apis mellifera* or *T. castaneum*. Autophagy is the process of degrading proteins, organelles and other cellular waste products, and is an important cellular process when organisms are faced with nutrient limitation or starvation [83]. A total of 16 autophagy genes were found in the *L. decemlineata* genome, with one-to-one orthology and high sequence similarity to *T. castaneum*.

Predator Defense

Leaf beetles, including *L. decemlineata*, use a combination of chemical defense and aposematism to deter predators [84]. Feeding experiments have demonstrated that *L. decemlineata* is distasteful to both anuran and avian predators [85,86]. Defensive glands are present in nearly uninterrupted lines along the pronotum and elytron of *L. decemlineata* [87] and contain two toxic amino acid derivatives, gamma-L-glutamyl-L-2-amino-Z3,5-hexadienoic acid and 2-aminoethanol [88]. In addition, the compound β-leptinotarsin-h has been isolated from hemolymph and acts as a calcium flux antagonist in vertebrate neuronal systems [89,90]. Using available peptide sequences, we identified the β-leptinotarsin-h as LDEC002191-RA. A broader search for toxins based on homology to known arthropod toxins found 67 strong candidates (**Table 24S**), including most notably 20 venom carboxylesterase-6 genes.

Bt Receptors

The entomopathogenic bacterium *Bacillus thuringiensis* (Bt) is the most widely used commercially successful biological control agent, and has been developed in transgenic potatoes where it has been effective in controlling *L. decemlineata* [91,92]. Bt toxins interact with protein receptors in the midgut of susceptible insects, which include aminopeptidase-N proteins in

Lepidoptera [93] and cadherin-like proteins in beetles [94,95], such as E-cadherin (TmCad1) in *T. molitor*. Notably, there are only 14 cadherin-like protein receptors found in *L. decemlineata* (**Table 25S**), compared to 61 in *T. castaneum*. It is currently unclear what other potential receptor sites for Bt toxins exist in *L. decemlineata*.

Supplementary References Cited

1. Camacho C, Coulouris G, Avagyan V, Ma N, Papadopoulos J, Bealer K, et al. BLAST+: architecture and applications. BMC Bioinformatics. 2009;10:421.

2. McKenna DD, Scully ED, Pauchet Y, Hoover K, Kirsch R, Geib SM, et al. Genome of the Asian longhorned beetle (*Anoplophora glabripennis*), a globally significant invasive species, reveals key functional and evolutionary innovations at the beetle–plant interface. Genome Biol 2016;17:227.

3. Larkin MA, Blackshields G, Brown NP, Chenna R, McGettigan PA, McWilliam H, et al. Clustal W and Clustal X version 2.0. Bioinformatics. 2007;23:2947–8.

4. Kozomara A, Griffiths-Jones S. miRBase: annotating high confidence microRNAs using deep sequencing data. Nucleic Acids Res. 2013;42:D68--D73.

5. Langmead B, Salzberg SL. Fast gapped-read alignment with Bowtie 2. Nat. Methods. 2012;9:357.

6. Tribolium genome sequencing consortium. The genome of the model beetle and pest *Tribolium castaneum*. Nature 2008;452(7190):949-955.

7. Edgar RC. MUSCLE: multiple sequence alignment with high accuracy and high throughput. Nucleic Acids Res. 2004;32:1792–7.

8. Capella-Gutierrez S, Silla-Martinez JM, Gabaldon T. trimAl: a tool for automated alignment trimming in large-scale phylogenetic analyses. Bioinformatics 2009;25:1972-1973.

9. Price MN, Dehal PS, Arkin AP. FastTree 2--approximately maximum-likelihood trees for large alignments. PLoS One. 2010;5:e9490.

10. Guindon S, Dufayard JF, Lefort V, Anisimova M, Hordijk W, Gascuel O. New algorithms and methods to estimate maximum-likelihood phylogenies: assessing the performance of PhyML 3.0. Syst Biol. 2010;59(3):307-21.

11. Sievers F, Wilm A, Dineen D, Gibson TJ, Karplus K, Li W, et al. Fast, scalable generation of high-quality protein multiple sequence alignments using Clustal Omega. Mol Syst Biol. 2011;7:539.

12. Zuckerkandl E, Pauling L. Evolutionary divergence and convergence in proteins. Evol Genes Proteins. 1965;97:97–166.

13. Tamura K, Stecher G, Peterson D, Filipski A, Kumar S. MEGA6: Molecular Evolutionary Genetics Analysis version 6.0. Mol Biol Evol. 2013;30:2725–9.

14. Saitou N, Nei M. The neighbor-joining method: a new method for reconstructing phylogenetic trees. Mol. Biol. Evol. 1987;4:406–25.

15. Gascuel O. BIONJ: an improved version of the NJ algorithm based on a simple model of sequence data. Mol. Biol. Evol. 1997;14:685–95.

16. Rawlings ND, Waller M, Barrett AJ, Bateman A. MEROPS: the database of proteolytic enzymes, their substrates and inhibitors. Nucleic Acids Res. 2014;42:D503--D509.

17. Altschul SF, Madden TL, Schäffer AA, Zhang J, Zhang Z, Miller W, et al. Gapped BLAST and PSI-BLAST: a new generation of protein database search programs. Nucleic Acids Res. 1997;25:3389–402.

18. Lombard V, Golaconda Ramulu H, Drula E, Coutinho PM, Henrissat B. The carbohydrate-active enzymes database (CAZy) in 2013. Nucleic Acids Res. 2013;42:D490--D495.

19. Cantarel BL, Coutinho PM, Rancurel C, Bernard T, Lombard V, Henrissat B. The Carbohydrate-Active EnZymes database (CAZy): an expert resource for glycogenomics. Nucleic Acids Res. 2008;37:D233--D238.
20. Hawthorne DJ. AFLP-Based genetic linkage map of the Colorado potato beetle *Leptinotarsa decemlineata*: Sex chromosomes and a pyrethroid-resistance candidate gene. Genetics. 2001;158:695–700.
21. Krumlauf R. Evolution of the vertebrate Hox homeobox genes. BioEssays, 1992;14(4):245-252.
22. Benoit JB, Adelman, ZN, Reinhardt K, Dolan A, Poelchau M, Jennings, EC, et al. Unique features of a global human ectoparasite identified through sequencing of the bed bug genome. Nat Commun. 2016;7:10165.
23. Cavodeassi F, Modolell J, Gómez-Skarmeta JL. The Iroquois family of genes: from body building to neural patterning. Development 2001;128:2847-2855.
24. McNeill H, Yang CH, Brodsky M, Ungos J, Simon MA. mirror encodes a novel PBX-class homeoprotein that functions in the definition of the dorsal-ventral border in the *Drosophila* eye. Genes Dev. 1997;11(8):1073-1082.
25. Brown SJ, Fellers JP, Shippy TD, Richardson EA, Maxwell M, Stuart JJ, et al. Sequence of the *Tribolium castaneum* homeotic complex: The region corresponding to the *Drosophila melanogaster* Antennapedia Complex. Genetics, 2002;160:1067-1074.
26. Akam M, Averof M, Castelli-Gair J, Dawes R, Falciani F, Ferrier D. The evolving role of Hox genes in arthropods. Development 1994;Supplement(1994):209-215.
27. Hughes CL, Kaufman TC. Hox genes and the evolution of the arthropod body plan. Evol Dev. 2002;4(6):459-499.

28. Panfilio KA, Akam M. A comparison of Hox3 and Zen protein coding sequences in taxa that span the Hox3/zen divergence. Dev Genes Evol. 2007;217(4):323-329.

29. Hekmat-Scafe DS, Scafe CR, McKinney AJ, Tanouye MA. Genome-wide analysis of the odorant-binding protein gene family in *Drosophila melanogaster*. Genome Res 2002;12(9): 1357-1369.

30. Pelosi P, Zhou JJ, Ban LP, Calvello M. Soluble proteins in insect chemical communication. Cell Mol Life Sci 2006;63(14):1658-1676.

31. Sánchez-Gracia A, Vieira FG, Rozas J. Molecular evolution of the major chemosensory gene families in insects. Heredity 2009;103(3):208-216.

32. Große-Wilde E, Svatoš A, Krieger J. A pheromone-binding protein mediates the bombykol-induced activation of a pheromone receptor in vitro. Chem Senses 2006;31(6):547-555.

33. Leal WS. Odorant reception in insects: Roles of receptors, binding proteins, and degrading enzymes. Annu Rev Entomol 2013;58(1):373-391.

34. Liu Y, Sun L, Cao D, Walker WB, Zhang Y, Wang G. Identification of candidate olfactory genes in *Leptinotarsa decemlineata* by antennal transcriptome analysis. Front Ecol Evol. 2015;3:60.

35. Andersson MN, Grosse-Wilde E, Keeling CI, Bengtsson JM, Yuen MM, Li M, Hillbur Y, Bohlmann J, Hansson BS, Schlyter F. Antennal transcriptome analysis of the chemosensory gene families in the tree killing bark beetles, *Ips typographus* and *Dendroctonus ponderosae* (Coleoptera: Curculionidae: Scolytinae). BMC Genomics 2013;14:198:1-16.

36. Nei M, Niimura Y, Nozawa M. The evolution of animal chemosensory receptor gene repertoires: roles of chance and necessity. Nat Rev Genet 2008;9(12):951-963.

37. Min S, Minrong A, Shin SA, Sug GSB. Dedicated olfactory neurons mediating attraction behavior to ammonia and amines in *Drosophila*. Proc Natl Acad Sci U S A 2013;110:E1321-E1329.

38. Missbach C, Dweck HKM, Vogel H, Vilcinskas A, Stensmyr MC, Hansson BS, et al. Evolution of insect olfactory receptors. eLife 2014;3:e02115.

39. Engsontia P, Sanderson AP, Cobb M, Walden KKO, Robertson HM, Brown S. The red flour beetle's large nose: An expanded odorant receptor gene family in *Tribolium castaneum*. Insect Biochem Mol Bio. 2008;38:387-397.

40. Mitchell RF, Hall LP, Reagel PF, McKenna DD, Baker TC, Hildebrand JG. Odorant receptors and antennal lobe morphology offer a new approach to understanding the olfactory biology of the Asian longhorned beetle. J Comp Physiol A 2017;203:99-109.

41. Mitchell RF, Hughes DT, Luetje CW, Millar JG, Soriano-Agatón F, Hanks LM, et al. Sequencing and characterizing odorant receptors of the cerambycid beetle *Megacyllene caryae*. Insect Biochem Mol Bio.l 2012;42:499-505.

42. Sato K, Tanaka K, Touhara K. Sugar-regulated cation channel formed by an insect gustatory receptor. Proc Natl Acad Sci U S A. 2011;108:11680–11685.

43. Liman ER, Zhang YV, Montell C. Peripheral coding of taste. *Neuron* 2014;81:984-1000. 45.

44. Robertson HM, Warr CG, Carlson JR. Molecular evolution of the insect chemoreceptor gene superfamily in *Drosophila melanogaster*. Proc Natl Acad Sci U S A. 2003;100(Suppl 2):14537–14542.

45. Jones WD, Cayirlioglu P, Kadow IG, Vosshall LB. Two chemosensory receptors together mediate carbon dioxide detection in *Drosophila.* Nature 2007;445:86–90.

46. Kwon JY, Dahanukar A, Weiss LA, Carlson JR. The molecular basis of CO_2 reception in *Drosophila*. Proc Natl Acad Sci U S A. 2007;104:3574–3578.

47. Dahanukar A, Lei YT, Kwon JY, Carlson JR. Two Gr genes underlie sugar reception in *Drosophila*. Neuron 2007;56:503–516.

48. Jiao Y, Moon SJ, Wang X, Ren Q, Montell C. Gr64f is required in combination with other gustatory receptors for sugar detection in *Drosophila*. Curr Biol. 2008;18:1797–1801.

49. Slone J, Daniels J, Amrein H. Sugar receptors in *Drosophila*. Curr Biol. 2007;17:1809–1816.

50. Kent L, Robertson H. Evolution of the sugar receptors in insects. BMC Evolutionary Biology 2009;9:41.

51. Terrapon N, Li C, Robertson HM, Ji L, Meng X, Booth W, et al. Molecular traces of alternative social organization in a termite genome. Nat Commun 2014;5:3636.

52. Miyamoto T, Slone J., Song X, Amrein H. A fructose receptor functions as a nutrient sensor in the *Drosophila* brain. Cell 2012;151:1113–1125.

53. Weiss LA, Dahanukar A, Kwon JY, Banerjee D, Carlson JR. The molecular and cellular basis of bitter taste in *Drosophila*. Neuron 2011;69:258-272.

54. Lee Y, Kang MJ, Shim J, Cheong CU, Moon SJ, Montell C. Gustatory receptors required for avoiding the insecticide L-canavanine. J Neurosci. 2012;32:1429–1435.

55. Lee Y, Moon SJ, Montell C. Multiple gustatory receptors required for the caffeine response in *Drosophila*. Proc Natl Acad Sci U S A. 2009;106:4495–4500.

56. Bray S, Amrein H. A putative *Drosophila* pheromone receptor expressed in male-specific taste neurons is required for efficient courtship. Neuron 2003;39:1019–1029.

57. Goldman AL, Van der Goes van Naters W, Lessing D, Warr CG, Carlson JR. Coexpression of two functional odor receptors in one neuron. *Neuron* 2005;45:661-666.

58. Fishilevich E, and Vosshall LB. Genetic and functional subdivision of the *Drosophila* antennal lobe. Curr Biol. 2007;17:1180.

59. Ray A, van der Goes van Naters W, Shiraiwa T, Carlson JR. Mechanisms of odor receptor gene choice in *Drosophila*. Neuron 2007;53:353-369.

60. Rytz R, Croset V, Benton R. Ionotropic receptors (IRs): chemosensory ionotropic glutamate receptors in *Drosophila* and beyond. Insect Biochem Mol Biol. 2013;43(9):888-97.

61. Croset V, Rytz R, Cummins SF, Budd A, Brawand D, Kaessmann H, et al. Ancient protostome origin of chemosensory ionotropic glutamate receptors and the evolution of insect taste and olfaction. PLoS Genet. 2010;;6(8):e1001064.

62. Ganguly A, Pang L, Duong VK, Lee A, Schoniger H, Varady E, et al. A molecular and cellular context-dependent role for Ir76b in detection of amino acid taste. Cell Rep. 2017;18(3):737-750.

63. Knecht ZA, Silbering AF, Ni L, Klein M, Budelli G, Bell R, et al. Distinct combinations of variant ionotropic glutamate receptors mediate thermosensation and hygrosensation in *Drosophila*. eLife. 2016;5: e17879.

64. Knecht ZA, Silbering AF, Cruz J, Yang L, Croset V, Benton R, et al. Ionotropic Receptor-dependent moist and dry cells control hygrosensation in *Drosophila*. eLife. 2017;6: e26654.

65. Hussain A, Zhang M, Üçpunar HK, Svensson T, Quillery E, Gompel N, et al. Ionotropic chemosensory receptors mediate the taste and smell of polyamines. PLoS Biol. 2016;14(5):e1002454. Erratum in: PLoS Biol. 2016;14(6):e1002505.

66. Prieto-Godino LL, Rytz R, Bargeton B, Abuin L, Arguello JR, Peraro MD, et al. Olfactory receptor pseudo-pseudogenes. Nature. 2016;539(7627):93-97.

67. Prieto-Godino LL, Rytz R, Cruchet S, Bargeton B, Abuin L, Silbering AF, Ruta V, Dal Peraro M,Benton R. Evolution of acid-sensing olfactory circuits in drosophilids. Neuron. 2017;93(3):661-676.

68. Koh TW, He Z, Gorur-Shandilya S, Menuz K, Larter NK, Stewart S, et al. The *Drosophila* IR20a clade of ionotropic receptors are candidate taste and pheromone receptors. Neuron. 2014;83(4):850-65.

69. Stewart S, Koh TW, Ghosh AC, Carlson JR. Candidate ionotropic taste receptors in the *Drosophila* larva. Proc Natl Acad Sci U S A. 2015;112(14):4195-201.

70. Denlinger DL, Yocum GD, Rinehart JP. Hormonal Control of Diapause. In: Gilbert LI, editor. Insect Endocrinology. Academic Press; 2012. p. 430–63.

71. Tauber MJ, Tauber CA, Masaki S. Seasonal Adaptations of Insects. Oxford, U. K.: Oxford University Press; 1986.

72. Lee RE, Denlinger DL. Cold tolerance in diapausing and non-diapausing stages of the flesh fly, *Sarcophaga crassipalpis*. Physiol Entomol. 1985;10:309–15.

73. Rinehart JP, Li A, Yocum GD, Robich RM, Hayward SAL, Denlinger DL. Up-regulation of heat shock proteins is essential for cold survival during insect diapause. Proc Natl Acad Sci U S A. 104:11130–7.

74. Yocum GD. Isolation and characterization of three diapause-associated transcripts from the Colorado potato beetle, *Leptinotarsa decemlineata*. J Insect Physiol. 2003;49:161–9.

75. Yocum GD, Rinehart JP, Chirumamilla-Chapara A, Larson ML. Characterization of gene expression patterns during the initiation and maintenance phases of diapause in the Colorado potato beetle, *Leptinotarsa decemlineata*. J Insect Physiol. 2009;55:32–9.

76. Yocum GD, Toutges MJ, Roehrdanz RL, Dihle PJ. Insertion of miniature subterminal inverted repeat-like elements in diapause-regulated genes in the Colorado potato beetle, *Leptinotarsa decemlineata* (Coleoptera: Chrysomelidae). Eur J Entomol. 2011;108:197–203.

77. Goto SG. A novel gene that is up-regulated during recovery from cold shock in Drosophila melanogaster. Gene. 2001;270:259–64.

78. Colinet H, Lee SF, Hoffmann A. Functional characterization of the *Frost* gene in *Drosophila melanogaster*: importance for recovery from chill coma. PLoS One. 2010;

79. Zachariassen KE, Husby JA. Antifreeze effect of thermal hysteresis agents protects highly supercooled insects. Nature. 1982;298:865–7.

80. Liou YC, Thibault P, Walker VK, Davies PL, Graham LA. A complex family of highly heterogeneous and internally repetitive hyperactive antifreeze proteins from the beetle *Tenebrio molitor*. Biochemistry. 1999;38:11415-11424.

81. Benoit JB, Hansen IA, Szuter EM, Drake LL, Burnett DL, Attardo GM. Emerging roles of aquaporins in relation to the physiology of blood-feeding arthropods. J Comp Physiol B. 2014;184:811–25.

82. Shi G-Q, Yu Q-Y, Zhang Z. Annotation and evolution of the antioxidant genes in the silkworm, *Bombyx mori*. Arch Insect Biochem. Physiol. 2012;79:87–103.

83. Malagoli D, Abdalla FC, Cao Y, Feng Q, Fujisaki K, Gregorc A, Matsuo T, Nezis IP, Papassideri IS, Sass M, Silva-Zacarin EC. Autophagy and its physiological relevance in arthropods: current knowledge and perspectives. Autophagy. 2010;6:575–88.

84. Deroe C, Pasteels JM. Distribution of adult defense glands in chrysomelids (Coleoptera: Chrysomelidae) and its significance in the evolution of defense mechanisms within the family. J Chem Ecol. 1982;8:67–82.

85. Boiteau G, McCarthy PC. Is there a role for stripes of adults and colour of larvae in determining the avoidance of the Colorado potato beetle by the American toad? Can J Zool. 2010;88:468–78.

86. Hough-Goldstein JA, Geiger J, Chang D, Saylor W. Palatability and toxicity of the Colorado potato beetle (Coleoptera: Chrysomelidae) to domestic chickens. Ann Entomol Soc Am. 1993;86:158.

87. Deroe C, Pasteels JM. Defensive mechanisms against predation in the Colorado beetle (*Leptinotarsa decemlineata*, Say). Arch Biol Sci. 1977;88:289–304.

88. Timmermans M, Randoux T, Daloze D, Braekman J-C, Pasteels JM, Lesages L. The chemical defence of Doryphorina beetles (Coleoptera: Chrysomelidae). Biochem Syst Ecol. 1992;20:343–9.

89. Hsiao TH, Fraenkel G. Properties of leptinotarsin: A toxic hemolymph protein from the Colorado potato beetle. Toxicon. 1969;

90. Crosland RD, Fitch RW, Hines HB. Characterization of β-leptinotarsin-h and the effects of calcium flux antagonists on its activity. Toxicon. 2005;45:829–41.

91. Zhou Z, Pang J, Guo W, Zhong N, Tian Y, Xia G, Wu J. Evaluation of the resistance of transgenic potato plants expressing various levels of Cry3A against the Colorado potato beetle (*Leptinotarsa decemlineata* Say) in the laboratory and field. Pest Manag Sci. 2012;68:1595–604.

92. Reed GL, Jensen AS, Riebe J, Head G, Duan JJ. Transgenic Bt potato and conventional insecticides for Colorado potato beetle management: comparative efficacy and non-target impacts. Entomol Exp Appl. 2001;100:89–100.

93. Knight PJK, Crickmore N, Ellar DJ. The receptor for *Bacillus thuringiensis* CrylA (c) delta-endotoxin in the brush border membrane of the lepidopteran *Manduca sexta* is aminopeptidase N. Mol Microbiol. 1994;11:429–36.

94. Fabrick J, Oppert C, Lorenzen MD, Morris K, Oppert B, Jurat-Fuentes JL. A novel *Tenebrio molitor* cadherin is a functional receptor for *Bacillus thuringiensis* Cry3Aa toxin. J Biol Chem. 2009;284:18401–10.

95. Vadlamudi RK, Weber E, Ji I, Ji TH, Bulla LA. Cloning and expression of a receptor for an insecticidal toxin of *Bacillus thuringiensis*. J Biol Chem. 1995;270:5490–4.

Supplementary Tables

Table 1S. Positional information for the annotated homeobox genes. Canonical Hox and Iro-C cluster genes are marked with an asterisk (*).

Gene	Scaffold: start..end	Locus length (nt)	Number of CDS exons
ANTP-class homeobox protein	Scaffold63:2067241..2077415	10,175	2
Multi-homeodomain protein	Scaffold63:3137133..3149049	11,917	3
intermediate neuroblasts defective	Scaffold63:3280621..3281538	918	1
*labial**	Scaffold63:3744845..3813550	68,706	2
*proboscipedia**	Scaffold63:3894360..4009077	114,718	6
*zerknüllt**	Scaffold63:4273821..4283268	9,448	3
zen-like	Scaffold63:4249154..4263329	14,176	3
*Deformed**	Scaffold527:216729..178194	38,534	2
*Sex combs reduced**	Scaffold527:486777..377520	109,256	3
*fushi tarazu**	Scaffold527:544743..538364	6,378	2
*Antennapedia**	Scaffold527:959581..934045	25,535	2
*Ultrabithorax**	Scaffold1588:40031..306611	266,581	2
*abdominal-A**	Scaffold549:409738..287522	122,215	3 or 4 (alternate isoforms)
*Abdominal-B**	Scaffold549:1026448..1020843	5,604	2
*iroquois**	Scaffold948:333348..401390	68,043	7
*mirror**	Scaffold12:99112..152497	53,386	5

Table 2S. Interpro classification of rapidly changing gene families in the *Leptinotarsa decemlineata* lineage.

Orthgroup IDs	Interpro Number	Interpro Classifiction
EOG8F4VSG	IPR003560	2,3-dihydro-2,3-dihydroxybenzoate dehydrogenase
EOG8DV82W	IPR013604	7TM chemoreceptor
EOG8W6RCS,EOG89360G,EOG8RZ1D8	IPR022242	87kDa Transposase
EOG83N9TJ	IPR000412	ABC-2 transporter
EOG88GZHQ	IPR024371	Acetyl-coenzyme A transporter 1
EOG8RZ1DX	IPR015876	Acyl-CoA desaturase
EOG8S1WN4,EOG8BCH22,EOG873S63	IPR016181	Acyl-CoA N-acyltransferase
EOG83FK9M	IPR014043	Acyl transferase
EOG83FK9M	IPR016035	Acyl transferase/acyl hydrolase/lysophospholipase
EOG8NGK1F	IPR002656	Acyltransferase 3
EOG8D2950	IPR002466	Adenosine deaminase/editase
EOG83FK9M	IPR013149	Alcohol dehydrogenase, C-terminal
EOG8D2950	IPR020471	Aldo/keto reductase

EOG8D2950	IPR018170	Aldo/keto reductase, conserved site
EOG83FK9M	IPR029058	Alpha/Beta hydrolase fold
EOG8TTK15,EOG8STVK8	IPR001616	Alphaherpesvirus alkaline exonuclease
EOG8F4VSG	IPR018764	Anti-sigma K factor RskA
EOG8CJXZG,EOG8CRPGZ	IPR006111	Archaeal RpoK/eukaryotic RPB6 RNA polymerase subunit
EOG89360G,EOG8KSRZK	IPR016024	Armadillo-type fold
EOG8CNT5M	IPR011021	Arrestin-like, N-terminal
EOG8CNT5M	IPR011022	Arrestin C-terminal-like domain
EOG8GTNV8,EOG8DRCSN,EOG8GTNT0,EOG8K0T47,EOG88973C,EOG854CDR,EOG8Z91BB	IPR021109	Aspartic peptidase domain
EOG8GTNV8,EOG8K0T47	IPR001969	Aspartic peptidase, active site
EOG8FJBR5,EOG8SFCMS,EOG8CRPGZ,EOG8Z91BB	IPR017956	AT hook, DNA-binding motif
EOG88D38H	IPR003652	Ataxin, AXH domain
EOG89360G	IPR007663	Baculoviridae p74
EOG873S63	IPR004941	Baculovirus FP protein
EOG8FJBR5	IPR018379	BEN domain
EOG8HDWBC,EOG84QWFZ,EOG8NZX9G,EOG8CRPGZ,EOG8QNQD0,EOG8C5G20	IPR004210	BESS motif
EOG83FK9M	IPR018201	Beta-ketoacyl synthase, active site
EOG83FK9M	IPR014031	Beta-ketoacyl synthase, C-terminal
EOG83FK9M	IPR014030	Beta-ketoacyl synthase, N-terminal
EOG83R628	IPR001370	BIR repeat
EOG8HB12K,EOG83R628	IPR011705	BTB/Kelch-associated
EOG8HB12K,EOG83R628,EOG8F4VRC	IPR000210	BTB/POZ domain
EOG8GJ22Z	IPR000008	C2 domain
EOG8M3CQJ	IPR001715	Calponin homology domain
EOG88SK71	IPR016184	Capsid/spike protein, ssDNA virus
EOG8TXF6D	IPR003010	Carbon-nitrogen hydrolase
EOG80306V	IPR003146	Carboxypeptidase, activation peptide
EOG89360G	IPR001315	CARD domain
EOG847J8K	IPR001071	Cellular retinaldehyde binding/alpha-tocopherol transport
EOG8M3CQJ	IPR010441	CH-like domain in sperm protein
EOG8GTNV8	IPR002423	Chaperonin Cpn60/TCP-1 family
EOG8935ZF	IPR011583	Chitinase II
EOG8S1WN4	IPR007051	CHORD domain
EOG8J6V5R,EOG8Q87B6,EOG8BS00V,EOG8C8B8R	IPR023780	Chromo domain
EOG8J6V5R,EOG8Q87B6,EOG8BS00V,EOG8BCH22,EOG8DNHJQ	IPR016197	Chromo domain-like
EOG8J6V5R,EOG8Q87B6,EOG8BS00V,EOG8BCH22,EOG8XSP61,EOG8C8B8R	IPR000953	Chromo/chromo shadow domain
EOG847J8K	IPR001251	CRAL-TRIO lipid binding domain
EOG847J8K	IPR011074	CRAL/TRIO, N-terminal domain
EOG80P6ND	IPR000095	CRIB domain
EOG88SK71	IPR018490	Cyclic nucleotide-binding-like
EOG88SK71	IPR000595	Cyclic nucleotide-binding domain
EOG8PP0Z9	IPR013763	Cyclin-like

EOG8PP0Z9	IPR004367	Cyclin, C-terminal domain
EOG8PP0Z9	IPR006671	Cyclin, N-terminal
EOG89360G	IPR009069	Cysteine alpha-hairpin motif superfamily
EOG8JDKNM	IPR025661	Cysteine peptidase, asparagine active site
EOG8JDKNM	IPR000169	Cysteine peptidase, cysteine active site
EOG8JDKNM	IPR025660	Cysteine peptidase, histidine active site
EOG83N9TJ	IPR001128	Cytochrome P450
EOG83N9TJ	IPR017972	Cytochrome P450, conserved site
EOG83N9TJ	IPR002401	Cytochrome P450, E-class, group I
EOG83N9TJ	IPR002402	Cytochrome P450, E-class, group II
EOG83N9TJ	IPR002403	Cytochrome P450, E-class, group IV
EOG8JWXV4	IPR022730	DAZ associated protein 2
EOG8NCPTW,EOG82VBX9,EOG8Z91BS,EOG89360G,EOG8B5RMV,EOG854CDX,EOG8BZQGV	IPR004875	DDE superfamily endonuclease domain
EOG8935ZF	IPR011545	DEAD/DEAH box helicase domain
EOG8W3W3W	IPR008181	Deoxyuridine triphosphate nucleotidohydrolase
EOG88973C	IPR005034	Dicer dimerisation domain
EOG88973C	IPR016177	DNA-binding domain
EOG8NCPTW,EOG82VBX9,EOG8Z91BS,EOG8B5RMV,EOG854CDX,EOG8XSP61,EOG8BZQGV	IPR007889	DNA binding HTH domain, Psq-type
EOG8RZ1DV	IPR011010	DNA breaking-rejoining enzyme, catalytic core
EOG8QJV3K,EOG8NCPTW,EOG8NZX7W,EOG87WR48,EOG89360G,EOG84J50X,EOG880MB7,EOG8HB12K,EOG8FR367,EOG8RZ1D8,EOG83R628,EOG808QQS,EOG8CRPGZ,EOG81ZHSB,EOG8FJBPS,EOG8C8B8R	IPR025398	Domain of unknown function DUF4371
EOG8W3W3W	IPR029054	dUTPase-like
EOG88GZHQ	IPR008603	Dynactin subunit 4
EOG84QWFZ	IPR000620	EamA domain
EOG8KPWSW,EOG8SJ7TM	IPR002048	EF-hand domain
EOG8WDGTK	IPR000742	EGF-like domain
EOG8W6RD1	IPR009022	Elongation factor G, domain III
EOG8DBWWP,EOG8JWXV4,EOG8NZX7W,EOG8JT2QV,EOG8W6RD1,EOG8Q5C4Z,EOG8SFCMS,EOG873S63	IPR005135	Endonuclease/exonuclease/phosphatase
EOG8Z91BB	IPR022048	Envelope fusion protein-like
EOG80306V	IPR001873	Epithelial sodium channel
EOG8N3335	IPR002259	Equilibrative nucleoside transporter
EOG8DNHJQ	IPR020579	Exonuclease VII, large subunit, C-terminal
EOG8TTK15,EOG8STVK8	IPR011604	Exonuclease, phage-type/RecB, C-terminal
EOG8S1WN4	IPR013520	Exonuclease, RNase T/DNA polymerase III
EOG89626S	IPR029041	FAD-linked oxidoreductase-like
EOG870X00	IPR029214	FAM183 family
EOG8RZ1DX	IPR005804	Fatty acid desaturase domain
EOG8RZ1DX	IPR001522	Fatty acid desaturase type 1, conserved site
EOG83FK9M	IPR023102	Fatty acid synthase, domain 2
EOG8DV82W	IPR026055	Fatty acyl-CoA reductase
EOG83N9TJ	IPR000885	Fibrillar collagen, C-terminal

EOG8DNHJQ	IPR002181	Fibrinogen, alpha/beta/gamma chain, C-terminal globular domain
EOG8DNHJQ	IPR014716	Fibrinogen, alpha/beta/gamma chain, C-terminal globular, subdomain 1
EOG8DNHJQ	IPR014715	Fibrinogen, alpha/beta/gamma chain, C-terminal globular, subdomain 2
EOG8DNHJQ	IPR020837	Fibrinogen, conserved site
EOG8QZB47,EOG8S7N55,EOG8KSRZK	IPR003961	Fibronectin type III
EOG8F4VRC	IPR027705	Flotillin family
EOG80306V	IPR000276	G protein-coupled receptor, rhodopsin-like
EOG80P6ND	IPR006605	G2 nidogen/fibulin G2F
EOG88973C	IPR025724	GAG-pre-integrase domain
EOG8Z91BB	IPR004957	Gag polyprotein
EOG8HDWBC	IPR008979	Galactose-binding domain-like
EOG83R628	IPR015916	Galactose oxidase, beta-propeller
EOG85MQN0	IPR000305	GIY-YIG nuclease superfamily
EOG85F05D	IPR004046	Glutathione S-transferase, C-terminal
EOG85TG3K,EOG85F05D,EOG8BCH22	IPR010987	Glutathione S-transferase, C-terminal-like
EOG85TG3K,EOG85F05D,EOG8BCH22	IPR004045	Glutathione S-transferase, N-terminal
EOG85F05D	IPR006194	Glycine-tRNA synthetase, heterodimeric
EOG8935ZF	IPR001223	Glycoside hydrolase family 18, catalytic domain
EOG8935ZF	IPR017853	Glycoside hydrolase superfamily
EOG8F4VSG	IPR000743	Glycoside hydrolase, family 28
EOG8BCH22	IPR007235	Glycosyl transferase, family 28, C-terminal
EOG8BCH22	IPR004276	Glycosyltransferase family 28, N-terminal domain
EOG8BCH22,EOG873S63	IPR000182	GNAT domain
EOG8JMB63	IPR009038	GOLD domain
EOG80306V	IPR017452	GPCR, rhodopsin-like, 7TM
EOG83FK9M	IPR011032	GroES-like
EOG8W9MJT	IPR023214	HAD-like domain
EOG8QNQD0,EOG8F4VSG	IPR010562	Haemolymph juvenile hormone binding
EOG8Q87B6,EOG8PP0ZK,EOG8Z0DNN,EOG808QQS	IPR027806	Harbinger transposase-derived nuclease domain
EOG8QJV3K,EOG8NCPTW,EOG8W6RCS,EOG8PP0ZK,EOG8NZX7W,EOG87WR48,EOG8FXTNG,EOG89360G,EOG84J50X,EOG880MB7,EOG8HB12K,EOG8FR367,EOG8RZ1D8,EOG8CJXZG,EOG83R628,EOG834ZPV,EOG88SK71,EOG8QNQD0,EOG81ZHSB,EOG8Z91BB,EOG8XKXNP	IPR008906	HAT, C-terminal dimerisation domain
EOG8JWXV4,EOG8935ZF	IPR014001	Helicase superfamily 1/2, ATP-binding domain
EOG8935ZF	IPR001650	Helicase, C-terminal
EOG8BCH22	IPR002717	Histone acetyltransferase domain, MYST-type
EOG8NCPTW,EOG8W6RD1,EOG82VBX9,EOG8Z91BS,EOG89360G,EOG8B5RMV,EOG854CDX,EOG8XSP61,EOG8W3W40,EOG8BZQGV	IPR009057	Homeobox domain-like
EOG8NCPTW,EOG8W6RD1,EOG8Z91BS,EOG8B5RMV,EOG854CDX,EOG8XSP61,EOG8BZQGV	IPR006600	HTH CenpB-type DNA-binding domain
EOG8KSRZK	IPR013151	Immunoglobulin
EOG8Q87B6,EOG8S7N55,EOG854CDX,EOG8KSRZK	IPR007110	Immunoglobulin-like domain
EOG8S7N55,EOG8KSRZK	IPR013783	Immunoglobulin-like fold

EOG8CNT5M	IPR014756	Immunoglobulin E-set
EOG8KSRZK	IPR013098	Immunoglobulin I-set
EOG8S7N55,EOG8KSRZK	IPR003599	Immunoglobulin subtype
EOG8KSRZK	IPR003598	Immunoglobulin subtype 2
EOG89360G	IPR001494	Importin-beta, N-terminal domain
EOG8QJV4S,EOG8DNHJQ	IPR000618	Insect cuticle protein
EOG8RZ1DV	IPR013762	Integrase-like, catalytic domain
EOG8DV82W,EOG8J6V5R,EOG8GTNV8,EOG8QJV3K,EOG8DRCSN,EOG89360G,EOG8GTNT0,EOG8K0T47,EOG88973C,EOG8XSP61,EOG8MKQZ3,EOG8Z91BB,EOG8C8B8R	IPR001584	Integrase, catalytic core
EOG8RZ1DV	IPR002104	Integrase, catalytic domain
EOG83N9TJ	IPR006141	Intein N-terminal splicing region
EOG8GTNV8	IPR001664	Intermediate filament protein
EOG8SJ7TM,EOG88SK71	IPR005821	Ion transport domain
EOG80P6ND	IPR000048	IQ motif, EF-hand binding site
EOG8W9MJT	IPR008949	Isoprenoid synthase domain
EOG8HB12K	IPR024445	ISXO2-like transposase domain
EOG8Q87B6	IPR002350	Kazal domain
EOG8JWXV4	IPR015915	Kelch-type beta propeller
EOG8HB12K,EOG83R628	IPR006652	Kelch repeat type 1
EOG80P6ND	IPR003386	Lecithin:cholesterol/phospholipid:diacylglycerol acyltransferase
EOG80306V	IPR015819	Lipid transport protein, beta-sheet shell
EOG80306V	IPR001747	Lipid transport protein, N-terminal
EOG8DV82W	IPR010920	LSM domain
EOG8DV82W	IPR001163	LSM domain, eukaryotic/archaea-type
EOG81C99V	IPR000751	M-phase inducer phosphatase
EOG8HDWBC,EOG84QWFZ,EOG8NZX9G,EOG8QNQD0,EOG8C5G20	IPR006578	MADF domain
EOG80P6ND,EOG8BCH22,EOG8R2686	IPR011701	Major facilitator superfamily
EOG8CNT5M,EOG80P6ND,EOG8R2686	IPR020846	Major facilitator superfamily domain
EOG8CNT5M,EOG80P6ND	IPR005828	Major facilitator, sugar transporter-like
EOG8DV82W	IPR013120	Male sterility, NAD-binding
EOG83FK9M	IPR016036	Malonyl-CoA ACP transacylase, ACP-binding
EOG8DNHJQ	IPR019466	Matrilin, coiled-coil trimerisation domain
EOG8Q87B6	IPR003604	Matrin/U1-C-like, C2H2-type zinc finger
EOG8WSZRB	IPR019087	Mediator of RNA polymerase II transcription subunit 15
EOG88973C	IPR001739	Methyl-CpG DNA binding
EOG89626S	IPR003171	Methylenetetrahydrofolate reductase
EOG8ZKN28	IPR025714	Methyltransferase domain
EOG8ZKN28	IPR013216	Methyltransferase type 11
EOG8ZKN28	IPR013217	Methyltransferase type 12
EOG84J50X	IPR011057	Mss4-like
EOG8QJV3K,EOG84J50X,EOG8QNQD0,EOG8FR38B,EOG8C8B8R	IPR018289	MULE transposase domain
EOG8Q87B6	IPR011031	Multihaem cytochrome

EOG8HDWBC,EOG89360G	IPR017877	Myb-like domain
EOG8W3W40	IPR001609	Myosin head, motor domain
EOG8DV82W,EOG8JWXV4,EOG83FK9M,EOG8F4VSG	IPR016040	NAD(P)-binding domain
EOG8D2950	IPR023210	NADP-dependent oxidoreductase domain
EOG8DNHJQ	IPR007007	Ninjurin
EOG89360G	IPR000536	Nuclear hormone receptor, ligand-binding domain
EOG8F4VRC	IPR004255	O-acyltransferase, WSD1, N-terminal
EOG8Q5C4Z,EOG8RZ1DX	IPR004117	Olfactory receptor, insect
EOG8DV82W	IPR003421	Opine dehydrogenase
EOG8PP0ZK	IPR018020	Oxo-4-hydroxy-4-carboxy-5-ureidoimidazoline decarboxylase
EOG8JWXV4,EOG8935ZF	IPR027417	P-loop containing nucleoside triphosphate hydrolase
EOG84J50X	IPR001429	P2X purinoreceptor
EOG84QWH7	IPR008967	p53-like transcription factor, DNA-binding
EOG84QWH7	IPR002117	p53 tumour suppressor family
EOG84QWH7	IPR011615	p53, DNA-binding domain
EOG84QWH7	IPR010991	p53, tetramerisation domain
EOG84QWH7	IPR012346	p53/RUNT-type transcription factor, DNA-binding domain
EOG88SK71	IPR001610	PAC motif
EOG8F4VSG	IPR006626	Parallel beta-helix repeat
EOG8GF5X2,EOG8WDGTK	IPR013607	Parvovirus coat protein VP1, N-terminal
EOG88SK71	IPR000700	PAS-associated, C-terminal
EOG88SK71	IPR000014	PAS domain
EOG8RZ1DV	IPR003100	PAZ domain
EOG8GJ22Z	IPR001478	PDZ domain
EOG8F4VSG	IPR012334	Pectin lyase fold
EOG8F4VSG	IPR011050	Pectin lyase fold/virulence factor
EOG8DRCSN,EOG8K0T47	IPR001995	Peptidase A2A, retrovirus, catalytic
EOG8JDKNM	IPR013128	Peptidase C1A
EOG8JDKNM	IPR015643	Peptidase C1A, cathepsin B
EOG8JDKNM	IPR000668	Peptidase C1A, papain C-terminal
EOG8JDKNM	IPR012599	Peptidase C1A, propeptide
EOG8CZF0X	IPR014782	Peptidase M1, membrane alanine aminopeptidase, N-terminal
EOG80306V,EOG80P6ND	IPR000834	Peptidase M14, carboxypeptidase A
EOG80306V,EOG8BCH22,EOG8ZKN28,EOG8F4VSG	IPR009003	Peptidase S1, PA clan
EOG84J50X	IPR002579	Peptide methionine sulphoxide reductase MrsB
EOG81C99V	IPR008914	Phosphatidylethanolamine-binding protein
EOG8NZX7W	IPR002498	Phosphatidylinositol-4-phosphate 5-kinase, core
EOG8DV82W	IPR002123	Phospholipid/glycerol acyltransferase
EOG84J50X,EOG83FK9M	IPR009081	Phosphopantetheine binding ACP domain
EOG83N9TJ	IPR001683	Phox homologous domain
EOG89626S	IPR013717	PIG-P

EOG8BGCB3,EOG8P8J0G,EOG8KPWTR,EOG8FJBR5,EOG80S2Z1,EOG8CJXZG,EOG8T4GB8,EOG8XKXP3,EOG8CRPGZ,EOG85MQNM,EOG8W3W40,EOG8XKXNP	IPR029526	PiggyBac transposable element-derived protein
EOG8HDWB0	IPR029060	PIN domain-like
EOG8RZ1DV	IPR003165	Piwi domain
EOG8F4VSG	IPR009440	Plasmid segregation protein ParM/StbA
EOG8BCH22	IPR001849	Pleckstrin homology domain
EOG83FK9M	IPR020801	Polyketide synthase, acyl transferase domain
EOG83FK9M	IPR020841	Polyketide synthase, beta-ketoacyl synthase domain
EOG83FK9M	IPR020807	Polyketide synthase, dehydratase domain
EOG83FK9M	IPR020843	Polyketide synthase, enoylreductase domain
EOG83FK9M	IPR013968	Polyketide synthase, ketoreductase domain
EOG83FK9M	IPR020806	Polyketide synthase, phosphopantetheine-binding domain
EOG8W9MJT	IPR000092	Polyprenyl synthetase
EOG88SK71	IPR013099	Potassium channel domain
EOG88SK71	IPR003938	Potassium channel, voltage-dependent, EAG/ELK/ERG
EOG88SK71	IPR003950	Potassium channel, voltage-dependent, ELK
EOG8W6RD1	IPR006579	Pre-C2HC domain
EOG80306V	IPR009020	Protease propeptides/proteinase inhibitor I9
EOG8PP0Z9,EOG80P6ND,EOG8RZ1D8,EOG8KSRZK,EOG83N9TJ	IPR011009	Protein kinase-like domain
EOG8Q87B6,EOG873S63	IPR002219	Protein kinase C-like, phorbol ester/diacylglycerol-binding domain
EOG8PP0Z9,EOG80P6ND,EOG8RZ1D8,EOG8KSRZK	IPR000719	Protein kinase domain
EOG8KSRZK	IPR017441	Protein kinase, ATP binding site
EOG8FJBR5,EOG808QQS	IPR005312	Protein of unknown function DUF1759
EOG8VMHZ0	IPR028006	Protein of unknown function DUF4570
EOG8BCH22	IPR007999	Protein of unknown function DUF745
EOG80P6ND	IPR018625	Protein Pet100
EOG8JHFXB	IPR001901	Protein translocase complex, SecE/Sec61-gamma subunit
EOG8JHFXB	IPR023391	Protein translocase SecE domain
EOG8S7N55	IPR000242	PTP type protein phosphatase
EOG8BS00V,EOG8Z91BB	IPR000313	PWWP domain
EOG8RZ1D8	IPR015793	Pyruvate kinase, barrel
EOG8HB12K,EOG8B2WBM	IPR001374	R3H domain
EOG8GJ22Z	IPR010911	Rab-binding domain
EOG8QNQD0	IPR019018	Rab-binding domain FIP-RBD
EOG8S1WNX	IPR019568	Rapsyn, myristoylation/linker region, N-terminal
EOG8TTK15,EOG8STVK8,EOG8G4K6Z	IPR011335	Restriction endonuclease type II-like
EOG8GTNV8,EOG8DRCSN,EOG8GTNT0,EOG8K0T47,EOG88973C,EOG854CDR,EOG8Z91BB	IPR018061	Retropepsins
EOG8GTNV8,EOG8DRCSN,EOG8K0T47,EOG88973C,EOG854CDR,EOG8Z91BB	IPR005162	Retrotransposon gag domain
EOG8GTNV8,EOG83R628	IPR008042	Retrotransposon, Pao
EOG8GTNV8,EOG88973C	IPR008916	Retrovirus capsid, C-terminal

EOG8DBWWP,EOG8GTNV8,EOG8QJV3K,EOG8BS00V,EOG8NZX7W,EOG8W6RD1,EOG8DRCSN,EOG89360G,EOG880MB7,EOG8GTNT0,EOG8HB12K,EOG8Q5C4Z,EOG8FR367,EOG8RZ1D8,EOG873S63,EOG8RZ1DV,EOG808QQS,EOG8QNQD0,EOG8Z91BB	IPR000477	Reverse transcriptase domain
EOG8W6RD1,EOG88973C,EOG808QQS	IPR013103	Reverse transcriptase, RNA-dependent DNA polymerase
EOG83N9TJ	IPR000198	Rho GTPase-activating protein domain
EOG83N9TJ	IPR008936	Rho GTPase activation protein
EOG81C99V	IPR001763	Rhodanese-like domain
EOG8Q87B6	IPR008805	RIB43A
EOG8BGCB3,EOG8DV82W,EOG8J6V5R,EOG8Q87B6,EOG8GTNV8,EOG8QJV3K,EOG8NCPTW,EOG8W6RCS,EOG8JWXV4,EOG8PP0ZK,EOG8NZX7W,EOG87WR48,EOG8W6RD1,EOG8FXTNG,EOG8DRCSN,EOG89360G,EOG84J50X,EOG880MB7,EOG8GTNT0,EOG8K0T47,EOG8HB12K,EOG8XWJFT,EOG8SFCMS,EOG8S1WN4,EOG8FR367,EOG8N5ZD2,EOG83R62R,EOG8RZ1D8,EOG8CJXZG,EOG83R628,EOG8RZ1DV,EOG88973C,EOG834ZPV,EOG88SK71,EOG8QNQD0,EOG8XSP61,EOG8MKQZ3,EOG81ZHSB,EOG8Z91BB,EOG8XKXNP,EOG8C8B8R	IPR012337	Ribonuclease H-like domain
EOG8JWXV4,EOG8W6RD1,EOG83R62R	IPR002156	Ribonuclease H domain
EOG8ZCWNB	IPR027534	Ribosomal protein L12 family
EOG8JMB63	IPR005484	Ribosomal protein L18
EOG8GTNV8	IPR016082	Ribosomal protein L30, ferredoxin-like fold domain
EOG82589B,EOG8P8HZZ	IPR001865	Ribosomal protein S2
EOG8P8HZZ	IPR018130	Ribosomal protein S2, conserved site
EOG8P8HZZ	IPR027498	Ribosomal protein S2, eukaryotic
EOG8P8HZZ	IPR005707	Ribosomal protein S2, eukaryotic/archaeal
EOG82589B,EOG8P8HZZ	IPR023591	Ribosomal protein S2, flavodoxin-like domain
EOG8W6RD1	IPR020568	Ribosomal protein S5 domain 2-type fold
EOG8W6RD1	IPR014721	Ribosomal protein S5 domain 2-type fold, subgroup
EOG873S63	IPR000772	Ricin B, lectin domain
EOG85HVHB,EOG88SK71	IPR014710	RmlC-like jelly roll fold
EOG8BCH22	IPR025995	RNA binding activity-knot of a chromodomain
EOG8935ZF	IPR014014	RNA helicase, DEAD-box type, Q motif
EOG8DV82W,EOG8JHFXB,EOG8S1WN4,EOG8DNHJQ	IPR000504	RNA recognition motif domain
EOG89360G	IPR024934	Rubredoxin-like domain
EOG873S63	IPR018527	Rubredoxin, iron-binding site
EOG8ZKN28	IPR029063	S-adenosyl-L-methionine-dependent methyltransferase
EOG8RZ1D8,EOG8RZ1DV,EOG8Z91BB	IPR003034	SAP domain
EOG88973C	IPR003309	SCAN domain
EOG80306V,EOG8BCH22,EOG8ZKN28,EOG8F4VSG	IPR001254	Serine proteases, trypsin domain
EOG8KSRZK	IPR008271	Serine/threonine-protein kinase, active site
EOG80306V,EOG8RZ1DV,EOG8TXF6D	IPR001214	SET domain
EOG83FK9M	IPR000980	SH2 domain
EOG8F4VSG	IPR002347	Short-chain dehydrogenase/reductase SDR
EOG8F4VSG	IPR020904	Short-chain dehydrogenase/reductase, conserved site
EOG8HB12K,EOG83R628,EOG8F4VRC	IPR011333	SKP1/BTB/POZ domain
EOG8STVK8,EOG873S63	IPR010989	SNARE

EOG8BCH22	IPR005024	Snf7 family
EOG8HB12X	IPR009846	Splicing factor 3B subunit 5/RDS3 complex subunit 10
EOG8HB12X	IPR017089	Splicing factor 3B, subunit 5
EOG8Q5C4Z	IPR001190	SRCR domain
EOG8BS00V	IPR013761	Sterile alpha motif/pointed domain
EOG80P6ND	IPR005829	Sugar transporter, conserved site
EOG80P6ND	IPR003663	Sugar/inositol transporter
EOG8STVK8	IPR000727	Target SNARE coiled-coil homology domain
EOG83FK9M	IPR013625	Tensin/EPS8 phosphotyrosine-binding domain
EOG8S1WNX	IPR013026	Tetratricopeptide repeat-containing domain
EOG8S7N55	IPR000594	THIF-type NAD/FAD binding fold
EOG83FK9M	IPR001031	Thioesterase
EOG83FK9M	IPR016039	Thiolase-like
EOG85TG3K,EOG85F05D,EOG8BCH22	IPR012336	Thioredoxin-like fold
EOG81C99V	IPR001307	Thiosulphate sulfurtransferase, conserved site
EOG8DNHJQ	IPR000884	Thrombospondin type-1 (TSP1) repeat
EOG83R628	IPR007725	Timeless C-terminal
EOG873S63	IPR000157	Toll/interleukin-1 receptor homology (TIR) domain
EOG8S1WNX	IPR019167	Topoisomerase II-associated protein PAT1
EOG84QWH7	IPR024631	Transcription factor p53, C-terminal, Drosophila
EOG8S4RZ6	IPR003923	Transcription initiation factor TFIID, 23-30kDa subunit
EOG8BZQGV	IPR002514	Transposase IS3/IS911family
EOG8W6RCS,EOG89360G,EOG8RZ1D8	IPR021896	Transposase protein
EOG8PP0ZK,EOG89360G,EOG8Z0DNN,EOG808QQS	IPR027805	Transposase, Helix-turn-helix domain
EOG8BS00V	IPR004242	Transposon, En/Spm-like
EOG8Q87B6	IPR011017	TRASH domain
EOG8S1WN4	IPR004686	Tricarboxylate/iron carrier
EOG8JWXV4	IPR011004	Trimeric LpxA-like
EOG8ZCWNB	IPR001859	Trypanosoma cruzi ribosomal protein P2-like
EOG8C8B8R	IPR004567	Type II pantothenate kinase
EOG80P6ND,EOG8RZ1D8,EOG8KSRZK	IPR020635	Tyrosine-protein kinase, catalytic domain
EOG83N9TJ	IPR004147	UbiB domain
EOG8BCH22	IPR002213	UDP-glucuronosyl/UDP-glucosyltransferase
EOG8BCH22	IPR006326	UDP-glycosyltransferase, MGT
EOG84J50X	IPR003653	Ulp1 protease family, C-terminal catalytic domain
EOG80306V	IPR015816	Vitellinogen, beta-sheet N-terminal
EOG80306V	IPR015255	Vitellinogen, open beta-sheet
EOG80306V	IPR011030	Vitellinogen, superhelical
EOG8SJ7TM	IPR002077	Voltage-dependent calcium channel, alpha-1 subunit
EOG8SJ7TM	IPR014873	Voltage-dependent calcium channel, alpha-1 subunit, IQ domain

EOG8SJ7TM	IPR005446	Voltage-dependent calcium channel, L-type, alpha-1 subunit
EOG8SJ7TM	IPR005448	Voltage-dependent calcium channel, P/Q-type, alpha-1 A
EOG80306V	IPR001846	von Willebrand factor, type D domain
EOG8S1WN4	IPR017986	WD40-repeat-containing domain
EOG8S1WN4	IPR001680	WD40 repeat
EOG8S1WN4	IPR015943	WD40/YVTN repeat-like-containing domain
EOG8VMHXG	IPR008893	WGR domain
EOG8W6RD1	IPR001202	WW domain
EOG8TTK15,EOG8STVK8,EOG8G4K6Z	IPR019080	YqaJ viral recombinase
EOG8Q87B6,EOG86T5J0,EOG84QWFZ	IPR013087	Zinc finger C2H2-type
EOG8Q87B6,EOG89360G,EOG84QWFZ	IPR012934	Zinc finger, AD-type
EOG8Q87B6,EOG84J50X,EOG8GTNT0	IPR003656	Zinc finger, BED-type
EOG8S1WN4	IPR001562	Zinc finger, Btk motif
EOG8W6RCS,EOG8PP0ZK,EOG8W6RD1,EOG8TTK15,EOG89360G,EOG84J50X,EOG8CNT5M,EOG8Z0DNN,EOG8HB12K,EOG8RZ1D8,EOG808QQS,EOG8CRPGZ,EOG8QNQD9,EOG8C8B8R	IPR006612	Zinc finger, C2CH-type
EOG8S1WN4	IPR000571	Zinc finger, CCCH-type
EOG8DBWWP,EOG8DV82W,EOG8GTNV8,EOG8W6RD1,EOG82589B,EOG8DRCSN,EOG8GTNT0,EOG8K0T47,EOG8Q5C4Z,EOG8SFCMS,EOG86HJTC,EOG8BCH22,EOG8RZ1D8,EOG8CJXZG,EOG88973C,EOG854CDR,EOG8SJ7TM,EOG8Z91BB,EOG8C8B8R	IPR001878	Zinc finger, CCHC-type
EOG8Q87B6,EOG85MQN0	IPR022755	Zinc finger, double-stranded RNA binding
EOG8CRPGZ,EOG88SK71,EOG8W3W40,EOG8FR38B	IPR007588	Zinc finger, FLYWCH-type
EOG8GJ22Z	IPR017455	Zinc finger, FYVE-related
EOG8QZB47,EOG8JWXV4,EOG8TTK15,EOG8STVK8,EOG873S63,EOG854CDX,EOG8GJ22Z	IPR011011	Zinc finger, FYVE/PHD-type
EOG8Q87B6	IPR015318	Zinc finger, GAGA-binding factor
EOG8GTNT0,EOG8Z91BB	IPR015416	Zinc finger, H2C2-type, histone UAS binding
EOG8Q87B6	IPR001781	Zinc finger, LIM-type
EOG80306V,EOG80P6ND,EOG8BZQGV,EOG8TXF6D	IPR002893	Zinc finger, MYND-type
EOG8W6RCS	IPR001628	Zinc finger, nuclear hormone receptor-type
EOG8QZB47,EOG8STVK8,EOG873S63	IPR019787	Zinc finger, PHD-finger
EOG8QZB47,EOG8Q87B6,EOG8STVK8,EOG873S63	IPR001965	Zinc finger, PHD-type
EOG873S63	IPR001841	Zinc finger, RING-type
EOG8HB12K,EOG873S63,EOG8GJ22Z	IPR013083	Zinc finger, RING/FYVE/PHD-type
EOG8STVK8,EOG8FJBR5,EOG808QQS,EOG8G4K6Z	IPR007527	Zinc finger, SWIM-type
EOG8NCPTW,EOG89360G,EOG880MB7,EOG8RZ1D8,EOG83R628,EOG8CRPGZ	IPR006580	Zinc finger, TTF-type

Table 3S. Gene ontology classification of rapidly changing gene families in the Leptinotarsa decemlineata lineage.

GO ID	GO Definition	# of families with GO ID	OrthoDB family IDs
GO:0003676	nucleic acid binding	56	EOG8BGCB3,EOG8DV82W,EOG8J6V5R,EOG8Q87B6,EOG8GTNV8,EOG8QJV3K,EOG8NCPTW,EOG8W6RCS,EOG8JHFXB,EOG8PP0ZK,EOG8S1WN4,EOG8NZX7W,EOG87WR48,EOG8W6RD1,EOG86T5J0,EOG82589B,EOG8FXTNG,EOG8DRCSN,EOG89360G,EOG84J50X,EOG8MKQZ3,EOG8GTNT0,EOG85HVHB,EOG8Z0DNN,EOG8K0T47,EOG8HB12K,EOG8XWJFT,EOG8935ZF,EOG8Q5C4Z,EOG8SFCMS,EOG86HJTC,EOG8BCH22,EOG8FR367,EOG8B2WBM,EOG8N5ZD2,EOG83R62R,EOG8RZ1D8,EOG8CJXZG,EOG83R628,EOG8RZ1DV,EOG88973C,EOG854CDX,EOG808QQS,EOG834ZPV,EOG8CRPGZ,EOG88SK71,EOG8QNQD0,EOG8XSP61,EOG8BZQGV,EOG880MB7,EOG81ZHSB,EOG8DNHJQ,EOG8Z91BB,EOG8XKXNP,EOG8FJBPS,EOG8C8B8R
GO:0008270	zinc ion binding	26	EOG8QZB47,EOG8Q87B6,EOG8GTNV8,EOG8W6RCS,EOG8W6RD1,EOG80306V,EOG82589B,EOG8DRCSN,EOG89360G,EOG8STVK8,EOG8FJBR5,EOG8GTNT0,EOG8K0T47,EOG8Q5C4Z,EOG8SFCMS,EOG86HJTC,EOG8BCH22,EOG8RZ1D8,EOG873S63,EOG88973C,EOG854CDX,EOG808QQS,EOG83FK9M,EOG8Z91BB,EOG8CZF0X,EOG8C8B8R
GO:0003677	DNA binding	21	EOG8NCPTW,EOG8W6RD1,EOG8TTK15,EOG89360G,EOG84J50X,EOG8STVK8,EOG8B5RMV,EOG8FJBR5,EOG84QWFZ,EOG8SFCMS,EOG8CJXZG,EOG8RZ1DV,EOG88973C,EOG854CDX,EOG8G4K6Z,EOG8CRPGZ,EOG8QNQD0,EOG8XSP61,EOG8BZQGV,EOG8Z91BB,EOG8C5G20
GO:0003723	RNA binding	19	EOG8DBWWP,EOG8GTNV8,EOG8QJV3K,EOG8BS00V,EOG8NZX7W,EOG8W6RD1,EOG8DRCSN,EOG89360G,EOG8D2950,EOG8HB12K,EOG8935ZF,EOG8Q5C4Z,EOG8FR367,EOG8RZ1D8,EOG8RZ1DV,EOG88D38H,EOG8QNQD0,EOG880MB7,EOG8Z91BB
GO:0005634	nucleus	17	EOG8J6V5R,EOG8Q87B6,EOG8W6RCS,EOG8JHFXB,EOG8W3W3W,EOG8PP0Z9,EOG8TTK15,EOG8P8HZZ,EOG89360G,EOG8STVK8,EOG81C99V,EOG8935ZF,EOG83R628,EOG88973C,EOG8S4RZ6,EOG84QWH7,EOG8C8B8R
GO:0003964	RNA-directed DNA polymerase activity	17	EOG8DBWWP,EOG8GTNV8,EOG8QJV3K,EOG8BS00V,EOG8NZX7W,EOG8W6RD1,EOG8DRCSN,EOG89360G,EOG8HB12K,EOG8Q5C4Z,EOG8FR367,EOG8RZ1D8,EOG8RZ1DV,EOG8QNQD0,EOG880MB7,EOG8Z91BB,EOG85MQN0
GO:0046872	metal ion binding	16	EOG8Q87B6,EOG8S1WN4,EOG86T5J0,EOG89360G,EOG84J50X,EOG8D2950,EOG85HVHB,EOG8RZ1D8,EOG873S63,EOG88SK8F,EOG83R628,EOG8SJ7TM,EOG85MQN0,EOG8FJBPS,EOG8GJ22Z,EOG8C8B8R
GO:0016021	integral component of membrane	11	EOG8DV82W,EOG80306V,EOG8CNT5M,EOG8N3335,EOG80P6ND,EOG8RZ1DX,EOG88GZHQ,EOG8SJ7TM,EOG88SK71,EOG8R2686,EOG8JMB63
GO:0015074	DNA integration	10	EOG8J6V5R,EOG8GTNV8,EOG8QJV3K,EOG8DRCSN,EOG8MKQZ3,EOG8GTNT0,EOG8RZ1DV,EOG8XSP61,EOG8Z91BB,EOG8C8B8R
GO:0005737	cytoplasm	7	EOG8S1WNX,EOG8GTNV8,EOG8JHFXB,EOG8W3W3W,EOG8PP0Z9,EOG8S4RZ6,EOG83N9TJ
GO:0005524	ATP binding	6	EOG8GTNV8,EOG84J50X,EOG8935ZF,EOG8RZ1D8,EOG8KSRZK,EOG8C8B8R
GO:0000166	nucleotide binding	5	EOG8DV82W,EOG8JHFXB,EOG8S1WN4,EOG8XSP61,EOG8DNHJQ
GO:0004190	aspartic-type endopeptidase activity	5	EOG8GTNV8,EOG8DRCSN,EOG8K0T47,EOG88973C,EOG8Z91BB
GO:0016020	membrane	5	EOG8JHFXB,EOG84J50X,EOG80P6ND,EOG873S63,EOG88SK71
GO:0005515	protein binding	5	EOG80306V,EOG8PP0Z9,EOG81C99V,EOG8S4RZ6,EOG84QWH7
GO:0006351	transcription, DNA-templated	5	EOG89360G,EOG8CJXZG,EOG8CRPGZ,EOG84QWH7,EOG8Z91BB
GO:0005622	intracellular	5	EOG847J8K,EOG89360G,EOG81C99V,EOG8Z91BB,EOG83

			N9TJ
GO:0003735	structural constituent of ribosome	4	EOG82589B,EOG8P8HZZ,EOG8ZCWNB,EOG8JMB63
GO:0016740	transferase activity	4	EOG85F05D,EOG8BCH22,EOG8W9MJT,EOG83FK9M
GO:0003700	transcription factor activity, sequence-specific DNA binding	4	EOG8W6RCS,EOG89360G,EOG88973C,EOG84QWH7
GO:0005198	structural molecule activity	4	EOG8Q87B6,EOG8GTNV8,EOG8GF5X2,EOG8WDGTK
GO:0006909	phagocytosis	3	EOG8DV82W,EOG8CNT5M,EOG8RZ1DX
GO:0007052	mitotic spindle organization	3	EOG8P8HZZ,EOG8935ZF,EOG8HB12X
GO:0005506	iron ion binding	3	EOG89360G,EOG8RZ1DX,EOG83N9TJ
GO:0003899	DNA-directed RNA polymerase activity	3	EOG8CJXZG,EOG8CRPGZ,EOG8Z91BB
GO:0006412	translation	3	EOG82589B,EOG8P8HZZ,EOG8JMB63
GO:0007165	signal transduction	3	EOG8CNT5M,EOG873S63,EOG83N9TJ
GO:0016491	oxidoreductase activity	3	EOG8D2950,EOG83FK9M,EOG8F4VSG
GO:0004523	RNA-DNA hybrid ribonuclease activity	3	EOG8W6RD1,EOG83R62R,EOG8Z91BB
GO:0005811	lipid particle	3	EOG8RZ1DX,EOG83FK9M,EOG83N9TJ
GO:0000398	mRNA splicing, via spliceosome	3	EOG8JHFXB,EOG8935ZF,EOG8HB12X
GO:0004518	nuclease activity	3	EOG8TTK15,EOG8STVK8,EOG8G4K6Z
GO:0019028	viral capsid	3	EOG8GF5X2,EOG8WDGTK,EOG8Z91BB
GO:0005840	ribosome	3	EOG8P8HZZ,EOG8ZCWNB,EOG8JMB63
GO:0006886	intracellular protein transport	2	EOG89360G,EOG8GJ22Z
GO:0006629	lipid metabolic process	2	EOG8DV82W,EOG83N9TJ
GO:0007498	mesoderm development	2	EOG81C99V,EOG8M3CQJ
GO:0005875	microtubule associated complex	2	EOG8P8HZZ,EOG8935ZF
GO:0043565	sequence-specific DNA binding	2	EOG8W6RCS,EOG84QWH7
GO:0000281	mitotic cytokinesis	2	EOG8PP0Z9,EOG8RZ1DX
GO:0006974	cellular response to DNA damage stimulus	2	EOG8PP0Z9,EOG84QWH7
GO:0005615	extracellular space	2	EOG80306V,EOG8QJV4S
GO:0007275	multicellular organismal development	2	EOG8P8HZZ,EOG8SJ7TM
GO:0007619	courtship behavior	2	EOG8RZ1DX,EOG8SJ7TM
GO:0000086	G2/M transition of mitotic cell cycle	2	EOG8PP0Z9,EOG81C99V
GO:0016747	transferase activity, transferring acyl groups other than amino-acyl groups	2	EOG83FK9M,EOG8NGK1F
GO:0004316	3-oxoacyl-[acyl-carrier-protein] reductase (NADPH) activity	2	EOG83FK9M,EOG8F4VSG
GO:0016568	chromatin modification	2	EOG8TTK15,EOG8STVK8
GO:0022891	substrate-specific transmembrane transporter activity	2	EOG8CNT5M,EOG80P6ND
GO:0004527	exonuclease activity	2	EOG8S1WN4,EOG8STVK8
GO:0048786	presynaptic active zone	2	EOG8SJ7TM,EOG8GJ22Z
GO:0004181	metallocarboxypeptidase activity	2	EOG80306V,EOG8BCH22

GO:0004672	protein kinase activity	2	EOG8RZ1D8,EOG8KSRZK
GO:0005216	ion channel activity	2	EOG84J50X,EOG8SJ7TM
GO:0007269	neurotransmitter secretion	2	EOG8SJ7TM,EOG8GJ22Z
GO:0008340	determination of adult lifespan	2	EOG8DV82W,EOG84QWH7
GO:0022008	neurogenesis	2	EOG80306V,EOG8F4VSG
GO:0003707	steroid hormone receptor activity	2	EOG8W6RCS,EOG89360G
GO:0016192	vesicle-mediated transport	2	EOG873S63,EOG8GJ22Z
GO:0000785	chromatin	2	EOG8JHFXB,EOG84QWH7
GO:0008168	methyltransferase activity	2	EOG8BCH22,EOG8ZKN28
GO:0009987	cellular process	2	EOG8PP0Z9,EOG81C99V
GO:0000381	regulation of alternative mRNA splicing, via spliceosome	2	EOG8JHFXB,EOG8935ZF
GO:0048477	oogenesis	2	EOG8JHFXB,EOG84QWH7
GO:0015935	small ribosomal subunit	2	EOG82589B,EOG8P8HZZ
GO:0031410	cytoplasmic vesicle	2	EOG8CNT5M,EOG8RZ1DX
GO:0071011	precatalytic spliceosome	2	EOG8JHFXB,EOG8HB12X
GO:0006406	mRNA export from nucleus	2	EOG8JHFXB,EOG8935ZF
GO:0005576	extracellular region	2	EOG8Q87B6,EOG80306V
GO:0022627	cytosolic small ribosomal subunit	1	EOG8P8HZZ
GO:0048102	autophagic cell death	1	EOG8JDKNM
GO:0008069	dorsal/ventral axis specification, ovarian follicular epithelium	1	EOG8JHFXB
GO:0006282	regulation of DNA repair	1	EOG84QWH7
GO:0035071	salivary gland cell autophagic cell death	1	EOG8JDKNM
GO:0007283	spermatogenesis	1	EOG81C99V
GO:0046331	lateral inhibition	1	EOG81C99V
GO:0001614	purinergic nucleotide receptor activity	1	EOG84J50X
GO:0044212	transcription regulatory region DNA binding	1	EOG84QWH7
GO:0080019	fatty-acyl-CoA reductase (alcohol-forming) activity	1	EOG8DV82W
GO:0016810	hydrolase activity, acting on carbon-nitrogen (but not peptide) bonds	1	EOG8TXF6D
GO:0006457	protein folding	1	EOG8GTNV8
GO:0030686	90S preribosome	1	EOG8P8HZZ
GO:0060305	regulation of cell diameter	1	EOG81C99V
GO:0035556	intracellular signal transduction	1	EOG8S1WN4
GO:0008219	cell death	1	EOG84QWH7
GO:0016607	nuclear speck	1	EOG8935ZF
GO:0052871	alpha-tocopherol omega-hydroxylase activity	1	EOG83N9TJ
GO:0050051	leukotriene-B4 20-monooxygenase activity	1	EOG83N9TJ
GO:0006887	exocytosis	1	EOG8SJ7TM

GO:0035999	tetrahydrofolate interconversion	1	EOG89626S
GO:0008643	carbohydrate transport	1	EOG80P6ND
GO:0043022	ribosome binding	1	EOG8P8HZZ
GO:0016787	hydrolase activity	1	EOG8W3W3W
GO:0019094	pole plasm mRNA localization	1	EOG8JHFXB
GO:0015771	trehalose transport	1	EOG80P6ND
GO:0016788	hydrolase activity, acting on ester bonds	1	EOG83FK9M
GO:0009267	cellular response to starvation	1	EOG84QWH7
GO:0004842	ubiquitin-protein transferase activity	1	EOG8HB12K
GO:0016079	synaptic vesicle exocytosis	1	EOG8GJ22Z
GO:0008021	synaptic vesicle	1	EOG8GJ22Z
GO:0051123	RNA polymerase II transcriptional preinitiation complex assembly	1	EOG8S4RZ6
GO:0008026	ATP-dependent helicase activity	1	EOG8935ZF
GO:0009411	response to UV	1	EOG84QWH7
GO:0030955	potassium ion binding	1	EOG8RZ1D8
GO:0000123	histone acetyltransferase complex	1	EOG8S4RZ6
GO:0000122	negative regulation of transcription from RNA polymerase II promoter	1	EOG84QWH7
GO:0000287	magnesium ion binding	1	EOG8RZ1D8
GO:0005509	calcium ion binding	1	EOG8SJ7TM
GO:0008859	exoribonuclease II activity	1	EOG8S1WN4
GO:0008283	cell proliferation	1	EOG81C99V
GO:0016324	apical plasma membrane	1	EOG8SJ7TM
GO:0016325	oocyte microtubule cytoskeleton organization	1	EOG8JHFXB
GO:0071013	catalytic step 2 spliceosome	1	EOG8JHFXB
GO:0016323	basolateral plasma membrane	1	EOG8SJ7TM
GO:0046928	regulation of neurotransmitter secretion	1	EOG8SJ7TM
GO:0007405	neuroblast proliferation	1	EOG84QWH7
GO:0006978	DNA damage response, signal transduction by p53 class mediator resulting in transcription of p21 class mediator	1	EOG84QWH7
GO:0030259	lipid glycosylation	1	EOG8BCH22
GO:0006338	chromatin remodeling	1	EOG8935ZF
GO:0007279	pole cell formation	1	EOG81C99V
GO:0004667	prostaglandin-D synthase activity	1	EOG85F05D
GO:0004197	cysteine-type endopeptidase activity	1	EOG8JDKNM
GO:0004743	pyruvate kinase activity	1	EOG8RZ1D8
GO:0008608	attachment of spindle microtubules to kinetochore	1	EOG8PP0Z9

GO:0042593	glucose homeostasis	1	EOG8RZ1DX
GO:0045887	positive regulation of synaptic growth at neuromuscular junction	1	EOG8SJ7TM
GO:0045886	negative regulation of synaptic growth at neuromuscular junction	1	EOG8S1WNX
GO:0050908	detection of light stimulus involved in visual perception	1	EOG8SJ7TM
GO:0007274	neuromuscular synaptic transmission	1	EOG8GJ22Z
GO:0006096	glycolytic process	1	EOG8RZ1D8
GO:0008354	germ cell migration	1	EOG8W9MJT
GO:0007474	imaginal disc-derived wing vein specification	1	EOG88D38H
GO:0051301	cell division	1	EOG8PP0Z9
GO:0000278	mitotic cell cycle	1	EOG81C99V
GO:0019216	regulation of lipid metabolic process	1	EOG8RZ1DX
GO:0007314	oocyte anterior/posterior axis specification	1	EOG8JHFXB
GO:0000447	endonucleolytic cleavage in ITS1 to separate SSU-rRNA from 5.8S rRNA and LSU-rRNA from tricistronic rRNA transcript (SSU-rRNA, 5.8S rRNA, LSU-rRNA)	1	EOG8P8HZZ
GO:0007602	phototransduction	1	EOG8SJ7TM
GO:0044163	host cytoskeleton	1	EOG8Z91BB
GO:0000932	cytoplasmic mRNA processing body	1	EOG8S1WNX
GO:0022857	transmembrane transporter activity	1	EOG80P6ND
GO:0005762	mitochondrial large ribosomal subunit	1	EOG8JMB63
GO:0006414	translational elongation	1	EOG8ZCWNB
GO:0005681	spliceosomal complex	1	EOG8935ZF
GO:0047451	3-hydroxyoctanoyl-[acyl-carrier-protein] dehydratase activity	1	EOG83FK9M
GO:0004497	monooxygenase activity	1	EOG83N9TJ
GO:0070330	aromatase activity	1	EOG83N9TJ
GO:0007618	mating	1	EOG83N9TJ
GO:0043523	regulation of neuron apoptotic process	1	EOG84QWH7
GO:0016592	mediator complex	1	EOG8WSZRB
GO:0005549	odorant binding	1	EOG8RZ1DX
GO:0004364	glutathione transferase activity	1	EOG85F05D
GO:0045787	positive regulation of cell cycle	1	EOG84QWH7
GO:0047886	farnesol dehydrogenase activity	1	EOG8F4VSG
GO:0016746	transferase activity, transferring acyl groups	1	EOG8DV82W
GO:0003730	mRNA 3'-UTR binding	1	EOG8JHFXB
GO:0005319	lipid transporter activity	1	EOG80306V
GO:0006936	muscle contraction	1	EOG8SJ7TM

GO:0047134	protein-disulfide reductase activity	1	EOG854CDX
GO:0008076	voltage-gated potassium channel complex	1	EOG88SK71
GO:0004386	helicase activity	1	EOG8935ZF
GO:0016891	endoribonuclease activity, producing 5'-phosphomonoesters	1	EOG88973C
GO:0000155	phosphorelay sensor kinase activity	1	EOG88SK71
GO:0005829	cytosol	1	EOG84QWH7
GO:0004312	fatty acid synthase activity	1	EOG83FK9M
GO:0004313	[acyl-carrier-protein] S-acetyltransferase activity	1	EOG83FK9M
GO:0016296	palmitoyl-[acyl-carrier-protein] hydrolase activity	1	EOG83FK9M
GO:0046533	negative regulation of photoreceptor cell differentiation	1	EOG84QWH7
GO:0004317	3-hydroxypalmitoyl-[acyl-carrier-protein] dehydratase activity	1	EOG83FK9M
GO:0004314	[acyl-carrier-protein] S-malonyltransferase activity	1	EOG83FK9M
GO:0004315	3-oxoacyl-[acyl-carrier-protein] synthase activity	1	EOG83FK9M
GO:0004319	enoyl-[acyl-carrier-protein] reductase (NADPH, B-specific) activity	1	EOG83FK9M
GO:0046718	viral entry into host cell	1	EOG8Z91BB
GO:0004032	alditol:NADP+ 1-oxidoreductase activity	1	EOG8D2950
GO:0008251	tRNA-specific adenosine deaminase activity	1	EOG8D2950
GO:0003837	beta-ureidopropionase activity	1	EOG8TXF6D
GO:0008315	meiotic G2/MI transition	1	EOG81C99V
GO:0006807	nitrogen compound metabolic process	1	EOG8TXF6D
GO:0046080	dUTP metabolic process	1	EOG8W3W3W
GO:0051078	meiotic nuclear envelope disassembly	1	EOG81C99V
GO:0006208	pyrimidine nucleobase catabolic process	1	EOG8TXF6D
GO:0006207	'de novo' pyrimidine nucleobase biosynthetic process	1	EOG8TXF6D
GO:0006919	activation of cysteine-type endopeptidase activity involved in apoptotic process	1	EOG84QWH7
GO:0006200	obsolete ATP catabolic process	1	EOG8935ZF
GO:0006367	transcription initiation from RNA polymerase II promoter	1	EOG8S4RZ6
GO:0004144	diacylglycerol O-acyltransferase activity	1	EOG8F4VRC
GO:0020037	heme binding	1	EOG83N9TJ
GO:0030141	secretory granule	1	EOG8Q87B6
GO:0050790	regulation of catalytic activity	1	EOG8JDKNM
GO:0010212	response to ionizing radiation	1	EOG84QWH7
GO:0051233	spindle midzone	1	EOG8PP0Z9

GO:0009953	dorsal/ventral pattern formation	1	EOG8JHFXB
GO:0045433	male courtship behavior, veined wing generated song production	1	EOG8SJ7TM
GO:0052869	arachidonic acid omega-hydroxylase activity	1	EOG83N9TJ
GO:0003746	translation elongation factor activity	1	EOG8S1WN4
GO:0045495	pole plasm	1	EOG8PP0Z9
GO:0003743	translation initiation factor activity	1	EOG8S4RZ6
GO:0006633	fatty acid biosynthetic process	1	EOG8RZ1DX
GO:0016705	oxidoreductase activity, acting on paired donors, with incorporation or reduction of molecular oxygen	1	EOG83N9TJ
GO:0071480	cellular response to gamma radiation	1	EOG84QWH7
GO:0009314	response to radiation	1	EOG84QWH7
GO:0019953	sexual reproduction	1	EOG8Q87B6
GO:0035335	peptidyl-tyrosine dephosphorylation	1	EOG81C99V
GO:0035330	regulation of hippo signaling	1	EOG8CNT5M
GO:0007049	cell cycle	1	EOG8PP0Z9
GO:0016063	rhodopsin biosynthetic process	1	EOG8F4VSG
GO:0005581	collagen trimer	1	EOG83N9TJ
GO:0071805	potassium ion transmembrane transport	1	EOG88SK71
GO:0002027	regulation of heart rate	1	EOG8SJ7TM
GO:0006749	glutathione metabolic process	1	EOG85F05D
GO:0008033	tRNA processing	1	EOG8D2950
GO:0033962	cytoplasmic mRNA processing body assembly	1	EOG8S1WNX
GO:0008138	protein tyrosine/serine/threonine phosphatase activity	1	EOG81C99V
GO:0005215	transporter activity	1	EOG847J8K
GO:0008134	transcription factor binding	1	EOG84QWH7
GO:0005869	dynactin complex	1	EOG88GZHQ
GO:0007131	reciprocal meiotic recombination	1	EOG84QWH7
GO:2000685	positive regulation of cellular response to X-ray	1	EOG84QWH7
GO:0007030	Golgi organization	1	EOG81C99V
GO:0031625	ubiquitin protein ligase binding	1	EOG84QWH7
GO:0004984	olfactory receptor activity	1	EOG8RZ1DX
GO:0004594	pantothenate kinase activity	1	EOG8C8B8R
GO:0016239	positive regulation of macroautophagy	1	EOG84QWH7
GO:0004170	dUTP diphosphatase activity	1	EOG8W3W3W
GO:0030246	carbohydrate binding	1	EOG8BCH22
GO:0048047	mating behavior, sex discrimination	1	EOG8RZ1DX
GO:0042045	epithelial fluid transport	1	EOG8SJ7TM

GO:0005819	spindle	1	EOG8PP0Z9
GO:0003887	DNA-directed DNA polymerase activity	1	EOG8XSP61
GO:0004674	protein serine/threonine kinase activity	1	EOG8KSRZK
GO:0045931	positive regulation of mitotic cell cycle	1	EOG81C99V
GO:0045930	negative regulation of mitotic cell cycle	1	EOG81C99V
GO:0046620	regulation of organ growth	1	EOG84QWH7
GO:0004000	adenosine deaminase activity	1	EOG8D2950
GO:0004004	ATP-dependent RNA helicase activity	1	EOG8935ZF
GO:0030430	host cell cytoplasm	1	EOG8Z91BB
GO:0007268	synaptic transmission	1	EOG8SJ7TM
GO:0008344	adult locomotory behavior	1	EOG8SJ7TM
GO:0009744	response to sucrose	1	EOG8RZ1DX
GO:0006555	methionine metabolic process	1	EOG89626S
GO:0008299	isoprenoid biosynthetic process	1	EOG8W9MJT
GO:0008298	intracellular mRNA localization	1	EOG8JHFXB
GO:0006396	RNA processing	1	EOG8D2950
GO:0005783	endoplasmic reticulum	1	EOG8RZ1DX
GO:0006470	protein dephosphorylation	1	EOG81C99V
GO:0005789	endoplasmic reticulum membrane	1	EOG83N9TJ
GO:0005778	peroxisomal membrane	1	EOG8DV82W
GO:0035186	syncytial blastoderm mitotic cell cycle	1	EOG8PP0Z9
GO:0016999	antibiotic metabolic process	1	EOG8BCH22
GO:0005777	peroxisome	1	EOG8DV82W
GO:0055085	transmembrane transport	1	EOG8R2686
GO:0019058	viral life cycle	1	EOG89360G
GO:0032440	2-alkenal reductase [NAD(P)] activity	1	EOG80P6ND
GO:0019428	allantoin biosynthetic process	1	EOG8PP0ZK
GO:0042127	regulation of cell proliferation	1	EOG84QWH7
GO:0006605	protein targeting	1	EOG8JHFXB
GO:0007079	mitotic chromosome movement towards spindle pole	1	EOG8PP0Z9
GO:0055114	oxidation-reduction process	1	EOG85F05D
GO:0045792	negative regulation of cell size	1	EOG81C99V
GO:0070509	calcium ion import	1	EOG8SJ7TM
GO:0016758	transferase activity, transferring hexosyl groups	1	EOG8BCH22
GO:0045494	photoreceptor cell maintenance	1	EOG88D38H
GO:0007297	ovarian follicle cell migration	1	EOG8JHFXB
GO:0042391	regulation of membrane potential	1	EOG88SK71
GO:0000028	ribosomal small subunit assembly	1	EOG8P8HZZ

GO:0048167	regulation of synaptic plasticity	1	EOG8GJ22Z
GO:0010025	wax biosynthetic process	1	EOG8DV82W
GO:0000022	mitotic spindle elongation	1	EOG8P8HZZ
GO:0042981	regulation of apoptotic process	1	EOG89360G
GO:0014016	neuroblast differentiation	1	EOG84QWH7
GO:0031571	mitotic G1 DNA damage checkpoint	1	EOG84QWH7
GO:0003724	RNA helicase activity	1	EOG8935ZF
GO:0006810	transport	1	EOG8JMB63
GO:0004043	L-aminoadipate-semialdehyde dehydrogenase activity	1	EOG8DV82W
GO:0006813	potassium ion transport	1	EOG88SK71
GO:0016301	kinase activity	1	EOG8RZ1D8
GO:0016307	phosphatidylinositol phosphate kinase activity	1	EOG8NZX7W
GO:0006816	calcium ion transport	1	EOG8SJ7TM
GO:0003824	catalytic activity	1	EOG8S7N55
GO:0042302	structural constituent of cuticle	1	EOG8QJV4S
GO:0007424	open tracheal system development	1	EOG81C99V
GO:0007422	peripheral nervous system development	1	EOG81C99V
GO:0006355	regulation of transcription, DNA-templated	1	EOG8S4RZ6
GO:0006352	DNA-templated transcription, initiation	1	EOG8S4RZ6
GO:0004602	glutathione peroxidase activity	1	EOG85F05D
GO:0007369	gastrulation	1	EOG81C99V
GO:0031012	extracellular matrix	1	EOG8QJV4S
GO:0008234	cysteine-type peptidase activity	1	EOG8JDKNM
GO:0008237	metallopeptidase activity	1	EOG8CZF0X
GO:0001700	embryonic development via the syncytial blastoderm	1	EOG8PP0Z9
GO:0015574	trehalose transmembrane transporter activity	1	EOG80P6ND
GO:0019013	viral nucleocapsid	1	EOG8JHFXB
GO:0045977	positive regulation of mitotic cell cycle, embryonic	1	EOG81C99V
GO:0015450	P-P-bond-hydrolysis-driven protein transmembrane transporter activity	1	EOG8JHFXB
GO:0032504	multicellular organism reproduction	1	EOG8QJV4S
GO:0006723	cuticle hydrocarbon biosynthetic process	1	EOG8RZ1DX
GO:0030798	trans-aconitate 2-methyltransferase activity	1	EOG8ZKN28
GO:0004519	endonuclease activity	1	EOG8DBWWP
GO:0016717	oxidoreductase activity, acting on paired donors, with oxidation of a pair of donors resulting in the reduction of molecular oxygen to two molecules of water	1	EOG8RZ1DX
GO:0002039	p53 binding	1	EOG84QWH7

GO:0005201	extracellular matrix structural constituent	1	EOG83N9TJ
GO:0005891	voltage-gated calcium channel complex	1	EOG8SJ7TM
GO:0007140	male meiosis	1	EOG81C99V
GO:0045017	glycerolipid biosynthetic process	1	EOG8F4VRC
GO:0010332	response to gamma radiation	1	EOG84QWH7
GO:0030720	oocyte localization involved in germarium-derived egg chamber formation	1	EOG8JHFXB
GO:0015020	glucuronosyltransferase activity	1	EOG8BCH22
GO:0010506	regulation of autophagy	1	EOG8RZ1DX
GO:0004161	dimethylallyltranstransferase activity	1	EOG8W9MJT
GO:0046427	positive regulation of JAK-STAT cascade	1	EOG80P6ND
GO:0030330	DNA damage response, signal transduction by p53 class mediator	1	EOG84QWH7
GO:0007586	digestion	1	EOG80306V
GO:0006915	apoptotic process	1	EOG84QWH7
GO:0009058	biosynthetic process	1	EOG83FK9M
GO:0042025	host cell nucleus	1	EOG8Z91BB
GO:0000775	chromosome, centromeric region	1	EOG8PP0Z9
GO:0006310	DNA recombination	1	EOG8RZ1DV
GO:0000079	regulation of cyclin-dependent protein serine/threonine kinase activity	1	EOG8PP0Z9
GO:0007099	centriole replication	1	EOG81C99V
GO:0001047	core promoter binding	1	EOG84QWH7
GO:0004768	stearoyl-CoA 9-desaturase activity	1	EOG8RZ1DX
GO:0035062	omega speckle	1	EOG8JHFXB
GO:0004180	carboxypeptidase activity	1	EOG80306V
GO:1990248	regulation of transcription from RNA polymerase II promoter in response to DNA damage	1	EOG84QWH7
GO:0007293	germarium-derived egg chamber formation	1	EOG8JHFXB
GO:0003851	2-hydroxyacylsphingosine 1-beta-galactosyltransferase activity	1	EOG8BCH22
GO:0016539	intein-mediated protein splicing	1	EOG83N9TJ
GO:0031982	vesicle	1	EOG80306V
GO:0016538	cyclin-dependent protein serine/threonine kinase regulator activity	1	EOG8PP0Z9
GO:0000461	endonucleolytic cleavage to generate mature 3'-end of SSU-rRNA from (SSU-rRNA, 5.8S rRNA, LSU-rRNA)	1	EOG8P8HZZ
GO:0035561	regulation of chromatin binding	1	EOG8PP0Z9
GO:0042246	tissue regeneration	1	EOG84QWH7
GO:0005703	polytene chromosome puff	1	EOG8JHFXB

GO:0018685	alkane 1-monooxygenase activity	1	EOG83N9TJ
GO:0050062	long-chain-fatty-acyl-CoA reductase activity	1	EOG8DV82W
GO:0005669	transcription factor TFIID complex	1	EOG8S4RZ6
GO:0060179	male mating behavior	1	EOG8RZ1DX
GO:0007632	visual behavior	1	EOG8SJ7TM
GO:0004320	oleoyl-[acyl-carrier-protein] hydrolase activity	1	EOG83FK9M
GO:0007067	mitotic nuclear division	1	EOG81C99V
GO:0004930	G-protein coupled receptor activity	1	EOG80306V
GO:0016295	myristoyl-[acyl-carrier-protein] hydrolase activity	1	EOG83FK9M
GO:0007348	regulation of syncytial blastoderm mitotic cell cycle	1	EOG81C99V
GO:0007349	cellularization	1	EOG81C99V
GO:0050111	mycocerosate synthase activity	1	EOG83FK9M
GO:0075521	microtubule-dependent intracellular transport of viral material towards nucleus	1	EOG8Z91BB
GO:0005337	nucleoside transmembrane transporter activity	1	EOG8N3335
GO:0005667	transcription factor complex	1	EOG84QWH7
GO:0000124	SAGA complex	1	EOG8S4RZ6
GO:0008010	structural constituent of chitin-based larval cuticle	1	EOG8QJV4S
GO:0008152	metabolic process	1	EOG85F05D
GO:0051445	regulation of meiotic cell cycle	1	EOG81C99V
GO:0004687	myosin light chain kinase activity	1	EOG8KSRZK
GO:0004725	protein tyrosine phosphatase activity	1	EOG81C99V
GO:0004337	geranyltranstransferase activity	1	EOG8W9MJT
GO:0005249	voltage-gated potassium channel activity	1	EOG88SK71
GO:0005245	voltage-gated calcium channel activity	1	EOG8SJ7TM
GO:0033119	negative regulation of RNA splicing	1	EOG8JHFXB
GO:0015937	coenzyme A biosynthetic process	1	EOG8C8B8R
GO:0008331	high voltage-gated calcium channel activity	1	EOG8SJ7TM
GO:0008332	low voltage-gated calcium channel activity	1	EOG8SJ7TM
GO:0035220	wing disc development	1	EOG8W9MJT
GO:0034644	cellular response to UV	1	EOG84QWH7
GO:0010165	response to X-ray	1	EOG84QWH7
GO:0019076	viral release from host cell	1	EOG8Z91BB
GO:0001104	RNA polymerase II transcription cofactor activity	1	EOG8WSZRB
GO:0045893	positive regulation of transcription, DNA-templated	1	EOG84QWH7
GO:0086010	membrane depolarization during action potential	1	EOG8SJ7TM
GO:0003960	NADPH:quinone reductase activity	1	EOG83FK9M

GO:0003690	double-stranded DNA binding	1	EOG84QWH7
GO:0045451	pole plasm oskar mRNA localization	1	EOG8JHFXB
GO:0070073	clustering of voltage-gated calcium channels	1	EOG8GJ22Z
GO:0008630	intrinsic apoptotic signaling pathway in response to DNA damage	1	EOG84QWH7
GO:0042811	pheromone biosynthetic process	1	EOG8RZ1DX
GO:0042810	pheromone metabolic process	1	EOG8RZ1DX
GO:0017148	negative regulation of translation	1	EOG8JHFXB
GO:0002121	inter-male aggressive behavior	1	EOG8BCH22
GO:0003682	chromatin binding	1	EOG84QWH7
GO:0003684	damaged DNA binding	1	EOG84QWH7
GO:0003729	mRNA binding	1	EOG8JHFXB
GO:0008284	positive regulation of cell proliferation	1	EOG81C99V
GO:0000184	nuclear-transcribed mRNA catabolic process, nonsense-mediated decay	1	EOG8JHFXB
GO:0004871	signal transducer activity	1	EOG88SK71
GO:0040003	chitin-based cuticle development	1	EOG8QJV4S
GO:0008521	acetyl-CoA transporter activity	1	EOG88GZHQ
GO:0030529	ribonucleoprotein complex	1	EOG8JHFXB
GO:0035234	ectopic germ cell programmed cell death	1	EOG84QWH7
GO:0006400	tRNA modification	1	EOG8D2950
GO:0005694	chromosome	1	EOG81C99V
GO:0006405	RNA export from nucleus	1	EOG8JHFXB
GO:0006407	rRNA export from nucleus	1	EOG8P8HZZ
GO:0070328	triglyceride homeostasis	1	EOG8RZ1DX
GO:0007346	regulation of mitotic cell cycle	1	EOG81C99V
GO:0004489	methylenetetrahydrofolate reductase (NAD(P)H) activity	1	EOG89626S
GO:0005882	intermediate filament	1	EOG8GTNV8
GO:0005886	plasma membrane	1	EOG80P6ND
GO:0042771	intrinsic apoptotic signaling pathway in response to DNA damage by p53 class mediator	1	EOG84QWH7

Table 4S. Transcription factors with predicted motifs.

Gene_ID	DBDs[1]	Species_mapped_from[2]	Gene_mapped_from[3]	Num_MMs[4]	DBD %ID[5]	Source_Type[6]	IUPAC_fwd[7]	IUPAC_rev[8]	Author	Year	PMID
LDEC000035	Homeobox	*Mus musculus*	ENSMUSG00000029646	7	0.879	PBM	NWWAYDRNN	NNYHRTWWN	Berger	2008	18585359
LDEC000063	HLH	*Drosophila melanogaster*	FBgn0001994	19	0.635	B1H	VAHCAGCTGAY	RTCAGCTGDWB	Zhu	2011	21097781
LDEC000070	Homeobox	*Homo sapiens*	ENSG00000100987	11	0.776	SELEX	NNYAATTRRBN	NVYYAATTRNN	Jolma	2013	23332764
LDEC000161	CBFB_NFYA	*Homo sapiens*	ENSG00000001167	4	0.930	Transfac	NYTGATTGGYTAVH	DBTARCCAATCARN	Matys	2006	16381825
LDEC000201	Homeobox	*Drosophila melanogaster*	FBgn0014343	0	1.000	B1H	TGTWW	WWACA	Mathelier	2014	24194598
LDEC000284	zf-C2H2,zf-C2H2	*Drosophila melanogaster*	FBgn0259750	0	1.000	B1H	NNNBNNNNDNRGKWCCTGGYBDNNBN	NVNNHVRCCAGGWMCYNHNNNNVNNN	Zhu	2011	21097781
LDEC000516	GATA	*Drosophila melanogaster*	FBgn0038391	8	0.771	B1H	WGATAAG	CTTATCW	Zhu	2011	21097781
LDEC000649	Homeobox	*Drosophila melanogaster*	FBgn0052532	13	0.772	B1H	YTAATTR	YAATTAR	Mathelier	2014	24194598
LDEC000822	Ets	*Drosophila melanogaster*	FBgn0039225	7	0.918	PBM	NDCCGGAWRY	RYWTCCGGHN	Wei	2010	20517297
LDEC000894	Homeobox	*Drosophila melanogaster*	FBgn0025776	11	0.807	B1H	YTAATKA	TMATTAR	Mathelier	2014	24194598
LDEC000905	Homeobox	*Drosophila melanogaster*	FBgn0002522	4	0.930	B1H	TTAATKA	TMATTAA	Mathelier	2014	24194598
LDEC000910	Homeobox	*Drosophila melanogaster*	FBgn0051481	0	1.000	B1H	YTAATKA	TMATTAR	Mathelier	2014	24194598
LDEC000916	Homeobox	*Homo sapiens*	ENSG00000120093	12	0.789	SELEX	NBTAATKRNN	NNYMATTAVN	Jolma	2013	23332764
LDEC000946	HLH	*Drosophila melanogaster*	FBgn0011277	2	0.962	Transfac	NDRGGVMGCAGCTGMKNCCHHN	NDDGGNMKCAGCTGCKBCCYHN	Matys	2006	16381825
LDEC001043	HLH	*Homo sapiens*	ENSG00000135547	6	0.893	PBM	RCACGTGNN	NNCACGTGY	Weirauch	2014	25215497
LDEC001157	zf-C2H2,zf-C2H2,zf-C2H2,zf-C2H2,zf-C2H2	*Drosophila melanogaster*	FBgn0001981	15	0.872	B1H	CACCTGH	DCAGGTG	Zhu	2011	21097781
LDEC001249	Homeobox	*Drosophila melanogaster*	FBgn0000157	14	0.754	PBM	NNTAATTABN	NVTAATTANN	Weirauch	2014	25215497
LDEC001262	Homeobox	*Drosophila melanogaster*	FBgn0000606	8	0.860	PBM	NTAATNDNN	NNHNATTAN	Weirauch	2014	25215497
LDEC001304	Homeobox	*Drosophila melanogaster*	FBgn0001235	8	0.862	B1H	TGACAK	MTGTCA	Mathelier	2014	24194598
LDEC001414	HLH	*Drosophila melanogaster*	FBgn0263118	18	0.700	B1H	AMCAYMTGKH	DMCAKRTGKT	Zhu	2011	21097781
LDEC001528	Fork_head	*Neurospora crassa*	NCU06173	51	0.474	PBM	DTGTTTAY	RTAAACAH	Weirauch	2014	25215497
LDEC001639	Homeobox	*Drosophila melanogaster*	FBgn0004854	10	0.825	B1H	CAATTAW	WTAATTG	Mathelier	2014	24194598

LDEC001973	zf-C2H2,zf-C2H2,zf-C2H2	*Drosophila melanogaster*	FBgn0261705	12	0.836	B1H	VRCCACRCCCH	DGGGYGTGGYB	Zhu	2011	21097781
LDEC002142	CSD	*Homo sapiens*	ENSG00000065978	12	0.831	Transfac	HNATTGGHNNN	NNNDCCAATND	Matys	2006	16381825
LDEC002322	Homeobox	*Homo sapiens*	ENSG00000016082	0	1.000	Transfac	HYATTANBH	DVNTAATRD	Matys	2006	16381825
LDEC003050	zf-C4	*Anopheles gambiae*	AGAP010438	1	0.986	PBM	NRGDKCRNN	NNYGMHCYN	Weirauch	2014	25215497
LDEC003257	Homeobox	*Drosophila melanogaster*	FBgn0003460	1	0.982	PBM	NDATMYNNN	NNNRKATHN	Weirauch	2014	25215497
LDEC003555	zf-C4	*Drosophila melanogaster*	FBgn0015381	5	0.932	B1H	AAAAGTCANH	DNTGACTTTT	Zhu	2011	21097781
LDEC003580	Homeobox	*Drosophila melanogaster*	FBgn0054031	7	0.877	PBM	NNNDNNRN	NYNNHNNN	Weirauch	2014	25215497
LDEC003645	HLH	*Drosophila melanogaster*	FBgn0000137	16	0.754	B1H	RCAGGTG	CACCTGY	Zhu	2011	21097781
LDEC003759	zf-C2H2,zf-C2H2,zf-C2H2	*Drosophila melanogaster*	FBgn0011236	9	0.873	B1H	NNRCTTTCWCYNNNN	NNNNNRGWGAAAGYNN	Zhu	2011	21097781
LDEC003820	HMG_box	*Caenorhabditis elegans*	T22B7.1	5	0.928	PBM	NWATTGTTNN	NNAACAATWN	Narasimhan	2015	25905672
LDEC003904	MH1	*Drosophila melanogaster*	FBgn0011648	6	0.943	Transfac	NBYGYCKM	KMGRCRVN	Matys	2006	16381825
LDEC004019	HLH	*Drosophila melanogaster*	FBgn0023094	9	0.833	B1H	KCACGTGAC	GTCACGTGM	Zhu	2011	21097781
LDEC004155	Fork_head	*Drosophila melanogaster*	FBgn0015396	14	0.859	Transfac	DNNVBKNATRGCGTCYDNRNHB	VDNYNHRGACGCYATNMVBNNH	Matys	2006	16381825
LDEC004212	SRF-TF	*Drosophila melanogaster*	FBgn0011656	0	1.000	ChIP-seq	YTAWWWWTAR	YTAWWWWTAR	Boyle	2014	25164757
LDEC004228	bZIP_1	*Drosophila melanogaster*	FBgn0262975	23	0.635	ChIP-seq	TGAYTBDGCA	TGCHVARTCA	Boyle	2014	25164757
LDEC004327	Homeobox	*Drosophila melanogaster*	FBgn0000439	0	1.000	Transfac	NNNNNNNATTAMYNNNN	NNNNRKTAATNNNNNN	Matys	2006	16381825
LDEC004332	Homeobox	*Homo sapiens*	ENSG00000120087	6	0.895	HocoMoco	WTGATTDATK	MATHAATCAW	Kulakovskiy	2013	23175603
LDEC004337	Homeobox	*Drosophila melanogaster*	FBgn0260642	0	1.000	Transfac	MGATTGR	YCAATCK	Matys	2006	16381825
LDEC004436	Homeobox	*Drosophila melanogaster*	FBgn0033748	5	0.914	B1H	TGACAB	BTGTCA	Mathelier	2014	24194598
LDEC004670	HLH	*Drosophila melanogaster*	FBgn0015014	2	0.963	B1H	VTACGTGACB	VGTCACGTAB	Zhu	2011	21097781
LDEC004697	HLH	*Drosophila melanogaster*	FBgn0263112	7	0.883	B1H	CACGTGA	TCACGTG	Zhu	2011	21097781
LDEC004703	Myb_DNA-binding	*Homo sapiens*	ENSG00000139613	3	0.935	ChIP-seq	RRACTACAWBTCCCAKVAKGC	GCMTBMTGGGAVWTGTAGTYY	Gerstein	2012	22955619
LDEC004787	TF_AP-2	*Mus musculus*	ENSMUSG00000042477	47	0.774	PBM	CCBVRGGCNN	NNGCCYBVGG	Badis	2009	19443739
LDEC004800	Homeobox	*Homo sapiens*	ENSG00000136352	17	0.707	Transfac	NNYNCTTGDVNN	NNBHCAAGNRNN	Matys	2006	16381825
LDEC004833	bZIP_1	*Mus musculus*	ENSMUSG00000056216	22	0.651	PBM	NVTTDCNNNN	NNNNGHAABN	Weirauch	2014	25215497

LDEC004906	bZIP_1	*Drosophila melanogaster*	FBgn0001291	18	0.719	B1H	SRTGASTCAKH	DMTGASTCAYS	Zhu	2011	21097781
LDEC005027	HLH	*Mus musculus*	ENSMUSG00000045680	4	0.925	PBM	MCAKMTGKY	RMCAKMTGK	Weirauch	2014	25215497
LDEC005040	HLH	*Drosophila melanogaster*	FBgn0262656	20	0.623	B1H	HCCACGTGBY	RVCACGTGGD	Zhu	2011	21097781
LDEC005305	Homeobox	*Drosophila melanogaster*	FBgn0000014	0	1.000	Transfac	HHHHATTDVH	DBHAATDDDD	Matys	2006	16381825
LDEC005307	Homeobox	*Drosophila melanogaster*	FBgn0000015	2	0.965	Transfac	TTTTATK	MATAAAA	Matys	2006	16381825
LDEC005682	zf-C4	*Drosophila melanogaster*	FBgn0003720	20	0.722	PBM	NRASGTCAANNN	NNNTTGACSTYN	Weirauch	2014	25215497
LDEC005794	zf-C4	*Caenorhabditis elegans*	F11A1.3	20	0.714	ChIP-seq	VMHRCAMRCRCRCWC	GWGYGYGYKTGYDKB	Mathelier	2014	24194598
LDEC005898	zf-C2H2,zf-C2H2,zf-C2H2,zf-C2H2,zf-C2H2,zf-C2H2,zf-C2H2,zf-C2H2	*Drosophila melanogaster*	FBgn0027339	21	0.900	B1H	TSKTTTTTTTT	AAAAAAAAMSA	Zhu	2011	21097781
LDEC006101	HLH	*Homo sapiens*	ENSG00000158773	20	0.672	PBM	NNCACGTGRYN	NRYCACGTGNN	Weirauch	2014	25215497
LDEC006285	RHD	*Gallus gallus*	ENSGALG00000000671	66	0.590	PBM	NRTGGAAAWN	NWTTTCCAYN	Weirauch	2014	25215497
LDEC006476	Homeobox	*Homo sapiens*	ENSG00000109132	16	0.719	SELEX	TAAYBYAATTA	TAATTRVRTTA	Jolma	2013	23332764
LDEC006514	HLH	*Drosophila melanogaster*	FBgn0032741	18	0.723	B1H	DAAMCABCTGY	RCAGVTGKTTH	Zhu	2011	21097781
LDEC006528	HLH	*Homo sapiens*	ENSG00000122691	3	0.942	PBM	NACATATGKN	NMCATATGTN	Weirauch	2014	25215497
LDEC006538	zf-C2H2,zf-C2H2,zf-C2H2,zf-C2H2	*Drosophila melanogaster*	FBgn0013469	5	0.946	B1H	NNNNMCACCCMCRCN	NGYGKGGGTGKNNNN	Zhu	2011	21097781
LDEC006593	Homeobox	*Drosophila melanogaster*	FBgn0000611	13	0.783	B1H	NTTTGAYR	YRTCAAAN	Mathelier	2014	24194598
LDEC006635	HLH	*Drosophila melanogaster*	FBgn0002735	12	0.793	B1H	CTTGACW	WGTCAAG	Zhu	2011	21097781
LDEC006636	HLH	*Drosophila melanogaster*	FBgn0002735	12	0.793	B1H	CTTGACW	WGTCAAG	Zhu	2011	21097781
LDEC006645	Runt	*Drosophila melanogaster*	FBgn0003300	31	0.772	B1H	YTGCGGTTW	WAACCGCAR	Zhu	2011	21097781
LDEC007243	zf-C2H2,zf-C2H2,zf-C2H2	*Drosophila melanogaster*	FBgn0039169	6	0.918	B1H	BDDGCCMCGCCCMYN	NRKGGGCGKGGCHHV	Zhu	2011	21097781
LDEC007489	bZIP_1	*Homo sapiens*	ENSG00000118260	13	0.790	PBM	NNDCGTMAB	VTKACGHNN	Weirauch	2014	25215497
LDEC007631	Homeobox	*Drosophila melanogaster*	FBgn0000492	2	0.965	B1H	SCAATTA	TAATTGS	Mathelier	2014	24194598

LDEC007795	Homeobox	*Mus musculus*	ENSMUSG00000038805	1	0.982	PBM	NNNAYMN	NKRTNNN	Berger	2008	18585359
LDEC008003	Homeobox	*Homo sapiens*	ENSG00000165495	5	0.915	SELEX	TGACAGSTGTCA	TGACASCTGTCA	Jolma	2013	23332764
LDEC008024	Homeobox	*Mus musculus*	ENSMUSG00000028736	16	0.719	PBM	NNVATTWN	NWAATBNN	Berger	2008	18585359
LDEC008343	zf-C2H2,zf-C2H2,zf-C2H2	*Drosophila melanogaster*	FBgn0020378	0	1.000	B1H	NKHHMCGCCCMYNN	NNRKGGGCGKDDMN	Zhu	2011	21097781
LDEC008345	zf-C2H2,zf-C2H2,zf-C2H2	*Drosophila melanogaster*	FBgn0020378	9	0.877	SELEX	VMCACBCCCMCH	DGKGGGVGTGKB	Jolma	2013	23332764
LDEC008474	RHD	*Drosophila melanogaster*	FBgn0260632	37	0.784	Transfac	HSRGAAAAHYV	BRDTTTTCYSD	Matys	2006	16381825
LDEC008485	Pou,Homeobox	*Apis mellifera*	amel4_ncbi_hmm42917	4	0.970	PBM	NNWATBHNNN	NNNDVATWNN	Weirauch	2014	25215497
LDEC008541	T-box	*Homo sapiens*	ENSG00000184058	36	0.811	Transfac	AGGTGTKAAAG	CTTTMACACCT	Matys	2006	16381825
LDEC008640	Homeobox	*Drosophila melanogaster*	FBgn0026411	13	0.772	B1H	TTAATTA	TAATTAA	Mathelier	2014	24194598
LDEC008700	HMG_box	*Homo sapiens*	ENSG00000181449	1	0.986	Transfac	DDHNYYWTTGTNWTKN	NMAWNACAAWRRNDHH	Matys	2006	16381825
LDEC008709	Homeobox	*Drosophila melanogaster*	FBgn0033748	15	0.746	B1H	TGACAB	BTGTCA	Mathelier	2014	24194598
LDEC008856	TBP,TBP	*Homo sapiens*	ENSG00000112592	14	0.919	Transfac	VDATAAA	TTTATHB	Matys	2006	16381825
LDEC008941	Homeobox	*Drosophila melanogaster*	FBgn0002023	0	1.000	PBM	NYVATNNNNN	NNNNNATBRN	Weirauch	2014	25215497
LDEC008984	PAX	*Homo sapiens*	ENSG00000196092	34	0.728	PBM	NNYGVNYNNN	NNNRNBCRNN	Weirauch	2014	25215497
LDEC009466	zf-C4	*Drosophila melanogaster*	FBgn0000546	7	0.900	B1H	RAGGTCAW	WTGACCTY	Zhu	2011	21097781
LDEC009525	Homeobox	*Drosophila melanogaster*	FBgn0030408	6	0.895	B1H	TTAATDG	CHATTAA	Mathelier	2014	24194598
LDEC009532	Ets	*Drosophila melanogaster*	FBgn0000567	2	0.977	Transfac	NNAAYCMGGAARTNNDV	BHNNAYTTCCKGRWTNN	Matys	2006	16381825
LDEC009536	zf-C2H2,zf-C2H2,zf-C2H2	*Drosophila melanogaster*	FBgn0003028	6	0.914	Transfac	HWCHGTTA	TAACDGWD	Matys	2006	16381825
LDEC009603	Homeobox	*Mus musculus*	ENSMUSG00000027168	5	0.912	PBM	TTAATTVG	CBAATTAA	Berger	2008	18585359
LDEC009702	HMG_box	*Homo sapiens*	ENSG00000148737	3	0.957	Transfac	BCTTTGAWS	SWTCAAAGV	Matys	2006	16381825
LDEC009722	Homeobox	*Homo sapiens*	ENSG00000183072	8	0.860	Transfac	VHCACTTSNVD	HBNSAAGTGDB	Matys	2006	16381825
LDEC009723	Homeobox	*Homo sapiens*	ENSG00000109705	5	0.912	Transfac	YACTYV	BRAGTR	Matys	2006	16381825
LDEC009726	Homeobox	*Drosophila melanogaster*	FBgn0011278	3	0.947	B1H	TNRTTA	TAAYNA	Mathelier	2014	24194598
LDEC009844	Fork_head	*Homo sapiens*	ENSG00000164916	11	0.887	PBM	NTRTKKWYNN	NNRWMMAYAN	Weirauch	2014	25215497
LDEC009878	zf-C4	*Drosophila melanogaster*	FBgn0035849	6	0.914	B1H	MAAGGTCA	TGACCTTK	Zhu	2011	21097781

LDEC009949	HLH	*Drosophila melanogaster*	FBgn0002922	8	0.846	B1H	RACASCTGACGC	GCGTCAGSTGTY	Zhu	2011	21097781
LDEC010173	zf-C4	*Drosophila melanogaster*	FBgn0023546	4	0.943	B1H	AAGGTCA	TGACCTT	Zhu	2011	21097781
LDEC010779	Homeobox	*Drosophila melanogaster*	FBgn0030058	12	0.789	B1H	TTAATTA	TAATTAA	Mathelier	2014	24194598
LDEC010970	HLH	*Homo sapiens*	ENSG00000070444	15	0.717	PBM	NCACGTGNN	NNCACGTGN	Weirauch	2014	25215497
LDEC011025	Homeobox	*Drosophila melanogaster*	FBgn0015561	3	0.947	B1H	BTAATTR	YAATTAV	Mathelier	2014	24194598
LDEC011332	Ets	*Drosophila melanogaster*	FBgn0003118	4	0.952	PBM	NNVMGGAWRH	DYWTCCKBNN	Wei	2010	20517297
LDEC011477	HLH	*Drosophila melanogaster*	FBgn0004666	13	0.759	B1H	VWACGTGACY	RGTCACGTWB	Zhu	2011	21097781
LDEC011519	Homeobox	*Homo sapiens*	ENSG00000188620	0	1.000	Transfac	CACGCACTTG	CAAGTGCGTG	Matys	2006	16381825
LDEC011522	Homeobox	*Drosophila melanogaster*	FBgn0002941	2	0.965	B1H	YTAATKR	YMATTAR	Mathelier	2014	24194598
LDEC011571	HLH	*Drosophila melanogaster*	FBgn0003513	2	0.961	B1H	TGCGTGAC	GTCACGCA	Zhu	2011	21097781
LDEC011614	Homeobox	*Homo sapiens*	ENSG00000156150	15	0.737	SELEX	NTAATYNRATTAN	NTAATYNRATTAN	Jolma	2013	23332764
LDEC011791	DM	*Drosophila melanogaster*	FBgn0039683	1	0.979	PBM	WRTWDCNDNN	NNHNGHWAYW	Weirauch	2014	25215497
LDEC011807	RFX_DNA_binding	*Mus musculus*	ENSMUSG00000024206	11	0.843	SELEX	SGTTGCTARGCAACV	BGTTGCYTAGCAACS	Jolma	2013	23332764
LDEC011897	RFX_DNA_binding	*Mus musculus*	ENSMUSG00000037674	31	0.575	PBM	GTYRCYRNSB	VSNYRGYRAC	Weirauch	2013	23354101
LDEC011938	MH1	*Homo sapiens*	ENSG00000162599	19	0.827	Transfac	NGCCARD	HYTGGCN	Matys	2006	16381825
LDEC011944	zf-C2H2,zf-C2H2,zf-C2H2	*Drosophila melanogaster*	FBgn0025679	17	0.767	B1H	NNHMCGCCCCYYNN	NNRRGGGGCGKDNN	Zhu	2011	21097781
LDEC011960	bZIP_1	*Homo sapiens*	ENSG00000165030	25	0.583	PBM	NNNBRTAAYN	NRTTAYVNNN	Weirauch	2014	25215497
LDEC012179	zf-C4	*Drosophila melanogaster*	FBgn0003651	1	0.986	B1H	ARGGTCA	TGACCYK	Zhu	2011	21097781
LDEC012244	HLH	*Drosophila melanogaster*	FBgn0037937	2	0.962	B1H	CABCTGTBMC	GKVACAGVTG	Zhu	2011	21097781
LDEC012258	GATA,GATA	*Drosophila melanogaster*	FBgn0003117	16	0.771	ChIP-seq	ANHNNBHGATAASSDNNB	VNNHSSTTATCDVNNDNT	Boyle	2014	25164757
LDEC012259	GATA,GATA	*Drosophila melanogaster*	FBgn0003117	12	0.829	ChIP-seq	ANHNNBHGATAASSDNNB	VNNHSSTTATCDVNNDNT	Boyle	2014	25164757
LDEC012278	Fork_head	*Mus musculus*	ENSMUSG00000048756	69	0.434	PBM	NTGTTKAY	RTMAACAN	Weirauch	2013	23354101
LDEC012449	HLH	*Homo sapiens*	ENSG00000100644	16	0.692	Transfac	HRKACGTGSVSB	VSBSCACGTMYD	Matys	2006	16381825
LDEC012456	Fork_head	*Drosophila melanogaster*	FBgn0000659	7	0.927	Transfac	WKWWTGTTKACWNW	WNWGTMAACAWWMW	Matys	2006	16381825
LDEC012480	zf-C4	*Homo sapiens*	ENSG00000153234	7	0.900	Transfac	AAGGYYR	YRRCCTT	Matys	2006	16381825
LDEC012509	GCM	*Drosophila melanogaster*	FBgn0014179	39	0.727	Transfac	NDNATGCGGGYNB	VNRCCCGCATNHN	Matys	2006	16381825

LDEC012729	Ets	*Drosophila melanogaster*	FBgn0000097	10	0.885	B1H	RCCGGAARY	RYTTCCGGY	Zhu	2011	21097781
LDEC012767	PAX	*Drosophila melanogaster*	FBgn0005561	5	0.960	B1H	NNGTYACGSHYSRNY	RNYSRDSCGTRACNN	Zhu	2011	21097781
LDEC012804	HLH	*Homo sapiens*	ENSG00000162367	10	0.811	Transfac	DCCABCTGNY	RNCAGVTGGH	Matys	2006	16381825
LDEC013196	HTH_psq	*Drosophila melanogaster*	FBgn0004870	0	1.000	B1H	KAATTAWATAW	WTATWTAATTM	Zhu	2011	21097781
LDEC013204	zf-C2H2,zf-C2H2,zf-C2H2,zf-C2H2,zf-C2H2	*Drosophila melanogaster*	FBgn0035144	15	0.870	B1H	NVCCACCTGYHVVNN	NNBBDRCAGGTGGBN	Zhu	2011	21097781
LDEC013372	Fork_head	*Homo sapiens*	ENSG00000129654	57	0.486	Transfac	HWDTGTTTGTTTA	TAAACAAACAHWD	Matys	2006	16381825
LDEC013488	zf-C2H2,zf-C2H2,zf-C2H2,zf-C2H2,zf-C2H2	*Drosophila melanogaster*	FBgn0030532	8	0.932	B1H	YVVGCCTAYHBM	KVDRTAGGCBBR	Zhu	2011	21097781
LDEC013573	Ets	*Drosophila melanogaster*	FBgn0005658	25	0.702	B1H	CCGGAARY	RYTTCCGG	Zhu	2011	21097781
LDEC013667	Fork_head	*Drosophila melanogaster*	FBgn0004567	9	0.906	PBM	DTGTTTAY	RTAAACAH	Weirauch	2014	25215497
LDEC013670	Fork_head	*Homo sapiens*	ENSG00000176165	8	0.917	PBM	DTGTTTAY	RTAAACAH	Weirauch	2014	25215497
LDEC013736	Ets	*Homo sapiens*	ENSG00000154727	22	0.738	PBM	RSCGGAARY	RYTTCCGSY	Wei	2010	20517297
LDEC013937	zf-C2H2,zf-C2H2,zf-C2H2,zf-C2H2,zf-C2H2	*Drosophila melanogaster*	FBgn0004859	12	0.908	Transfac	GACCACCCAMG	CKTGGGTGGTC	Matys	2006	16381825
LDEC013944	Homeobox	*Drosophila melanogaster*	FBgn0003944	0	1.000	Transfac	NNVNNNNTAATKRNBHNND	HNNDVNYMATTANNNNBNN	Matys	2006	16381825
LDEC014245	HLH	*Drosophila melanogaster*	FBgn0004666	3	0.943	B1H	VWACGTGACY	RGTCACGTWB	Zhu	2011	21097781
LDEC014511	zf-C4	*Drosophila melanogaster*	FBgn0001078	1	0.986	PBM	RRGGTCAH	DTGACCYY	Weirauch	2014	25215497
LDEC014963	zf-C2H2,zf-C2H2,zf-C2H2,zf-C2H2,zf-C2H2,zf-C2H2	*Drosophila melanogaster*	FBgn0031375	12	0.914	B1H	BBRTTGCTCWTTTBN	NVAAAWGAGCAAYVV	Zhu	2011	21097781
LDEC015014	HLH,bZIP_1	*Homo sapiens*	ENSG00000125952	21	0.767	ChIP-seq	RRNCACATGK	MCATGTGNYY	Mathelier	2014	24194598
LDEC015106	Homeobox	*Drosophila melanogaster*	FBgn0027364	7	0.870	B1H	TGABAC	GTVTCA	Mathelier	2014	24194598
LDEC015171	ARID	*Drosophila melanogaster*	FBgn0004795	21	0.772	PBM	NDATHRNN	NNYDATHN	Weirauch	2014	25215497

LDEC015330	Fork_head	*Homo sapiens*	ENSG00000103241	3	0.969	Transfac	VNDTGTTTATDYR	YRHATAAACAHNB	Matys	2006	16381825
LDEC015408	HLH	*Drosophila melanogaster*	FBgn0002735	13	0.776	B1H	CTTGACW	WGTCAAG	Zhu	2011	21097781
LDEC015702	HLH	*Drosophila melanogaster*	FBgn0010433	7	0.868	B1H	MCAYMTGNC	GNCAKRTGK	Zhu	2011	21097781
LDEC015716	zf-C2H2,zf-C2H2,zf-C2H2,zf-C2H2,zf-C2H2,zf-C2H2,zf-C2H2	*Drosophila melanogaster*	FBgn0003053	34	0.817	B1H	AGCATCM	KGATGCT	Zhu	2011	21097781
LDEC015753	Homeobox	*Drosophila melanogaster*	FBgn0015904	0	1.000	B1H	TGTWW	WWACA	Mathelier	2014	24194598
LDEC015949	MH1	*Drosophila melanogaster*	FBgn0011655	2	0.981	B1H	ABDSMMHGVCGM	KCGBCDKKSHVT	Zhu	2011	21097781
LDEC016095	HLH	*Drosophila melanogaster*	FBgn0000413	2	0.963	B1H	VCCAGATGTB	VACATCTGGB	Zhu	2011	21097781
LDEC016261	Homeobox	*Homo sapiens*	ENSG00000170370	3	0.947	SELEX	YMATTAVYTAATKR	YMATTARBTAATKR	Jolma	2013	23332764
LDEC016277	Pou,Homeobox	*Bombyx mori*	SGF3_BOMMO	2	0.985	Transfac	KTRTKCA	TGMAYAM	Matys	2006	16381825
LDEC016362	HLH	*Drosophila melanogaster*	FBgn0037475	5	0.906	HocoMoco	HDGGRAANHSHVCABVTGHSH	DSDCABVTGBDSDNTTYCCHD	Kulakovskiy	2013	23175603
LDEC016433	zf-C2H2,zf-C2H2,zf-C2H2,zf-C2H2,zf-C2H2	*Drosophila melanogaster*	FBgn0035625	20	0.829	B1H	RCTTTCMCTTTY	RAAAGKGAAAGY	Zhu	2011	21097781
LDEC016518	Homeobox	*Drosophila melanogaster*	FBgn0010323	15	0.737	PBM	NRGATTAN	NTAATCYN	Weirauch	2014	25215497
LDEC016582	Homeobox	*Drosophila melanogaster*	FBgn0020617	12	0.789	B1H	YTAATTR	YAATTAR	Mathelier	2014	24194598
LDEC016907	Homeobox	*Drosophila melanogaster*	FBgn0027364	6	0.889	B1H	TGABAC	GTVTCA	Mathelier	2014	24194598
LDEC017129	Homeobox	*Drosophila melanogaster*	FBgn0000099	4	0.930	PBM	NHHDTTRNNN	NNNYAAHDDN	Weirauch	2014	25215497
LDEC017209	Homeobox	*Drosophila melanogaster*	FBgn0040318	13	0.772	B1H	DTAATKA	TMATTAH	Mathelier	2014	24194598
LDEC017422	zf-C2H2,zf-C2H2,zf-C2H2	*Drosophila melanogaster*	FBgn0261434	13	0.817	B1H	TCACGCCYM	KRGGCGTGA	Mathelier	2014	24194598
LDEC017472	HLH	*Drosophila melanogaster*	FBgn0039411	4	0.923	B1H	RDDTCGTGAYY	RRTCACGAHHY	Zhu	2011	21097781
LDEC017703	bZIP_1	*Homo sapiens*	ENSG00000172216	22	0.656	PBM	VTTRCRHMAB	VTKDYGYAAB	Weirauch	2014	25215497
LDEC018156	zf-C2H2,zf-C2H2,zf-C2H2,zf-C2H2	*Drosophila melanogaster*	FBgn0036179	11	0.880	B1H	HHCCCCCHCCCMCHM	KDGKGGGDGGGGGDD	Zhu	2011	21097781

LDEC018359	LAG1-DNAbind	*Drosophila melanogaster*	FBgn0004837	14	0.895	Transfac	RBYGTGRGAAMCB	VGKTTCYCACRVY	Matys	2006	16381825
LDEC018714	bZIP_1	*Mus musculus*	ENSMUSG00000034271	26	0.594	PBM	NNNHRTCAYN	NRTGAYDNNN	Badis	2009	19443739
LDEC018804	HLH	*Drosophila melanogaster*	FBgn0023091	7	0.868	B1H	HCCATATGGYG	CRCCATATGGD	Zhu	2011	21097781
LDEC018805	Homeobox	*Drosophila melanogaster*	FBgn0000492	2	0.965	B1H	SCAATTA	TAATTGS	Mathelier	2014	24194598
LDEC018842	Fork_head	*Drosophila melanogaster*	FBgn0262477	45	0.494	B1H	NNWTTTTTTYNNNNN	NNNNNRAAAAAAWNN	Zhu	2011	21097781
LDEC018964	HLH	*Drosophila melanogaster*	FBgn0002931	11	0.792	B1H	VCAGRTGKB	VMCAYCTGB	Zhu	2011	21097781
LDEC019090	zf-C2H2,zf-C2H2,zf-C2H2,zf-C2H2	*Drosophila melanogaster*	FBgn0036179	24	0.739	B1H	HHCCCCCHCCCMCHM	KDGKGGGDGGGGGDD	Zhu	2011	21097781
LDEC019364	GATA	*Drosophila melanogaster*	FBgn0032223	6	0.829	B1H	TGATAASS	SSTTATCA	Zhu	2011	21097781
LDEC019440	Pou,Homeobox	*Homo sapiens*	ENSG00000152192	33	0.756	Transfac	DBMAYAAVTHA	THABTTRTKBH	Matys	2006	16381825
LDEC019549	E2F_TDP	*Homo sapiens*	ENSG00000205250	9	0.866	Transfac	GCGSGARRDA	THYYTCSCGC	Matys	2006	16381825
LDEC019585	Homeobox	*Drosophila melanogaster*	FBgn0024184	14	0.754	B1H	YTAATTR	YAATTAR	Mathelier	2014	24194598
LDEC019709	Homeobox	*Drosophila melanogaster*	FBgn0052105	5	0.912	B1H	YTAATTA	TAATTAR	Mathelier	2014	24194598
LDEC020077	Fork_head	*Homo sapiens*	ENSG00000187140	7	0.927	Transfac	DWWTRTTTDHTY	RADHAAAYAWWH	Matys	2006	16381825
LDEC020561	Homeobox	*Drosophila melanogaster*	FBgn0011701	2	0.965	B1H	TTAATTA	TAATTAA	Mathelier	2014	24194598
LDEC020805	Homeobox	*Anopheles gambiae*	AGAP009986	9	0.850	PBM	NNNNTGTHNN	NNDACANNNN	Weirauch	2014	25215497
LDEC020812	zf-C2H2,zf-C2H2,zf-C2H2,zf-C2H2,zf-C2H2	*Homo sapiens*	ENSG00000119866	28	0.763	Transfac	YTCWBTTYY	RRAAVWGAR	Matys	2006	16381825
LDEC021031	Fork_head	*Homo sapiens*	ENSG00000171956	13	0.865	PBM	TRTTKAYWYW	WRWRTMAAYA	Weirauch	2014	25215497
LDEC021054	HLH	*Drosophila melanogaster*	FBgn0001168	3	0.948	Transfac	NNBVCACGCGVSHN	NDSBCGCGTGBVNN	Matys	2006	16381825
LDEC021156	Homeobox	*Homo sapiens*	ENSG00000143355	3	0.935	SELEX	TAATKRCYAATTA	TAATTRGYMATTA	Jolma	2013	23332764
LDEC021157	Homeobox	*Drosophila melanogaster*	FBgn0000099	0	1.000	Transfac	DNNATTDK	MHAATNNH	Matys	2006	16381825
LDEC021181	HLH	*Drosophila melanogaster*	FBgn0259938	9	0.836	B1H	RTCACGTGACCR	YGGTCACGTGAY	Zhu	2011	21097781
LDEC021566	Homeobox	*Drosophila melanogaster*	FBgn0020912	13	0.772	PBM	NTAATCYNN	NNRGATTAN	Weirauch	2014	25215497
LDEC021908	Fork_head	*Drosophila melanogaster*	FBgn0014143	6	0.938	Transfac	BNNNMTATTTAYYNWD	HWNARTAAATAKNNNV	Matys	2006	16381825
LDEC022044	zf-C4	*Drosophila pseudoobscura*	FBgn0246284	1	0.986	PBM	RAGATCAM	KTGATCTY	Weirauch	2014	25215497

LDEC022429	Homeobox	*Homo sapiens*	ENSG00000136944	3	0.947	SELEX	DTAATTRV	BYAATTAH	Jolma	2013	23332764
LDEC022528	Ets	*Homo sapiens*	ENSG00000154727	36	0.571	PBM	RSCGGAARY	RYTTCCGSY	Wei	2010	20517297
LDEC022561	Ets	*Drosophila melanogaster*	FBgn0005658	5	0.940	PBM	NNVMGGAWRN	NYWTCCKBNN	Wei	2010	20517297
LDEC023181	Fork_head	*Drosophila melanogaster*	FBgn0004895	26	0.761	B1H	TGTTTWBM	KVWAAACA	Zhu	2011	21097781
LDEC023547	GATA	*Drosophila melanogaster*	FBgn0032223	7	0.800	B1H	TGATAASS	SSTTATCA	Zhu	2011	21097781
LDEC023792	HLH	*Drosophila melanogaster*	FBgn0032651	1	0.982	B1H	MCATCTGKY	RMCAGATGK	Zhu	2011	21097781
LDEC024027	HLH	*Drosophila melanogaster*	FBgn0003270	8	0.849	B1H	RMCAYMTGBYV	BRVCAKRTGKY	Zhu	2011	21097781

1 DNA binding domains contained within the TF (N to C terminus order). PFAM IDs are provided.
2 Species from which the DNA binding motif was mapped.
3 Gene from which the DNA binding motif was mapped.
4 Number of mismatches in the amino acid alignment between the two DBDs.
5 Percent identity between the two DBDs (i.e., the fraction of identical amino acids in the DBD alignment).
6 Type of experiment used to derive the motif. B1H = Bacteria one-hypbrid; Compiled = compiled from various sources; PBM = protein binding microarray.
7 IUPAC sequence of the DNA binding motif (forward direction).
8 IUPAC sequence of the DNA binding motif (reverse direction).

Table 5S. Significantly differentially expressed genes, after Bonferroni correction, in mid-gut versus whole larval tissues of *Leptinotarsa decemlineata*.

Feature ID	Gene name	Experiment - Fold Change (normalized values)	Larvae (GE) - Normalized expression values	Gut (GE) - Normalized expression values
LDEC000066-RA	cuticular protein 27a	-66932.58	2614.43	0.04
LDEC000075-RA	flexible cuticle protein 12	-20725.68	4047.79	0.20
LDEC000078-RA	hypothetical protein TcasGA2_TC002836	-11499.69	17967.38	1.56
LDEC000222-RA	cuticle protein cp5	-6534.60	255.25	0.04
LDEC000273-RA	---NA---	-5620.48	1317.24	0.23
LDEC000483-RA	larval cuticle protein lcp-30	-5406.58	4434.87	0.82
LDEC000493-RA	---NA---	-4628.19	180.78	0.04
LDEC000576-RA	cuticle protein cp5	-4604.64	359.72	0.08
LDEC000616-RA	cuticle protein	-4373.42	2220.77	0.51
LDEC000675-RA	endocuticle structural glycoprotein bd-1-like	-4312.17	3031.84	0.70
LDEC000682-RA	ribosomal protein s27e	-37127	371.27	0.00
LDEC000701-RA	---NA---	-2914.56	24931.96	8.55
LDEC000826-RA	40s ribosomal protein s15	-2588.82	101.12	0.04
LDEC000846-RA	larval pupal cuticle protein h1c	-2416.50	377.56	0.16
LDEC000850-RA	glycine-rich cell wall structural	-2316.26	5971.33	2.58
LDEC000918-RA	larval cuticle protein 8-like	-2043.47	1117.47	0.55
LDEC000922-RA	cytosolic carboxypeptidase 6	-1770.10	967.98	0.55
LDEC000982-RA	atp-binding cassette sub-family b member mitochondrial	-1697.10	596.61	0.35
LDEC001040-RA	cytochrome p450	-1589.11	7448.59	4.69
LDEC001049-RA	larval pupal cuticle protein h1c	-1162.07	680.87	0.59
LDEC001064-RA	---NA---	-950.26	74.24	0.08
LDEC001178-RA	maltase 2-like	-889.43	486.38	0.55
LDEC001179-RA	fatty acyl- reductase cg5065	-718.54	84.20	0.12
LDEC001198-RA	larval cuticle protein lcp-17-like	-694.11	1680.96	2.42
LDEC001223-RA	hypothetical protein IscW_ISCW024931	-527.10	823.55	1.56
LDEC001224-RA	larval cuticle protein lcp-17-like	-498.19	194.60	0.39
LDEC001352-RA	adfb like protein	-456.17	231.64	0.51
LDEC001356-RA	larval cuticle protein a2b-like	-446.20	296.29	0.66
LDEC001389-RA	---NA---	-424.14	298.21	0.70
LDEC001394-RA	transmembrane protein 63a	-41.87	41.87	0.00
LDEC001405-RA	larval cuticle protein lcp-30	-399.87	406.09	1.02
LDEC001536-RA	endocuticle structural glycoprotein bd-2-like	-384.83	210.44	0.55
LDEC001644-RA	quaking related	-374.15	43.84	0.12

LDEC001677-RA	---NA---	-344.02	1034.70	3.01
LDEC001678-RA	abp1_ripcl ame: full=probable antibacterial peptide polyprotein	-300.92	152.80	0.51
LDEC001728-RA	plasma membrane calcium-transporting atpase	-284.67	122.31	0.43
LDEC001766-RA	cuticular protein cpr2	-281.23	43.94	0.16
LDEC001912-RA	hypothetical protein D910_05825	-250.36	244.48	0.98
LDEC001996-RA	flexible cuticle protein 12-like	-236.74	64.73	0.27
LDEC002052-RA	attacin-like immune protein	-235.32	1296.06	5.51
LDEC002053-RA	acyl- delta desaturase-like	-235.05	73.45	0.31
LDEC002055-RA	mucin- partial	-226.72	309.95	1.37
LDEC002056-RA	antifreeze protein maxi-like	-215.26	75.67	0.35
LDEC002058-RA	b1 protein	-214.20	744.64	3.48
LDEC002059-RA	fatty acid synthase- partial	-205.47	48.16	0.23
LDEC002196-RA	cathepsin l precursor	-202.61	63.31	0.31
LDEC002245-RA	---NA---	-199.78	343.35	1.72
LDEC002280-RA	chorion protein s38	-183.12	407.70	2.23
LDEC002333-RA	hypothetical protein IscW_ISCW024931	-181.44	333.10	1.84
LDEC002407-RA	agap006497-pa-like protein	-168.33	1045.43	6.21
LDEC002439-RA	fatty acid synthase	-162.94	50.91	0.31
LDEC002468-RA	cuticular protein analogous to peritrophins 3-e	-161.64	271.50	1.68
LDEC002522-RA	protein takeout	-160.58	137.99	0.86
LDEC002525-RA	cuticle protein 65-like	-160.05	50.01	0.31
LDEC002545-RA	aldo-keto reductase	-149.00	448.14	3.01
LDEC002632-RA	cuticle protein 1	-147.48	92.17	0.62
LDEC002682-RA	4-hydroxyphenylpyruvate dioxygenase	-146.76	424.22	2.89
LDEC002907-RA	fatty acyl- reductase cg5065-like	-144.13	28.15	0.20
LDEC003080-RA	fatty acyl- reductase 1	-141.22	110.32	0.78
LDEC003087-RA	---NA---	-141.17	452.16	3.20
LDEC003102-RA	zinc finger protein 512b	-134.97	47.45	0.35
LDEC003166-RA	attacin-like immune protein	-130.25	925.92	7.11
LDEC003202-RA	larval cuticle protein 8-like	-126.95	44.63	0.35
LDEC003318-RA	PREDICTED: uncharacterized protein LOC655864	-126.13	98.53	0.78
LDEC003320-RA	cytochrome p450 cyp4g56	-124.82	112.14	0.90
LDEC003321-RA	peroxidasin homolog	-119.84	154.47	1.29
LDEC003447-RA	hypothetical protein DAPPUDRAFT_105533	-116.14	176.93	1.52
LDEC003490-RA	indole-3-acetaldehyde oxidase-like	-114.97	143.70	1.25
LDEC003727-RA	adult cuticle protein 1-like	-112.86	149.89	1.33
LDEC003745-RA	pupal cuticle protein 20	-111.55	104.57	0.94
LDEC003808-RA	tyrosine aminotransferase	-109.04	362.02	3.32
LDEC003996-RA	larval cuticle protein lcp-30	-107.61	58.85	0.55
LDEC004409-RA	eukaryotic translation initiation factor 3 subunit a	-104.27	81.46	0.78

LDEC004508-RA	c-type lectin 5	-104.17	638.84	6.13
LDEC004521-RA	cuticular protein 92f	-103.65	157.90	1.52
LDEC004537-RA	fatty acid synthase	-95.79	119.73	1.25
LDEC004552-RA	fatty acid synthase	-95.29	338.70	3.55
LDEC004553-RA	alanine--glyoxylate aminotransferase 2-like	-94.92	478.30	5.04
LDEC004665-RA	---NA---	-94.64	417.72	4.41
LDEC004866-RA	elongation of very long chain fatty acids protein 7	-94.28	176.77	1.87
LDEC005075-RA	fatty acyl- reductase 1-like	-94.15	209.62	2.23
LDEC005215-RA	fatty acyl- reductase cg5065	-93.80	175.87	1.87
LDEC005220-RA	fatty acid synthase	-91.26	192.49	2.11
LDEC005245-RA	40s ribosomal protein sa	-90.55	265.27	2.93
LDEC005337-RA	ejaculatory bulb-specific protein 3	-89.18	2281.73	25.58
LDEC005367-RA	hypothetical protein L798_04546	-85.98	87.32	1.02
LDEC005396-RA	myosin regulatory light chain 2	-84.99	637.39	7.50
LDEC005425-RA	apolipoprotein d-like	-84.96	76.32	0.90
LDEC005468-RA	PREDICTED: uncharacterized protein LOC655532 isoform X2	-84.63	178.50	2.11
LDEC005683-RA	agap012703-pa-like protein	-84.18	65.77	0.78
LDEC005684-RA	diapause-associated transcript-2	-83.80	85.10	1.02
LDEC005689-RA	isoform a	-83.66	163.40	1.95
LDEC005691-RA	integument esterase	-82.24	144.56	1.76
LDEC005719-RA	ice-structuring glycoprotein	-81.91	73.58	0.90
LDEC005754-RA	odorant binding partial	-81.87	51.16	0.62
LDEC005786-RA	fibril-forming collagen alpha chain-like	-76.96	667.39	8.67
LDEC005787-RA	apolipoprotein d-like	-75.67	434.49	5.74
LDEC005788-RA	hypothetical protein TcasGA2_TC015372	-72.72	332.32	4.57
LDEC005826-RA	venom acid phosphatase acph-1-like	-72.25	174.97	2.42
LDEC005827-RA	protein takeout	-72.14	608.62	8.44
LDEC005855-RA	hydroxyacid oxidase 1	-70.45	365.99	5.20
LDEC005947-RA	antifreeze protein maxi	-70.37	63.22	0.90
LDEC006043-RA	---NA---	-70.35	1780.72	25.31
LDEC006073-RA	pupal cuticle protein 36-like isoform x2	-70.07	353.09	5.04
LDEC006077-RA	muscle actin	-68.39	20252.10	296.12
LDEC006230-RA	venom acid phosphatase acph-1	-66.66	151.02	2.27
LDEC006357-RA	aldo-keto reductase	-65.71	43.63	0.66
LDEC006388-RA	esterase	-61.81	45.88	0.74
LDEC006415-RA	mesenchymal stem cell protein dscd75	-61.60	89.03	1.45
LDEC006416-RA	cartilage oligomeric matrix protein	-60.79	244.55	4.02
LDEC006479-RA	---NA---	-59.85	58.45	0.98
LDEC006484-RA	myofilin variant a	-59.73	1336.78	22.38
LDEC006517-RA	troponin t isoform x2	-59.40	4912.19	82.69

LDEC006531-RA	proclotting enzyme	-59.27	402.84	6.80
LDEC006671-RA	isoform a	-58.00	38.52	0.66
LDEC006673-RA	chymotrypsin-c-like isoform x1	-57.96	149.43	2.58
LDEC006704-RA	cathepsin l	-56.50	37.52	0.66
LDEC006718-RA	gb12811-like partial	-56.49	150.04	2.66
LDEC006847-RA	collagen alpha-1 chain-like	-56.42	39.67	0.70
LDEC006864-RA	chitin deacetylase 1 precursor	-56.21	935.27	16.64
LDEC007026-RA	cytochrome p450 4g15-like	-56.20	79.03	1.41
LDEC007095-RA	troponin i	-55.50	1547.97	27.89
LDEC007268-RA	attacin-like immune protein	-54.98	912.64	16.60
LDEC007385-RA	glucose dehydrogenase	-54.64	27.75	0.51
LDEC007413-RA	xanthine dehydrogenase oxidase	-52.75	150.41	2.85
LDEC007486-RA	hypothetical protein YQE_00903, partial	-52.52	582.56	11.09
LDEC007487-RA	isoform a	-52.20	114.19	2.19
LDEC007549-RA	fatty acid synthase	-51.76	703.52	13.59
LDEC007554-RA	cytochrome p450 4c3-like	-51.51	88.53	1.72
LDEC007594-RA	PREDICTED: uncharacterized protein LOC661334 isoform X1	-50.82	67.49	1.33
LDEC007613-RA	trans- -dihydrobenzene- -diol dehydrogenase-like	-50.72	35.66	0.70
LDEC007614-RA	PREDICTED: uncharacterized protein LOC661334 isoform X2	-50.68	53.44	1.05
LDEC007768-RA	abc transporter	-49.97	56.61	1.13
LDEC007802-RA	mitochondrial thioredoxin 2	-49.91	64.33	1.29
LDEC007843-RA	mdl2	-49.29	125.15	2.54
LDEC007940-RA	acyl- synthetase short-chain family member mitochondrial	-49.25	246.24	5.00
LDEC008122-RA	gamma-interferon-inducible lysosomal thiol reductase	-48.87	104.99	2.15
LDEC008123-RA	cartilage oligomeric matrix protein	-48.58	259.94	5.35
LDEC008144-RA	angiotensin-converting enzyme-like	-47.74	104.42	2.19
LDEC008160-RA	probable salivary secreted peptide	-47.62	535.65	11.25
LDEC008164-RA	odorant-binding protein 17	-47.42	548.32	11.56
LDEC008201-RA	tropomyosin- partial	-47.28	864.27	18.28
LDEC008225-RA	inorganic phosphate cotransporter	-46.25	74.06	1.60
LDEC008247-RA	ribonucleoside-diphosphate reductase large subunit	-45.98	104.17	2.27
LDEC008327-RA	troponin c	-45.92	229.61	5.00
LDEC008347-RA	prostaglandin e synthase 3	-43.82	280.71	6.41
LDEC008388-RA	glycoside hydrolase family 1	-43.69	34.13	0.78
LDEC008408-RA	glutamate semialdehyde dehydrogenase	-43.41	125.48	2.89
LDEC008413-RA	glutathione s-transferase epsilon	-43.30	131.92	3.05
LDEC008460-RA	actin	-43.03	803.45	18.67
LDEC008494-RA	retinol dehydrogenase 14	-42.59	28.28	0.66
LDEC008660-RA	blackjack	-41.94	743.78	17.73
LDEC008757-RA	stabilin-1 isoform x1	-41.85	148.76	3.55

LDEC008795-RA	isoform c	-41.71	123.81	2.97
LDEC008944-RA	cytochrome p450 9z4	-41.46	63.16	1.52
LDEC009076-RA	prostatic acid phosphatase	-39.92	344.60	8.63
LDEC009139-RA	larval cuticle protein lcp-17-like	-39.89	35.83	0.90
LDEC009143-RA	juvenile hormone partial	-39.81	287.69	7.23
LDEC009144-RA	60s ribosomal protein l7a-like	-39.76	549.74	13.83
LDEC009250-RA	apolipophorin- partial	-39.75	1046.58	26.33
LDEC009289-RA	prostatic acid phosphatase	-39.29	173.42	4.41
LDEC009315-RA	---NA---	-39.08	221.37	5.66
LDEC009414-RA	protein takeout-like	-38.48	166.83	4.34
LDEC009420-RA	glucose dehydrogenase	-38.22	101.50	2.66
LDEC009430-RA	elongation factor 1-alpha isoform x1	-38.20	10023.35	262.41
LDEC009552-RA	probable serine hydrolase isoform x5	-37.49	79.08	2.11
LDEC009553-RA	tropomyosin 1	-37.49	909.34	24.26
LDEC009558-RA	93 kda serpin	-37.47	143.43	3.83
LDEC009631-RA	40s ribosomal protein s15	-37.46	357.00	9.53
LDEC009708-RA	uracil-dna degrading isoform b	-37.37	217.48	5.82
LDEC009735-RA	collagen alpha-1 chain	-37.23	228.28	6.13
LDEC009889-RA	agap006502-pa-like protein	-37.22	114.86	3.09
LDEC009896-RA	cytochrome p450-like protein	-37.06	112.93	3.05
LDEC009984-RA	40s ribosomal protein s2	-36.90	2399.69	65.04
LDEC010001-RA	hypothetical protein EAI_07617	-36.72	162.10	4.41
LDEC010126-RA	elongation of very long chain fatty acids protein aael008004-like	-36.28	34.01	0.94
LDEC010139-RA	serine protease	-36.18	98.94	2.73
LDEC010368-RA	diapause-associated transcript-2	-35.86	558.93	15.59
LDEC010397-RA	cuticular protein rr-1 family	-35.68	83.63	2.34
LDEC010425-RA	protein anoxia up-regulated isoform x4	-35.63	211.52	5.94
LDEC010502-RA	udp-glucuronosyltransferase 2c1-like	-35.56	109.72	3.09
LDEC010503-RA	delta-1-pyrroline-5-carboxylate synthase	-35.53	124.92	3.52
LDEC010521-RA	pro-phenol oxidase subunit 2	-35.24	320.72	9.10
LDEC010794-RA	lectin subunit alpha-like	-34.53	94.41	2.73
LDEC010852-RA	acyl- delta desaturase-like	-34.40	83.30	2.42
LDEC010880-RA	collagen alpha-5 chain	-33.90	582.66	17.19
LDEC010881-RA	paxillin isoform x5	-33.90	266.17	7.85
LDEC011071-RA	juvenile hormone partial	-33.85	306.75	9.06
LDEC011160-RA	prostatic acid phosphatase-like	-33.81	335.40	9.92
LDEC011221-RA	cytochrome p450	-33.46	98.02	2.93
LDEC011294-RA	endocuticle structural glycoprotein bd-8-like	-33.26	59.77	1.80
LDEC011312-RA	tryptophan oxygenase	-33.05	47.77	1.45
LDEC011313-RA	aldo-keto reductase family 4 member c9-like	-32.93	90.03	2.73

LDEC011381-RA	chitin-binding protein	-32.92	204.45	6.21
LDEC011382-RA	serpin b6	-32.81	41.01	1.25
LDEC011383-RA	pathogenesis-related protein 5	-32.80	51.24	1.56
LDEC011384-RA	chitin deacetylase 2 isoform b precursor	-32.42	614.10	18.94
LDEC011422-RA	antifreeze protein maxi	-32.28	50.44	1.56
LDEC011511-RA	protein disulfide-isomerase	-31.80	233.53	7.34
LDEC011512-RA	ump-cmp kinase	-31.41	117.77	3.75
LDEC011657-RA	ef-hand calcium-binding domain-containing protein 1	-31.37	1002.28	31.95
LDEC011658-RA	---NA---	-31.09	64.37	2.07
LDEC011960-RA	mast cell protease 6	-30.79	134.69	4.37
LDEC011988-RA	hypothetical protein D910_06903	-30.71	39.59	1.29
LDEC012045-RA	venom allergen 5-like	-30.69	115.09	3.75
LDEC012092-RA	serine protease h164	-30.61	659.98	21.56
LDEC012171-RA	niemann-pick type c-2a	-30.22	108.59	3.59
LDEC012277-RA	endonuclease and reverse transcriptase-like protein	-30.21	132.18	4.37
LDEC012285-RA	PREDICTED: uncharacterized protein LOC662719	-29.54	71.53	2.42
LDEC012585-RA	scavenger receptor class b member 1	-29.35	162.77	5.55
LDEC012625-RA	low quality protein: cell wall protein tir4-like	-29.27	30.87	1.05
LDEC012644-RA	isoform a	-29.16	83.15	2.85
LDEC012661-RA	heat shock protein	-29.01	40.80	1.41
LDEC012765-RA	protein disulfide-isomerase	-28.97	70.15	2.42
LDEC012813-RA	protein takeout-like	-28.62	2494.91	87.18
LDEC012814-RA	glycine cleavage system h mitochondrial isoform x2	-28.55	78.05	2.73
LDEC012846-RA	collagen alpha-1 chain-like	-28.23	116.89	4.14
LDEC013070-RA	myosin light chain 2	-28.04	4293.74	153.12
LDEC013186-RA	isoform a	-27.98	55.74	1.99
LDEC013397-RA	atp-dependent rna helicase wm6	-27.98	42.62	1.52
LDEC013494-RA	---NA---	-27.94	174.59	6.25
LDEC013723-RA	venom acid phosphatase acph-1-like	-27.47	85.85	3.12
LDEC013732-RA	isoform a	-27.39	51.36	1.87
LDEC013799-RA	juvenile hormone binding protein partial	-27.04	54.92	2.03
LDEC013885-RA	c-1-tetrahydrofolate cytoplasmic isoform x2	-26.92	77.82	2.89
LDEC013889-RA	udp-glucuronosyltransferase 2b9	-26.90	246.97	9.18
LDEC014022-RA	heat shock cognate 70	-26.86	1010.23	37.62
LDEC014063-RA	cuticular protein analogous to peritrophins 3-d1	-26.77	34.51	1.29
LDEC014138-RA	40s ribosomal protein s2	-26.75	50.15	1.87
LDEC014178-RA	protein yellow-like	-26.42	28.90	1.09
LDEC014265-RA	serine protease snake-like	-26.36	120.48	4.57
LDEC014268-RA	yellow- partial	-25.98	55.82	2.15
LDEC014277-RA	udp-glucuronosyltransferase 2b20-like	-25.90	114.30	4.41

LDEC014425-RA	argininosuccinate synthase	-25.73	56.28	2.19
LDEC014505-RA	pacifastin-like protease inhibitor cvp4	-25.71	127.55	4.96
LDEC014520-RA	mucin 12ea	-25.66	607.43	23.67
LDEC014544-RA	heat shock protein 70	-25.55	1255.59	49.14
LDEC014547-RA	lipase 3	-25.46	247.66	9.73
LDEC014553-RA	27 kda hemolymph protein	-25.27	240.82	9.53
LDEC014696-RA	pr-5-like protein	-25.21	36.43	1.45
LDEC014789-RA	tyrosine-protein phosphatase non-receptor type 23-like	-25.15	29.47	1.17
LDEC014900-RA	cytochrome p450 307a1	-25.06	67.53	2.70
LDEC015149-RA	peptidoglycan-recognition protein s2	-25.00	60.55	2.42
LDEC015194-RA	gustatory receptor candidate 59	-24.95	46.78	1.87
LDEC015196-RA	mast cell protease 6	-24.94	227.00	9.10
LDEC015595-RA	collagen alpha- chain	-24.94	2547.63	102.14
LDEC015650-RA	isoform a	-24.66	124.25	5.04
LDEC015651-RA	elongation of very long chain fatty acids protein 1-like	-24.49	381.76	15.59
LDEC015776-RA	tropomyosin 1	-24.22	1313.98	54.26
LDEC015787-RA	eukaryotic translation initiation factor 4b-like	-24.11	240.18	9.96
LDEC015907-RA	5 nucleotidase	-24.07	45.13	1.87
LDEC016089-RA	alpha- sarcomeric isoform x1	-24.06	38.54	1.60
LDEC016130-RA	---NA---	-23.97	1246.05	51.99
LDEC016254-RA	sphingomyelin phosphodiesterase-like	-23.94	50.49	2.11
LDEC016324-RA	93 kda serpin	-23.43	46.68	1.99
LDEC016412-RA	skin secretory protein xp2-like	-23.36	40.15	1.72
LDEC016524-RA	salivary c-type lectin	-23.34	101.20	4.34
LDEC016562-RA	glucose dehydrogenase	-23.31	92.88	3.98
LDEC016579-RA	odorant-binding protein 18	-23.24	215.18	9.26
LDEC016751-RA	protein takeout	-23.08	82.94	3.59
LDEC016975-RA	---NA---	-22.83	461.86	20.23
LDEC017119-RA	abc transporter g family member 23	-22.51	50.11	2.23
LDEC017128-RA	---NA---	-22.48	567.37	25.23
LDEC017168-RA	GA15068	-22.40	105.87	4.73
LDEC017229-RA	voltage-dependent anion channel	-22.22	335.88	15.12
LDEC017308-RA	60s ribosomal protein l8	-22.02	1027.96	46.68
LDEC017569-RA	PREDICTED: uncharacterized protein LOC103313125	-22.00	33.52	1.52
LDEC017626-RA	msr- isoform d	-21.83	151.81	6.95
LDEC017718-RA	abc transporter g family member 23	-21.78	34.03	1.56
LDEC017723-RA	arylsulfatase b	-21.75	32.29	1.48
LDEC017726-RA	isoform a	-21.72	65.33	3.01
LDEC017886-RA	lipase 3	-21.67	122.74	5.66
LDEC017913-RA	ubiquitin carboxyl-terminal hydrolase 2 isoform x2	-21.67	195.50	9.02

LDEC017972-RA	agap009935-pa-like protein	-21.52	36.14	1.68
LDEC018059-RA	integument esterase	-21.51	100.83	4.69
LDEC018060-RA	pdz and lim domain protein 1 isoform x2	-21.47	204.64	9.53
LDEC018144-RA	alpha- sarcomeric	-21.45	394.56	18.40
LDEC018364-RA	nucleolar protein 58-like	-21.44	158.26	7.38
LDEC018545-RA	myosin light chain alkali isoform x1	-21.44	1414.26	65.97
LDEC018586-RA	nuclear pore complex protein ddb_g0274915 homolog isoform x1	-21.35	178.44	8.36
LDEC018703-RA	mitochondrial import inner membrane translocase subunit tim8	-21.10	35.43	1.68
LDEC018833-RA	40s ribosomal protein s2	-21.09	95.54	4.53
LDEC018935-RA	isoform a	-20.95	221.75	10.59
LDEC018941-RA	protein takeout-like	-20.86	72.53	3.48
LDEC019098-RA	-like protein partial	-20.76	144.33	6.95
LDEC019328-RA	39s ribosomal protein mitochondrial	-20.76	103.78	5.00
LDEC019355-RA	chromobox protein homolog 5	-20.72	428.86	20.70
LDEC019539-RA	protein yellow-like	-20.50	37.64	1.84
LDEC019589-RA	60s ribosomal protein l27a	-20.47	327.89	16.01
LDEC019765-RA	atp-citrate synthase isoform x1	-20.30	1088.61	53.63
LDEC019971-RA	fibulin 1 and	-20.29	96.68	4.77
LDEC020095-RA	long-chain fatty acid transport protein 4	-20.26	117.12	5.78
LDEC020119-RA	prostatic acid phosphatase-like	-20.25	168.52	8.32
LDEC020286-RA	15-hydroxyprostaglandin dehydrogenase	-20.23	37.14	1.84
LDEC020383-RA	15-hydroxyprostaglandin dehydrogenase	-20.16	207.88	10.31
LDEC020472-RA	---NA---	-20.13	56.61	2.81
LDEC020740-RA	40s ribosomal protein s8	-20.08	157.69	7.85
LDEC020822-RA	troponin c ia	-20.06	425.52	21.21
LDEC020877-RA	serine protease h3	-20.03	187.74	9.37
LDEC020878-RA	chitinase 10 precursor	-20.01	168.06	8.40
LDEC021063-RA	alpha-tocopherol transfer	-19.91	91.79	4.61
LDEC021064-RA	acetyl- carboxylase isoform x3	-19.87	131.97	6.64
LDEC021068-RA	fatty acid synthase	-19.58	1414.60	72.26
LDEC021135-RA	calcium-transporting atpase sarcoplasmic endoplasmic reticulum type isoform x1	-19.51	1966.54	100.82
LDEC021189-RA	angiotensin-converting enzyme-like	-19.34	103.50	5.35
LDEC021195-RA	probable small nuclear ribonucleoprotein e	-19.27	39.90	2.07
LDEC021196-RA	inorganic phosphate cotransporter	-19.16	219.99	11.48
LDEC021198-RA	ribosomal protein l24e	-19.05	496.27	26.05
LDEC021200-RA	arylsulfatase b-like	-19.02	98.80	5.20
LDEC021274-RA	luciferin-regenerating partial	-19.02	78.74	4.14
LDEC021542-RA	mitochondrial carnitine acylcarnitine carrier protein cacl	-18.92	48.77	2.58
LDEC021699-RA	outer dense fiber protein 3 isoform x1	-18.86	215.10	11.41

LDEC021825-RA	inorganic phosphate cotransporter	-18.86	310.11	16.44
LDEC021826-RA	hypothetical protein YQE_08768, partial	-18.83	41.18	2.19
LDEC021900-RA	excitatory amino acid transporter 3	-18.77	36.66	1.95
LDEC021911-RA	juvenile hormone acid o-methyltransferase	-18.64	50.24	2.70
LDEC021928-RA	24-dehydrocholesterol reductase	-18.52	31.83	1.72
LDEC021944-RA	ubiquitin-conjugating enzyme e2 c	-18.42	69.79	3.79
LDEC022358-RA	myosin heavy chain	-18.41	10838.33	588.64
LDEC022798-RA	adipocyte plasma membrane-associated	-18.39	522.33	28.40
LDEC022799-RA	lysozyme precursor	-18.37	76.78	4.18
LDEC022800-RA	AGAP006960-PA	-18.31	33.61	1.84
LDEC022801-RA	ubiquitin-40s ribosomal protein s27a	-18.28	184.19	10.08
LDEC022883-RA	small heat shock protein	-18.23	229.97	12.62
LDEC023414-RA	zinc finger c2hc domain-containing protein 1b-like	-18.23	46.99	2.58
LDEC023602-RA	transcriptional regulator def1	-18.13	361.90	19.96
LDEC023802-RA	muscle lim protein mlp84b-like isoform x1	-18.09	2046.06	113.12
LDEC023804-RA	isoform c	-18.04	71.15	3.95
LDEC024202-RA	10 kda heat shock mitochondrial	-18.02	149.93	8.32
LDEC024360-RA	cuticular protein analogous to peritrophins 1-c precursor	-17.98	136.27	7.58
LDEC024548-RA	protein yellow-like	-17.94	57.47	3.20
LDEC024629-RA	nucleolar and coiled-body phosphoprotein 1-like isoform x3	-17.94	489.07	27.26
LDEC003536-RA	high molecular weight subunit dx5-like	-17.90	157.34	8.79
LDEC021669-RA	60s acidic ribosomal protein p2	-17.85	618.40	34.65
LDEC011288-RA	pxcc family protein	-17.77	61.07	3.44
LDEC000809-RA	hydroxyacyl-coenzyme a mitochondrial	-17.76	301.02	16.95
LDEC005727-RA	serine threonine-protein kinase polo	-17.75	66.55	3.75
LDEC003972-RA	serine protease easter	-17.72	116.97	6.60
LDEC015348-RA	protein isoforms b c	-17.64	71.65	4.06
LDEC017475-RA	sodium-coupled monocarboxylate transporter 1	-17.60	202.76	11.52
LDEC013324-RA	28 kda desiccation stress protein precursor	-17.60	472.86	26.87
LDEC003400-RA	PREDICTED: myophilin	-17.55	265.36	15.12
LDEC010285-RA	nucleosome assembly protein 1-like 1	-17.39	632.25	36.37
LDEC005045-RA	low quality protein: short form-like	-17.37	2907.48	167.37
LDEC006478-RA	c-1-tetrahydrofolate cytoplasmic	-17.30	139.20	8.05
LDEC009322-RA	scavenger receptor class b member 1	-17.30	100.68	5.82
LDEC011579-RA	pou domain protein cf1a	-17.27	48.56	2.81
LDEC015349-RA	fatty acyl- reductase cg5065 isoform x2	-17.14	62.28	3.63
LDEC003990-RA	d-beta-hydroxybutyrate mitochondrial	-17.13	80.31	4.69
LDEC011656-RA	---NA---	-17.07	960.41	56.25
LDEC009158-RA	nucleolar protein 56	-17.02	228.71	13.44
LDEC022001-RA	PREDICTED: uncharacterized protein	-16.99	82.97	4.88

	LOC103312569			
LDEC009934-RA	regucalcin-like isoform x1	-16.98	263.27	15.51
LDEC000780-RA	40s ribosomal protein s2	-16.91	1578.86	93.35
LDEC008057-RA	alpha-tocopherol transfer	-16.90	66.02	3.91
LDEC006672-RA	probable adenylate kinase isoenzyme	-16.79	121.34	7.23
LDEC007021-RA	long form-like	-16.75	751.82	44.88
LDEC006050-RA	60s ribosomal protein l35a	-16.65	535.13	32.15
LDEC024513-RA	---NA---	-16.64	122.87	7.38
LDEC001030-RA	dihydrolipoamide succinyltransferase component of 2-oxoglutarate dehydrogenase	-16.64	92.94	5.59
LDEC000731-RA	protein takeout-like	-16.60	94.64	5.70
LDEC017156-RA	proclotting enzyme	-16.52	70.98	4.30
LDEC023170-RA	fatty acid synthase-like isoform x3	-16.46	581.32	35.31
LDEC002142-RA	i m not dead yet	-16.43	32.73	1.99
LDEC007893-RA	protein disulfide-isomerase	-16.38	66.53	4.06
LDEC023007-RA	cytochrome p450 6k1	-16.29	643.80	39.53
LDEC005894-RA	sterol o-acyltransferase 1-like	-16.27	73.11	4.49
LDEC002263-RA	hypothetical protein L798_04546	-16.26	41.28	2.54
LDEC009853-RA	elongation of very long chain fatty acids protein aael008004-like	-16.24	121.80	7.50
LDEC005728-RA	c-1-tetrahydrofolate cytoplasmic isoform x2	-16.22	651.32	40.15
LDEC009157-RA	microtubule-associated protein tau-like	-16.19	34.15	2.11
LDEC006014-RA	24-dehydrocholesterol reductase	-16.18	59.40	3.67
LDEC011577-RA	lambda-crystallin homolog	-16.13	182.70	11.33
LDEC018191-RA	sodium-coupled monocarboxylate transporter 1	-16.11	262.37	16.29
LDEC007013-RA	hypothetical protein YQE_02064, partial [Dendroctonus ponderosae]	-16.11	44.67	2.77
LDEC002758-RA	tyrosine-protein phosphatase 10d isoform x1	-16.10	1198.54	74.45
LDEC001118-RA	farnesoic acid o-methyltransferase-like protein	-16.03	688.72	42.97
LDEC005725-RA	long form	-16.02	327.97	20.47
LDEC005914-RA	46 kda fk506-binding nuclear protein	-16.02	149.54	9.34
LDEC014165-RA	PREDICTED: uncharacterized protein LOC661483	-15.99	148.05	9.26
LDEC011582-RA	cuticular protein analogous to peritrophins 3-b precursor	-15.94	143.24	8.98
LDEC006052-RA	muscular protein 20	-15.94	271.48	17.03
LDEC011583-RA	cuticular protein analogous to peritrophins 3-a1 precursor	-15.92	68.41	4.30
LDEC004838-RA	glutathione s-transferase epsilon	-15.90	49.06	3.09
LDEC008468-RA	calumenin	-15.89	381.60	24.02
LDEC001798-RA	rna-binding protein squid isoform x3	-15.87	628.74	39.61
LDEC021171-RA	esterase	-15.85	81.73	5.16
LDEC005085-RA	PREDICTED: uncharacterized protein LOC103314372	-15.79	441.08	27.93
LDEC007592-RA	galectin-4-like isoform x2	-15.79	115.36	7.30

LDEC011117-RA	peptidyl-alpha-hydroxyglycine alpha-amidating lyase 2 isoform x1	-15.76	69.56	4.41
LDEC007998-RA	hormone receptor in 46-like protein	-15.73	49.77	3.16
LDEC012842-RA	lim domain-binding protein 3 isoform x1	-15.70	208.45	13.28
LDEC013409-RA	c-1-tetrahydrofolate cytoplasmic	-15.69	80.90	5.16
LDEC017098-RA	60 kda heat shock mitochondrial-like	-15.68	365.12	23.28
LDEC007593-RA	ribose-phosphate pyrophosphokinase 1 isoform x1	-15.61	78.66	5.04
LDEC018073-RA	troponin i isoform x12	-15.55	81.40	5.23
LDEC018480-RA	40s ribosomal protein s2	-15.47	39.28	2.54
LDEC004939-RA	serine protease	-15.47	170.41	11.02
LDEC007070-RA	serine-arginine protein 55 isoform x7	-15.47	283.41	18.32
LDEC014169-RA	cytochrome p450 partial	-15.46	155.75	10.08
LDEC013553-RA	odorant-binding protein 5	-15.40	252.60	16.41
LDEC008084-RA	40s ribosomal protein s28	-15.39	116.64	7.58
LDEC010981-RA	aspartyl asparaginyl beta-hydroxylase	-15.38	79.28	5.16
LDEC013855-RA	protein henna	-15.37	48.02	3.12
LDEC015353-RA	phosphatidylinositol transfer protein	-15.33	55.71	3.63
LDEC004014-RA	heat shock 70 kda protein cognate 5	-15.33	153.88	10.04
LDEC003829-RA	c-type lectin galactose-binding isoform-like	-15.32	203.51	13.28
LDEC008607-RA	esterase	-15.32	298.61	19.49
LDEC005268-RA	atp-citrate synthase	-15.28	192.14	12.58
LDEC011542-RA	kynurenine alpha-aminoadipate mitochondrial-like	-15.23	150.54	9.88
LDEC010190-RA	rna-binding protein squid isoform x3	-15.13	618.15	40.86
LDEC004826-RA	pyrroline-5-carboxylate reductase 2	-15.10	46.58	3.09
LDEC024338-RA	adenosylhomocysteinase b	-15.09	184.52	12.23
LDEC016278-RA	probable serine threonine-protein kinase nek3 isoform x1	-15.09	59.52	3.95
LDEC007078-RA	hypothetical protein D910_08753	-15.06	360.70	23.94
LDEC003222-RA	scavenger receptor class b member 1	-15.02	104.46	6.95
LDEC004574-RA	acyl- delta desaturase isoform x1	-14.99	32.21	2.15
LDEC005884-RA	60s ribosomal protein l6	-14.91	311.56	20.90
LDEC004253-RA	sodium-coupled monocarboxylate transporter 1	-14.86	101.58	6.84
LDEC024465-RA	af117576_1kazal-type proteinase inhibitor	-14.78	81.98	5.55
LDEC008159-RA	ribonucleoside-diphosphate reductase subunit m2 b	-14.75	84.12	5.70
LDEC005371-RA	circadian clock-controlled protein	-14.71	191.30	13.01
LDEC003963-RA	charged multivesicular body protein 3	-14.68	51.60	3.52
LDEC000762-RA	muscle-specific protein 20-like	-14.61	148.38	10.16
LDEC009006-RA	PREDICTED: uncharacterized protein LOC102674856	-14.60	94.09	6.44
LDEC005923-RA	prostatic acid phosphatase	-14.60	60.44	4.14
LDEC003331-RA	membrane-bound alkaline phosphatase	-14.59	80.90	5.55
LDEC017108-RA	small nuclear ribonucleoprotein sm d3	-14.57	76.82	5.27

LDEC009033-RA	ribosomal protein l10ae	-14.56	1107.12	76.05
LDEC017593-RA	histone	-14.52	159.38	10.98
LDEC002760-RA	protein arginine n-methyltransferase 1 isoform x1	-14.52	196.76	13.55
LDEC010573-RA	39s ribosomal protein mitochondrial	-14.45	58.69	4.06
LDEC004781-RA	integumentary mucin -like isoform x1	-14.44	666.78	46.17
LDEC007118-RA	l-xylulose reductase	-14.41	95.72	6.64
LDEC005651-RA	glycine cleavage system h protein	-14.36	34.22	2.38
LDEC003973-RA	four and a half lim domains protein 2 isoform x5	-14.30	259.12	18.12
LDEC007644-RA	aldehyde mitochondrial	-14.28	842.81	59.02
LDEC010151-RA	PREDICTED: uncharacterized protein LOC656585	-14.28	68.60	4.80
LDEC016749-RA	pupal cuticle protein 20	-14.26	33.98	2.38
LDEC019779-RA	rrna 2 -o-methyltransferase fibrillarin	-14.24	92.88	6.52
LDEC005370-RA	midline fasciclin	-14.23	335.10	23.55
LDEC000760-RA	hypothetical protein TcasGA2_TC013635	-14.21	51.60	3.63
LDEC009102-RA	ribosomal protein l22	-14.21	621.46	43.75
LDEC005567-RA	venom acid phosphatase acph-1-like	-14.20	33.84	2.38
LDEC018087-RA	cysteine dioxygenase type 1-like	-14.14	57.45	4.06
LDEC002382-RA	na+ + atpase alpha-subunit partial	-14.13	105.45	7.46
LDEC014146-RA	tubby-related protein 1	-14.05	110.86	7.89
LDEC007807-RA	acidic mammalian chitinase-like	-14.02	123.73	8.83
LDEC017393-RA	inosine-uridine preferring nucleoside hydrolase-like	-13.99	42.08	3.01
LDEC006988-RA	glyoxylate reductase hydroxypyruvate reductase	-13.97	87.29	6.25
LDEC005975-RA	la protein homolog	-13.92	106.56	7.66
LDEC012841-RA	cytosolic 10-formyltetrahydrofolate dehydrogenase	-13.92	97.31	6.99
LDEC005758-RA	acetyl-coa carboxylase	-13.90	312.29	22.46
LDEC002424-RA	40s ribosomal protein s28	-13.86	57.91	4.18
LDEC000979-RA	lachesin	-13.80	52.83	3.83
LDEC024126-RA	tyrosine 3-monooxygenase	-13.80	68.99	5.00
LDEC006128-RA	60s acidic ribosomal protein p1	-13.77	1550.48	112.57
LDEC014462-RA	lysosomal pro-x carboxypeptidase	-13.66	82.19	6.02
LDEC002231-RA	choline-phosphate cytidylyltransferase a-like isoform x2	-13.62	310.70	22.81
LDEC002143-RA	na+ + atpase alpha-subunit 1	-13.55	32.83	2.42
LDEC018931-RA	agap011225-pa-like protein	-13.55	1579.34	116.56
LDEC014796-RA	upstream activation factor subunit spp27	-13.50	598.35	44.33
LDEC012033-RA	ribosomal protein l7ae	-13.46	1123.33	83.43
LDEC014795-RA	serine arginine repetitive matrix protein 1	-13.46	366.33	27.23
LDEC001692-RA	15-hydroxyprostaglandin dehydrogenase	-13.45	49.38	3.67
LDEC022744-RA	l-lactate dehydrogenase isoform x2	-13.44	714.07	53.12
LDEC008853-RA	voltage-dependent anion channel	-13.43	706.64	52.61

LDEC016298-RA	oligopeptidase a	-13.42	118.42	8.83
LDEC001987-RA	citrate synthase	-13.41	2735.63	203.94
LDEC010761-RA	fggy carbohydrate kinase domain-containing protein	-13.40	63.87	4.77
LDEC002600-RA	seminal fluid partial	-13.38	100.37	7.50
LDEC013653-RA	farnesyl diphosphate synthase	-13.38	134.87	10.08
LDEC017096-RA	elongation of very long chain fatty acids protein 7	-13.37	36.04	2.70
LDEC009388-RA	chromo domain-containing protein cec-1	-13.26	97.86	7.38
LDEC004602-RA	t-complex protein 1 subunit delta	-13.25	215.31	16.25
LDEC006099-RA	isoform o	-13.25	96.77	7.30
LDEC006951-RA	chymotrypsin-c-like isoform x1	-13.25	60.02	4.53
LDEC000085-RA	protein l 37cc	-13.24	306.08	23.12
LDEC002683-RA	von willebrand factor d and egf domain-containing protein	-13.24	442.54	33.44
LDEC000238-RA	na+ + atpase alpha-subunit 1	-13.20	159.34	12.07
LDEC018983-RA	la-related protein 6	-13.17	659.82	50.11
LDEC008157-RA	isoform b	-13.16	63.22	4.80
LDEC011895-RA	serine protease gd-like isoform x2	-13.10	61.93	4.73
LDEC006047-RA	juvenile hormone binding protein partial	-13.10	95.70	7.30
LDEC018086-RA	lactase-phlorizin hydrolase	-13.08	49.55	3.79
LDEC012533-RA	fatty acyl- reductase cg5065-like	-13.06	185.17	14.18
LDEC020340-RA	monocarboxylate transporter	-13.00	39.09	3.01
LDEC000772-RA	protein i m not dead yet	-12.98	73.03	5.62
LDEC012448-RA	hypothetical protein D910_04523	-12.98	53.73	4.14
LDEC003614-RA	mesencephalic astrocyte-derived neurotrophic factor homolog	-12.95	311.09	24.02
LDEC004958-RA	prostatic acid phosphatase	-12.91	262.20	20.31
LDEC001744-RA	complement component 1 q subcomponent-binding mitochondrial	-12.89	151.04	11.72
LDEC017981-RA	agap011197-pa-like protein	-12.87	2080.94	161.63
LDEC010734-RA	PREDICTED: uncharacterized protein LOC103314279	-12.85	43.17	3.36
LDEC004144-RA	microtubule-associated protein futsch-like isoform x12	-12.83	36.58	2.85
LDEC017377-RA	PREDICTED: uncharacterized protein LOC103314372	-12.76	362.79	28.44
LDEC007619-RA	transcription factor btf3 homolog 4	-12.74	470.81	36.95
LDEC010207-RA	---NA---	-12.74	435.26	34.18
LDEC022543-RA	z9 acyl- desaturase b	-12.72	673.72	52.97
LDEC023381-RA	aspartyl asparaginyl beta-hydroxylase isoform x1	-12.66	173.06	13.67
LDEC006900-RA	acetyl- carboxylase isoform x2	-12.64	95.31	7.54
LDEC006045-RA	atp synthase b mitochondrial	-12.63	291.98	23.12
LDEC008467-RA	---NA---	-12.62	787.06	62.38
LDEC005753-RA	hypothetical protein TcasGA2_TC003147	-12.61	168.00	13.32
LDEC019012-RA	isoform a	-12.57	212.57	16.91
LDEC005654-RA	t-complex protein 1 subunit beta	-12.55	217.59	17.34

LDEC003616-RA	protein disulfide-isomerase a5	-12.54	36.73	2.93
LDEC008684-RA	cystine knot toxin	-12.52	69.94	5.59
LDEC016361-RA	serine--trna cytoplasmic	-12.51	171.51	13.71
LDEC016655-RA	atp-dependent rna helicase wm6	-12.45	238.38	19.14
LDEC005763-RA	reverse partial	-12.42	121.24	9.77
LDEC004599-RA	venom carboxylesterase-6	-12.40	94.41	7.62
LDEC003783-RA	isoform c	-12.34	338.24	27.42
LDEC009165-RA	hypothetical protein D910_01838, partial	-12.33	115.59	9.37
LDEC013552-RA	splicing factor 3b subunit 4	-12.33	65.48	5.31
LDEC018973-RA	troponin c-like	-12.27	56.09	4.57
LDEC021641-RA	PREDICTED: fumarylacetoacetase	-12.27	63.75	5.20
LDEC015684-RA	60s ribosomal protein l23	-12.25	726.03	59.25
LDEC008099-RA	farnesyl diphosphate synthase	-12.23	122.79	10.04
LDEC023006-RA	polypyrimidine tract-binding protein 1 isoform x3	-12.22	105.97	8.67
LDEC000563-RA	cell wall protein dan4	-12.21	82.49	6.76
LDEC015117-RA	tubulin alpha-3	-12.21	96.79	7.93
LDEC009802-RA	ptpla domain protein	-12.10	87.44	7.23
LDEC013118-RA	ruvb-like 1-like	-12.10	83.64	6.91
LDEC015280-RA	ribonucleoside-diphosphate reductase large subunit-like	-12.09	58.54	4.84
LDEC004241-RA	carrier protein 1-like	-12.03	548.93	45.62
LDEC006895-RA	isocitrate dehydrogenase	-12.02	42.25	3.52
LDEC004679-RA	ornithine mitochondrial	-12.01	56.28	4.69
LDEC005965-RA	PREDICTED: uncharacterized protein LOC103314280	-12.00	81.12	6.76
LDEC010916-RA	leukocyte elastase inhibitor	-11.99	35.60	2.97
LDEC013717-RA	peptidyl-prolyl cis-trans isomerase	-11.93	1680.50	140.81
LDEC021581-RA	ester hydrolase c11orf54 homolog	-11.93	155.64	13.05
LDEC013633-RA	ribose-phosphate pyrophosphokinase 1 isoform x2	-11.91	186.11	15.62
LDEC017303-RA	ribosomal protein l4	-11.90	2043.19	171.63
LDEC006352-RA	d-3-phosphoglycerate dehydrogenase	-11.90	211.54	17.77
LDEC020371-RA	glyoxalase domain-containing protein 4-like	-11.88	167.52	14.10
LDEC015091-RA	heat shock 70 kda protein cognate 5	-11.87	419.97	35.39
LDEC003945-RA	40s ribosomal protein s20	-11.86	565.89	47.73
LDEC011341-RA	aconitate mitochondrial-like isoform x2	-11.83	124.35	10.51
LDEC008598-RA	transformer-2 protein homolog beta isoform x2	-11.82	132.05	11.17
LDEC015417-RA	zinc carboxypeptidase	-11.82	41.08	3.48
LDEC021795-RA	RT01145p	-11.81	95.01	8.05
LDEC013915-RA	scavenger receptor protein	-11.76	120.82	10.27
LDEC003875-RA	nucleoplasmin isoform 2	-11.76	502.86	42.77
LDEC003053-RA	nuclear autoantigenic sperm protein	-11.75	51.87	4.41
LDEC017095-RA	60s ribosomal protein l6	-11.75	1213.05	103.24

LDEC004865-RA	multidrug resistance-associated protein	-11.74	72.47	6.17
LDEC004990-RA	t-complex protein 1 subunit eta	-11.73	100.81	8.59
LDEC001346-RA	hypothetical protein D910_00165	-11.70	376.28	32.15
LDEC001762-RA	imaginal disc growth factor 4 precursor	-11.70	671.13	57.34
LDEC016510-RA	pathogenesis-related protein 5	-11.65	219.81	18.87
LDEC008691-RA	superoxide dismutase	-11.63	208.97	17.97
LDEC007635-RA	probable multidrug resistance-associated protein lethal 03659 isoform x1	-11.62	78.99	6.80
LDEC006048-RA	probable hydroxyacid-oxoacid mitochondrial	-11.62	41.30	3.55
LDEC005882-RA	shematrin-like protein 2 isoform x2	-11.52	84.58	7.34
LDEC018192-RA	set and mynd domain-containing protein 4-like	-11.49	69.10	6.02
LDEC012375-RA	mki67 fha domain-interacting nucleolar phospho	-11.47	42.58	3.71
LDEC013730-RA	zinc finger protein	-11.47	143.78	12.54
LDEC010962-RA	sortilin-related receptor-like	-11.46	123.50	10.78
LDEC005653-RA	small nuclear ribonucleoprotein-associated protein b	-11.43	79.01	6.91
LDEC012088-RA	protein ssxt isoform x1	-11.42	34.34	3.01
LDEC017157-RA	ig-like and fibronectin type-iii domain-containing protein	-11.41	40.11	3.52
LDEC005871-RA	titin-like isoform x5	-11.40	34.30	3.01
LDEC009538-RA	regucalcin-like isoform x1	-11.40	309.03	27.11
LDEC004412-RA	organic cation transporter protein	-11.36	57.70	5.08
LDEC010949-RA	enhancer of split mgamma protein	-11.34	35.87	3.16
LDEC000193-RA	beta-amyloid-like partial	-11.32	92.44	8.16
LDEC012089-RA	39s ribosomal protein mitochondrial	-11.29	37.92	3.36
LDEC014122-RA	replication protein a 70 kda dna-binding subunit	-11.29	65.25	5.78
LDEC005652-RA	spermidine synthase	-11.23	34.67	3.09
LDEC003294-RA	cell wall protein dan4	-11.17	92.08	8.24
LDEC017757-RA	adipocyte plasma membrane-associated	-11.17	57.16	5.12
LDEC022336-RA	t-complex protein 1 subunit alpha	-11.14	146.21	13.12
LDEC009004-RA	dnaj homolog subfamily c member 22	-11.09	76.25	6.87
LDEC006133-RA	polyadenylate-binding protein 2-b isoform x4	-11.09	50.67	4.57
LDEC013729-RA	proliferating cell nuclear antigen	-11.08	70.14	6.33
LDEC016748-RA	venom acid phosphatase acph-1-like	-11.08	51.51	4.65
LDEC000795-RA	glucose dehydrogenase	-11.08	234.47	21.17
LDEC014197-RA	lipoyltransferase mitochondrial	-11.07	236.08	21.33
LDEC003311-RA	acyl-protein thioesterase 1	-11.06	366.92	33.16
LDEC018262-RA	mitochondrial amidoxime reducing component 2	-11.05	90.68	8.20
LDEC000077-RA	fatty acyl- reductase 1	-11.05	47.06	4.26
LDEC003254-RA	ribosomal protein l13	-11.03	1171.83	106.28
LDEC017373-RA	myc box-dependent-interacting protein 1 isoform x2	-10.99	139.48	12.69
LDEC019738-RA	slit homolog 3	-10.98	35.18	3.20

LDEC010948-RA	procollagen- -oxoglutarate 5-dioxygenase 3	-10.96	163.61	14.92
LDEC004210-RA	type i cytoskeletal 9 isoform x2	-10.94	80.35	7.34
LDEC024630-RA	slit homolog 3	-10.93	52.08	4.77
LDEC004242-RA	atp-dependent rna helicase isoform a	-10.90	39.19	3.59
LDEC015377-RA	gamma-interferon-inducible lysosomal thiol reductase-like	-10.90	75.33	6.91
LDEC005749-RA	probable 28s rrna (cytosine-c)-methyltransferase	-10.87	53.06	4.88
LDEC006360-RA	atp-dependent rna helicase p62	-10.86	554.58	51.05
LDEC002711-RA	sphingomyelin phosphodiesterase isoform x2	-10.85	44.09	4.06
LDEC006314-RA	procollagen- -oxoglutarate 5-dioxygenase 3-like	-10.85	79.66	7.34
LDEC014917-RA	filamin a-interacting protein 1-like	-10.83	61.36	5.66
LDEC002557-RA	guanine nucleotide-binding 3 homolog	-10.82	60.04	5.55
LDEC006878-RA	luciferin-regenerating partial	-10.80	118.08	10.94
LDEC000756-RA	hypothetical protein YQE_01777, partial	-10.79	390.34	36.17
LDEC010270-RA	neurofilament triplet m	-10.76	250.51	23.28
LDEC000775-RA	heterogeneous nuclear ribonucleoprotein k isoform x1	-10.74	200.94	18.71
LDEC018920-RA	ka261_mesma ame: full=potassium channel blocker alpha-ktx ame: full=neurotoxin 86 flags: precursor	-10.72	338.30	31.56
LDEC009783-RA	piopio-like protein	-10.71	85.79	8.01
LDEC010164-RA	junctophilin-1 isoform x2	-10.68	49.25	4.61
LDEC009709-RA	cyc_sarpe ame: full=cytochrome c	-10.64	467.18	43.90
LDEC009502-RA	dopamine n-acetyltransferase-like isoform x4	-10.60	65.44	6.17
LDEC013728-RA	plasminogen activator inhibitor 1 rna-binding partial	-10.59	224.24	21.17
LDEC016969-RA	serine proteinase	-10.59	139.75	13.20
LDEC018534-RA	double-stranded rna-specific editase 1 isoform x2	-10.58	281.11	26.56
LDEC010720-RA	60s ribosomal protein l18-like	-10.58	72.74	6.87
LDEC003986-RA	hypothetical antimicrobial peptide	-10.55	267.76	25.39
LDEC002556-RA	b1 protein	-10.53	203.66	19.33
LDEC000058-RA	gelsolin-related protein of 125 kda isoform x2	-10.53	81.86	7.77
LDEC016473-RA	60s ribosomal protein l21	-10.50	167.33	15.94
LDEC014671-RA	homogentisate -dioxygenase	-10.50	35.26	3.36
LDEC019172-RA	gly-rich protein	-10.48	81.50	7.77
LDEC003113-RA	methyltransferase-like protein 23	-10.44	272.05	26.05
LDEC020469-RA	peptide methionine sulfoxide reductase	-10.44	132.95	12.73
LDEC015347-RA	polyadenylate-binding protein-interacting protein 1-like isoform 2	-10.43	78.60	7.54
LDEC022012-RA	neuroendocrine protein 7b2	-10.39	43.44	4.18
LDEC008530-RA	tho complex subunit 4	-10.37	149.93	14.45
LDEC006894-RA	phosphoglycerate kinase	-10.37	132.47	12.77
LDEC000908-RA	ubiquitin-like protein partial	-10.37	1553.84	149.84
LDEC002501-RA	ap-1 complex subunit beta-1 isoform x1	-10.32	68.91	6.68

LDEC012851-RA	cytochrome p450-like protein	-10.32	138.22	13.40
LDEC000858-RA	long form-like	-10.30	307.89	29.88
LDEC001225-RA	eukaryotic translation initiation factor 3 subunit j	-10.29	187.33	18.20
LDEC002529-RA	growth arrest and dna damage-inducible proteins-interacting protein 1-like	-10.29	61.47	5.98
LDEC012345-RA	protease m1 zinc metalloprotease	-10.28	102.39	9.96
LDEC020565-RA	isoform b	-10.25	268.76	26.21
LDEC006842-RA	phosphogluconate mutase	-10.25	128.50	12.54
LDEC021000-RA	major facilitator superfamily domain-containing protein 6	-10.23	204.27	19.96
LDEC019636-RA	PREDICTED: uncharacterized protein LOC655454	-10.22	34.74	3.40
LDEC002964-RA	esterase b1-like	-10.20	84.05	8.24
LDEC020810-RA	caprin homolog	-10.18	125.67	12.34
LDEC019996-RA	apolipoprotein d-like	-10.17	34.97	3.44
LDEC016525-RA	proteoglycan 4	-10.17	80.25	7.89
LDEC014330-RA	rhythmically expressed gene 5 protein	-10.17	72.68	7.15
LDEC008701-RA	protein lsm12 homolog	-10.17	41.70	4.10
LDEC014467-RA	inter-alpha-trypsin inhibitor heavy chain h4-like isoform x1	-10.15	66.59	6.56
LDEC009415-RA	eukaryotic translation initiation factor 2 subunit 1	-10.14	101.79	10.04
LDEC005049-RA	60s ribosomal protein l3	-10.14	1617.92	159.56
LDEC014889-RA	mitochondrial import receptor subunit tom22 homolog isoform x2	-10.14	83.55	8.24
LDEC020299-RA	mitochondrial import inner membrane translocase subunit tim13	-10.13	36.01	3.55
LDEC010015-RA	histone h2a-like	-10.10	105.34	10.43
LDEC021665-RA	polyadenylate-binding protein 1-like isoform 3	-10.10	56.40	5.59
LDEC003299-RA	39s ribosomal protein mitochondrial	-10.08	46.83	4.65
LDEC014126-RA	60s ribosomal protein l31	-10.07	691.04	68.63
LDEC008220-RA	juvenile hormone acid methyltransferase	-10.02	56.78	5.66
LDEC015597-RA	piggybac transposable element-derived protein 4-like	-10.01	124.75	12.46
LDEC019995-RA	chitooligosaccharidolytic beta-n-acetylglucosaminidase	-10.00	161.67	16.17
LDEC010233-RA	ethanolamine-phosphate cytidylyltransferase	-9.99	110.40	11.05
LDEC023632-RA	PREDICTED: uncharacterized protein LOC103314372	-9.90	256.76	25.94
LDEC003545-RA	s-formylglutathione hydrolase	-9.89	100.05	10.12
LDEC015453-RA	atp-binding cassette sub-family d member 3	-9.85	194.35	19.73
LDEC015975-RA	facilitated trehalose transporter tret1	-9.85	43.86	4.45
LDEC019788-RA	fk506-binding protein 2 isoform x1	-9.82	95.91	9.77
LDEC022004-RA	serine protease	-9.80	78.82	8.05
LDEC009707-RA	serine protease persephone-like	-9.79	76.48	7.81
LDEC005976-RA	nadh dehydrogenase	-9.78	123.37	12.62
LDEC017506-RA	peptidyl-prolyl cis-trans isomerase fkbp14- partial	-9.76	358.86	36.76

LDEC001758-RA	serine proteinase partial	-9.74	36.91	3.79
LDEC011997-RA	ribosomal protein l27e	-9.73	843.90	86.75
LDEC010111-RA	basement membrane-specific heparan sulfate proteoglycan core protein isoform x14	-9.72	438.90	45.15
LDEC023974-RA	mitochondrial ribosomal protein l37	-9.71	67.16	6.91
LDEC014331-RA	phosphoglyceromutase	-9.71	271.69	27.97
LDEC014088-RA	probable histone-binding protein caf1	-9.71	121.78	12.54
LDEC015119-RA	lipase maturation factor 2	-9.71	186.64	19.22
LDEC006705-RA	protein brambleberry-like	-9.69	39.76	4.10
LDEC006115-RA	pdz and lim domain protein zasp	-9.69	144.20	14.88
LDEC019483-RA	prohibitin-2 isoform x2	-9.68	201.99	20.86
LDEC015475-RA	eukaryotic translation initiation factor 2 gamma	-9.67	98.63	10.19
LDEC018896-RA	probable serine hydrolase	-9.67	57.05	5.90
LDEC015672-RA	rac gtpase-activating protein 1-like	-9.67	40.78	4.22
LDEC018747-RA	atp-citrate synthase	-9.63	165.53	17.19
LDEC006619-RA	branched-chain-amino-acid cytosolic	-9.62	69.91	7.27
LDEC013069-RA	ribosomal protein l12e	-9.61	451.53	46.99
LDEC001424-RA	proton-coupled amino acid transporter 4	-9.60	140.67	14.65
LDEC015457-RA	calnexin	-9.58	368.65	38.47
LDEC011237-RA	inter-alpha-trypsin inhibitor heavy chain h4-like isoform x1	-9.58	385.67	40.27
LDEC013203-RA	isocitrate dehydrogenase	-9.56	98.96	10.35
LDEC007608-RA	isoform p	-9.54	466.72	48.90
LDEC008098-RA	acetyl coenzyme a isoform b	-9.54	55.17	5.78
LDEC006909-RA	ribosomal protein l15e	-9.53	735.84	77.18
LDEC009803-RA	alpha-n-acetylgalactosaminidase-like isoform x1	-9.53	101.26	10.62
LDEC020611-RA	cytochrome c oxidase polypeptide iv	-9.50	771.18	81.17
LDEC013281-RA	ribosomal protein s8e	-9.49	1401.61	147.65
LDEC008132-RA	alpha- partial	-9.45	61.30	6.48
LDEC012445-RA	serine protease s1a- partial	-9.45	49.46	5.23
LDEC021271-RA	icarapin-like partial	-9.44	71.13	7.54
LDEC021989-RA	seryl-trna mitochondrial	-9.43	106.06	11.25
LDEC003531-RA	juvenile hormone esterase binding protein	-9.42	81.31	8.63
LDEC006944-RA	93 kda serpin	-9.41	210.94	22.42
LDEC005273-RA	molybdenum cofactor biosynthesis protein 1	-9.40	55.05	5.86
LDEC018298-RA	mitochondrial nadh-ubiquinone oxidoreductase 9 kda subunit-like protein	-9.39	43.27	4.61
LDEC003355-RA	muscle protein 20-like protein	-9.39	1660.30	176.87
LDEC012911-RA	40s ribosomal protein sa	-9.39	119.15	12.69
LDEC003475-RA	probable nucleoporin nup58 isoform x1	-9.35	51.11	5.47
LDEC021397-RA	peptidyl-prolyl cis-trans isomerase 5	-9.31	331.24	35.58

LDEC009204-RA	quaking related	-9.29	209.70	22.58
LDEC006675-RA	transketolase-like protein 2 isoform x1	-9.27	537.78	58.04
LDEC007953-RA	60s ribosomal protein l37a	-9.26	443.84	47.93
LDEC002153-RA	nadh dehydrogenase	-9.26	115.32	12.46
LDEC007394-RA	40s ribosomal protein s12	-9.23	544.33	58.94
LDEC000771-RA	transketolase-like protein 2 isoform x1	-9.23	1220.87	132.34
LDEC013444-RA	dehydrogenase reductase sdr family protein 7-like	-9.21	45.32	4.92
LDEC009497-RA	probable phenylalanine--trna ligase alpha subunit	-9.21	79.47	8.63
LDEC003007-RA	atp synthase subunit mitochondrial	-9.20	460.94	50.11
LDEC006238-RA	probable isocitrate dehydrogenase	-9.19	225.45	24.53
LDEC001493-RA	lipid storage droplets surface-binding protein 1-like isoform x1	-9.18	220.81	24.06
LDEC001515-RA	glucose dehydrogenase	-9.17	141.09	15.39
LDEC006160-RA	protein transport protein sec61 subunit gamma	-9.14	72.43	7.93
LDEC015525-RA	probable enoyl- mitochondrial-like	-9.13	957.75	104.88
LDEC004391-RA	hairy cell leukemia	-9.10	380.34	41.79
LDEC015177-RA	pdz and lim domain protein zasp-like	-9.07	42.89	4.73
LDEC006858-RA	na+ + atpase alpha-subunit partial	-9.07	93.15	10.27
LDEC019168-RA	glutamate mitochondrial	-9.06	408.72	45.11
LDEC004744-RA	aquaporin aqpcic	-9.06	88.11	9.73
LDEC016681-RA	dnaj homolog subfamily c member 10-like	-9.05	60.48	6.68
LDEC003448-RA	heat shock protein 90	-9.03	104.80	11.60
LDEC020000-RA	spondin-1	-9.03	196.15	21.72
LDEC008349-RA	serine protease easter	-9.02	94.11	10.43
LDEC011014-RA	protein-tyrosine sulfotransferase	-8.99	36.89	4.10
LDEC013339-RA	40s ribosomal protein sa	-8.99	2972.75	330.65
LDEC021974-RA	nhp2-like protein 1	-8.99	43.17	4.80
LDEC021797-RA	protein mago nashi homolog	-8.99	42.12	4.69
LDEC003855-RA	simila to cg6426	-8.99	84.58	9.41
LDEC016118-RA	nadh dehydrogenase	-8.98	133.98	14.92
LDEC009177-RA	hypothetical protein YQE_01873, partial	-8.97	43.12	4.80
LDEC016638-RA	40s ribosomal protein s10	-8.97	1092.96	121.79
LDEC014837-RA	arginine serine-rich splicing	-8.96	636.35	71.05
LDEC001590-RA	four and a half lim domains	-8.95	525.78	58.75
LDEC005270-RA	12 kda fk506-binding protein	-8.95	270.82	30.27
LDEC012447-RA	prostaglandin e synthase 3	-8.93	190.19	21.29
LDEC004240-RA	28 kda heat- and acid-stable phosphoprotein	-8.92	127.18	14.26
LDEC003112-RA	bifunctional purine biosynthesis protein purh	-8.91	241.10	27.07
LDEC006678-RA	glyoxylate reductase hydroxypyruvate reductase-like	-8.90	214.41	24.10
LDEC018409-RA	heat shock protein 60	-8.90	57.68	6.48

LDEC001690-RA	cystathionine gamma-lyase	-8.89	221.29	24.88
LDEC013113-RA	glycerol-3-phosphate dehydrogenase	-8.88	417.13	46.99
LDEC003946-RA	ribosomal protein l19e	-8.87	1126.74	127.06
LDEC000558-RA	dehydrogenase reductase sdr family member 11-like	-8.86	37.39	4.22
LDEC014461-RA	ribosomal protein l36e	-8.81	397.72	45.15
LDEC000668-RA	enolase	-8.81	913.27	103.71
LDEC017598-RA	nhp2-like protein 1	-8.80	37.48	4.26
LDEC021947-RA	40s ribosomal protein s5	-8.78	1296.88	147.69
LDEC015637-RA	ribosome biogenesis regulatory protein homolog	-8.78	78.20	8.91
LDEC012360-RA	mitochondrial nadh dehydrogenase fe-s protein	-8.77	71.59	8.16
LDEC008142-RA	ribosomal protein s23	-8.73	966.33	110.66
LDEC007343-RA	acyl-coa thioesterase	-8.70	62.51	7.19
LDEC014792-RA	ribosomal protein l14	-8.68	913.86	105.31
LDEC005909-RA	atp synthase subunit mitochondrial	-8.67	218.84	25.23
LDEC014666-RA	chitin synthase	-8.67	88.70	10.23
LDEC007477-RA	shc sh2 domain-binding protein 1	-8.64	68.14	7.89
LDEC015759-RA	glutamic acid-rich isoform x3	-8.64	86.02	9.96
LDEC020880-RA	scavenger receptor class b member 1 isoform x2	-8.61	83.03	9.65
LDEC012306-RA	39s ribosomal protein mitochondrial	-8.60	76.55	8.91
LDEC013772-RA	fatty acid synthase	-8.59	360.51	41.95
LDEC008040-RA	succinyl- ligase	-8.57	228.38	26.64
LDEC000802-RA	tp53-regulating kinase	-8.57	122.53	14.30
LDEC019869-RA	glutathione peroxidase	-8.57	40.82	4.77
LDEC007240-RA	protein n-terminal asparagine amidohydrolase	-8.54	61.01	7.15
LDEC014488-RA	vasa rna helicase	-8.51	54.19	6.37
LDEC008365-RA	importin-7 isoform x1	-8.49	223.84	26.37
LDEC008350-RA	dihydrolipoamide dehydrogenase e3 subunit	-8.49	409.47	48.24
LDEC004861-RA	sparc	-8.48	628.70	74.10
LDEC017682-RA	uncharacterized threonine-rich gpi-anchored glyco	-8.47	272.42	32.15
LDEC023468-RA	histidine decarboxylase isoform x1	-8.47	37.39	4.41
LDEC024177-RA	fumarate mitochondrial	-8.46	255.74	30.23
LDEC003786-RA	fatty acyl- reductase cg5065-like	-8.42	41.10	4.88
LDEC007952-RA	tetraspanin 42ee	-8.39	95.31	11.37
LDEC009001-RA	elongation factor tu	-8.39	254.50	30.35
LDEC002268-RA	probable aconitate mitochondrial	-8.38	889.51	106.17
LDEC019030-RA	mitochondrial ribosomal protein l1	-8.37	69.64	8.32
LDEC014105-RA	udp-glucuronosyltransferase 2b16	-8.36	90.50	10.82
LDEC004397-RA	transmembrane protein 161b	-8.36	54.54	6.52
LDEC003397-RA	rna-binding protein lark isoform x4	-8.36	325.82	38.98
LDEC001622-RA	glutathione s-transferase epsilon	-8.35	64.25	7.69

LDEC015477-RA	chymotrypsin inhibitor-like	-8.35	294.18	35.23
LDEC001968-RA	ribosomal protein s6	-8.35	1526.99	182.96
LDEC016599-RA	39s ribosomal protein mitochondrial	-8.34	49.84	5.98
LDEC010893-RA	multiple inositol polyphosphate phosphatase 1	-8.32	71.84	8.63
LDEC018238-RA	mitochondrial import receptor subunit tom40	-8.32	180.95	21.76
LDEC003725-RA	apolipoprotein o-like	-8.31	50.99	6.13
LDEC013831-RA	fatty acid synthase	-8.31	199.37	23.98
LDEC018261-RA	adenine nucleotide translocase	-8.31	5704.56	686.80
LDEC022867-RA	lethal isoform a	-8.30	91.79	11.05
LDEC003148-RA	alcohol dehydrogenase class-3-like	-8.30	475.37	57.26
LDEC002959-RA	14-3-3 epsilon protein	-8.29	171.28	20.66
LDEC003831-RA	fact complex subunit ssrp1	-8.29	121.07	14.61
LDEC007868-RA	rpii140-upstream gene protein	-8.28	37.83	4.57
LDEC002197-RA	s-adenosylmethionine synthetase	-8.27	2115.03	255.65
LDEC003866-RA	asparagine--trna cytoplasmic	-8.27	200.00	24.18
LDEC020806-RA	coiled-coil domain-containing protein 47	-8.27	109.51	13.24
LDEC006511-RA	glyoxalase domain-containing protein 4-like	-8.26	96.10	11.64
LDEC018417-RA	t-complex protein 1 subunit gamma	-8.25	275.33	33.36
LDEC012939-RA	ribosomal protein l8e	-8.24	472.89	57.42
LDEC010362-RA	nadh:ubiquinone dehydrogenase	-8.22	87.61	10.66
LDEC014750-RA	juvenile hormone-inducible protein	-8.21	258.98	31.56
LDEC007396-RA	basement membrane-specific heparan sulfate proteoglycan core protein isoform x15	-8.20	54.46	6.64
LDEC004900-RA	probable aconitate mitochondrial	-8.19	171.72	20.98
LDEC009654-RA	phosphoribosylformylglycinamidine synthase-like	-8.19	133.33	16.29
LDEC012084-RA	probable nucleolar gtp-binding protein 1	-8.18	103.86	12.69
LDEC010984-RA	ribosomal protein l7e	-8.18	1251.18	152.96
LDEC014816-RA	nuclear pore complex protein ddb_g0274915 homolog	-8.18	88.45	10.82
LDEC001282-RA	40s ribosomal protein s16	-8.17	988.65	120.97
LDEC014808-RA	g-protein coupled receptor 158	-8.16	68.54	8.40
LDEC023280-RA	neurochondrin homolog	-8.15	178.92	21.95
LDEC000565-RA	stromal cell-derived factor 2	-8.14	118.62	14.57
LDEC007844-RA	galectin-4-like isoform x2	-8.14	49.57	6.09
LDEC016512-RA	chitinase 7	-8.13	61.61	7.58
LDEC007894-RA	succinyl- synthetase small	-8.12	299.86	36.91
LDEC004009-RA	serine arginine-rich splicing factor 7-like isoform x1	-8.10	85.43	10.55
LDEC013635-RA	acetyl-coenzyme a synthetase	-8.08	101.37	12.54
LDEC007822-RA	basement membrane-specific heparan sulfate proteoglycan core protein isoform x6	-8.07	432.04	53.55
LDEC024578-RA	40s ribosomal protein s13	-8.06	649.78	80.58

LDEC019714-RA	rna-binding protein 8a	-8.06	50.38	6.25
LDEC014876-RA	angio-associated migratory cell protein	-8.05	44.02	5.47
LDEC013846-RA	tubulin beta-1 chain	-8.05	4276.86	531.50
LDEC002254-RA	eukaryotic translation initiation factor 2a	-8.04	143.89	17.89
LDEC005676-RA	lon protease mitochondrial isoform x1	-8.01	44.44	5.55
LDEC007424-RA	glucose dehydrogenase	-8.01	63.81	7.97
LDEC001143-RA	translocation protein sec63 homolog	-8.01	74.75	9.34
LDEC000052-RA	odorant binding protein 8	-8.00	239.61	29.96
LDEC014400-RA	28s ribosomal protein mitochondrial	-7.99	90.49	11.33
LDEC013377-RA	n-acetylneuraminate lyase-like	-7.99	216.46	27.11
LDEC006785-RA	protein d2-like isoform x2	-7.97	296.20	37.15
LDEC015741-RA	ubiquitin carboxyl-terminal hydrolase isozyme l5	-7.97	50.74	6.37
LDEC022926-RA	presequence mitochondrial	-7.97	199.19	25.00
LDEC004322-RA	cytochrome c oxidase subunit 6c	-7.96	152.40	19.14
LDEC018966-RA	heat shock protein 90	-7.95	2609.00	328.07
LDEC005034-RA	gtp-binding nuclear protein ran	-7.95	283.11	35.62
LDEC016755-RA	dehydrogenase reductase sdr family member 11-like	-7.95	61.15	7.69
LDEC015145-RA	40s ribosomal protein s14	-7.93	708.98	89.41
LDEC012234-RA	alpha-n-acetylgalactosaminidase	-7.92	73.58	9.30
LDEC001732-RA	zinc finger rna-binding protein	-7.91	322.49	40.78
LDEC004031-RA	prefoldin subunit 2	-7.90	69.46	8.79
LDEC018033-RA	phosphatidylserine decarboxylase	-7.90	86.38	10.94
LDEC005452-RA	dna replication licensing factor mcm6	-7.89	65.06	8.24
LDEC014257-RA	periodic tryptophan protein 1 homolog	-7.89	60.42	7.66
LDEC002286-RA	atp synthase	-7.88	1027.06	130.35
LDEC004093-RA	nadh dehydrogenase	-7.88	78.16	9.92
LDEC018453-RA	mucin- partial	-7.87	473.03	60.11
LDEC023561-RA	60s ribosomal protein l11	-7.87	1010.67	128.47
LDEC010704-RA	monocarboxylate transporter 12	-7.85	58.29	7.42
LDEC006942-RA	ribosomal protein L39 [Danaus plexippus]	-7.85	191.59	24.41
LDEC019737-RA	hypothetical protein TcasGA2_TC015315	-7.81	144.89	18.55
LDEC004943-RA	atp synthase	-7.80	314.23	40.27
LDEC017202-RA	proliferation-associated protein 2g4	-7.80	295.91	37.93
LDEC007877-RA	cytochrome heme mitochondrial	-7.79	282.38	36.25
LDEC011559-RA	60s ribosomal protein l34	-7.79	203.85	26.17
LDEC001608-RA	protein 5nuc-like	-7.79	48.06	6.17
LDEC005903-RA	very long-chain-fatty-acid-- ligase bubblegum isoform x1	-7.79	503.34	64.65
LDEC014489-RA	39s ribosomal protein mitochondrial	-7.78	44.36	5.70
LDEC003266-RA	abc transporter g family member 23-like isoform x2	-7.78	231.73	29.80
LDEC003532-RA	protein disulfide-isomerase	-7.76	546.55	70.43

LDEC005388-RA	tetratricopeptide repeat protein 26-like isoform x2	-7.75	115.32	14.88
LDEC000562-RA	39s ribosomal protein mitochondrial	-7.73	48.29	6.25
LDEC008133-RA	agap010464-pa-like protein	-7.71	148.26	19.22
LDEC006681-RA	tubulin beta-1 chain	-7.70	305.43	39.65
LDEC008683-RA	interleukin enhancer-binding factor 2 homolog	-7.69	170.72	22.19
LDEC003889-RA	cleavage and polyadenylation specificity factor subunit 5	-7.69	46.55	6.05
LDEC009269-RA	apoptosis inhibitor 5	-7.69	75.35	9.80
LDEC005491-RA	methylcrotonoyl- carboxylase beta mitochondrial	-7.68	117.03	15.23
LDEC002701-RA	myb-like protein x	-7.66	42.22	5.51
LDEC000699-RA	titin-like isoform x5	-7.65	87.84	11.48
LDEC013647-RA	PREDICTED: uncharacterized protein LOC662064 isoform X3	-7.65	64.23	8.40
LDEC018600-RA	beta-parvin isoform x1	-7.62	92.57	12.15
LDEC007616-RA	elongation factor 1 gamma	-7.62	717.01	94.10
LDEC020107-RA	synaptic vesicle glycoprotein 2c	-7.61	48.46	6.37
LDEC010451-RA	ribosomal protein s26	-7.61	564.84	74.22
LDEC014600-RA	protein 5nuc-like	-7.61	76.06	10.00
LDEC002561-RA	ribosomal protein l27ae	-7.60	101.87	13.40
LDEC002190-RA	60s ribosomal protein l18	-7.60	995.57	130.97
LDEC018333-RA	60s ribosomal protein l21	-7.59	463.01	61.01
LDEC014599-RA	apolipoprotein d	-7.59	87.99	11.60
LDEC008668-RA	thioredoxin-dependent peroxide mitochondrial	-7.57	166.20	21.95
LDEC024633-RA	acyl-coa thioesterase	-7.56	82.94	10.98
LDEC015497-RA	mitochondrial inner membrane protein isoform x1	-7.55	239.30	31.68
LDEC004464-RA	chromobox protein homolog 5-like	-7.54	118.44	15.70
LDEC003634-RA	60s ribosomal protein l44	-7.53	599.71	79.61
LDEC004822-RA	ran gtpase-activating protein 1	-7.52	58.16	7.73
LDEC024471-RA	signal recognition particle 19 kda protein	-7.51	47.54	6.33
LDEC001796-RA	glutamine synthetase 2 cytoplasmic isoform x2	-7.51	82.72	11.02
LDEC013929-RA	laminin subunit gamma-1 isoform x2	-7.49	84.60	11.29
LDEC003795-RA	dna repair protein rad51	-7.49	41.24	5.51
LDEC019367-RA	atp synthase subunit mitochondrial	-7.48	1218.57	162.80
LDEC001024-RA	neo-calmodulin-like isoform x2	-7.48	39.17	5.23
LDEC007093-RA	eukaryotic translation initiation factor 5a	-7.48	276.31	36.95
LDEC016660-RA	protein ssxt isoform x1	-7.46	110.18	14.76
LDEC012681-RA	protein singed	-7.45	70.71	9.49
LDEC001044-RA	atp synthase subunit mitochondrial-like	-7.43	3271.53	440.02
LDEC012071-RA	PREDICTED: uncharacterized protein LOC663405 isoform X2	-7.42	48.69	6.56
LDEC002259-RA	eukaryotic translation initiation factor 4 gamma 3-like isoform x3	-7.40	689.58	93.20
LDEC010005-RA	venom protease-like	-7.40	43.35	5.86

LDEC014579-RA	probable aconitate mitochondrial	-7.38	74.62	10.12
LDEC002756-RA	jerky protein homolog-like	-7.37	42.62	5.78
LDEC001686-RA	rna-binding protein with serine-rich domain 1	-7.37	52.97	7.19
LDEC005806-RA	nascent polypeptide-associated complex subunit alpha	-7.37	477.15	64.76
LDEC020428-RA	ocia domain-containing protein 1	-7.37	43.15	5.86
LDEC015181-RA	nadh dehydrogenase	-7.36	73.93	10.04
LDEC016736-RA	epidermal cell surface receptor	-7.36	67.84	9.22
LDEC004591-RA	39s ribosomal protein mitochondrial	-7.36	39.07	5.31
LDEC004392-RA	importin subunit beta-1 isoform x2	-7.35	132.34	18.01
LDEC000955-RA	acidic leucine-rich nuclear phosphoprotein 32 family member a	-7.35	98.46	13.40
LDEC012341-RA	fibrillin-2 isoform x2	-7.35	67.45	9.18
LDEC004427-RA	low quality protein: water dikinase	-7.33	314.29	42.85
LDEC005713-RA	stomatin-like protein mitochondrial isoform x2	-7.33	39.24	5.35
LDEC014123-RA	atpase family aaa domain-containing protein 3	-7.33	107.92	14.73
LDEC004603-RA	cleavage and polyadenylation specificity factor subunit cg7185 isoform x3	-7.33	46.66	6.37
LDEC011549-RA	40s ribosomal protein s9	-7.32	728.41	99.45
LDEC016722-RA	polyribonucleotide nucleotidyltransferase mitochondrial	-7.32	50.63	6.91
LDEC008587-RA	inner nuclear membrane protein man1 isoform x1	-7.32	106.66	14.57
LDEC006874-RA	ribosomal protein l17	-7.31	1053.29	144.13
LDEC009032-RA	isocitrate dehydrogenase	-7.30	123.52	16.91
LDEC001326-RA	antichymotrypsin-2-like isoform x4	-7.30	759.01	103.98
LDEC009474-RA	nadh dehydrogenase subunit 3	-7.30	437.94	60.00
LDEC005428-RA	60s ribosomal protein l23a	-7.30	1714.84	235.03
LDEC000045-RA	ubiquinol-cytochrome c reductase complex 14kd subunit	-7.27	142.57	19.61
LDEC000773-RA	tetraspanin-9	-7.27	56.51	7.77
LDEC002447-RA	---NA---	-7.27	90.56	12.46
LDEC001300-RA	abc transporter g family member 20 isoform x2	-7.26	61.57	8.48
LDEC013922-RA	aldose reductase	-7.26	910.05	125.35
LDEC004433-RA	mitochondrial import receptor subunit tom20 homolog	-7.25	125.76	17.34
LDEC003940-RA	eukaryotic translation initiation factor 3 subunit a	-7.25	515.40	71.09
LDEC024395-RA	safb-like transcription modulator	-7.25	189.35	26.13
LDEC006513-RA	trifunctional purine biosynthetic protein adenosine-3	-7.24	126.17	17.42
LDEC013268-RA	probable medium-chain specific acyl-mitochondrial	-7.23	367.02	50.74
LDEC012374-RA	short-chain dehydrogenease reductase	-7.20	182.60	25.35
LDEC000192-RA	ubiquinol-cytochrome c reductase	-7.20	433.69	60.23
LDEC004487-RA	small nuclear ribonucleoprotein sm d1	-7.19	74.12	10.31
LDEC019895-RA	40s ribosomal protein s24	-7.19	765.16	106.48
LDEC007395-RA	proton-coupled amino acid transporter	-7.18	73.47	10.23

	4-like			
LDEC004199-RA	isoleucyl trna synthetase	-7.18	67.28	9.37
LDEC005235-RA	pantothenate kinase mitochondrial-like	-7.17	117.70	16.41
LDEC017191-RA	unkown protein	-7.17	212.70	29.69
LDEC003256-RA	40s ribosomal protein s3a	-7.16	1639.66	229.13
LDEC011578-RA	bifunctional purine biosynthesis protein purh	-7.16	41.08	5.74
LDEC003518-RA	malic enzyme	-7.15	83.76	11.72
LDEC015841-RA	splicing factor 3b subunit 2 isoform x2	-7.13	107.56	15.08
LDEC016586-RA	zinc finger protein 706-like	-7.13	55.71	7.81
LDEC000211-RA	heat shock protein 90	-7.12	633.88	88.98
LDEC014804-RA	probable multidrug resistance-associated protein lethal 03659	-7.09	272.24	38.40
LDEC003705-RA	malate mitochondrial	-7.07	680.62	96.28
LDEC004845-RA	lysyl-trna synthetase	-7.07	134.44	19.02
LDEC005730-RA	mitochondrial import inner membrane translocase subunit tim44	-7.06	68.41	9.69
LDEC019184-RA	protein transport protein sec61 subunit beta	-7.06	202.45	28.67
LDEC007874-RA	phosphoglycerate kinase	-7.06	76.09	10.78
LDEC022542-RA	t-complex protein 1 subunit epsilon	-7.05	119.75	16.99
LDEC023875-RA	26s proteasome non-atpase regulatory subunit 1	-7.05	125.25	17.77
LDEC000125-RA	nadh dehydrogenase	-7.04	220.94	31.37
LDEC021273-RA	zinc finger protein on ecdysone puffs	-7.03	470.79	66.95
LDEC014332-RA	glyceraldehyde-3-phosphate dehydrogenase	-7.03	1406.40	200.11
LDEC007895-RA	40s ribosomal protein s19a-like	-7.02	793.58	113.12
LDEC002667-RA	f-box lrr-repeat protein	-7.01	100.26	14.30
LDEC020824-RA	cystathionine beta-synthase	-7.01	98.61	14.06
LDEC005180-RA	ribosomal protein l4e	-7.00	178.17	25.47
LDEC000525-RA	low quality protein: calreticulin-like	-6.99	3864.38	553.22
LDEC010780-RA	atp synthase lipid-binding mitochondrial	-6.97	2770.89	397.32
LDEC008803-RA	very long-chain-fatty-acid-- ligase bubblegum isoform x1	-6.97	136.95	19.65
LDEC007378-RA	nadh-ubiquinone reductase 75 kda subunit precursor	-6.96	125.69	18.05
LDEC001409-RA	nadp-dependent malic enzyme	-6.96	148.13	21.29
LDEC010572-RA	dentin sialophosphoprotein isoform x1	-6.96	299.11	43.01
LDEC007862-RA	atp-dependent protease la	-6.95	51.03	7.34
LDEC008881-RA	glutathione s-transferase 1-6	-6.94	148.76	21.44
LDEC005880-RA	acetyl- mitochondrial	-6.94	184.23	26.56
LDEC009998-RA	-trans-enoyl- mitochondrial	-6.93	67.66	9.77
LDEC003583-RA	choline transporter-like protein 1	-6.92	159.03	22.97
LDEC006173-RA	probable cysteine mitochondrial	-6.92	52.18	7.54
LDEC019778-RA	triosephosphate isomerase	-6.92	302.96	43.79
LDEC001691-RA	cdgsh iron-sulfur domain-containing protein 2 homolog	-6.91	118.19	17.11

LDEC016513-RA	u1 small nuclear ribonucleoprotein a	-6.91	40.47	5.86
LDEC008865-RA	trans- -dihydrobenzene- -diol dehydrogenase-like	-6.90	83.28	12.07
LDEC003582-RA	cyclin-dependent kinase 4	-6.89	77.76	11.29
LDEC019107-RA	letm1 and ef-hand domain-containing protein anon- mitochondrial	-6.89	171.31	24.88
LDEC017885-RA	cd151 antigen-like	-6.86	113.90	16.60
LDEC019182-RA	adenylate kinase	-6.85	150.20	21.91
LDEC019751-RA	peroxiredoxin 1	-6.84	703.63	102.85
LDEC017853-RA	cellular fabp-like protein isoform 1 precursor	-6.84	238.04	34.80
LDEC021311-RA	6-phosphogluconate decarboxylating	-6.82	742.93	108.98
LDEC002528-RA	nucleolar gtp-binding protein 2	-6.81	40.68	5.98
LDEC016959-RA	actin-like protein 6b	-6.80	42.23	6.21
LDEC007566-RA	nadh-ubiquinone oxidoreductase 39 kda subunit	-6.80	210.04	30.90
LDEC011920-RA	hypoxia up-regulated protein 1 isoform x1	-6.78	301.47	44.45
LDEC020511-RA	mitochondrial inner membrane organizing system protein 1-like	-6.77	69.31	10.23
LDEC019597-RA	peroxiredoxin prdx5	-6.77	208.60	30.82
LDEC009161-RA	---NA---	-6.76	72.13	10.66
LDEC013421-RA	ttc27 protein	-6.76	56.78	8.40
LDEC018230-RA	39s ribosomal protein mitochondrial	-6.76	50.95	7.54
LDEC020216-RA	neurexin-4 isoform x1	-6.75	69.12	10.23
LDEC004435-RA	afg3-like protein 2	-6.74	179.90	26.68
LDEC010781-RA	pentatricopeptide repeat-containing protein mitochondrial-like	-6.74	84.79	12.58
LDEC003939-RA	rna-binding protein 34	-6.72	72.49	10.78
LDEC008037-RA	cubilin	-6.72	132.80	19.76
LDEC005064-RA	ribosomal protein s18	-6.72	738.16	109.92
LDEC003903-RA	nose resistant to fluoxetine protein 6	-6.71	101.93	15.19
LDEC021137-RA	activating transcription factor of chaperone-like	-6.70	1908.96	284.71
LDEC017031-RA	lanb2	-6.69	41.83	6.25
LDEC011767-RA	inter-alpha-trypsin inhibitor heavy chain h4-like isoform x2	-6.69	207.74	31.05
LDEC014307-RA	y-box factor homolog isoform x1	-6.68	1508.90	225.73
LDEC017900-RA	methylcrotonoyl- carboxylase subunit mitochondrial isoform x2	-6.68	93.44	13.98
LDEC020664-RA	kruppel-like protein 1	-6.67	63.35	9.49
LDEC005271-RA	small ubiquitin-related modifier 3	-6.67	64.65	9.69
LDEC008333-RA	adenosine kinase	-6.67	54.73	8.20
LDEC009952-RA	gpi transamidase component pig-s	-6.67	106.58	15.98
LDEC021583-RA	60s acidic ribosomal protein p0	-6.66	2752.51	413.18
LDEC006706-RA	d-arabinitol dehydrogenase 1	-6.66	56.97	8.55
LDEC006952-RA	long-chain-fatty-acid-- ligase 4 isoform x10	-6.66	900.76	135.27
LDEC001412-RA	eukaryotic translation initiation factor 4h	-6.65	308.90	46.48
LDEC007252-RA	proteasome activator complex subunit	-6.64	84.58	12.73

	3-like			
LDEC001727-RA	ribosome biogenesis protein bop1 homolog	-6.64	70.29	10.59
LDEC021639-RA	proton-coupled amino acid transporter 1	-6.62	46.01	6.95
LDEC014944-RA	probable sulfite mitochondrial-like	-6.61	60.46	9.14
LDEC006485-RA	isoform a	-6.60	41.24	6.25
LDEC009874-RA	odorant binding protein	-6.59	56.13	8.52
LDEC012329-RA	autophagy-related protein 13 homolog isoform x1	-6.59	64.58	9.80
LDEC015723-RA	PREDICTED: phosphoserine phosphatase [Tribolium castaneum]	-6.58	85.27	12.97
LDEC001698-RA	protein disulfide-isomerase a6	-6.58	463.85	70.54
LDEC010494-RA	---NA---	-6.57	57.24	8.71
LDEC004640-RA	digestive cysteine proteinase intestain	-6.57	55.69	8.48
LDEC005399-RA	splicing factor 3b subunit 3	-6.56	318.44	48.55
LDEC012938-RA	60s ribosomal protein l13a	-6.56	1018.03	155.23
LDEC012332-RA	eukaryotic translation initiation factor 3 subunit b	-6.56	441.05	67.26
LDEC002421-RA	sorbitol dehydrogenase	-6.56	390.51	59.57
LDEC016496-RA	arginase- mitochondrial	-6.55	41.99	6.41
LDEC001027-RA	serpin b12	-6.55	214.64	32.77
LDEC006693-RA	gelsolin-related protein of 125 kda isoform x2	-6.55	140.69	21.48
LDEC012646-RA	probable methylmalonate-semialdehyde dehydrogenase	-6.55	126.84	19.37
LDEC008716-RA	camp-responsive element-binding 2	-6.54	54.69	8.36
LDEC020284-RA	PREDICTED: uncharacterized protein LOC100142033	-6.54	1670.11	255.34
LDEC002547-RA	microtubule-associated protein rp eb family member 1	-6.54	264.87	40.51
LDEC003360-RA	guanine nucleotide-binding protein subunit beta-like protein isoform x1	-6.54	898.44	137.45
LDEC017313-RA	ribosomal protein s7	-6.53	1009.37	154.52
LDEC007974-RA	trimeric intracellular cation channel type b	-6.52	89.11	13.67
LDEC017994-RA	maguk p55 subfamily member 2	-6.52	50.65	7.77
LDEC011504-RA	tissue inhibitor of metalloproteases	-6.51	54.17	8.32
LDEC012058-RA	protein fam10a4	-6.51	90.01	13.83
LDEC009495-RA	3-hydroxyisobutyrate mitochondrial	-6.51	162.98	25.04
LDEC018083-RA	coleoptericin a	-6.51	242.23	37.22
LDEC011021-RA	4-aminobutyrate aminotransferase	-6.50	94.01	14.45
LDEC014102-RA	succinyl-coa:3-ketoacid-coenzyme a transferase	-6.50	55.36	8.52
LDEC004864-RA	eukaryotic translation initiation factor 5b	-6.50	51.78	7.97
LDEC002232-RA	probable atp-dependent rna helicase ddx23	-6.49	119.11	18.36
LDEC008332-RA	dna polymerase delta catalytic subunit	-6.48	44.57	6.87
LDEC000262-RA	upf0326 protein fam152b	-6.48	59.25	9.14
LDEC000769-RA	far upstream element-binding protein 1	-6.47	318.33	49.18
LDEC015758-RA	thioredoxin-related transmembrane protein 1-like	-6.47	109.21	16.87

LDEC002997-RA	phosphoglycolate phosphatase 2-like	-6.47	41.68	6.44
LDEC005097-RA	g-protein coupled receptor 158	-6.47	51.78	8.01
LDEC010788-RA	ribosomal l1 domain-containing protein cg13096-like	-6.46	44.42	6.87
LDEC008817-RA	gtp-binding protein	-6.45	60.23	9.34
LDEC009942-RA	isovaleryl coenzyme a dehydrogenase	-6.44	237.41	36.87
LDEC015134-RA	ribosomal protein s3	-6.43	1367.65	212.57
LDEC018225-RA	nadh dehydrogenase	-6.43	49.50	7.69
LDEC022234-RA	39s ribosomal protein mitochondrial	-6.43	86.37	13.44
LDEC021444-RA	eukaryotic translation initiation factor 2 subunit 2	-6.43	238.96	37.19
LDEC008331-RA	peroxisomal acyl-coenzyme a oxidase 3	-6.42	154.83	24.10
LDEC006148-RA	pyruvate dehydrogenase e1 component subunit mitochondrial	-6.42	387.14	60.27
LDEC011752-RA	solute carrier family 25 member 46-like	-6.42	48.63	7.58
LDEC004606-RA	atp synthase-coupling factor mitochondrial	-6.42	182.20	28.40
LDEC017743-RA	elongation factor 1 beta	-6.41	607.88	94.80
LDEC019482-RA	heat shock protein 70	-6.41	6992.21	1090.88
LDEC016962-RA	protein llp homolog	-6.41	51.07	7.97
LDEC021520-RA	mitochondrial import inner membrane translocase subunit tim17-b-like	-6.39	69.92	10.94
LDEC023680-RA	translocon-associated protein subunit beta	-6.39	168.02	26.29
LDEC002334-RA	protein disulfide-isomerase a3	-6.39	1631.85	255.34
LDEC017897-RA	multidrug resistance-associated protein 4-like	-6.39	54.38	8.52
LDEC022719-RA	suppressor of g2 allele of skp1 homolog	-6.38	43.84	6.87
LDEC003910-RA	pleiotrophic factor-alpha-1	-6.37	43.31	6.80
LDEC018924-RA	---NA---	-6.37	151.56	23.79
LDEC006938-RA	o-linked n-acetylglucosamine ogt	-6.36	255.32	40.12
LDEC000214-RA	androgen-induced gene 1 isoform x1	-6.36	129.16	20.31
LDEC001577-RA	nucleoside diphosphate kinase	-6.36	1075.29	169.17
LDEC004810-RA	t-complex protein 1 subunit eta	-6.36	119.40	18.79
LDEC016402-RA	protein ctla-2-alpha	-6.35	214.87	33.83
LDEC009170-RA	PREDICTED: uncharacterized protein LOC657400 isoform X1	-6.34	55.23	8.71
LDEC000870-RA	28s ribosomal protein mitochondrial	-6.33	42.75	6.76
LDEC000877-RA	neutral alpha-glucosidase ab	-6.32	185.55	29.37
LDEC006966-RA	pre-rrna-processing protein tsr1 homolog	-6.32	55.02	8.71
LDEC001398-RA	lamin dm0-like isoform x1	-6.31	272.11	43.12
LDEC004051-RA	hrp65 protein	-6.29	249.99	39.72
LDEC007975-RA	titin isoform x2	-6.29	297.19	47.26
LDEC000706-RA	proteasome subunit beta type-7-like	-6.29	152.76	24.30
LDEC019587-RA	acetyl-coa carboxylase	-6.28	67.66	10.78
LDEC018015-RA	agap005993-pa-like protein	-6.27	60.78	9.69
LDEC014259-RA	proteasome maturation protein	-6.27	81.61	13.01

LDEC013749-RA	agap001711-pa-like protein	-6.26	108.13	17.26
LDEC020234-RA	pre-mrna-processing factor 19	-6.26	87.23	13.94
LDEC010576-RA	atp-binding cassette sub-family b member mitochondrial	-6.25	54.15	8.67
LDEC018610-RA	ribosomal protein s20	-6.23	209.62	33.63
LDEC004320-RA	saccharopine dehydrogenase-like oxidoreductase	-6.23	49.61	7.97
LDEC005021-RA	glutamate mitochondrial-like	-6.22	245.86	39.53
LDEC011032-RA	amidophosphoribosyltransferase	-6.22	113.65	18.28
LDEC009180-RA	28s ribosomal protein mitochondrial-like	-6.21	50.24	8.09
LDEC008293-RA	protein disulfide-isomerase a3	-6.21	53.37	8.59
LDEC008158-RA	thioredoxin reductase mitochondrial isoform x5	-6.21	677.51	109.17
LDEC003658-RA	single-stranded dna-binding mitochondrial	-6.20	42.89	6.91
LDEC008233-RA	n-(5-amino-5-carboxypentanoyl)-l-cysteinyl-d-valine synthase	-6.20	57.41	9.26
LDEC008092-RA	---NA---	-6.20	104.55	16.87
LDEC017590-RA	aspartate mitochondrial	-6.19	648.75	104.72
LDEC015973-RA	microtubule-associated protein futsch-like	-6.18	136.48	22.07
LDEC006298-RA	h+ transporting atp synthase o subunit isoform 1	-6.18	350.81	56.76
LDEC002110-RA	g-protein coupled receptor 143-like	-6.18	54.08	8.75
LDEC003276-RA	ribosomal protein l27ae	-6.17	304.45	49.33
LDEC008216-RA	cytochrome b-c1 complex subunit mitochondrial	-6.16	499.24	81.01
LDEC002455-RA	neutral and basic amino acid transport protein rbat-like isoform x2	-6.14	54.00	8.79
LDEC021246-RA	venom metalloproteinase 3 isoform x2	-6.14	55.65	9.06
LDEC022329-RA	unconventional myosin-xv	-6.14	78.91	12.85
LDEC008741-RA	antichymotrypsin-2-like isoform x3	-6.14	59.71	9.73
LDEC014721-RA	inosine-5 -monophosphate dehydrogenase	-6.11	43.46	7.11
LDEC021670-RA	heat shock protein beta-1-like isoform 4	-6.11	254.52	41.64
LDEC017176-RA	e3 ubiquitin-protein ligase sina	-6.10	61.68	10.12
LDEC022805-RA	glutamate synthase	-6.09	233.86	38.40
LDEC013226-RA	60s ribosomal protein l32-like	-6.09	722.28	118.63
LDEC006147-RA	ran-specific gtpase-activating	-6.08	105.26	17.30
LDEC013030-RA	electron transfer flavoprotein subunit beta	-6.08	214.47	35.27
LDEC003947-RA	protoporphyrinogen oxidase	-6.08	45.57	7.50
LDEC010356-RA	atp synthase subunit mitochondrial-like	-6.07	765.10	126.13
LDEC009203-RA	reactive oxygen species modulator 1-like	-6.06	56.36	9.30
LDEC016958-RA	prefoldin subunit 6	-6.06	45.19	7.46
LDEC011968-RA	eukaryotic translation initiation factor 3 subunit e-like	-6.05	139.14	23.01
LDEC021150-RA	multiprotein bridging factor 1	-6.03	83.63	13.87
LDEC015496-RA	anopheles gambiae pest agap012587-pa	-6.03	58.89	9.77
LDEC011884-RA	proteasome subunit beta type-5	-6.03	125.50	20.82

LDEC008744-RA	PREDICTED: uncharacterized protein LOC661760	-6.03	70.14	11.64
LDEC016690-RA	atp synthase subunit mitochondrial	-6.02	316.47	52.54
LDEC012481-RA	lysyl oxidase homolog 3	-6.02	48.46	8.05
LDEC003244-RA	charged multivesicular body protein 2a	-6.02	70.58	11.72
LDEC007867-RA	arylphorin-like hexameric storage protein	-6.02	60.65	10.08
LDEC010853-RA	t-complex protein 1 subunit theta	-6.02	231.04	38.40
LDEC002958-RA	protein transport protein sec61 subunit alpha isoform 2	-6.01	1082.51	180.03
LDEC015088-RA	28s ribosomal protein mitochondrial	-6.01	109.21	18.16
LDEC012336-RA	cytochrome p450-like protein	-6.01	72.07	11.99
LDEC000084-RA	blastoderm-specific protein 25d- partial	-6.00	113.52	18.91
LDEC003052-RA	lamin dm0-like isoform x1	-6.00	99.42	16.56
LDEC004092-RA	low quality protein: leucine-rich repeat-containing protein ddb_g0290503	-6.00	47.10	7.85
LDEC002048-RA	cytochrome p450-like protein	-6.00	47.56	7.93
LDEC016646-RA	atp synthase subunit mitochondrial	-5.99	243.81	40.70
LDEC013744-RA	mitochondrial enolase superfamily member 1-like isoform x2	-5.98	578.88	96.75
LDEC020749-RA	prostaglandin f synthase-like	-5.98	78.74	13.16
LDEC019087-RA	39s ribosomal protein mitochondrial	-5.97	55.74	9.34
LDEC013266-RA	ribosomal protein l5	-5.96	252.43	42.38
LDEC000680-RA	nadh dehydrogenase 1 alpha subcomplex subunit 12	-5.94	83.49	14.06
LDEC018306-RA	isoform b	-5.94	131.01	22.07
LDEC014923-RA	clustered mitochondria protein homolog	-5.93	222.46	37.50
LDEC009910-RA	serine-arginine protein 55-like isoform x2	-5.93	223.97	37.77
LDEC008616-RA	double-stranded rna-binding protein staufen homolog 2 isoform x2	-5.92	606.84	102.46
LDEC002408-RA	carbonyl reductase	-5.92	143.32	24.22
LDEC021139-RA	nadh: ubiquinone dehydrogenase	-5.91	101.31	17.15
LDEC002400-RA	alpha-aminoadipic semialdehyde dehydrogenase	-5.90	81.88	13.87
LDEC020995-RA	probable aconitate mitochondrial	-5.90	158.19	26.80
LDEC005929-RA	leucine-rich ppr motif-containing mitochondrial	-5.90	309.94	52.54
LDEC012334-RA	probable medium-chain specific acyl-mitochondrial	-5.90	208.26	35.31
LDEC011587-RA	cytochrome p450-like protein	-5.89	146.44	24.84
LDEC016073-RA	60s ribosomal protein l9	-5.89	942.95	160.07
LDEC024057-RA	aminoacyl trna synthase complex-interacting multifunctional protein 1	-5.89	67.82	11.52
LDEC011628-RA	6-phosphogluconate decarboxylating	-5.88	124.56	21.17
LDEC005329-RA	alpha-( )-fucosyltransferase	-5.88	83.17	14.14
LDEC003987-RA	n-myc protein	-5.88	70.46	11.99
LDEC016658-RA	kelch domain-containing protein 10 homolog	-5.87	49.55	8.44
LDEC015476-RA	nadh dehydrogenase	-5.86	81.52	13.91
LDEC024312-RA	vacuolar protein sorting 4	-5.86	79.64	13.59

LDEC010050-RA	multidrug resistance-associated protein 4	-5.86	108.00	18.44
LDEC011802-RA	e3 ubiquitin-protein ligase siah1-like	-5.86	99.99	17.07
LDEC019546-RA	15-hydroxyprostaglandin dehydrogenase	-5.85	84.60	14.45
LDEC003012-RA	protein ovo isoform x2	-5.85	98.69	16.87
LDEC001382-RA	prostaglandin reductase 1	-5.84	73.51	12.58
LDEC006931-RA	phosphoribosylaminoimidazole carboxylase	-5.84	129.98	22.26
LDEC023462-RA	protein tumorous imaginal mitochondrial	-5.83	77.03	13.20
LDEC020338-RA	very long-chain-fatty-acid-- ligase bubblegum isoform x1	-5.82	316.66	54.37
LDEC009405-RA	proteasomal ubiquitin receptor adrm1	-5.81	115.57	19.88
LDEC005757-RA	low quality protein: nicalin	-5.81	111.45	19.18
LDEC017881-RA	PREDICTED: uncharacterized protein C14orf119	-5.81	63.08	10.86
LDEC001822-RA	tyrosine--trna cytoplasmic	-5.81	83.72	14.41
LDEC004942-RA	agap006339-pa-like protein	-5.81	68.28	11.76
LDEC005648-RA	alanine--trna cytoplasmic	-5.81	65.33	11.25
LDEC013999-RA	3 (2) -bisphosphate nucleotidase 1 isoform x2	-5.81	55.78	9.61
LDEC000451-RA	heat shock 70 kda protein cognate 3 isoform x1	-5.80	2582.96	444.98
LDEC004198-RA	heterochromatin protein	-5.80	83.76	14.45
LDEC017707-RA	sodium potassium-transporting atpase subunit beta-1	-5.79	74.20	12.81
LDEC003241-RA	ctp synthase	-5.79	68.49	11.84
LDEC008538-RA	probable wrky transcription factor protein 1	-5.78	70.92	12.27
LDEC014262-RA	leucine-rich repeat-containing protein egg-6	-5.78	843.00	145.81
LDEC021640-RA	dolichyl-diphosphooligosaccharide--protein glycosyltransferase subunit 2	-5.78	428.82	74.18
LDEC003895-RA	chaperone activity of bc1 complex-mitochondrial-like	-5.76	49.77	8.63
LDEC012761-RA	low quality protein: papilin-like	-5.76	125.17	21.72
LDEC004930-RA	pyruvate kinase	-5.76	563.95	97.96
LDEC022969-RA	proteasome subunit alpha type-2	-5.75	111.49	19.37
LDEC015842-RA	probable phosphoserine aminotransferase	-5.75	676.25	117.53
LDEC018617-RA	probable cytochrome p450 mitochondrial	-5.75	62.91	10.94
LDEC013544-RA	proteoglycan 4-like isoform x1	-5.75	251.99	43.83
LDEC002035-RA	nadh dehydrogenase	-5.75	72.93	12.69
LDEC008924-RA	nadh dehydrogenase	-5.75	68.22	11.87
LDEC001333-RA	cytochrome p450	-5.74	122.05	21.25
LDEC014931-RA	cytochrome p450 cyp6cr2	-5.73	100.76	17.58
LDEC009379-RA	elongation factor ts	-5.73	62.43	10.90
LDEC020510-RA	purine biosynthesis protein pur6	-5.72	97.04	16.95
LDEC021057-RA	protein qil1-like isoform x2	-5.72	48.48	8.48
LDEC014065-RA	juvenile hormone binding protein partial	-5.72	3379.63	591.06
LDEC006903-RA	prefoldin subunit 5	-5.70	48.58	8.52

LDEC003192-RA	hydroxysteroid dehydrogenase-like protein 2	-5.70	208.41	36.56
LDEC020846-RA	leucine-rich repeat-containing protein ddb_g0290503-like isoform x1	-5.70	159.99	28.08
LDEC007117-RA	udp-glucuronosyltransferase partial	-5.69	93.55	16.44
LDEC014658-RA	translocation associated membrane protein	-5.69	465.61	81.87
LDEC007239-RA	mitochondrial import inner membrane translocase subunit tim50-c-like isoform x1	-5.68	44.82	7.89
LDEC012384-RA	collagen alpha-2 chain-like	-5.67	764.74	134.92
LDEC002685-RA	cytochrome c oxidase subunit mitochondrial	-5.67	209.20	36.91
LDEC021558-RA	polyadenylate-binding protein 4-like	-5.67	106.45	18.79
LDEC011340-RA	60s ribosomal protein l35a	-5.66	259.65	45.86
LDEC001815-RA	ribosomal protein l10	-5.65	651.11	115.31
LDEC001334-RA	nadh-ubiquinone oxidoreductase 49 kda subunit	-5.64	206.82	36.64
LDEC020631-RA	ribosomal protein l26e	-5.64	589.42	104.45
LDEC010338-RA	sarcosine mitochondrial	-5.64	49.15	8.71
LDEC007478-RA	protein ltv1 homolog	-5.64	76.04	13.48
LDEC022647-RA	actin-binding protein anillin isoform x6	-5.62	72.05	12.81
LDEC005598-RA	fructose- -bisphosphatase 1	-5.62	97.73	17.38
LDEC019591-RA	eukaryotic translation initiation factor 3 subunit l	-5.62	248.08	44.14
LDEC009737-RA	glycerol-3-phosphate mitochondrial isoform x1	-5.62	350.75	62.46
LDEC010772-RA	facilitated trehalose transporter tret1-2 homolog	-5.61	249.76	44.49
LDEC004922-RA	protein disulfide isomerase	-5.61	1088.20	193.97
LDEC004666-RA	glycogen phosphorylase	-5.60	465.44	83.08
LDEC000874-RA	40s ribosomal protein s25	-5.60	498.71	89.06
LDEC002247-RA	protein cepu-1	-5.60	182.52	32.62
LDEC000473-RA	dna replication licensing factor mcm3	-5.60	46.34	8.28
LDEC015524-RA	pyruvate mitochondrial isoform x1	-5.59	100.93	18.05
LDEC015100-RA	mannose-6-phosphate isomerase-like	-5.59	59.37	10.62
LDEC013441-RA	innexin inx3	-5.58	94.24	16.87
LDEC002531-RA	guanine nucleotide-binding protein subunit beta-like protein isoform x2	-5.58	306.20	54.92
LDEC023144-RA	methylglutaconyl- mitochondrial	-5.58	66.86	11.99
LDEC010014-RA	eukaryotic peptide chain release factor gtp-binding subunit erf3a isoform x2	-5.57	178.69	32.07
LDEC010351-RA	phosphate carrier mitochondrial	-5.57	1287.30	231.16
LDEC005723-RA	acyl- -binding protein	-5.57	218.95	39.33
LDEC000705-RA	dolichyl-diphosphooligosaccharide--protein glycosyltransferase subunit 1	-5.56	341.25	61.36
LDEC010381-RA	tubulin beta-1 chain	-5.56	152.25	27.38
LDEC002690-RA	acyl- dehydrogenase family member mitochondrial	-5.56	51.66	9.30
LDEC011311-RA	dentin sialophospho	-5.55	68.08	12.27
LDEC014890-RA	agap008116-pa-like protein	-5.54	61.72	11.13
LDEC017992-RA	n-alpha-acetyltransferase 10	-5.54	60.84	10.98

LDEC004797-RA	protein translation factor sui1 homolog	-5.53	160.41	28.98
LDEC024457-RA	coatomer subunit zeta-1	-5.53	57.49	10.39
LDEC018349-RA	phytanoyl- peroxisomal-like	-5.53	113.67	20.55
LDEC001162-RA	f-box only protein 21-like	-5.53	47.27	8.55
LDEC013817-RA	fatty-acid amide hydrolase 2-b isoform x2	-5.51	47.16	8.55
LDEC003760-RA	prefoldin subunit 3	-5.51	74.50	13.51
LDEC003818-RA	methionine aminopeptidase	-5.51	52.52	9.53
LDEC008330-RA	60s ribosomal protein l18a	-5.51	962.50	174.80
LDEC011066-RA	coronin-6 isoform x2	-5.50	86.12	15.66
LDEC020917-RA	mfs-type transporter c6orf192 homolog	-5.48	82.25	15.00
LDEC014066-RA	bystin isoform 1	-5.48	55.21	10.08
LDEC009663-RA	carbonic anhydrase 1	-5.47	60.67	11.09
LDEC005395-RA	kynurenine formamidase	-5.47	56.17	10.27
LDEC015566-RA	protein fam98b	-5.46	56.07	10.27
LDEC019372-RA	transmembrane emp24 domain-containing protein 2	-5.45	145.92	26.76
LDEC013265-RA	inorganic pyrophosphatase-like	-5.45	268.91	49.33
LDEC007347-RA	s-phase kinase-associated protein 1	-5.44	146.29	26.87
LDEC021085-RA	beta-lactamase-like protein 2 homolog	-5.43	94.34	17.38
LDEC008093-RA	oxidase peroxidase	-5.42	80.62	14.88
LDEC006167-RA	secretion-regulating guanine nucleotide exchange factor	-5.42	123.98	22.89
LDEC016900-RA	40s ribosomal protein s15aa	-5.41	603.26	111.44
LDEC023834-RA	glycogenin-2-like isoform x3	-5.41	169.36	31.29
LDEC013099-RA	dna replication licensing factor mcm5	-5.41	65.04	12.03
LDEC023503-RA	omega-amidase nit2 isoform x2	-5.40	52.14	9.65
LDEC014486-RA	superoxide dismutase	-5.40	82.02	15.19
LDEC015355-RA	annexin isoform c	-5.40	797.99	147.84
LDEC015109-RA	eukaryotic translation initiation factor 3 subunit i	-5.40	197.68	36.64
LDEC010269-RA	ribosomal protein p2	-5.39	174.03	32.30
LDEC005731-RA	PREDICTED: uncharacterized protein C05D11.1-like	-5.39	191.84	35.62
LDEC005673-RA	mitochondrial inner membrane protein oxa1l	-5.38	54.88	10.19
LDEC001999-RA	eukaryotic translation initiation factor 3 subunit h	-5.38	181.03	33.63
LDEC016650-RA	-like protein subfamily c member 3	-5.38	96.29	17.89
LDEC004622-RA	er membrane protein complex subunit 1 isoform x2	-5.38	146.59	27.26
LDEC011790-RA	PREDICTED: uncharacterized protein LOC655078	-5.38	48.08	8.94
LDEC007859-RA	dna topoisomerase mitochondrial isoform x3	-5.37	285.81	53.24
LDEC005605-RA	hypothetical protein TcasGA2_TC000871	-5.37	53.66	10.00
LDEC006658-RA	gtp-binding protein 128up	-5.36	63.91	11.91
LDEC017300-RA	cd109 antigen	-5.35	69.35	12.97
LDEC003498-RA	proline dehydrogenase isoform 1	-5.35	85.81	16.05

LDEC021798-RA	zinc carboxypeptidase	-5.34	78.07	14.61
LDEC012143-RA	udp-glucose:glycoprotein glucosyltransferase	-5.34	83.03	15.55
LDEC003862-RA	upf0389 protein cg9231	-5.34	47.10	8.83
LDEC012711-RA	glucose-6-phosphate dehydrogenase isoform x1	-5.33	224.22	42.03
LDEC004036-RA	calcium-binding mitochondrial carrier protein aralar1 isoform x2	-5.33	159.59	29.92
LDEC007485-RA	enolase-phosphatase e1-like	-5.33	65.57	12.30
LDEC012397-RA	eukaryotic translation initiation factor 4e	-5.33	108.00	20.27
LDEC007882-RA	proteasome subunit beta type-2-like	-5.33	88.65	16.64
LDEC009098-RA	dna-(apurinic or apyrimidinic site) lyase- partial	-5.32	58.83	11.05
LDEC010158-RA	calcium-binding mitochondrial carrier protein aralar1 isoform x1	-5.32	70.44	13.24
LDEC003296-RA	PREDICTED: testican-2	-5.32	49.21	9.26
LDEC007561-RA	PREDICTED: uncharacterized protein LOC660966	-5.31	63.47	11.95
LDEC001934-RA	40s ribosomal protein s11-like	-5.30	481.10	90.78
LDEC006502-RA	signal recognition particle receptor subunit alpha homolog	-5.30	80.52	15.19
LDEC023320-RA	cytochrome c oxidase subunit va	-5.30	311.83	58.86
LDEC021922-RA	succinate dehydrogenase	-5.29	85.14	16.09
LDEC004176-RA	histidine decarboxylase-like	-5.28	88.05	16.68
LDEC009863-RA	pdz and lim domain protein zasp	-5.28	93.78	17.77
LDEC004034-RA	mitochondrial ribosomal protein l28	-5.27	50.69	9.61
LDEC022378-RA	ornithine decarboxylase 1-like	-5.27	456.51	86.56
LDEC004818-RA	basement membrane-specific heparan sulfate proteoglycan core protein isoform x8	-5.27	490.04	93.00
LDEC007159-RA	---NA---	-5.26	64.96	12.34
LDEC005599-RA	signal peptidase complex subunit 3	-5.26	126.82	24.10
LDEC000730-RA	eukaryotic translation initiation factor 3 subunit c	-5.26	219.70	41.76
LDEC007683-RA	imaginal disc growth factor 2 precursor	-5.26	412.26	78.36
LDEC008919-RA	26s protease regulatory subunit 10b	-5.26	118.94	22.62
LDEC019194-RA	protein lethal essential for life	-5.25	106.51	20.27
LDEC016847-RA	methionine aminopeptidase 2	-5.25	105.16	20.04
LDEC019904-RA	endoplasmic reticulum resident protein 29	-5.25	49.19	9.37
LDEC006191-RA	protein canopy-1-like	-5.25	47.54	9.06
LDEC007909-RA	atp synthase subunit mitochondrial	-5.25	132.76	25.31
LDEC005707-RA	ubiquitin carboxyl-terminal hydrolase 5	-5.24	63.29	12.07
LDEC013764-RA	protein canopy 4	-5.24	48.73	9.30
LDEC022662-RA	heterogeneous nuclear ribonucleoprotein a2 b1 homolog isoform x1	-5.24	233.57	44.57
LDEC005287-RA	ef1alpha-like isoform c	-5.23	171.01	32.69
LDEC013827-RA	translation initiation factor eif-2b subunit delta	-5.22	60.96	11.68
LDEC003313-RA	gpi transamidase component pig-t	-5.22	60.34	11.56

LDEC017975-RA	microtubule-associated protein futsch isoform x2	-5.21	326.97	62.73
LDEC009642-RA	nadh-ubiquinone oxidoreductase ashi subunit	-5.21	111.22	21.37
LDEC023039-RA	glycogen debranching enzyme-like	-5.20	128.49	24.69
LDEC012654-RA	ring finger protein nhl-1	-5.20	85.16	16.37
LDEC018433-RA	muscle m-line assembly protein unc-89 isoform x2	-5.20	430.45	82.73
LDEC019082-RA	aspartate cytoplasmic	-5.20	370.03	71.13
LDEC019706-RA	tyrosine-protein phosphatase 10d isoform x3	-5.20	104.36	20.08
LDEC019777-RA	tar dna-binding protein 43 isoform x2	-5.20	66.99	12.89
LDEC022100-RA	proteasome subunit alpha type-3	-5.19	111.35	21.44
LDEC021134-RA	multiple inositol polyphosphate phosphatase 1	-5.19	54.33	10.47
LDEC019801-RA	translocon-associated protein subunit alpha	-5.19	430.95	83.04
LDEC001643-RA	very long-chain specific acyl-mitochondrial	-5.17	65.08	12.58
LDEC024631-RA	4-aminobutyrate mitochondrial	-5.15	123.81	24.02
LDEC001197-RA	dolichyl-diphosphooligosaccharide protein glycotransferase	-5.15	123.14	23.91
LDEC018334-RA	probable elongation factor 1-delta isoform x3	-5.15	327.91	63.67
LDEC022183-RA	mitochondrial ubiquinol-cytochrome c reductase hinge protein	-5.15	105.60	20.51
LDEC018312-RA	regulator of nonsense transcripts 1-like isoform 1	-5.14	295.62	57.46
LDEC016867-RA	39s ribosomal protein mitochondrial	-5.14	63.06	12.27
LDEC002709-RA	gamma-glutamyltranspeptidase 1 isoform x2	-5.14	97.77	19.02
LDEC024223-RA	protein unzipped	-5.13	74.58	14.53
LDEC022276-RA	elongation of very long chain fatty acids protein aael008004	-5.13	54.33	10.59
LDEC020053-RA	proteasome subunit alpha type-7-1	-5.13	99.97	19.49
LDEC003069-RA	60s ribosomal protein l29	-5.12	151.69	29.61
LDEC001419-RA	ribosomal protein l37e	-5.11	343.18	67.22
LDEC014011-RA	transmembrane protein 53	-5.10	115.84	22.73
LDEC016521-RA	grpe protein mitochondrial	-5.09	59.88	11.76
LDEC021086-RA	atp-dependent rna helicase ddx1	-5.09	84.51	16.60
LDEC008074-RA	nadh dehydrogenase	-5.09	85.47	16.80
LDEC019810-RA	moesin ezrin radixin	-5.09	249.42	49.02
LDEC006930-RA	peptidoglycan-recognition protein 2	-5.09	73.30	14.41
LDEC016378-RA	tubulointerstitial nephritis	-5.08	138.01	27.15
LDEC001011-RA	peptidoglycan-recognition protein precursor	-5.08	143.93	28.32
LDEC007366-RA	thioredoxin domain-containing	-5.08	81.38	16.01
LDEC019760-RA	zinc finger matrin-type protein cg9776-like isoform x2	-5.07	79.41	15.66
LDEC005088-RA	eukaryotic translation initiation factor 6	-5.07	85.94	16.95
LDEC002696-RA	chromatin-remodeling complex atpase chain iswi	-5.07	121.91	24.06
LDEC008312-RA	ribosomal protein s4e	-5.06	1162.11	229.44
LDEC013326-RA	nadh-ubiquinone oxidoreductase 75 kda subunit	-5.06	196.36	38.79

LDEC017044-RA	eukaryotic translation initiation factor 3 subunit e	-5.06	95.10	18.79
LDEC017412-RA	dead-box helicase dbp80	-5.05	52.49	10.39
LDEC020683-RA	coleoptericin a	-5.05	159.62	31.64
LDEC004625-RA	ribosomal protein l5	-5.04	1469.12	291.31
LDEC012591-RA	dihydrolipoamide acetyltransferase component of pyruvate dehydrogenase	-5.04	458.22	90.97
LDEC001045-RA	protein transport protein sec23a isoform x2	-5.04	421.52	83.71
LDEC007618-RA	hemocyte protein-glutamine gamma-glutamyltransferase-like	-5.03	97.63	19.41
LDEC003764-RA	mucin-2-like isoform x2	-5.02	48.67	9.69
LDEC014500-RA	60s acidic ribosomal protein p0	-5.02	49.44	9.84
LDEC002159-RA	abhydrolase domain-containing protein 2	-5.02	101.98	20.31
LDEC024487-RA	probable signal peptidase complex subunit 2	-5.02	133.52	26.60
LDEC005971-RA	transcription elongation factor b polypeptide 2	-5.02	53.50	10.66
LDEC005492-RA	serine palmitoyltransferase 2	-5.01	182.50	36.44
LDEC001731-RA	electron transfer flavoprotein subunit mitochondrial	-5.00	331.01	66.17
LDEC012586-RA	double-stranded rna-specific editase 1 isoform x2	-5.00	96.71	19.33
LDEC012928-RA	eukaryotic translation initiation factor 3 subunit f-1	-5.00	250.28	50.04
LDEC014064-RA	myelin expression factor 2	-4.98	406.73	81.60
LDEC015788-RA	rab gdp dissociation inhibitor alpha	-4.98	57.33	11.52
LDEC019897-RA	transcription regulator protein bach2	-4.97	125.11	25.19
LDEC012430-RA	nucleolysin tiar	-4.97	114.44	23.05
LDEC011144-RA	tubulin alpha-1 chain	-4.97	5702.44	1148.42
LDEC006097-RA	ets dna-binding protein pokkuri	-4.96	115.57	23.28
LDEC017106-RA	ecdysone 20-monooxygenase isoform x2	-4.96	83.45	16.84
LDEC003703-RA	succinate dehydrogenase	-4.93	96.35	19.53
LDEC006832-RA	mitochondrial carrier homolog 2-like isoform x1	-4.93	60.44	12.27
LDEC012059-RA	mitochondrial nadh-ubiquinone oxidoreductase aggg subunit	-4.91	73.68	15.00
LDEC007755-RA	uroporphyrinogen decarboxylase isoform x5	-4.91	71.09	14.49
LDEC003431-RA	dolichyl-diphosphooligosaccharide--protein glycosyltransferase subunit stt3a	-4.89	114.15	23.36
LDEC005221-RA	inter-alpha-trypsin inhibitor heavy chain h4-like isoform x1	-4.88	61.61	12.62
LDEC014275-RA	microsomal triglyceride transfer protein large subunit	-4.88	78.51	16.09
LDEC000269-RA	vacuolar atp synthase subunit g	-4.88	200.36	41.09
LDEC018944-RA	restin homolog	-4.87	158.55	32.54
LDEC023464-RA	basement membrane-specific heparan sulfate proteoglycan core protein isoform x3	-4.86	104.05	21.41
LDEC000759-RA	vanin-like protein 1	-4.85	116.22	23.94
LDEC006781-RA	stress-induced-phosphoprotein 1	-4.85	207.82	42.85
LDEC004278-RA	sumo-conjugating enzyme ubc9-b	-4.85	132.03	27.23

LDEC004170-RA	serine threonine-protein kinase d3	-4.84	97.79	20.19
LDEC023328-RA	probable gpi-anchored adhesin-like protein pga55 isoform x1	-4.84	134.00	27.69
LDEC001838-RA	srsf protein kinase 1	-4.84	87.65	18.12
LDEC007681-RA	dnaj homolog subfamily c member 2	-4.83	100.76	20.86
LDEC007775-RA	dentin sialophospho	-4.81	77.84	16.17
LDEC018906-RA	apoptosis-inducing factor mitochondrial	-4.81	128.12	26.64
LDEC012197-RA	serine threonine-protein phosphatase alpha-2 isoform	-4.80	157.84	32.85
LDEC000781-RA	-like protein subfamily c member 11	-4.80	57.43	11.95
LDEC001926-RA	cop9 signalosome complex subunit 2	-4.80	92.23	19.22
LDEC002145-RA	chaoptin-like isoform x2	-4.79	155.58	32.50
LDEC014453-RA	intracellular protein transport protein uso1-like isoform x1	-4.76	90.87	19.10
LDEC008426-RA	synaptic vesicle glycoprotein 2c-like isoform x1	-4.75	170.64	35.94
LDEC009115-RA	sulfhydryl oxidase 2-like	-4.74	96.50	20.35
LDEC001813-RA	mitochondrial import inner membrane translocase subunit tim23 isoform x1	-4.74	76.31	16.09
LDEC020770-RA	alpha-tocopherol transfer isoform x2	-4.74	191.82	40.47
LDEC011677-RA	14-3-3 zeta	-4.73	521.57	110.35
LDEC007709-RA	golgin subfamily a member 2-like	-4.72	396.03	83.82
LDEC004105-RA	interferon-related developmental regulator 1	-4.72	320.65	67.89
LDEC004127-RA	leghemoglobin lb5-10	-4.72	79.68	16.87
LDEC016249-RA	eukaryotic translation initiation factor 1a	-4.72	217.53	46.13
LDEC004905-RA	heat shock 70 kda protein 4 isoform x1	-4.71	381.39	80.89
LDEC016119-RA	3-hydroxy-3-methylglutaryl-coenzyme a reductase	-4.71	80.44	17.07
LDEC024343-RA	PREDICTED: uncharacterized protein LOC659663	-4.71	54.67	11.60
LDEC004299-RA	isoform b	-4.71	610.88	129.72
LDEC018531-RA	mitochondrial 2-oxoglutarate malate carrier	-4.71	112.39	23.87
LDEC007668-RA	nuclear valosin-containing	-4.71	120.63	25.62
LDEC009407-RA	myb-binding protein 1a	-4.71	57.55	12.23
LDEC001129-RA	PREDICTED: uncharacterized protein LOC103314979	-4.71	348.28	74.02
LDEC004897-RA	3-hydroxyisobutyryl- mitochondrial isoform x1	-4.69	128.89	27.50
LDEC014304-RA	ecdysone-inducible protein e75 isoform x3	-4.69	210.90	45.00
LDEC013264-RA	selenium-binding protein 1	-4.69	53.27	11.37
LDEC017247-RA	dna-directed rna polymerase ii subunit rpb3	-4.68	71.73	15.31
LDEC017401-RA	eukaryotic initiation factor 4a-ii	-4.68	1299.55	277.53
LDEC016635-RA	calmodulin	-4.68	643.12	137.38
LDEC017469-RA	inorganic phosphate cotransporter isoform x1	-4.67	393.87	84.29
LDEC010996-RA	amidotransferase subunit	-4.66	56.20	12.07
LDEC012729-RA	acetyl- mitochondrial	-4.65	74.29	15.98
LDEC019774-RA	serine protease p146	-4.65	82.25	17.69

LDEC006771-RA	tar dna-binding protein 43 isoform x2	-4.65	90.93	19.57
LDEC021813-RA	translation elongation factor 2	-4.64	5818.79	1253.06
LDEC005583-RA	catalase	-4.64	1429.49	308.07
LDEC002289-RA	importin subunit alpha-4	-4.63	55.57	11.99
LDEC004511-RA	dna replication licensing factor mcm4	-4.63	57.20	12.34
LDEC006114-RA	gamma-interferon-inducible lysosomal thiol reductase-like	-4.62	430.93	93.35
LDEC018186-RA	nuclear fragile x mental retardation-interacting protein 1	-4.61	79.97	17.34
LDEC016481-RA	importin-5	-4.61	450.82	97.89
LDEC003999-RA	ankyrin repeat domain-containing protein 12-like isoform x1	-4.60	61.59	13.40
LDEC018591-RA	serine threonine-protein phosphatase 2a 65 kda regulatory subunit a alpha isoform	-4.60	236.96	51.56
LDEC016968-RA	translationally-controlled tumor protein homolog	-4.60	658.92	143.39
LDEC012869-RA	39s ribosomal protein mitochondrial	-4.58	58.10	12.69
LDEC022268-RA	uncharacterized polypeptide	-4.58	61.49	13.44
LDEC003626-RA	suppression of tumorigenicity 1	-4.57	214.08	46.79
LDEC017882-RA	methylcrotonoyl- carboxylase subunit mitochondrial	-4.57	205.84	45.04
LDEC019657-RA	iron-sulfur cluster assembly enzyme mitochondrial	-4.56	66.99	14.69
LDEC014079-RA	twitchin- partial	-4.55	73.47	16.13
LDEC005048-RA	inorganic phosphate cotransporter	-4.55	167.00	36.68
LDEC002665-RA	bag domain-containing protein samui isoform x1	-4.55	114.48	25.16
LDEC014810-RA	ubiquitin ribosomal protein s30e fusion protein	-4.55	557.54	122.53
LDEC013832-RA	upf0160 protein	-4.55	84.74	18.63
LDEC009154-RA	26s proteasome non-atpase regulatory subunit 4	-4.55	83.13	18.28
LDEC003523-RA	nadh dehydrogenase	-4.54	215.16	47.34
LDEC001976-RA	ubiquitin-conjugating enzyme e2	-4.54	90.49	19.92
LDEC005176-RA	probable beta-hexosaminidase fdl	-4.54	53.18	11.72
LDEC006638-RA	fatbody protein 3rev-g1	-4.53	299.28	66.01
LDEC018522-RA	proteasome subunit alpha type-6-like	-4.53	64.96	14.34
LDEC003840-RA	glutathione s-transferase omega-1	-4.53	79.56	17.58
LDEC021704-RA	malectin-a isoform x1	-4.53	86.44	19.10
LDEC017194-RA	serine protease s1a-1	-4.52	182.16	40.27
LDEC009387-RA	protein sda1 homolog	-4.52	54.17	11.99
LDEC001821-RA	histone deacetylase rpd3	-4.51	75.12	16.64
LDEC008067-RA	proteasome subunit alpha type-5	-4.51	75.79	16.80
LDEC008101-RA	sorting nexin-8	-4.51	53.02	11.76
LDEC020713-RA	eukaryotic translation initiation factor 3 subunit m	-4.50	139.54	30.98
LDEC007473-RA	e3 ubiquitin-protein ligase synoviolin a	-4.50	67.57	15.00
LDEC019787-RA	probable atp-dependent rna helicase ddx27-like	-4.50	54.46	12.11
LDEC017508-RA	protein catecholamines up	-4.49	60.75	13.51

LDEC005858-RA	n-acetylgalactosaminyltransferase 7	-4.49	61.15	13.63
LDEC002972-RA	nadh dehydrogenase subunit 2	-4.48	59.21	13.20
LDEC009411-RA	uncharacterized peptidase c1-like protein	-4.48	483.61	107.85
LDEC012243-RA	2-oxoisovalerate dehydrogenase subunit mitochondrial	-4.48	62.36	13.91
LDEC020702-RA	coatomer subunit beta	-4.48	332.57	74.18
LDEC010455-RA	nadh dehydrogenase	-4.47	229.13	51.21
LDEC006159-RA	neutral and basic amino acid transport protein rbat	-4.47	112.98	25.27
LDEC001290-RA	t-complex protein 1 subunit zeta	-4.46	164.03	36.76
LDEC007869-RA	dnaj homolog subfamily a member 1	-4.46	367.75	82.46
LDEC011907-RA	h aca ribonucleoprotein complex subunit 4	-4.44	132.28	29.76
LDEC022022-RA	lysozyme i-2	-4.44	187.75	42.26
LDEC011415-RA	laminin subunit beta-1	-4.44	423.24	95.31
LDEC002228-RA	glucosidase 2 subunit beta	-4.44	341.80	76.99
LDEC021799-RA	translocon-associated protein subunit gamma	-4.43	218.05	49.18
LDEC014662-RA	high mobility group protein	-4.43	507.12	114.45
LDEC003309-RA	probable methylmalonate-semialdehyde dehydrogenase	-4.43	252.77	57.11
LDEC001140-RA	organic cation transporter	-4.43	66.38	15.00
LDEC018331-RA	globin-like partial	-4.43	116.85	26.40
LDEC015301-RA	probable splicing arginine serine-rich 7	-4.42	113.63	25.70
LDEC021225-RA	pre-mrna 3 -end-processing factor fip1	-4.42	77.88	17.62
LDEC006019-RA	succinate dehydrogenase	-4.42	102.98	23.32
LDEC017339-RA	transcription elongation factor s-ii	-4.41	65.33	14.80
LDEC019998-RA	tumor suppressor candidate 3	-4.41	84.32	19.14
LDEC021104-RA	low quality protein: dihydropteridine reductase	-4.40	87.71	19.92
LDEC018585-RA	actin-related protein 5	-4.40	102.62	23.32
LDEC014393-RA	u1 small nuclear ribonucleoprotein 70 kda	-4.40	56.55	12.85
LDEC008758-RA	ovalbumin-related protein x-like isoform x1	-4.40	108.21	24.61
LDEC001521-RA	glutathione s-transferase	-4.39	677.74	154.41
LDEC003144-RA	splicing factor 3a subunit 1	-4.37	83.72	19.14
LDEC013534-RA	inositol oxygenase	-4.37	60.71	13.91
LDEC009536-RA	annexin-b9-like isoform x1	-4.36	97.67	22.38
LDEC013201-RA	hypothetical protein YQE_09086, partial	-4.36	126.42	28.98
LDEC005746-RA	probable enoyl- mitochondrial-like	-4.36	173.77	39.84
LDEC012324-RA	flavin reductase	-4.35	66.44	15.27
LDEC019633-RA	glutathione peroxidase	-4.34	147.59	33.98
LDEC008493-RA	cystathionine beta-synthase isoform x2	-4.33	154.41	35.62
LDEC010407-RA	oxygen-dependent coproporphyrinogen-iii oxidase	-4.33	79.83	18.44
LDEC014481-RA	profilin- partial	-4.33	149.26	34.49
LDEC018508-RA	chitobiosyldiphosphodolichol beta-mannosyltransferase	-4.32	69.48	16.09

LDEC006763-RA	thioredoxin-like protein 1	-4.31	93.99	21.80
LDEC004858-RA	ubiquitin-conjugating enzyme e2 g1	-4.31	128.62	29.84
LDEC004408-RA	serine threonine-protein phosphatase 2a 56 kda regulatory subunit gamma isoform isoform x2	-4.31	61.95	14.37
LDEC016394-RA	low quality protein: hemocytin-like	-4.31	86.81	20.16
LDEC003194-RA	pyridine nucleotide-disulfide oxidoreductase domain-containing protein 1	-4.30	65.80	15.31
LDEC015854-RA	probable gpi-anchored adhesin-like protein pga55	-4.29	339.75	79.21
LDEC007446-RA	calcium-independent phospholipase a2-gamma	-4.28	68.28	15.94
LDEC009010-RA	ribonuclease kappa	-4.28	83.57	19.53
LDEC004780-RA	proteasome subunit beta type-1	-4.28	146.84	34.33
LDEC001911-RA	protein casc3	-4.28	70.31	16.44
LDEC012412-RA	vacuolar h	-4.27	828.46	193.82
LDEC009685-RA	sodium potassium-transporting atpase subunit beta-2 isoform x2	-4.26	87.11	20.43
LDEC016596-RA	tricarboxylate transport mitochondrial	-4.26	135.58	31.80
LDEC000729-RA	uroporphyrinogen decarboxylase	-4.26	96.43	22.62
LDEC012668-RA	PREDICTED: uncharacterized protein LOC100141567 isoform X3	-4.26	156.73	36.80
LDEC002539-RA	chitinase 5 precursor	-4.26	377.45	88.63
LDEC003788-RA	trypsin 7	-4.26	337.68	79.29
LDEC022440-RA	probable multidrug resistance-associated protein lethal 03659	-4.26	322.30	75.70
LDEC016760-RA	succinyl- ligase	-4.25	127.55	30.04
LDEC001591-RA	oligosaccharyl transferase	-4.25	271.00	63.83
LDEC015793-RA	protein will die slowly	-4.25	78.11	18.40
LDEC017703-RA	udp-galactose translocator	-4.23	57.55	13.59
LDEC005597-RA	atp-binding cassette sub-family f member 2	-4.23	137.32	32.46
LDEC001115-RA	probable transaldolase	-4.23	479.20	113.28
LDEC018589-RA	aldose reductase	-4.22	1143.22	271.16
LDEC017189-RA	serine protease mitochondrial-like	-4.22	55.32	13.12
LDEC001596-RA	26s proteasome non-atpase regulatory subunit 13	-4.21	184.65	43.83
LDEC013137-RA	protein fam57a isoform x1	-4.21	62.20	14.76
LDEC004708-RA	26s proteasome non-atpase regulatory subunit 11	-4.21	109.63	26.05
LDEC015913-RA	glycogen	-4.19	59.12	14.10
LDEC011419-RA	dolichyl-diphosphooligosaccharide--protein glycosyltransferase subunit stt3a	-4.19	64.12	15.31
LDEC004919-RA	leucine--trna cytoplasmic	-4.18	226.94	54.26
LDEC014975-RA	casein kinase ii subunit beta	-4.17	116.36	27.93
LDEC018370-RA	soluble trehalase	-4.16	109.97	26.40
LDEC004803-RA	glutathione s-	-4.15	421.29	101.40
LDEC015605-RA	40s ribosomal protein s29	-4.15	159.36	38.40
LDEC007233-RA	protein sec13 homolog	-4.15	89.05	21.48

LDEC016325-RA	low quality protein: neuroglian-like	-4.14	336.97	81.48
LDEC018963-RA	juvenile hormone binding protein partial	-4.14	3712.94	897.85
LDEC017855-RA	proteasome subunit alpha type-1	-4.13	107.89	26.09
LDEC004465-RA	krueppel homolog 2	-4.13	126.09	30.51
LDEC003389-RA	cysteine proteinase cg12163	-4.13	299.22	72.46
LDEC002208-RA	serine threonine-protein kinase d3-like	-4.12	74.08	17.97
LDEC019169-RA	monocarboxylate transporter 10-like isoform 1	-4.12	63.26	15.35
LDEC004461-RA	unc93-like protein	-4.12	260.98	63.40
LDEC002449-RA	carbonic anhydrase 6-like	-4.12	58.04	14.10
LDEC006618-RA	protein aubergine	-4.11	107.98	26.25
LDEC009490-RA	single-stranded dna-binding protein 3 isoform x1	-4.11	122.12	29.73
LDEC005079-RA	ubiquitin carboxyl-terminal hydrolase 2-like isoform x2	-4.10	1196.17	291.51
LDEC014615-RA	retinol dehydrogenase 12	-4.10	80.92	19.73
LDEC018767-RA	cop9 signalosome complex subunit 3	-4.10	60.25	14.69
LDEC002595-RA	leucine-rich repeat and immunoglobulin-like domain-containing nogo receptor-interacting protein 2 isoform x1	-4.10	215.96	52.65
LDEC013103-RA	golgi reassembly-stacking protein 2	-4.10	66.92	16.33
LDEC015948-RA	ribosomal protein l35	-4.10	456.66	111.48
LDEC010108-RA	valine--trna ligase	-4.09	61.22	14.96
LDEC007077-RA	trans-2-enoyl- mitochondrial	-4.09	147.95	36.17
LDEC015708-RA	rna-binding protein luc7-like 2 isoform x2	-4.09	67.39	16.48
LDEC007525-RA	26s proteasome non-atpase regulatory subunit 7-like	-4.08	92.46	22.66
LDEC017243-RA	neurexin-4-like isoform x2	-4.08	97.19	23.83
LDEC015606-RA	proteasome subunit beta type-3-like	-4.07	104.07	25.58
LDEC018661-RA	protein ergic-53	-4.06	187.28	46.13
LDEC024311-RA	casein kinase ii subunit alpha	-4.06	111.45	27.46
LDEC003450-RA	programmed cell death protein 4	-4.06	148.18	36.52
LDEC013015-RA	profilin	-4.06	92.71	22.85
LDEC011490-RA	thioredoxin-2-like isoform x2	-4.06	561.27	138.39
LDEC013727-RA	agap006602-pa-like protein	-4.05	446.70	110.19
LDEC005436-RA	mitochondrial intermediate peptidase	-4.05	67.38	16.64
LDEC001743-RA	aldo-keto reductase	-4.05	102.48	25.31
LDEC000871-RA	cytochrome p450-like protein	-4.05	93.59	23.12
LDEC018934-RA	bifunctional aminoacyl-trna synthetase	-4.05	88.80	21.95
LDEC021657-RA	aldehyde dimeric nadp-preferring-like	-4.04	114.96	28.44
LDEC016693-RA	mitochondrial import receptor subunit tom70-like	-4.04	107.00	26.48
LDEC024072-RA	nucleolar complex protein 2 homolog	-4.03	59.77	14.84
LDEC000098-RA	zinc finger protein	-4.03	169.55	42.11
LDEC000976-RA	mitochondrial enolase superfamily member 1-like isoform x2	-4.02	383.90	95.39

LDEC004802-RA	60s ribosomal protein l30	-4.01	623.36	155.27
LDEC011295-RA	hypothetical protein TcasGA2_TC004643	-4.01	66.28	16.52
LDEC021961-RA	serine protease inhibitor i ii-like	-4.01	99.28	24.76
LDEC014412-RA	atp-dependent rna helicase p62 isoform x1	-4.01	165.30	41.25
LDEC020802-RA	rna polymerase ii second largest subunit	-4.01	114.06	28.48
LDEC019637-RA	solute carrier family 35 member b1	-4.00	70.40	17.62
LDEC016032-RA	low quality protein: transcriptional activator haca	-4.00	160.45	40.15
LDEC006653-RA	melanotransferrin	-3.99	122.72	30.74
LDEC016520-RA	phosphatase 2c gamma	-3.98	124.00	31.13
LDEC022464-RA	zinc finger rna-binding protein	-3.98	136.13	34.18
LDEC014814-RA	chromodomain-helicase-dna-binding protein mi-2 homolog isoform x1	-3.97	290.43	73.12
LDEC001530-RA	multidrug resistance-associated protein 1 isoform x1	-3.97	88.90	22.38
LDEC001054-RA	dynein light chain cytoplasmic	-3.97	75.98	19.14
LDEC005454-RA	transmembrane gtpase marf	-3.96	168.17	42.42
LDEC020897-RA	lachesin	-3.96	63.62	16.05
LDEC021211-RA	pre-mrna-processing-splicing factor 8	-3.96	75.37	19.02
LDEC009280-RA	phd and ring finger domain-containing protein 1 isoform x1	-3.95	205.94	52.15
LDEC014258-RA	dolichyl-phosphate beta-glucosyltransferase	-3.95	68.01	17.23
LDEC018365-RA	nipped-b-like protein b	-3.95	115.78	29.33
LDEC000046-RA	akirin-2 isoform 2	-3.94	175.41	44.49
LDEC014226-RA	antichymotrypsin-2-like isoform x2	-3.94	196.03	49.72
LDEC014285-RA	transitional endoplasmic reticulum atpase ter94	-3.94	620.04	157.30
LDEC021660-RA	eukaryotic translation initiation factor 4 gamma 3-like isoform x3	-3.92	135.81	34.61
LDEC020392-RA	egf domain-specific o-linked n-acetylglucosamine transferase	-3.92	67.89	17.30
LDEC000527-RA	cohesin subunit sa-1	-3.92	90.12	23.01
LDEC017533-RA	protein singed	-3.91	103.96	26.56
LDEC003482-RA	cysteine proteinase	-3.91	518.88	132.61
LDEC005267-RA	glutamine--fructose-6-phosphate aminotransferase	-3.91	86.92	22.23
LDEC009311-RA	arginine--trna cytoplasmic-like	-3.91	94.51	24.18
LDEC016723-RA	malate cytoplasmic	-3.89	236.75	60.82
LDEC014164-RA	v-type proton atpase subunit d 1	-3.89	269.92	69.37
LDEC015365-RA	low quality protein: sideroflexin-2-like	-3.89	66.53	17.11
LDEC019969-RA	pre-mrna-splicing factor atp-dependent rna helicase prp1	-3.88	65.98	16.99
LDEC006669-RA	---NA---	-3.88	1728.29	445.21
LDEC017478-RA	serine arginine-rich splicing factor 1-like	-3.88	161.89	41.72
LDEC000643-RA	golgi phosphoprotein 3 homolog rotini	-3.88	76.98	19.84
LDEC020562-RA	hydroxysteroid dehydrogenase-like protein 2	-3.87	59.77	15.43
LDEC005900-RA	serine palmitoyltransferase 1	-3.87	101.01	26.09

LDEC014139-RA	tolloid-like protein 1	-3.87	68.93	17.81
LDEC020680-RA	acyl- dehydrogenase	-3.87	197.55	51.05
LDEC005785-RA	mini-chromosome maintenance complex-binding protein	-3.86	389.10	100.70
LDEC010722-RA	swi snf complex subunit smarcc2	-3.86	118.23	30.62
LDEC022610-RA	poly	-3.86	72.20	18.71
LDEC005922-RA	probable dol-p-man:man c -pp-dol alpha- -mannosyltransferase	-3.86	92.69	24.02
LDEC010654-RA	alpha-mannosidase 2	-3.86	77.72	20.16
LDEC022836-RA	udp-glucuronosyltransferase 2b7	-3.85	83.11	21.56
LDEC011225-RA	eukaryotic translation initiation factor 3 subunit d	-3.85	195.92	50.94
LDEC008150-RA	coatomer subunit alpha	-3.85	364.63	94.80
LDEC010782-RA	dna topoisomerase 2	-3.85	97.48	25.35
LDEC012328-RA	transmembrane emp24 domain-containing protein bai	-3.84	143.53	37.34
LDEC018713-RA	serine threonine-protein phosphatase 5	-3.84	61.21	15.94
LDEC000698-RA	phenylalanyl-trna synthetase beta chain	-3.84	66.15	17.23
LDEC009627-RA	attacin-like immune protein	-3.83	172.48	45.00
LDEC003904-RA	signal peptidase complex catalytic subunit sec11c	-3.83	125.61	32.81
LDEC005928-RA	ccr4-not transcription complex subunit 7	-3.83	96.29	25.16
LDEC011236-RA	thioredoxin reductase protein	-3.83	103.90	27.15
LDEC004703-RA	clathrin light chain isoform x2	-3.82	111.87	29.26
LDEC001151-RA	caax prenyl protease 1 homolog	-3.82	86.77	22.69
LDEC001486-RA	prostaglandin reductase 1-like	-3.82	113.86	29.80
LDEC017318-RA	abhydrolase domain-containing protein 16a	-3.82	81.75	21.41
LDEC010566-RA	pre-mrna-splicing factor clf-	-3.81	287.59	75.50
LDEC010945-RA	60s ribosomal protein l28	-3.80	542.89	142.81
LDEC016252-RA	26s protease regulatory subunit 4	-3.80	312.00	82.11
LDEC014315-RA	ring finger protein 10	-3.80	81.63	21.48
LDEC016567-RA	cwf19-like protein 2 homolog	-3.79	102.56	27.03
LDEC003474-RA	glycine--trna ligase	-3.79	73.70	19.45
LDEC011664-RA	probable atp-dependent rna helicase ddx47-like	-3.79	67.76	17.89
LDEC023498-RA	probable multidrug resistance-associated protein lethal 03659	-3.78	151.54	40.08
LDEC008590-RA	ubiquitin-like modifier-activating enzyme 1-like	-3.78	421.02	111.36
LDEC014838-RA	transmembrane emp24 domain-containing protein 7	-3.78	84.51	22.38
LDEC004244-RA	short-chain dehydrogenase	-3.77	134.39	35.62
LDEC004660-RA	atp synthase subunit mitochondrial	-3.77	322.85	85.70
LDEC002238-RA	protein lingerer isoform x1	-3.76	350.81	93.28
LDEC004918-RA	dolichol-phosphate mannosyltransferase	-3.76	66.69	17.73
LDEC017398-RA	probable peroxisomal acyl-coenzyme a oxidase 1	-3.76	217.82	58.00
LDEC002755-RA	atp-binding cassette sub-family e member 1	-3.75	85.66	22.81

LDEC023585-RA	propionyl- carboxylase alpha mitochondrial	-3.75	116.83	31.17
LDEC007264-RA	dnaj homolog subfamily b member 11	-3.75	126.32	33.71
LDEC017888-RA	v-type proton atpase subunit c	-3.74	77.32	20.66
LDEC019699-RA	PREDICTED: uncharacterized protein LOC103578083 isoform X1	-3.74	120.28	32.19
LDEC012486-RA	cytochrome c oxidase assembly protein cox15	-3.74	102.60	27.46
LDEC004221-RA	sterol regulatory element-binding protein 1	-3.73	127.78	34.26
LDEC008269-RA	vanin-like protein 1	-3.73	81.40	21.83
LDEC020017-RA	rna-binding protein 25 isoform x1	-3.73	84.30	22.62
LDEC000033-RA	upf0364 protein c6orf211 homolog	-3.72	79.26	21.29
LDEC016020-RA	endoplasmic reticulum resident protein 44 isoform x3	-3.72	62.16	16.72
LDEC018788-RA	aldo-keto reductase	-3.72	663.62	178.62
LDEC008955-RA	n-acetyltransferase 10	-3.71	68.47	18.44
LDEC001327-RA	yellow-1 precursor	-3.71	103.69	27.93
LDEC017431-RA	2-hydroxyacylsphingosine 1-beta-galactosyltransferase-like	-3.71	179.57	48.40
LDEC015412-RA	aquaporin -like	-3.71	88.59	23.91
LDEC018366-RA	gamma-aminobutyric acid receptor-associated protein	-3.70	203.35	54.92
LDEC000615-RA	gtp-binding protein sar2	-3.69	171.41	46.40
LDEC007703-RA	peroxisomal multifunctional enzyme type 2	-3.69	458.81	124.21
LDEC014321-RA	filamin-a isoform x4	-3.69	349.91	94.88
LDEC024353-RA	beta- -galactosyltransferase 5-like	-3.69	126.01	34.18
LDEC019981-RA	lipoamide acyltransferase component of branched-chain alpha-keto acid dehydrogenase mitochondrial	-3.69	67.95	18.44
LDEC013018-RA	coatomer subunit beta	-3.68	224.60	61.05
LDEC003295-RA	atpase inhibitor mai- mitochondrial	-3.67	211.09	57.54
LDEC022529-RA	cytochrome p450 6bq11	-3.67	488.63	133.20
LDEC004607-RA	protein aubergine	-3.67	80.79	22.03
LDEC003817-RA	atp-dependent zinc metalloprotease yme1 homolog	-3.67	78.34	21.37
LDEC005655-RA	zinc finger ccch domain-containing protein 18	-3.66	70.27	19.18
LDEC016956-RA	excitatory amino acid transporter 3-like	-3.66	66.67	18.20
LDEC013903-RA	ubiquitin-conjugating enzyme e2 variant 2	-3.66	247.47	67.61
LDEC000626-RA	protein ctla-2-alpha	-3.66	127.64	34.88
LDEC019516-RA	filamin-a isoform x3	-3.65	495.10	135.46
LDEC017103-RA	lysosome-associated membrane glycoprotein 1	-3.64	326.55	89.76
LDEC006647-RA	sh3 domain-binding protein 5 homolog	-3.63	91.67	25.23
LDEC017407-RA	utp-glucose-1-phosphate uridylyltransferase 2	-3.63	255.46	70.35
LDEC015047-RA	inorganic phosphate cotransporter	-3.63	134.27	37.03
LDEC004586-RA	integrin alpha-ps2	-3.63	101.83	28.08
LDEC001319-RA	polya-binding protein	-3.63	917.56	253.11
LDEC006692-RA	pyrroline-5-carboxylate dehydrogenase	-3.62	169.61	46.79

LDEC017481-RA	hrp65 protein	-3.62	79.97	22.07
LDEC013811-RA	isoform a	-3.62	66.82	18.48
LDEC015374-RA	rhomboid protein mitochondrial	-3.61	89.95	24.92
LDEC010953-RA	cop9 signalosome complex subunit 5	-3.61	65.98	18.28
LDEC016584-RA	twitchin isoform x5	-3.61	232.15	64.33
LDEC006875-RA	histidine--trna cytoplasmic isoform x2	-3.60	77.47	21.52
LDEC004069-RA	fructose -bisphosphate aldolase	-3.59	785.59	218.58
LDEC010420-RA	ubiquilin-1	-3.58	273.16	76.25
LDEC013028-RA	phosphatidylserine synthase 1	-3.58	126.03	35.19
LDEC004664-RA	amp deaminase 2 isoform x6	-3.58	96.66	27.03
LDEC002089-RA	nadph--cytochrome p450 reductase isoform x2	-3.57	204.75	57.30
LDEC002336-RA	cg12262-pa	-3.57	75.08	21.01
LDEC009738-RA	prostatic acid phosphatase-like	-3.57	331.65	92.96
LDEC013540-RA	eukaryotic translation initiation factor 5b	-3.57	93.32	26.17
LDEC009940-RA	endoplasmic reticulum lectin 1 isoform x2	-3.57	89.13	25.00
LDEC005881-RA	protein ptcd3 mitochondrial	-3.56	131.19	36.83
LDEC005376-RA	transmembrane 9 superfamily member 3	-3.55	217.84	61.33
LDEC011289-RA	ig-like and fibronectin type-iii domain-containing protein	-3.55	94.87	26.72
LDEC008622-RA	fatty acyl- reductase cg8306	-3.54	90.08	25.43
LDEC019150-RA	organic cation transporter protein	-3.54	107.60	30.43
LDEC004247-RA	v-type proton atpase subunit c	-3.54	238.19	67.38
LDEC011539-RA	enhancer of mrna-decapping protein 4	-3.53	74.12	21.01
LDEC019195-RA	protein phosphatase 1d	-3.52	79.49	22.58
LDEC006756-RA	ubiquitin-conjugating enzyme e2-17 kda	-3.52	389.67	110.70
LDEC015834-RA	calponin transgelin	-3.51	225.22	64.10
LDEC013064-RA	nad kinase-like isoform x12	-3.51	79.51	22.66
LDEC009944-RA	receptor-type tyrosine-protein phosphatase f-like isoform x2	-3.51	121.53	34.65
LDEC017679-RA	peroxisomal acyl-coenzyme a oxidase 3-like	-3.51	620.85	177.06
LDEC013286-RA	muscle m-line assembly protein unc-partial	-3.50	524.44	149.68
LDEC010965-RA	dentin sialophospho	-3.50	128.49	36.76
LDEC003081-RA	prostaglandin reductase 1	-3.50	92.84	26.56
LDEC000157-RA	nadh-cytochrome b5 reductase 2 isoform x1	-3.49	179.15	51.33
LDEC001337-RA	diacylglycerol kinase theta isoform x2	-3.48	90.62	26.01
LDEC017538-RA	bifunctional 3 -phosphoadenosine 5 -phosphosulfate synthase	-3.48	123.94	35.58
LDEC011900-RA	proteasome subunit beta type-4	-3.48	149.87	43.04
LDEC000581-RA	cyclic amp response element-binding protein a-like	-3.48	118.94	34.18
LDEC000275-RA	receptor expression-enhancing protein 5	-3.48	74.22	21.33
LDEC000050-RA	solute carrier family 25 member 35	-3.47	133.06	38.36
LDEC017466-RA	juvenile hormone binding protein	-3.46	110.53	31.95

	partial			
LDEC004907-RA	cofilin actin-depolymerizing factor homolog	-3.46	247.33	71.56
LDEC017833-RA	aminoacylase-1 isoform x1	-3.45	73.81	21.37
LDEC001415-RA	threonine--trna cytoplasmic isoform x1	-3.45	184.54	53.43
LDEC016636-RA	poly -specific endoribonuclease homolog	-3.45	74.56	21.60
LDEC009109-RA	microsomal glutathione s-transferase 1	-3.45	141.19	40.94
LDEC012673-RA	ethanolaminephosphotransferase 1-like	-3.44	125.99	36.60
LDEC014918-RA	non-specific lipid-transfer protein	-3.44	452.28	131.48
LDEC009905-RA	26s protease regulatory subunit 6a	-3.44	168.88	49.10
LDEC023847-RA	tricarboxylate transport mitochondrial	-3.44	315.05	91.60
LDEC010165-RA	hypothetical protein SINV_05686	-3.44	86.35	25.12
LDEC018429-RA	6-phosphofructo-2-kinase fructose- -bisphosphatase	-3.44	148.74	43.28
LDEC017037-RA	isoform a	-3.43	102.39	29.84
LDEC008052-RA	PREDICTED: uncharacterized protein LOC659018	-3.43	146.44	42.69
LDEC010057-RA	monocarboxylate transporter 3 isoform x6	-3.43	257.35	75.04
LDEC003611-RA	cytoplasmic a3	-3.43	4472.62	1304.62
LDEC001135-RA	26s proteasome non-atpase regulatory subunit 6-like	-3.43	105.18	30.70
LDEC008670-RA	contactin	-3.42	78.93	23.08
LDEC001855-RA	cysteine-rich with egf-like domain protein 2	-3.42	73.91	21.64
LDEC003752-RA	adenylosuccinate synthetase	-3.41	148.20	43.44
LDEC018327-RA	v-type proton atpase subunit e	-3.41	437.12	128.12
LDEC019600-RA	atp-binding cassette sub-family e member 1	-3.41	199.67	58.55
LDEC019322-RA	trifunctional enzyme subunit mitochondrial	-3.41	208.93	61.33
LDEC012245-RA	mitochondrial succinate dehydrogenase cytochrome b subunit	-3.40	124.35	36.52
LDEC001689-RA	tether containing ubx domain for glut4-like	-3.40	105.18	30.90
LDEC016776-RA	serine threonine-protein phosphatase 4 regulatory subunit 1-like isoform x10	-3.40	105.22	30.94
LDEC014877-RA	polyadenylate-binding protein 1-like isoform 1	-3.40	3473.33	1022.10
LDEC022622-RA	pyruvate dehydrogenase e1 component subunit mitochondrial	-3.40	156.08	45.94
LDEC009819-RA	protein phosphatase 1b	-3.39	179.84	53.08
LDEC023525-RA	atp-dependent helicase brm isoform x1	-3.38	143.53	42.46
LDEC014318-RA	alanine--trna cytoplasmic	-3.38	131.70	38.98
LDEC007792-RA	prolow-density lipoprotein receptor-related protein 1-like	-3.38	90.52	26.80
LDEC020605-RA	double-stranded rna-specific editase adar-like	-3.38	76.40	22.62
LDEC003164-RA	serine protease	-3.37	1168.59	346.35
LDEC010954-RA	v-type proton atpase catalytic subunit a	-3.36	1187.29	353.30
LDEC020333-RA	low quality protein: heterogeneous nuclear ribonucleoprotein h-like	-3.35	68.10	20.31
LDEC012678-RA	counting factor associated protein d-like	-3.35	628.95	187.96

LDEC007927-RA	elongation factor 1-alpha	-3.34	83.99	25.12
LDEC012287-RA	cytochrome c oxidase subunit iii	-3.34	3786.83	1132.91
LDEC010103-RA	solute carrier family 26 member 10 isoform x2	-3.34	161.77	48.44
LDEC009281-RA	vesicle-associated membrane protein-associated protein b	-3.34	94.30	28.24
LDEC021975-RA	glutamine synthetase 2 cytoplasmic isoform x2	-3.34	96.56	28.94
LDEC011857-RA	serrate rna effector molecule homolog isoform x2	-3.32	159.07	47.97
LDEC019877-RA	mog interacting and ectopic p-granules protein 1 isoform x2	-3.32	104.26	31.44
LDEC018663-RA	zinc finger protein	-3.31	99.28	30.04
LDEC008711-RA	nadph--cytochrome p450 reductase isoform x2	-3.30	715.70	216.59
LDEC004223-RA	actin-interacting protein 1	-3.30	89.93	27.23
LDEC000763-RA	probable multidrug resistance-associated protein lethal 03659	-3.30	88.44	26.80
LDEC020732-RA	glutathione s-transferase delta	-3.30	75.92	23.01
LDEC005687-RA	selenoprotein s b-like	-3.30	85.62	25.98
LDEC021834-RA	alpha-n-acetylgalactosaminidase-like isoform x2	-3.29	149.41	45.43
LDEC009354-RA	heat shock protein 40	-3.28	128.50	39.14
LDEC016502-RA	40s ribosomal protein s28	-3.28	124.63	37.97
LDEC008396-RA	proteasome subunit alpha type-4	-3.28	89.11	27.15
LDEC011408-RA	eukaryotic translation initiation factor 3 subunit g-like	-3.28	125.25	38.20
LDEC010892-RA	heterogeneous nuclear ribonucleoprotein u-like protein 1	-3.28	225.70	68.86
LDEC003838-RA	glutamine synthetase 2	-3.28	166.41	50.78
LDEC017745-RA	activating transcription factor 7-interacting protein 1 isoform x4	-3.27	142.40	43.51
LDEC014271-RA	activator of 90 kda heat shock protein atpase homolog 1	-3.27	106.79	32.65
LDEC017411-RA	low quality protein: papilin-like	-3.27	167.02	51.13
LDEC003627-RA	bax inhibitor	-3.27	495.52	151.71
LDEC000634-RA	probable saccharopine dehydrogenase	-3.26	91.39	28.01
LDEC000704-RA	26s proteasome non-atpase regulatory subunit 14	-3.26	150.29	46.09
LDEC011360-RA	dimethyladenosine transferase mitochondrial	-3.25	141.09	43.36
LDEC010104-RA	protein prrc2c isoform x6	-3.25	395.23	121.67
LDEC012046-RA	phosphoglycolate phosphatase 2-like	-3.25	104.36	32.15
LDEC018745-RA	transmembrane protein 183	-3.25	126.89	39.10
LDEC007987-RA	aldose 1-epimerase	-3.24	193.04	59.61
LDEC012453-RA	gamma-interferon-inducible lysosomal thiol reductase-like	-3.24	525.13	162.18
LDEC021486-RA	heat shock protein 70	-3.23	201.23	62.30
LDEC017914-RA	isoform a	-3.23	186.84	57.85
LDEC000088-RA	v-type proton atpase 16 kda proteolipid subunit	-3.23	1490.62	461.54
LDEC021164-RA	low quality protein: sarcalumenin-like	-3.23	191.09	59.18
LDEC001849-RA	serine threonine-protein kinase nek2	-3.23	79.08	24.49
LDEC006655-RA	exportin-1	-3.22	75.44	23.40

LDEC001264-RA	apolipoprotein li-ii precursor	-3.22	1082.74	335.80
LDEC006127-RA	basement membrane-specific heparan sulfate proteoglycan core protein	-3.22	71.51	22.19
LDEC007244-RA	timeless-interacting protein	-3.22	77.76	24.14
LDEC016644-RA	vacuolar atp synthase subunit f	-3.22	81.38	25.27
LDEC004611-RA	ebna2 binding protein p100	-3.22	937.55	291.20
LDEC005895-RA	leucine-rich repeat-containing protein ddb_g0290503 isoform x1	-3.22	154.97	48.20
LDEC014762-RA	protein elys-like isoform x3	-3.22	85.77	26.68
LDEC001861-RA	coatomer subunit gamma	-3.21	172.14	53.63
LDEC014363-RA	charged multivesicular body protein 4b	-3.21	86.81	27.07
LDEC015879-RA	protein malvolio-like	-3.19	107.02	33.51
LDEC019162-RA	26s protease regulatory subunit 7	-3.19	154.76	48.51
LDEC009097-RA	protein lsm14 homolog b isoform x3	-3.19	72.59	22.77
LDEC009338-RA	unc93-like protein	-3.19	168.90	53.01
LDEC008016-RA	v-type proton atpase subunit d	-3.19	245.43	77.03
LDEC017112-RA	monocarboxylate transporter 14 isoform x2	-3.19	79.64	25.00
LDEC018561-RA	nucleoprotein tpr isoform x2	-3.18	189.59	59.53
LDEC005838-RA	fatty-acid amide hydrolase 2-like	-3.18	78.55	24.69
LDEC016203-RA	ankyrin-3-like isoform x3	-3.18	85.24	26.80
LDEC016464-RA	small integral membrane protein 14-like isoform x1	-3.18	112.39	35.35
LDEC001233-RA	calcineurin subunit b type 2	-3.17	104.15	32.85
LDEC016077-RA	rna polymerase-associated protein ctr9 homolog	-3.17	110.68	34.92
LDEC004260-RA	kda midgut protein	-3.17	108.94	34.37
LDEC019035-RA	tetratricopeptide repeat protein 14-like protein	-3.16	201.59	63.71
LDEC016018-RA	ubiquilin-1	-3.16	91.02	28.79
LDEC005824-RA	polypeptide n-acetylgalactosaminyltransferase 5 isoform x1	-3.16	168.32	53.32
LDEC012559-RA	eukaryotic peptide chain release factor subunit 1 isoform x1	-3.16	96.89	30.70
LDEC003767-RA	atp-dependent rna helicase belle	-3.15	125.74	39.88
LDEC018195-RA	26s proteasome non-atpase regulatory subunit 2	-3.14	99.72	31.72
LDEC023294-RA	lysosomal aspartic protease	-3.14	1720.30	548.29
LDEC002489-RA	ubiquitin carboxyl-terminal hydrolase 45	-3.14	86.75	27.65
LDEC019594-RA	transmembrane protein 14c	-3.13	73.24	23.40
LDEC020557-RA	muscle m-line assembly protein unc-89-like	-3.13	116.62	37.30
LDEC008586-RA	alpha- partial	-3.12	139.41	44.65
LDEC001757-RA	spectrin beta chain isoform x4	-3.12	1278.41	409.59
LDEC020839-RA	n-acetyllactosaminide beta- -n-acetylglucosaminyltransferase	-3.12	101.27	32.50
LDEC000099-RA	nuclear pore complex protein nup93	-3.11	80.90	25.98
LDEC013405-RA	immediate early response gene 5-like protein	-3.11	87.82	28.20
LDEC007638-RA	cyclin-dependent kinase 12 isoform x3	-3.11	117.70	37.85

LDEC014141-RA	26s protease regulatory subunit 8	-3.11	131.17	42.22
LDEC014316-RA	cullin-associated nedd8-dissociated protein 1	-3.10	177.06	57.07
LDEC005102-RA	probable 4-coumarate-- ligase 1	-3.09	158.21	51.25
LDEC003271-RA	inositol hexakisphosphate and diphosphoinositol-pentakisphosphate kinase 2 isoform x10	-3.08	141.44	45.97
LDEC002342-RA	golgi integral membrane protein 4	-3.08	94.18	30.62
LDEC008263-RA	vacuolar atp synthase subunit s1	-3.07	400.94	130.62
LDEC002389-RA	hexosaminidase 1 isoform x1	-3.07	127.49	41.56
LDEC005101-RA	zinc finger cchc domain-containing protein 7	-3.06	86.25	28.20
LDEC023888-RA	sorbin and sh3 domain-containing protein 1	-3.06	87.02	28.48
LDEC000571-RA	echinoderm microtubule-associated 2 isoform x2	-3.05	100.91	33.08
LDEC005814-RA	cleft lip and palate transmembrane protein 1 homolog	-3.03	193.10	63.67
LDEC007634-RA	major facilitator superfamily domain-containing protein 6 isoform x2	-3.03	135.73	44.76
LDEC020898-RA	porphobilinogen deaminase	-3.03	111.05	36.64
LDEC003275-RA	e3 ubiquitin-protein ligase rnf185-like isoform x2	-3.02	82.23	27.19
LDEC022708-RA	er membrane protein complex subunit 7 homolog	-3.02	104.30	34.49
LDEC005137-RA	rab5 gdp gtp exchange factor	-3.02	101.47	33.59
LDEC012543-RA	isoform a	-3.02	212.26	70.31
LDEC003884-RA	importin subunit alpha	-3.01	157.92	52.42
LDEC005802-RA	adp-ribosylation factor 1	-3.01	315.42	104.88
LDEC019595-RA	serpin peptidase inhibitor 3	-3.01	245.45	81.68
LDEC022497-RA	agap004396-pa-like protein	-3.00	507.87	169.02
LDEC018549-RA	serine arginine repetitive matrix protein 1 isoform x1	-3.00	84.68	28.20
LDEC014306-RA	arginine kinase	-3.00	3771.39	1257.40
LDEC015084-RA	nadh dehydrogenase	-3.00	107.64	35.90
LDEC009300-RA	thioredoxin domain-containing protein 17-like	-2.99	215.37	71.91
LDEC017501-RA	juvenile hormone epoxide hydrolase-like protein 3	-2.99	327.39	109.53
LDEC003432-RA	26s proteasome non-atpase regulatory subunit 12	-2.98	130.40	43.71
LDEC024197-RA	cathepsin b	-2.98	82.30	27.62
LDEC024655-RA	synaptic vesicle glycoprotein 2b-like	-2.98	129.17	43.36
LDEC001314-RA	alanine aminotransferase 2 isoform x2	-2.98	217.02	72.89
LDEC013797-RA	hypothetical protein TcasGA2_TC015166	-2.98	100.55	33.79
LDEC006784-RA	glutamate--cysteine ligase catalytic subunit	-2.97	175.85	59.18
LDEC010054-RA	cleft lip and palate transmembrane protein 1-like protein	-2.97	102.48	34.49
LDEC007307-RA	cytochrome p450-like protein	-2.96	238.92	80.58
LDEC009736-RA	PREDICTED: uncharacterized protein LOC664426 isoform X1	-2.96	128.75	43.51
LDEC002750-RA	translocation protein sec62	-2.96	106.37	35.97
LDEC019874-RA	tropomodulin isoform x1	-2.96	228.72	77.38

LDEC002457-RA	at-rich interactive domain-containing protein 2 isoform x3	-2.95	119.11	40.31
LDEC019816-RA	hydroxyacyl-coenzyme a mitochondrial	-2.95	114.15	38.63
LDEC006412-RA	probable salivary secreted peptide	-2.95	560.74	189.95
LDEC007772-RA	multidrug resistance-associated protein 1 isoform x1	-2.95	100.78	34.14
LDEC019000-RA	insulin-like growth factor 2 mrna-binding protein 1 isoform x4	-2.95	102.42	34.72
LDEC016503-RA	peptidyl-prolyl cis-trans mitochondrial	-2.94	81.52	27.69
LDEC014554-RA	nedd8-conjugating enzyme ubc12	-2.94	93.57	31.83
LDEC009468-RA	low quality protein: titin-like	-2.93	1143.78	390.49
LDEC016301-RA	tppp family protein cg45057	-2.91	98.02	33.71
LDEC012335-RA	er lumen protein retaining receptor	-2.90	195.19	67.42
LDEC020999-RA	b-cell receptor-associated protein 31	-2.89	209.96	72.69
LDEC015706-RA	dipeptidyl peptidase 3 isoform x1	-2.88	182.58	63.32
LDEC013640-RA	nadp-dependent malic enzyme isoform x2	-2.87	301.87	105.27
LDEC002922-RA	probable multidrug resistance-associated protein lethal 03659	-2.87	155.20	54.14
LDEC000118-RA	translocator protein	-2.86	463.01	161.63
LDEC004550-RA	atpase family aaa domain-containing protein 1-a-like	-2.86	93.09	32.50
LDEC005842-RA	PREDICTED: uncharacterized protein CG7065 homolog isoform X2	-2.86	97.88	34.22
LDEC018428-RA	dnaj homolog subfamily a member 2	-2.86	246.62	86.25
LDEC001619-RA	surfeit locus protein 4 homolog	-2.86	278.05	97.30
LDEC008195-RA	flocculation protein flo11 isoform x1	-2.86	278.43	97.50
LDEC002706-RA	protein takeout	-2.85	3401.16	1192.48
LDEC012338-RA	coatomer subunit delta-like	-2.85	165.41	58.00
LDEC019613-RA	ribosome-binding factor mitochondrial	-2.85	84.64	29.69
LDEC019170-RA	3-hydroxyacyl-coa dehyrogenase	-2.85	258.60	90.70
LDEC008792-RA	eif5-mimic protein	-2.85	344.66	120.97
LDEC010726-RA	renin receptor	-2.83	118.16	41.79
LDEC006512-RA	proton-coupled amino acid transporter 4 isoform x3	-2.82	268.33	95.07
LDEC004658-RA	titin isoform x1	-2.81	118.96	42.26
LDEC009380-RA	oxidoreductase glyr1 homolog	-2.81	103.00	36.64
LDEC001393-RA	constitutive coactivator of ppar-gamma-like protein 1 isoform x3	-2.81	140.02	49.84
LDEC013673-RA	probable aconitate mitochondrial	-2.81	1069.54	381.27
LDEC019794-RA	v-type proton atpase 21 kda proteolipid subunit	-2.80	119.59	42.65
LDEC016711-RA	monoacylglycerol lipase abhd12-like	-2.80	161.39	57.61
LDEC015354-RA	aminopeptidase n	-2.78	140.02	50.35
LDEC009837-RA	cationic amino acid transporter	-2.78	92.15	33.16
LDEC001354-RA	transcription factor cp2-like protein 1	-2.78	149.93	53.98
LDEC014392-RA	zinc finger protein 271-like	-2.76	111.07	40.19
LDEC008934-RA	cysteine string protein	-2.76	91.06	33.05
LDEC006100-RA	isoform c	-2.75	772.98	280.81

LDEC003747-RA	isoform h	-2.75	171.37	62.30
LDEC003279-RA	serine hydroxymethyltransferase	-2.75	372.23	135.34
LDEC000568-RA	derlin-1	-2.75	104.70	38.08
LDEC014183-RA	agap007663-pa-like protein	-2.75	107.73	39.22
LDEC011106-RA	electron transfer flavoprotein-ubiquinone mitochondrial	-2.74	148.39	54.10
LDEC001029-RA	nodal modulator 3-like	-2.74	167.44	61.09
LDEC016608-RA	isoform b	-2.71	149.43	55.11
LDEC018340-RA	3-hydroxyacyl- dehydrogenase type-2	-2.71	144.20	53.20
LDEC017184-RA	furin-like protease isoform 1-crr isoform x1	-2.70	184.52	68.24
LDEC020871-RA	flocculation protein flo11 isoform x5	-2.70	169.13	62.58
LDEC011303-RA	probable atp-dependent rna helicase ddx17-like	-2.70	496.58	183.74
LDEC008710-RA	adenosine diphosphatase	-2.70	236.29	87.50
LDEC017615-RA	cytosolic purine 5 -nucleotidase isoform x1	-2.70	228.76	84.84
LDEC019511-RA	integrin beta-ps isoform x2	-2.69	237.87	88.28
LDEC024502-RA	protein yellow-like isoform x2	-2.69	620.91	230.57
LDEC000681-RA	propionyl- carboxylase beta mitochondrial	-2.69	185.11	68.79
LDEC016072-RA	hypothetical protein KGM_03566	-2.68	467.39	174.37
LDEC009230-RA	ubiquitin-conjugating enzyme e2-17 kda	-2.68	112.54	42.03
LDEC019900-RA	camp-dependent protein kinase	-2.68	106.52	39.80
LDEC018181-RA	hypothetical protein YQE_05172, partial	-2.68	9804.66	3664.98
LDEC005498-RA	ribosomal protein ubq l40e	-2.67	806.59	302.41
LDEC013232-RA	maternal protein tudor isoform x1	-2.66	92.15	34.69
LDEC002288-RA	unc93-like protein	-2.65	105.07	39.65
LDEC014482-RA	serine-threonine kinase receptor-associated protein	-2.65	149.22	56.33
LDEC005040-RA	ras-related protein rac1	-2.65	193.27	72.97
LDEC003766-RA	protein tis11	-2.65	220.79	83.47
LDEC008038-RA	multiple coagulation factor deficiency protein 2 homolog	-2.64	205.29	77.73
LDEC001829-RA	v-type proton atpase subunit h isoform x1	-2.63	462.51	175.54
LDEC002764-RA	histone	-2.63	411.34	156.24
LDEC007567-RA	golgin subfamily a member 2-like	-2.62	184.73	70.43
LDEC002527-RA	cg9135-pa	-2.62	240.09	91.79
LDEC004287-RA	cytochrome p450-like protein	-2.61	133.37	51.05
LDEC018210-RA	trna-splicing ligase homolog	-2.60	94.84	36.52
LDEC011709-RA	aldehyde dimeric nadp-preferring-like	-2.59	150.92	58.36
LDEC010000-RA	cathepsin b	-2.58	111.78	43.28
LDEC003768-RA	glycoside hydrolase family 28	-2.57	460.63	179.25
LDEC005789-RA	gamma-interferon-inducible lysosomal thiol reductase-like	-2.57	643.84	250.89
LDEC012728-RA	26s proteasome non-atpase regulatory subunit 1	-2.56	143.66	56.17
LDEC012500-RA	splicing arginine serine-rich 2	-2.56	164.32	64.29

LDEC009342-RA	lipid storage droplets surface-binding protein 1 isoform x1	-2.55	539.73	211.47
LDEC017589-RA	probable 26s proteasome non-atpase regulatory subunit 3	-2.55	158.84	62.30
LDEC004140-RA	juvenile hormone epoxide hydrolase-like protein 2	-2.55	217.53	85.35
LDEC024632-RA	vigilin	-2.55	612.99	240.65
LDEC006326-RA	protein transport protein sec24c-like isoform x1	-2.54	314.61	123.86
LDEC006359-RA	cg1561	-2.54	116.26	45.78
LDEC003301-RA	unc-112-related protein	-2.54	190.86	75.19
LDEC007558-RA	ras-like gtp-binding protein rho1 isoform x2	-2.53	339.41	133.94
LDEC016597-RA	death-associated lim-only protein	-2.53	198.68	78.63
LDEC022337-RA	fatty-acid amide hydrolase 2-like	-2.52	198.22	78.59
LDEC014093-RA	biotin--protein ligase isoform x1	-2.51	122.03	48.67
LDEC014436-RA	signal recognition particle 54 kda protein	-2.50	100.58	40.15
LDEC016277-RA	translational activator gcn1	-2.50	199.04	79.72
LDEC017763-RA	nadh dehydrogenase subunit 1	-2.50	264.60	106.05
LDEC016955-RA	metal transporter cnnm4	-2.48	405.90	163.78
LDEC003573-RA	aminopeptidase -like	-2.46	180.05	73.04
LDEC008777-RA	vigilin	-2.45	417.34	170.07
LDEC000213-RA	synaptic vesicle glycoprotein 2b	-2.45	1178.17	480.64
LDEC004327-RA	nadh dehydrogenase	-2.44	361.66	147.96
LDEC008707-RA	calpain-b isoform x1	-2.44	113.50	46.48
LDEC020409-RA	actin-related protein 2 isoform x2	-2.44	127.70	52.34
LDEC000249-RA	2-oxoglutarate mitochondrial-like isoform x1	-2.43	291.44	119.72
LDEC020034-RA	pescadillo homolog	-2.43	106.03	43.71
LDEC018424-RA	protein homolog isoform x1	-2.38	494.41	207.92
LDEC005482-RA	oxysterol-binding protein 1	-2.36	116.45	49.37
LDEC015116-RA	eukaryotic translation initiation factor 4 gamma 2 isoform x2	-2.36	453.04	192.26
LDEC014874-RA	juvenile hormone esterase	-2.35	125.65	53.55
LDEC020709-RA	plasma membrane calcium-transporting atpase 2 isoform x1	-2.35	251.99	107.46
LDEC005740-RA	lissencephaly-1 homolog	-2.34	146.52	62.54
LDEC006636-RA	nadh dehydrogenase subunit 4	-2.33	609.79	261.16
LDEC007602-RA	stress-associated endoplasmic reticulum protein 2	-2.33	264.64	113.43
LDEC004033-RA	transmembrane emp24 domain-containing protein eca	-2.32	123.66	53.24
LDEC018153-RA	dehydrogenase reductase sdr family member 11-like	-2.32	197.32	85.07
LDEC014170-RA	hypothetical protein D910_06773	-2.32	193.56	83.55
LDEC016869-RA	glutathione s-transferase-like	-2.31	333.83	144.37
LDEC003250-RA	hypoxia-inducible factor 1 alpha	-2.30	206.30	89.88
LDEC016483-RA	transport and golgi organization protein 1 isoform x1	-2.29	140.04	61.29
LDEC002322-RA	dyslexia-associated protein kiaa0319-like protein	-2.27	222.98	98.16

LDEC000892-RA	alpha- partial	-2.27	700.55	309.24
LDEC010549-RA	luciferase homolog	-2.26	256.26	113.28
LDEC011741-RA	tpa_inf: hdc16691	-2.26	195.90	86.75
LDEC012392-RA	14-3-3 protein zeta	-2.25	245.09	108.86
LDEC013437-RA	rho gdp-dissociation inhibitor 2	-2.24	216.00	96.40
LDEC000265-RA	lysosomal aspartic protease	-2.24	186.76	83.51
LDEC010680-RA	ras-related protein rab-11a	-2.23	170.47	76.44
LDEC019806-RA	protein gpr107	-2.23	129.40	58.04
LDEC007044-RA	dnaj homolog subfamily c member 7	-2.23	267.15	119.96
LDEC023354-RA	cathepsin b	-2.21	129.50	58.63
LDEC002623-RA	26s proteasome non-atpase regulatory subunit 2	-2.19	177.69	81.09
LDEC002643-RA	microtubule-associated protein futsch isoform x1	-2.18	185.49	85.15
LDEC014140-RA	kynurenine--oxoglutarate transaminase 3 isoform x2	-2.18	438.00	201.32
LDEC005030-RA	e3 sumo-protein ligase 2	-2.18	183.42	84.33
LDEC015168-RA	glycoside hydrolase family 28	-2.17	1466.11	675.36
LDEC022002-RA	defensin precursor	-2.17	135.82	62.65
LDEC011196-RA	transcriptional activator protein pur-alpha isoform x1	-2.16	153.76	71.09
LDEC001566-RA	innexin 2	-2.16	202.40	93.71
LDEC021702-RA	protein slowmo	-2.14	146.13	68.43
LDEC003376-RA	integral membrane protein 2c-like isoform x1	-2.13	347.23	162.88
LDEC011371-RA	PREDICTED: LOW QUALITY PROTEIN: uncharacterized protein LOC101888582	-2.13	943.18	442.91
LDEC006153-RA	serine threonine-protein phosphatase 2a catalytic subunit alpha isoform	-2.12	270.33	127.34
LDEC007911-RA	glycoside hydrolase family 45 protein	-2.11	287.25	135.89
LDEC011491-RA	death-associated protein 1	-2.11	137.38	65.04
LDEC001662-RA	short-chain specific acyl- mitochondrial	-2.11	227.71	107.89
LDEC021334-RA	calmodulin	-2.10	235.18	111.83
LDEC022265-RA	an1-type zinc finger protein 6 isoform x3	-2.10	194.35	92.50
LDEC016786-RA	beta- -glucanase	-2.09	225.62	107.73
LDEC010053-RA	ras-related protein rab-1a	-2.09	269.43	129.10
LDEC009333-RA	isocitrate dehydrogenase	-2.08	187.18	89.96
LDEC009270-RA	pollen-specific leucine-rich repeat extensin-like protein 1	-2.07	199.04	96.09
LDEC013225-RA	calsyntenin-1	-2.07	282.67	136.52
LDEC003369-RA	translocon-associated protein subunit delta	-2.06	195.75	95.15
LDEC000004-RA	rna polymerase ii largest subunit	-2.05	257.89	125.58
LDEC008409-RA	solute carrier family 25 member 35	-2.05	351.71	171.75
LDEC020644-RA	trypsin-like serine proteinase	-2.03	166.29	81.87
LDEC007376-RA	cg3595	-2.01	159.66	79.33
LDEC020927-RA	ankyrin-3-like isoform x2	-2.01	174.42	86.95
LDEC018999-RA	transmembrane protein 214-a isoform	-2.00	157.32	78.71

	x2			
LDEC003068-RA	glutamine--fructose-6-phosphate aminotransferase	-1.99	237.88	119.29
LDEC010227-RA	lysosomal aspartic protease	-1.96	1440.12	734.77
LDEC004844-RA	neuroplastin isoform x1	-1.94	438.82	225.93
LDEC014622-RA	atp-dependent rna helicase p62	-1.94	1331.80	687.27
LDEC018226-RA	maternal protein exuperantia-like	-1.93	212.69	110.42
LDEC002534-RA	purine nucleoside phosphorylase-like isoform x2	-1.92	214.91	111.67
LDEC014729-RA	protein tyrosine phosphatase type iva 1	-1.91	323.29	168.86
LDEC010470-RA	cytochrome b5-related	-1.91	194.37	101.79
LDEC007315-RA	ribonuclease x25	-1.88	282.63	150.46
LDEC023707-RA	chitin-binding protein	-1.88	2757.49	1468.37
LDEC014179-RA	glucose dehydrogenase	-1.87	278.18	148.78
LDEC017711-RA	ribosome-binding protein 1-like isoform x1	-1.86	261.80	140.77
LDEC005105-RA	ornithine decarboxylase antizyme partial	-1.86	939.31	505.72
LDEC006670-RA	chondroitin proteoglycan-2	-1.82	220.02	120.70
LDEC019491-RA	cathepsin l	-1.82	266.24	146.44
LDEC016136-RA	protein hu-li tai shao isoform x2	-1.80	244.69	136.24
LDEC018787-RA	clathrin heavy chain	-1.79	492.52	275.18
LDEC015001-RA	nadh dehydrogenase subunit 4	-1.77	337.11	190.15
LDEC019789-RA	xanthine dehydrogenase isoform x1	-1.77	282.09	159.29
LDEC003231-RA	reticulon-1-a-like isoform x5	-1.73	408.78	236.28
LDEC018943-RA	membrane-associated progesterone receptor component 1-like	-1.72	235.95	136.99
LDEC006414-RA	myrosinase 1-like	-1.71	900.53	525.68
LDEC007304-RA	craniofacial development protein 2-like	-1.70	303.67	178.19
LDEC002144-RA	nadh dehydrogenase subunit 5	-1.64	707.33	431.19
LDEC004265-RA	205 kda microtubule-associated	-1.63	359.24	219.91
LDEC018398-RA	agap002583-pa-like protein	-1.61	307.73	191.20
LDEC015589-RA	ferritin 2	-1.58	823.03	521.54
LDEC010353-RA	abhydrolase domain-containing protein 3-like	-1.56	949.81	609.31
LDEC000710-RA	glycoside hydrolase family 45 protein	-1.51	427.67	283.23
LDEC012017-RA	gamma-interferon-inducible lysosomal thiol reductase-like	-1.50	1049.76	698.68
LDEC024276-RA	glycoside hydrolase family 1	-1.33	1119.65	844.80
LDEC008991-RA	digestive cysteine proteinase intestain	1.10	15032.00	16532.98
LDEC001704-RA	endo-beta- -glucanase	1.38	499.74	688.76
LDEC015309-RA	ferritin subunit	1.43	852.58	1218.10
LDEC010714-RA	probable salivary secreted peptide	1.43	374.95	537.59
LDEC013066-RA	endo-beta- -glucanase	1.48	1485.83	2200.67
LDEC014014-RA	atp-binding cassette sub-family g member 4 isoform x2	1.52	400.71	607.20
LDEC010412-RA	muscle-specific protein 300	1.52	490.01	744.46
LDEC023489-RA	glycoside hydrolase family 31	1.58	256.09	404.43

LDEC017588-RA	fatty acid-binding protein 12-like	1.58	848.88	1344.62
LDEC008000-RA	cytochrome p450 9z4	1.59	394.02	628.17
LDEC004940-RA	glycoside hydrolase family protein 48	1.62	259.88	421.93
LDEC016916-RA	cathepsin l-like proteinase	1.68	661.95	1114.05
LDEC004989-RA	cathepsin l	1.76	133.54	234.56
LDEC021033-RA	---NA---	1.76	149.01	262.06
LDEC015237-RA	chitin binding peritrophin-	1.77	148.07	262.06
LDEC013260-RA	cytochrome p450 cyp6bk17	1.83	115.61	212.14
LDEC014996-RA	glycosyl hydrolase	1.84	268.60	494.16
LDEC014667-RA	lysosomal aspartic protease	1.85	951.98	1765.19
LDEC020734-RA	-trehalose-phosphate synthase	1.92	119.50	229.95
LDEC008992-RA	cytokine receptor	1.94	120.82	234.44
LDEC000716-RA	vanin-like protein 1	1.94	190.17	369.47
LDEC002669-RA	leucine-rich repeat-containing protein ddb_g0290503-like isoform x1	1.96	97.27	190.65
LDEC014283-RA	rna-directed dna polymerase from mobile element partial	1.96	95.58	187.53
LDEC000854-RA	digestive cysteine protease intestain	2.00	140.21	279.75
LDEC021028-RA	endo-beta- -glucanase	2.00	87.78	175.34
LDEC002229-RA	esterase	2.00	113.65	227.45
LDEC003236-RA	spectrin alpha non-erythrocytic 1 isoform x4	2.00	477.90	958.04
LDEC000024-RA	23 kda integral membrane	2.01	183.23	368.42
LDEC000679-RA	myosin 1a isoform x1	2.01	79.89	160.73
LDEC001669-RA	ferritin 2	2.01	886.15	1785.54
LDEC012691-RA	mariner transposase	2.09	68.60	143.47
LDEC010623-RA	glycoside hydrolase family protein 48	2.12	3410.19	7245.47
LDEC016019-RA	low quality protein: brachyurin-like	2.14	137.34	293.42
LDEC003810-RA	fibronectin type-iii domain-containing protein 3a isoform x2	2.15	173.42	372.95
LDEC007406-RA	isoform b	2.18	102.37	222.68
LDEC008329-RA	peritrophic matrix protein 1-c precursor	2.18	1124.40	2447.61
LDEC006791-RA	poly -binding protein 3 isoform x1	2.18	69.41	151.20
LDEC010146-RA	gamma-interferon-inducible lysosomal thiol reductase-like	2.21	189.23	417.52
LDEC008922-RA	nedd4-binding protein 2	2.22	165.87	367.95
LDEC005886-RA	probable protein phosphatase 2c	2.23	112.39	250.61
LDEC011063-RA	serine protease	2.24	121.53	271.86
LDEC009811-RA	nose resistant to fluoxetine protein 6-like	2.26	72.66	164.52
LDEC004028-RA	aminopeptidase n	2.27	151.48	343.42
LDEC003629-RA	multidrug resistance-associated protein 4-like	2.27	55.48	125.81
LDEC012029-RA	rna polymerase ii elongation factor ell-like	2.28	83.80	191.20
LDEC024010-RA	cadherin-like protein	2.28	53.89	123.00
LDEC002603-RA	protein btg1-like	2.35	78.89	185.11
LDEC011561-RA	serine protease p98	2.36	64.65	152.34

LDEC007972-RA	class b secretin-like g-protein coupled receptor	2.37	60.98	144.76
LDEC020050-RA	fatty acid-binding	2.38	65.90	156.75
LDEC012860-RA	glycosyl hydrolase	2.42	386.93	936.36
LDEC012186-RA	creb-regulated transcription coactivator 1-like isoform x1	2.43	54.25	131.83
LDEC006633-RA	cathepsin b	2.44	337.64	823.01
LDEC008970-RA	cathepsin b	2.44	584.90	1426.84
LDEC017246-RA	PREDICTED: uncharacterized protein LOC659539	2.44	107.41	262.41
LDEC002684-RA	protein gawky isoform x1	2.47	47.89	118.16
LDEC015379-RA	agap011476-pa-like protein	2.47	66.09	163.16
LDEC001534-RA	probable atp-dependent rna helicase dhx35	2.48	67.80	167.96
LDEC006288-RA	vanin-like protein 1	2.49	118.52	295.22
LDEC011092-RA	ctl-like protein 2	2.49	42.58	106.17
LDEC008020-RA	15-hydroxyprostaglandin dehydrogenase	2.52	54.75	137.84
LDEC008075-RA	suppressor of presenilin protein 4 isoform x4	2.52	84.83	213.62
LDEC012816-RA	digestive cysteine protease intestain	2.54	91.85	232.84
LDEC001556-RA	glycoside hydrolase family 28 protein	2.54	934.60	2377.19
LDEC011567-RA	race-specific elicitor a4-like	2.56	38.06	97.30
LDEC013814-RA	microtubule-associated protein futsch isoform x1	2.57	54.67	140.62
LDEC022369-RA	PREDICTED: uncharacterized protein LOC105199588	2.60	48.94	127.22
LDEC016695-RA	insulin-like growth factor-binding protein complex acid labile subunit	2.64	98.97	260.81
LDEC007280-RA	cytochrome p450 6a2	2.64	319.63	843.08
LDEC008876-RA	low quality protein: sialin	2.64	46.05	121.56
LDEC023142-RA	reverse transcriptase	2.64	209.54	553.76
LDEC014455-RA	sh3 domain-containing kinase-binding protein 1 isoform x1	2.65	71.23	188.78
LDEC017005-RA	prophenoloxidase iv	2.65	170.34	451.58
LDEC010803-RA	atp-binding cassette sub-family g member 1	2.66	36.52	97.10
LDEC007327-RA	cytochrome p450 6a2	2.67	538.45	1435.05
LDEC016421-RA	probable deoxyhypusine synthase	2.68	57.68	154.84
LDEC014781-RA	f-box lrr-repeat protein 6 isoform x1	2.70	34.78	93.94
LDEC010725-RA	protein beta isoform-like isoform x1	2.71	39.65	107.26
LDEC017742-RA	e3 ubiquitin-protein ligase huwe1	2.71	82.42	223.27
LDEC007060-RA	---NA---	2.71	61.44	166.71
LDEC000818-RA	cathepsin l precursor	2.72	158.24	430.64
LDEC017784-RA	tom1-like protein 2 isoform x1	2.74	68.12	186.36
LDEC010493-RA	chitin deacetylase 1	2.74	1891.54	5177.40
LDEC014703-RA	diuretic hormone receptor-like	2.77	38.02	105.27
LDEC001256-RA	guanine nucleotide-releasing factor 2 isoform x5	2.77	42.41	117.65
LDEC002116-RA	phospholipase a2 inhibitor-like	2.79	31.98	89.14
LDEC010973-RA	PREDICTED: uncharacterized protein	2.81	47.83	134.41

	LOC655909 isoform X2			
LDEC022889-RA	xaa-pro aminopeptidase 1	2.82	49.61	139.99
LDEC015502-RA	inactive pancreatic lipase-related protein 1	2.84	161.02	456.66
LDEC003308-RA	agap010471-pa-like protein	2.84	41.51	118.08
LDEC005930-RA	leucine-rich repeat-containing protein 24-like isoform x1	2.85	53.48	152.69
LDEC016932-RA	fat-like cadherin-related tumor suppressor homolog	2.86	47.93	136.91
LDEC009116-RA	u-box domain containing 5	2.88	172.88	497.44
LDEC002427-RA	atp-binding cassette sub-family a member 3-like	2.89	124.25	359.01
LDEC001525-RA	PREDICTED: septin-2 [Tribolium castaneum]	2.89	30.93	89.41
LDEC019515-RA	juvenile hormone esterase isoform a	2.91	153.86	447.17
LDEC021034-RA	protein unc-79 homolog	2.92	33.92	99.06
LDEC022023-RA	e3 ubiquitin-protein ligase huwe1	2.95	92.82	274.21
LDEC009638-RA	ubiquitin carboxyl-terminal hydrolase 24-like isoform x1	2.96	73.93	218.66
LDEC016472-RA	apoptotic protease-activating factor 1	2.96	40.51	119.99
LDEC002037-RA	glycoside hydrolase family 1	2.97	156.65	464.66
LDEC016508-RA	facilitated trehalose transporter tret1-2 homolog	2.97	103.61	307.91
LDEC023986-RA	prickle-like protein 2	2.98	31.45	93.63
LDEC008072-RA	PREDICTED: uncharacterized protein LOC659539	2.98	28.53	85.15
LDEC015743-RA	f-box lrr-repeat protein 14	2.99	27.71	82.89
LDEC022367-RA	leucine-rich repeat neuronal protein 1-like	3.00	59.40	178.00
LDEC005001-RA	histone-lysine n-methyltransferase setmar-like	3.01	38.13	114.68
LDEC016746-RA	esterase	3.02	129.73	391.47
LDEC008434-RA	PREDICTED: uncharacterized protein LOC660742	3.04	41.39	125.70
LDEC005119-RA	inactive pancreatic lipase-related protein 1	3.05	52.77	160.73
LDEC001589-RA	glycosyl hydrolase	3.05	436.16	1329.86
LDEC004426-RA	dentin sialophosphoprotein isoform x3	3.05	307.14	938.00
LDEC010935-RA	arf-gap with rho-gap ank repeat and ph domain-containing protein 1	3.06	26.66	81.60
LDEC005720-RA	rho gtpase activating protein 15	3.07	24.87	76.48
LDEC002791-RA	atp-binding cassette sub-family g member 4	3.10	151.88	470.60
LDEC005869-RA	leucine-rich transmembrane protein	3.10	29.40	91.25
LDEC008727-RA	glycosyl hydrolase	3.12	29.57	92.34
LDEC003010-RA	tyrosine-protein kinase	3.13	39.23	122.61
LDEC007471-RA	pleckstrin-like proteiny domain-containing family m member 2	3.14	23.45	73.55
LDEC007042-RA	atp-binding cassette sub-family a member 3-like isoform x4	3.14	111.87	351.08
LDEC005266-RA	glycoside hydrolase family 28 protein	3.16	383.04	1208.57
LDEC016781-RA	tbc1 domain family member 9	3.16	31.94	100.82
LDEC002598-RA	hypothetical protein TcasGA2_TC001060	3.16	101.54	320.69
LDEC012734-RA	glycoside hydrolase family 45 protein	3.18	219.49	698.87

LDEC019116-RA	deoxyribonuclease tatdn1	3.20	40.51	129.52
LDEC012378-RA	u2 small nuclear ribonucleoprotein a	3.21	23.67	75.86
LDEC009512-RA	ccaat enhancer-binding	3.22	48.25	155.30
LDEC000725-RA	protein mothers against dpp	3.23	43.92	141.87
LDEC003347-RA	ras-related and estrogen-regulated growth inhibitor	3.24	69.94	226.63
LDEC000957-RA	high affinity cationic amino acid transporter 1-like	3.24	37.67	122.18
LDEC002170-RA	PREDICTED: uncharacterized protein LOC657683	3.25	121.74	395.61
LDEC019265-RA	nadh dehydrogenase	3.25	22.36	72.77
LDEC004438-RA	low affinity cationic amino acid transporter 2 isoform x1	3.27	31.31	102.38
LDEC004410-RA	protein argonaute-2-like	3.27	72.72	237.92
LDEC015958-RA	mothers against decapentaplegic homolog 4 isoform x3	3.28	45.47	149.02
LDEC000639-RA	protein cereblon-like	3.29	37.08	121.99
LDEC010341-RA	chitin synthase 2	3.29	40.89	134.72
LDEC010802-RA	potentail helicase mov-10	3.30	22.06	72.73
LDEC014411-RA	PREDICTED: uncharacterized protein CG10915 isoform X1	3.30	32.04	105.74
LDEC000825-RA	2-hydroxyacylsphingosine 1-beta-galactosyltransferase-like	3.36	28.09	94.45
LDEC012696-RA	juvenile hormone esterase isoform a	3.41	41.35	140.81
LDEC009913-RA	multiple epidermal growth factor-like domains protein 11	3.41	40.66	138.51
LDEC002217-RA	attractin-like protein 1	3.41	99.84	340.10
LDEC007679-RA	e3 ubiquitin-protein ligase ubr4 isoform x2	3.46	23.99	82.96
LDEC005260-RA	glycosyl hydrolase	3.46	32.60	112.77
LDEC010246-RA	tgf-beta receptor type-1 isoform x2	3.46	68.95	238.74
LDEC002002-RA	zinc finger protein 264-like isoform x1	3.47	43.65	151.67
LDEC017891-RA	von willebrand factor type egf and pentraxin domain-containing protein 1-like	3.48	18.84	65.50
LDEC009212-RA	PREDICTED: uncharacterized protein LOC655909 isoform X2	3.49	78.07	272.21
LDEC011291-RA	beta-mannosidase isoform x2	3.49	46.12	161.01
LDEC007682-RA	flj37770-like protein	3.50	20.64	72.22
LDEC012948-RA	digestive cysteine protease intestain	3.52	1338.29	4713.60
LDEC002998-RA	---NA---	3.54	23.61	83.63
LDEC015220-RA	aromatic-l-amino-acid decarboxylase-like	3.54	90.93	322.33
LDEC006350-RA	hypothetical protein D910_02874	3.57	34.51	123.20
LDEC002538-RA	serine threonine protein kinase	3.60	75.65	272.06
LDEC004809-RA	de cadherin-like protein	3.61	76.31	275.49
LDEC007246-RA	f-box only protein 6	3.62	22.29	80.62
LDEC010280-RA	venom serine carboxypeptidase-like	3.64	31.41	114.29
LDEC005919-RA	PREDICTED: uncharacterized protein LOC656855 isoform X3	3.64	458.14	1667.89
LDEC006184-RA	unc93-like protein mfsd11	3.65	24.64	89.96
LDEC016697-RA	chromatin assembly factor 1 subunit b	3.66	18.30	66.95

LDEC003624-RA	tyrosine-protein phosphatase non-receptor type 1 isoform x1	3.69	24.09	88.78
LDEC020554-RA	zinc finger protein partial	3.70	47.16	174.52
LDEC015043-RA	atp synthase mitochondrial f1 complex assembly factor 2	3.71	41.16	152.53
LDEC011669-RA	flocculation protein flo11	3.71	49.29	182.76
LDEC020388-RA	btb poz domain-containing protein 7 isoform x1	3.71	17.63	65.47
LDEC004564-RA	glycosyl hydrolase	3.73	222.08	828.48
LDEC001177-RA	PREDICTED: uncharacterized protein LOC659539	3.74	30.39	113.67
LDEC022212-RA	zinc finger mym-type protein 1	3.74	21.73	81.29
LDEC003834-RA	isoform b	3.75	19.51	73.16
LDEC002648-RA	PREDICTED: uncharacterized protein KIAA1109	3.76	23.34	87.77
LDEC009221-RA	protein phosphatase 1 regulatory subunit sds22	3.80	17.42	66.21
LDEC022473-RA	zinc finger protein 704-like	3.80	63.93	243.15
LDEC015409-RA	rhomboid-related protein 3-like isoform 2	3.81	15.96	60.86
LDEC013904-RA	udp-glucuronosyltransferase 2b7	3.83	72.95	279.60
LDEC017604-RA	PREDICTED: hypothetical protein LOC100748865	3.83	53.31	204.44
LDEC013013-RA	cadherin 1	3.86	49.32	190.46
LDEC005110-RA	glycosyl hydrolase	3.88	182.37	707.50
LDEC010372-RA	protein toll	3.90	32.71	127.42
LDEC009557-RA	hypothetical protein	3.90	16.48	64.22
LDEC001320-RA	ddb1- and cul4-associated factor 6-partial	3.99	20.62	82.22
LDEC014972-RA	grip and coiled-coil domain-containing protein 2-like	3.99	41.39	165.30
LDEC017851-RA	digestive cysteine proteinase intestain	4.03	2399.21	9661.99
LDEC004364-RA	motile sperm domain-containing protein 2	4.05	99.24	402.09
LDEC002679-RA	protein tamozhennic	4.06	29.74	120.62
LDEC007262-RA	extracellular domains-containing protein cg31004-like	4.07	126.61	515.25
LDEC012551-RA	solute carrier family 35 member c2	4.07	51.07	208.08
LDEC014768-RA	cysteine and histidine-rich protein 1 homolog	4.10	17.69	72.50
LDEC011854-RA	rna-directed dna polymerase from mobile element jockey-like	4.12	58.96	243.00
LDEC002670-RA	viral iap-associated factor homolog	4.13	73.12	302.29
LDEC011110-RA	chitin synthase	4.15	27.57	114.56
LDEC009197-RA	comm domain-containing protein 3	4.18	13.26	55.43
LDEC018635-RA	glycosyl hydrolase	4.18	20.24	84.64
LDEC003449-RA	rna-directed dna polymerase from mobile element jockey-like	4.20	55.05	231.08
LDEC002736-RA	ankyrin repeat and sam domain-containing protein 1a-like	4.20	18.86	79.25
LDEC004448-RA	lipopolysaccharide-induced tumor necrosis factor-alpha factor homolog	4.20	16.86	70.89
LDEC001941-RA	hypothetical protein YQE_11513, partial	4.24	38.73	164.33
LDEC002406-RA	metallophosphoesterase 1	4.25	22.11	93.98

LDEC003067-RA	scp-related protein	4.27	32.98	140.89
LDEC008880-RA	apoptosis 1 inhibitor	4.28	213.80	915.62
LDEC017099-RA	proton-coupled amino acid transporter 4	4.30	30.10	129.33
LDEC000848-RA	equilibrative nucleoside transporter 3	4.32	21.08	91.13
LDEC015965-RA	atp-binding cassette sub-family a member 3 isoform x1	4.32	41.72	180.42
LDEC023405-RA	PREDICTED: beta-mannosidase-like	4.34	88.97	386.04
LDEC016006-RA	mitogen-activated protein kinase 1	4.40	114.99	506.38
LDEC019805-RA	neutral alpha-glucosidase ab	4.42	39.70	175.62
LDEC015740-RA	fragile x mental retardation syndrome-related protein 1 isoform x5	4.44	12.00	53.32
LDEC019740-RA	gaba-gated ion channel	4.45	67.18	299.28
LDEC023744-RA	ankyrin unc44	4.47	37.96	169.84
LDEC018923-RA	digestive cysteine protease intestain	4.48	757.65	3391.40
LDEC013528-RA	probable phospholipid-transporting atpase ia isoform x4	4.48	20.56	92.10
LDEC003824-RA	glycosyl hydrolase	4.53	23.34	105.66
LDEC019424-RA	ankyrin unc44	4.54	14.81	67.22
LDEC000777-RA	ankyrin unc44	4.54	34.13	155.07
LDEC014682-RA	cysteine--trna cytoplasmic-like	4.56	51.26	233.62
LDEC010101-RA	cytochrome p450 6k1	4.58	11.84	54.22
LDEC013602-RA	transposable element tc3 transposase	4.59	72.84	334.59
LDEC015609-RA	receptor-binding cancer antigen expressed on o cells	4.60	20.18	92.77
LDEC009330-RA	glycosyl hydrolase	4.61	97.40	448.73
LDEC020830-RA	hypothetical protein D910_10956	4.61	52.62	242.45
LDEC009556-RA	fatty acid-biding protein	4.62	48.12	222.29
LDEC004447-RA	sarcosine mitochondrial	4.63	20.56	95.27
LDEC018182-RA	inducible metalloproteinase inhibitor	4.64	26.71	124.02
LDEC020309-RA	venom dipeptidyl peptidase 4	4.64	17.46	81.05
LDEC004071-RA	protein peter pan	4.64	26.67	123.90
LDEC007576-RA	glycoside hydrolase family 1	4.65	22.31	103.78
LDEC014508-RA	chondroitin proteoglycan-2-like	4.67	326.24	1523.71
LDEC017465-RA	ubiquitin carboxyl-terminal hydrolase 2-like isoform x2	4.68	14.87	69.57
LDEC009123-RA	zinc finger fyve domain-containing protein 26	4.69	18.66	87.46
LDEC003902-RA	digestive cysteine protease intestain	4.71	676.04	3186.99
LDEC015125-RA	ectopic p granules protein 5 homolog	4.74	15.00	71.09
LDEC008667-RA	sortilin-related receptor-like	4.74	153.70	728.75
LDEC011015-RA	agap007060-pa-like protein	4.76	10.71	50.97
LDEC003042-RA	isoform b	4.76	17.48	83.24
LDEC008270-RA	laccase isoform f	4.80	30.47	146.28
LDEC014668-RA	atp-binding cassette sub-family g member 4	4.85	75.21	364.59
LDEC006881-RA	eukaryotic translation initiation factor 4 gamma 3-like isoform x3	4.87	10.10	49.18

LDEC007407-RA	---NA---	4.88	20.83	101.60
LDEC016910-RA	e3 ubiquitin-protein ligase hecw2 isoform x3	4.88	10.67	52.11
LDEC018304-RA	protein phosphatase-7	4.91	13.34	65.50
LDEC010921-RA	myosin heavy chain kinase d	4.94	11.44	56.48
LDEC010343-RA	ly-6 neurotoxin-related protein	4.99	26.33	131.28
LDEC016734-RA	interference hedgehog-like isoform x1	4.99	50.19	250.26
LDEC020736-RA	ankyrin repeat and sterile alpha motif domain-containing protein 1b	5.04	13.93	70.19
LDEC013243-RA	15-hydroxyprostaglandin dehydrogenase	5.05	13.76	69.45
LDEC013358-RA	lian-aa1 retrotransposon protein	5.13	24.83	127.42
LDEC005677-RA	integrin alpha-ps3-like	5.14	12.07	62.03
LDEC014836-RA	cardioacceleratory peptide receptor-like isoform x1	5.20	17.28	89.80
LDEC000711-RA	ring finger protein nhl-1-like	5.20	27.98	145.46
LDEC009339-RA	juvenile hormone acid o-methyltransferase	5.24	12.49	65.47
LDEC000277-RA	glycoside hydrolase family 1	5.26	25.16	132.45
LDEC012364-RA	insulinprotein enhancer protein isl	5.28	13.64	72.11
LDEC022581-RA	phosphatidate phosphatase isoform x2	5.29	9.18	48.51
LDEC006154-RA	chitin synthase 2	5.31	44.30	235.14
LDEC000580-RA	digestive cysteine protease intestain	5.35	109.21	584.58
LDEC003356-RA	cytochrome p450 6a2	5.36	8.74	46.87
LDEC001063-RA	coiled-coil domain-containing protein lobo homolog	5.38	17.55	94.37
LDEC018500-RA	fact complex subunit spt16-like	5.41	111.89	604.78
LDEC023272-RA	pyruvate mitochondrial isoform x1	5.41	84.79	458.88
LDEC009044-RA	syntaxin-binding protein 5 isoform x3	5.43	45.80	248.78
LDEC013335-RA	leucine-rich repeats and immunoglobulin-like domains protein 2 isoform x2	5.48	18.74	102.69
LDEC012034-RA	protein tprxl isoform x2	5.48	10.58	57.97
LDEC023304-RA	hook protein	5.48	14.31	78.51
LDEC007956-RA	pre-mrna-splicing factor syf2	5.51	19.60	108.00
LDEC012032-RA	maguk p55 subfamily member 5-a isoform x1	5.52	11.34	62.58
LDEC007516-RA	4-coumarate-- ligase 1-like	5.53	13.38	73.94
LDEC016666-RA	atm interactor	5.53	10.90	60.35
LDEC007269-RA	protocadherin beta-15-like	5.56	59.12	328.73
LDEC021846-RA	protein turtle isoform x1	5.59	71.53	400.14
LDEC002092-RA	low quality protein: metabotropic glutamate receptor- partial	5.60	9.10	51.01
LDEC000843-RA	lap4 protein (scribble protein) (smell-impaired protein)	5.62	29.45	165.54
LDEC005931-RA	e3 ubiquitin-protein ligase herc2-like	5.73	18.38	105.39
LDEC014577-RA	patatin-like phospholipase domain-containing protein 2-like	5.75	56.97	327.64
LDEC017665-RA	regucalcin-like isoform x2	5.76	66.36	381.97
LDEC020550-RA	esterase	5.76	8.22	47.38

LDEC005098-RA	rhomboid-related protein 2	5.79	8.37	48.47
LDEC018200-RA	fatty acid hydroxylase domain-containing protein 2 isoform x2	5.81	8.34	48.44
LDEC016761-RA	helicase mov-10-	5.85	39.99	233.97
LDEC001388-RA	reverse partial	5.86	33.98	199.01
LDEC013938-RA	xk-related protein 6-like	5.89	62.87	370.02
LDEC007273-RA	lysosomal aspartic protease	5.99	82.94	497.12
LDEC007610-RA	kv channel-interacting protein 1	5.99	14.54	87.18
LDEC007878-RA	isoform a	6.03	8.12	48.98
LDEC014909-RA	esterase	6.08	8.51	51.76
LDEC008675-RA	---NA---	6.08	7.51	45.70
LDEC006163-RA	spatacsin isoform x1	6.13	17.25	105.74
LDEC014103-RA	alpha-tocopherol transfer protein	6.17	12.69	78.24
LDEC006443-RA	transcription initiation factor tfiid subunit 2	6.17	7.97	49.22
LDEC000637-RA	hypothetical protein YQE_07193, partial	6.25	16.48	102.92
LDEC012313-RA	agap013097-pa-like protein	6.30	9.20	57.97
LDEC019994-RA	facilitated trehalose transporter tret1-like	6.36	1438.15	9145.45
LDEC010574-RA	digestive cysteine protease intestain	6.39	130.88	836.01
LDEC019753-RA	salivary glue protein sgs-3-like	6.42	10.33	66.29
LDEC000494-RA	peritrophic membrane chitin binding protein	6.46	9.41	60.78
LDEC018916-RA	ribosomal protein s5	6.50	11.27	73.28
LDEC005623-RA	neutral alpha-glucosidase ab-like	6.51	18.32	119.21
LDEC007358-RA	aquaporin aqpcic	6.55	15.85	103.78
LDEC016420-RA	salivary protein mys2 precursor	6.59	11.44	75.43
LDEC004855-RA	timeless isoform b	6.62	14.31	94.72
LDEC021559-RA	lipase 3	6.69	35.49	237.25
LDEC018396-RA	inositol hexakisphosphate kinase 1 isoform x2	6.76	9.64	65.15
LDEC002565-RA	lysosomal aspartic protease	6.79	45.47	308.62
LDEC001806-RA	beta-galactosidase-1-like protein 2	6.86	80.69	553.45
LDEC022387-RA	centrosomal protein of 290 kda-like isoform x1	6.88	27.63	190.11
LDEC010110-RA	neutral alpha-glucosidase ab	6.95	150.60	1046.67
LDEC012960-RA	polyadenylation factor	7.04	8.76	61.64
LDEC001932-RA	leucine-rich transmembrane	7.04	11.69	82.34
LDEC004876-RA	glycoside hydrolase family 28 protein	7.10	56.05	397.91
LDEC008898-RA	transient receptor potential channel pyrexia isoform x1	7.10	10.83	76.87
LDEC007677-RA	PREDICTED: uncharacterized protein LOC103568218 isoform X3	7.12	68.37	486.93
LDEC006111-RA	sodium potassium calcium exchanger 4-like	7.30	10.19	74.41
LDEC005942-RA	peptidoglycan-recognition protein sc2-like	7.36	14.89	109.60
LDEC007811-RA	sh3 and multiple ankyrin repeat domains protein 3 isoform x1	7.58	31.06	235.46
LDEC021938-RA	cytochrome b-c1 complex subunit	7.59	9.66	73.32

	mitochondrial-like			
LDEC011534-RA	glycosyl hydrolase	7.70	5.58	42.93
LDEC021317-RA	endoplasmic reticulum metallopeptidase 1-like	7.78	30.24	235.22
LDEC002671-RA	headcase protein	7.85	25.91	203.39
LDEC019925-RA	PREDICTED: uncharacterized protein LOC103313770	7.89	11.73	92.53
LDEC002635-RA	retrovirus-related pol polyprotein from transposon partial	7.91	5.79	45.78
LDEC002639-RA	rna-directed dna polymerase from mobile element jockey-like	7.93	13.66	108.32
LDEC016747-RA	cgmp-dependent protein kinase	7.93	10.27	81.48
LDEC018721-RA	carboxypeptidase d	7.96	9.08	72.34
LDEC012330-RA	dedicator of cytokinesis protein partial	7.97	6.32	50.43
LDEC004510-RA	transcription factor deformed	7.99	7.45	59.57
LDEC023757-RA	arginine kinase	8.01	22.11	177.02
LDEC011702-RA	---NA---	8.01	32.77	262.45
LDEC007900-RA	fatty acid-binding liver	8.04	6.02	48.40
LDEC012724-RA	pancreatic triacylglycerol lipase-like	8.08	56.66	457.95
LDEC002404-RA	atp-binding cassette sub-family g member 4	8.11	6.65	53.90
LDEC022000-RA	inorganic phosphate cotransporter	8.16	8.32	67.85
LDEC005175-RA	equilibrative nucleoside transporter 1	8.33	10.83	90.15
LDEC001811-RA	lipase 1-like	8.36	4.79	40.04
LDEC013856-RA	neutral alpha-glucosidase ab	8.36	48.44	405.14
LDEC016809-RA	reverse transcriptase	8.43	7.47	63.00
LDEC000857-RA	zinc finger protein 260	8.46	13.61	115.11
LDEC012252-RA	gut-specific chitinase	8.47	76.36	646.69
LDEC010875-RA	endoplasmic reticulum metallopeptidase 1-like	8.72	33.98	296.39
LDEC014284-RA	pax-interacting protein 1	8.76	9.73	85.31
LDEC008496-RA	phosphatidylethanolamine-binding protein	8.79	111.66	981.75
LDEC004513-RA	glucose transporter type 1 isoform x1	8.95	6.42	57.46
LDEC021347-RA	GI13433	9.25	5.12	47.30
LDEC005756-RA	exonuclease gor-like protein	9.25	42.98	397.64
LDEC007365-RA	encapsulation-relating protein	9.25	87.90	813.32
LDEC022211-RA	endopolygalacturonase	9.27	49.55	459.43
LDEC007033-RA	ankyrin unc44	9.28	18.65	173.12
LDEC000757-RA	serine--trna mitochondrial	9.34	61.99	578.72
LDEC008012-RA	nesprin-1 isoform x1	9.38	95.39	895.00
LDEC021659-RA	neutral alpha-glucosidase ab	9.44	18.49	174.56
LDEC005963-RA	digestive cysteine protease intestain	9.45	38.69	365.65
LDEC020714-RA	udp-n-acetylhexosamine pyrophosphorylase	9.50	6.53	62.07
LDEC002038-RA	proteoglycan 4-like isoform x2	9.51	8.32	79.10
LDEC002890-RA	neutral alpha-glucosidase ab-like	9.75	43.13	420.72
LDEC020328-RA	agap006905-pa-like protein	9.82	28.63	281.08

LDEC001096-RA	pancreatic lipase-related protein 2-like	9.83	38.08	374.32
LDEC009661-RA	lian-aa1-like retrotransposon protein	9.91	37.23	369.12
LDEC003351-RA	serine threonine-protein kinase pak 3 isoform x3	9.94	44.15	438.85
LDEC001311-RA	ephrin-b2 isoform x1	10.05	3.74	37.54
LDEC023544-RA	growth hormone-regulated tbc protein 1-a isoform x1	10.13	8.68	87.93
LDEC010528-RA	---NA---	10.22	9.96	101.87
LDEC002752-RA	dentin sialophosphoprotein isoform x3	10.44	24.76	258.50
LDEC021393-RA	ankyrin unc44	10.56	20.27	214.05
LDEC010122-RA	isoform a	10.72	5.19	55.66
LDEC004823-RA	starry isoform b	10.76	16.98	182.76
LDEC015050-RA	polycomb group protein psc-like isoform x3	10.92	3.55	38.71
LDEC012643-RA	---NA---	10.94	70.81	774.45
LDEC010660-RA	serine protease snake-like	11.17	20.91	233.47
LDEC010547-RA	nuclear pore complex protein nup153	11.29	72.74	821.44
LDEC020896-RA	zinc finger autosomal	11.36	13.43	152.53
LDEC018397-RA	---NA---	11.36	3.93	44.61
LDEC007891-RA	hemocyte protein-glutamine gamma-	11.46	13.78	157.88
LDEC002666-RA	udp-glucuronosyltransferase 2b17-like	11.63	18.24	212.14
LDEC007765-RA	probable g-protein coupled receptor 125-like	11.66	4.35	50.74
LDEC014104-RA	isoform b	11.74	5.58	65.47
LDEC010508-RA	guanyl-nucleotide exchange factor	11.96	3.33	39.88
LDEC004973-RA	facilitated trehalose transporter tret1 isoform x1	11.97	3.89	46.56
LDEC004745-RA	protein lethal essential for life	11.98	10.41	124.68
LDEC014791-RA	PREDICTED: uncharacterized protein LOC103312905	11.99	3.24	38.83
LDEC015541-RA	glycosyl hydrolase	12.25	9.95	121.79
LDEC007978-RA	mariner transposase	12.46	7.76	96.67
LDEC020710-RA	uracil dna	12.46	6.71	83.55
LDEC007901-RA	transcription factor sox-4-like	12.61	12.15	153.16
LDEC003596-RA	protein tanc2 isoform x2	13.03	24.28	316.27
LDEC012249-RA	integral membrane protein dgcr2 idd-partial	13.06	4.29	56.05
LDEC004927-RA	muscle-specific protein 300	13.68	17.02	232.72
LDEC015677-RA	15-hydroxyprostaglandin dehydrogenase	13.73	3.85	52.89
LDEC004398-RA	hypothetical protein D910_02431	13.82	3.45	47.65
LDEC020596-RA	e3 ubiquitin-protein ligase siah2-like	13.83	3.47	47.97
LDEC020591-RA	cyclic gmp-amp synthase	14.00	5.48	76.72
LDEC007384-RA	ankyrin-3-like isoform x4	14.03	10.44	146.56
LDEC021108-RA	protein prrc1-like isoform x1	14.11	18.15	255.96
LDEC010329-RA	heat repeat-containing protein 6	14.33	5.35	76.60
LDEC010157-RA	a-kinase anchor protein partial	14.39	2.93	42.19
LDEC007080-RA	cytochrome p450 mitochondrial	14.72	24.89	366.31

LDEC016007-RA	zinc finger protein 271-like	14.74	3.14	46.33
LDEC017425-RA	thap domain-containing protein 9	14.89	5.73	85.31
LDEC014788-RA	yellow-e precursor	15.01	15.08	226.32
LDEC011207-RA	histone-lysine n-methyltransferase 2d isoform x1	15.06	15.54	234.05
LDEC023516-RA	agap002124-pa-like protein	15.10	3.07	46.29
LDEC010096-RA	syntaxin 1a	15.44	5.58	86.09
LDEC013654-RA	enhancer of split mgamma	15.67	3.10	48.63
LDEC014460-RA	glycoside hydrolase family 28 protein	16.07	51.13	821.76
LDEC014507-RA	low quality protein: probable e3 ubiquitin-protein ligase mycbp2-like	16.49	7.95	131.17
LDEC003952-RA	---NA---	16.89	2.40	40.47
LDEC009655-RA	atp-binding cassette sub-family g member 5	17.12	2.32	39.69
LDEC005077-RA	myosin light chain smooth muscle	17.14	3.33	57.15
LDEC005094-RA	potassium sodium hyperpolarization-activated cyclic nucleotide-gated channel 1-like	17.15	5.17	88.71
LDEC004070-RA	general transcription factor iih subunit 1	17.37	18.43	320.30
LDEC006869-RA	hypothetical protein YQE_09144, partial	17.45	4.12	71.91
LDEC000535-RA	thioredoxin domain-containing protein 15	17.52	24.28	425.37
LDEC004157-RA	ac1147-like partial	18.23	37.58	685.20
LDEC009341-RA	sortilin-related receptor-like	18.69	21.62	403.93
LDEC006620-RA	leukocyte receptor	18.84	17.61	331.86
LDEC000714-RA	udp-glucuronosyltransferase 2c1-like	18.87	4.31	81.36
LDEC010056-RA	senecionine n-oxygenase	19.61	13.91	272.88
LDEC004479-RA	esterase	20.20	1.95	39.49
LDEC014142-RA	---NA---	20.38	2.82	57.42
LDEC022837-RA	alpha partial	20.47	115.57	2366.21
LDEC000100-RA	paxillin isoform x3	20.62	5.63	116.17
LDEC003557-RA	PREDICTED: uncharacterized protein LOC100141760 isoform X2	20.80	16.88	351.08
LDEC003512-RA	t family of potassium channels protein 9	21.07	2.84	59.76
LDEC008867-RA	proactivator polypeptide	21.27	52.54	1117.52
LDEC001026-RA	protein lethal essential for life	21.37	2.70	57.73
LDEC003514-RA	voltage-dependent l-type calcium channel subunit beta-2 isoform x2	21.64	1.71	36.91
LDEC000778-RA	uv radiation resistance-associated gene protein	22.80	15.60	355.65
LDEC021320-RA	hypothetical protein X975_24243, partial	23.18	25.89	600.09
LDEC012240-RA	and pleckstrin domain-containing protein 2	23.24	29.74	691.02
LDEC015582-RA	c-type lectin mannose-binding isoform	23.24	2.49	57.89
LDEC007675-RA	nfx1-type zinc finger-containing protein 1-like	23.53	1.80	42.38
LDEC012588-RA	bromodomain-containing protein 4-like	24.01	45.49	1092.25
LDEC020840-RA	sjoegren syndrome nuclear autoantigen 1 homolog	24.20	1.80	43.59
LDEC002281-RA	trna methyltransferase	24.56	10.48	257.49

LDEC000016-RA	adult-specific cuticular protein acp-20	24.59	17.82	438.14
LDEC008794-RA	lipase member i	24.98	4.92	123.00
LDEC004785-RA	zinc transporter zip3-like	25.14	17.63	443.26
LDEC007429-RA	glucose dehydrogenase	25.41	1.19	30.19
LDEC017004-RA	pdz domain-containing protein 8	25.69	11.08	284.60
LDEC012042-RA	cuticular protein	29.07	1.25	36.21
LDEC002396-RA	lipase 3	29.27	2.28	66.75
LDEC003403-RA	wd repeat and fyve domain-containing protein 3	29.50	74.43	2195.32
LDEC012337-RA	PREDICTED: uncharacterized protein LOC103313825	30.28	1.30	39.45
LDEC020262-RA	copia protein	31.15	3.05	94.92
LDEC002340-RA	pickpocket protein 28-like	31.78	0.94	29.84
LDEC009907-RA	s phase cyclin a-associated protein in the endoplasmic reticulum	32.79	25.52	837.03
LDEC005244-RA	glutamate mitochondrial	34.02	1.05	35.86
LDEC006490-RA	muscle-specific protein 300	34.30	7.86	269.48
LDEC012302-RA	leukocyte tyrosine kinase receptor	36.78	2.64	97.26
LDEC004843-RA	glycoside hydrolase family 1	36.86	1.15	42.38
LDEC011796-RA	PREDICTED: uncharacterized protein LOC103311554, partial	39.23	1.02	39.84
LDEC023391-RA	diapause-associated transcript-2	40.38	0.80	32.50
LDEC020750-RA	transforming growth factor beta	40.49	17.65	714.54
LDEC001136-RA	mushroom body large-type kenyon cell-specific protein 1	42.45	6.75	286.35
LDEC007375-RA	transcription factor adf- partial	42.89	9.41	403.57
LDEC022806-RA	histone-lysine n-methyltransferase setmar	42.96	7.45	320.26
LDEC009625-RA	titin-like isoform x2	43.96	1.02	44.65
LDEC020397-RA	translational activator of cytochrome c oxidase 1	46.03	1.55	71.44
LDEC001386-RA	hypothetical protein G5I_04203	46.81	44.11	2064.98
LDEC013414-RA	neogenin-like isoform x3	47.37	4.35	206.04
LDEC006992-RA	nmda receptor-regulated protein 1	47.81	17.15	820.04
LDEC008039-RA	endocuticle structural protein bd-6-like	48.65	0.57	27.97
LDEC007345-RA	micronuclear linker histone poly isoform x3	50.54	5.65	285.69
LDEC021172-RA	leucine-rich repeat-containing protein 4	52.39	0.96	50.19
LDEC011062-RA	muscle-specific protein 300	52.76	0.82	43.47
LDEC004588-RA	lipase 3	52.78	2.15	113.28
LDEC008210-RA	protein wnt-2-like	54.14	0.79	42.54
LDEC018962-RA	glycosyl hydrolase	56.13	0.50	27.97
LDEC004760-RA	---NA---	56.24	0.84	47.42
LDEC009185-RA	e3 ubiquitin-protein ligase herc2	57.62	7.84	451.58
LDEC003417-RA	PREDICTED: uncharacterized protein LOC103518485	58.01	1.21	70.04
LDEC007043-RA	PREDICTED: uncharacterized protein LOC103521977	58.49	0.75	43.71
LDEC010969-RA	hypothetical protein TcasGA2_TC007902	59.42	30.91	1836.67

LDEC010292-RA	PREDICTED: uncharacterized protein LOC103310965	59.79	0.63	37.81
LDEC023658-RA	protein shroom	60.76	15.98	971.05
LDEC014378-RA	j domain-containing protein	64.05	18.24	1168.38
LDEC015587-RA	e3 ubiquitin-protein ligase sina	64.12	1.05	67.57
LDEC008973-RA	hypothetical protein TcasGA2_TC013968	64.33	9.39	604.07
LDEC006925-RA	nerve growth factor receptor	65.76	1.40	91.99
LDEC017936-RA	agap006140-pa-like protein	72.59	2.95	214.21
LDEC018595-RA	dna polymerase	74.99	9.20	689.73
LDEC005334-RA	hypothetical protein YQE_08052, partial	75.35	2.11	158.82
LDEC016419-RA	rnase h and integrase-like protein	79.84	0.34	27.54
LDEC001967-RA	chorion peroxidase	81.84	0.38	31.37
LDEC011168-RA	receptor tyrosine kinase torso-like protein	83.33	3.72	309.79
LDEC011001-RA	retrovirus-related pol polyprotein from transposon partial	83.57	0.36	30.43
LDEC020367-RA	juvenile hormone esterase isoform a	85.71	0.77	65.70
LDEC018629-RA	calcineurin-binding protein cabin-1-like	88.70	13.28	1177.87
LDEC007114-RA	hypothetical protein KGM_11902	88.72	0.36	32.30
LDEC007571-RA	ddb1- and cul4-associated factor 7	91.87	44.76	4112.61
LDEC017993-RA	hypothetical protein	99.55	162.19	16146.48
LDEC022110-RA	voltage-dependent t-type calcium channel subunit alpha-1g	114.22	0.54	61.29
LDEC006635-RA	---NA---	114.95	4.66	535.29
LDEC011147-RA	wd repeat-containing protein on y chromosome	116.48	0.54	62.50
LDEC017172-RA	PREDICTED: uncharacterized protein LOC103314916	117.74	6.88	809.96
LDEC003925-RA	glycosyl hydrolase	121.84	0.59	72.38
LDEC004120-RA	alpha-tocopherol transfer	123.83	0.23	28.48
LDEC008623-RA	horma domain-containing protein 1	126.21	0.69	87.07
LDEC011013-RA	pathogenesis-related protein 5	126.54	1.25	157.61
LDEC003553-RA	diapause-associated transcript-2	132.76	0.88	117.03
LDEC006139-RA	4-aminobutyrate mitochondrial	134.45	1.42	190.65
LDEC000690-RA	peritrophic matrix protein 14 precursor	139.90	0.36	50.94
LDEC002361-RA	intraflagellar transport protein 122-like protein	140.49	0.75	104.99
LDEC019632-RA	cuticle protein precursor	142.47	0.90	128.31
LDEC022189-RA	transcription factor sox-15 isoform x1	142.66	1.48	210.50
LDEC007574-RA	alpha-catulin isoform x2	146.08	0.69	100.78
LDEC016694-RA	---NA---	151.66	0.19	29.06
LDEC002494-RA	nuclease harbi1	159.22	0.34	54.92
LDEC017875-RA	facilitated trehalose transporter tret1	161.98	0.29	46.56
LDEC022660-RA	hypothetical protein SINV_04094	184.81	0.17	31.87
LDEC018338-RA	glucose dehydrogenase	197.47	0.15	30.27
LDEC006657-RA	---NA---	203.43	0.29	58.47

LDEC024379-RA	---NA---	203.67	0.23	46.83
LDEC007863-RA	PREDICTED: uncharacterized protein LOC100570366	208.12	0.19	39.88
LDEC001809-RA	rho gtpase-activating protein gacv-partial	211.75	34.70	7348.31
LDEC015538-RA	zinc finger mym-type protein 1-like	219.56	0.13	29.45
LDEC001839-RA	atp-dependent dna helicase pif1-like	246.35	0.13	33.05
LDEC007982-RA	minus-c odorant binding protein 3	248.85	0.23	57.22
LDEC009404-RA	piggybac transposable element-derived protein 4-like	2629	0.00	26.29
LDEC010421-RA	nadh dehydrogenase subunit 1	262.95	0.15	40.31
LDEC007838-RA	rna-directed dna polymerase from mobile element jockey-like	270.09	0.19	51.76
LDEC013193-RA	PREDICTED: uncharacterized protein LOC100569635	274.25	0.21	57.81
LDEC003827-RA	PREDICTED: uncharacterized protein LOC102671488	2746	0.00	27.46
LDEC021326-RA	PREDICTED: uncharacterized protein LOC103312896	275.18	0.42	116.01
LDEC004450-RA	PREDICTED: uncharacterized protein LOC103521349, partial	2769	0.00	27.69
LDEC011323-RA	paired box protein pax-5	280.12	2.18	611.92
LDEC008629-RA	tripeptidyl-peptidase 2 isoform x2	280.93	101.60	28542.00
LDEC006429-RA	agap007074-pa-like protein	2836	0.00	28.36
LDEC021488-RA	PREDICTED: uncharacterized protein LOC103318189 isoform X1	294.72	0.23	67.77
LDEC010230-RA	replication factor c4	2961	0.00	29.61
LDEC018235-RA	reverse partial	296.65	0.29	85.27
LDEC003911-RA	radial spoke head protein 4 homolog a-like	3039	0.00	30.39
LDEC023566-RA	PREDICTED: uncharacterized protein LOC100573528 isoform X1	306.10	0.11	35.19
LDEC003958-RA	ankyrin unc44	3262	0.00	32.62
LDEC015403-RA	tigger transposable element-derived protein 1-like	347.34	0.10	33.28
LDEC010259-RA	PREDICTED: uncharacterized protein LOC103313140	3476	0.00	34.76
LDEC001803-RA	voltage-dependent calcium channel subunit alpha-2 delta-3-like	351.93	0.50	175.34
LDEC020374-RA	probable atp-dependent rna helicase cg8611	352.62	19.39	6838.14
LDEC005670-RA	probable dna polymerase	3566	0.00	35.66
LDEC022187-RA	52 kda repressor of the inhibitor of the protein kinase-like	3644	0.00	36.44
LDEC019876-RA	juvenile hormone esterase isoform a	369.07	0.31	113.16
LDEC000610-RA	esterase	425.34	0.86	366.78
LDEC009328-RA	rna-directed dna polymerase from mobile element partial	4472	0.00	44.72
LDEC007728-RA	cytosol aminopeptidase	456.60	0.06	26.25
LDEC015376-RA	jerky protein homolog-like	458.52	0.34	158.16
LDEC011060-RA	nose resistant to fluoxetine protein 6	4590	0.00	45.90
LDEC023376-RA	thap domain-containing protein	4676	0.00	46.76
LDEC009634-RA	reverse transcriptase	489.06	0.52	253.03
LDEC015659-RA	tetratricopeptide repeat protein 29	533.87	0.21	112.53
LDEC004057-RA	hypothetical protein EAG_03557	536.22	124.44	66728.42

LDEC012664-RA	hypothetical protein TcasGA2_TC002334	5390	0.00	53.90
LDEC006845-RA	ion channel nompc	550.31	5.38	2963.25
LDEC010454-RA	agap007105-pa-like protein	553.08	0.06	31.80
LDEC004545-RA	jerky protein homolog-like	5605	0.00	56.05
LDEC015722-RA	hypothetical protein TcasGA2_TC002334	561.18	25.60	14366.84
LDEC008262-RA	multidrug resistance-associated protein 4	567.43	0.15	86.99
LDEC009812-RA	zinc finger protein 271-like	611.50	97.17	59421.59
LDEC010571-RA	PREDICTED: uncharacterized protein LOC105197973	6492	0.00	64.92
LDEC011636-RA	retrovirus-related pol polyprotein from transposon partial	657.81	0.13	88.24
LDEC014143-RA	ac transposable element-derived protein partial	669.46	0.13	89.80
LDEC003427-RA	lipase 3-like	685.37	16.96	11623.06
LDEC009584-RA	PREDICTED: uncharacterized protein LOC105205337	6894	0.00	68.94
LDEC006790-RA	major antigen-like	693.73	0.06	39.88
LDEC024339-RA	hypothetical protein D910_04816	705.28	0.04	27.03
LDEC005883-RA	potassium sodium hyperpolarization-activated cyclic nucleotide-gated channel 1-like	706.30	0.08	54.14
LDEC003625-RA	chymotrypsin inhibitor-like	7132	0.00	71.32
LDEC002429-RA	facilitated trehalose transporter tret1-2 homolog	7789	0.00	77.89
LDEC024413-RA	hypothetical protein TcasGA2_TC010277	7925	0.00	79.25
LDEC003310-RA	dynein heavy chain	810.26	0.04	31.05
LDEC005624-RA	peroxisomal acyl-coenzyme a oxidase 3 isoform x1	829.28	0.11	95.35
LDEC013267-RA	reverse transcriptase	830.98	0.06	47.77
LDEC007659-RA	hypothetical protein TcasGA2_TC001846	844.91	0.04	32.38
LDEC013319-RA	reverse transcriptase	894.64	0.56	497.16
LDEC014670-RA	multidrug resistance-associated protein 4-like	913.19	0.04	35.00
LDEC016901-RA	bifunctional arginine demethylase and lysyl-hydroxylase jmjd6	921.35	0.04	35.31
LDEC005430-RA	wash complex subunit fam21a	938.50	15.81	14836.81
LDEC011012-RA	tigger transposable element-derived protein 6-like	938.67	0.04	35.97
LDEC007241-RA	transcription factor spt20 homolog	9578	0.00	95.78
LDEC005604-RA	retrovirus-related pol polyprotein from transposon tnt 1- partial	9636	0.00	96.36
LDEC023828-RA	zinc finger ccch domain-containing protein 13	1000.02	119.71	119710.99
LDEC017456-RA	teneurin-a-like isoform x3	1013.95	0.13	136.01
LDEC010717-RA	cathepsin partial	10453	0.00	104.53
LDEC003754-RA	neutrophil collagenase	1182.26	0.06	67.97
LDEC005772-RA	rho gtpase-activating protein gacv-partial	1186.05	43.25	51296.59
LDEC019701-RA	uracil dna	1205.70	0.04	46.21
LDEC018309-RA	---NA---	1304.56	0.04	50.00
LDEC012137-RA	hypothetical protein X777_05526, partial	1343.97	0.11	154.52

LDEC002018-RA	---NA---	13995	0.00	139.95
LDEC013858-RA	innexin inx7	2041.44	0.04	78.24
LDEC004565-RA	dc-stamp domain-containing protein 1-like	2088.66	0.46	960.58
LDEC015671-RA	nuclear cap-binding protein subunit 2	21651	0.00	216.51
LDEC017984-RA	chaoptic-like protein	2304.39	0.23	529.90
LDEC007903-RA	arylphorin-like hexameric storage protein	2670.79	0.23	614.15
LDEC007622-RA	PREDICTED: uncharacterized protein LOC103569733	2676.39	0.04	102.57
LDEC012233-RA	trans- -enoyl- reductase	2910.81	0.04	111.56
LDEC009422-RA	PREDICTED: uncharacterized protein LOC103314818 isoform X3	29670	0.00	296.70
LDEC010733-RA	PREDICTED: uncharacterized protein LOC103314729	30065	0.00	300.65
LDEC009329-RA	transmembrane protease	3260.39	0.04	124.95
LDEC005612-RA	hypothetical protein TcasGA2_TC001429	3530.47	0.02	67.65
LDEC006391-RA	AAEL017356-PA	3699.09	0.34	1275.91
LDEC009513-RA	PREDICTED: uncharacterized protein LOC103312770	4035.48	0.08	309.32
LDEC003039-RA	cathepsin partial	7925.90	0.06	455.64
LDEC010683-RA	r2 protein	9578.90	1.90	18172.08
LDEC003564-RA	hypothetical protein TcasGA2_TC010197	11909.03	0.10	1141.04
LDEC018598-RA	wd repeat and fyve domain-containing protein 3-like	119283	0.00	1192.83
LDEC017505-RA	zinc finger bed domain-containing partial	19737.05	0.13	2647.49
LDEC016135-RA	octopamine receptor partial	21039.57	0.13	2822.21
LDEC014002-RA	gtp-binding protein di-ras2	28021.60	0.02	536.97
LDEC017467-RA	PREDICTED: uncharacterized protein LOC105199705	29636.00	0.08	2271.61
LDEC020677-RA	cathepsin l	30964.00	0.04	1186.70
LDEC014308-RA	2209395adiapause protein 1	34954.13	0.04	1339.62
LDEC013821-RA	---NA---	37969.91	0.04	1455.20
LDEC013014-RA	probable multidrug resistance-associated protein lethal 03659 isoform x3	779505	0.00	7795.05
LDEC010016-RA	voltage-dependent t-type calcium channel subunit alpha-1g	101931.85	0.42	42972.08

Table 6S. Significantly differentially expressed genes, after Bonferroni correction, in an adult male and female *Leptinotarsa decemlineata*.

Feature ID	Gene name	Experiment - Fold Change (normalized values)	Adult male - Normalized expression values	Adult female - Normalized expression values
LDEC007572-RA	hypothetical protein TcasGA2_TC009650	4266.56	0.29	1216.73
LDEC016351-RA	arginine serine-rich splicing factor	3025.31	0.05	143.79
LDEC022063-RA	---NA---	1775.50	0.10	168.78
LDEC002409-RA	PREDICTED: uncharacterized protein LOC100906494	1552.49	0.10	147.58
LDEC001554-RA	female sterile m3	1323.15	0.05	62.89
LDEC001553-RA	grip and coiled-coil domain-containing protein pfc0235w-like	1139.24	0.05	54.15
LDEC000175-RA	sterol regulatory element-binding protein cleavage-activating protein	828.37	0.10	78.74
LDEC000096-RA	krueppel c2h2-type zinc finger	719.28	0.10	68.37
LDEC004859-RA	protein d7	610.20	0.10	58.01
LDEC013399-RA	hypothetical protein TcasGA2_TC009650	502.60	0.10	47.78
LDEC000848-RA	fatty acid-binding liver	380.49	0.10	36.17
LDEC011269-RA	sorting nexin-4-like	345.87	0.10	32.88
LDEC017199-RA	---NA---	313.97	0.14	44.77
LDEC000487-RA	g2 mitotic-specific cyclin-b3	291.24	0.33	96.90
LDEC006145-RA	hypothetical protein TcasGA2_TC011429	220.40	0.19	41.90
LDEC004916-RA	spfh domain-containing protein 1	196.37	1.57	308.00
LDEC019814-RA	---NA---	150.35	0.62	92.90
LDEC004195-RA	cholesterol desaturase daf-36	136.90	0.33	45.55
LDEC004854-RA	tubulin alpha-1 chain-like	129.09	0.38	49.09
LDEC004853-RA	tubulin alpha-1 chain	126.88	0.48	60.31
LDEC010877-RA	cathepsin b precursor	124.77	1.09	136.40
LDEC013763-RA	agap005178-pa-like protein	118.64	0.71	84.58
LDEC001642-RA	PREDICTED: uncharacterized protein LOC103314540	116.01	0.24	27.57
LDEC006721-RA	isoform b	103.66	0.90	93.61
LDEC001262-RA	transcription factor homolog	97.42	0.57	55.56
LDEC014744-RA	heterotrimeric gtp-binding protein alpha subunit g-alpha-q	92.89	0.57	52.98
LDEC019649-RA	---NA---	89.41	1.14	102.00
LDEC014639-RA	cytochrome p450 9z4	88.17	2.04	180.21
LDEC009305-RA	e3 ubiquitin-protein ligase siah1b	83.77	0.48	39.81
LDEC016838-RA	myotubularin-related protein 14	83.29	3.94	328.57

LDEC010565-RA	lys-63-specific deubiquitinase brcc36-like isoform x2	75.62	0.43	32.35
LDEC005352-RA	ankyrin repeat and sam domain-containing protein 6-like	69.19	0.62	42.75
LDEC000761-RA	aldose reductase	66.88	0.81	54.04
LDEC000908-RA	ka261_mesma ame: full=potassium channel blocker alpha-ktx ame: full=neurotoxin 86 flags: precursor	64.78	0.57	36.95
LDEC001212-RA	mrna cap-binding protein eif4e	63.57	0.76	48.34
LDEC019869-RA	glucose dehydrogenase	52.71	1.76	92.69
LDEC018594-RA	pericentriolar material 1 protein isoform x4	51.67	1.95	100.69
LDEC013779-RA	protein claret segregational	41.12	2.57	105.53
LDEC007217-RA	cytochrome p450 6k1	40.85	5.28	215.53
LDEC020419-RA	protein beta isoform-like isoform x18	36.78	1.66	61.19
LDEC006597-RA	protein asteroid	32.97	8.37	275.80
LDEC022696-RA	vitellogenin receptor	29.48	1.28	37.83
LDEC000821-RA	juvenile hormone-inducible protein	28.23	1.05	29.52
LDEC004932-RA	protein bicaudal c	27.40	1.14	31.25
LDEC022697-RA	vitellogenin receptor	23.92	1.90	45.48
LDEC017412-RA	uracil dna	23.03	2.71	62.39
LDEC023093-RA	beta- -galactosyltransferase 5-like	19.95	1.81	36.03
LDEC007553-RA	vitellogenin receptor	18.95	10.50	199.07
LDEC004440-RA	eri1 exoribonuclease 2-like isoform x3	18.95	5.56	105.36
LDEC018965-RA	lymphoid-specific helicase-like	17.43	3.47	60.48
LDEC002557-RA	juvenile hormone partial	17.06	7.51	128.08
LDEC013928-RA	bifunctional udp-n-acetylglucosamine transferase and deubiquitinase alg13 isoform x1	15.88	2.14	33.97
LDEC017948-RA	---NA---	15.76	2.04	32.21
LDEC007552-RA	vitellogenin receptor	15.29	4.75	72.69
LDEC018060-RA	esterase	15.00	3.52	52.77
LDEC007638-RA	ecdysone 20-monooxygenase isoform x2	13.78	16.16	222.71
LDEC003924-RA	serine threonine-protein kinase nek8	13.75	2.99	41.16
LDEC015624-RA	glutathione s-transferase 1-like	12.86	4.14	53.19
LDEC000143-RA	PREDICTED: uncharacterized protein LOC100141808	12.66	8.03	101.71
LDEC000217-RA	ubiquitin carboxyl-terminal hydrolase 35	12.25	2.76	33.76
LDEC015538-RA	potentail helicase mov-10	11.88	4.61	54.78
LDEC001728-RA	peptidoglycan-recognition protein sc2-like	10.99	6.08	66.89
LDEC008871-RA	e3 ubiquitin-protein ligase siah2-like	10.64	28.09	298.91
LDEC004155-RA	forkhead box protein p4	10.40	12.31	128.08
LDEC023861-RA	meiosis arrest female protein 1 isoform x2	10.13	5.04	51.03
LDEC020380-RA	eukaryotic translation initiation factor 4e transporter-like isoform x2	9.98	4.61	46.01
LDEC009652-RA	poly rna polymerase gld-2 homolog a-like	9.97	14.35	143.08
LDEC009134-RA	cyclin partial	9.63	5.13	49.44

LDEC009524-RA	mitogen-activated protein kinase erk-	9.58	3.85	36.88
LDEC008209-RA	PREDICTED: uncharacterized protein LOC103312207	9.49	7.03	66.75
LDEC020501-RA	beta- -glucosyltransferase	9.26	4.33	40.06
LDEC008970-RA	enolase-phosphatase e1-like	9.19	20.72	190.37
LDEC012294-RA	leucine-rich repeats and immunoglobulin-like domains protein 1	9.17	4.71	43.14
LDEC009249-RA	bipolar kinesin krp-130	8.34	7.46	62.25
LDEC016555-RA	meiosis arrest female protein 1 isoform x2	7.97	5.42	43.18
LDEC016554-RA	meiosis arrest female protein 1 isoform x1	7.53	15.21	114.49
LDEC003887-RA	agap011183-pa-like protein	7.43	12.12	90.03
LDEC003354-RA	isoform b	7.16	10.88	77.93
LDEC013885-RA	pancreatic triacylglycerol lipase-like	6.96	15.45	107.48
LDEC005608-RA	e3 ubiquitin-protein ligase siah2-like	6.80	7.65	52.02
LDEC002556-RA	juvenile hormone partial	6.73	12.22	82.18
LDEC019546-RA	microtubule-associated protein futsch-like	6.64	34.74	230.68
LDEC003025-RA	f-box lrr-repeat protein 4	6.51	7.46	48.59
LDEC012661-RA	regucalcin-like isoform x2	6.32	12.83	81.12
LDEC018236-RA	set and mynd domain-containing protein 4-like	6.31	9.79	61.79
LDEC018567-RA	probable myosin light chain kinase ddb_g0279831	6.23	12.93	80.48
LDEC008679-RA	laccase 1 isoform x1	6.16	10.79	66.50
LDEC018655-RA	PREDICTED: uncharacterized protein LOC660825	5.97	8.75	52.24
LDEC006198-RA	hunchback	5.87	23.67	138.91
LDEC018741-RA	heat shock 70 kda protein cognate 4	5.71	8.46	48.31
LDEC000793-RA	pro-phenol oxidase subunit 2	5.51	33.84	186.44
LDEC017298-RA	2 -phosphodiesterase 12-like	5.40	10.55	57.01
LDEC008797-RA	#NAME?	5.29	10.65	56.31
LDEC014976-RA	zinc finger protein 484-like	5.26	11.83	62.25
LDEC020084-RA	feline leukemia virus subgroup c receptor-related protein 2-like	5.21	28.95	150.91
LDEC020382-RA	eukaryotic translation initiation factor 4e transporter-like isoform x4	5.21	40.50	211.07
LDEC009553-RA	ankyrin unc44	5.20	10.50	54.64
LDEC019548-RA	trafficking kinesin-binding protein milt-like	5.05	20.25	102.31
LDEC020044-RA	growth factor receptor-bound protein 14-like	4.93	26.66	131.44
LDEC005537-RA	toll-like receptor 3	4.84	11.69	56.59
LDEC000794-RA	pro-phenol oxidase subunit 2	4.81	10.60	50.96
LDEC009537-RA	hyaluronan mediated motility receptor-like isoform x1	4.76	13.12	62.46
LDEC001146-RA	protein isoform b-like	4.75	14.88	70.64
LDEC005298-RA	pr domain zinc finger protein partial	4.75	53.00	251.56
LDEC004988-RA	nfx1-type zinc finger-containing protein 1-like isoform x1	4.66	12.74	59.42
LDEC013432-RA	kinesin-like protein nod	4.59	11.69	53.72

LDEC017031-RA	isoform a	4.59	11.36	52.13
LDEC002991-RA	PREDICTED: uncharacterized protein LOC103314074	4.52	50.57	228.62
LDEC014462-RA	probable salivary secreted peptide	4.50	65.26	293.85
LDEC006903-RA	peptidoglycan-recognition protein precursor	4.25	29.71	126.31
LDEC011968-RA	kynurenine--oxoglutarate transaminase 3 isoform x2	4.25	21.01	89.29
LDEC000534-RA	methenyltetrahydrofolate synthase domain-containing protein	4.24	20.44	86.67
LDEC014904-RA	female sterile nasrat	4.24	18.87	79.98
LDEC008359-RA	inorganic phosphate cotransporter	4.23	15.35	65.01
LDEC010104-RA	protein singed	4.23	25.14	106.42
LDEC019972-RA	gamma-interferon-inducible lysosomal thiol reductase-like	4.23	24.91	105.29
LDEC007360-RA	synaptic vesicle glycoprotein 2b-like	4.11	18.01	74.04
LDEC000956-RA	agap010793-pa-like protein	4.08	52.76	215.03
LDEC003154-RA	nuclear pore complex protein ddb_g0274915 homolog isoform x1	4.05	20.63	83.52
LDEC010871-RA	cathepsin b precursor	4.01	62.36	250.35
LDEC011073-RA	low quality protein: trithorax group protein osa	4.01	43.11	172.71
LDEC003786-RA	gamma-interferon-inducible lysosomal thiol reductase-like	4.00	83.37	333.52
LDEC001019-RA	e3 ubiquitin-protein ligase rnf220-like isoform x2	3.99	20.06	80.12
LDEC008909-RA	histone deacetylase complex subunit sap130	3.96	14.59	57.83
LDEC010425-RA	gut-specific chitinase	3.91	48.81	190.83
LDEC005758-RA	diapause-associated transcript-2	3.85	133.65	514.58
LDEC005947-RA	serine threonine-protein kinase pak 3 isoform x3	3.83	54.42	208.66
LDEC010990-RA	slit homolog 3	3.74	19.58	73.33
LDEC013919-RA	cyclomaltodextrin partial	3.61	56.99	206.01
LDEC001314-RA	isoform b	3.59	28.47	102.07
LDEC024272-RA	sphingolipid delta -desaturase des1	3.56	29.14	103.77
LDEC002018-RA	protein lsm14 homolog b isoform x3	3.55	64.93	230.78
LDEC014857-RA	ww domain-containing adapter protein with coiled-coil-like isoform x3	3.53	52.66	185.84
LDEC013663-RA	protein phosphatase 1 regulatory subunit 3b	3.48	20.34	70.85
LDEC007209-RA	nfx1-type zinc finger-containing protein 1-like isoform x1	3.48	36.27	126.10
LDEC018522-RA	lipase 3	3.46	59.22	205.02
LDEC003930-RA	serine threonine kinase nlk	3.44	44.58	153.28
LDEC010361-RA	succinyl- synthetase small	3.40	20.91	71.17
LDEC003710-RA	round spermatid basic protein 1	3.40	23.00	78.25
LDEC019100-RA	bifunctional methylenetetrahydrofolate dehydrogenase mitochondrial isoform x3	3.36	40.40	135.86
LDEC007358-RA	protein elys-like isoform x3	3.36	23.62	79.42
LDEC020878-RA	hypothetical protein TcasGA2_TC013968	3.34	21.77	72.66
LDEC000918-RA	glycosyl hydrolase	3.29	158.46	521.06

LDEC013494-RA	protein takeout-like	3.29	39.69	130.49
LDEC002019-RA	protein lsm14 homolog b isoform x4	3.26	29.04	94.63
LDEC001224-RA	glycosyl hydrolase	3.24	23.57	76.48
LDEC003442-RA	hypothetical protein YQE_03563, partial	3.24	214.69	695.39
LDEC006590-RA	thyroid adenoma-associated protein homolog	3.22	35.55	114.38
LDEC015196-RA	lysosomal aspartic protease	3.21	46.77	150.34
LDEC015194-RA	lysosomal aspartic protease	3.20	109.37	350.47
LDEC007173-RA	---NA---	3.20	56.47	180.85
LDEC000150-RA	gem-associated protein 5	3.19	39.21	125.14
LDEC015591-RA	uncharacterized threonine-rich gpi-anchored glyco	3.17	26.57	84.30
LDEC013455-RA	zinc transporter 1	3.11	24.38	75.74
LDEC018746-RA	hypothetical protein YQE_08038, partial	3.05	50.14	153.14
LDEC004242-RA	PREDICTED: uncharacterized protein LOC103314372	3.04	40.88	124.08
LDEC000995-RA	insulin-like receptor	3.03	25.90	78.57
LDEC016448-RA	PREDICTED: uncharacterized protein LOC103312904	2.97	65.73	195.18
LDEC017851-RA	protein casc3	2.95	48.20	142.31
LDEC004665-RA	udp-glucuronosyltransferase 2b7	2.89	53.19	153.49
LDEC021327-RA	dnaj homolog subfamily c member 16-like	2.82	530.53	1496.46
LDEC007212-RA	nfx1-type zinc finger-containing protein 1-like isoform x1	2.81	31.18	87.70
LDEC018730-RA	protein smaug homolog 1	2.80	51.24	143.51
LDEC000615-RA	protein lethal essential for life	2.78	33.75	93.86
LDEC013918-RA	maltase 2	2.78	121.39	337.45
LDEC013640-RA	constitutive coactivator of ppar-gamma-like protein 1 isoform x3	2.76	63.97	176.39
LDEC018941-RA	alpha partial	2.75	303.67	835.40
LDEC006825-RA	muscle m-line assembly protein unc-89-like	2.75	39.59	108.68
LDEC007592-RA	---NA---	2.72	1612.68	4393.18
LDEC021303-RA	thyroid receptor-interacting protein 11-like	2.72	116.78	317.10
LDEC020670-RA	phosphoenolpyruvate carboxykinase	2.69	528.96	1422.14
LDEC017156-RA	apolipophorin- partial	2.68	577.30	1545.90
LDEC011578-RA	46 kda fk506-binding nuclear protein	2.67	50.24	134.24
LDEC003980-RA	protein argonaute-3	2.67	36.93	98.42
LDEC012338-RA	cytochrome p450-like protein	2.65	82.27	217.90
LDEC006232-RA	receptor-type tyrosine-protein phosphatase f-like	2.56	94.68	242.32
LDEC001179-RA	protocadherin beta-15-like	2.54	121.06	307.83
LDEC021910-RA	probable low-specificity l-threonine aldolase 2 isoform x1	2.51	76.14	191.04
LDEC014642-RA	cytochrome p450 9z4	2.50	64.59	161.28
LDEC008226-RA	cyclic gmp-amp synthase	2.48	41.97	104.15
LDEC003401-RA	isoform b	2.48	131.37	325.35
LDEC011313-RA	facilitated trehalose transporter tret1-like	2.48	920.27	2278.41

LDEC004221-RA	casein kinase ii subunit alpha	2.48	78.04	193.20
LDEC018543-RA	fibrous sheath cabyr-binding protein isoform x7	2.47	42.97	106.31
LDEC001880-RA	protein pat1 homolog 1	2.47	179.52	443.69
LDEC021068-RA	glycosyl hydrolase	2.46	88.31	217.44
LDEC022004-RA	lysosomal aspartic protease	2.46	824.74	2025.87
LDEC020164-RA	facilitated trehalose transporter tret1	2.45	45.39	111.34
LDEC016498-RA	probable serine threonine-protein kinase ddb_g0282963	2.44	76.19	186.19
LDEC016034-RA	acyl- delta desaturase	2.43	65.73	159.86
LDEC003148-RA	glucosidase 2 subunit beta	2.43	75.48	183.50
LDEC007506-RA	pancreatic triacylglycerol lipase-like	2.43	48.34	117.25
LDEC003518-RA	safb-like transcription modulator	2.42	69.25	167.65
LDEC001030-RA	protein disulfide-isomerase a3	2.42	74.00	178.76
LDEC017303-RA	---NA---	2.42	104.00	251.20
LDEC018833-RA	hypothetical protein TcasGA2_TC007902	2.41	94.25	227.17
LDEC003616-RA	apolipoprotein li-ii precursor	2.40	340.98	818.94
LDEC022111-RA	hypothetical protein TcasGA2_TC000293	2.40	53.99	129.64
LDEC009315-RA	ly-6 neurotoxin-related protein	2.39	172.96	413.22
LDEC010880-RA	cathepsin b	2.38	135.27	321.74
LDEC006735-RA	PREDICTED: uncharacterized protein LOC103313913 isoform X1	2.36	75.10	177.24
LDEC003414-RA	ankyrin repeat domain-containing protein 17	2.35	177.86	417.64
LDEC005245-RA	helicase mov-10-	2.34	88.31	206.26
LDEC001355-RA	serine threonine-protein kinase haspin-like protein	2.34	88.41	206.47
LDEC001765-RA	aminopeptidase n	2.34	55.89	130.52
LDEC006607-RA	myosin heavy chain 95f isoform x1	2.33	337.13	787.05
LDEC016586-RA	kynurenine alpha-aminoadipate mitochondrial-like	2.30	312.46	719.10
LDEC022003-RA	lysosomal aspartic protease-like	2.30	387.84	892.41
LDEC002149-RA	probable multidrug resistance-associated protein lethal 03659	2.29	101.19	231.42
LDEC013643-RA	facilitated trehalose transporter tret1	2.26	94.16	212.87
LDEC001566-RA	PREDICTED: uncharacterized protein CG10915 isoform X1	2.25	64.45	145.21
LDEC021138-RA	endonuclease-reverse transcriptase	2.24	99.62	223.53
LDEC007737-RA	peptidyl-prolyl cis-trans isomerase-like 2-like	2.24	107.89	241.86
LDEC018745-RA	major facilitator superfamily domain-containing protein 6 isoform x2	2.24	142.64	319.54
LDEC003795-RA	fatty acid synthase	2.24	234.51	524.24
LDEC003125-RA	tubulin-specific chaperone a	2.23	123.39	275.06
LDEC001178-RA	PREDICTED: uncharacterized protein LOC656855 isoform X3	2.22	1025.22	2276.22
LDEC019089-RA	l-allo-threonine aldolase	2.22	103.33	229.26
LDEC018238-RA	agap004396-pa-like protein	2.21	350.29	773.07
LDEC007614-RA	low quality protein: calreticulin-like	2.21	262.93	580.19
LDEC008159-RA	lysosomal aspartic protease	2.20	344.73	759.48

LDEC001075-RA	zinc-type alcohol dehydrogenase-like protein	2.19	156.56	342.79
LDEC003582-RA	delta-1-pyrroline-5-carboxylate synthase	2.17	62.74	136.36
LDEC005021-RA	leucine-rich repeat-containing protein ddb_g0290503-like isoform x1	2.16	84.79	183.32
LDEC011732-RA	major facilitator superfamily domain-containing protein 1-like	2.16	60.79	131.26
LDEC011080-RA	zinc finger mym-type protein 3	2.16	72.63	156.67
LDEC013717-RA	hydroxyacid oxidase 1	2.16	103.61	223.31
LDEC007282-RA	juvenile hormone acid methyltransferase	2.15	92.54	198.90
LDEC022662-RA	nadh dehydrogenase subunit 1	2.15	510.47	1095.59
LDEC013104-RA	serine threonine-protein phosphatase 2a 55 kda regulatory subunit b alpha isoform isoform x1	2.14	102.52	219.46
LDEC019073-RA	sporulation-specific protein 15	2.13	159.37	340.10
LDEC003829-RA	protein transport protein sec61 subunit alpha isoform 2	2.13	105.80	225.33
LDEC006502-RA	secretion-regulating guanine nucleotide exchange factor	2.12	72.58	154.06
LDEC005620-RA	ankyrin-1-like isoform x3	2.12	247.01	523.75
LDEC016690-RA	surfeit locus protein 4 homolog	2.11	70.25	148.50
LDEC014505-RA	protein argonaute-2-like	2.10	97.67	205.05
LDEC003808-RA	atp-binding cassette sub-family g member 4	2.10	66.68	139.97
LDEC004071-RA	calcium-independent phospholipase a2-gamma	2.10	86.36	180.99
LDEC006357-RA	glycoside hydrolase family protein 48	2.09	667.27	1396.66
LDEC010111-RA	rna-binding protein lark isoform x4	2.08	91.21	189.48
LDEC009437-RA	aldose reductase	2.08	72.29	150.16
LDEC016836-RA	polya-binding protein interacting	2.07	90.40	187.32
LDEC011517-RA	sporulation-specific protein 15	2.07	199.81	412.69
LDEC019483-RA	gamma-interferon-inducible lysosomal thiol reductase-like	2.06	473.11	975.08
LDEC001614-RA	nad dependent epimerase	2.05	79.85	163.79
LDEC003369-RA	mog interacting and ectopic p-granules protein 1 isoform x2	2.04	113.74	232.16
LDEC001072-RA	eh domain-containing protein 3	2.02	155.42	314.48
LDEC004548-RA	PREDICTED: uncharacterized protein LOC661444	2.02	173.48	349.98
LDEC006826-RA	lim and calponin homology domains-containing protein 1 isoform x2	2.01	109.18	219.78
LDEC007937-RA	protein strawberry notch	1.99	77.95	155.47
LDEC009925-RA	lipophorin receptor	1.98	286.46	568.09
LDEC013716-RA	isocitrate dehydrogenase	1.96	179.33	351.96
LDEC003678-RA	hypothetical protein YQE_05934, partial	1.96	105.80	207.60
LDEC007487-RA	u-box domain containing 5	1.96	177.81	348.17
LDEC024548-RA	cytochrome p450 6a2	1.96	1065.33	2085.78
LDEC007843-RA	cadherin 1	1.95	85.89	167.79
LDEC020645-RA	tho complex subunit 2	1.94	109.22	212.24
LDEC000066-RA	hypothetical protein EAG_03557	1.94	104.76	203.50

LDEC017044-RA	PREDICTED: uncharacterized protein LOC659539	1.94	347.92	675.71
LDEC010363-RA	neurogenic protein mastermind-partial	1.94	92.11	178.86
LDEC020217-RA	myosin heavy non-muscle isoform x1	1.93	243.59	471.05
LDEC007944-RA	probable serine threonine-protein kinase ddb_g0282963 isoform x1	1.93	105.42	203.32
LDEC000083-RA	poly rna polymerase protein cid1	1.92	106.99	204.91
LDEC014909-RA	tppp family protein cg45057	1.90	115.16	219.17
LDEC021326-RA	defensin precursor	1.90	1793.68	3411.66
LDEC006435-RA	rna-binding protein fusilli isoform x2	1.89	97.29	183.50
LDEC008670-RA	leucine-rich repeat-containing protein 24-like isoform x1	1.89	106.18	200.17
LDEC016249-RA	23 kda integral membrane	1.87	174.81	326.48
LDEC006511-RA	d-3-phosphoglycerate dehydrogenase	1.87	251.29	469.03
LDEC003847-RA	alpha-aminoadipic semialdehyde mitochondrial isoform x1	1.85	138.64	256.58
LDEC009333-RA	amp deaminase 2 isoform x6	1.83	117.78	215.56
LDEC015913-RA	protein toll	1.82	175.34	318.44
LDEC017469-RA	cullin-associated nedd8-dissociated protein 1	1.81	165.36	299.90
LDEC005653-RA	mucin- partial	1.81	176.19	319.01
LDEC006484-RA	extracellular domains-containing protein cg31004-like	1.81	121.44	219.56
LDEC020336-RA	aldo-keto reductase	1.80	133.37	239.88
LDEC008144-RA	cytochrome p450 6a2	1.80	723.54	1299.30
LDEC021947-RA	leucine-rich ppr motif-containing mitochondrial	1.79	162.03	290.52
LDEC003960-RA	reverse transcriptase	1.79	1477.70	2648.60
LDEC003226-RA	nuclear receptor coactivator 7 isoform x5	1.79	147.82	264.86
LDEC008160-RA	lysosomal aspartic protease	1.79	1093.61	1954.23
LDEC001405-RA	sortilin-related receptor-like	1.79	283.94	507.25
LDEC001101-RA	agap008807-pa-like protein	1.78	126.52	225.47
LDEC008460-RA	zinc finger protein 271-like	1.77	135.46	240.41
LDEC009330-RA	purine nucleoside phosphorylase-like isoform x2	1.76	242.02	426.95
LDEC015379-RA	ribosome-binding protein 1-like isoform x1	1.76	215.45	379.92
LDEC006128-RA	rna-binding protein squid isoform x3	1.76	166.92	293.00
LDEC005887-RA	adenylosuccinate lyase	1.75	185.13	323.82
LDEC018465-RA	cathepsin b	1.74	226.05	394.29
LDEC006522-RA	protein phosphatase 1l	1.74	184.46	321.49
LDEC008683-RA	protein prrc2c isoform x6	1.74	224.25	390.50
LDEC005598-RA	phosphoribosylaminoimidazole carboxylase	1.74	312.13	543.25
LDEC004822-RA	cysteine proteinase cg12163	1.73	244.11	423.13
LDEC000085-RA	spectrin beta chain isoform x4	1.73	323.39	558.00
LDEC008040-RA	PREDICTED: uncharacterized protein LOC103314979	1.73	504.05	869.69
LDEC006988-RA	rna-binding protein squid isoform x3	1.73	204.57	352.88
LDEC019539-RA	peritrophic matrix protein 1-c	1.72	569.64	982.16

	precursor			
LDEC014553-RA	mitogen-activated protein kinase 1	1.72	160.08	275.76
LDEC008587-RA	clustered mitochondria protein homolog	1.72	143.68	246.64
LDEC003340-RA	ubiquitin carboxyl-terminal hydrolase 47 isoform x1	1.72	238.36	409.08
LDEC007388-RA	ammecr1-like protein	1.71	252.29	432.51
LDEC008164-RA	cytoplasmic a3	1.71	1503.65	2573.25
LDEC008161-RA	lysosomal aspartic protease	1.70	230.52	392.27
LDEC014164-RA	importin subunit alpha	1.70	156.09	265.54
LDEC017401-RA	clathrin heavy chain	1.70	254.85	432.44
LDEC015900-RA	rps6-p70-protein partial	1.69	209.99	354.51
LDEC001096-RA	hypothetical protein TcasGA2_TC004643	1.68	157.56	265.29
LDEC012911-RA	cytochrome p450 6bq11	1.68	273.49	459.76
LDEC007909-RA	nucleoprotein tpr isoform x2	1.68	171.15	287.58
LDEC004393-RA	nuclear factor related to kappa-b-binding protein isoform x1	1.67	210.84	353.02
LDEC002545-RA	proactivator polypeptide	1.67	239.36	400.76
LDEC000182-RA	atp-dependent rna	1.67	474.54	794.41
LDEC003831-RA	isoform a	1.67	195.16	326.51
LDEC021271-RA	atp-dependent rna helicase p62	1.67	714.32	1190.58
LDEC018935-RA	heat shock 70 kda protein cognate 3 isoform x1	1.66	208.61	347.29
LDEC007575-RA	e3 ubiquitin-protein ligase ubr4 isoform x1	1.65	476.34	786.91
LDEC006485-RA	eukaryotic translation initiation factor 4 gamma 2 isoform x2	1.65	197.49	325.98
LDEC007679-RA	xanthine dehydrogenase isoform x1	1.65	335.18	553.05
LDEC017981-RA	atp-dependent rna helicase p62	1.65	230.76	380.59
LDEC001493-RA	splicing factor 3b subunit 3	1.65	183.46	302.20
LDEC016510-RA	fatty acid synthase	1.65	339.31	558.22
LDEC000846-RA	fatty acid-binding protein 12-like	1.64	222.15	364.38
LDEC017559-RA	esterase	1.64	206.52	338.72
LDEC007070-RA	aldehyde mitochondrial	1.62	289.88	470.94
LDEC006126-RA	ras gtpase-activating protein-binding protein 2	1.61	189.83	306.41
LDEC014330-RA	sorbitol dehydrogenase	1.61	312.98	504.92
LDEC005651-RA	ubiquitin carboxyl-terminal hydrolase 2-like isoform x2	1.61	733.91	1182.72
LDEC012938-RA	vigilin	1.61	279.05	449.50
LDEC016723-RA	protein ctla-2-alpha	1.61	408.61	656.00
LDEC018653-RA	atp-dependent rna helicase me31b	1.61	309.75	497.24
LDEC002701-RA	probable atp-dependent rna helicase ddx17-like	1.60	1014.85	1622.16
LDEC009735-RA	juvenile hormone binding protein partial	1.59	368.26	585.43
LDEC012360-RA	titin isoform x2	1.58	305.81	482.87
LDEC012939-RA	vigilin	1.55	533.85	827.26
LDEC006099-RA	v-type proton atpase catalytic subunit a	1.54	432.00	667.43
LDEC003658-RA	phosphoribosylformylglycinamidine synthase-like	1.54	262.84	405.58

LDEC011312-RA	hypothetical protein YQE_05172, partial	1.54	1519.81	2344.95
LDEC018225-RA	myrosinase 1-like	1.54	452.20	696.24
LDEC018262-RA	synaptic vesicle glycoprotein 2b	1.54	1164.62	1791.47
LDEC024632-RA	nadh dehydrogenase subunit 4	1.54	413.13	635.19
LDEC003530-RA	filamin-a isoform x1	1.52	305.85	465.39
LDEC007522-RA	leucine-rich repeat protein soc-2 isoform x1	1.52	474.30	718.78
LDEC001326-RA	ornithine decarboxylase antizyme partial	1.51	466.93	705.55
LDEC002253-RA	protein fam151b isoform x1	1.51	323.34	488.07
LDEC003545-RA	isoform c	1.51	381.14	575.03
LDEC008387-RA	homolog of	1.51	334.04	503.64
LDEC018226-RA	glycoside hydrolase family 1	1.50	625.02	940.61
LDEC016408-RA	sestrin homolog	1.50	376.44	566.07
LDEC000701-RA	cytochrome p450	1.50	3111.06	4665.55
LDEC005599-RA	amidophosphoribosyltransferase	1.49	292.93	437.46
LDEC005882-RA	probable aconitate mitochondrial	1.49	372.54	554.85
LDEC008684-RA	probable phosphoserine aminotransferase	1.49	3562.40	5301.41
LDEC000772-RA	sparc	1.49	294.35	437.43
LDEC001678-RA	glycoside hydrolase family 28 protein	1.48	284.42	421.01
LDEC011020-RA	lim and sh3 domain protein lasp	1.48	327.38	484.57
LDEC001049-RA	aminopeptidase n	1.48	1301.98	1922.06
LDEC001024-RA	vacuolar atp synthase subunit s1	1.47	328.72	484.53
LDEC011577-RA	ribosomal protein l7ae	1.47	593.31	873.02
LDEC016089-RA	collagen alpha- chain	1.46	406.81	592.33
LDEC000826-RA	spectrin alpha non-erythrocytic 1 isoform x4	1.44	352.10	508.17
LDEC018424-RA	ribonuclease x25	1.44	457.66	658.97
LDEC000780-RA	protein takeout	1.43	1130.35	1615.51
LDEC002775-RA	S-antigen protein, putative	1.41	398.20	562.82
LDEC000760-RA	aldose reductase	1.41	476.20	672.88
LDEC021669-RA	40s ribosomal protein s2	1.41	404.24	568.94
LDEC024631-RA	nadh dehydrogenase subunit 5	1.40	1411.63	1979.65
LDEC007413-RA	heat shock protein 70	1.40	2204.57	3086.42
LDEC000445-RA	longitudinals lacking isoform 4	1.39	902.54	1258.38
LDEC001677-RA	glycoside hydrolase family 28 protein	1.39	530.34	738.14
LDEC001692-RA	fatty acid synthase-like isoform x3	1.39	743.27	1033.55
LDEC010151-RA	v-type proton atpase 16 kda proteolipid subunit	1.38	1068.37	1478.09
LDEC005654-RA	chitin-binding protein	1.36	2317.98	3157.37
LDEC001198-RA	heat shock protein 90	1.35	943.23	1275.55
LDEC020740-RA	atp synthase subunit mitochondrial-like	1.32	1101.12	1457.42
LDEC017506-RA	importin-5	1.32	703.25	927.31
LDEC024629-RA	cytochrome c oxidase subunit iii	1.32	5727.33	7551.51
LDEC024633-RA	nadh dehydrogenase subunit 4	1.30	1497.99	1947.51

LDEC014178-RA	atp synthase lipid-binding mitochondrial	1.28	1318.09	1683.00
LDEC003397-RA	bifunctional purine biosynthesis protein purh	1.22	1476.61	1801.91
LDEC009708-RA	low quality protein: short form-like	1.20	2346.49	2812.46
LDEC010793-RA	mature sequence toxin-like lfec	1.19	3477.56	4121.31
LDEC004552-RA	translation elongation factor 2	1.16	5820.35	6757.63
LDEC017005-RA	pollen-specific leucine-rich repeat extensin-like protein 1	1.15	3892.73	4476.24
LDEC021419-RA	---NA---	1.14	3386.63	3877.18
LDEC003437-RA	hypothetical protein YQE_12777, partial	1.13	5613.26	6319.10
LDEC018144-RA	myosin heavy chain	-1.10	8374.40	7579.47
LDEC020822-RA	muscle actin	-1.17	5352.32	4582.48
LDEC005827-RA	PREDICTED: uncharacterized protein LOC103568218 isoform X3	-1.18	2233.61	1889.65
LDEC003990-RA	60s ribosomal protein l23a	-1.22	1798.81	1468.96
LDEC003957-RA	hypothetical protein D910_10187	-1.25	1582.88	1267.52
LDEC000226-RA	tyrosine hydroxylase	-1.26	2536.90	2019.71
LDEC007013-RA	ribosomal protein l13	-1.26	1280.59	1013.06
LDEC004014-RA	nucleoside diphosphate kinase	-1.27	1183.11	933.78
LDEC005396-RA	tubulin alpha-1 chain	-1.30	3518.67	2696.91
LDEC002600-RA	ribosomal protein s18	-1.32	1137.62	862.65
LDEC008741-RA	tubby-related protein 1	-1.33	805.87	607.06
LDEC003053-RA	translationally-controlled tumor protein homolog	-1.33	1538.16	1152.28
LDEC008084-RA	40s ribosomal protein s16	-1.34	806.10	599.73
LDEC007802-RA	apoptosis 1 inhibitor	-1.35	1270.52	941.89
LDEC014400-RA	cuticular protein 92f	-1.35	667.98	494.80
LDEC005786-RA	nesprin-1 isoform x1	-1.36	1124.46	828.21
LDEC005085-RA	40s ribosomal protein s10	-1.37	1158.63	844.10
LDEC010190-RA	60s ribosomal protein l13a	-1.38	950.88	688.60
LDEC022744-RA	40s ribosomal protein s14	-1.38	797.74	577.08
LDEC005287-RA	ferritin 2	-1.38	807.10	583.17
LDEC016655-RA	40s ribosomal protein s12	-1.39	752.49	542.50
LDEC018545-RA	troponin i	-1.39	770.03	552.13
LDEC005679-RA	cuticular protein ld-cp1v1	-1.40	693.22	495.68
LDEC011542-RA	ribosomal protein l14	-1.42	803.73	565.19
LDEC016254-RA	myosin light chain 2	-1.42	1050.36	737.51
LDEC005894-RA	40s ribosomal protein s5	-1.43	1031.35	722.04
LDEC014064-RA	ferritin subunit	-1.43	1300.70	907.98
LDEC011549-RA	hypothetical protein TcasGA2_TC003147	-1.43	562.75	392.27
LDEC015377-RA	40s ribosomal protein s25	-1.46	440.13	301.99
LDEC002760-RA	chitin deacetylase 2 isoform b precursor	-1.48	951.59	643.54
LDEC016749-RA	integumentary mucin -like isoform x1	-1.49	887.43	597.50
LDEC006479-RA	muscle lim protein mlp84b-like isoform x1	-1.49	1538.87	1034.29

LDEC003962-RA	---NA---	-1.50	1686.35	1124.75
LDEC010761-RA	6-phosphogluconate decarboxylating	-1.51	379.52	251.84
LDEC006314-RA	60s ribosomal protein l30	-1.51	556.76	368.98
LDEC003400-RA	myosin light chain alkali isoform x1	-1.51	896.98	592.79
LDEC006014-RA	ribosomal protein l10ae	-1.51	617.70	408.09
LDEC024230-RA	cuticular protein 13	-1.52	1579.41	1040.52
LDEC008220-RA	60s ribosomal protein l28	-1.52	748.50	492.67
LDEC000982-RA	40s ribosomal protein s2	-1.53	911.43	594.77
LDEC006427-RA	odorant binding protein 1	-1.55	498.87	322.05
LDEC006966-RA	reverse partial	-1.55	1042.61	670.90
LDEC009934-RA	40s ribosomal protein s3a	-1.56	1839.78	1178.86
LDEC003276-RA	28 kda heat- and acid-stable phosphoprotein	-1.56	349.44	223.70
LDEC008467-RA	60s ribosomal protein l44	-1.57	345.40	219.85
LDEC003614-RA	60s ribosomal protein l32-like	-1.57	1350.13	857.30
LDEC019172-RA	isoform c	-1.58	677.44	428.16
LDEC012940-RA	PREDICTED: LOW QUALITY PROTEIN: uncharacterized protein LOC658528	-1.62	986.62	608.44
LDEC014063-RA	ferritin 2	-1.63	1102.93	676.35
LDEC008233-RA	protein translation factor sui1 homolog	-1.66	292.21	176.32
LDEC009889-RA	yellow-e precursor	-1.66	379.29	227.99
LDEC011311-RA	abhydrolase domain-containing protein 3-like	-1.66	1237.10	743.49
LDEC002424-RA	40s ribosomal protein s19a-like	-1.67	817.65	489.70
LDEC024223-RA	c-1-tetrahydrofolate cytoplasmic	-1.67	527.91	315.79
LDEC013915-RA	elongation of very long chain fatty acids protein 1-like	-1.67	290.22	173.56
LDEC016748-RA	hypothetical protein D910_08753	-1.71	296.87	173.56
LDEC006478-RA	muscle protein 20-like protein	-1.73	3160.25	1822.30
LDEC007608-RA	ribosomal protein l35	-1.74	382.99	220.27
LDEC002231-RA	ribosomal protein l22	-1.75	532.24	304.04
LDEC004351-RA	microtubule-associated protein futsch isoform x5	-1.75	240.36	137.21
LDEC000558-RA	ribosomal protein l27ae	-1.75	396.64	226.39
LDEC011884-RA	antichymotrypsin-2-like isoform x2	-1.75	422.78	241.15
LDEC015047-RA	multiple inositol polyphosphate phosphatase 1	-1.76	217.07	123.12
LDEC014316-RA	serine palmitoyltransferase 1	-1.76	291.02	165.03
LDEC017904-RA	---NA---	-1.78	512.56	287.97
LDEC023320-RA	c-1-tetrahydrofolate cytoplasmic isoform x2	-1.79	615.99	343.22
LDEC020511-RA	eukaryotic translation initiation factor 1a	-1.80	274.63	152.57
LDEC015532-RA	glycine-rich protein	-1.80	1177.74	653.95
LDEC004820-RA	rna-binding protein nova-1 isoform x2	-1.81	714.42	395.07
LDEC004864-RA	40s ribosomal protein s15	-1.81	277.15	152.82
LDEC017171-RA	galactose-specific c-type lectin	-1.83	475.06	259.73
LDEC021798-RA	gamma-interferon-inducible	-1.84	391.98	212.91

	lysosomal thiol reductase-like			
LDEC020734-RA	aquaporin -like	-1.85	254.00	137.14
LDEC013180-RA	chemosensory protein 6	-1.86	611.80	328.14
LDEC013335-RA	elongation factor 1-alpha	-1.87	208.85	111.94
LDEC019779-RA	c-1-tetrahydrofolate cytoplasmic isoform x2	-1.87	5026.93	2681.87
LDEC014917-RA	ribosomal protein l36e	-1.89	261.27	138.13
LDEC005871-RA	60s ribosomal protein l37a	-1.91	408.85	214.43
LDEC013113-RA	ribosomal protein l37e	-1.91	348.77	182.62
LDEC020053-RA	apoptosis-inducing factor mitochondrial	-1.94	181.42	93.68
LDEC017905-RA	protoheme ix mitochondrial-like	-1.94	1035.91	532.73
LDEC000877-RA	nadp-dependent malic enzyme isoform x2	-1.95	183.61	94.35
LDEC001389-RA	gaba-gated ion channel	-1.96	435.85	222.64
LDEC014808-RA	dna topoisomerase mitochondrial isoform x3	-1.97	239.07	121.46
LDEC012229-RA	hypothetical protein TcasGA2_TC001876	-1.98	172.82	87.45
LDEC014488-RA	pdz and lim domain protein 1 isoform x2	-2.00	189.45	94.67
LDEC002259-RA	protein takeout	-2.01	186.13	92.83
LDEC024126-RA	60s ribosomal protein l7a-like	-2.02	762.47	377.58
LDEC012071-RA	40s ribosomal protein s8	-2.03	187.17	91.98
LDEC002758-RA	chitin deacetylase 1 precursor	-2.04	1700.23	833.17
LDEC005521-RA	protein takeout-like	-2.05	509.66	248.69
LDEC018881-RA	differentially expressed in fdcp 6-like protein	-2.06	177.62	86.39
LDEC010494-RA	serine proteinase	-2.06	237.51	115.37
LDEC024395-RA	ribosomal protein s20	-2.07	194.25	93.75
LDEC014399-RA	cuticle protein	-2.07	171.44	82.64
LDEC014467-RA	hypothetical protein D910_05825	-2.08	172.39	82.71
LDEC003983-RA	rwd domain-containing protein 2a	-2.10	146.11	69.61
LDEC020749-RA	gamma-aminobutyric acid receptor-associated protein	-2.13	372.92	175.29
LDEC010707-RA	hypothetical protein D910_06287	-2.13	134.18	62.92
LDEC006934-RA	alpha beta hydrolase domain-containing protein 11	-2.16	212.93	98.70
LDEC003495-RA	tubulin-specific chaperone cofactor e-like protein	-2.17	242.45	111.76
LDEC007223-RA	thap domain-containing protein partial	-2.17	294.92	135.72
LDEC010005-RA	presequence mitochondrial	-2.18	294.11	134.87
LDEC010330-RA	PREDICTED: uncharacterized protein LOC656470	-2.19	530.34	242.14
LDEC015280-RA	troponin c ia	-2.20	544.79	247.66
LDEC000980-RA	dynactin subunit 5	-2.21	180.66	81.89
LDEC009353-RA	tubulin polyglutamylase ttll5	-2.21	384.42	173.91
LDEC000979-RA	60s ribosomal protein l31	-2.22	614.23	277.29
LDEC000758-RA	endo-beta- -glucanase	-2.22	445.12	200.45
LDEC020300-RA	peptidyl-prolyl cis-trans isomerase fkbp8	-2.24	130.75	58.43
LDEC010788-RA	peptide methionine sulfoxide reductase	-2.28	386.56	169.87

LDEC007589-RA	formin-like protein 8	-2.29	578.06	252.76
LDEC002059-RA	digestive cysteine proteinase intestain	-2.35	3673.24	1565.93
LDEC015677-RA	nadh dehydrogenase subunit 2	-2.35	179.47	76.41
LDEC000165-RA	cyclin-dependent kinase inhibitor 1b-like isoform x4	-2.35	123.53	52.59
LDEC010780-RA	chitooligosaccharidolytic beta-n-acetylglucosaminidase	-2.35	172.82	73.51
LDEC003837-RA	endonuclease and reverse transcriptase-like protein	-2.36	13842.89	5872.58
LDEC005505-RA	agap004519-pa-like protein	-2.37	136.17	57.55
LDEC010802-RA	atm interactor	-2.38	111.27	46.82
LDEC015541-RA	inositol oxygenase	-2.38	185.75	77.89
LDEC014332-RA	muscle-specific protein 20-like	-2.41	136.60	56.62
LDEC023974-RA	muscular protein 20	-2.41	153.57	63.63
LDEC014321-RA	isoform a	-2.42	245.49	101.61
LDEC010860-RA	g2 mitotic-specific cyclin-b	-2.42	115.21	47.64
LDEC018331-RA	polyadenylate-binding protein-interacting protein 1-like isoform 2	-2.42	159.13	65.76
LDEC001840-RA	alpha-tocopherol transfer	-2.43	131.85	54.22
LDEC017271-RA	esterase fe4	-2.46	189.41	77.08
LDEC000838-RA	#NAME?	-2.49	145.44	58.50
LDEC010502-RA	esterase	-2.52	123.81	49.19
LDEC019778-RA	c-1-tetrahydrofolate cytoplasmic	-2.52	1090.52	433.22
LDEC003301-RA	muscle m-line assembly protein unc-89-like	-2.53	384.85	152.18
LDEC017273-RA	juvenile hormone esterase	-2.55	180.52	70.85
LDEC010576-RA	---NA---	-2.57	245.30	95.41
LDEC010015-RA	counting factor associated protein d-like	-2.58	123.67	47.92
LDEC015177-RA	small heat shock protein	-2.60	296.78	113.99
LDEC000920-RA	glycosyl hydrolase	-2.60	125.19	48.06
LDEC017272-RA	venom carboxylesterase-6-like	-2.65	123.77	46.68
LDEC010935-RA	#NAME?	-2.68	140.97	52.63
LDEC000855-RA	fatty acid-binding protein	-2.69	592.98	220.59
LDEC013547-RA	cuticle protein 21	-2.74	440.36	160.43
LDEC000045-RA	ribosomal protein L39 [Danaus plexippus]	-2.75	252.57	91.87
LDEC014730-RA	lanc-like protein 3 homolog	-2.76	106.89	38.79
LDEC012958-RA	alkylglycerol monooxygenase-like	-2.77	572.83	206.61
LDEC021671-RA	cuticle protein 6	-2.82	132.23	46.93
LDEC016769-RA	cytochrome p450 412a1	-2.87	142.87	49.83
LDEC016722-RA	protein ctla-2-alpha	-2.89	87.22	30.19
LDEC001175-RA	tpa: cuticle protein	-2.90	222.91	76.83
LDEC006288-RA	elongation factor ts	-2.90	129.38	44.59
LDEC018350-RA	PREDICTED: uncharacterized protein LOC658581	-2.95	111.69	37.90
LDEC013548-RA	glycine-rich protein	-2.99	6069.97	2027.03
LDEC002747-RA	---NA---	-3.04	82.70	27.18
LDEC000801-RA	juvenile hormone-inducible protein	-3.07	431.00	140.36

LDEC007227-RA	a-kinase anchor protein 9-like isoform x1	-3.07	110.70	36.03
LDEC003163-RA	methylmalonic aciduria and homocystinuria type d mitochondrial isoform x1	-3.09	102.52	33.23
LDEC002262-RA	udp-glucuronosyltransferase 2b10-like	-3.12	130.90	41.97
LDEC000919-RA	glycosyl hydrolase	-3.12	112.74	36.10
LDEC008890-RA	odorant-binding protein 4	-3.14	93.68	29.83
LDEC005070-RA	glycine-rich protein	-3.18	2454.86	773.11
LDEC019170-RA	hypothetical protein D910_04523	-3.18	92.49	29.13
LDEC000604-RA	---NA---	-3.19	106.85	33.48
LDEC004652-RA	ubiquitin carboxyl-terminal hydrolase	-3.19	113.64	35.60
LDEC002105-RA	ubx domain-containing protein 4	-3.24	197.53	60.94
LDEC013853-RA	kinesin-like protein kif18a-like	-3.24	83.60	25.76
LDEC002538-RA	dynein light chain cytoplasmic	-3.27	182.04	55.63
LDEC011670-RA	succinate dehydrogenase assembly factor mitochondrial-like	-3.28	82.18	25.06
LDEC018698-RA	cytochrome p450 9z4	-3.39	156.23	46.04
LDEC002596-RA	vacuolar protein sorting-associated protein 52 homolog	-3.61	110.08	30.47
LDEC020175-RA	cytochrome p450 9z4	-3.69	99.76	27.04
LDEC016516-RA	venom allergen 3-like	-3.70	95.68	25.84
LDEC005354-RA	isoform d	-3.78	70.44	18.62
LDEC013889-RA	inactive pancreatic lipase-related protein 1	-3.83	106.85	27.92
LDEC003722-RA	prostaglandin reductase 1-like	-3.88	85.79	22.12
LDEC005610-RA	atrophin-1 isoform x1	-3.88	429.43	110.67
LDEC018613-RA	leucine-rich repeat-containing protein ddb_g0290503-like isoform x1	-3.89	129.23	33.20
LDEC023675-RA	cytochrome p450	-3.92	639.46	163.26
LDEC020428-RA	prostatic acid phosphatase-like	-3.98	126.33	31.78
LDEC000494-RA	---NA---	-4.00	369.88	92.37
LDEC009270-RA	venom acid phosphatase acph-1-like	-4.03	66.64	16.53
LDEC024010-RA	isoform a	-4.05	2110.60	521.27
LDEC013287-RA	cytochrome p450 9z4	-4.13	3099.70	750.46
LDEC010503-RA	juvenile hormone esterase isoform a	-4.15	122.82	29.62
LDEC021800-RA	cytochrome p450 9z4	-4.15	211.36	50.93
LDEC014701-RA	tbc1 domain family member 9	-4.20	102.76	24.49
LDEC007706-RA	cytochrome p450 4c1 isoform x2	-4.20	63.83	15.18
LDEC012660-RA	protein prenyltransferase alpha subunit repeat-containing protein 1-b	-4.25	571.73	134.56
LDEC003879-RA	dynein heavy chain axonemal	-4.33	194.35	44.91
LDEC001712-RA	inverted formin-2	-4.33	346.02	79.88
LDEC008894-RA	protein expanded	-4.35	73.10	16.81
LDEC021796-RA	gamma-interferon-inducible lysosomal thiol reductase-like	-4.43	462.70	104.54
LDEC020429-RA	prostatic acid phosphatase	-4.43	129.28	29.20
LDEC018558-RA	dentin sialophosphoprotein isoform	-4.47	130.09	29.13

	x3			
LDEC020758-RA	cytochrome p450 9e2	-4.47	400.30	89.47
LDEC012858-RA	camp-dependent protein kinase type ii regulatory subunit	-4.54	68.02	14.97
LDEC018630-RA	ubiquitin-like modifier-activating enzyme atg7-like isoform x3	-4.56	62.07	13.63
LDEC006908-RA	nad-dependent protein deacetylase sirt2	-4.58	124.67	27.22
LDEC022770-RA	cytochrome p450 9z4	-4.61	286.60	62.22
LDEC004614-RA	proton-coupled amino acid transporter 4-like	-4.63	104.95	22.69
LDEC023820-RA	cytochrome p450 9e2-like	-4.68	163.60	34.93
LDEC022674-RA	cytochrome p450 9z4	-4.76	590.46	124.04
LDEC003963-RA	atp-binding cassette sub-family b member mitochondrial	-4.79	58.79	12.28
LDEC001696-RA	phosphoglycerate mutase 1	-4.79	111.08	23.18
LDEC006907-RA	hth-type transcriptional repressor -like	-4.81	84.46	17.55
LDEC019169-RA	tyrosine 3-monooxygenase	-4.94	92.64	18.76
LDEC005068-RA	glycine-rich protein	-4.94	1597.09	323.12
LDEC020757-RA	cytochrome p450	-5.14	655.72	127.65
LDEC020176-RA	cytochrome p450 9z4	-5.14	3606.89	702.01
LDEC012705-RA	casein kinase ii subunit alpha	-5.19	140.12	27.00
LDEC006606-RA	cytochrome p450 9e2-like	-5.26	192.88	36.70
LDEC015015-RA	traf-interacting partial	-5.33	68.11	12.78
LDEC019798-RA	gastrula zinc finger protein	-5.48	222.44	40.56
LDEC022329-RA	cytochrome p450	-5.55	107.13	19.29
LDEC020604-RA	hypothetical protein D910_02954	-5.63	138.69	24.63
LDEC006967-RA	glyceraldehyde-3-phosphate dehydrogenase	-5.68	48.62	8.56
LDEC009046-RA	neuropilin and tolloid-like protein 2-like	-5.76	51.95	9.02
LDEC007768-RA	potassium sodium hyperpolarization-activated cyclic nucleotide-gated channel 1-like	-6.16	129.33	20.99
LDEC021799-RA	cytochrome p450 9z4	-6.18	113.64	18.40
LDEC006994-RA	calpain-b isoform x2	-6.22	69.11	11.11
LDEC002055-RA	digestive cysteine protease intestain	-6.27	755.87	120.65
LDEC000854-RA	fatty acid-binding	-6.59	323.63	49.09
LDEC011194-RA	heat shock protein 68	-6.86	65.59	9.56
LDEC015188-RA	1-acylglycerol-3-phosphate o-acyltransferase abhd5-like isoform x2	-6.96	70.68	10.16
LDEC018375-RA	enkurin domain-containing protein 1 isoform x1	-7.04	58.32	8.28
LDEC014237-RA	dentin sialophospho isoform x1	-7.32	66.59	9.10
LDEC003956-RA	cuticle protein 63	-7.37	152.81	20.74
LDEC013578-RA	serine threonine-protein phosphatase 4 regulatory subunit 1-like isoform x3	-7.53	114.83	15.25
LDEC001153-RA	gpi-anchored wall transfer protein 1	-7.69	84.94	11.04
LDEC011227-RA	hypothetical protein YQE_05602, partial	-7.81	38.40	4.92
LDEC004370-RA	testis-specific serine threonine-protein kinase 1-like	-8.22	98.67	12.00

LDEC008892-RA	transposable element tc3 transposase	-8.23	38.74	4.71
LDEC003189-RA	nadh dehydrogenase	-9.09	246.82	27.14
LDEC020903-RA	multiple ankyrin repeats single kh domain protein	-9.18	39.64	4.32
LDEC010726-RA	15-hydroxyprostaglandin dehydrogenase	-9.27	165.31	17.84
LDEC013721-RA	ef-hand calcium-binding domain-containing protein 1	-9.43	59.41	6.30
LDEC016420-RA	esterase	-9.98	55.09	5.52
LDEC011333-RA	kinesin-like protein kif16b	-10.15	38.78	3.82
LDEC004896-RA	---NA---	-10.19	48.34	4.74
LDEC015711-RA	upf0573 protein c2orf70 homolog	-10.22	49.57	4.85
LDEC009417-RA	adenosine deaminase cecr1-like	-10.48	42.30	4.03
LDEC001186-RA	eh domain-containing protein 3	-10.88	50.43	4.64
LDEC009369-RA	---NA---	-10.94	174.58	15.96
LDEC004032-RA	toxin 1	-11.00	88.79	8.07
LDEC008895-RA	odorant-binding protein 4	-11.01	58.46	5.31
LDEC003838-RA	---NA---	-11.20	694.46	62.00
LDEC012861-RA	alcohol dehydrogenase	-11.85	100.24	8.46
LDEC018530-RA	transcriptional regulator atrx homolog	-11.89	37.88	3.19
LDEC021048-RA	alpha-tubulin n-acetyltransferase-like	-12.43	291.69	23.46
LDEC005012-RA	dynein intermediate chain ciliary	-12.73	43.25	3.40
LDEC009036-RA	ef-hand domain-containing protein 1-like	-13.36	80.85	6.05
LDEC006990-RA	calpain-a-like isoform 1	-13.95	43.44	3.11
LDEC004124-RA	hypothetical protein YQE_08744, partial	-14.31	35.46	2.48
LDEC008529-RA	ropporin-1-like protein	-14.51	54.42	3.75
LDEC002000-RA	sulfotransferase 1c4-like	-14.59	103.23	7.08
LDEC006841-RA	b-box type zinc finger protein ncl-1-like isoform x1	-14.64	32.13	2.19
LDEC010652-RA	coiled-coil domain-containing protein 61-like	-15.29	61.69	4.03
LDEC000464-RA	coiled-coil domain-containing protein 108-like isoform x3	-15.65	54.28	3.47
LDEC020747-RA	hypothetical protein YQE_11871, partial	-15.76	31.23	1.98
LDEC001263-RA	extracellular signal-regulated kinase 2	-17.82	66.21	3.72
LDEC001338-RA	tektin-3-like	-17.84	53.04	2.97
LDEC000528-RA	PREDICTED: uncharacterized protein LOC100881963	-18.21	46.39	2.55
LDEC010199-RA	tubulin glycylase 3a-like	-18.28	91.21	4.99
LDEC015389-RA	leucine-rich repeat-containing protein 48	-18.65	46.86	2.51
LDEC009877-RA	retinol dehydrogenase 13	-19.45	158.99	8.18
LDEC000115-RA	rib43a-like with coiled-coils protein 2	-19.55	35.98	1.84
LDEC002154-RA	radial spoke head protein 3 homolog	-21.22	66.83	3.15
LDEC016911-RA	c-jun-amino-terminal kinase-interacting	-22.43	76.19	3.40
LDEC000564-RA	kinesin-like protein kif9	-22.53	125.19	5.56
LDEC009511-RA	cilia- and flagella-associated protein	-23.75	42.87	1.80

	69-like			
LDEC010196-RA	tubulin glycylase 3a-like isoform x2	-23.87	227.24	9.52
LDEC017395-RA	coiled-coil domain-containing protein 42 homolog	-24.29	248.44	10.23
LDEC002210-RA	ciliary dynein heavy	-25.28	85.89	3.40
LDEC013433-RA	transmembrane protein 223	-25.46	69.39	2.73
LDEC002699-RA	protein maats1-like	-25.56	59.70	2.34
LDEC002923-RA	nucleic-acid-binding protein from transposon x-element	-25.75	85.65	3.33
LDEC005036-RA	dynein heavy chain axonemal	-25.99	82.80	3.19
LDEC016339-RA	centrosomal protein of 290 kda	-26.04	33.18	1.27
LDEC012220-RA	fk506-binding protein 5-like isoform x4	-26.33	81.99	3.11
LDEC010538-RA	hydrocephalus-inducing protein homolog	-26.41	44.87	1.70
LDEC015400-RA	hypothetical protein D910_05843	-26.96	88.74	3.29
LDEC013714-RA	probable multidrug resistance-associated protein lethal 03659	-27.57	31.23	1.13
LDEC018097-RA	polyadenylate-binding protein 1-like isoform 1	-27.70	91.16	3.29
LDEC022883-RA	digestive cysteine protease intestain	-28.56	787.28	27.57
LDEC001008-RA	succinate dehydrogenase	-29.63	53.47	1.80
LDEC015048-RA	26s proteasome non-atpase regulatory subunit 2	-29.80	71.72	2.41
LDEC013315-RA	forkhead box protein n5-like isoform x3	-31.43	82.32	2.62
LDEC002004-RA	e3 ubiquitin-protein ligase march2-like	-31.98	65.64	2.05
LDEC012407-RA	tetratricopeptide repeat protein 25-like	-35.89	41.92	1.17
LDEC019899-RA	protein claret segregational	-36.07	62.55	1.73
LDEC001845-RA	alpha-tocopherol transfer	-39.02	524.82	13.45
LDEC010109-RA	hypothetical protein YQE_12170, partial	-39.58	50.43	1.27
LDEC013867-RA	ring finger protein 145	-40.19	167.83	4.18
LDEC009941-RA	phosphoglycerate kinase	-40.26	447.44	11.11
LDEC007302-RA	glutamine synthetase 2	-40.44	62.98	1.56
LDEC015049-RA	26s proteasome non-atpase regulatory subunit 2	-40.51	53.04	1.31
LDEC016529-RA	coiled-coil domain-containing protein 65	-40.91	37.64	0.92
LDEC015155-RA	unknown	-41.10	88.74	2.16
LDEC011150-RA	lish domain-containing protein fopnl-like	-41.63	39.78	0.96
LDEC017402-RA	centromere-associated protein e-like	-41.97	35.65	0.85
LDEC023002-RA	pupal cuticle protein 20	-42.00	59.46	1.42
LDEC010535-RA	hydrocephalus-inducing protein homolog	-42.77	39.35	0.92
LDEC004151-RA	polycystic kidney disease protein 1-like 2	-43.59	128.05	2.94
LDEC007298-RA	wd repeat-containing protein 34-like	-44.58	41.02	0.92
LDEC003232-RA	ammonium transporter 1 member 3-like	-45.92	50.38	1.10
LDEC006746-RA	gamma-glutamyltranspeptidase 1-like isoform x2	-46.64	54.47	1.17
LDEC004733-RA	glucose dehydrogenase	-49.07	109.41	2.23

LDEC003361-RA	proton-coupled amino acid transporter 4 isoform x1	-50.51	119.77	2.37
LDEC022047-RA	cyclin-dependent kinase-like 4 isoform x1	-50.70	43.06	0.85
LDEC018396-RA	dynein heavy chain	-51.34	218.02	4.25
LDEC015154-RA	PREDICTED: uncharacterized protein LOC103312842	-52.59	94.92	1.80
LDEC011224-RA	hypothetical protein TcasGA2_TC015485	-56.65	56.13	0.99
LDEC018389-RA	dynein beta ciliary-like isoform x2	-56.70	136.46	2.41
LDEC013593-RA	hypothetical protein D910_04991	-56.93	36.27	0.64
LDEC000703-RA	antifreeze protein maxi	-58.32	111.46	1.91
LDEC018390-RA	dynein heavy chain	-60.20	36.22	0.60
LDEC006520-RA	transcription factor sox-10-like isoform x2	-60.39	70.53	1.17
LDEC013847-RA	PREDICTED: uncharacterized protein LOC103307735	-60.67	36.50	0.60
LDEC003208-RA	perq amino acid-rich with gyf domain-containing protein 2-like	-61.64	229.05	3.72
LDEC002625-RA	major antigen-like	-63.88	108.51	1.70
LDEC006293-RA	dynein heavy chain axonemal	-64.56	31.99	0.50
LDEC015606-RA	cystine knot toxin	-64.65	114.40	1.77
LDEC005192-RA	hypothetical protein D910_12530	-65.09	34.55	0.53
LDEC012441-RA	morn repeat-containing protein 5-like	-65.67	44.16	0.67
LDEC003214-RA	hypothetical protein D910_04081	-66.00	32.70	0.50
LDEC021135-RA	4-aminobutyrate mitochondrial	-67.22	45.20	0.67
LDEC007016-RA	biogenesis of lysosome-related organelles complex 1 subunit 2-like	-67.76	148.67	2.19
LDEC018239-RA	hypothetical protein TcasGA2_TC012239	-68.49	33.94	0.50
LDEC018234-RA	golgin subfamily a member 4-like	-71.26	40.35	0.57
LDEC014993-RA	mitochondrial import inner membrane translocase subunit tim50-c	-72.85	139.21	1.91
LDEC013105-RA	interferon regulatory factor 2-binding isoform x1	-73.62	114.64	1.56
LDEC016023-RA	zinc finger and btb domain-containing protein 24 isoform x2	-74.22	49.91	0.67
LDEC006138-RA	PREDICTED: uncharacterized protein LOC103315014 isoform X1	-76.19	132.13	1.73
LDEC002598-RA	PREDICTED: uncharacterized protein LOC103313140	-79.05	58.75	0.74
LDEC017760-RA	aael004257- partial	-81.83	43.44	0.53
LDEC016663-RA	aaa atpase	-83.39	32.46	0.39
LDEC014077-RA	isoform e	-84.16	35.74	0.42
LDEC022024-RA	mucin related isoform a	-84.25	143.11	1.70
LDEC024113-RA	ring finger protein partial	-85.15	45.20	0.53
LDEC022714-RA	hypothetical protein TcasGA2_TC001927	-85.25	63.36	0.74
LDEC015839-RA	mitogen-activated protein kinase erk-	-85.83	33.41	0.39
LDEC007936-RA	myotubularin	-86.52	159.22	1.84
LDEC017040-RA	tubulin alpha chain-like	-88.12	137.22	1.56
LDEC014883-RA	coiled-coil domain-containing protein 147-like	-88.41	93.87	1.06
LDEC011223-RA	na+ + atpase alpha-subunit partial	-88.55	50.14	0.57

LDEC002028-RA	outer dense fiber protein 2-like	-91.63	230.23	2.51
LDEC004064-RA	malate dehydrogenase	-92.95	62.50	0.67
LDEC003242-RA	kinesin-like protein klp10a	-95.52	54.09	0.57
LDEC009902-RA	dynein heavy chain axonemal	-97.32	51.66	0.53
LDEC009664-RA	PREDICTED: uncharacterized protein LOC103312201	-98.71	55.89	0.57
LDEC015289-RA	endothelin-converting enzyme 1-like	-99.38	28.14	0.28
LDEC004267-RA	hypothetical protein D910_07448	-105.16	74.43	0.71
LDEC015275-RA	wd repeat-containing protein 66-like	-105.38	55.94	0.53
LDEC003746-RA	4-coumarate-- ligase 1-like	-106.63	37.74	0.35
LDEC005954-RA	probable inactive protein kinase ddb_g0270444-like isoform x2	-110.13	31.18	0.28
LDEC021939-RA	aldose 1-epimerase	-112.20	95.30	0.85
LDEC016080-RA	coiled-coil domain-containing protein 37	-112.48	31.84	0.28
LDEC002564-RA	ribonuclease h2 subunit c-like	-113.86	36.27	0.32
LDEC000087-RA	tetratricopeptide repeat protein 18-like	-114.32	32.37	0.28
LDEC017041-RA	tubulin alpha chain	-117.11	41.45	0.35
LDEC002739-RA	tektin-b1-like isoform x2	-119.53	38.07	0.32
LDEC003467-RA	e3 ubiquitin-protein ligase trim37-like isoform x2	-120.20	59.55	0.50
LDEC006129-RA	hypothetical protein YQE_09365, partial	-121.83	30.18	0.25
LDEC017641-RA	malate mitochondrial	-126.06	231.99	1.84
LDEC003690-RA	odorant binding protein	-126.47	26.85	0.21
LDEC009175-RA	coiled-coil domain-containing protein 113-like	-128.42	36.36	0.28
LDEC001986-RA	upf0704 protein c6orf165 homolog	-131.88	70.01	0.53
LDEC001846-RA	hypothetical protein YQE_08339, partial	-135.67	417.74	3.08
LDEC009060-RA	upf0605 protein cg18335-like	-139.22	59.13	0.42
LDEC010337-RA	circumsporozoite protein	-140.51	79.56	0.57
LDEC001963-RA	leucine-rich repeat-containing protein 51-like	-142.36	105.80	0.74
LDEC015764-RA	upf0704 protein c6orf165 homolog	-142.53	80.71	0.57
LDEC011040-RA	PREDICTED: uncharacterized protein LOC103310485	-142.61	80.75	0.57
LDEC020593-RA	PREDICTED: uncharacterized protein LOC100574550	-144.37	30.66	0.21
LDEC002546-RA	hypothetical protein YQE_05003, partial	-146.23	87.98	0.60
LDEC006291-RA	dynein heavy chain axonemal	-149.20	58.08	0.39
LDEC013333-RA	coiled-coil domain-containing protein 39	-149.74	42.40	0.28
LDEC002185-RA	flagellar attachment zone protein 1	-150.19	95.68	0.64
LDEC024104-RA	PREDICTED: uncharacterized protein LOC100142328	-153.01	81.23	0.53
LDEC003915-RA	ubiquitin carboxyl-terminal	-156.69	6382.91	40.73
LDEC003363-RA	hypothetical protein YQE_07867, partial	-157.65	72.53	0.46
LDEC007037-RA	PREDICTED: uncharacterized protein LOC103312559	-160.94	34.17	0.21
LDEC007368-RA	hypothetical protein D910_09223	-164.20	87.17	0.53
LDEC010917-RA	hypothetical protein D910_09343	-164.89	105.04	0.64

LDEC019915-RA	Rm62	-166.53	35.36	0.21
LDEC004651-RA	dynein heavy chain	-171.47	224.53	1.31
LDEC001951-RA	glyceraldehyde-3-phosphate dehydrogenase	-174.74	55.66	0.32
LDEC012371-RA	phosphatidate phosphatase ppapdc1a-like	-176.13	87.26	0.50
LDEC006130-RA	hypothetical protein D910_09009	-177.72	56.61	0.32
LDEC014665-RA	serine threonine-protein kinase paka-like	-180.33	70.20	0.39
LDEC013649-RA	---NA---	-185.00	52.38	0.28
LDEC010102-RA	talin-1 isoform x1	-186.60	118.87	0.64
LDEC008626-RA	hypothetical protein YQE_11743, partial	-194.40	137.60	0.71
LDEC005803-RA	tubulin beta-1 chain	-197.21	1305.17	6.62
LDEC019428-RA	PREDICTED: uncharacterized protein LOC103314495	-199.10	112.74	0.57
LDEC001878-RA	lim-like muscle protein	-199.12	133.89	0.67
LDEC010155-RA	methyltransferase nsun7	-199.21	42.30	0.21
LDEC001650-RA	atp synthase subunit mitochondrial-like	-200.78	28.42	0.14
LDEC007675-RA	---NA---	-202.01	257.37	1.27
LDEC006333-RA	tetratricopeptide repeat protein 39c-like	-202.03	100.10	0.50
LDEC006736-RA	protein efr3-like protein a	-203.89	79.37	0.39
LDEC022403-RA	cathepsin b-like proteinase	-203.99	64.97	0.32
LDEC021001-RA	collagen alpha-1	-207.01	51.28	0.25
LDEC003152-RA	protein disulfide-isomerase a3	-210.85	37.31	0.18
LDEC008267-RA	sperm-associated antigen 6-like	-211.97	45.01	0.21
LDEC022648-RA	cytochrome oxidase subunit partial	-214.34	75.86	0.35
LDEC020587-RA	isoform b	-224.95	31.84	0.14
LDEC010002-RA	oxidative stress-induced growth inhibitor 2	-228.65	32.37	0.14
LDEC019296-RA	kelch-like protein 10-like	-229.88	48.81	0.21
LDEC013810-RA	coiled-coil domain-containing protein 96	-230.66	32.65	0.14
LDEC016106-RA	tripartite motif-containing protein 71-like	-238.61	50.67	0.21
LDEC002713-RA	f-box only protein 39-like	-240.69	272.58	1.13
LDEC000704-RA	antifreeze protein maxi	-241.00	170.58	0.71
LDEC003466-RA	e3 ubiquitin-protein ligase trim37-like	-246.89	52.43	0.21
LDEC009896-RA	PREDICTED: uncharacterized protein LOC103312770	-249.46	35.31	0.14
LDEC008422-RA	dentin sialophosphoprotein	-250.47	35.46	0.14
LDEC011420-RA	retinitis pigmentosa 1-like 1 protein	-251.48	35.60	0.14
LDEC010357-RA	hypothetical protein YQE_00629, partial	-256.13	63.45	0.25
LDEC016446-RA	iq domain-containing protein g-like	-2605	26.05	0.00
LDEC013722-RA	ef-hand calcium-binding domain-containing protein 1	-2704	27.04	0.00
LDEC003635-RA	protein lap4	-2733	27.33	0.00
LDEC005166-RA	fact complex subunit ssrp1-like	-273.66	125.91	0.46
LDEC009292-RA	PREDICTED: uncharacterized protein LOC103314814	-2742	27.42	0.00

LDEC017197-RA	v-type proton atpase subunit f 1-like	-2757	27.57	0.00
LDEC017830-RA	---NA---	-277.55	88.41	0.32
LDEC000508-RA	cytosolic non-specific dipeptidase-like	-282.25	59.93	0.21
LDEC012331-RA	ubiquitin-protein ligase	-2842	28.42	0.00
LDEC018212-RA	PREDICTED: uncharacterized protein LOC103314845	-288.75	102.19	0.35
LDEC001261-RA	hypothetical protein D910_07352	-289.75	41.02	0.14
LDEC007831-RA	coiled-coil domain-containing protein 151-like	-290.89	51.47	0.18
LDEC019661-RA	ef-hand calcium-binding domain-containing protein 1	-291.10	41.21	0.14
LDEC014383-RA	abnormal spindle-like microcephaly-associated protein homolog	-2928	29.28	0.00
LDEC008403-RA	kelch-like protein 10-like	-2937	29.37	0.00
LDEC011981-RA	protein nedd1	-294.12	62.45	0.21
LDEC002623-RA	major antigen-like	-295.10	543.07	1.84
LDEC010003-RA	hypothetical protein KGM_06951	-2956	29.56	0.00
LDEC016739-RA	dynein heavy chain	-2956	29.56	0.00
LDEC004353-RA	wd repeat-containing protein 16	-297.07	52.57	0.18
LDEC010555-RA	soluble nsf attachment protein	-299.49	42.40	0.14
LDEC000139-RA	heat shock cognate 71 kda isoform x1	-300.24	95.63	0.32
LDEC002616-RA	agap012275-pa-like protein	-3037	30.37	0.00
LDEC008627-RA	e3 ubiquitin-protein ligase ubr3	-306.65	32.56	0.11
LDEC008989-RA	protein disulfide-isomerase	-3089	30.89	0.00
LDEC016539-RA	vesicle-associated membrane protein-associated protein b	-3146	31.46	0.00
LDEC016445-RA	hypothetical protein TcasGA2_TC006984	-318.05	123.81	0.39
LDEC005660-RA	regulator of nonsense transcripts 2	-322.99	91.45	0.28
LDEC005889-RA	hypothetical protein TcasGA2_TC005656	-325.54	57.61	0.18
LDEC014713-RA	wd repeat domain 61	-326.13	69.25	0.21
LDEC010198-RA	tubulin tyrosine ligase-like 3a	-327.47	69.54	0.21
LDEC011849-RA	outer dense fiber protein 3	-328.87	186.22	0.57
LDEC001248-RA	cytochrome c oxidase subunit mitochondrial	-3346	33.46	0.00
LDEC013055-RA	cytochrome b-c1 complex subunit 8	-3427	34.27	0.00
LDEC023634-RA	nadh dehydrogenase	-3479	34.79	0.00
LDEC018784-RA	short transient receptor potential channel 5-like	-349.85	49.53	0.14
LDEC014121-RA	cytochrome c-2	-3546	35.46	0.00
LDEC010331-RA	leucine-rich repeat-containing protein 23-like	-356.57	50.48	0.14
LDEC021105-RA	tubulin polyglutamylase ttll6	-359.92	152.86	0.42
LDEC011874-RA	kelch repeat and btb domain-containing protein 12	-3622	36.22	0.00
LDEC007110-RA	agap001754-pa-like protein	-3669	36.69	0.00
LDEC013782-RA	succinate dehydrogenase	-3674	36.74	0.00
LDEC017772-RA	serine protease 53-like	-369.33	26.14	0.07
LDEC014943-RA	l-2-hydroxyglutarate mitochondrial-like	-369.86	65.45	0.18

LDEC001340-RA	vitellogenin	-370.45	78.66	0.21
LDEC013117-RA	tctex1 domain-containing protein 1-like	-3707	37.07	0.00
LDEC021361-RA	proteasome-associated protein ecm29 homolog	-376.04	53.23	0.14
LDEC007702-RA	methyltransferase wbscr22	-378.06	53.52	0.14
LDEC008302-RA	sorbitol dehydrogenase	-3788	37.88	0.00
LDEC008942-RA	PREDICTED: uncharacterized protein LOC661884	-379.32	120.82	0.32
LDEC004567-RA	map microtubule affinity-regulating kinase 3-like	-381.86	40.54	0.11
LDEC007308-RA	tektin-1	-384.99	40.88	0.11
LDEC009813-RA	testicular haploid expressed gene	-3916	39.16	0.00
LDEC002651-RA	probable pseudouridine-5 -monophosphatase isoform x2	-392.60	41.68	0.11
LDEC003286-RA	regulator of microtubule dynamics protein 1-like isoform x2	-3935	39.35	0.00
LDEC016809-RA	radial spoke head protein 4 homolog a-like	-395.74	42.02	0.11
LDEC019079-RA	hypothetical protein YQE_12002, partial	-398.87	56.47	0.14
LDEC002162-RA	small subunit processome component 20-like protein	-406.03	86.22	0.21
LDEC008402-RA	kelch-like protein 10-like	-4092	40.92	0.00
LDEC002063-RA	hypothetical protein TcasGA2_TC012351	-425.73	30.13	0.07
LDEC009193-RA	hypothetical protein TcasGA2_TC003077	-4278	42.78	0.00
LDEC008431-RA	thap domain-containing protein 4-like	-4335	43.35	0.00
LDEC016767-RA	testis-specific serine threonine-protein kinase 4-like	-436.48	61.79	0.14
LDEC007890-RA	PREDICTED: uncharacterized protein C1orf177 homolog	-4392	43.92	0.00
LDEC017555-RA	aspartate mitochondrial	-4430	44.30	0.00
LDEC002486-RA	15-hydroxyprostaglandin dehydrogenase	-4444	44.44	0.00
LDEC017901-RA	cytochrome c oxidase subunit va	-4477	44.77	0.00
LDEC009496-RA	---NA---	-4492	44.92	0.00
LDEC024265-RA	hypothetical protein D910_02300	-4496	44.96	0.00
LDEC018180-RA	kelch-like protein 10	-457.29	97.10	0.21
LDEC010156-RA	methyltransferase nsun7	-457.52	48.58	0.11
LDEC010107-RA	agap009741-pa-like protein	-460.65	32.61	0.07
LDEC019463-RA	protein mcm10 homolog	-4796	47.96	0.00
LDEC005124-RA	tetratricopeptide repeat protein 19	-480.80	85.08	0.18
LDEC011362-RA	leucine-rich repeat-containing protein ddb_g0290503	-481.60	255.66	0.53
LDEC015696-RA	isoform a	-4886	48.86	0.00
LDEC014556-RA	beta tubulin partial	-5019	50.19	0.00
LDEC002987-RA	amine oxidase	-504.39	124.96	0.25
LDEC007571-RA	PREDICTED: uncharacterized protein LOC102671488	-508.91	288.17	0.57
LDEC017806-RA	hypothetical protein DAPPUDRAFT_102793	-521.22	184.46	0.35
LDEC010450-RA	---NA---	-5357	53.57	0.00
LDEC022432-RA	coiled-coil domain-containing protein 37-like	-541.23	57.46	0.11

LDEC019014-RA	ubiquitin carboxyl-terminal hydrolase 5 isoform x1	-542.57	76.81	0.14
LDEC013407-RA	inorganic phosphate cotransporter-like	-5442	54.42	0.00
LDEC012877-RA	tetratricopeptide repeat protein 25-like	-5456	54.56	0.00
LDEC012507-RA	low quality protein: homeotic protein spalt-major	-549.96	38.93	0.07
LDEC000225-RA	hypothetical protein X975_15918, partial	-555.04	137.50	0.25
LDEC015247-RA	protein phosphatase 1 regulatory subunit 42-like	-555.11	58.94	0.11
LDEC021187-RA	mitochondrial import inner membrane translocase subunit tim16	-5589	55.89	0.00
LDEC004095-RA	radial spoke head 1 homolog	-561.38	39.73	0.07
LDEC000937-RA	protein anoxia up-regulated-like	-5708	57.08	0.00
LDEC009503-RA	histone gonadal-like	-577.83	81.80	0.14
LDEC017704-RA	cuticle protein	-5822	58.22	0.00
LDEC015388-RA	aldo-keto reductase	-5846	58.46	0.00
LDEC014702-RA	tbc1 domain family member 9	-590.75	167.26	0.28
LDEC011024-RA	adenylate cyclase type 10-like	-594.28	42.06	0.07
LDEC009475-RA	PREDICTED: uncharacterized protein LOC103314315	-6022	60.22	0.00
LDEC000109-RA	isoform a	-6079	60.79	0.00
LDEC003478-RA	protein d2-like isoform x2	-6098	60.98	0.00
LDEC002064-RA	---NA---	-6108	61.08	0.00
LDEC007637-RA	protein kinase c-binding protein 1	-6203	62.03	0.00
LDEC022529-RA	---NA---	-622.91	969.99	1.56
LDEC020879-RA	kif1-binding protein homolog	-6255	62.55	0.00
LDEC010075-RA	PREDICTED: uncharacterized protein LOC100141550	-626.51	44.35	0.07
LDEC000524-RA	photoreceptor outer segment membrane glycoprotein 2 isoform x1	-630.76	66.97	0.11
LDEC011876-RA	kelch repeat and btb domain-containing protein 12	-631.53	379.95	0.60
LDEC005196-RA	hypothetical protein D910_12529	-6611	66.11	0.00
LDEC009814-RA	testicular haploid expressed gene	-6630	66.30	0.00
LDEC010320-RA	cyclic nucleotide-binding domain-containing protein 2-like	-6692	66.92	0.00
LDEC021408-RA	major centromere autoantigen	-6702	67.02	0.00
LDEC005974-RA	tripeptidyl-peptidase 2-like	-681.57	48.24	0.07
LDEC009224-RA	hypothetical protein D910_09825	-685.60	48.53	0.07
LDEC002160-RA	retrovirus-related pol polyprotein from transposon tnt 1- partial	-688.42	121.82	0.18
LDEC012689-RA	titin isoform x2	-694.90	762.38	1.10
LDEC010305-RA	hypothetical protein TcasGA2_TC007828	-6992	69.92	0.00
LDEC007570-RA	melanoma-associated antigen e1	-704.63	374.06	0.53
LDEC014954-RA	sun domain-containing protein 2-like	-711.12	50.33	0.07
LDEC017922-RA	hypothetical protein YQE_12741, partial	-722.54	76.71	0.11
LDEC011846-RA	hypothetical protein YQE_03498, partial	-7282	72.82	0.00
LDEC023948-RA	hypothetical protein	-7424	74.24	0.00

	TcasGA2_TC004492			
LDEC009640-RA	microtubule-associated protein jupiter-like	-748.50	79.47	0.11
LDEC023222-RA	soluble trehalase	-7643	76.43	0.00
LDEC022717-RA	beta- partial	-7776	77.76	0.00
LDEC018546-RA	malate mitochondrial	-785.66	55.61	0.07
LDEC008553-RA	guanine nucleotide-binding protein subunit beta-like protein isoform x1	-787.67	55.75	0.07
LDEC008950-RA	spermine oxidase-like	-788.34	27.90	0.04
LDEC012525-RA	growth arrest-specific protein 8	-7956	79.56	0.00
LDEC023278-RA	myosin ia heavy chain-like	-798.42	56.51	0.07
LDEC005446-RA	peptidylprolyl isomerase domain and wd repeat-containing protein 1	-802.02	312.22	0.39
LDEC008264-RA	PREDICTED: uncharacterized protein LOC103315166	-803.12	28.42	0.04
LDEC007125-RA	testis-specific serine threonine-protein kinase 1-like	-8132	81.32	0.00
LDEC007024-RA	homer protein homolog 2-like	-815.20	57.70	0.07
LDEC017532-RA	proteasome activator complex subunit 3 isoform x2	-8204	82.04	0.00
LDEC005581-RA	stretch regulated skeletal muscle	-829.98	29.37	0.04
LDEC016462-RA	ferm domain-containing protein 5-like	-8327	83.27	0.00
LDEC005193-RA	hypothetical protein YQE_11734, partial	-859.07	182.42	0.21
LDEC006993-RA	hypothetical protein D910_02044, partial	-8627	86.27	0.00
LDEC007798-RA	ef-hand domain containing protein	-8660	86.60	0.00
LDEC012242-RA	PREDICTED: uncharacterized protein LOC105183325 isoform X1	-8660	86.60	0.00
LDEC015860-RA	facilitated trehalose transporter tret1-2 homolog	-8855	88.55	0.00
LDEC006335-RA	glutamine-rich protein 2-like	-899.14	63.64	0.07
LDEC004049-RA	potassium-channel inhibitor kcug1a precursor	-900.48	63.74	0.07
LDEC021548-RA	metaxin-2-like isoform x1	-900.48	63.74	0.07
LDEC017556-RA	aspartate mitochondrial	-9031	90.31	0.00
LDEC005197-RA	hypothetical protein D910_12529	-929.36	296.02	0.32
LDEC012807-RA	---NA---	-939.51	299.25	0.32
LDEC008506-RA	rna polymerase partial	-949.50	33.60	0.04
LDEC009903-RA	inositol-3-phosphate synthase 1-a	-9591	95.91	0.00
LDEC006987-RA	hypothetical protein TcasGA2_TC002146	-963.38	204.57	0.21
LDEC005791-RA	low quality protein: serine protease easter	-984.42	69.68	0.07
LDEC021461-RA	hypothetical protein YQE_00864, partial	-990.80	140.26	0.14
LDEC011703-RA	hypothetical protein YQE_01902, partial	-991.14	105.23	0.11
LDEC008933-RA	arginine kinase	-10038	100.38	0.00
LDEC010736-RA	dopamine n-acetyltransferase	-10130	101.10	0.00
LDEC017826-RA	nuclease harbi1	-10110	101.10	0.00
LDEC013029-RA	tubulin alpha-1 chain	-1012.62	107.51	0.11
LDEC024399-RA	mitochondrial carrier protein	-1016.65	71.96	0.07
LDEC000840-RA	lipopolysaccharide-induced tumor necrosis factor-alpha factor	-1036.13	73.34	0.07

	homolog			
LDEC021891-RA	a-kinase anchor protein 14-like	-1059.63	75.00	0.07
LDEC012831-RA	---NA---	-1067.69	75.57	0.07
LDEC008948-RA	peroxisomal n -acetyl-spermine spermidine oxidase	-1082.46	38.31	0.04
LDEC003554-RA	coiled-coil domain-containing protein 40	-1097.23	194.16	0.18
LDEC008406-RA	coiled-coil domain-containing protein 37	-11070	110.70	0.00
LDEC020325-RA	hypothetical protein YQE_12498, partial	-11093	110.93	0.00
LDEC000078-RA	wd repeat and fyve domain-containing protein 3-like	-1129.47	39.97	0.04
LDEC018150-RA	lactosylceramide 4-alpha-galactosyltransferase	-1131.48	80.09	0.07
LDEC014458-RA	dual specificity protein phosphatase cdc14a-like	-11678	116.78	0.00
LDEC004050-RA	hypothetical protein D910_01930	-1174.68	124.72	0.11
LDEC009839-RA	protein fam154a-like	-12139	121.39	0.00
LDEC018151-RA	hypothetical protein YQE_12145, partial	-12172	121.72	0.00
LDEC021295-RA	isoform b	-12481	124.81	0.00
LDEC000816-RA	peptidyl-prolyl cis-trans isomerase-like	-1261.08	44.63	0.04
LDEC017423-RA	leucine-rich repeat-containing protein 27 isoform x1	-1265.11	89.55	0.07
LDEC021027-RA	probable cytochrome p450 mitochondrial-like	-1322.86	187.27	0.14
LDEC009494-RA	c-type lectin 6	-1356.43	48.01	0.04
LDEC004268-RA	hypothetical protein D910_07447	-1381.95	48.91	0.04
LDEC006644-RA	pyruvate kinase	-1381.95	48.91	0.04
LDEC004096-RA	PREDICTED: uncharacterized protein LOC103312818 isoform X2	-1417.32	150.48	0.11
LDEC020315-RA	---NA---	-14701	147.01	0.00
LDEC021531-RA	hypothetical protein YQE_08544, partial	-14844	148.44	0.00
LDEC006336-RA	glutamine-rich protein 2-like	-1524.98	107.94	0.07
LDEC021543-RA	centrosomal protein of 78 kda	-15319	153.19	0.00
LDEC011905-RA	15-hydroxyprostaglandin dehydrogenase	-15409	154.09	0.00
LDEC006527-RA	leucine rich repeat containing 34	-1579.37	55.89	0.04
LDEC012118-RA	camp and camp-inhibited cgmp 3 -cyclic phosphodiesterase 10a-like	-1603.55	56.75	0.04
LDEC009298-RA	multidrug resistance	-16298	162.98	0.00
LDEC005167-RA	hypothetical protein YQE_01150, partial	-16502	165.02	0.00
LDEC020968-RA	hexokinase type 2-like isoform x6	-16902	169.02	0.00
LDEC010345-RA	carbonic anhydrase 13-like	-17096	170.96	0.00
LDEC019015-RA	PREDICTED: uncharacterized protein LOC103314218	-17453	174.53	0.00
LDEC022351-RA	voltage-gated cation ion channel dsc1	-17538	175.38	0.00
LDEC019701-RA	cytosol aminopeptidase	-1769.34	1815.92	1.03
LDEC000585-RA	enolase isoform x2	-1797.61	127.24	0.07
LDEC019180-RA	hypothetical protein D910_10293	-1834.54	64.93	0.04
LDEC008393-RA	g-protein coupled receptor mth2	-1858.72	131.56	0.07
LDEC018877-RA	major antigen-like	-1862.07	131.80	0.07

LDEC019206-RA	cap-gly domain-containing linker protein 1-like	-1921.17	135.98	0.07
LDEC018335-RA	centrosomal protein of 120 kda-like	-1921.84	68.02	0.04
LDEC003221-RA	keratin-associated protein 9-1-like	-19568	195.68	0.00
LDEC017613-RA	hypothetical protein YQE_01790, partial	-2052.28	581.05	0.28
LDEC018582-RA	serine protease mitochondrial	-2246.84	79.52	0.04
LDEC004642-RA	scp-like protein	-22928	229.28	0.00
LDEC000721-RA	rho gtpase-activating protein 11a-like	-2406.66	85.17	0.04
LDEC016616-RA	testis-specific serine threonine-protein kinase 1-like	-24459	244.59	0.00
LDEC021407-RA	hypothetical protein YQE_05759, partial	-24649	246.49	0.00
LDEC009613-RA	hypothetical protein D910_11253	-2541.63	179.90	0.07
LDEC014621-RA	---NA---	-2658.47	376.34	0.14
LDEC017712-RA	---NA---	-27254	272.54	0.00
LDEC017620-RA	dynein assembly factor axonemal	-2753.83	194.92	0.07
LDEC005026-RA	tubulin-specific chaperone	-2809.56	99.43	0.04
LDEC019013-RA	trinucleotide repeat-containing gene 18 protein	-2810.91	99.48	0.04
LDEC006979-RA	transcriptional enhancer factor tef-1 isoform x2	-28304	283.04	0.00
LDEC006978-RA	PREDICTED: titin	-2835.30	602.06	0.21
LDEC004376-RA	hypothetical protein D910_08597	-29245	292.45	0.00
LDEC016102-RA	2-oxoglutarate mitochondrial-like isoform x3	-2953.94	209.08	0.07
LDEC006474-RA	xk-related protein 4	-2958.64	104.71	0.04
LDEC003398-RA	PREDICTED: uncharacterized protein LOC103315104	-32938	329.38	0.00
LDEC018011-RA	---NA---	-33475	334.75	0.00
LDEC005890-RA	---NA---	-3674.46	130.04	0.04
LDEC011363-RA	c-amp-dependent rap1 guanine-nucleotide exchange factor	-39212	392.12	0.00
LDEC002949-RA	casein kinase i isoform gamma-3 isoform x2	-4128.39	146.11	0.04
LDEC018347-RA	hypothetical protein YQE_04263, partial	-4135.11	146.34	0.04
LDEC011536-RA	cytosol aminopeptidase	-50410	504.10	0.00
LDEC010304-RA	high mobility group b protein 4-like isoform x2	-7295.19	258.18	0.04
LDEC007111-RA	restin homolog isoform x2	-9634.71	681.96	0.07
LDEC006910-RA	neurofilament heavy polypeptide-like	-14825.75	2098.77	0.14
LDEC021703-RA	protein fam133a-like isoform x2	-156748	1567.48	0.00

Table 7S. Significantly differentially expressed genes, after Bonferroni correction, in an adult male and larval *Leptinotarsa decemlineata*.

Feature ID	Gene name	Experiment - Fold Change (normalized values)	Adult male - Normalized expression values	Larvae (GE) - Normalized expression values
LDEC003837-RA	endonuclease and reverse transcriptase-like protein	-180597.98	13842.89	0.08
LDEC024230-RA	cuticular protein 13	-41210.87	1579.41	0.04
LDEC013181-RA	chemosensory protein 8	-35193.56	674.40	0.02
LDEC021419-RA	---NA---	-338663	3386.63	0.00
LDEC013548-RA	glycine-rich protein	-31676.19	6069.97	0.19
LDEC019701-RA	cytosol aminopeptidase	-31587.97	1815.92	0.06
LDEC006905-RA	endocuticle structural glycoprotein bd-	-29368.48	1125.55	0.04
LDEC022529-RA	---NA---	-25309.40	969.99	0.04
LDEC006910-RA	neurofilament heavy polypeptide-like	-209877	2098.77	0.00
LDEC003957-RA	hypothetical protein D910_10187	-20650.70	1582.88	0.08
LDEC012689-RA	titin isoform x2	-19892.34	762.38	0.04
LDEC007111-RA	restin homolog isoform x2	-17793.97	681.96	0.04
LDEC006978-RA	PREDICTED: titin	-15709.24	602.06	0.04
LDEC021703-RA	protein fam133a-like isoform x2	-156748	1567.48	0.00
LDEC017613-RA	hypothetical protein YQE_01790, partial	-15161.09	581.05	0.04
LDEC006899-RA	cuticular protein isoform a	-110806	1108.06	0.00
LDEC001846-RA	hypothetical protein YQE_08339, partial	-10899.86	417.74	0.04
LDEC017905-RA	protoheme ix mitochondrial-like	-103591	1035.91	0.00
LDEC015532-RA	glycine-rich protein	-10243.40	1177.74	0.11
LDEC003221-RA	keratin-associated protein 9-1-like	-10211.56	195.68	0.02
LDEC002623-RA	major antigen-like	-9446.79	543.07	0.06
LDEC019015-RA	PREDICTED: uncharacterized protein LOC103314218	-9107.81	174.53	0.02
LDEC001840-RA	alpha-tocopherol transfer	-6880.47	131.85	0.02
LDEC006898-RA	endocuticle structural glycoprotein bd-1	-67929	679.29	0.00
LDEC003437-RA	hypothetical protein YQE_12777, partial	-6657.46	5613.26	0.84
LDEC021407-RA	hypothetical protein YQE_05759, partial	-6431.52	246.49	0.04
LDEC018396-RA	dynein heavy chain	-5688.66	218.02	0.04
LDEC010793-RA	mature sequence toxin-like lfec	-5499.29	3477.56	0.63
LDEC011536-RA	cytosol aminopeptidase	-5261.30	504.10	0.10
LDEC006427-RA	odorant binding protein 1	-49887	498.87	0.00

LDEC017423-RA	leucine-rich repeat-containing protein 27 isoform x1	-4672.96	89.55	0.02
LDEC017904-RA	---NA---	-4458.00	512.56	0.11
LDEC005124-RA	tetratricopeptide repeat protein 19	-4439.81	85.08	0.02
LDEC003962-RA	---NA---	-4400.13	1686.35	0.38
LDEC018150-RA	lactosylceramide 4-alpha-galactosyltransferase	-4179.37	80.09	0.02
LDEC006736-RA	protein efr3-like protein a	-4142.17	79.37	0.02
LDEC003956-RA	cuticle protein 63	-3987.15	152.81	0.04
LDEC011363-RA	c-amp-dependent rap1 guanine-nucleotide exchange factor	-39212	392.12	0.00
LDEC012807-RA	---NA---	-3904.06	299.25	0.08
LDEC011876-RA	kelch repeat and btb domain-containing protein 12	-37995	379.95	0.00
LDEC014621-RA	---NA---	-37634	376.34	0.00
LDEC015048-RA	26s proteasome non-atpase regulatory subunit 2	-3742.83	71.72	0.02
LDEC007570-RA	melanoma-associated antigen e1	-37406	374.06	0.00
LDEC021461-RA	hypothetical protein YQE_00864, partial	-3659.74	140.26	0.04
LDEC006138-RA	PREDICTED: uncharacterized protein LOC103315014 isoform X1	-3447.67	132.13	0.04
LDEC005466-RA	protein mesh isoform x3	-3430.31	65.73	0.02
LDEC002650-RA	---NA---	-3424.73	262.51	0.08
LDEC019180-RA	hypothetical protein D910_10293	-3388.15	64.93	0.02
LDEC018011-RA	---NA---	-33475	334.75	0.00
LDEC004049-RA	potassium-channel inhibitor kcug1a precursor	-3326.14	63.74	0.02
LDEC000585-RA	enolase isoform x2	-3319.94	127.24	0.04
LDEC003398-RA	PREDICTED: uncharacterized protein LOC103315104	-32938	329.38	0.00
LDEC005070-RA	glycine-rich protein	-3202.68	2454.86	0.77
LDEC003838-RA	---NA---	-3020.02	694.46	0.23
LDEC019428-RA	PREDICTED: uncharacterized protein LOC103314495	-2941.68	112.74	0.04
LDEC004376-RA	hypothetical protein D910_08597	-29245	292.45	0.00
LDEC007571-RA	PREDICTED: uncharacterized protein LOC102671488	-28817	288.17	0.00
LDEC006979-RA	transcriptional enhancer factor tef-1 isoform x2	-28304	283.04	0.00
LDEC013029-RA	tubulin alpha-1 chain	-2805.27	107.51	0.04
LDEC011703-RA	hypothetical protein YQE_01902, partial	-2745.74	105.23	0.04
LDEC006474-RA	xk-related protein 4	-2732.10	104.71	0.04
LDEC017712-RA	---NA---	-27254	272.54	0.00
LDEC021105-RA	tubulin polyglutamylase ttll6	-2658.93	152.86	0.06
LDEC010304-RA	high mobility group b protein 4-like isoform x2	-25818	258.18	0.00
LDEC009494-RA	c-type lectin 6	-2505.14	48.01	0.02
LDEC016616-RA	testis-specific serine threonine-protein kinase 1-like	-24459	244.59	0.00
LDEC002713-RA	f-box only protein 39-like	-2370.79	272.58	0.11
LDEC017556-RA	aspartate mitochondrial	-2356.32	90.31	0.04
LDEC005446-RA	peptidylprolyl isomerase domain and wd repeat-containing protein 1	-2327.62	312.22	0.13

LDEC002028-RA	outer dense fiber protein 2-like	-23023	230.23	0.00
LDEC004642-RA	scp-like protein	-22928	229.28	0.00
LDEC006993-RA	hypothetical protein D910_02044, partial	-2250.91	86.27	0.04
LDEC002160-RA	retrovirus-related pol polyprotein from transposon tnt 1- partial	-2119.04	121.82	0.06
LDEC016102-RA	2-oxoglutarate mitochondrial-like isoform x3	-20908	209.08	0.00
LDEC007936-RA	myotubularin	-2077.29	159.22	0.08
LDEC012525-RA	growth arrest-specific protein 8	-2076.05	79.56	0.04
LDEC018582-RA	serine protease mitochondrial	-2074.81	79.52	0.04
LDEC006987-RA	hypothetical protein TcasGA2_TC002146	-20408	204.57	0.00
LDEC019014-RA	ubiquitin carboxyl-terminal hydrolase 5 isoform x1	-2004.12	76.81	0.04
LDEC005689-RA	cathepsin partial	-1993.37	114.59	0.06
LDEC000919-RA	glycosyl hydrolase	-1961.12	112.74	0.06
LDEC005603-RA	cytochrome p450 partial	-1909.86	73.20	0.04
LDEC009175-RA	coiled-coil domain-containing protein 113-like	-1897.46	36.36	0.02
LDEC015974-RA	cuticle protein 1	-1889.81	4707.77	2.49
LDEC024399-RA	mitochondrial carrier protein	-1877.62	71.96	0.04
LDEC021027-RA	probable cytochrome p450 mitochondrial-like	-18727	187.27	0.00
LDEC022024-RA	mucin related isoform a	-1867.08	143.11	0.08
LDEC012916-RA	cytochrome p450 partial	-1830.49	35.08	0.02
LDEC001986-RA	upf0704 protein c6orf165 homolog	-1826.77	70.01	0.04
LDEC005791-RA	low quality protein: serine protease easter	-1818.09	69.68	0.04
LDEC009613-RA	hypothetical protein D910_11253	-17990	179.90	0.00
LDEC022351-RA	voltage-gated cation ion channel dsc1	-17538	175.38	0.00
LDEC000524-RA	photoreceptor outer segment membrane glycoprotein 2 isoform x1	-1747.40	66.97	0.04
LDEC010107-RA	agap009741-pa-like protein	-1701.51	32.61	0.02
LDEC005890-RA	---NA---	-1696.55	130.04	0.08
LDEC020968-RA	hexokinase type 2-like isoform x6	-16902	169.02	0.00
LDEC013867-RA	ring finger protein 145	-16783	167.83	0.00
LDEC014702-RA	tbc1 domain family member 9	-16726	167.26	0.00
LDEC005602-RA	cytochrome p450	-1662.45	127.43	0.08
LDEC005167-RA	hypothetical protein YQE_01150, partial	-16502	165.02	0.00
LDEC020879-RA	kif1-binding protein homolog	-1632.06	62.55	0.04
LDEC004064-RA	malate dehydrogenase	-1630.82	62.50	0.04
LDEC009298-RA	multidrug resistance	-16298	162.98	0.00
LDEC016767-RA	testis-specific serine threonine-protein kinase 4-like	-1612.22	61.79	0.04
LDEC000109-RA	isoform a	-1586.18	60.79	0.04
LDEC010003-RA	hypothetical protein KGM_06951	-1542.77	29.56	0.02
LDEC011905-RA	15-hydroxyprostaglandin dehydrogenase	-15409	154.09	0.00
LDEC006897-RA	endocuticle structural glycoprotein bd-8-like	-1532.85	88.12	0.06
LDEC021543-RA	centrosomal protein of 78 kda	-15319	153.19	0.00

LDEC004096-RA	PREDICTED: uncharacterized protein LOC103312818 isoform X2	-15048	150.48	0.00
LDEC000937-RA	protein anoxia up-regulated-like	-1489.44	57.08	0.04
LDEC021531-RA	hypothetical protein YQE_08544, partial	-14844	148.44	0.00
LDEC012331-RA	ubiquitin-protein ligase	-1483.24	28.42	0.02
LDEC020315-RA	---NA---	-14701	147.01	0.00
LDEC018347-RA	hypothetical protein YQE_04263, partial	-14634	146.34	0.00
LDEC002949-RA	casein kinase i isoform gamma-3 isoform x2	-14611	146.11	0.00
LDEC012785-RA	chymotrypsin-like proteinase 6d precursor	-1449.76	55.56	0.04
LDEC001696-RA	phosphoglycerate mutase 1	-1449.14	111.08	0.08
LDEC016769-RA	cytochrome p450 412a1	-14287	142.87	0.00
LDEC020604-RA	hypothetical protein D910_02954	-13869	138.69	0.00
LDEC009640-RA	microtubule-associated protein jupiter-like	-1382.38	79.47	0.06
LDEC001963-RA	leucine-rich repeat-containing protein 51-like	-1380.31	105.80	0.08
LDEC017171-RA	galactose-specific c-type lectin	-1377.28	475.06	0.34
LDEC004191-RA	27 kda hemolymph protein	-1371.63	26.28	0.02
LDEC015372-RA	synaptic vesicle protein 2	-1366.67	26.19	0.02
LDEC019206-RA	cap-gly domain-containing linker protein 1-like	-13598	135.98	0.00
LDEC007675-RA	---NA---	-1343.10	257.37	0.19
LDEC020978-RA	troponin isoform 1-like	-1330.70	102.00	0.08
LDEC021664-RA	peroxidase-like isoform 1	-1315.82	50.43	0.04
LDEC008393-RA	g-protein coupled receptor mth2	-13156	131.56	0.00
LDEC002262-RA	udp-glucuronosyltransferase 2b10-like	-13090	130.90	0.00
LDEC016023-RA	zinc finger and btb domain-containing protein 24 isoform x2	-1302.18	49.91	0.04
LDEC017620-RA	dynein assembly factor axonemal	-1271.48	194.92	0.15
LDEC005974-RA	tripeptidyl-peptidase 2-like	-1258.77	48.24	0.04
LDEC000920-RA	glycosyl hydrolase	-12519	125.19	0.00
LDEC021295-RA	isoform b	-12481	124.81	0.00
LDEC006908-RA	nad-dependent protein deacetylase sirt2	-12467	124.67	0.00
LDEC015389-RA	leucine-rich repeat-containing protein 48	-1222.81	46.86	0.04
LDEC018151-RA	hypothetical protein YQE_12145, partial	-12172	121.72	0.00
LDEC009839-RA	protein fam154a-like	-12199	121.39	0.00
LDEC000528-RA	PREDICTED: uncharacterized protein LOC100881963	-1210.41	46.39	0.04
LDEC008942-RA	PREDICTED: uncharacterized protein LOC661884	-12082	120.82	0.00
LDEC000225-RA	hypothetical protein X975_15918, partial	-1195.94	137.50	0.11
LDEC005193-RA	hypothetical protein YQE_11734, partial	-1189.94	182.42	0.15
LDEC008267-RA	sperm-associated antigen 6-like	-1174.44	45.01	0.04
LDEC024265-RA	hypothetical protein D910_02300	-1173.20	44.96	0.04
LDEC015155-RA	unknown	-1157.70	88.74	0.08
LDEC013105-RA	interferon regulatory factor 2-binding isoform x1	-11464	114.64	0.00

LDEC020429-RA	prostatic acid phosphatase	-1124.42	129.28	0.11
LDEC010345-RA	carbonic anhydrase 13-like	-1115.22	170.96	0.15
LDEC000721-RA	rho gtpase-activating protein 11a-like	-1111.19	85.17	0.08
LDEC020325-RA	hypothetical protein YQE_12498, partial	-11093	110.93	0.00
LDEC008406-RA	coiled-coil domain-containing protein 37	-11070	110.70	0.00
LDEC004050-RA	hypothetical protein D910_01930	-1084.74	124.72	0.11
LDEC017041-RA	tubulin alpha chain	-1081.43	41.45	0.04
LDEC006336-RA	glutamine-rich protein 2-like	-10794	107.94	0.00
LDEC007637-RA	protein kinase c-binding protein 1	-1078.95	62.03	0.06
LDEC019661-RA	ef-hand calcium-binding domain-containing protein 1	-1075.23	41.21	0.04
LDEC015764-RA	upf0704 protein c6orf165 homolog	-1052.90	80.71	0.08
LDEC012861-RA	alcohol dehydrogenase	-1046.21	100.24	0.10
LDEC003423-RA	cuticular protein ld-cp2	-1044.66	680.63	0.65
LDEC012507-RA	low quality protein: homeotic protein spalt-major	-1015.70	38.93	0.04
LDEC010736-RA	dopamine n-acetyltransferase	-10110	101.10	0.00
LDEC017826-RA	nuclease harbi1	-10110	101.10	0.00
LDEC021939-RA	aldose 1-epimerase	-994.62	95.30	0.10
LDEC005026-RA	tubulin-specific chaperone	-9943	99.43	0.00
LDEC018877-RA	major antigen-like	-982.57	131.80	0.13
LDEC008893-RA	odorant-binding protein 4	-9805	98.05	0.00
LDEC009903-RA	inositol-3-phosphate synthase 1-a	-9591	95.91	0.00
LDEC000139-RA	heat shock cognate 71 kda isoform x1	-9563	95.63	0.00
LDEC015154-RA	PREDICTED: uncharacterized protein LOC103312842	-9492	94.92	0.00
LDEC003363-RA	hypothetical protein YQE_07867, partial	-946.25	72.53	0.08
LDEC005166-RA	fact complex subunit ssrp1-like	-938.63	125.91	0.13
LDEC008890-RA	odorant-binding protein 4	-9368	93.68	0.00
LDEC001008-RA	succinate dehydrogenase	-930.13	53.47	0.06
LDEC008422-RA	dentin sialophosphoprotein	-925.17	35.46	0.04
LDEC016445-RA	hypothetical protein TcasGA2_TC006984	-923.04	123.81	0.13
LDEC014993-RA	mitochondrial import inner membrane translocase subunit tim50-c	-908.11	139.21	0.15
LDEC005192-RA	hypothetical protein D910_12530	-901.60	34.55	0.04
LDEC015400-RA	hypothetical protein D910_05843	-8874	88.74	0.00
LDEC015860-RA	facilitated trehalose transporter tret1-2 homolog	-8855	88.55	0.00
LDEC017830-RA	---NA---	-8841	88.41	0.00
LDEC003954-RA	chromatin assembly factor 1 subunit fas2	-8669	86.69	0.00
LDEC021138-RA	endonuclease-reverse transcriptase	-866.47	99.62	0.11
LDEC007798-RA	ef-hand domain containing protein	-8660	86.60	0.00
LDEC012242-RA	PREDICTED: uncharacterized protein LOC105183325 isoform X1	-8660	86.60	0.00
LDEC020977-RA	troponin c type iiia-like protein	-865.64	33.18	0.04
LDEC005196-RA	hypothetical protein D910_12529	-862.54	66.11	0.08

LDEC002162-RA	small subunit processome component 20-like protein	-8622	86.22	0.00
LDEC005197-RA	hypothetical protein D910_12529	-858.20	296.02	0.34
LDEC003401-RA	isoform b	-856.96	131.37	0.15
LDEC003960-RA	reverse transcriptase	-847.40	1477.70	1.74
LDEC006907-RA	hth-type transcriptional repressor -like	-8446	84.46	0.00
LDEC003554-RA	coiled-coil domain-containing protein 40	-844.35	194.16	0.23
LDEC000621-RA	venom acid phosphatase acph-1-like	-839.60	32.18	0.04
LDEC016462-RA	ferm domain-containing protein 5-like	-8327	83.27	0.00
LDEC017532-RA	proteasome activator complex subunit 3 isoform x2	-8204	82.04	0.00
LDEC001591-RA	minus-c odorant binding protein 3	-818.72	188.27	0.23
LDEC007125-RA	testis-specific serine threonine-protein kinase 1-like	-8132	81.32	0.00
LDEC024104-RA	PREDICTED: uncharacterized protein LOC100142328	-8123	81.23	0.00
LDEC023150-RA	af377988_1trypsin-like serine protease	-801.15	46.06	0.06
LDEC010337-RA	circumsporozoite protein	-7956	79.56	0.00
LDEC010199-RA	tubulin glycylase 3a-like	-793.30	91.21	0.11
LDEC015923-RA	lipase 3-like	-780.07	29.90	0.04
LDEC022717-RA	beta- partial	-7776	77.76	0.00
LDEC002486-RA	15-hydroxyprostaglandin dehydrogenase	-773.04	44.44	0.06
LDEC017922-RA	hypothetical protein YQE_12741, partial	-7671	76.71	0.00
LDEC002546-RA	hypothetical protein YQE_05003, partial	-765.19	87.98	0.11
LDEC023222-RA	soluble trehalase	-7643	76.43	0.00
LDEC007890-RA	PREDICTED: uncharacterized protein C1orf177 homolog	-763.95	43.92	0.06
LDEC012831-RA	---NA---	-7557	75.57	0.00
LDEC021891-RA	a-kinase anchor protein 14-like	-7500	75.00	0.00
LDEC002210-RA	ciliary dynein heavy	-747.00	85.89	0.11
LDEC004267-RA	hypothetical protein D910_07448	-7443	74.43	0.00
LDEC023948-RA	hypothetical protein TcasGA2_TC004492	-7424	74.24	0.00
LDEC006130-RA	hypothetical protein D910_09009	-738.52	56.61	0.08
LDEC004149-RA	polycystic kidney disease protein 1-like 2	-736.66	56.47	0.08
LDEC000840-RA	lipopolysaccharide-induced tumor necrosis factor-alpha factor homolog	-7334	73.34	0.00
LDEC008894-RA	protein expanded	-7310	73.10	0.00
LDEC011846-RA	hypothetical protein YQE_03498, partial	-7282	72.82	0.00
LDEC008553-RA	guanine nucleotide-binding protein subunit beta-like protein isoform x1	-727.36	55.75	0.08
LDEC019772-RA	facilitated trehalose transporter tret1	-725.50	27.80	0.04
LDEC009292-RA	PREDICTED: uncharacterized protein LOC103314814	-715.58	27.42	0.04
LDEC004733-RA	glucose dehydrogenase	-713.72	109.41	0.15
LDEC018389-RA	dynein beta ciliary-like isoform x2	-712.11	136.46	0.19
LDEC009503-RA	histone gonadal-like	-711.44	81.80	0.11
LDEC019558-RA	fatty acyl- reductase cg5065	-710.37	68.06	0.10
LDEC005068-RA	glycine-rich protein	-700.37	1597.09	2.28

LDEC010305-RA	hypothetical protein TcasGA2_TC007828	-6992	69.92	0.00
LDEC010198-RA	tubulin tyrosine ligase-like 3a	-6954	69.54	0.00
LDEC014713-RA	wd repeat domain 61	-6925	69.25	0.00
LDEC018335-RA	centrosomal protein of 120 kda-like	-6802	68.02	0.00
LDEC007624-RA	GJ17783	-672.02	206.04	0.31
LDEC021408-RA	major centromere autoantigen	-6702	67.02	0.00
LDEC010320-RA	cyclic nucleotide-binding domain-containing protein 2-like	-6692	66.92	0.00
LDEC009814-RA	testicular haploid expressed gene	-6630	66.30	0.00
LDEC016106-RA	tripartite motif-containing protein 71-like	-661.01	50.67	0.08
LDEC014943-RA	l-2-hydroxyglutarate mitochondrial-like	-6545	65.45	0.00
LDEC017040-RA	tubulin alpha chain-like	-650.98	137.22	0.21
LDEC018784-RA	short transient receptor potential channel 5-like	-646.13	49.53	0.08
LDEC021548-RA	metaxin-2-like isoform x1	-6374	63.74	0.00
LDEC006335-RA	glutamine-rich protein 2-like	-6364	63.64	0.00
LDEC011362-RA	leucine-rich repeat-containing protein ddb_g0290503	-635.32	255.66	0.40
LDEC010357-RA	hypothetical protein YQE_00629, partial	-6345	63.45	0.00
LDEC010156-RA	methyltransferase nsun7	-633.73	48.58	0.08
LDEC018630-RA	ubiquitin-like modifier-activating enzyme atg7-like isoform x3	-6207	62.07	0.00
LDEC014883-RA	coiled-coil domain-containing protein 147-like	-612.33	93.87	0.15
LDEC017170-RA	isoform a	-611.40	46.86	0.08
LDEC002064-RA	---NA---	-6108	61.08	0.00
LDEC014665-RA	serine threonine-protein kinase paka-like	-610.58	70.20	0.11
LDEC003478-RA	protein d2-like isoform x2	-6098	60.98	0.00
LDEC019151-RA	hypothetical protein D910_11774	-608.92	46.67	0.08
LDEC009475-RA	PREDICTED: uncharacterized protein LOC103314315	-6022	60.22	0.00
LDEC000508-RA	cytosolic non-specific dipeptidase-like	-5993	59.93	0.00
LDEC013721-RA	ef-hand calcium-binding domain-containing protein 1	-5941	59.41	0.00
LDEC001730-RA	inducible metalloproteinase inhibitor	-593.63	34.13	0.06
LDEC015247-RA	protein phosphatase 1 regulatory subunit 42-like	-5894	58.94	0.00
LDEC002598-RA	PREDICTED: uncharacterized protein LOC103313140	-5875	58.75	0.00
LDEC015388-RA	aldo-keto reductase	-5846	58.46	0.00
LDEC017901-RA	cytochrome c oxidase subunit va	-584.12	44.77	0.08
LDEC001878-RA	lim-like muscle protein	-582.26	133.89	0.23
LDEC017704-RA	cuticle protein	-5822	58.22	0.00
LDEC001951-RA	glyceraldehyde-3-phosphate dehydrogenase	-580.90	55.66	0.10
LDEC007024-RA	homer protein homolog 2-like	-5770	57.70	0.00
LDEC017641-RA	malate mitochondrial	-576.50	231.99	0.40
LDEC005889-RA	hypothetical protein TcasGA2_TC005656	-5761	57.61	0.00
LDEC022432-RA	coiled-coil domain-containing protein 37-like	-5746	57.46	0.00

LDEC012118-RA	camp and camp-inhibited cgmp 3 -cyclic phosphodiesterase 10a-like	-5675	56.75	0.00
LDEC023278-RA	myosin ia heavy chain-like	-5651	56.51	0.00
LDEC019079-RA	hypothetical protein YQE_12002, partial	-5647	56.47	0.00
LDEC015275-RA	wd repeat-containing protein 66-like	-5594	55.94	0.00
LDEC006527-RA	leucine rich repeat containing 34	-5589	55.89	0.00
LDEC021187-RA	mitochondrial import inner membrane translocase subunit tim16	-5589	55.89	0.00
LDEC018546-RA	malate mitochondrial	-5561	55.61	0.00
LDEC010555-RA	soluble nsf attachment protein	-553.12	42.40	0.08
LDEC010155-RA	methyltransferase nsun7	-551.88	42.30	0.08
LDEC012877-RA	tetratricopeptide repeat protein 25-like	-5456	54.56	0.00
LDEC013407-RA	inorganic phosphate cotransporter-like	-5442	54.42	0.00
LDEC011981-RA	protein nedd1	-543.19	62.45	0.11
LDEC007831-RA	coiled-coil domain-containing protein 151-like	-537.24	51.47	0.10
LDEC010450-RA	---NA---	-5357	53.57	0.00
LDEC007702-RA	methyltransferase wbscr22	-5352	53.52	0.00
LDEC007308-RA	tektin-1	-533.27	40.88	0.08
LDEC021361-RA	proteasome-associated protein ecm29 homolog	-5323	53.23	0.00
LDEC015473-RA	probable multidrug resistance-associated protein lethal 03659	-532.03	40.78	0.08
LDEC004353-RA	wd repeat-containing protein 16	-5257	52.57	0.00
LDEC013649-RA	---NA---	-5238	52.38	0.00
LDEC010331-RA	leucine-rich repeat-containing protein 23-like	-5048	50.48	0.00
LDEC014954-RA	sun domain-containing protein 2-like	-5033	50.33	0.00
LDEC010055-RA	glucose dehydrogenase	-503.10	115.69	0.23
LDEC014556-RA	beta tubulin partial	-5019	50.19	0.00
LDEC001650-RA	atp synthase subunit mitochondrial-like	-494.41	28.42	0.06
LDEC004268-RA	hypothetical protein D910_07447	-4891	48.91	0.00
LDEC006644-RA	pyruvate kinase	-4891	48.91	0.00
LDEC009224-RA	hypothetical protein D910_09825	-4853	48.53	0.00
LDEC019463-RA	protein mcm10 homolog	-4796	47.96	0.00
LDEC008933-RA	arginine kinase	-476.23	100.38	0.21
LDEC001269-RA	39s ribosomal protein mitochondrial-like	-470.64	36.08	0.08
LDEC003242-RA	kinesin-like protein klp10a	-470.44	54.09	0.11
LDEC018385-RA	dynein heavy chain axonemal	-466.92	35.79	0.08
LDEC011420-RA	retinitis pigmentosa 1-like 1 protein	-464.44	35.60	0.08
LDEC009896-RA	PREDICTED: uncharacterized protein LOC103312770	-460.72	35.31	0.08
LDEC000497-RA	PREDICTED: uncharacterized protein LOC100164320	-460.03	1119.56	2.43
LDEC024113-RA	ring finger protein partial	-4520	45.20	0.00
LDEC009496-RA	---NA---	-4492	44.92	0.00
LDEC000816-RA	peptidyl-prolyl cis-trans isomerase-like	-4463	44.63	0.00
LDEC010075-RA	PREDICTED: uncharacterized protein LOC100141550	-4435	44.35	0.00

LDEC010102-RA	talin-1 isoform x1	-443.10	118.87	0.27
LDEC017555-RA	aspartate mitochondrial	-4430	44.30	0.00
LDEC017906-RA	lipase 3	-442.12	33.89	0.08
LDEC012441-RA	morn repeat-containing protein 5-like	-4416	44.16	0.00
LDEC011223-RA	na+ + atpase alpha-subunit partial	-436.13	50.14	0.11
LDEC003697-RA	zinc finger protein zxdc	-435.71	100.19	0.23
LDEC002651-RA	probable pseudouridine-5 - monophosphatase isoform x2	-435.05	41.68	0.10
LDEC008431-RA	thap domain-containing protein 4-like	-4335	43.35	0.00
LDEC005803-RA	tubulin beta-1 chain	-428.37	1305.17	3.05
LDEC009193-RA	hypothetical protein TcasGA2_TC003077	-4278	42.78	0.00
LDEC003214-RA	hypothetical protein D910_04081	-426.62	32.70	0.08
LDEC016663-RA	aaa atpase	-423.52	32.46	0.08
LDEC018180-RA	kelch-like protein 10	-422.28	97.10	0.23
LDEC011024-RA	adenylate cyclase type 10-like	-4206	42.06	0.00
LDEC016809-RA	radial spoke head protein 4 homolog a-like	-4202	42.02	0.00
LDEC001794-RA	nose resistant to fluoxetine protein 6	-418.82	56.18	0.13
LDEC020587-RA	isoform b	-415.46	31.84	0.08
LDEC001340-RA	vitellogenin	-410.50	78.66	0.19
LDEC015503-RA	cuticle protein precursor	-410.29	94.35	0.23
LDEC001261-RA	hypothetical protein D910_07352	-4102	41.02	0.00
LDEC011881-RA	serpin peptidase inhibitor 18	-409.88	62.83	0.15
LDEC008402-RA	kelch-like protein 10-like	-4092	40.92	0.00
LDEC009813-RA	testicular haploid expressed gene	-408.76	39.16	0.10
LDEC005601-RA	cytochrome p450	-406.16	31.13	0.08
LDEC002625-RA	major antigen-like	-404.47	108.51	0.27
LDEC006333-RA	tetratricopeptide repeat protein 39c-like	-401.82	100.10	0.25
LDEC022201-RA	agap007079-pa-like protein	-4012	40.12	0.00
LDEC000078-RA	wd repeat and fyve domain-containing protein 3-like	-3997	39.97	0.00
LDEC004095-RA	radial spoke head 1 homolog	-3973	39.73	0.00
LDEC020903-RA	multiple ankyrin repeats single kh domain protein	-3964	39.64	0.00
LDEC015049-RA	26s proteasome non-atpase regulatory subunit 2	-395.44	53.04	0.13
LDEC003286-RA	regulator of microtubule dynamics protein 1-like isoform x2	-3935	39.35	0.00
LDEC000182-RA	atp-dependent rna	-393.08	474.54	1.21
LDEC003915-RA	ubiquitin carboxyl-terminal	-392.34	6382.91	16.27
LDEC012220-RA	fk506-binding protein 5-like isoform x4	-388.96	81.99	0.21
LDEC016739-RA	dynein heavy chain	-385.69	29.56	0.08
LDEC005638-RA	glucose dehydrogenase	-384.84	752.21	1.95
LDEC008895-RA	odorant-binding protein 4	-381.35	58.46	0.15
LDEC008302-RA	sorbitol dehydrogenase	-3788	37.88	0.00
LDEC017760-RA	aael004257- partial	-377.84	43.44	0.11
LDEC003746-RA	4-coumarate-- ligase 1-like	-3774	37.74	0.00

LDEC004760-RA	alpha-tocopherol transfer	-377.22	86.74	0.23
LDEC008891-RA	odorant-binding protein 4	-3726	37.26	0.00
LDEC013117-RA	tctex1 domain-containing protein 1-like	-3707	37.07	0.00
LDEC005719-RA	multidrug resistance-associated protein 4	-368.02	56.42	0.15
LDEC013782-RA	succinate dehydrogenase	-3674	36.74	0.00
LDEC007110-RA	agap001754-pa-like protein	-3669	36.69	0.00
LDEC010917-RA	hypothetical protein D910_09343	-365.44	105.04	0.29
LDEC002564-RA	ribonuclease h2 subunit c-like	-3627	36.27	0.00
LDEC011874-RA	kelch repeat and btb domain-containing protein 12	-3622	36.22	0.00
LDEC018390-RA	dynein heavy chain	-3622	36.22	0.00
LDEC003016-RA	set and mynd domain-containing protein 4	-362.13	27.76	0.08
LDEC013547-RA	cuticle protein 21	-359.07	440.36	1.23
LDEC011878-RA	antichymotrypsin-2-like isoform x3	-357.76	171.39	0.48
LDEC014077-RA	isoform e	-3574	35.74	0.00
LDEC021635-RA	circadian clock-controlled protein	-355.93	40.92	0.11
LDEC014121-RA	cytochrome c-2	-3546	35.46	0.00
LDEC012069-RA	hypothetical protein D910_05118	-352.83	27.04	0.08
LDEC004567-RA	map microtubule affinity-regulating kinase 3-like	-352.62	40.54	0.11
LDEC018234-RA	golgin subfamily a member 4-like	-350.97	40.35	0.11
LDEC023634-RA	nadh dehydrogenase	-3479	34.79	0.00
LDEC019013-RA	trinucleotide repeat-containing gene 18 protein	-346.09	99.48	0.29
LDEC013055-RA	cytochrome b-c1 complex subunit 8	-3427	34.27	0.00
LDEC008627-RA	e3 ubiquitin-protein ligase ubr3	-339.81	32.56	0.10
LDEC008506-RA	rna polymerase partial	-3360	33.60	0.00
LDEC001248-RA	cytochrome c oxidase subunit mitochondrial	-3346	33.46	0.00
LDEC015839-RA	mitogen-activated protein kinase erk-	-3341	33.41	0.00
LDEC017806-RA	hypothetical protein DAPPUDRAFT_102793	-331.94	184.46	0.56
LDEC016080-RA	coiled-coil domain-containing protein 37	-3184	31.84	0.00
LDEC013180-RA	chemosensory protein 6	-316.11	611.80	1.94
LDEC013593-RA	hypothetical protein D910_04991	-315.42	36.27	0.11
LDEC016539-RA	vesicle-associated membrane protein-associated protein b	-3146	31.46	0.00
LDEC000115-RA	rib43a-like with coiled-coils protein 2	-312.94	35.98	0.11
LDEC008989-RA	protein disulfide-isomerase	-3089	30.89	0.00
LDEC006129-RA	hypothetical protein YQE_09365, partial	-3018	30.18	0.00
LDEC002063-RA	hypothetical protein TcasGA2_TC012351	-3013	30.13	0.00
LDEC011040-RA	PREDICTED: uncharacterized protein LOC103310485	-301.01	80.75	0.27
LDEC014107-RA	elongation of very long chain fatty acids protein aael008004	-297.35	96.87	0.33
LDEC005581-RA	stretch regulated skeletal muscle	-2937	29.37	0.00
LDEC008403-RA	kelch-like protein 10-like	-2937	29.37	0.00
LDEC014383-RA	abnormal spindle-like microcephaly-associated protein homolog	-2928	29.28	0.00

LDEC006896-RA	cuticular protein rr-1 motif 32	-291.60	89.40	0.31
LDEC011145-RA	acylphosphatase-2-like isoform x3	-287.41	132.18	0.46
LDEC008264-RA	PREDICTED: uncharacterized protein LOC103315166	-2842	28.42	0.00
LDEC003467-RA	e3 ubiquitin-protein ligase trim37-like isoform x2	-282.53	59.55	0.21
LDEC015289-RA	endothelin-converting enzyme 1-like	-2814	28.14	0.00
LDEC006293-RA	dynein heavy chain axonemal	-278.21	31.99	0.11
LDEC017197-RA	v-type proton atpase subunit f 1-like	-2757	27.57	0.00
LDEC022714-RA	hypothetical protein TcasGA2_TC001927	-275.52	63.36	0.23
LDEC003961-RA	PREDICTED: uncharacterized protein LOC655051	-273.39	94.30	0.34
LDEC000906-RA	piggybac transposable element-derived protein 3-like	-273.33	26.19	0.10
LDEC003635-RA	protein lap4	-2733	27.33	0.00
LDEC003453-RA	endocuticle structural glycoprotein bd-1-like	-272.24	130.42	0.48
LDEC016446-RA	iq domain-containing protein g-like	-271.85	26.05	0.10
LDEC013722-RA	ef-hand calcium-binding domain-containing protein 1	-2704	27.04	0.00
LDEC011404-RA	circadian clock-controlled	-263.94	85.98	0.33
LDEC013085-RA	PREDICTED: uncharacterized protein LOC103314347 isoform X2	-263.12	373.11	1.42
LDEC003232-RA	ammonium transporter 1 member 3-like	-262.92	50.38	0.19
LDEC017408-RA	15-hydroxyprostaglandin dehydrogenase	-2628	26.28	0.00
LDEC001450-RA	tpa_inf: hypothetical secreted protein 323	-2624	26.24	0.00
LDEC018413-RA	digestive cysteine proteinase 2	-2624	26.24	0.00
LDEC017772-RA	serine protease 53-like	-2614	26.14	0.00
LDEC023002-RA	pupal cuticle protein 20	-258.58	59.46	0.23
LDEC015696-RA	isoform a	-254.98	48.86	0.19
LDEC006291-RA	dynein heavy chain axonemal	-252.58	58.08	0.23
LDEC005660-RA	regulator of nonsense transcripts 2	-251.17	91.45	0.36
LDEC009916-RA	PREDICTED: uncharacterized protein LOC103312712 isoform X1	-247.62	56.94	0.23
LDEC008626-RA	hypothetical protein YQE_11743, partial	-247.61	137.60	0.56
LDEC016529-RA	coiled-coil domain-containing protein 65	-245.55	37.64	0.15
LDEC013810-RA	coiled-coil domain-containing protein 96	-243.43	32.65	0.13
LDEC007368-RA	hypothetical protein D910_09223	-239.42	87.17	0.36
LDEC002185-RA	flagellar attachment zone protein 1	-237.76	95.68	0.40
LDEC005072-RA	glycine-rich protein	-235.73	112.93	0.48
LDEC019296-RA	kelch-like protein 10-like	-231.57	48.81	0.21
LDEC009915-RA	PREDICTED: uncharacterized protein LOC103312712 isoform X2	-224.93	81.89	0.36
LDEC010196-RA	tubulin glycylase 3a-like isoform x2	-223.75	227.24	1.02
LDEC004151-RA	polycystic kidney disease protein 1-like 2	-215.55	128.05	0.59
LDEC010942-RA	soma ferritin	-206.52	344.31	1.67
LDEC013315-RA	forkhead box protein n5-like isoform x3	-204.57	82.32	0.40
LDEC000564-RA	kinesin-like protein kif9	-204.16	125.19	0.61

LDEC013433-RA	transmembrane protein 223	-201.18	69.39	0.34
LDEC013333-RA	coiled-coil domain-containing protein 39	-201.13	42.40	0.21
LDEC011849-RA	outer dense fiber protein 3	-194.36	186.22	0.96
LDEC002938-RA	serine threonine protein	-193.78	29.71	0.15
LDEC022648-RA	cytochrome oxidase subunit partial	-188.51	75.86	0.40
LDEC010002-RA	oxidative stress-induced growth inhibitor 2	-187.68	32.37	0.17
LDEC000005-RA	facilitated trehalose transporter tret1	-187.27	28.71	0.15
LDEC019915-RA	Rm62	-184.54	35.36	0.19
LDEC007374-RA	pancreatic lipase-related protein 2-like	-184.24	88.26	0.48
LDEC006520-RA	transcription factor sox-10-like isoform x2	-184.04	70.53	0.38
LDEC010109-RA	hypothetical protein YQE_12170, partial	-175.44	50.43	0.29
LDEC013847-RA	PREDICTED: uncharacterized protein LOC103307735	-173.17	36.50	0.21
LDEC000889-RA	sarcolemmal associated protein	-172.38	26.43	0.15
LDEC011224-RA	hypothetical protein TcasGA2_TC015485	-172.31	56.13	0.33
LDEC018212-RA	PREDICTED: uncharacterized protein LOC103314845	-172.02	102.19	0.59
LDEC008948-RA	peroxisomal n -acetyl-spermine spermidine oxidase	-166.60	38.31	0.23
LDEC018097-RA	polyadenylate-binding protein 1-like isoform 1	-158.58	91.16	0.57
LDEC002616-RA	agap012275-pa-like protein	-158.49	30.37	0.19
LDEC018543-RA	fibrous sheath cabyr-binding protein isoform x7	-149.48	42.97	0.29
LDEC010652-RA	coiled-coil domain-containing protein 61-like	-146.34	61.69	0.42
LDEC007016-RA	biogenesis of lysosome-related organelles complex 1 subunit 2-like	-141.06	148.67	1.05
LDEC003466-RA	e3 ubiquitin-protein ligase trim37-like	-136.79	52.43	0.38
LDEC012407-RA	tetratricopeptide repeat protein 25-like	-136.73	41.92	0.31
LDEC002987-RA	amine oxidase	-135.85	124.96	0.92
LDEC003953-RA	---NA---	-133.07	211.65	1.59
LDEC017395-RA	coiled-coil domain-containing protein 42 homolog	-132.29	248.44	1.88
LDEC019899-RA	protein claret segregational	-130.57	62.55	0.48
LDEC006915-RA	low quality protein: cilia- and flagella-associated protein 61-like	-127.62	26.90	0.21
LDEC002154-RA	radial spoke head protein 3 homolog	-124.55	66.83	0.54
LDEC012486-RA	c-type lectin mannose-binding isoform	-124.00	308.90	2.49
LDEC009664-RA	PREDICTED: uncharacterized protein LOC103312201	-121.54	55.89	0.46
LDEC009060-RA	upf0605 protein cg18335-like	-118.67	59.13	0.50
LDEC022237-RA	---NA---	-115.24	28.71	0.25
LDEC020593-RA	PREDICTED: uncharacterized protein LOC100574550	-114.27	30.66	0.27
LDEC016911-RA	c-jun-amino-terminal kinase-interacting	-113.60	76.19	0.67
LDEC011333-RA	kinesin-like protein kif16b	-112.44	38.78	0.34
LDEC008892-RA	transposable element tc3 transposase	-112.30	38.74	0.34
LDEC001158-RA	organic cation transporter 1-like isoform x2	-111.81	27.85	0.25
LDEC004032-RA	toxin 1	-107.75	88.79	0.82

LDEC017271-RA	esterase fe4	-104.04	189.41	1.82
LDEC010912-RA	proline-rich protein 4-like	-103.92	115.50	1.11
LDEC019556-RA	fatty acyl- reductase 1-like	-102.72	137.79	1.34
LDEC012498-RA	proton-associated sugar transporter a-like	-99.21	32.32	0.33
LDEC003077-RA	odorant-binding protein 4	-96.93	70.58	0.73
LDEC018944-RA	juvenile hormone esterase isoform a	-92.58	70.96	0.77
LDEC024566-RA	hypothetical protein YQE_00236, partial	-92.00	38.78	0.42
LDEC015659-RA	glycosyl hydrolase	-91.58	45.63	0.50
LDEC009511-RA	cilia- and flagella-associated protein 69-like	-89.49	42.87	0.48
LDEC010535-RA	hydrocephalus-inducing protein homolog	-89.29	39.35	0.44
LDEC004769-RA	alpha-tocopherol transfer	-85.52	72.10	0.84
LDEC007120-RA	a disintegrin and metalloproteinase with thrombospondin motifs 7-like	-84.88	29.28	0.34
LDEC017402-RA	centromere-associated protein e-like	-84.56	35.65	0.42
LDEC021792-RA	wd-repeat protein	-83.46	27.19	0.33
LDEC007589-RA	formin-like protein 8	-82.65	578.06	6.99
LDEC001375-RA	retinol dehydrogenase 12	-82.32	33.13	0.40
LDEC006746-RA	gamma-glutamyltranspeptidase 1-like isoform x2	-81.21	54.47	0.67
LDEC007298-RA	wd repeat-containing protein 34-like	-79.28	41.02	0.52
LDEC002739-RA	tektin-b1-like isoform x2	-76.41	38.07	0.50
LDEC017615-RA	titin-like isoform x2	-74.32	75.48	1.02
LDEC018239-RA	hypothetical protein TcasGA2_TC012239	-73.79	33.94	0.46
LDEC001263-RA	extracellular signal-regulated kinase 2	-73.51	66.21	0.90
LDEC003152-RA	protein disulfide-isomerase a3	-72.11	37.31	0.52
LDEC007037-RA	PREDICTED: uncharacterized protein LOC103312559	-71.33	34.17	0.48
LDEC002366-RA	aael014823- partial	-66.91	115.40	1.72
LDEC017258-RA	zinc finger bed domain-containing protein 5-like	-66.35	30.51	0.46
LDEC023633-RA	hypothetical protein YQE_08895, partial	-65.08	119.73	1.84
LDEC009902-RA	dynein heavy chain axonemal	-64.19	51.66	0.80
LDEC009941-RA	phosphoglycerate kinase	-63.97	447.44	6.99
LDEC002699-RA	protein maats1-like	-62.31	59.70	0.96
LDEC019154-RA	chemosensory protein	-61.31	37.60	0.61
LDEC008950-RA	spermine oxidase-like	-60.67	27.90	0.46
LDEC017272-RA	venom carboxylesterase-6-like	-57.16	123.77	2.17
LDEC000713-RA	cytosolic carboxypeptidase 2-like isoform x1	-56.81	79.47	1.40
LDEC020979-RA	troponin isoform 1-like	-56.42	59.46	1.05
LDEC024010-RA	isoform a	-54.80	2110.60	38.52
LDEC000464-RA	coiled-coil domain-containing protein 108-like isoform x3	-54.47	54.28	1.00
LDEC016516-RA	venom allergen 3-like	-53.12	95.68	1.80
LDEC014638-RA	cytochrome p450 9z4	-52.68	806.53	15.31
LDEC007771-RA	unknown	-52.66	166.50	3.16

LDEC012839-RA	odorant-binding protein 56a	-52.55	273.91	5.21
LDEC020140-RA	isoform a	-51.79	49.62	0.96
LDEC000165-RA	cyclin-dependent kinase inhibitor 1b-like isoform x4	-50.36	123.53	2.45
LDEC011227-RA	hypothetical protein YQE_05602, partial	-50.10	38.40	0.77
LDEC004633-RA	digestive cysteine protease intestain	-49.93	36.36	0.73
LDEC012958-RA	alkylglycerol monooxygenase-like	-49.90	572.83	11.48
LDEC008843-RA	prostatic acid phosphatase	-46.82	50.24	1.07
LDEC015419-RA	palmitoyltransferase zdhhc17-like	-46.36	101.29	2.18
LDEC009877-RA	retinol dehydrogenase 13	-45.34	158.99	3.51
LDEC005954-RA	probable inactive protein kinase ddb_g0270444-like isoform x2	-45.20	31.18	0.69
LDEC001845-RA	alpha-tocopherol transfer	-44.90	524.82	11.69
LDEC014458-RA	dual specificity protein phosphatase cdc14a-like	-44.48	116.78	2.63
LDEC017158-RA	hypothetical protein TcasGA2_TC014108	-44.46	34.93	0.79
LDEC011974-RA	agap011897-pa-like protein	-43.72	29.33	0.67
LDEC017037-RA	myosin light chain smooth muscle	-43.33	144.49	3.33
LDEC010726-RA	15-hydroxyprostaglandin dehydrogenase	-42.92	165.31	3.85
LDEC007337-RA	agap000696-pa-like protein	-42.48	122.91	2.89
LDEC013714-RA	probable multidrug resistance-associated protein lethal 03659	-41.78	31.23	0.75
LDEC022047-RA	cyclin-dependent kinase-like 4 isoform x1	-40.86	43.06	1.05
LDEC019798-RA	gastrula zinc finger protein	-40.31	222.44	5.52
LDEC005620-RA	ankyrin-1-like isoform x3	-40.03	247.01	6.17
LDEC000656-RA	agap013007-pa-like protein	-39.55	134.89	3.41
LDEC017273-RA	juvenile hormone esterase	-39.25	180.52	4.60
LDEC011194-RA	heat shock protein 68	-38.90	65.59	1.69
LDEC012371-RA	phosphatidate phosphatase ppapdc1a-like	-38.59	87.26	2.26
LDEC017035-RA	PREDICTED: uncharacterized protein LOC105205580	-38.23	30.04	0.79
LDEC011150-RA	lish domain-containing protein fopnl-like	-37.75	39.78	1.05
LDEC016770-RA	cytochrome p450 4c1-like	-37.41	104.66	2.80
LDEC001996-RA	mushroom body large-type kenyon cell-specific protein 1	-36.80	248.25	6.75
LDEC022019-RA	tpa: cuticle protein	-35.80	185.89	5.19
LDEC009369-RA	---NA---	-35.59	174.58	4.91
LDEC017038-RA	nuclear anchorage protein 1-like	-35.49	601.87	16.96
LDEC017225-RA	lysoplasmalogenase-like protein tmem86a	-35.41	154.71	4.37
LDEC000793-RA	pro-phenol oxidase subunit 2	-35.32	33.84	0.96
LDEC009046-RA	neuropilin and tolloid-like protein 2-like	-35.21	51.95	1.48
LDEC000495-RA	cytochrome c oxidase subunit partial	-34.84	30.04	0.86
LDEC002396-RA	fatty acid hydroxylase domain-containing protein 2 isoform x2	-34.34	286.22	8.34
LDEC021001-RA	collagen alpha-1	-34.31	51.28	1.49
LDEC010052-RA	glucose dehydrogenase	-34.20	30.80	0.90
LDEC013182-RA	chemosensory protein	-34.10	31.37	0.92

LDEC021699-RA	cuticle protein precursor	-33.56	30.23	0.90
LDEC014637-RA	PREDICTED: uncharacterized protein K02A2.6-like	-33.39	77.43	2.32
LDEC005827-RA	PREDICTED: uncharacterized protein LOC103568218 isoform X3	-32.67	2233.61	68.37
LDEC000159-RA	multiple epidermal growth factor-like domains protein 10	-32.40	90.02	2.78
LDEC002282-RA	arrestin domain-containing protein 3	-32.32	103.42	3.20
LDEC013734-RA	vacuolar protein sorting-associated protein 27 isoform x2	-32.22	419.83	13.03
LDEC021135-RA	4-aminobutyrate mitochondrial	-31.88	45.20	1.42
LDEC003361-RA	proton-coupled amino acid transporter 4 isoform x1	-31.73	119.77	3.78
LDEC015711-RA	upf0573 protein c2orf70 homolog	-31.55	49.57	1.57
LDEC005036-RA	dynein heavy chain axonemal	-31.54	82.80	2.63
LDEC022054-RA	hypothetical protein YQE_00535, partial	-31.21	28.71	0.92
LDEC012659-RA	glutamate--cysteine ligase catalytic subunit	-30.90	33.75	1.09
LDEC014525-RA	heat shock protein 70	-30.89	129.04	4.18
LDEC006855-RA	colostrum trypsin	-30.66	59.93	1.95
LDEC011676-RA	nuak family snf1-like kinase 1	-30.60	34.60	1.13
LDEC005679-RA	cuticular protein ld-cp1v1	-30.05	693.22	23.07
LDEC014189-RA	lipase member k-like	-29.95	52.81	1.76
LDEC016685-RA	sodium channel and clathrin linker 1-like isoform x2	-29.31	46.06	1.57
LDEC014484-RA	PREDICTED: uncharacterized protein LOC103315055	-29.17	37.45	1.28
LDEC004124-RA	hypothetical protein YQE_08744, partial	-28.91	35.46	1.23
LDEC006671-RA	serine protease snake-like	-28.87	603.48	20.91
LDEC016339-RA	centrosomal protein of 290 kda	-28.85	33.18	1.15
LDEC009154-RA	hypothetical protein YQE_09144, partial	-28.75	118.44	4.12
LDEC011807-RA	transcription factor rfx3 isoform x1	-28.31	28.76	1.02
LDEC006695-RA	PREDICTED: titin	-28.21	31.89	1.13
LDEC003208-RA	perq amino acid-rich with gyf domain-containing protein 2-like	-28.12	229.05	8.14
LDEC007302-RA	glutamine synthetase 2	-28.09	62.98	2.24
LDEC015622-RA	glutathione s-transferase	-27.93	71.72	2.57
LDEC021671-RA	cuticle protein 6	-27.71	132.23	4.77
LDEC009036-RA	ef-hand domain-containing protein 1-like	-27.58	80.85	2.93
LDEC008529-RA	ropporin-1-like protein	-27.57	54.42	1.97
LDEC011677-RA	equilibrative nucleoside transporter 1	-27.33	295.92	10.83
LDEC000226-RA	tyrosine hydroxylase	-26.55	2536.90	95.56
LDEC003958-RA	glucose dehydrogenase	-26.52	31.51	1.19
LDEC018522-RA	lipase 3	-25.97	59.22	2.28
LDEC017761-RA	f-box only protein 25	-25.29	66.40	2.63
LDEC009889-RA	yellow-e precursor	-25.15	379.29	15.08
LDEC007768-RA	potassium sodium hyperpolarization-activated cyclic nucleotide-gated channel 1-like	-25.00	129.33	5.17
LDEC010538-RA	hydrocephalus-inducing protein homolog	-24.65	44.87	1.82

LDEC021228-RA	gastrula zinc finger protein	-23.85	37.93	1.59
LDEC000604-RA	---NA---	-23.23	106.85	4.60
LDEC015570-RA	lysosomal pro-x carboxypeptidase	-23.19	34.22	1.48
LDEC006337-RA	protein yellow	-23.15	78.52	3.39
LDEC015776-RA	headcase protein	-22.86	592.17	25.91
LDEC005826-RA	PREDICTED: hypothetical protein LOC100748865	-22.33	1190.38	53.31
LDEC010330-RA	PREDICTED: uncharacterized protein LOC656470	-22.05	530.34	24.05
LDEC020176-RA	cytochrome p450 9z4	-21.96	3606.89	164.22
LDEC014237-RA	dentin sialophospho isoform x1	-21.86	66.59	3.05
LDEC013868-RA	ring finger protein 145	-21.60	31.04	1.44
LDEC006967-RA	glyceraldehyde-3-phosphate dehydrogenase	-21.32	48.62	2.28
LDEC008186-RA	PREDICTED: uncharacterized protein LOC103521349, partial	-20.93	64.97	3.10
LDEC000589-RA	protein croquemort-like	-20.77	96.72	4.66
LDEC007522-RA	leucine-rich repeat protein soc-2 isoform x1	-20.22	474.30	23.45
LDEC021642-RA	muscle m-line assembly protein unc-89-like	-19.63	34.60	1.76
LDEC017005-RA	pollen-specific leucine-rich repeat extensin-like protein 1	-19.56	3892.73	199.04
LDEC002534-RA	sjoegren syndrome nuclear autoantigen 1 homolog	-19.55	35.22	1.80
LDEC000852-RA	inactive pancreatic lipase-related protein 1 isoform x1	-18.73	52.05	2.78
LDEC006974-RA	low density lipoprotein receptor adapter protein 1	-18.47	102.66	5.56
LDEC006841-RA	b-box type zinc finger protein ncl-1-like isoform x1	-18.43	32.13	1.74
LDEC018881-RA	differentially expressed in fdcp 6-like protein	-18.39	177.62	9.66
LDEC020001-RA	PREDICTED: uncharacterized protein LOC655780	-18.18	40.07	2.20
LDEC018992-RA	glycine-rich cell wall structural protein	-18.14	38.93	2.15
LDEC001254-RA	zinc finger	-17.98	37.22	2.07
LDEC016916-RA	15-hydroxyprostaglandin dehydrogenase	-17.90	246.30	13.76
LDEC002431-RA	hypothetical protein TcasGA2_TC007080	-17.90	256.52	14.33
LDEC011244-RA	cytoplasmic a3	-17.83	34.51	1.94
LDEC011680-RA	equilibrative nucleoside transporter 3	-17.44	54.14	3.10
LDEC011854-RA	cysteine and histidine-rich protein 1 homolog	-17.42	308.04	17.69
LDEC023675-RA	cytochrome p450	-17.33	639.46	36.91
LDEC014701-RA	tbc1 domain family member 9	-17.13	102.76	6.00
LDEC005680-RA	cuticular protein 100a	-17.09	154.23	9.03
LDEC014399-RA	cuticle protein	-17.04	171.44	10.06
LDEC006641-RA	kinesin-like protein klp10a	-16.85	58.13	3.45
LDEC018266-RA	ubiquitin-like-conjugating enzyme atg10 isoform x3	-16.74	40.73	2.43
LDEC008217-RA	fas-binding factor 1	-16.67	45.68	2.74
LDEC002004-RA	e3 ubiquitin-protein ligase march2-like	-16.63	65.64	3.95
LDEC022959-RA	leukocyte elastase inhibitor-like	-16.41	39.92	2.43
LDEC014730-RA	lanc-like protein 3 homolog	-16.36	106.89	6.53

LDEC014642-RA	cytochrome p450 9z4	-16.21	64.59	3.99
LDEC002923-RA	nucleic-acid-binding protein from transposon x-element	-16.02	85.65	5.35
LDEC019378-RA	myosin light chain smooth muscle	-15.99	37.07	2.32
LDEC002239-RA	e3 ubiquitin-protein ligase siah1-like	-15.73	121.20	7.70
LDEC005752-RA	PREDICTED: uncharacterized protein LOC657906	-15.73	40.40	2.57
LDEC012380-RA	isoform s	-15.37	111.88	7.28
LDEC017558-RA	esterase fe4-like	-15.36	61.22	3.99
LDEC021362-RA	hypothetical protein D910_11256	-14.96	131.04	8.76
LDEC021048-RA	alpha-tubulin n-acetyltransferase-like	-14.92	291.69	19.55
LDEC007332-RA	dna-directed rna polymerase i subunit rpa1 isoform x1	-14.87	75.24	5.06
LDEC018530-RA	transcriptional regulator atrx homolog	-14.75	37.88	2.57
LDEC003189-RA	nadh dehydrogenase	-14.72	246.82	16.77
LDEC023689-RA	b1 protein	-14.62	39.78	2.72
LDEC013341-RA	leucine-rich repeat	-14.62	47.62	3.26
LDEC021327-RA	dnaj homolog subfamily c member 16-like	-14.54	530.53	36.49
LDEC022010-RA	venom carboxylesterase-6-like	-14.39	167.68	11.65
LDEC000838-RA	#NAME?	-14.29	145.44	10.18
LDEC005012-RA	dynein intermediate chain ciliary	-14.20	43.25	3.05
LDEC003983-RA	rwd domain-containing protein 2a	-14.17	146.11	10.31
LDEC006021-RA	cytochrome p450 6bq10	-14.14	175.86	12.44
LDEC003539-RA	gamma-interferon-inducible lysosomal thiol reductase-like	-13.92	33.60	2.41
LDEC017394-RA	coiled-coil domain-containing protein 42 homolog	-13.74	35.03	2.55
LDEC005885-RA	chitinase-3-like protein 1	-13.71	49.15	3.58
LDEC022770-RA	cytochrome p450 9z4	-13.67	286.60	20.96
LDEC011540-RA	zinc finger ccch domain-containing protein 3	-13.67	217.92	15.94
LDEC018960-RA	ac transposable element-derived protein partial	-13.55	72.96	5.38
LDEC015015-RA	traf-interacting partial	-13.51	68.11	5.04
LDEC010733-RA	apolipoprotein d-like	-13.47	471.16	34.97
LDEC017614-RA	titin-like isoform x1	-13.47	1404.03	104.24
LDEC015331-RA	peptidoglycan-recognition protein lc-like isoform x1	-13.25	45.20	3.41
LDEC021326-RA	defensin precursor	-13.21	1793.68	135.82
LDEC018156-RA	zinc finger and btb domain-containing protein 49-like isoform x2	-13.05	44.25	3.39
LDEC004413-RA	asparagine--trna cytoplasmic	-13.01	39.64	3.05
LDEC023820-RA	cytochrome p450 9e2-like	-12.97	163.60	12.61
LDEC008288-RA	serine threonine-protein phosphatase 6 regulatory ankyrin repeat subunit a-like	-12.96	44.96	3.47
LDEC003028-RA	ubiquitin-conjugating enzyme e2-230k	-12.90	58.32	4.52
LDEC004651-RA	dynein heavy chain	-12.88	224.53	17.44
LDEC000703-RA	antifreeze protein maxi	-12.87	111.46	8.66
LDEC013680-RA	nucleoporin-like 2	-12.81	182.37	14.24
LDEC000590-RA	protein croquemort-like isoform x3	-12.79	95.06	7.44

LDEC012340-RA	trichohyalin isoform x1	-12.77	76.10	5.96
LDEC010249-RA	band 7 protein agap004871	-12.68	44.96	3.55
LDEC003959-RA	glucose dehydrogenase	-12.63	88.31	6.99
LDEC022674-RA	cytochrome p450 9z4	-12.58	590.46	46.95
LDEC011389-RA	nose resistant to fluoxetine protein 6-like isoform x1	-12.55	48.81	3.89
LDEC009352-RA	---NA---	-12.50	63.97	5.12
LDEC000615-RA	protein lethal essential for life	-12.49	33.75	2.70
LDEC001153-RA	gpi-anchored wall transfer protein 1	-12.42	84.94	6.84
LDEC015038-RA	toll-like receptor partial	-12.40	100.05	8.07
LDEC020707-RA	major facilitator superfamily protein	-12.34	51.57	4.18
LDEC003765-RA	GI16351	-12.34	39.26	3.18
LDEC000998-RA	protein takeout-like	-12.17	44.54	3.66
LDEC010172-RA	hormone receptor 4 isoform x2	-11.98	168.54	14.07
LDEC018613-RA	leucine-rich repeat-containing protein ddb_g0290503-like isoform x1	-11.98	129.23	10.79
LDEC023570-RA	4-coumarate-- ligase 1-like	-11.94	40.97	3.43
LDEC016716-RA	rna-binding protein 40	-11.94	122.63	10.27
LDEC007898-RA	PREDICTED: uncharacterized protein LOC103312446	-11.91	45.20	3.79
LDEC013805-RA	cryptochrome 2	-11.82	129.28	10.94
LDEC005786-RA	nesprin-1 isoform x1	-11.79	1124.46	95.39
LDEC006490-RA	hypothetical protein	-11.74	193.45	16.48
LDEC003011-RA	tetratricopeptide repeat protein 36 homolog	-11.69	107.27	9.18
LDEC006084-RA	transcription factor adf-1	-11.64	83.18	7.15
LDEC013528-RA	protein tprxl isoform x2	-11.47	121.34	10.58
LDEC017119-RA	udp-glucuronosyltransferase 2b17-like	-11.38	207.66	18.24
LDEC003633-RA	sodium-coupled neutral amino acid transporter 9	-11.34	61.74	5.44
LDEC012598-RA	peroxidase-like isoform 1	-11.31	51.14	4.52
LDEC020757-RA	cytochrome p450	-11.30	655.72	58.01
LDEC011815-RA	yellow-c precursor	-11.27	74.10	6.57
LDEC007706-RA	cytochrome p450 4c1 isoform x2	-11.25	63.83	5.67
LDEC014933-RA	hypothetical protein YQE_10880, partial	-11.19	36.88	3.30
LDEC007227-RA	a-kinase anchor protein 9-like isoform x1	-11.15	110.70	9.93
LDEC001894-RA	zinc finger protein partial	-11.12	74.57	6.71
LDEC003377-RA	protein henna	-11.11	184.37	16.59
LDEC002630-RA	hypothetical protein YQE_07290, partial	-11.07	138.31	12.49
LDEC020674-RA	zinc finger protein 2 homolog	-11.04	68.73	6.23
LDEC008282-RA	serine threonine-protein phosphatase 6 regulatory ankyrin repeat subunit a-like	-10.93	52.38	4.79
LDEC000787-RA	arylsulfatase b-like	-10.91	60.41	5.54
LDEC012660-RA	protein prenyltransferase alpha subunit repeat-containing protein 1-b	-10.85	571.73	52.68
LDEC017739-RA	inorganic phosphate cotransporter	-10.85	34.93	3.22
LDEC010678-RA	e3 ubiquitin-protein ligase topors	-10.82	61.60	5.69

LDEC006426-RA	PREDICTED: mucin-4	-10.77	197.39	18.32
LDEC010047-RA	inner centromere protein a-like	-10.76	78.76	7.32
LDEC007954-RA	general receptor for phosphoinositides 1-associated scaffold protein	-10.67	168.54	15.79
LDEC018662-RA	inorganic phosphate cotransporter	-10.61	38.64	3.64
LDEC011325-RA	zinc finger protein 271-like	-10.50	41.64	3.97
LDEC013907-RA	f-box lrr-repeat protein 20 isoform x3	-10.42	43.73	4.20
LDEC002253-RA	protein fam151b isoform x1	-10.42	323.34	31.04
LDEC017269-RA	pickpocket protein 28-like	-10.35	41.26	3.99
LDEC005521-RA	protein takeout-like	-10.35	509.66	49.25
LDEC011291-RA	---NA---	-10.31	40.50	3.93
LDEC018503-RA	staga complex 65 subunit gamma-like isoform x2	-10.30	56.66	5.50
LDEC013578-RA	serine threonine-protein phosphatase 4 regulatory subunit 1-like isoform x3	-10.28	114.83	11.17
LDEC006371-RA	u11 u12 small nuclear ribonucleoprotein 48 kda	-10.27	52.14	5.08
LDEC004019-RA	circadian protein clock arnt bmal pas	-10.25	79.18	7.72
LDEC010802-RA	atm interactor	-10.20	111.27	10.90
LDEC003594-RA	tyrosine-protein phosphatase non-receptor	-10.17	560.52	55.11
LDEC004287-RA	inositol hexakisphosphate kinase 1 isoform x2	-10.10	97.34	9.64
LDEC020758-RA	cytochrome p450 9e2	-10.10	400.30	39.65
LDEC007955-RA	general receptor for phosphoinositides 1-associated scaffold protein	-9.89	96.30	9.73
LDEC004855-RA	retrovirus-related pol polyprotein from transposon partial	-9.83	56.89	5.79
LDEC015737-RA	chitin deacetylase 5 isoform x5	-9.76	571.26	58.54
LDEC019590-RA	aminopeptidase n	-9.75	228.24	23.40
LDEC009065-RA	PREDICTED: uncharacterized protein LOC100141997	-9.75	133.94	13.74
LDEC002494-RA	cytochrome p450 6k1	-9.73	115.26	11.84
LDEC010754-RA	hypothetical protein DAPPUDRAFT_15773	-9.70	249.72	25.75
LDEC004707-RA	pdz domain protein	-9.66	56.28	5.83
LDEC018558-RA	dentin sialophosphoprotein isoform x3	-9.63	130.09	13.51
LDEC005125-RA	calmodulin-binding transcription activator 2-like isoform x3	-9.62	69.11	7.19
LDEC019373-RA	stretchin- isoform g	-9.48	356.24	37.58
LDEC004632-RA	digestive cysteine protease intestain	-9.46	203.47	21.52
LDEC023114-RA	upf0420 protein c16orf58 homolog	-9.41	250.34	26.60
LDEC008059-RA	probable ribonuclease p mrp protein subunit pop5-like	-9.40	39.97	4.25
LDEC019660-RA	meiosis-specific nuclear structural protein 1-like	-9.32	63.93	6.86
LDEC021910-RA	probable low-specificity l-threonine aldolase 2 isoform x1	-9.31	76.14	8.18
LDEC016293-RA	non-histone protein 10-like	-9.30	43.11	4.64
LDEC014644-RA	cytochrome p450 9z4	-9.24	67.63	7.32
LDEC013672-RA	transcriptional-regulating factor 1 isoform x2	-9.18	116.12	12.65
LDEC002903-RA	PREDICTED: uncharacterized protein LOC664170 isoform X1	-9.11	68.97	7.57
LDEC021034-RA	ubiquitin carboxyl-terminal hydrolase 2-like isoform x2	-9.09	135.22	14.87

LDEC015907-RA	laccase isoform f	-9.08	276.67	30.47
LDEC003632-RA	sodium-coupled neutral amino acid transporter 9	-9.05	81.37	8.99
LDEC003593-RA	tyrosine-protein phosphatase non-receptor	-9.05	70.44	7.78
LDEC002250-RA	brca1-a complex subunit abraxas-like	-9.05	53.76	5.94
LDEC018663-RA	inorganic phosphate cotransporter	-9.02	75.05	8.32
LDEC018375-RA	enkurin domain-containing protein 1 isoform x1	-8.95	58.32	6.52
LDEC005274-RA	protein fam179b-like	-8.88	71.63	8.07
LDEC022111-RA	hypothetical protein TcasGA2_TC000293	-8.78	53.99	6.15
LDEC006966-RA	reverse partial	-8.60	1042.61	121.24
LDEC001049-RA	aminopeptidase n	-8.60	1301.98	151.48
LDEC001926-RA	PREDICTED: uncharacterized protein LOC103313770	-8.57	100.48	11.73
LDEC005610-RA	atrophin-1 isoform x1	-8.52	429.43	50.38
LDEC001186-RA	eh domain-containing protein 3	-8.52	50.43	5.92
LDEC015277-RA	nck-interacting protein with sh3 domain	-8.50	41.68	4.91
LDEC014731-RA	thymidylate kinase	-8.44	64.88	7.68
LDEC006252-RA	phosphatidylinositol 3-kinase catalytic subunit type 3	-8.37	78.76	9.41
LDEC023555-RA	mucin-2-like isoform x1	-8.29	91.21	11.00
LDEC013733-RA	tyrosine-protein phosphatase non-receptor type 23	-8.27	88.31	10.67
LDEC008478-RA	embryonic polarity	-8.22	77.85	9.47
LDEC013049-RA	zinc finger protein 271-like	-8.22	53.57	6.52
LDEC003056-RA	PREDICTED: uncharacterized protein LOC660988 isoform X2	-8.17	139.78	17.11
LDEC018222-RA	cytochrome p450 4c1	-8.14	182.89	22.46
LDEC003408-RA	zinc finger protein ozf	-8.14	43.82	5.38
LDEC008866-RA	chromobox protein partial	-8.06	112.84	14.01
LDEC009691-RA	wd repeat-containing protein 81 isoform x1	-8.04	100.57	12.51
LDEC000682-RA	protein turtle isoform x1	-8.03	574.16	71.53
LDEC018698-RA	cytochrome p450 9z4	-8.01	156.23	19.51
LDEC006124-RA	cadherin 23	-7.99	60.79	7.61
LDEC010191-RA	---NA---	-7.98	52.90	6.63
LDEC023354-RA	polyadenylation factor	-7.96	69.73	8.76
LDEC006388-RA	zinc finger protein 704-like	-7.96	508.71	63.93
LDEC009552-RA	ankyrin-3-like isoform x4	-7.93	82.80	10.44
LDEC023320-RA	c-1-tetrahydrofolate cytoplasmic isoform x2	-7.92	615.99	77.82
LDEC007506-RA	pancreatic triacylglycerol lipase-like	-7.91	48.34	6.11
LDEC019778-RA	c-1-tetrahydrofolate cytoplasmic	-7.83	1090.52	139.20
LDEC017616-RA	ankyrin-2-like isoform x5	-7.82	39.40	5.04
LDEC011361-RA	superoxide dismutase	-7.77	68.49	8.81
LDEC012229-RA	hypothetical protein TcasGA2_TC001876	-7.75	172.82	22.29
LDEC009571-RA	myosin heavy non-muscle isoform x3	-7.73	112.65	14.56
LDEC015039-RA	insulin-like growth factor-binding protein complex acid labile partial	-7.73	156.04	20.20

LDEC019779-RA	c-1-tetrahydrofolate cytoplasmic isoform x2	-7.72	5026.93	651.32
LDEC007026-RA	PREDICTED: uncharacterized protein LOC103314916	-7.71	53.04	6.88
LDEC013895-RA	acyl- synthetase family member mitochondrial	-7.69	95.25	12.38
LDEC005792-RA	biogenesis of lysosome-related organelles complex 1 subunit 6-like isoform x1	-7.68	40.50	5.27
LDEC002657-RA	4-coumarate-- ligase 1-like	-7.58	45.20	5.96
LDEC020581-RA	m13 peptidase	-7.56	110.32	14.58
LDEC009690-RA	wd repeat-containing protein 81 isoform x2	-7.53	42.11	5.60
LDEC012022-RA	zinc finger protein 271-like	-7.53	42.11	5.60
LDEC013154-RA	PREDICTED: uncharacterized protein LOC103312202 isoform X2	-7.53	59.13	7.86
LDEC003879-RA	dynein heavy chain axonemal	-7.52	194.35	25.83
LDEC001227-RA	rna-binding protein pno1	-7.49	68.35	9.12
LDEC014022-RA	bromodomain-containing protein 4-like	-7.45	339.08	45.49
LDEC013287-RA	cytochrome p450 9z4	-7.44	3099.70	416.81
LDEC018699-RA	cytochrome p450 9e2	-7.41	146.87	19.83
LDEC015045-RA	prkr-interacting protein 1 homolog	-7.35	72.15	9.81
LDEC011739-RA	ribosome biogenesis protein wdr12 homolog	-7.30	383.09	52.49
LDEC008741-RA	tubby-related protein 1	-7.27	805.87	110.86
LDEC003747-RA	4-coumarate-- ligase 1-like	-7.25	96.96	13.38
LDEC000801-RA	juvenile hormone-inducible protein	-7.11	431.00	60.61
LDEC005612-RA	comm domain-containing protein 3	-7.09	94.01	13.26
LDEC010707-RA	hypothetical protein D910_06287	-7.07	134.18	18.99
LDEC011738-RA	nuclear pore complex protein nup214	-7.05	123.15	17.46
LDEC007274-RA	spermatogenesis-associated protein 13-like isoform x2	-7.05	56.89	8.07
LDEC006286-RA	nuclear factor of activated t-cells 5	-7.03	102.71	14.60
LDEC005137-RA	reverse transcriptase	-7.02	52.47	7.47
LDEC000984-RA	leucine-rich repeat-containing protein 58-like	-7.01	260.42	37.18
LDEC005788-RA	muscle-specific protein 300	-7.00	54.99	7.86
LDEC017560-RA	esterase fe4-like	-7.00	179.52	25.66
LDEC011736-RA	nuclear pore complex protein nup214	-6.96	79.47	11.42
LDEC004545-RA	mariner transposase	-6.93	475.49	68.60
LDEC010559-RA	rrna-processing protein utp23 homolog	-6.90	80.28	11.63
LDEC021544-RA	serine incorporator	-6.88	188.55	27.40
LDEC014183-RA	rna polymerase ii elongation factor ell-like	-6.86	574.92	83.80
LDEC009801-RA	titin isoform x1	-6.83	2942.42	430.64
LDEC009244-RA	dual specificity protein phosphatase 21	-6.83	59.13	8.66
LDEC006589-RA	zinc finger ccch-type with g patch domain-containing	-6.79	50.38	7.42
LDEC014114-RA	glycogen-binding subunit 76a	-6.77	46.34	6.84
LDEC015590-RA	protein cnppd1-like	-6.76	198.86	29.41
LDEC000938-RA	inositol-tetrakisphosphate 1-kinase	-6.75	60.32	8.93
LDEC016412-RA	zinc finger protein 260	-6.72	91.45	13.61

LDEC011517-RA	sporulation-specific protein 15	-6.71	199.81	29.76
LDEC015271-RA	syntaxin-1a-like isoform x2	-6.71	112.84	16.82
LDEC013607-RA	slit homolog 2 protein	-6.68	52.47	7.86
LDEC007871-RA	zinc finger protein 277	-6.66	80.23	12.05
LDEC000634-RA	isoform a	-6.64	340.79	51.36
LDEC019089-RA	l-allo-threonine aldolase	-6.62	103.33	15.60
LDEC022146-RA	sprt-like domain-containing protein spartan	-6.62	46.67	7.05
LDEC015705-RA	fat-like cadherin-related tumor suppressor homolog isoform x1	-6.61	62.07	9.39
LDEC004584-RA	cold shock domain-containing protein e1	-6.60	1410.11	213.59
LDEC009315-RA	ly-6 neurotoxin-related protein	-6.57	172.96	26.33
LDEC008306-RA	protein daughter of sevenless	-6.57	46.82	7.13
LDEC020945-RA	ubiquitin-like protein 3	-6.54	126.76	19.37
LDEC007361-RA	protein takeout	-6.54	98.91	15.12
LDEC024223-RA	c-1-tetrahydrofolate cytoplasmic	-6.53	527.91	80.90
LDEC021242-RA	immunoglobulin-binding protein 1-like	-6.51	217.97	33.48
LDEC007229-RA	protein lethal essential for life	-6.50	59.89	9.22
LDEC017718-RA	low quality protein: probable e3 ubiquitin-protein ligase mycbp2-like	-6.50	51.66	7.95
LDEC003612-RA	werner syndrome helicase	-6.49	93.63	14.43
LDEC001389-RA	gaba-gated ion channel	-6.49	435.85	67.18
LDEC003621-RA	zinc finger cchc-type and rna-binding motif-containing protein 1-like	-6.48	44.44	6.86
LDEC006546-RA	zinc finger protein 658-like isoform x1	-6.48	70.25	10.85
LDEC016420-RA	esterase	-6.47	55.09	8.51
LDEC006606-RA	cytochrome p450 9e2-like	-6.45	192.88	29.91
LDEC013638-RA	histone h2a deubiquitinase mysm1-like	-6.39	85.27	13.34
LDEC008571-RA	ubiquitin-conjugating enzyme e2 w	-6.39	120.77	18.91
LDEC007754-RA	PREDICTED: uncharacterized protein LOC100141968	-6.36	148.25	23.30
LDEC020285-RA	conserved protein	-6.34	55.28	8.72
LDEC015906-RA	laccase 2 precursor	-6.32	46.01	7.28
LDEC017884-RA	hypothetical protein G5I_14800	-6.30	233.61	37.06
LDEC016116-RA	dynactin subunit 3-like	-6.29	129.85	20.66
LDEC010195-RA	upf0430 protein cg31712	-6.29	54.80	8.72
LDEC004655-RA	serine threonine-protein phosphatase 4 regulatory subunit 4-like	-6.27	121.91	19.43
LDEC004770-RA	venom carboxylesterase-6-like	-6.26	299.48	47.81
LDEC003628-RA	zinc finger protein partial	-6.24	104.23	16.69
LDEC008073-RA	protein son-like isoform x1	-6.23	70.91	11.38
LDEC006327-RA	72 kda inositol polyphosphate 5-phosphatase isoform x1	-6.22	50.33	8.09
LDEC001712-RA	inverted formin-2	-6.22	346.02	55.63
LDEC006694-RA	large proline-rich protein bag6	-6.21	131.99	21.25
LDEC001175-RA	tpa: cuticle protein	-6.20	222.91	35.93
LDEC006995-RA	leukocyte receptor tyrosine protein kinase	-6.16	75.48	12.24
LDEC008551-RA	transcription initiation factor tfiid	-6.16	80.28	13.03

	subunit 5-like			
LDEC010460-RA	zinc finger protein partial	-6.16	45.06	7.32
LDEC003267-RA	thyrotropin-releasing hormone receptor isoform x3	-6.15	130.04	21.14
LDEC003397-RA	bifunctional purine biosynthesis protein purh	-6.12	1476.61	241.10
LDEC010167-RA	protein tyrosine non-receptor type nt6	-6.12	52.19	8.53
LDEC006249-RA	phosphatidylinositol 3-kinase catalytic subunit type 3	-6.10	182.85	29.99
LDEC007929-RA	dna polymerase beta-like	-6.10	70.44	11.56
LDEC010792-RA	facilitated trehalose transporter tret1-2 homolog	-6.08	212.03	34.88
LDEC007829-RA	transcriptional protein swt1	-6.08	72.77	11.98
LDEC010245-RA	polynucleotide kinase- 3 -phosphatase	-6.07	357.95	58.94
LDEC012858-RA	camp-dependent protein kinase type ii regulatory subunit	-6.07	68.02	11.21
LDEC020860-RA	nuclear excision repair protein rad23	-6.05	163.26	27.00
LDEC009353-RA	tubulin polyglutamylase ttll5	-6.03	384.42	63.79
LDEC020175-RA	cytochrome p450 9z4	-6.02	99.76	16.58
LDEC020702-RA	timeless isoform b	-6.02	86.12	14.31
LDEC011960-RA	histone-lysine n-methyltransferase 2d isoform x1	-6.01	93.40	15.54
LDEC001810-RA	zinc finger	-6.00	71.77	11.96
LDEC013100-RA	gem-associated protein 2	-5.97	65.78	11.02
LDEC019392-RA	vacuolar protein sorting-associated protein 13a-like isoform x1	-5.96	79.33	13.30
LDEC010124-RA	factor viii intron 22	-5.95	95.77	16.10
LDEC007802-RA	apoptosis 1 inhibitor	-5.94	1270.52	213.80
LDEC007081-RA	uba-like domain-containing protein 2	-5.91	265.79	44.94
LDEC012705-RA	casein kinase ii subunit alpha	-5.88	140.12	23.82
LDEC006232-RA	receptor-type tyrosine-protein phosphatase f-like	-5.84	94.68	16.21
LDEC019393-RA	vacuolar protein sorting-associated protein	-5.84	56.04	9.60
LDEC008075-RA	an1-type zinc finger protein 6 isoform x3	-5.83	1133.68	194.35
LDEC002306-RA	period isoform x1	-5.82	51.00	8.76
LDEC020890-RA	gonadal protein gdl	-5.82	57.56	9.89
LDEC008211-RA	hypothetical protein X975_24243, partial	-5.82	60.08	10.33
LDEC016322-RA	protein alpha isoform isoform x1	-5.81	47.43	8.16
LDEC014988-RA	cytosolic carboxypeptidase-like protein 5 isoform x1	-5.79	113.07	19.55
LDEC010376-RA	alanine--trna mitochondrial-like	-5.78	60.32	10.44
LDEC011811-RA	vacuolar protein sorting-associated protein 13b	-5.77	161.65	28.00
LDEC020409-RA	dentin sialophospho	-5.77	449.25	77.84
LDEC009513-RA	pleckstrin-like proteiny domain-containing family m member 2	-5.77	135.27	23.45
LDEC018465-RA	cathepsin b	-5.73	226.05	39.47
LDEC003502-RA	dopamine n-acetyltransferase-like isoform x4	-5.69	238.60	41.97
LDEC001823-RA	zinc finger swim domain-containing protein 7-like	-5.66	52.81	9.33
LDEC013255-RA	protein crebrf homolog	-5.63	103.47	18.38

LDEC000855-RA	fatty acid-binding protein	-5.63	592.98	105.36
LDEC007743-RA	a-kinase anchor protein mitochondrial	-5.62	103.85	18.47
LDEC018939-RA	target of rapamycin complex 2 subunit mapkap1	-5.60	55.85	9.96
LDEC021725-RA	juvenile hormone esterase	-5.57	140.12	25.16
LDEC006994-RA	calpain-b isoform x2	-5.56	69.11	12.44
LDEC009944-RA	cytochrome b-c1 complex subunit mitochondrial-like	-5.56	53.66	9.66
LDEC002745-RA	ras-interacting protein rip3-like isoform x1	-5.55	55.47	10.00
LDEC002706-RA	probable atp-dependent rna helicase dhx35	-5.54	375.86	67.80
LDEC006984-RA	glycine n-methyltransferase	-5.54	240.45	43.40
LDEC010443-RA	tetraspanin isoform a	-5.54	448.49	81.02
LDEC001454-RA	mediator of rna polymerase ii transcription subunit 22	-5.53	51.00	9.22
LDEC019376-RA	myosin light chain	-5.52	51.43	9.31
LDEC018052-RA	nuclear pore complex protein nup50	-5.52	102.00	18.47
LDEC016408-RA	sestrin homolog	-5.51	376.44	68.31
LDEC014438-RA	ac transposable element-derived protein partial	-5.51	88.17	16.00
LDEC014692-RA	mediator of rna polymerase ii transcription subunit 6	-5.50	68.30	12.42
LDEC011079-RA	upf0547 protein c16orf87-like	-5.50	74.38	13.53
LDEC010493-RA	serine protease s1a- partial	-5.49	271.68	49.46
LDEC022062-RA	protein fam107b isoform x2	-5.49	147.82	26.92
LDEC018540-RA	cd151 antigen-like	-5.47	141.07	25.81
LDEC018850-RA	maternal tudor protein	-5.44	61.36	11.29
LDEC008903-RA	zinc finger autosomal	-5.43	94.54	17.40
LDEC003335-RA	facilitated trehalose transporter tret1-like	-5.41	49.72	9.20
LDEC007979-RA	zinc phosphodiesterase	-5.37	102.57	19.09
LDEC004579-RA	serine threonine-protein kinase tricorner isoform x2	-5.37	317.93	59.17
LDEC009661-RA	myosin heavy chain kinase d	-5.37	61.46	11.44
LDEC005604-RA	cytochrome p450 6a2	-5.36	46.86	8.74
LDEC015913-RA	protein toll	-5.36	175.34	32.71
LDEC000981-RA	choline-phosphate cytidylyltransferase b-like	-5.36	70.87	13.22
LDEC005735-RA	calcium release-activated calcium channel protein 1-like	-5.35	144.30	26.96
LDEC012062-RA	cytochrome p450 6a2	-5.35	114.64	21.42
LDEC002596-RA	vacuolar protein sorting-associated protein 52 homolog	-5.35	110.08	20.58
LDEC014029-RA	---NA---	-5.33	111.74	20.96
LDEC006035-RA	serine threonine-protein kinase plk4	-5.31	85.74	16.15
LDEC009280-RA	---NA---	-5.30	110.36	20.83
LDEC023728-RA	tbc1 domain family member 10a	-5.29	110.74	20.93
LDEC011513-RA	4-coumarate-- ligase 1-like	-5.28	309.70	58.69
LDEC008684-RA	probable phosphoserine aminotransferase	-5.27	3562.40	676.25
LDEC017324-RA	flj37770-like protein	-5.27	152.19	28.90
LDEC000850-RA	fatty acid-biding protein	-5.26	253.10	48.12

LDEC008991-RA	ankyrin repeat and sterile alpha motif domain-containing protein 1b	-5.25	73.15	13.93
LDEC009779-RA	isoform a	-5.25	275.48	52.51
LDEC009532-RA	ecdysone-induced protein 74ef isoform a isoform x1	-5.24	53.04	10.12
LDEC001747-RA	eye-specific diacylglycerol kinase isoform x1	-5.23	64.69	12.36
LDEC008150-RA	kv channel-interacting protein 1	-5.23	76.10	14.54
LDEC021904-RA	transmembrane protein 127-like	-5.22	67.92	13.01
LDEC010524-RA	t-cell activation mitochondrial isoform x2	-5.22	49.10	9.41
LDEC023021-RA	protein henna	-5.20	116.88	22.46
LDEC022548-RA	protein decapentaplegic isoform x1	-5.17	53.80	10.41
LDEC000980-RA	dynactin subunit 5	-5.15	180.66	35.09
LDEC003698-RA	sex-lethal homolog isoform x5	-5.12	283.99	55.42
LDEC002207-RA	glycerol kinase 5	-5.11	57.18	11.19
LDEC012138-RA	PREDICTED: uncharacterized protein LOC655754 isoform X1	-5.10	83.89	16.44
LDEC015042-RA	glutathione s-transferase theta-1-like isoform x2	-5.07	71.96	14.20
LDEC010371-RA	vacuolar protein sorting-associated protein 33a	-5.06	82.61	16.33
LDEC002720-RA	zinc finger protein 271-like	-5.05	104.47	20.68
LDEC012541-RA	n-acetylneuraminate lyase-like	-5.05	83.32	16.50
LDEC018756-RA	aael009711- partial	-5.03	87.69	17.42
LDEC017762-RA	dna-directed rna polymerases and iii subunit rpabc5	-5.03	59.79	11.88
LDEC023277-RA	probable gpi-anchored adhesin-like protein pga55 isoform x3	-5.01	73.24	14.62
LDEC017254-RA	phosphoglucomutase-2	-4.98	55.66	11.17
LDEC007888-RA	vacuolar protein sorting-associated protein 53 homolog	-4.96	121.49	24.51
LDEC004771-RA	carboxyl choline esterase	-4.96	50.05	10.10
LDEC010669-RA	phosphatidate phosphatase lpin3	-4.92	85.03	17.27
LDEC002078-RA	dorsocross	-4.92	61.84	12.57
LDEC000857-RA	poly -binding protein 3 isoform x1	-4.92	341.31	69.41
LDEC000854-RA	fatty acid-binding	-4.91	323.63	65.90
LDEC004172-RA	cell cycle control protein 50a	-4.91	152.00	30.99
LDEC014985-RA	cytosolic carboxypeptidase-like protein 5 isoform x1	-4.88	58.03	11.88
LDEC015739-RA	arfaptin-2 isoform x1	-4.88	50.81	10.41
LDEC006677-RA	membrane magnesium transporter 1	-4.87	76.62	15.73
LDEC006934-RA	alpha beta hydrolase domain-containing protein 11	-4.85	212.93	43.86
LDEC004550-RA	protein btg1-like	-4.85	382.61	78.89
LDEC010340-RA	charged multivesicular body protein 7	-4.84	93.40	19.30
LDEC013301-RA	unconventional myosin-ixa isoform x1	-4.82	56.47	11.71
LDEC007993-RA	low quality protein: mucin-17	-4.82	125.19	25.98
LDEC018350-RA	PREDICTED: uncharacterized protein LOC658581	-4.81	111.69	23.21
LDEC000584-RA	restin homolog isoform x1	-4.81	172.68	35.89
LDEC005787-RA	muscle-specific protein 300	-4.81	81.80	17.02
LDEC010240-RA	ah receptor-interacting protein	-4.79	78.90	16.46

LDEC005816-RA	serine threonine-protein phosphatase 4 regulatory subunit 3 isoform x1	-4.78	90.21	18.88
LDEC021088-RA	cd63 antigen-like	-4.77	108.61	22.78
LDEC019024-RA	zinc finger protein 271-like	-4.75	60.13	12.65
LDEC007613-RA	transposable element tc3 transposase	-4.73	344.73	72.84
LDEC002679-RA	upf0364 protein c6orf211 homolog	-4.73	374.77	79.26
LDEC019099-RA	activating signal cointegrator 1 complex subunit 1	-4.73	70.20	14.85
LDEC010384-RA	mediator of rna polymerase ii transcription subunit 15-like isoform x4	-4.72	103.09	21.86
LDEC009572-RA	adp-ribosylation factor-like protein 6-interacting protein 4	-4.70	107.08	22.77
LDEC005290-RA	transcription factor	-4.68	62.93	13.43
LDEC023612-RA	laccase 2	-4.67	53.61	11.48
LDEC010765-RA	neural wiskott-aldrich syndrome protein	-4.67	60.22	12.90
LDEC002122-RA	ras-related protein rab-2a	-4.65	169.92	36.50
LDEC006859-RA	ubiquitin thioesterase otu1	-4.64	172.58	37.19
LDEC010432-RA	phospholipase d3-like isoform x2	-4.63	78.23	16.88
LDEC018560-RA	polynucleotide 5 -hydroxyl-kinase nol9	-4.62	113.36	24.55
LDEC019036-RA	nose resistant to fluoxetine protein 6	-4.61	58.79	12.74
LDEC015435-RA	ca(2+) calmodulin-responsive adenylate cyclase isoform x7	-4.61	61.55	13.36
LDEC013735-RA	dna-binding protein ets97d-like	-4.61	62.83	13.64
LDEC007664-RA	PREDICTED: uncharacterized protein LOC657045	-4.60	443.64	96.50
LDEC017188-RA	---NA---	-4.59	269.45	58.71
LDEC001898-RA	serine threonine-protein kinase wnk1-like isoform x1	-4.59	688.47	150.08
LDEC011670-RA	succinate dehydrogenase assembly factor mitochondrial-like	-4.59	82.18	17.92
LDEC009197-RA	lipopolysaccharide-induced tumor necrosis factor-alpha factor homolog	-4.59	77.33	16.86
LDEC006166-RA	bis(5 -nucleosyl)-tetraphosphatase	-4.57	151.43	33.13
LDEC005343-RA	xanthine dehydrogenase	-4.57	135.65	29.70
LDEC007576-RA	e3 ubiquitin-protein ligase ubr4 isoform x2	-4.56	109.41	23.99
LDEC002545-RA	proactivator polypeptide	-4.56	239.36	52.54
LDEC008335-RA	venom dipeptidyl peptidase 4 isoform x1	-4.55	244.40	53.66
LDEC010970-RA	max-binding protein mnt	-4.55	179.42	39.44
LDEC017559-RA	esterase	-4.54	206.52	45.45
LDEC005771-RA	twinfilin isoform x1	-4.54	73.10	16.10
LDEC008105-RA	protein painting of fourth isoform x1	-4.54	98.53	21.71
LDEC001392-RA	conserved oligomeric golgi complex subunit 8	-4.54	56.09	12.36
LDEC022799-RA	reverse partial	-4.53	154.04	33.98
LDEC004370-RA	testis-specific serine threonine-protein kinase 1-like	-4.53	98.67	21.77
LDEC016642-RA	fanconi anemia group m protein homolog	-4.53	57.46	12.69
LDEC024339-RA	protein ssxt isoform x1	-4.53	155.52	34.34
LDEC001101-RA	agap008807-pa-like protein	-4.53	126.52	27.94
LDEC006753-RA	zinc finger protein 271-like	-4.53	108.27	23.91
LDEC005367-RA	grip and coiled-coil domain-containing	-4.53	187.36	41.39

	protein 2-like			
LDEC010491-RA	serine protease s1a- partial	-4.52	222.72	49.25
LDEC006116-RA	PREDICTED: uncharacterized protein C6orf106 homolog isoform X1	-4.51	70.20	15.56
LDEC007972-RA	titin isoform x1	-4.50	535.23	118.96
LDEC013507-RA	furin-like protease 2	-4.48	55.85	12.46
LDEC002695-RA	protein ariadne-1	-4.48	82.84	18.51
LDEC009121-RA	relish	-4.47	54.09	12.09
LDEC004273-RA	tripartite motif-containing protein 2 isoform x3	-4.45	67.92	15.25
LDEC005623-RA	isoform b	-4.45	86.84	19.51
LDEC012820-RA	unknown	-4.45	269.49	60.61
LDEC007934-RA	mob kinase activator-like 3	-4.44	53.00	11.94
LDEC004820-RA	rna-binding protein nova-1 isoform x2	-4.44	714.42	160.97
LDEC021181-RA	hairy enhancer-of-split related with yrpw motif-like protein	-4.43	80.61	18.19
LDEC003737-RA	probable helicase with zinc finger domain	-4.43	64.02	14.45
LDEC014513-RA	nuclear hormone receptor ftz-	-4.42	81.18	18.36
LDEC005755-RA	diapause-associated transcript-2	-4.42	151.57	34.28
LDEC007932-RA	zinc finger protein 729-like isoform x1	-4.42	118.49	26.81
LDEC010179-RA	kazal-type proteinase inhibitor-like protein	-4.42	202.76	45.88
LDEC007102-RA	leucine-rich repeat-containing protein 15-like	-4.41	56.99	12.92
LDEC014321-RA	isoform a	-4.40	245.49	55.74
LDEC007904-RA	protein dopey-1 homolog	-4.40	91.02	20.70
LDEC020994-RA	nucleotide exchange factor sil1	-4.39	82.89	18.88
LDEC005973-RA	exosome component 10	-4.39	188.03	42.87
LDEC002249-RA	dna excision repair protein ercc-6-like	-4.38	132.47	30.26
LDEC001063-RA	protein unc-79 homolog	-4.35	147.63	33.92
LDEC020111-RA	rna exonuclease nef-sp	-4.34	185.75	42.83
LDEC020670-RA	phosphoenolpyruvate carboxykinase	-4.33	528.96	122.26
LDEC015081-RA	ornithine mitochondrial	-4.32	86.03	19.93
LDEC007908-RA	cdk5 regulatory subunit-associated protein 3	-4.31	231.04	53.56
LDEC007835-RA	guanine nucleotide-binding 1	-4.30	67.06	15.60
LDEC000144-RA	dynein light	-4.30	146.72	34.13
LDEC004614-RA	proton-coupled amino acid transporter 4-like	-4.30	104.95	24.41
LDEC012391-RA	transient receptor potential cation channel trpm	-4.29	55.04	12.84
LDEC006953-RA	ubiquitin conjugation factor e4 b	-4.29	102.09	23.82
LDEC007554-RA	hypothetical protein YQE_11513, partial	-4.28	165.93	38.73
LDEC010651-RA	optic atrophy 3 protein homolog	-4.28	70.72	16.52
LDEC023016-RA	u6 snrna-associated sm-like protein lsm8	-4.28	58.08	13.57
LDEC002171-RA	afadin isoform x2	-4.28	92.59	21.63
LDEC009063-RA	zinc finger	-4.28	97.25	22.75
LDEC019976-RA	cell division cycle protein 123 homolog	-4.27	86.31	20.20

LDEC013344-RA	ccr4-not transcription complex subunit 4	-4.26	294.07	69.00
LDEC003664-RA	fasciclin-2 isoform x3	-4.24	55.51	13.09
LDEC000074-RA	zinc finger fyve domain-containing protein 1-like	-4.24	66.30	15.64
LDEC001620-RA	sv2-like protein 1	-4.24	153.00	36.12
LDEC000677-RA	nuclear rna export factor 2-like	-4.23	75.19	17.76
LDEC014400-RA	cuticular protein 92f	-4.23	667.98	157.90
LDEC016618-RA	protein sde2 homolog	-4.22	89.36	21.19
LDEC016864-RA	pleckstrin homology-like domain family b member 1	-4.22	58.89	13.97
LDEC008540-RA	phd finger and cxxc domain-containing protein cg17446-like	-4.21	54.99	13.07
LDEC014818-RA	huntingtin interacting protein	-4.21	155.37	36.93
LDEC010226-RA	phytanoyl- dioxygenase domain-containing protein 1 homolog	-4.20	107.46	25.56
LDEC016448-RA	PREDICTED: uncharacterized protein LOC103312904	-4.20	65.73	15.64
LDEC008252-RA	trna pseudouridine synthase pus10	-4.20	55.70	13.26
LDEC003342-RA	nucleolin-like isoform x2	-4.19	93.97	22.40
LDEC005666-RA	colorectal mutant cancer protein isoform x2	-4.19	94.25	22.50
LDEC009472-RA	low quality protein: huntingtin-like	-4.19	130.85	31.25
LDEC021177-RA	PREDICTED: uncharacterized protein LOC105252462	-4.18	224.63	53.67
LDEC006709-RA	5 -3 exoribonuclease 1-like isoform x1	-4.18	103.28	24.68
LDEC016650-RA	probable nucleolar gtp-binding protein 1	-4.18	433.85	103.86
LDEC014925-RA	polycomb group protein psc-like	-4.16	158.89	38.21
LDEC017722-RA	polyadenylate-binding protein-interacting protein 1	-4.15	108.04	26.04
LDEC002000-RA	sulfotransferase 1c4-like	-4.14	103.23	24.91
LDEC005505-RA	agap004519-pa-like protein	-4.13	136.17	32.94
LDEC003701-RA	retinal protein	-4.13	123.01	29.80
LDEC007437-RA	cklf-like marvel transmembrane domain-containing protein 4-like	-4.12	140.26	34.05
LDEC003495-RA	tubulin-specific chaperone cofactor e-like protein	-4.12	242.45	58.89
LDEC017508-RA	zinc finger protein 264-like isoform x1	-4.11	179.28	43.65
LDEC007263-RA	cell differentiation protein rcd1 homolog	-4.10	160.22	39.05
LDEC002669-RA	tyrosine-protein phosphatase non-receptor type 1 isoform x1	-4.10	98.81	24.09
LDEC008408-RA	glycoside hydrolase family 1	-4.10	103.19	25.16
LDEC015714-RA	det1- and ddb1-associated protein 1-like	-4.08	68.68	16.82
LDEC016130-RA	hypothetical protein D910_10956	-4.07	214.31	52.62
LDEC014539-RA	zinc finger protein 106-like isoform x1	-4.07	120.96	29.72
LDEC002109-RA	upf0183 protein cg7083 isoform x1	-4.07	62.36	15.33
LDEC019073-RA	sporulation-specific protein 15	-4.06	159.37	39.21
LDEC013706-RA	low quality protein: membralin	-4.06	120.58	29.68
LDEC003481-RA	protein d2-like isoform x2	-4.06	59.32	14.60
LDEC009713-RA	fh1 fh2 domain-containing protein 3 isoform x4	-4.06	61.84	15.23
LDEC016648-RA	rna-binding protein pno1	-4.06	198.58	48.96
LDEC010296-RA	peptidyl-prolyl cis-trans isomerase g-like	-4.05	76.67	18.93

LDEC011365-RA	hypothetical protein YQE_09758, partial	-4.05	57.99	14.33
LDEC009118-RA	nuclear factor nf-kappa-b p110 subunit isoform x2	-4.04	116.40	28.82
LDEC010725-RA	15-hydroxyprostaglandin dehydrogenase	-4.03	220.68	54.75
LDEC013897-RA	general transcription factor iie subunit 2-like	-4.03	66.54	16.52
LDEC007992-RA	low quality protein: mucin-17	-4.02	148.72	36.96
LDEC013725-RA	and pleckstrin domain-containing protein 2-like	-4.01	96.30	23.99
LDEC017391-RA	cullin-2	-4.01	300.20	74.87
LDEC000519-RA	ubiquitin carboxyl-terminal hydrolase 2-like isoform x2	-4.01	159.70	39.86
LDEC021825-RA	---NA---	-4.00	131.13	32.77
LDEC003007-RA	isoform b	-4.00	1074.84	268.76
LDEC017926-RA	protein winged eye isoform x1	-4.00	110.79	27.73
LDEC001174-RA	broad- partial	-3.99	76.76	19.24
LDEC005613-RA	PREDICTED: uncharacterized protein LOC100141684	-3.99	70.87	17.76
LDEC018513-RA	protein fam134c-like isoform x1	-3.99	113.36	28.44
LDEC020999-RA	PREDICTED: uncharacterized protein LOC662064 isoform X3	-3.99	256.00	64.23
LDEC013830-RA	mediator of rna polymerase ii transcription subunit 29	-3.98	87.45	21.96
LDEC003202-RA	wash complex subunit fam21a	-3.98	62.93	15.81
LDEC007777-RA	nuclear export mediator factor nemf homolog	-3.98	162.46	40.82
LDEC021977-RA	patched domain-containing protein 3	-3.98	93.73	23.55
LDEC001814-RA	synaptosomal-associated protein 29	-3.96	70.63	17.82
LDEC014856-RA	---NA---	-3.96	385.47	97.27
LDEC002381-RA	PREDICTED: uncharacterized protein LOC664340	-3.96	90.97	23.00
LDEC016503-RA	mitochondrial import inner membrane translocase subunit tim17-b-like	-3.95	276.53	69.92
LDEC000053-RA	tubulin-specific chaperone c	-3.95	421.97	106.72
LDEC001831-RA	zinc finger protein 845-like	-3.95	92.30	23.36
LDEC008516-RA	ufm1-specific protease 2	-3.95	119.92	30.37
LDEC019310-RA	yorkie homolog	-3.94	170.16	43.21
LDEC006463-RA	protein goliath-like	-3.94	85.17	21.63
LDEC020300-RA	peptidyl-prolyl cis-trans isomerase fkbp8	-3.93	130.75	33.27
LDEC007982-RA	nucleolar complex protein 2 homolog	-3.93	234.84	59.77
LDEC000177-RA	protein arginine n-methyltransferase 9 isoform x1	-3.93	60.55	15.43
LDEC007662-RA	3-ketodihydrosphingosine reductase	-3.92	94.87	24.22
LDEC019455-RA	sorting and assembly machinery component 50 homolog	-3.92	102.81	26.25
LDEC009473-RA	huntingtin isoform x2	-3.90	66.35	17.00
LDEC003896-RA	peroxidasin homolog isoform x1	-3.90	100.24	25.72
LDEC008000-RA	PREDICTED: uncharacterized protein KIAA1109	-3.89	90.83	23.34
LDEC021174-RA	low-density lipoprotein receptor-related protein 6	-3.88	112.46	28.99
LDEC006105-RA	mitochondrial isoform x3	-3.87	70.20	18.13
LDEC000269-RA	spatacsin isoform x1	-3.87	66.73	17.25

LDEC004560-RA	persulfide dioxygenase mitochondrial	-3.87	217.97	56.34
LDEC009685-RA	bag domain-containing protein samui isoform x1	-3.86	442.36	114.48
LDEC019705-RA	mitochondrial-processing peptidase subunit alpha	-3.85	80.71	20.96
LDEC003908-RA	cap-gly domain-containing linker protein 1	-3.84	223.01	58.04
LDEC005977-RA	dcn1-like protein 1	-3.84	75.81	19.74
LDEC015308-RA	gdp-l-fucose synthase	-3.83	110.36	28.78
LDEC003562-RA	kat8 regulatory nsl complex subunit 3 isoform x2	-3.83	74.00	19.32
LDEC015706-RA	fat-like cadherin-related tumor suppressor homolog	-3.83	183.61	47.93
LDEC010804-RA	proteoglycan 4-like	-3.83	135.03	35.28
LDEC013396-RA	tetratricopeptide repeat domain 21b	-3.83	101.52	26.54
LDEC010341-RA	eukaryotic peptide chain release factor subunit 1 isoform x1	-3.81	368.93	96.89
LDEC005491-RA	ribose-phosphate pyrophosphokinase 1 isoform x2	-3.80	706.53	186.11
LDEC007228-RA	gastrula zinc finger protein	-3.79	93.40	24.62
LDEC004369-RA	liprin-beta-1 isoform x1	-3.79	254.62	67.22
LDEC003266-RA	proteoglycan 4-like isoform x1	-3.79	954.30	251.99
LDEC013741-RA	epithelial membrane protein	-3.79	84.75	22.38
LDEC004673-RA	major facilitator superfamily domain-containing protein 6-like	-3.78	111.98	29.63
LDEC010796-RA	tbc1 domain family member 14	-3.78	102.38	27.10
LDEC010492-RA	serine proteinase	-3.76	226.24	60.09
LDEC013070-RA	syntaxin-binding protein 5 isoform x3	-3.76	172.39	45.80
LDEC005585-RA	e3 ubiquitin-protein ligase herc2	-3.76	63.31	16.82
LDEC007674-RA	blastoderm-specific protein 25d	-3.76	120.54	32.04
LDEC007658-RA	probable gpi-anchored adhesin-like protein pga55	-3.75	76.29	20.37
LDEC008387-RA	homolog of	-3.74	334.04	89.20
LDEC000091-RA	hormone-sensitive lipase	-3.74	123.34	32.94
LDEC002585-RA	gtp-binding nuclear protein	-3.74	140.02	37.41
LDEC011693-RA	tubulin polyglutamylase ttll4-like	-3.74	184.13	49.21
LDEC003886-RA	PREDICTED: uncharacterized protein LOC103313983	-3.74	119.68	32.04
LDEC012850-RA	metaxin-1-like isoform x1	-3.73	76.86	20.60
LDEC007858-RA	peroxisome assembly protein 12	-3.73	82.56	22.15
LDEC013940-RA	PREDICTED: uncharacterized protein LOC103314045	-3.72	65.21	17.53
LDEC002192-RA	trna (guanine -n1)-methyltransferase	-3.72	64.17	17.27
LDEC017501-RA	tryptophan oxygenase	-3.71	177.29	47.77
LDEC003145-RA	androgen-induced protein 1	-3.71	97.34	26.23
LDEC000987-RA	bromo adjacent homology domain containing	-3.71	325.58	87.76
LDEC002118-RA	microtubule-associated protein futsch-like isoform x1	-3.71	125.76	33.92
LDEC000530-RA	caspase nc-like	-3.71	115.69	31.22
LDEC009122-RA	relish	-3.70	92.07	24.85
LDEC005802-RA	protein ltv1 homolog	-3.69	280.90	76.04
LDEC001213-RA	bsd domain-containing protein 1	-3.69	81.13	22.00

LDEC015930-RA	protein gdap2 homolog	-3.68	355.00	96.35
LDEC018936-RA	isoform b	-3.68	130.33	35.41
LDEC000992-RA	estradiol 17-beta-dehydrogenase 2	-3.68	80.56	21.90
LDEC005817-RA	serine threonine-protein phosphatase 4 regulatory subunit 3	-3.67	71.86	19.56
LDEC012321-RA	ran-binding protein 9-like	-3.66	106.32	29.01
LDEC007385-RA	nedd4-binding protein 2	-3.66	607.76	165.87
LDEC007273-RA	maguk p55 subfamily member 2	-3.66	185.56	50.65
LDEC016747-RA	hypothetical protein YQE_01873, partial	-3.66	157.80	43.12
LDEC011152-RA	cytochrome p450 18a1	-3.66	71.44	19.53
LDEC015375-RA	ras-related protein rab-1a	-3.65	118.82	32.56
LDEC019790-RA	pyrroline-5-carboxylate reductase 3	-3.63	77.66	21.37
LDEC009952-RA	suppression of tumorigenicity 1	-3.62	776.02	214.08
LDEC017890-RA	isocitrate dehydrogenase	-3.61	412.65	114.15
LDEC015295-RA	sarcoplasmic calcium-binding protein	-3.60	193.59	53.73
LDEC009629-RA	a-kinase anchor protein 17a	-3.60	66.49	18.47
LDEC011699-RA	vacuolar protein sorting-associated protein 11 homolog	-3.60	83.94	23.34
LDEC016364-RA	protein ddi1 homolog 2 isoform x1	-3.59	270.40	75.23
LDEC021319-RA	cytochrome c oxidase copper chaperone	-3.59	68.44	19.05
LDEC006935-RA	integral membrane protein gpr155	-3.59	107.32	29.87
LDEC012781-RA	osteopetrosis-associated transmembrane protein 1	-3.59	88.31	24.60
LDEC008023-RA	hermansky-pudlak syndrome 3 protein	-3.59	63.17	17.61
LDEC004693-RA	phospholipid scramblase 1-like isoform x1	-3.58	129.19	36.06
LDEC023469-RA	homogentisate -dioxygenase	-3.58	209.89	58.69
LDEC013325-RA	mitochondrial import inner membrane translocase subunit tim14	-3.57	67.21	18.82
LDEC014939-RA	telomere-associated protein rif1-like	-3.56	76.38	21.44
LDEC012691-RA	ddb1- and cul4-associated factor 6-partial	-3.56	73.39	20.62
LDEC022787-RA	poly -specific ribonuclease parn	-3.56	98.81	27.79
LDEC006735-RA	PREDICTED: uncharacterized protein LOC103313913 isoform X1	-3.56	75.10	21.12
LDEC014565-RA	nucleolar protein 14 homolog	-3.56	85.79	24.13
LDEC009402-RA	armadillo repeat-containing protein 5-like	-3.54	79.04	22.31
LDEC011419-RA	dnaj homolog subfamily c member 7	-3.54	944.56	267.15
LDEC006157-RA	nucleolar complex protein 3 homolog	-3.53	352.29	99.66
LDEC001429-RA	protein beta isoform-like isoform x32	-3.53	560.28	158.53
LDEC007823-RA	PREDICTED: uncharacterized protein LOC103312333 isoform X2	-3.53	282.33	79.89
LDEC008001-RA	PREDICTED: uncharacterized protein KIAA1109	-3.53	99.53	28.17
LDEC005083-RA	probable 39s ribosomal protein mitochondrial	-3.53	122.91	34.80
LDEC006020-RA	cytochrome p450 partial	-3.53	188.60	53.41
LDEC001064-RA	ankyrin unc44	-3.53	133.99	37.96
LDEC000747-RA	unknown	-3.52	78.99	22.44
LDEC002200-RA	calponin homology domain-containing protein ddb_g0272472-like	-3.51	85.27	24.32

LDEC003868-RA	nicotinamide mononucleotide adenylyltransferase 1-like	-3.50	74.76	21.37
LDEC012531-RA	general transcription factor 3c polypeptide	-3.49	98.72	28.28
LDEC007905-RA	protein dopey-1 homolog	-3.49	70.34	20.16
LDEC007820-RA	rna-directed dna polymerase from mobile element jockey-like	-3.48	135.36	38.88
LDEC006954-RA	ubiquitin conjugation factor e4 b	-3.48	83.32	23.93
LDEC021430-RA	serine proteinase	-3.48	323.44	92.92
LDEC004876-RA	cuticular protein analogous to peritrophins 3-d1	-3.48	120.06	34.51
LDEC019651-RA	golgin subfamily a member 4-like	-3.46	173.06	50.01
LDEC004144-RA	4-hydroxyphenylpyruvate dioxygenase	-3.46	1467.62	424.22
LDEC008584-RA	protein zer-1 homolog	-3.45	101.67	29.45
LDEC006673-RA	lian-aa1-like retrotransposon protein	-3.45	128.28	37.23
LDEC018934-RA	juvenile hormone esterase isoform a	-3.44	142.40	41.35
LDEC019707-RA	anaphase-promoting complex subunit 5-like	-3.44	72.34	21.04
LDEC000948-RA	hmg box transcription factor bbx	-3.44	109.70	31.92
LDEC015374-RA	ras-related protein rab-1a	-3.44	925.59	269.43
LDEC009800-RA	tumor susceptibility gene 101 protein	-3.43	66.45	19.39
LDEC000157-RA	---NA---	-3.42	193.73	56.61
LDEC010238-RA	casein kinase i isoform alpha isoform x1	-3.42	307.85	90.06
LDEC001065-RA	transcription initiation factor iia subunit 1	-3.41	90.54	26.52
LDEC011140-RA	phosphoglucomutase-2	-3.40	215.12	63.18
LDEC003776-RA	protein szt2-like isoform x1	-3.40	153.81	45.20
LDEC009226-RA	mucin-22 isoform x1	-3.40	116.16	34.15
LDEC019359-RA	fasciculation and elongation protein zeta-2	-3.40	183.13	53.85
LDEC024050-RA	ras-related protein rab-7a	-3.40	177.52	52.20
LDEC011352-RA	josephin-like isoform x2	-3.39	89.97	26.52
LDEC000704-RA	antifreeze protein maxi	-3.38	170.58	50.44
LDEC014503-RA	calcium uptake protein 1 mitochondrial isoform x2	-3.38	205.09	60.73
LDEC007736-RA	ubiquitin-conjugating enzyme e2-24 kda	-3.38	276.34	81.84
LDEC002736-RA	zinc finger protein 271-like	-3.37	374.63	111.07
LDEC021658-RA	tetratricopeptide repeat protein 19 mitochondrial	-3.37	101.67	30.16
LDEC000637-RA	mitochondrial nadh-ubiquinone oxidoreductase 9 kda subunit-like protein	-3.37	145.68	43.27
LDEC014762-RA	probable phospholipid-transporting atpase ia isoform x4	-3.37	69.20	20.56
LDEC007728-RA	pleiotrophic factor-alpha-1	-3.36	145.54	43.31
LDEC011355-RA	tetratricopeptide repeat protein 17-like isoform x1	-3.35	189.88	56.63
LDEC011549-RA	hypothetical protein TcasGA2_TC003147	-3.35	562.75	168.00
LDEC016756-RA	glycolipid transfer protein	-3.35	112.50	33.61
LDEC003977-RA	homeobox protein nk-	-3.34	137.69	41.18
LDEC005739-RA	zinc finger cchc domain-containing protein 8 homolog	-3.34	122.34	36.60
LDEC014668-RA	creb-regulated transcription coactivator 1-like isoform x1	-3.34	181.28	54.25

LDEC007223-RA	thap domain-containing protein partial	-3.34	294.92	88.30
LDEC012135-RA	polyhomeotic-like protein 1	-3.34	69.68	20.87
LDEC003093-RA	transmembrane and coiled-coil domain-containing protein 7-like isoform 1	-3.34	73.48	22.02
LDEC005932-RA	dna polymerase eta	-3.33	117.11	35.12
LDEC004830-RA	xk-related protein 7-like	-3.33	159.65	47.89
LDEC017229-RA	patatin-like phospholipase domain-containing protein 2-like	-3.32	189.36	56.97
LDEC002385-RA	transmembrane protein 131 isoform x1	-3.32	165.26	49.73
LDEC016579-RA	zinc finger ccch domain-containing protein 13	-3.32	397.78	119.71
LDEC002170-RA	zinc finger mym-type protein 1	-3.32	72.20	21.73
LDEC002333-RA	s phase cyclin a-associated protein in the endoplasmic reticulum	-3.32	84.65	25.52
LDEC005912-RA	heparan-alpha-glucosaminide n-acetyltransferase-like isoform x1	-3.32	154.19	46.51
LDEC004536-RA	e3 ubiquitin ligase	-3.31	123.72	37.33
LDEC011060-RA	peptidyl-prolyl cis-trans mitochondrial	-3.31	269.78	81.52
LDEC002288-RA	dehydrogenase reductase sdr family member 11-like	-3.30	201.86	61.15
LDEC003301-RA	muscle m-line assembly protein unc-89-like	-3.30	384.85	116.62
LDEC002523-RA	transposable element tc3 transposase	-3.30	85.51	25.95
LDEC003704-RA	swi snf-related matrix-associated actin-dependent regulator of chromatin subfamily b member 1	-3.30	107.42	32.60
LDEC003376-RA	protein henna	-3.28	157.66	48.02
LDEC012288-RA	death-associated protein kinase related	-3.28	163.12	49.69
LDEC010612-RA	probable rna-binding protein 19-like isoform x2	-3.28	81.23	24.80
LDEC008659-RA	trafficking protein particle complex subunit 4	-3.27	96.25	29.43
LDEC013823-RA	hypothetical protein D910_11256	-3.26	163.07	49.96
LDEC011950-RA	cyclin-g2 isoform x1	-3.26	572.88	175.89
LDEC007878-RA	agap009935-pa-like protein	-3.25	117.40	36.14
LDEC005426-RA	phosphatidylinositol-binding clathrin assembly protein lap-like isoform x2	-3.25	402.29	123.92
LDEC002747-RA	---NA---	-3.24	82.70	25.49
LDEC018500-RA	selenoprotein s b-like	-3.24	277.38	85.62
LDEC017044-RA	PREDICTED: uncharacterized protein LOC659539	-3.24	347.92	107.41
LDEC020680-RA	dnaj homolog subfamily c member 2	-3.24	326.34	100.76
LDEC007464-RA	rho gtpase-activating protein 18 isoform x2	-3.23	77.47	23.95
LDEC008291-RA	acidic fibroblast growth factor intracellular-binding protein	-3.22	284.09	88.19
LDEC007667-RA	protein flightless-1	-3.22	149.72	46.53
LDEC019792-RA	pre-mrna-splicing factor 18-like	-3.21	74.38	23.19
LDEC012241-RA	endothelin-converting enzyme 1 isoform x2	-3.21	125.15	39.03
LDEC016723-RA	protein ctla-2-alpha	-3.20	408.61	127.64
LDEC011141-RA	pctp-like protein	-3.20	91.73	28.67
LDEC005098-RA	oxidoreductase glyr1 homolog	-3.20	329.57	103.00
LDEC019652-RA	hypothetical protein D910_08010	-3.20	136.17	42.56
LDEC013896-RA	acyl- synthetase family member	-3.20	160.27	50.13

	mitochondrial			
LDEC015926-RA	n-alpha-acetyltransferase 20	-3.19	113.93	35.72
LDEC016934-RA	nose resistant to fluoxetine protein 6-like	-3.18	147.86	46.43
LDEC020774-RA	ap-2 complex subunit sigma	-3.18	106.51	33.52
LDEC012596-RA	xylulose kinase	-3.18	81.66	25.72
LDEC011381-RA	neutral alpha-glucosidase ab	-3.17	477.82	150.60
LDEC021220-RA	probable splicing factor 3b subunit 5	-3.16	100.10	31.64
LDEC020706-RA	septin-1 isoform x1	-3.16	82.08	25.95
LDEC010670-RA	phosphatidate phosphatase lpin3	-3.16	307.71	97.27
LDEC004762-RA	alpha-tocopherol transfer	-3.16	102.95	32.60
LDEC013749-RA	scavenger receptor protein	-3.15	380.76	120.82
LDEC011750-RA	two pore calcium channel protein 1-like	-3.15	178.24	56.59
LDEC003804-RA	atp-binding cassette sub-family g member 1 isoform x2	-3.15	138.26	43.94
LDEC005886-RA	agap010471-pa-like protein	-3.14	130.52	41.51
LDEC014455-RA	2-hydroxyacylsphingosine 1-beta-galactosyltransferase-like	-3.14	88.26	28.09
LDEC012008-RA	zinc finger mym-type protein 1-like	-3.14	124.53	39.65
LDEC017502-RA	tryptophan oxygenase	-3.13	113.60	36.26
LDEC012722-RA	forkhead box protein n3-like isoform x1	-3.13	106.61	34.05
LDEC004616-RA	amino acid transporter	-3.13	132.85	42.45
LDEC015023-RA	tyrosine-protein phosphatase corkscrew isoform x2	-3.13	145.11	46.43
LDEC020227-RA	mediator of rna polymerase ii transcription subunit 1	-3.12	114.78	36.79
LDEC016174-RA	phosphatidylcholine:ceramide cholinephosphotransferase 2 isoform x2	-3.11	298.68	95.95
LDEC003853-RA	abc transporter g family member 20 isoform x2	-3.11	82.56	26.54
LDEC007277-RA	protein kinase dc2	-3.11	137.98	44.38
LDEC021826-RA	---NA---	-3.11	220.02	70.81
LDEC003253-RA	glucose-induced degradation protein 8 homolog	-3.10	99.72	32.12
LDEC003340-RA	ubiquitin carboxyl-terminal hydrolase 47 isoform x1	-3.10	238.36	76.80
LDEC010458-RA	low-density lipoprotein receptor class a domain-containing protein 3	-3.10	104.99	33.84
LDEC020446-RA	lysine-specific demethylase 4b-like isoform x1	-3.10	272.06	87.75
LDEC003894-RA	ubiquitin thioesterase trabid	-3.10	135.22	43.61
LDEC012240-RA	fatty acyl- reductase cg5065-like	-3.10	127.43	41.10
LDEC000942-RA	serine threonine-protein kinase rio1-like	-3.10	114.36	36.93
LDEC000805-RA	cell division cycle protein 16 homolog	-3.09	78.33	25.31
LDEC004548-RA	PREDICTED: uncharacterized protein LOC661444	-3.09	173.48	56.22
LDEC010954-RA	intracellular protein transport protein uso1-like isoform x1	-3.08	279.57	90.87
LDEC002993-RA	protein hid1-like isoform x2	-3.08	82.32	26.77
LDEC001258-RA	pyruvate dehydrogenase (acetyl-transferring) mitochondrial	-3.07	97.48	31.73
LDEC018269-RA	coenzyme q-binding protein coq10 homolog mitochondrial	-3.07	106.89	34.82
LDEC015022-RA	isoform a	-3.06	109.56	35.78

LDEC015541-RA	inositol oxygenase	-3.06	185.75	60.71
LDEC016266-RA	annexin b9-like isoform x1	-3.06	144.25	47.16
LDEC018833-RA	hypothetical protein TcasGA2_TC007902	-3.05	94.25	30.91
LDEC005924-RA	unhealthy ribosome biogenesis protein 2-like protein	-3.05	122.25	40.15
LDEC005960-RA	cgmp-dependent 3 -cyclic phosphodiesterase-like	-3.03	121.82	40.15
LDEC008308-RA	t-complex protein 11-like protein 1	-3.03	93.54	30.83
LDEC015677-RA	nadh dehydrogenase subunit 2	-3.03	179.47	59.21
LDEC011221-RA	centrosomal protein of 290 kda-like isoform x1	-3.03	83.65	27.63
LDEC015047-RA	multiple inositol polyphosphate phosphatase 1	-3.02	217.07	71.84
LDEC002685-RA	atp-binding cassette sub-family e member 1	-3.02	602.25	199.67
LDEC013033-RA	brain-enriched guanylate kinase-associated protein	-3.01	92.45	30.68
LDEC003834-RA	tissue inhibitor of metalloproteases	-3.01	163.17	54.17
LDEC005785-RA	muscle-specific protein 300	-3.01	1474.32	490.01
LDEC001484-RA	protein disulfide-isomerase txndc10	-3.00	108.23	36.06
LDEC018182-RA	neutral and basic amino acid transport protein rbat-like isoform x2	-2.99	161.55	54.00
LDEC011071-RA	exonuclease gor-like protein	-2.99	128.57	42.98
LDEC009542-RA	transient receptor potential cation channel protein painless	-2.99	114.40	38.29
LDEC013772-RA	probable elongation factor 1-delta isoform x3	-2.99	979.54	327.91
LDEC011548-RA	vacuolar fusion protein ccz1 homolog	-2.98	216.31	72.61
LDEC017470-RA	protein dennd6a isoform x1	-2.98	89.21	29.95
LDEC009311-RA	set and mynd domain-containing protein 4-like	-2.97	205.33	69.10
LDEC005482-RA	autophagy-related protein 13 homolog isoform x1	-2.97	191.73	64.58
LDEC009543-RA	conserved oligomeric golgi complex subunit 3	-2.97	121.49	40.93
LDEC011239-RA	pol-like protein	-2.96	82.75	27.92
LDEC004182-RA	short gastrulation precursor	-2.96	103.80	35.03
LDEC003406-RA	protein wings apart-like isoform x1	-2.96	127.47	43.04
LDEC006034-RA	max-like protein x	-2.96	112.84	38.11
LDEC013826-RA	ccaat enhancer-binding protein zeta	-2.94	113.26	38.57
LDEC014031-RA	poly -binding-splicing factor half pint isoform x4	-2.93	164.22	56.03
LDEC003722-RA	prostaglandin reductase 1-like	-2.93	85.79	29.28
LDEC019041-RA	inositol polyphosphate 5-phosphatase ocrl-1	-2.93	86.41	29.51
LDEC015586-RA	selenoprotein m-like	-2.93	87.74	29.99
LDEC001862-RA	ras-related protein rab-7a	-2.92	108.56	37.12
LDEC016248-RA	tetraspanins-like protein-8	-2.92	534.66	182.89
LDEC017602-RA	protein phosphatase 1 regulatory subunit 7	-2.92	79.99	27.40
LDEC015830-RA	poly polymerase type 3	-2.91	115.59	39.67
LDEC004694-RA	kinesin-like protein kif13a	-2.91	232.23	79.81
LDEC010788-RA	peptide methionine sulfoxide reductase	-2.91	386.56	132.95
LDEC018559-RA	methyltransferase-like protein 6	-2.91	88.55	30.47

LDEC014508-RA	dehydrogenase reductase sdr family protein 7-like	-2.90	131.61	45.32
LDEC007715-RA	rho gtpase-activating protein 21 isoform x7	-2.90	80.80	27.84
LDEC014285-RA	sh3 domain-containing kinase-binding protein 1 isoform x1	-2.90	206.47	71.23
LDEC022670-RA	l-xylulose reductase	-2.89	80.99	28.00
LDEC000223-RA	protein jumonji	-2.89	96.25	33.29
LDEC003293-RA	serine threonine-protein phosphatase 2b catalytic subunit 2-like isoform x1	-2.89	439.17	152.19
LDEC010666-RA	mrg morf4l-binding protein	-2.88	109.60	38.00
LDEC017979-RA	phosphofurin acidic cluster sorting protein 2 isoform x3	-2.88	97.39	33.76
LDEC000572-RA	adp-ribosylation factor-like protein 8b-a	-2.88	94.25	32.69
LDEC001837-RA	zinc finger protein 271-like	-2.88	94.58	32.83
LDEC014316-RA	serine palmitoyltransferase 1	-2.88	291.02	101.01
LDEC020160-RA	histone-lysine n-methyltransferase setmar	-2.88	96.53	33.52
LDEC007575-RA	e3 ubiquitin-protein ligase ubr4 isoform x1	-2.88	476.34	165.43
LDEC005801-RA	multiple pdz domain protein	-2.88	122.91	42.69
LDEC020383-RA	rna-directed dna polymerase from mobile element jockey-like	-2.88	158.42	55.05
LDEC016265-RA	annexin b10-like	-2.88	89.78	31.22
LDEC004450-RA	phospholipase a2 inhibitor-like	-2.88	91.97	31.98
LDEC009925-RA	lipophorin receptor	-2.87	286.46	99.65
LDEC007271-RA	ubiquitin-protein ligase e3c-like	-2.87	105.99	36.89
LDEC010542-RA	mlx-interacting protein isoform x3	-2.87	111.98	39.01
LDEC000580-RA	agap007663-pa-like protein	-2.87	308.94	107.73
LDEC020734-RA	aquaporin -like	-2.87	254.00	88.59
LDEC021754-RA	chromatin assembly factor 1 subunit a-b-like	-2.86	108.42	37.85
LDEC002277-RA	actin-related protein 2 3 complex subunit 3-like	-2.86	142.54	49.82
LDEC007533-RA	major facilitator superfamily domain-containing protein 9-like	-2.86	186.74	65.38
LDEC003787-RA	gamma-interferon-inducible lysosomal thiol reductase-like isoform x2	-2.85	82.61	28.94
LDEC021303-RA	thyroid receptor-interacting protein 11-like	-2.84	116.78	41.18
LDEC007345-RA	low quality protein: leucine-rich repeat-containing protein ddb_g0290503	-2.84	133.56	47.10
LDEC016470-RA	repressor of rna polymerase iii transcription maf1 homolog	-2.83	243.07	85.75
LDEC016007-RA	poly -specific endoribonuclease homolog	-2.83	210.94	74.56
LDEC012940-RA	PREDICTED: LOW QUALITY PROTEIN: uncharacterized protein LOC658528	-2.83	986.62	348.99
LDEC005223-RA	tyrosine-protein kinase hopscotch	-2.82	104.90	37.14
LDEC012948-RA	camp-responsive element-binding 2	-2.82	154.42	54.69
LDEC001932-RA	protein lsm12 homolog	-2.82	117.59	41.70
LDEC022378-RA	eukaryotic translation initiation factor 3 subunit m	-2.82	393.12	139.54
LDEC002775-RA	S-antigen protein, putative	-2.82	398.20	141.38
LDEC004975-RA	regulator of nonsense transcripts 2	-2.81	99.29	35.30
LDEC001880-RA	protein pat1 homolog 1	-2.81	179.52	63.91

LDEC008542-RA	protein rer1	-2.80	113.17	40.36
LDEC009141-RA	makorin isoform a	-2.80	103.90	37.10
LDEC011057-RA	dis3-like exonuclease 2-like isoform x1	-2.80	171.25	61.19
LDEC017833-RA	protein tyrosine phosphatase type iva 1	-2.79	903.25	323.29
LDEC000245-RA	tyrosine phosphatase	-2.79	159.13	56.99
LDEC016219-RA	protein fam46c isoform x3	-2.79	127.43	45.70
LDEC010098-RA	ww domain-binding protein 2	-2.78	380.33	136.69
LDEC020095-RA	nuclear pore complex protein nup153	-2.77	201.76	72.74
LDEC015856-RA	ubiquitin carboxyl-terminal	-2.77	148.20	53.52
LDEC009435-RA	aldose reductase	-2.77	194.02	70.08
LDEC018368-RA	btb poz domain-containing protein kctd10	-2.77	117.07	42.29
LDEC014690-RA	anamorsin homolog	-2.77	180.85	65.38
LDEC013583-RA	glutathione s-transferase omega-1	-2.75	103.38	37.54
LDEC000075-RA	interference hedgehog-like isoform x1	-2.75	138.03	50.19
LDEC003276-RA	28 kda heat- and acid-stable phosphoprotein	-2.75	349.44	127.18
LDEC003165-RA	e3 ubiquitin-protein ligase herc2 isoform x1	-2.74	182.13	66.36
LDEC004864-RA	40s ribosomal protein s15	-2.74	277.15	101.12
LDEC007731-RA	cd63 antigen-like	-2.74	125.38	45.76
LDEC002629-RA	rna-binding protein nob1	-2.74	160.27	58.50
LDEC002382-RA	nucleosome assembly protein 1-like 1	-2.73	1727.56	632.25
LDEC008710-RA	serine protease p146	-2.73	224.53	82.25
LDEC002105-RA	ubx domain-containing protein 4	-2.71	197.53	72.80
LDEC015368-RA	5 -amp-activated protein kinase catalytic subunit alpha-2	-2.70	101.57	37.58
LDEC022301-RA	upf0505 protein c16orf62 homolog	-2.70	116.07	43.00
LDEC010571-RA	homogentisate -dioxygenase	-2.69	95.01	35.26
LDEC005965-RA	tyrosine aminotransferase	-2.69	974.93	362.02
LDEC012306-RA	15-hydroxyprostaglandin dehydrogenase	-2.68	557.05	207.88
LDEC000728-RA	ubiquitin-fold modifier 1	-2.68	110.79	41.39
LDEC013716-RA	isocitrate dehydrogenase	-2.67	179.33	67.28
LDEC002874-RA	zinc finger protein noc	-2.66	141.45	53.16
LDEC019753-RA	protein beta isoform-like isoform x1	-2.66	105.28	39.65
LDEC011629-RA	serine threonine-protein phosphatase pp1-beta catalytic subunit isoform x2	-2.64	122.34	46.26
LDEC016193-RA	supervillin-like isoform x1	-2.64	139.69	52.83
LDEC011065-RA	protein xmas-2	-2.64	114.31	43.25
LDEC004635-RA	cathepsin l protease inhibitor 1	-2.63	187.55	71.27
LDEC015738-RA	calcium uptake protein mitochondrial isoform x3	-2.63	101.05	38.40
LDEC004351-RA	microtubule-associated protein futsch isoform x5	-2.63	240.36	91.44
LDEC018941-RA	alpha partial	-2.63	303.67	115.57
LDEC010230-RA	protein sda1 homolog	-2.63	142.30	54.17
LDEC008009-RA	map microtubule affinity-regulating kinase 3	-2.61	103.61	39.65
LDEC010455-RA	leucine-rich repeat neuronal protein 1-like	-2.60	154.57	59.40

LDEC017891-RA	isocitrate dehydrogenase	-2.60	486.85	187.18
LDEC018309-RA	mucin-2-like isoform x2	-2.60	126.52	48.67
LDEC014477-RA	brahma-associated protein of 60 kda isoform x2	-2.60	115.07	44.32
LDEC017046-RA	v-type proton atpase 116 kda subunit a isoform 1	-2.59	252.53	97.42
LDEC007313-RA	tubulin-folding cofactor b	-2.59	111.17	42.96
LDEC010472-RA	chondroitin sulfate synthase 2	-2.59	147.96	57.18
LDEC021714-RA	extended synaptotagmin-2-b isoform x3	-2.59	140.55	54.35
LDEC016863-RA	pleckstrin homology-like domain family b member 1 isoform x2	-2.58	103.61	40.16
LDEC007938-RA	leucine-rich repeat-containing protein 47-like	-2.58	98.86	38.33
LDEC005599-RA	amidophosphoribosyltransferase	-2.58	292.93	113.65
LDEC014841-RA	longitudinals lacking	-2.57	126.52	49.15
LDEC008586-RA	probable deoxyhypusine synthase	-2.57	148.34	57.68
LDEC007677-RA	transport and golgi organization protein 1 isoform x1	-2.56	358.66	140.04
LDEC002088-RA	oxidoreductase glyr1 homolog	-2.56	177.29	69.23
LDEC002774-RA	mediator of rna polymerase ii transcription subunit 15-like	-2.56	530.29	207.26
LDEC022355-RA	traf2 and nck interacting tnik	-2.56	153.95	60.19
LDEC007737-RA	peptidyl-prolyl cis-trans isomerase-like 2-like	-2.55	107.89	42.27
LDEC005957-RA	autophagy-related protein 9a isoform x3	-2.55	138.69	54.36
LDEC010405-RA	maternal protein pumilio isoform x5	-2.55	207.89	81.50
LDEC014068-RA	PREDICTED: uncharacterized protein CG1785 isoform X1	-2.55	131.51	51.57
LDEC017653-RA	mitochondrial fission 1 protein	-2.55	150.67	59.08
LDEC004319-RA	mucin-17-like isoform x1	-2.55	206.90	81.15
LDEC001868-RA	traf-type zinc finger domain-containing protein 1-like isoform x2	-2.55	118.59	46.55
LDEC021491-RA	neurofilament heavy polypeptide-like isoform x2	-2.54	119.82	47.10
LDEC006844-RA	exportin-5 isoform x2	-2.54	144.97	56.99
LDEC014749-RA	atp-dependent clp protease atp-binding subunit clpx- mitochondrial isoform x2	-2.54	123.24	48.50
LDEC021800-RA	cytochrome p450 9z4	-2.54	211.36	83.22
LDEC009947-RA	protein diaphanous isoform x3	-2.54	114.59	45.15
LDEC002269-RA	calcium calmodulin-dependent protein kinase kinase	-2.53	127.95	50.67
LDEC008816-RA	homolog of isoform b	-2.53	152.05	60.21
LDEC008517-RA	transmembrane protein 62-like isoform x1	-2.53	246.44	97.59
LDEC009371-RA	ap-3 complex subunit sigma-2 isoform x1	-2.52	99.86	39.59
LDEC022507-RA	ccr4-not transcription	-2.51	132.09	52.54
LDEC010139-RA	cysteine--trna cytoplasmic-like	-2.51	128.76	51.26
LDEC015642-RA	syntenin-1	-2.51	260.18	103.61
LDEC002294-RA	isoform a	-2.51	179.71	71.67
LDEC002675-RA	serine threonine-protein kinase smg1	-2.50	132.47	52.89
LDEC017992-RA	PREDICTED: uncharacterized protein LOC100141567 isoform X3	-2.50	392.50	156.73
LDEC016738-RA	seven in	-2.50	105.09	41.99

LDEC020033-RA	nad-dependent deacetylase sirtuin-2	-2.50	107.32	42.90
LDEC008773-RA	15 kda selenoprotein	-2.50	108.04	43.19
LDEC019588-RA	fatty acid synthase	-2.50	124.86	49.96
LDEC014891-RA	tryptophanyl-trna cytoplasmic	-2.50	161.89	64.81
LDEC007084-RA	n-alpha-acetyltransferase 60 isoform x2	-2.49	170.25	68.45
LDEC013335-RA	elongation factor 1-alpha	-2.49	208.85	83.99
LDEC013178-RA	bromodomain-containing protein 8	-2.48	106.47	42.85
LDEC000494-RA	---NA---	-2.48	369.88	149.01
LDEC003904-RA	protein mothers against dpp	-2.48	108.89	43.92
LDEC007077-RA	tom1-like protein 2 isoform x1	-2.47	168.49	68.12
LDEC002389-RA	cwf19-like protein 2 homolog	-2.47	253.14	102.56
LDEC008902-RA	protein crossbronx homolog	-2.47	150.76	61.09
LDEC003341-RA	ubiquitin carboxyl-terminal hydrolase 47 isoform x1	-2.47	200.81	81.46
LDEC021274-RA	ubiquitin carboxyl-terminal hydrolase 24-like isoform x1	-2.46	182.13	73.93
LDEC001970-RA	fatty acyl- reductase cg5065 isoform x1	-2.46	264.27	107.29
LDEC007233-RA	suppressor of presenilin protein 4 isoform x4	-2.46	208.51	84.83
LDEC024633-RA	nadh dehydrogenase subunit 4	-2.46	1497.99	609.79
LDEC020216-RA	serine hydroxymethyltransferase	-2.45	912.14	372.23
LDEC003847-RA	alpha-aminoadipic semialdehyde mitochondrial isoform x1	-2.45	138.64	56.66
LDEC016594-RA	ac transposable element-derived protein partial	-2.44	161.84	66.23
LDEC005040-RA	n-myc protein	-2.44	172.06	70.46
LDEC009300-RA	yellow-1 precursor	-2.43	251.76	103.69
LDEC007489-RA	cyclic amp-responsive element-binding protein 1 isoform x1	-2.42	243.83	100.76
LDEC022614-RA	probable serine incorporator isoform x3	-2.42	143.35	59.35
LDEC002429-RA	membrane-associated progesterone receptor component 1-like	-2.41	569.55	235.95
LDEC020215-RA	ubiquitin carboxyl-terminal hydrolase 7-like	-2.41	144.25	59.94
LDEC021269-RA	cullin-4b-like	-2.40	119.01	49.50
LDEC019936-RA	carnitine o-palmitoyltransferase mitochondrial	-2.40	130.66	54.36
LDEC005598-RA	phosphoribosylaminoimidazole carboxylase	-2.40	312.13	129.98
LDEC005740-RA	small integral membrane protein 14-like isoform x1	-2.40	269.49	112.39
LDEC019900-RA	probable serine hydrolase	-2.40	136.74	57.05
LDEC002538-RA	dynein light chain cytoplasmic	-2.40	182.04	75.98
LDEC020451-RA	thyroid receptor-interacting protein 11	-2.39	138.22	57.72
LDEC010436-RA	docking protein 2	-2.39	113.83	47.60
LDEC011702-RA	ubiquitin carboxyl-terminal hydrolase 45	-2.38	206.71	86.75
LDEC001534-RA	cytokine receptor	-2.38	287.36	120.82
LDEC001096-RA	hypothetical protein TcasGA2_TC004643	-2.38	157.56	66.28
LDEC008574-RA	large proline-rich protein bag6-like	-2.36	234.51	99.22
LDEC002457-RA	26s proteasome non-atpase regulatory subunit 12	-2.36	307.71	130.40
LDEC020333-RA	prostaglandin f synthase-like	-2.36	185.60	78.74

LDEC015589-RA	sh3 domain-binding protein 5 homolog	-2.35	215.50	91.67
LDEC011062-RA	PREDICTED: uncharacterized protein LOC659663	-2.34	127.90	54.67
LDEC015682-RA	acylamino-acid-releasing enzyme	-2.34	168.02	71.82
LDEC006708-RA	cop9 signalosome complex subunit 8-like	-2.34	114.93	49.17
LDEC009346-RA	wd sam and u-box domain-containing protein 1-like	-2.34	127.90	54.77
LDEC003053-RA	translationally-controlled tumor protein homolog	-2.33	1538.16	658.92
LDEC013900-RA	dynein regulatory complex protein 1	-2.33	244.26	104.76
LDEC010048-RA	26s proteasome complex subunit dss1	-2.32	113.98	49.11
LDEC016561-RA	autophagy-related protein 101	-2.31	126.71	54.80
LDEC003274-RA	serine arginine-rich splicing factor 2 isoform x2	-2.31	148.63	64.29
LDEC013802-RA	transmembrane protein 256 homolog	-2.30	119.20	51.87
LDEC002042-RA	3-phosphoinositide-dependent protein kinase 1-like	-2.29	118.87	51.80
LDEC004252-RA	cationic amino acid transporter 2 isoform x2	-2.29	138.60	60.40
LDEC000643-RA	probable protein phosphatase 2c	-2.29	256.99	112.39
LDEC008817-RA	gamma-interferon-inducible lysosomal thiol reductase	-2.29	240.03	104.99
LDEC010528-RA	-like protein subfamily c member 11	-2.28	131.13	57.43
LDEC007591-RA	agap005575-pa-like protein	-2.28	285.75	125.21
LDEC001731-RA	acidic leucine-rich nuclear phosphoprotein 32 family member a	-2.27	223.63	98.46
LDEC015101-RA	serine threonine-protein kinase mark2 isoform x1	-2.27	281.80	124.35
LDEC008144-RA	cytochrome p450 6a2	-2.26	723.54	319.63
LDEC019699-RA	branched-chain-amino-acid cytosolic	-2.26	158.13	69.91
LDEC010549-RA	kruppel-like protein 1	-2.26	143.21	63.35
LDEC003356-RA	mitochondrial carrier homolog 2-like isoform x1	-2.26	136.55	60.44
LDEC007973-RA	smoothelin-like protein 1 isoform x6	-2.26	176.72	78.32
LDEC001488-RA	serine threonine-protein kinase	-2.25	121.58	54.08
LDEC001178-RA	PREDICTED: uncharacterized protein LOC656855 isoform X3	-2.24	1025.22	458.14
LDEC013856-RA	nedd8-conjugating enzyme ubc12	-2.24	209.18	93.57
LDEC006600-RA	uncharacterized family 31 glucosidase kiaa1161 isoform x2	-2.23	143.97	64.46
LDEC000445-RA	longitudinals lacking isoform 4	-2.23	902.54	404.50
LDEC009045-RA	protein mo25	-2.22	213.88	96.18
LDEC014615-RA	microsomal glutathione s-transferase 1	-2.22	313.41	141.19
LDEC007388-RA	ammecr1-like protein	-2.22	252.29	113.71
LDEC001136-RA	protein slowmo	-2.22	323.96	146.13
LDEC005887-RA	adenylosuccinate lyase	-2.22	185.13	83.55
LDEC005244-RA	serine arginine repetitive matrix protein 1 isoform x1	-2.21	187.50	84.68
LDEC012729-RA	ets dna-binding protein pokkuri	-2.21	255.90	115.57
LDEC003739-RA	ubiquinone biosynthesis protein mitochondrial	-2.21	152.29	68.83
LDEC005630-RA	myotubularin-related protein 3	-2.21	157.51	71.28
LDEC022506-RA	ccr4-not transcription complex subunit 1 isoform 1	-2.20	424.01	192.78

LDEC017398-RA	dentin sialophospho	-2.20	282.56	128.49
LDEC018431-RA	rab3 gtpase-activating protein non-catalytic subunit	-2.19	135.27	61.68
LDEC001354-RA	PREDICTED: uncharacterized protein LOC664426 isoform X1	-2.19	281.95	128.75
LDEC007239-RA	dnaj homolog subfamily a member 2	-2.18	537.99	246.62
LDEC019372-RA	protein ssxt isoform x1	-2.18	240.07	110.18
LDEC019196-RA	protein slit-like	-2.18	173.01	79.51
LDEC006745-RA	nuclear protein 1	-2.17	282.56	129.98
LDEC016181-RA	e3 ubiquitin-protein ligase trip12 isoform x1	-2.17	232.09	106.83
LDEC015758-RA	acidic mammalian chitinase-like	-2.16	267.21	123.73
LDEC016955-RA	tether containing ubx domain for glut4-like	-2.16	227.14	105.18
LDEC011884-RA	antichymotrypsin-2-like isoform x2	-2.16	422.78	196.03
LDEC021164-RA	srsf protein kinase 1	-2.16	188.98	87.65
LDEC009508-RA	plexin domain-containing protein 2-like	-2.15	174.20	81.04
LDEC012643-RA	sorbin and sh3 domain-containing protein 1	-2.15	186.93	87.02
LDEC002750-RA	sodium potassium-transporting atpase subunit beta-1	-2.14	159.08	74.20
LDEC013339-RA	isoform a	-2.13	472.87	221.75
LDEC013018-RA	actin-related protein 5	-2.12	217.88	102.62
LDEC013744-RA	scavenger receptor class b member 1	-2.12	221.58	104.46
LDEC018589-RA	leucine-rich repeat-containing protein ddb_g0290503 isoform x1	-2.12	327.76	154.97
LDEC014777-RA	protein angel isoform x2	-2.11	330.71	157.06
LDEC010351-RA	tropomodulin isoform x1	-2.10	479.34	228.72
LDEC016586-RA	kynurenine alpha-aminoadipate mitochondrial-like	-2.08	312.46	150.54
LDEC016932-RA	nose resistant to fluoxetine protein 6-like	-2.07	150.48	72.66
LDEC000770-RA	monocarboxylate transporter	-2.06	149.05	72.28
LDEC002400-RA	cubilin	-2.06	273.68	132.80
LDEC011512-RA	atp-binding cassette sub-family a member 3-like	-2.06	255.85	124.25
LDEC004105-RA	chitin synthase	-2.05	181.71	88.70
LDEC002701-RA	probable atp-dependent rna helicase ddx17-like	-2.04	1014.85	496.58
LDEC007757-RA	pyridoxal kinase	-2.04	218.83	107.08
LDEC024057-RA	eukaryotic translation initiation factor 3 subunit e-like	-2.04	283.89	139.14
LDEC011020-RA	lim and sh3 domain protein lasp	-2.04	327.38	160.52
LDEC007117-RA	tetratricopeptide repeat protein 26-like isoform x2	-2.03	234.27	115.32
LDEC005395-RA	atp synthase subunit mitochondrial	-2.03	269.59	132.76
LDEC007931-RA	exonuclease 3 -5 domain-like-containing protein 1	-2.03	260.56	128.37
LDEC018331-RA	polyadenylate-binding protein-interacting protein 1-like isoform 2	-2.02	159.13	78.60
LDEC003788-RA	gamma-interferon-inducible lysosomal thiol reductase-like	-2.02	152.48	75.33
LDEC015587-RA	e3 ubiquitin-protein ligase rnf185-like isoform x2	-2.02	166.35	82.23
LDEC006581-RA	nascent polypeptide-associated complex subunit muscle-specific form-like	-2.01	184.18	91.81
LDEC017911-RA	flavin-containing monooxygenase fmo	-2.01	180.47	90.01

	gs-ox-like 3-like			
LDEC019172-RA	isoform c	-2.00	677.44	338.24
LDEC005504-RA	PREDICTED: sideroflexin-3	-2.00	166.50	83.30
LDEC024631-RA	nadh dehydrogenase subunit 5	-2.00	1411.63	707.33
LDEC018764-RA	PREDICTED: uncharacterized protein LOC661670 isoform X2	-2.00	188.79	94.62
LDEC006992-RA	calpain-b isoform x1	-1.99	226.29	113.50
LDEC000050-RA	mitochondrial import receptor subunit tom70-like	-1.99	213.22	107.00
LDEC005492-RA	ribose-phosphate pyrophosphokinase 1 isoform x1	-1.99	156.61	78.66
LDEC007866-RA	vinculin isoform x1	-1.99	299.53	150.85
LDEC024548-RA	cytochrome p450 6a2	-1.98	1065.33	538.45
LDEC008777-RA	serine threonine-protein phosphatase 4 regulatory subunit 1-like isoform x10	-1.98	208.09	105.22
LDEC003658-RA	phosphoribosylformylglycinamidine synthase-like	-1.97	262.84	133.33
LDEC007303-RA	protein preli-like	-1.97	197.49	100.24
LDEC006054-RA	protein lap4-like isoform 1	-1.97	205.09	104.30
LDEC016847-RA	neuroplastin isoform x1	-1.95	856.68	438.82
LDEC013712-RA	ubiquitin carboxyl-terminal hydrolase 2 isoform x2	-1.95	236.94	121.66
LDEC011511-RA	atp-binding cassette sub-family a member 3-like isoform x4	-1.95	217.83	111.87
LDEC007747-RA	ctl-like protein 1	-1.94	363.51	187.16
LDEC008329-RA	rhomboid protein mitochondrial	-1.94	174.62	89.95
LDEC022662-RA	nadh dehydrogenase subunit 1	-1.93	510.47	264.60
LDEC005971-RA	atp-binding cassette sub-family f member 2	-1.92	263.84	137.32
LDEC018195-RA	atp-dependent rna helicase belle	-1.91	240.12	125.74
LDEC006478-RA	muscle protein 20-like protein	-1.90	3160.25	1660.30
LDEC014018-RA	f-box only protein 28	-1.89	217.40	115.13
LDEC012185-RA	renin receptor	-1.89	233.66	123.83
LDEC019777-RA	glycosyl hydrolase	-1.89	506.48	268.60
LDEC010476-RA	copper-transporting atpase 1 isoform x4	-1.88	224.15	119.42
LDEC010114-RA	PREDICTED: uncharacterized protein LOC658141 isoform X2	-1.88	188.69	100.55
LDEC001941-RA	hypothetical protein TcasGA2_TC015166	-1.87	188.50	100.55
LDEC003614-RA	60s ribosomal protein l32-like	-1.87	1350.13	722.28
LDEC001822-RA	serine threonine-protein phosphatase alpha-2 isoform	-1.86	293.69	157.84
LDEC009642-RA	programmed cell death protein 4	-1.86	275.01	148.18
LDEC005983-RA	filamin-c isoform x1	-1.85	212.32	114.78
LDEC003986-RA	nadph--cytochrome p450 reductase isoform x2	-1.85	1323.08	715.70
LDEC001405-RA	sortilin-related receptor-like	-1.85	283.94	153.70
LDEC011496-RA	leucine-rich repeat-containing protein ddb_g0290503	-1.85	252.00	136.48
LDEC022187-RA	cathepsin b	-1.84	238.60	129.50
LDEC020749-RA	gamma-aminobutyric acid receptor-associated protein	-1.83	372.92	203.35
LDEC001412-RA	leucine--trna cytoplasmic	-1.83	414.41	226.94
LDEC008233-RA	protein translation factor sui1 homolog	-1.82	292.21	160.41

LDEC002758-RA	chitin deacetylase 1 precursor	-1.82	1700.23	935.27
LDEC005077-RA	cytochrome b5-related	-1.82	353.10	194.37
LDEC011341-RA	isoform p	-1.81	845.22	466.72
LDEC007424-RA	nuclear pore complex protein ddb_g0274915 homolog isoform x1	-1.79	319.97	178.44
LDEC005952-RA	rna-binding protein 39	-1.79	250.10	140.00
LDEC004461-RA	luciferase homolog	-1.78	455.81	256.26
LDEC008099-RA	four and a half lim domains	-1.78	934.62	525.78
LDEC018739-RA	chloride intracellular channel exl-	-1.77	308.33	174.03
LDEC000858-RA	fatty acid synthase	-1.76	635.95	360.51
LDEC004640-RA	protein cepu-1	-1.76	321.73	182.52
LDEC003012-RA	trifunctional purine biosynthetic protein adenosine-3	-1.76	221.73	126.17
LDEC007734-RA	cd63 antigen	-1.75	232.14	132.55
LDEC020629-RA	PREDICTED: prosaposin	-1.75	598.26	342.53
LDEC001865-RA	protein-methionine sulfoxide oxidase mical3 isoform x8	-1.73	355.43	205.12
LDEC016789-RA	myeloid leukemia factor isoform x4	-1.72	243.54	141.19
LDEC001299-RA	protein cdv3 homolog	-1.71	296.97	174.05
LDEC010050-RA	proton-coupled amino acid transporter 4 isoform x3	-1.71	457.81	268.33
LDEC010566-RA	serine threonine-protein phosphatase 2a catalytic subunit alpha isoform	-1.69	456.81	270.33
LDEC019788-RA	cysteine proteinase	-1.65	856.44	518.88
LDEC001346-RA	ribosomal protein l12e	-1.64	739.99	451.53
LDEC015524-RA	glutathione s-transferase-like	-1.63	543.98	333.83
LDEC000775-RA	ubiquitin ribosomal protein s30e fusion protein	-1.63	908.39	557.54
LDEC001744-RA	ribosomal protein l24e	-1.62	804.68	496.27
LDEC018424-RA	ribonuclease x25	-1.62	457.66	282.63
LDEC018083-RA	eukaryotic translation initiation factor 3 subunit h	-1.62	292.59	181.03
LDEC013069-RA	h+ transporting atp synthase o subunit isoform 1	-1.59	557.48	350.81
LDEC018653-RA	atp-dependent rna helicase me31b	-1.58	309.75	195.53
LDEC017506-RA	importin-5	-1.56	703.25	450.82
LDEC008530-RA	thioredoxin-2-like isoform x2	-1.56	875.21	561.27
LDEC002760-RA	chitin deacetylase 2 isoform b precursor	-1.55	951.59	614.10
LDEC002600-RA	ribosomal protein s18	-1.54	1137.62	738.16
LDEC014064-RA	ferritin subunit	-1.53	1300.70	852.58
LDEC018453-RA	ribosomal protein l5	-1.52	382.47	252.43
LDEC024629-RA	cytochrome c oxidase subunit iii	-1.51	5727.33	3786.83
LDEC021171-RA	ribosomal protein l5	-1.51	2212.98	1469.12
LDEC008598-RA	40s ribosomal protein s15aa	-1.46	878.16	603.26
LDEC008040-RA	PREDICTED: uncharacterized protein LOC103314979	-1.45	504.05	348.28
LDEC003299-RA	muscle m-line assembly protein unc-89 isoform x2	-1.41	608.43	430.45
LDEC024126-RA	60s ribosomal protein l7a-like	-1.39	762.47	549.74
LDEC016655-RA	40s ribosomal protein s12	-1.38	752.49	544.33

LDEC008220-RA	60s ribosomal protein l28	-1.38	748.50	542.89
LDEC009012-RA	microtubule-actin cross-linking factor 1 isoform x9	-1.38	615.41	446.55
LDEC016749-RA	integumentary mucin -like isoform x1	-1.33	887.43	666.78
LDEC011311-RA	abhydrolase domain-containing protein 3-like	-1.30	1237.10	949.81
LDEC017569-RA	adenine nucleotide translocase	-1.28	7282.12	5704.56
LDEC021542-RA	chitin deacetylase 1	-1.25	2366.74	1891.54
LDEC005654-RA	chitin-binding protein	1.19	2317.98	2757.49
LDEC000809-RA	peptidyl-prolyl cis-trans isomerase	1.23	1371.52	1680.50
LDEC009708-RA	low quality protein: short form-like	1.24	2346.49	2907.48
LDEC012644-RA	s-adenosylmethionine synthetase	1.24	1705.51	2115.03
LDEC005894-RA	40s ribosomal protein s5	1.26	1031.35	1296.88
LDEC013855-RA	60s ribosomal protein l18	1.28	780.44	995.57
LDEC018144-RA	myosin heavy chain	1.29	8374.40	10838.33
LDEC006479-RA	muscle lim protein mlp84b-like isoform x1	1.33	1538.87	2046.06
LDEC001536-RA	elongation factor 1-alpha isoform x1	1.34	7473.81	10023.35
LDEC017098-RA	atp synthase	1.38	746.79	1027.06
LDEC010151-RA	v-type proton atpase 16 kda proteolipid subunit	1.40	1068.37	1490.62
LDEC005914-RA	ribosomal protein l7e	1.40	895.94	1251.18
LDEC003875-RA	eukaryotic translation initiation factor 3 subunit a	1.41	364.27	515.40
LDEC021641-RA	elongation factor 1 beta	1.44	423.20	607.88
LDEC017096-RA	atp synthase subunit mitochondrial-like	1.45	527.72	765.10
LDEC018238-RA	agap004396-pa-like protein	1.45	350.29	507.87
LDEC002143-RA	40s ribosomal protein s24	1.45	526.34	765.16
LDEC008057-RA	ribosomal protein s6	1.47	1039.67	1526.99
LDEC003355-RA	agap006602-pa-like protein	1.48	302.53	446.70
LDEC010981-RA	fatty acid synthase	1.49	473.35	703.52
LDEC005976-RA	imaginal disc growth factor 2 precursor	1.49	276.86	412.26
LDEC012938-RA	vigilin	1.50	279.05	417.34
LDEC005034-RA	alpha- partial	1.50	468.26	700.55
LDEC003531-RA	filamin-a isoform x3	1.51	328.00	495.10
LDEC014917-RA	ribosomal protein l36e	1.52	261.27	397.72
LDEC007118-RA	60s ribosomal protein l9	1.52	619.41	942.95
LDEC014122-RA	transcriptional regulator def1	1.53	237.27	361.90
LDEC018073-RA	ribosomal protein l17	1.53	688.23	1053.29
LDEC003475-RA	golgin subfamily a member 2-like	1.53	258.56	396.03
LDEC004599-RA	transketolase-like protein 2 isoform x1	1.54	349.72	537.78
LDEC012448-RA	40s ribosomal protein s20	1.54	367.17	565.89
LDEC024338-RA	long form-like	1.55	486.61	751.82
LDEC009322-RA	60s ribosomal protein l3	1.55	1046.46	1617.92
LDEC005763-RA	nucleoplasmin isoform 2	1.55	325.01	502.86
LDEC023007-RA	60s ribosomal protein l6	1.55	783.67	1213.05
LDEC014796-RA	---NA---	1.56	364.27	567.37

LDEC006675-RA	aspartate cytoplasmic	1.56	237.03	370.03
LDEC011313-RA	facilitated trehalose transporter tret1-like	1.56	920.27	1438.15
LDEC005337-RA	60s acidic ribosomal protein p0	1.57	1755.84	2752.51
LDEC003400-RA	myosin light chain alkali isoform x1	1.58	896.98	1414.26
LDEC000193-RA	60s ribosomal protein l21	1.58	293.35	463.01
LDEC010949-RA	isoform b	1.59	384.04	610.88
LDEC010720-RA	14-3-3 zeta	1.60	325.82	521.57
LDEC003536-RA	polyadenylate-binding protein 1-like isoform 1	1.61	2155.28	3473.33
LDEC013831-RA	v-type proton atpase subunit e	1.61	271.20	437.12
LDEC000762-RA	aldose reductase	1.61	563.70	910.05
LDEC005396-RA	tubulin alpha-1 chain	1.62	3518.67	5702.44
LDEC005651-RA	ubiquitin carboxyl-terminal hydrolase 2-like isoform x2	1.63	733.91	1196.17
LDEC010650-RA	phosphoglucose isomerase	1.63	234.99	384.19
LDEC014165-RA	ribosomal protein s3	1.64	835.43	1367.65
LDEC011997-RA	protein transport protein sec23a isoform x2	1.64	256.42	421.52
LDEC000877-RA	nadp-dependent malic enzyme isoform x2	1.64	183.61	301.87
LDEC008161-RA	lysosomal aspartic protease	1.67	230.52	384.52
LDEC010126-RA	40s ribosomal protein sa	1.68	1767.87	2972.75
LDEC002053-RA	digestive cysteine protease intestain	1.69	448.25	757.65
LDEC005045-RA	activating transcription factor of chaperone-like	1.70	1123.98	1908.96
LDEC001264-RA	rna polymerase ii largest subunit	1.70	151.67	257.89
LDEC021974-RA	dentin sialophosphoprotein isoform x1	1.70	175.48	299.11
LDEC007240-RA	fumarate mitochondrial	1.71	149.96	255.74
LDEC007953-RA	protein d2-like isoform x2	1.71	173.67	296.20
LDEC005273-RA	tubulin beta-1 chain	1.71	178.85	305.43
LDEC008098-RA	four and a half lim domains protein 2 isoform x5	1.71	151.29	259.12
LDEC019194-RA	adenosine diphosphatase	1.71	137.84	236.29
LDEC006238-RA	proliferation-associated protein 2g4	1.72	171.63	295.91
LDEC009707-RA	long form-like	1.73	178.14	307.89
LDEC001493-RA	splicing factor 3b subunit 3	1.74	183.46	318.44
LDEC017598-RA	eukaryotic translation initiation factor 5a	1.74	159.18	276.31
LDEC008467-RA	60s ribosomal protein l44	1.74	345.40	599.71
LDEC002190-RA	glutamate synthase	1.74	134.56	233.86
LDEC022004-RA	lysosomal aspartic protease	1.75	824.74	1440.12
LDEC016361-RA	aspartate mitochondrial	1.76	368.55	648.75
LDEC018192-RA	ribosomal protein l8e	1.76	268.50	472.89
LDEC001677-RA	glycoside hydrolase family 28 protein	1.76	530.34	934.60
LDEC008683-RA	protein prrc2c isoform x6	1.76	224.25	395.23
LDEC008701-RA	protein l 37cc	1.76	173.44	306.08
LDEC023974-RA	muscular protein 20	1.77	153.57	271.48
LDEC002055-RA	digestive cysteine protease intestain	1.77	755.87	1338.29

LDEC004907-RA	dyslexia-associated protein kiaa0319-like protein	1.77	125.76	222.98
LDEC010962-RA	fructose -bisphosphate aldolase	1.78	441.79	785.59
LDEC008350-RA	elongation factor tu	1.78	142.78	254.50
LDEC000562-RA	translocator protein	1.79	259.37	463.01
LDEC012911-RA	cytochrome p450 6bq11	1.79	273.49	488.63
LDEC018226-RA	glycoside hydrolase family 1	1.79	625.02	1119.65
LDEC006014-RA	ribosomal protein l10ae	1.79	617.70	1107.12
LDEC018600-RA	nadh dehydrogenase	1.79	123.10	220.94
LDEC003442-RA	hypothetical protein YQE_03563, partial	1.79	214.69	385.36
LDEC007477-RA	lipoyltransferase mitochondrial	1.80	131.42	236.08
LDEC014262-RA	trifunctional enzyme subunit mitochondrial	1.80	116.02	208.93
LDEC009709-RA	long form	1.81	181.66	327.97
LDEC005097-RA	leucine-rich repeat and immunoglobulin-like domain-containing nogo receptor-interacting protein 2 isoform x1	1.81	119.49	215.96
LDEC010916-RA	von willebrand factor d and egf domain-containing protein	1.81	244.73	442.54
LDEC017156-RA	apolipophorin- partial	1.81	577.30	1046.58
LDEC008853-RA	elongation factor 1 gamma	1.82	393.40	717.01
LDEC003854-RA	telomere length regulation protein tel2 homolog	1.83	107.61	196.55
LDEC006678-RA	atp-dependent rna helicase wm6	1.84	129.85	238.38
LDEC002964-RA	dihydrolipoamide acetyltransferase component of pyruvate dehydrogenase	1.84	248.91	458.22
LDEC002683-RA	annexin isoform c	1.84	433.28	797.99
LDEC004858-RA	multiple coagulation factor deficiency protein 2 homolog	1.84	111.41	205.29
LDEC004199-RA	glucose-6-phosphate dehydrogenase isoform x1	1.85	121.44	224.22
LDEC009177-RA	cytochrome heme mitochondrial	1.85	152.57	282.38
LDEC002667-RA	unc93-like protein	1.85	140.88	260.98
LDEC021271-RA	atp-dependent rna helicase p62	1.86	714.32	1331.80
LDEC018983-RA	60s ribosomal protein l35a	1.86	286.98	535.13
LDEC018924-RA	v-type proton atpase subunit c	1.87	127.33	238.19
LDEC008158-RA	phd and ring finger domain-containing protein 1 isoform x1	1.87	109.98	205.94
LDEC010362-RA	probable gpi-anchored adhesin-like protein pga55	1.89	180.23	339.75
LDEC019895-RA	acyl- -binding protein	1.89	115.83	218.95
LDEC011577-RA	ribosomal protein l7ae	1.89	593.31	1123.33
LDEC014876-RA	abc transporter g family member 23-like isoform x2	1.90	122.25	231.73
LDEC006360-RA	60s ribosomal protein l27a	1.90	172.82	327.89
LDEC015117-RA	chromobox protein homolog 5	1.90	225.86	428.86
LDEC021000-RA	peroxisomal acyl-coenzyme a oxidase 3-like	1.90	326.62	620.85
LDEC021947-RA	leucine-rich ppr motif-containing mitochondrial	1.91	162.03	309.94
LDEC021581-RA	cyc_sarpe ame: full=cytochrome c	1.92	243.73	467.18
LDEC008668-RA	spondin-1	1.92	102.24	196.15

LDEC009203-RA	atpase inhibitor mai- mitochondrial	1.92	109.75	211.09
LDEC006842-RA	high mobility group protein	1.92	263.50	507.12
LDEC009033-RA	long-chain-fatty-acid-- ligase 4 isoform x10	1.93	467.17	900.76
LDEC005270-RA	atp synthase subunit mitochondrial	1.93	163.88	316.47
LDEC017401-RA	clathrin heavy chain	1.93	254.85	492.52
LDEC003929-RA	elongation of very long chain fatty acids protein aael008004	1.94	95.87	186.07
LDEC023006-RA	ribosomal protein s27e	1.95	190.21	371.27
LDEC010761-RA	6-phosphogluconate decarboxylating	1.96	379.52	742.93
LDEC014486-RA	2-oxoglutarate mitochondrial-like isoform x1	1.96	148.48	291.44
LDEC017095-RA	atp synthase subunit mitochondrial	1.97	233.89	460.94
LDEC001762-RA	alcohol dehydrogenase class-3-like	1.97	240.88	475.37
LDEC019539-RA	peritrophic matrix protein 1-c precursor	1.97	569.64	1124.40
LDEC014454-RA	udp-glucuronosyltransferase partial	1.99	109.51	217.69
LDEC018534-RA	calnexin	1.99	185.41	368.65
LDEC018225-RA	myrosinase 1-like	1.99	452.20	900.53
LDEC019168-RA	protein anoxia up-regulated isoform x4	1.99	106.13	211.52
LDEC021397-RA	fatty acyl- reductase 1-like	2.00	105.04	209.62
LDEC018545-RA	troponin i	2.01	770.03	1547.97
LDEC020217-RA	myosin heavy non-muscle isoform x1	2.01	243.59	489.80
LDEC001326-RA	ornithine decarboxylase antizyme partial	2.01	466.93	939.31
LDEC014944-RA	adp-ribosylation factor 1	2.02	156.52	315.42
LDEC003142-RA	retinoid-inducible serine carboxypeptidase-like	2.02	164.22	330.94
LDEC003855-RA	nucleolar protein 56	2.02	113.12	228.71
LDEC019904-RA	isoform a	2.02	92.30	186.84
LDEC003545-RA	isoform c	2.03	381.14	772.98
LDEC018586-RA	prophenoloxidase iv	2.04	83.56	170.34
LDEC006160-RA	far upstream element-binding protein 1	2.05	155.47	318.33
LDEC018229-RA	chitinase 1	2.06	241.17	496.00
LDEC013324-RA	60s acidic ribosomal protein p1	2.06	752.25	1550.48
LDEC002501-RA	60s ribosomal protein l6	2.06	151.05	311.56
LDEC012447-RA	neurofilament triplet m	2.06	121.34	250.51
LDEC008960-RA	talin-1 isoform x4	2.07	129.80	268.89
LDEC013922-RA	heterogeneous nuclear ribonucleoprotein a2 b1 homolog isoform x1	2.08	112.31	233.57
LDEC010207-RA	thioredoxin reductase mitochondrial isoform x5	2.08	325.15	677.51
LDEC015741-RA	hrp65 protein	2.09	119.68	249.99
LDEC016959-RA	nadh dehydrogenase	2.09	109.46	229.13
LDEC014178-RA	atp synthase lipid-binding mitochondrial	2.10	1318.09	2770.89
LDEC004943-RA	fatbody protein 3rev-g1	2.12	141.12	299.28
LDEC003903-RA	stress-induced-phosphoprotein 1	2.12	97.91	207.82
LDEC003866-RA	interferon-related developmental regulator 1	2.13	150.53	320.65
LDEC001424-RA	eukaryotic translation initiation factor 4b-like	2.14	112.46	240.18

LDEC000772-RA	sparc	2.14	294.35	628.70
LDEC006785-RA	probable multidrug resistance-associated protein lethal 03659	2.14	150.72	322.30
LDEC006619-RA	protein disulfide-isomerase	2.14	108.94	233.53
LDEC017885-RA	ribosomal protein l4e	2.15	82.99	178.17
LDEC016660-RA	60s ribosomal protein l34	2.17	93.92	203.85
LDEC008216-RA	calponin transgelin	2.17	103.71	225.22
LDEC020565-RA	choline-phosphate cytidylyltransferase a-like isoform x2	2.18	142.54	310.70
LDEC015854-RA	short-chain dehydrogenase	2.18	61.65	134.39
LDEC000125-RA	hydroxysteroid dehydrogenase-like protein 2	2.19	95.30	208.41
LDEC013640-RA	constitutive coactivator of ppar-gamma-like protein 1 isoform x3	2.19	63.97	140.02
LDEC001515-RA	phosphoglyceromutase	2.20	123.24	271.69
LDEC019971-RA	gamma-interferon-inducible lysosomal thiol reductase-like	2.21	85.70	189.23
LDEC004603-RA	peroxiredoxin prdx5	2.23	93.73	208.60
LDEC012374-RA	heat shock 70 kda protein cognate 5	2.24	68.63	153.88
LDEC000525-RA	serine threonine-protein phosphatase 2a 65 kda regulatory subunit a alpha isoform	2.24	105.66	236.96
LDEC015672-RA	troponin c	2.24	102.28	229.61
LDEC016599-RA	ubiquitin carboxyl-terminal hydrolase 2 isoform x2	2.25	86.84	195.50
LDEC001577-RA	nadh dehydrogenase	2.26	59.22	133.98
LDEC007395-RA	#NAME?	2.27	63.69	144.33
LDEC001622-RA	t-complex protein 1 subunit beta	2.27	96.01	217.59
LDEC004394-RA	protein croquemort-like isoform x4	2.27	64.07	145.58
LDEC010852-RA	tubulin beta-1 chain	2.27	1881.65	4276.86
LDEC013999-RA	stromal cell-derived factor 2	2.27	52.14	118.62
LDEC005909-RA	ubiquitin-like modifier-activating enzyme 1-like	2.28	184.42	421.02
LDEC009090-RA	pancreatic triacylglycerol lipase-like	2.29	77.38	176.93
LDEC013268-RA	na+ + atpase alpha-subunit 1	2.29	69.68	159.34
LDEC006485-RA	eukaryotic translation initiation factor 4 gamma 2 isoform x2	2.29	197.49	453.04
LDEC000771-RA	uracil-dna degrading isoform b	2.30	94.68	217.48
LDEC004320-RA	probable peroxisomal acyl-coenzyme a oxidase 1	2.30	94.68	217.82
LDEC005749-RA	dihydrolipoamide dehydrogenase e3 subunit	2.30	177.90	409.47
LDEC013286-RA	alpha- partial	2.31	60.41	139.41
LDEC019751-RA	afg3-like protein 2	2.31	77.71	179.90
LDEC017743-RA	serine palmitoyltransferase 2	2.32	78.80	182.50
LDEC016596-RA	globin-like partial	2.32	50.33	116.85
LDEC006858-RA	dolichyl-diphosphooligosaccharide--protein glycosyltransferase subunit 1	2.32	146.87	341.25
LDEC007862-RA	14-3-3 epsilon protein	2.33	73.58	171.28
LDEC001116-RA	catenin delta-2 isoform x2	2.34	51.52	120.63
LDEC009437-RA	aldose reductase	2.34	72.29	169.28
LDEC009874-RA	utp-glucose-1-phosphate uridylyltransferase 2	2.34	108.94	255.46

LDEC003583-RA	glutamate semialdehyde dehydrogenase	2.37	52.95	125.48
LDEC000701-RA	cytochrome p450	2.39	3111.06	7448.59
LDEC000760-RA	aldose reductase	2.40	476.20	1143.22
LDEC017981-RA	atp-dependent rna helicase p62	2.40	230.76	554.58
LDEC022001-RA	---NA---	2.40	518.45	1246.05
LDEC001114-RA	5-aminolevulinate mitochondrial	2.41	63.21	152.06
LDEC014011-RA	cg9135-pa	2.41	99.81	240.09
LDEC008519-RA	facilitated trehalose transporter tret1	2.42	79.71	192.53
LDEC009139-RA	digestive cysteine proteinase intestain	2.42	993.28	2399.21
LDEC016962-RA	cofilin actin-depolymerizing factor homolog	2.42	102.24	247.33
LDEC016969-RA	translocation associated membrane protein	2.42	192.45	465.61
LDEC016483-RA	innexin 2	2.43	83.27	202.40
LDEC018920-RA	transitional endoplasmic reticulum atpase ter94	2.44	254.57	620.04
LDEC024513-RA	ribosomal protein s8e	2.44	575.39	1401.61
LDEC015790-RA	heterogeneous nuclear ribonucleoprotein 27c isoform x8	2.44	50.14	122.18
LDEC001044-RA	aspartyl asparaginyl beta-hydroxylase isoform x1	2.44	70.91	173.06
LDEC000918-RA	glycosyl hydrolase	2.44	158.46	386.93
LDEC018298-RA	chymotrypsin inhibitor-like	2.45	120.20	294.18
LDEC004844-RA	glycoside hydrolase family protein 48	2.45	106.13	259.88
LDEC011032-RA	GA15068	2.45	43.16	105.87
LDEC013264-RA	low quality protein: papilin-like	2.45	68.06	167.02
LDEC000058-RA	glycogen phosphorylase	2.46	189.36	465.44
LDEC004009-RA	o-linked n-acetylglucosamine ogt	2.46	103.76	255.32
LDEC017373-RA	serine arginine repetitive matrix protein 1	2.46	148.72	366.33
LDEC016722-RA	protein ctla-2-alpha	2.46	87.22	214.87
LDEC015453-RA	atp synthase b mitochondrial	2.48	117.97	291.98
LDEC013654-RA	juvenile hormone esterase	2.48	50.71	125.65
LDEC014671-RA	40s ribosomal protein s11-like	2.48	194.16	481.10
LDEC004397-RA	t-complex protein 1 subunit delta	2.49	86.55	215.31
LDEC013827-RA	thioredoxin-related transmembrane protein 1-like	2.49	43.82	109.21
LDEC005975-RA	imaginal disc growth factor 4 precursor	2.50	268.97	671.13
LDEC009474-RA	chromodomain-helicase-dna-binding protein mi-2 homolog isoform x1	2.51	115.88	290.43
LDEC019182-RA	interleukin enhancer-binding factor 2 homolog	2.51	68.11	170.72
LDEC018261-RA	plasminogen activator inhibitor 1 rna-binding partial	2.51	89.21	224.24
LDEC021068-RA	glycosyl hydrolase	2.51	88.31	222.08
LDEC017247-RA	glutamine synthetase 2	2.52	65.97	166.41
LDEC000758-RA	endo-beta- -glucanase	2.52	445.12	1123.50
LDEC012533-RA	malate mitochondrial	2.53	269.54	680.62
LDEC017475-RA	arginine kinase	2.53	1493.29	3771.39
LDEC003313-RA	blastoderm-specific protein 25d- partial	2.53	44.92	113.52

LDEC008388-RA	hypothetical protein	2.53	64.17	162.19
LDEC011303-RA	beta- -glucanase	2.53	89.21	225.62
LDEC014169-RA	ribosomal protein s23	2.53	381.76	966.33
LDEC015134-RA	alpha-tocopherol transfer isoform x2	2.55	75.29	191.82
LDEC005003-RA	zinc transporter zip1	2.55	49.38	125.94
LDEC009204-RA	methyltransferase-like protein 23	2.56	106.09	272.05
LDEC010338-RA	pantothenate kinase mitochondrial-like	2.57	45.87	117.70
LDEC003275-RA	splicing arginine serine-rich 2	2.57	64.02	164.32
LDEC009165-RA	polya-binding protein	2.57	357.38	917.56
LDEC015355-RA	profilin- partial	2.57	57.99	149.26
LDEC004051-RA	oligopeptidase a	2.58	45.91	118.42
LDEC010269-RA	alanine aminotransferase 2 isoform x2	2.58	84.13	217.02
LDEC014837-RA	uncharacterized threonine-rich gpi-anchored glyco	2.59	105.37	272.42
LDEC022805-RA	probable histone-binding protein caf1	2.59	46.96	121.78
LDEC004781-RA	cytochrome c oxidase polypeptide iv	2.60	296.68	771.18
LDEC012384-RA	t-complex protein 1 subunit epsilon	2.60	46.06	119.75
LDEC021640-RA	nadh-ubiquinone reductase 75 kda subunit precursor	2.60	48.29	125.69
LDEC004810-RA	galectin-4-like isoform x2	2.62	44.06	115.36
LDEC019587-RA	coatomer subunit beta	2.62	85.60	224.60
LDEC010407-RA	juvenile hormone epoxide hydrolase-like protein 2	2.62	82.89	217.53
LDEC020428-RA	prostatic acid phosphatase-like	2.63	126.33	331.65
LDEC020286-RA	glycoside hydrolase family 45 protein	2.63	83.56	219.49
LDEC015723-RA	vacuolar atp synthase subunit g	2.63	76.14	200.36
LDEC000982-RA	40s ribosomal protein s2	2.63	911.43	2399.69
LDEC019737-RA	glutamate mitochondrial-like	2.64	93.30	245.86
LDEC017313-RA	transformer-2 protein homolog beta isoform x2	2.64	50.10	132.05
LDEC003052-RA	la protein homolog	2.64	40.35	106.56
LDEC009853-RA	glyceraldehyde-3-phosphate dehydrogenase	2.65	531.52	1406.40
LDEC004917-RA	probable serine hydrolase isoform x5	2.65	55.23	146.31
LDEC018015-RA	probable aconitate mitochondrial	2.66	59.55	158.19
LDEC007895-RA	atp-citrate synthase	2.66	62.31	165.53
LDEC018191-RA	60s ribosomal protein l8	2.66	386.37	1027.96
LDEC019597-RA	t-complex protein 1 subunit alpha	2.66	54.90	146.21
LDEC011895-RA	adipocyte plasma membrane-associated	2.67	195.49	522.33
LDEC021798-RA	gamma-interferon-inducible lysosomal thiol reductase-like	2.68	391.98	1049.76
LDEC005653-RA	mucin- partial	2.68	176.19	473.03
LDEC018966-RA	glyoxylate reductase hydroxypyruvate reductase-like	2.69	79.85	214.41
LDEC004861-RA	non-specific lipid-transfer protein	2.69	168.40	452.28
LDEC017679-RA	probable multidrug resistance-associated protein lethal 03659	2.71	57.37	155.20
LDEC011579-RA	tropomyosin 1	2.71	485.14	1313.98
LDEC019738-RA	glutamate mitochondrial	2.71	150.86	408.72

LDEC007868-RA	lamin dm0-like isoform x1	2.71	100.29	272.11
LDEC008349-RA	gtp-binding nuclear protein ran	2.71	104.33	283.11
LDEC008607-RA	enolase	2.72	335.99	913.27
LDEC005884-RA	probable aconitate mitochondrial	2.73	325.77	889.51
LDEC003518-RA	safb-like transcription modulator	2.73	69.25	189.35
LDEC009655-RA	chondroitin proteoglycan-2	2.73	80.47	220.02
LDEC003973-RA	hypothetical protein YQE_00903, partial	2.74	212.27	582.56
LDEC006099-RA	v-type proton atpase catalytic subunit a	2.75	432.00	1187.29
LDEC012375-RA	heat shock 70 kda protein cognate 5	2.76	152.33	419.97
LDEC005806-RA	serine--trna cytoplasmic	2.76	62.17	171.51
LDEC007471-RA	translocon-associated protein subunit delta	2.76	70.91	195.75
LDEC018591-RA	proteasome subunit alpha type-3	2.76	40.31	111.35
LDEC001198-RA	heat shock protein 90	2.77	943.23	2609.00
LDEC020664-RA	serine protease snake-like	2.79	43.16	120.48
LDEC017308-RA	phosphatidylethanolamine-binding protein	2.79	39.97	111.66
LDEC004990-RA	glutathione s-transferase	2.80	242.21	677.74
LDEC007894-RA	atp-citrate synthase	2.82	68.25	192.14
LDEC013673-RA	upf0160 protein	2.82	30.09	84.74
LDEC016525-RA	paxillin isoform x5	2.82	94.25	266.17
LDEC014600-RA	xanthine dehydrogenase oxidase	2.83	53.23	150.41
LDEC022719-RA	adenylate kinase	2.83	53.09	150.20
LDEC010683-RA	death-associated protein 1	2.83	48.53	137.38
LDEC023381-RA	---NA---	2.83	163.12	461.86
LDEC014065-RA	t-complex protein 1 subunit eta	2.83	35.60	100.81
LDEC012590-RA	4-coumarate-- ligase 1-like	2.84	59.08	167.86
LDEC000955-RA	nadh-ubiquinone oxidoreductase 39 kda subunit	2.85	73.67	210.04
LDEC000527-RA	pre-mrna-processing factor 19	2.86	30.51	87.23
LDEC005452-RA	adenosylhomocysteinase b	2.86	64.55	184.52
LDEC008067-RA	pyruvate dehydrogenase e1 component subunit mitochondrial	2.87	54.42	156.08
LDEC002421-RA	malate cytoplasmic	2.87	82.51	236.75
LDEC019162-RA	aminopeptidase -like	2.87	62.74	180.05
LDEC005882-RA	probable aconitate mitochondrial	2.87	372.54	1069.54
LDEC008758-RA	scavenger receptor class b member 1 isoform x2	2.88	28.85	83.03
LDEC017886-RA	ribosomal protein l4	2.89	707.15	2043.19
LDEC000483-RA	calcium-transporting atpase sarcoplasmic endoplasmic reticulum type isoform x1	2.89	680.53	1966.54
LDEC012430-RA	phytanoyl- peroxisomal-like	2.90	39.16	113.67
LDEC016968-RA	type i cytoskeletal 9 isoform x2	2.90	27.66	80.35
LDEC007070-RA	aldehyde mitochondrial	2.91	289.88	842.81
LDEC024177-RA	mitochondrial enolase superfamily member 1-like isoform x2	2.91	132.04	383.90
LDEC004427-RA	serine-arginine protein 55-like isoform x2	2.91	77.00	223.97

LDEC006952-RA	translocon-associated protein subunit gamma	2.91	74.95	218.05
LDEC005731-RA	thioredoxin domain-containing protein 17-like	2.91	73.96	215.37
LDEC018610-RA	caprin homolog	2.92	43.11	125.67
LDEC013444-RA	inorganic phosphate cotransporter isoform x1	2.94	134.18	393.87
LDEC007869-RA	lamin dm0-like isoform x1	2.95	33.75	99.42
LDEC014014-RA	porphobilinogen deaminase	2.96	37.55	111.05
LDEC020740-RA	atp synthase subunit mitochondrial-like	2.97	1101.12	3271.53
LDEC011628-RA	restin homolog	2.97	53.33	158.55
LDEC008164-RA	cytoplasmic a3	2.97	1503.65	4472.62
LDEC013655-RA	juvenile hormone esterase	2.98	27.00	80.33
LDEC011578-RA	46 kda fk506-binding nuclear protein	2.98	50.24	149.54
LDEC006148-RA	angiotensin-converting enzyme-like	2.98	35.03	104.42
LDEC024312-RA	40s ribosomal protein s2	2.99	31.99	95.54
LDEC004900-RA	superoxide dismutase	2.99	69.77	208.97
LDEC016761-RA	biotin--protein ligase isoform x1	3.00	40.73	122.03
LDEC000780-RA	protein takeout	3.01	1130.35	3401.16
LDEC002153-RA	glycerol-3-phosphate mitochondrial isoform x1	3.03	115.73	350.75
LDEC021796-RA	gamma-interferon-inducible lysosomal thiol reductase-like	3.04	462.70	1404.65
LDEC001608-RA	scavenger receptor class b member 1	3.04	53.57	162.77
LDEC008120-RA	endopolygalacturonase	3.04	60.41	183.79
LDEC010890-RA	cathepsin b-like proteinase	3.05	32.18	98.23
LDEC014750-RA	probable isocitrate dehydrogenase	3.06	73.67	225.45
LDEC015417-RA	pupal cuticle protein 36-like isoform x2	3.07	115.12	353.09
LDEC006988-RA	rna-binding protein squid isoform x3	3.07	204.57	628.74
LDEC014331-RA	PREDICTED: myophilin	3.08	86.22	265.36
LDEC002455-RA	39s ribosomal protein mitochondrial	3.08	33.70	103.78
LDEC012869-RA	nucleolysin tiar	3.09	37.07	114.44
LDEC013715-RA	nad mitochondrial	3.09	28.38	87.61
LDEC012234-RA	cellular fabp-like protein isoform 1 precursor	3.09	77.05	238.04
LDEC020299-RA	peptidyl-prolyl cis-trans isomerase 5	3.10	106.75	331.24
LDEC010871-RA	cathepsin b precursor	3.11	62.36	193.64
LDEC005442-RA	unknown	3.11	84.37	262.11
LDEC013601-RA	prostatic acid phosphatase	3.11	31.56	98.07
LDEC011014-RA	peroxisomal multifunctional enzyme type 2	3.13	146.68	458.81
LDEC015168-RA	peptidoglycan-recognition protein 2	3.13	23.38	73.30
LDEC009502-RA	midline fasciclin	3.14	106.66	335.10
LDEC002142-RA	y-box factor homolog isoform x1	3.15	479.67	1508.90
LDEC017300-RA	atpase family aaa domain-containing protein 3	3.15	34.22	107.92
LDEC002528-RA	acetyl- carboxylase isoform x3	3.17	41.68	131.97
LDEC006718-RA	insulin-like growth factor-binding protein complex acid labile subunit	3.17	31.23	98.97
LDEC007413-RA	heat shock protein 70	3.17	2204.57	6992.21

LDEC005923-RA	farnesoic acid o-methyltransferase-like protein	3.17	217.07	688.72
LDEC003616-RA	apolipoprotein li-ii precursor	3.18	340.98	1082.74
LDEC015841-RA	probable methylmalonate-semialdehyde dehydrogenase	3.19	79.28	252.77
LDEC011582-RA	tropomyosin 1	3.19	284.85	909.34
LDEC002665-RA	unc93-like protein	3.19	52.90	168.90
LDEC008333-RA	PREDICTED: uncharacterized protein C05D11.1-like	3.21	59.84	191.84
LDEC017682-RA	probable multidrug resistance-associated protein lethal 03659	3.22	84.60	272.24
LDEC008426-RA	transmembrane protein 53	3.22	35.98	115.84
LDEC007315-RA	oxygen-dependent coproporphyrinogen-iii oxidase	3.24	24.67	79.83
LDEC002666-RA	unc93-like protein	3.25	32.37	105.07
LDEC022212-RA	probable saccharopine dehydrogenase	3.25	28.14	91.39
LDEC024276-RA	acetyl- mitochondrial	3.25	22.86	74.29
LDEC021246-RA	nadh dehydrogenase	3.26	110.98	361.66
LDEC006147-RA	angiotensin-converting enzyme-like	3.26	31.75	103.50
LDEC004946-RA	af285759_1d- core domains	3.28	22.05	72.40
LDEC014599-RA	indole-3-acetaldehyde oxidase-like	3.30	43.58	143.70
LDEC006412-RA	prostaglandin reductase 1	3.30	22.29	73.51
LDEC021988-RA	mannose-1-phosphate guanyltransferase beta	3.30	38.93	128.49
LDEC006909-RA	endocuticle structural glycoprotein bd-2-like	3.30	63.74	210.44
LDEC015135-RA	retinaldehyde-binding protein 1	3.31	74.29	245.59
LDEC013817-RA	venom carboxylesterase-6	3.33	28.38	94.41
LDEC000077-RA	voltage-dependent anion channel	3.35	100.38	335.88
LDEC002529-RA	acetyl-coa carboxylase	3.35	93.25	312.29
LDEC001745-RA	apy_tabya ame: full=apyrase ame: full=atp-diphosphatase short=adpase ame: full=atp-diphosphohydrolase ame: full=adenosine diphosphatase ame: allergen=tab y 1 flags: precursor	3.36	26.14	87.76
LDEC003498-RA	beta-amyloid-like partial	3.36	27.47	92.44
LDEC015475-RA	sodium-coupled monocarboxylate transporter 1	3.37	77.95	262.37
LDEC003818-RA	alpha-n-acetylgalactosaminidase-like isoform x1	3.37	30.04	101.26
LDEC016736-RA	hypothetical protein KGM_03566	3.37	138.64	467.39
LDEC018287-RA	laminin subunit alpha	3.37	25.81	87.02
LDEC005079-RA	inorganic phosphate cotransporter	3.38	39.78	134.27
LDEC011802-RA	polypyrimidine tract-binding protein 1 isoform x3	3.39	31.23	105.97
LDEC016073-RA	fibulin 1 and	3.41	28.33	96.68
LDEC007619-RA	heat shock protein 90	3.41	185.70	633.88
LDEC017593-RA	voltage-dependent anion channel	3.42	206.90	706.64
LDEC010948-RA	hypothetical protein D910_00165	3.42	110.08	376.28
LDEC005049-RA	pyruvate dehydrogenase e1 component subunit mitochondrial	3.42	113.22	387.14
LDEC019996-RA	cartilage oligomeric matrix protein	3.44	75.62	259.94
LDEC014126-RA	translocon-associated protein subunit alpha	3.44	125.19	430.95

LDEC011920-RA	tho complex subunit 4	3.44	43.54	149.93
LDEC006115-RA	zinc finger rna-binding protein	3.46	93.25	322.49
LDEC010888-RA	cathepsin b	3.46	33.46	115.93
LDEC003241-RA	3-hydroxyacyl-coa dehyrogenase	3.47	74.43	258.60
LDEC002633-RA	glypican-6 isoform x1	3.50	20.82	72.84
LDEC016520-RA	dnaj homolog subfamily b member 11	3.50	36.08	126.32
LDEC006878-RA	inter-alpha-trypsin inhibitor heavy chain h4-like isoform x1	3.50	110.08	385.67
LDEC013717-RA	hydroxyacid oxidase 1	3.53	103.61	365.99
LDEC017194-RA	procollagen- -oxoglutarate 5-dioxygenase 3-like	3.55	22.43	79.66
LDEC003946-RA	very long-chain-fatty-acid-- ligase bubblegum isoform x1	3.57	88.69	316.66
LDEC010111-RA	rna-binding protein lark isoform x4	3.57	91.21	325.82
LDEC010875-RA	cathepsin b	3.58	31.23	111.78
LDEC012668-RA	probable phenylalanine--trna ligase alpha subunit	3.58	22.20	79.47
LDEC010996-RA	polyadenylate-binding protein 4-like	3.58	29.71	106.45
LDEC020611-RA	laminin subunit beta-1	3.58	118.11	423.24
LDEC007378-RA	short-chain dehydrogenease reductase	3.61	50.52	182.60
LDEC022336-RA	60 kda heat shock mitochondrial-like	3.62	100.91	365.12
LDEC008944-RA	aromatic-l-amino-acid decarboxylase-like	3.62	25.10	90.93
LDEC005399-RA	venom allergen 5-like	3.62	31.75	115.09
LDEC016638-RA	t-complex protein 1 subunit gamma	3.63	75.90	275.33
LDEC007358-RA	protein elys-like isoform x3	3.63	23.62	85.77
LDEC003254-RA	glycoside hydrolase family 28	3.65	402.15	1466.11
LDEC009415-RA	probable medium-chain specific acyl-mitochondrial	3.65	100.48	367.02
LDEC003862-RA	glycogen debranching enzyme-like	3.66	35.08	128.49
LDEC002531-RA	acetyl- carboxylase isoform x2	3.68	25.90	95.31
LDEC013883-RA	GD18104	3.69	19.39	71.51
LDEC015597-RA	serine-arginine protein 55 isoform x7	3.69	76.71	283.41
LDEC006128-RA	rna-binding protein squid isoform x3	3.70	166.92	618.15
LDEC020558-RA	glutathione s transferase e6	3.71	68.92	255.69
LDEC006881-RA	inter-alpha-trypsin inhibitor heavy chain h4-like isoform x1	3.71	16.59	61.61
LDEC007893-RA	atp-citrate synthase isoform x1	3.73	291.88	1088.61
LDEC011583-RA	tropomyosin- partial	3.73	231.42	864.27
LDEC010233-RA	acyl- synthetase short-chain family member mitochondrial	3.74	65.92	246.24
LDEC014489-RA	isovaleryl coenzyme a dehydrogenase	3.74	63.45	237.41
LDEC013633-RA	mitochondrial enolase superfamily member 1-like isoform x2	3.76	153.76	578.88
LDEC015119-RA	regucalcin-like isoform x1	3.78	69.63	263.27
LDEC020822-RA	muscle actin	3.78	5352.32	20252.10
LDEC011066-RA	RT01145p	3.80	25.00	95.01
LDEC018480-RA	probable enoyl- mitochondrial-like	3.81	251.19	957.75
LDEC000846-RA	fatty acid-binding protein 12-like	3.82	222.15	848.88
LDEC019595-RA	neurexin-4 isoform x1	3.83	18.06	69.12

LDEC002361-RA	agap002583-pa-like protein	3.84	80.04	307.73
LDEC007807-RA	la-related protein 6	3.86	170.73	659.82
LDEC002595-RA	acyl-coa thioesterase	3.87	21.44	82.94
LDEC007262-RA	insulin-like growth factor 2 mrna-binding protein 1 isoform x4	3.88	26.43	102.42
LDEC002058-RA	digestive cysteine protease intestain	3.88	33.75	130.88
LDEC007028-RA	probable multidrug resistance-associated protein lethal 03659	3.89	28.85	112.20
LDEC019589-RA	troponin t isoform x2	3.90	1259.54	4912.19
LDEC021669-RA	40s ribosomal protein s2	3.91	404.24	1578.86
LDEC001815-RA	cuticular protein rr-1 family	3.93	21.29	83.63
LDEC019546-RA	microtubule-associated protein futsch-like	3.93	34.74	136.48
LDEC013728-RA	basement membrane-specific heparan sulfate proteoglycan core protein isoform x8	3.93	124.62	490.04
LDEC015496-RA	integument esterase	3.94	25.62	100.83
LDEC014810-RA	small nuclear ribonucleoprotein-associated protein b	3.94	20.06	79.01
LDEC021576-RA	multidrug resistance protein 1a	3.94	19.20	75.69
LDEC000085-RA	spectrin beta chain isoform x4	3.95	323.39	1278.41
LDEC016690-RA	surfeit locus protein 4 homolog	3.96	70.25	278.05
LDEC012332-RA	cytochrome p450-like protein	3.97	28.42	112.93
LDEC016681-RA	triosephosphate isomerase	3.97	76.24	302.96
LDEC015972-RA	myosin-2 essential light chain isoform x2	3.98	37.60	149.70
LDEC018597-RA	monocarboxylate transporter 9-like	3.98	14.78	58.89
LDEC017672-RA	probable multidrug resistance-associated protein lethal 03659	3.99	15.64	62.34
LDEC021660-RA	gamma-glutamyltranspeptidase 1 isoform x2	3.99	24.48	97.77
LDEC009387-RA	eukaryotic translation initiation factor 4 gamma 3-like isoform x3	4.05	33.56	135.81
LDEC015088-RA	acyl- dehydrogenase	4.05	48.77	197.55
LDEC001686-RA	moesin ezrin radixin	4.06	61.50	249.42
LDEC016254-RA	myosin light chain 2	4.09	1050.36	4293.74
LDEC002059-RA	digestive cysteine proteinase intestain	4.09	3673.24	15032.00
LDEC012761-RA	seminal fluid partial	4.09	24.53	100.37
LDEC007396-RA	serine protease h3	4.11	45.68	187.74
LDEC010104-RA	protein singed	4.13	25.14	103.96
LDEC008865-RA	cuticular protein analogous to peritrophins 1-c precursor	4.14	32.94	136.27
LDEC007394-RA	mast cell protease 6	4.14	54.85	227.00
LDEC000699-RA	lipid storage droplets surface-binding protein 1 isoform x1	4.17	129.52	539.73
LDEC016324-RA	PREDICTED: uncharacterized protein LOC657683	4.17	29.18	121.74
LDEC002247-RA	eukaryotic translation initiation factor 2 subunit 1	4.17	24.38	101.79
LDEC005758-RA	diapause-associated transcript-2	4.18	133.65	558.93
LDEC017303-RA	---NA---	4.19	104.00	435.26
LDEC004703-RA	swi snf complex subunit smarcc2	4.19	28.23	118.23
LDEC008468-RA	---NA---	4.20	228.81	960.41

LDEC004140-RA	cyclin-dependent kinase 12 isoform x3	4.21	27.95	117.70
LDEC003271-RA	organic cation transporter protein	4.22	13.69	57.70
LDEC013825-RA	cytosolic non-specific dipeptidase	4.22	42.25	178.21
LDEC010980-RA	guanine nucleotide-binding protein g g g subunit beta-1	4.22	21.86	92.29
LDEC011144-RA	PREDICTED: uncharacterized protein LOC662719	4.23	16.92	71.53
LDEC010420-RA	24-dehydrocholesterol reductase	4.24	14.02	59.40
LDEC009737-RA	juvenile hormone binding protein partial	4.31	22.20	95.70
LDEC013201-RA	dnaj homolog subfamily c member 22	4.31	17.68	76.25
LDEC010880-RA	cathepsin b	4.32	135.27	584.90
LDEC002057-RA	digestive cysteine protease intestain	4.33	523.49	2269.10
LDEC014278-RA	beta-galactosidase-1-like protein 2	4.34	33.18	144.01
LDEC004416-RA	f-actin-capping protein subunit alpha	4.38	14.31	62.72
LDEC003945-RA	very long-chain-fatty-acid-- ligase bubblegum isoform x1	4.39	114.69	503.34
LDEC017604-RA	actin-interacting protein 1	4.42	20.34	89.93
LDEC009032-RA	protein transport protein sec61 subunit beta	4.44	45.58	202.45
LDEC006513-RA	10 kda heat shock mitochondrial	4.44	33.75	149.93
LDEC008263-RA	dna replication licensing factor mcm6	4.44	14.64	65.06
LDEC007709-RA	esterase b1-like	4.45	18.87	84.05
LDEC007078-RA	protein disulfide isomerase	4.46	244.21	1088.20
LDEC018788-RA	alanine--trna cytoplasmic	4.47	29.47	131.70
LDEC003148-RA	glucosidase 2 subunit beta	4.53	75.48	341.80
LDEC022268-RA	probable multidrug resistance-associated protein lethal 03659 isoform x1	4.54	17.40	78.99
LDEC021639-RA	nadh-ubiquinone oxidoreductase 75 kda subunit	4.55	43.11	196.36
LDEC010781-RA	isoform a	4.57	27.19	124.25
LDEC009388-RA	eukaryotic translation initiation factor 4 gamma 3-like isoform x3	4.57	150.81	689.58
LDEC010881-RA	cathepsin b	4.58	73.72	337.64
LDEC023680-RA	aconitate mitochondrial-like isoform x2	4.59	27.09	124.35
LDEC019872-RA	carboxypeptidase a-like	4.59	19.77	90.75
LDEC018713-RA	protein transport protein sec61 subunit gamma	4.59	15.78	72.43
LDEC001027-RA	translocon-associated protein subunit beta	4.61	36.41	168.02
LDEC022806-RA	importin subunit alpha-4	4.62	12.03	55.57
LDEC002048-RA	tp53-regulating kinase	4.65	26.33	122.53
LDEC014088-RA	udp-glucuronosyltransferase 2b9	4.67	52.90	246.97
LDEC016473-RA	cuticular protein analogous to peritrophins 3-e	4.68	57.99	271.50
LDEC006646-RA	major antigen-like	4.72	13.64	64.35
LDEC000239-RA	serine protease	4.73	74.48	352.55
LDEC013099-RA	isocitrate dehydrogenase	4.74	20.87	98.96
LDEC023888-RA	acyl-coa thioesterase	4.77	13.12	62.51
LDEC013118-RA	protein disulfide-isomerase	4.77	114.55	546.55
LDEC008359-RA	inorganic phosphate cotransporter	4.80	15.35	73.74

LDEC009998-RA	mdl2	4.80	26.05	125.15
LDEC002622-RA	elongation factor mitochondrial	4.81	17.54	84.30
LDEC001643-RA	luciferin-regenerating partial	4.82	16.35	78.74
LDEC013727-RA	basement membrane-specific heparan sulfate proteoglycan core protein isoform x3	4.84	21.48	104.05
LDEC006903-RA	peptidoglycan-recognition protein precursor	4.85	29.71	143.93
LDEC011415-RA	methylcrotonoyl- carboxylase subunit mitochondrial isoform x2	4.85	19.25	93.44
LDEC001026-RA	cysteine-rich with egf-like domain protein 2	4.89	15.11	73.91
LDEC020880-RA	protein yellow-like isoform x2	4.91	126.43	620.91
LDEC013730-RA	basement membrane-specific heparan sulfate proteoglycan core protein isoform x14	4.92	89.12	438.90
LDEC019515-RA	serine protease p98	4.95	13.07	64.65
LDEC009339-RA	PREDICTED: uncharacterized protein LOC663405 isoform X2	4.95	9.84	48.69
LDEC008881-RA	acetyl- mitochondrial	4.95	37.22	184.23
LDEC001118-RA	transketolase-like protein 2 isoform x1	4.96	245.97	1220.87
LDEC008159-RA	lysosomal aspartic protease	4.99	344.73	1720.30
LDEC004918-RA	succinate dehydrogenase	4.99	19.30	96.35
LDEC006153-RA	15-hydroxyprostaglandin dehydrogenase	5.00	9.89	49.38
LDEC012345-RA	dolichyl-diphosphooligosaccharide--protein glycosyltransferase subunit 2	5.00	85.70	428.82
LDEC003725-RA	pathogenesis-related protein 5	5.02	43.77	219.81
LDEC005648-RA	glucose dehydrogenase	5.02	18.49	92.88
LDEC015100-RA	rrna 2 -o-methyltransferase fibrillarin	5.04	18.44	92.88
LDEC004433-RA	oligosaccharyl transferase	5.06	53.57	271.00
LDEC000730-RA	mitochondrial amidoxime reducing component 2	5.06	17.92	90.68
LDEC012651-RA	PREDICTED: uncharacterized protein LOC103312147	5.07	16.49	83.55
LDEC009536-RA	protein ovo isoform x2	5.08	19.44	98.69
LDEC022234-RA	serine protease easter	5.08	23.00	116.97
LDEC010015-RA	counting factor associated protein d-like	5.09	123.67	628.95
LDEC006357-RA	glycoside hydrolase family protein 48	5.11	667.27	3410.19
LDEC007638-RA	ecdysone 20-monooxygenase isoform x2	5.16	16.16	83.45
LDEC012661-RA	regucalcin-like isoform x2	5.17	12.83	66.36
LDEC009738-RA	juvenile hormone binding protein partial	5.18	10.60	54.92
LDEC016646-RA	protein ergic-53	5.18	36.12	187.28
LDEC010356-RA	synaptic vesicle glycoprotein 2c-like isoform x1	5.19	32.89	170.64
LDEC003256-RA	glycoside hydrolase family 28	5.19	88.74	460.63
LDEC010000-RA	PREDICTED: uncharacterized protein LOC655909 isoform X2	5.21	9.17	47.83
LDEC001999-RA	isoform a	5.23	40.59	212.26
LDEC009157-RA	heat shock cognate 70	5.24	192.88	1010.23
LDEC001314-RA	isoform b	5.25	28.47	149.43
LDEC018549-RA	hexosaminidase 1 isoform x1	5.27	24.19	127.49
LDEC003144-RA	glutamate--cysteine ligase catalytic subunit	5.28	33.32	175.85

LDEC005652-RA	mucin- partial	5.30	58.51	309.95
LDEC016118-RA	hypoxia up-regulated protein 1 isoform x1	5.32	56.66	301.47
LDEC022012-RA	40s ribosomal protein sa	5.35	49.57	265.27
LDEC005903-RA	fatty acyl- reductase cg5065-like	5.36	34.55	185.17
LDEC010381-RA	heat shock protein 90	5.39	19.44	104.80
LDEC000731-RA	tyrosine-protein phosphatase 10d isoform x1	5.40	221.96	1198.54
LDEC005018-RA	ubiquitin carboxyl-terminal hydrolase 10	5.41	10.93	59.12
LDEC006352-RA	alpha- sarcomeric	5.42	72.82	394.56
LDEC018831-RA	odorant binding protein	5.44	13.31	72.36
LDEC020510-RA	histone h2a-like	5.45	19.34	105.34
LDEC020000-RA	12 kda fk506-binding protein	5.54	48.91	270.82
LDEC015842-RA	probable methylmalonate-semialdehyde dehydrogenase	5.55	22.86	126.84
LDEC005684-RA	cathepsin l precursor	5.56	28.47	158.24
LDEC003143-RA	glutamate--cysteine ligase catalytic subunit	5.56	9.70	53.90
LDEC024655-RA	adipocyte plasma membrane-associated	5.57	10.27	57.16
LDEC007616-RA	atp-binding cassette sub-family d member 3	5.58	34.84	194.35
LDEC003910-RA	peroxisomal acyl-coenzyme a oxidase 3	5.60	27.66	154.83
LDEC016367-RA	agap004199-pa-like protein	5.61	11.45	64.27
LDEC018333-RA	serine protease	5.70	29.90	170.41
LDEC015477-RA	sodium-coupled monocarboxylate transporter 1	5.71	35.50	202.76
LDEC000757-RA	endo-beta- -glucanase	5.71	87.50	499.74
LDEC003331-RA	l-lactate dehydrogenase isoform x2	5.73	124.67	714.07
LDEC009538-RA	calumenin	5.74	66.49	381.60
LDEC012852-RA	probable chitinase 2 isoform x2	5.79	26.09	151.02
LDEC014791-RA	glycoside hydrolase family 45 protein	5.80	73.72	427.67
LDEC014789-RA	endo-beta- -glucanase	5.81	255.61	1485.83
LDEC000652-RA	odorant binding partial	5.84	11.55	67.47
LDEC011408-RA	udp-glucose:glycoprotein glucosyltransferase	5.88	14.12	83.03
LDEC016658-RA	nadh dehydrogenase	5.89	19.58	115.32
LDEC012816-RA	arylphorin-like hexameric storage protein	5.94	10.22	60.65
LDEC021583-RA	ump-cmp kinase	5.99	19.68	117.77
LDEC017157-RA	hypothetical protein TcasGA2_TC015372	6.00	55.42	332.32
LDEC011092-RA	udp-glucuronosyltransferase 2b7	6.01	13.83	83.11
LDEC020078-RA	venom serine carboxypeptidase	6.02	39.02	234.80
LDEC008876-RA	antichymotrypsin-2-like isoform x3	6.07	9.84	59.71
LDEC012841-RA	odorant-binding protein 17	6.10	89.93	548.32
LDEC023294-RA	sphingomyelin phosphodiesterase-like	6.14	8.22	50.49
LDEC004666-RA	2-hydroxyacylsphingosine 1-beta-galactosyltransferase-like	6.15	29.18	179.57
LDEC001987-RA	upstream activation factor subunit spp27	6.16	97.15	598.35
LDEC016089-RA	collagen alpha- chain	6.26	406.81	2547.63

LDEC007617-RA	atp-binding cassette sub-family d member 3	6.27	7.32	45.88
LDEC001223-RA	glycosyl hydrolase	6.29	69.35	436.16
LDEC013729-RA	basement membrane-specific heparan sulfate proteoglycan core protein isoform x6	6.30	68.59	432.04
LDEC001282-RA	protein arginine n-methyltransferase 1 isoform x1	6.32	31.13	196.76
LDEC008648-RA	agap005332-pc-like protein	6.33	35.27	223.11
LDEC019972-RA	gamma-interferon-inducible lysosomal thiol reductase-like	6.36	24.91	158.32
LDEC004034-RA	acetyl-coenzyme a synthetase	6.37	15.92	101.37
LDEC011312-RA	hypothetical protein YQE_05172, partial	6.45	1519.81	9804.66
LDEC009332-RA	probable cytochrome p450 mitochondrial isoform x1	6.45	6.46	41.72
LDEC024072-RA	glycoside hydrolase family 45 protein	6.55	43.82	287.25
LDEC015476-RA	sodium-coupled monocarboxylate transporter 1	6.56	15.49	101.58
LDEC013370-RA	kda midgut protein	6.58	8.13	53.46
LDEC010057-RA	proton-coupled amino acid transporter 4-like	6.61	11.12	73.47
LDEC009158-RA	heat shock protein 70	6.63	189.36	1255.59
LDEC017994-RA	pupal cuticle protein 20	6.65	15.73	104.57
LDEC006832-RA	cell wall protein dan4	6.68	12.36	82.49
LDEC017589-RA	93 kda serpin	6.73	6.94	46.68
LDEC012853-RA	midgut chitinase	6.78	13.17	89.30
LDEC010352-RA	methylthioribose-1-phosphate isomerase	6.81	7.08	48.19
LDEC018086-RA	collagen alpha-2 chain-like	6.87	111.36	764.74
LDEC003972-RA	myofilin variant a	6.90	193.64	1336.78
LDEC016032-RA	cytochrome p450 9z4	6.94	56.80	394.02
LDEC019995-RA	cartilage oligomeric matrix protein	7.01	34.89	244.55
LDEC014168-RA	aminopeptidase n	7.02	20.01	140.54
LDEC004180-RA	serine-pyruvate mitochondrial	7.07	5.75	40.64
LDEC005004-RA	h aca ribonucleoprotein complex subunit 1-like	7.08	5.94	42.06
LDEC003766-RA	gustatory receptor candidate 59	7.08	6.61	46.78
LDEC004736-RA	glucose dehydrogenase	7.14	6.08	43.44
LDEC013628-RA	c-terminal-binding protein isoform x3	7.17	5.70	40.89
LDEC013766-RA	pancreatic triacylglycerol lipase-like	7.19	6.08	43.73
LDEC010056-RA	proton-coupled amino acid transporter 1	7.22	6.37	46.01
LDEC014146-RA	z9 acyl- desaturase b	7.29	92.40	673.72
LDEC011160-RA	juvenile hormone binding protein partial	7.32	507.57	3712.94
LDEC008797-RA	#NAME?	7.32	10.65	77.93
LDEC003785-RA	gamma-interferon-inducible lysosomal thiol reductase-like	7.36	189.26	1392.56
LDEC013281-RA	odorant-binding protein 5	7.37	34.27	252.60
LDEC020469-RA	peptidyl-prolyl cis-trans isomerase fkbp14- partial	7.37	48.67	358.86
LDEC004606-RA	ribonucleoside-diphosphate reductase large subunit	7.38	14.12	104.17
LDEC000773-RA	histone	7.40	21.53	159.38

LDEC013013-RA	venom metalloproteinase 3 isoform x2	7.41	7.51	55.65
LDEC018741-RA	heat shock 70 kda protein cognate 4	7.42	8.46	62.81
LDEC021558-RA	venom acid phosphatase acph-1-like	7.43	11.55	85.85
LDEC002052-RA	digestive cysteine protease intestain	7.44	14.69	109.21
LDEC001337-RA	probable serine threonine-protein kinase nek3 isoform x1	7.45	7.98	59.52
LDEC003087-RA	citrate synthase	7.61	359.51	2735.63
LDEC012851-RA	hydroxyacyl-coenzyme a mitochondrial	7.62	39.50	301.02
LDEC021311-RA	pacifastin-like protease inhibitor cvp4	7.62	16.73	127.55
LDEC012334-RA	cytochrome p450 4c3-like	7.67	11.55	88.53
LDEC003786-RA	gamma-interferon-inducible lysosomal thiol reductase-like	7.72	83.37	643.84
LDEC019877-RA	aldo-keto reductase	7.73	13.26	102.48
LDEC001224-RA	glycosyl hydrolase	7.74	23.57	182.37
LDEC012445-RA	low quality protein: water dikinase	7.78	40.40	314.29
LDEC005713-RA	93 kda serpin	7.80	18.39	143.43
LDEC020168-RA	ero1-like protein	7.88	8.60	67.80
LDEC017191-RA	procollagen- -oxoglutarate 5-dioxygenase 3	8.01	20.44	163.61
LDEC010285-RA	fatty acid synthase	8.04	175.96	1414.60
LDEC006127-RA	---NA---	8.12	8.89	72.13
LDEC007021-RA	agap006497-pa-like protein	8.12	128.71	1045.43
LDEC024465-RA	myosin regulatory light chain 2	8.15	78.19	637.39
LDEC017465-RA	protein yellow-like	8.16	4.61	37.64
LDEC013653-RA	alanine--glyoxylate aminotransferase 2-like	8.19	58.37	478.30
LDEC014462-RA	probable salivary secreted peptide	8.21	65.26	535.65
LDEC023039-RA	esterase	8.23	9.93	81.73
LDEC017189-RA	serine threonine-protein kinase polo	8.24	8.08	66.55
LDEC012058-RA	udp-glucuronosyltransferase 2b20-like	8.35	13.69	114.30
LDEC001143-RA	protein takeout-like	8.36	19.96	166.83
LDEC010734-RA	apolipoprotein d-like	8.40	51.71	434.49
LDEC019998-RA	---NA---	8.41	7.65	64.37
LDEC004611-RA	ribonucleoside-diphosphate reductase large subunit-like	8.44	6.94	58.54
LDEC018548-RA	hexosaminidase 1 isoform x1	8.48	11.79	99.95
LDEC007682-RA	PREDICTED: uncharacterized protein LOC103314279	8.49	5.09	43.17
LDEC007249-RA	agap006502-pa-like protein	8.63	4.28	36.93
LDEC012287-RA	pathogenesis-related protein 5	8.69	5.89	51.24
LDEC001225-RA	ornithine decarboxylase 1-like	8.72	52.38	456.51
LDEC014453-RA	udp-glucuronosyltransferase 2b16	8.77	10.31	90.50
LDEC009230-RA	polyadenylate-binding protein 1-like isoform 3	8.79	6.42	56.40
LDEC004242-RA	PREDICTED: uncharacterized protein LOC103314372	8.88	40.88	362.79
LDEC000262-RA	udp-glucuronosyltransferase 2c1-like	8.91	12.31	109.72
LDEC006875-RA	inter-alpha-trypsin inhibitor heavy chain h4-like isoform x1	8.92	7.46	66.59
LDEC017984-RA	basement membrane-specific heparan	8.96	7.98	71.51

	sulfate proteoglycan core protein			
LDEC009663-RA	heat shock protein 70	9.01	22.34	201.23
LDEC014788-RA	endo-beta- -glucanase	9.01	9.74	87.78
LDEC011117-RA	actin	9.07	88.60	803.45
LDEC009735-RA	juvenile hormone binding protein partial	9.18	368.26	3379.63
LDEC006784-RA	zinc carboxypeptidase	9.28	8.41	78.07
LDEC021797-RA	gamma-interferon-inducible lysosomal thiol reductase-like	9.36	56.13	525.13
LDEC011636-RA	lanb2	9.46	4.42	41.83
LDEC018087-RA	collagen alpha-5 chain	9.50	61.31	582.66
LDEC018247-RA	probable c-5 sterol desaturase	9.73	5.28	51.34
LDEC008919-RA	ribonucleoside-diphosphate reductase subunit m2 b	9.78	8.60	84.12
LDEC012711-RA	fk506-binding protein 2 isoform x1	9.84	9.74	95.91
LDEC009144-RA	ac1147-like partial	9.88	3.80	37.58
LDEC003222-RA	c-type lectin 5	10.00	63.88	638.84
LDEC017031-RA	isoform a	10.05	11.36	114.19
LDEC006154-RA	15-hydroxyprostaglandin dehydrogenase	10.15	3.66	37.14
LDEC003963-RA	atp-binding cassette sub-family b member mitochondrial	10.15	58.79	596.61
LDEC003829-RA	protein transport protein sec61 subunit alpha isoform 2	10.23	105.80	1082.51
LDEC000535-RA	lysyl oxidase homolog 3	10.62	4.56	48.46
LDEC003431-RA	flexible cuticle protein 12-like	10.64	6.08	64.73
LDEC013647-RA	facilitated trehalose transporter tret1-2 homolog	10.79	23.15	249.76
LDEC010984-RA	trypsin 7	10.83	31.18	337.68
LDEC014890-RA	tubulointerstitial nephritis	10.88	12.69	138.01
LDEC016562-RA	---NA---	11.09	160.51	1780.72
LDEC006874-RA	inter-alpha-trypsin inhibitor heavy chain h4-like isoform x2	11.15	18.63	207.74
LDEC002263-RA	ef-hand calcium-binding domain-containing protein 1	11.36	88.26	1002.28
LDEC013887-RA	pancreatic triacylglycerol lipase-like	11.36	3.37	38.34
LDEC004919-RA	serine protease persephone-like	11.83	6.46	76.48
LDEC017855-RA	alpha-n-acetylgalactosaminidase-like isoform x2	11.91	12.55	149.41
LDEC014889-RA	uncharacterized peptidase c1-like protein	11.97	40.40	483.61
LDEC006944-RA	---NA---	11.97	18.49	221.37
LDEC009180-RA	lysozyme i-2	12.04	15.59	187.75
LDEC010103-RA	protein singed	12.10	5.85	70.71
LDEC018935-RA	heat shock 70 kda protein cognate 3 isoform x1	12.38	208.61	2582.96
LDEC009006-RA	protein takeout	12.75	47.72	608.62
LDEC007347-RA	protein takeout	12.93	6.42	82.94
LDEC015759-RA	chitinase 5 precursor	13.04	28.95	377.45
LDEC012233-RA	neo-calmodulin-like isoform x2	13.08	2.99	39.17
LDEC007822-RA	---NA---	13.12	13.31	174.59
LDEC014647-RA	peroxidase-like isoform 1	13.22	2.47	32.67

LDEC004093-RA	coleoptericin a	13.27	18.25	242.23
LDEC015142-RA	mitochondrial amidoxime reducing component 2	13.54	3.28	44.40
LDEC019760-RA	membrane-bound alkaline phosphatase	13.62	5.94	80.90
LDEC005370-RA	mucin 12ea	13.70	44.35	607.43
LDEC007593-RA	blackjack	13.85	53.71	743.78
LDEC013203-RA	collagen alpha-1 chain	13.92	16.40	228.28
LDEC003449-RA	larval cuticle protein lcp-17-like	13.96	2.57	35.83
LDEC005371-RA	serine protease h164	14.00	47.15	659.98
LDEC007614-RA	low quality protein: calreticulin-like	14.70	262.93	3864.38
LDEC020736-RA	40s ribosomal protein s2	14.76	2.66	39.28
LDEC002286-RA	peroxidasin homolog	15.48	9.98	154.47
LDEC017853-RA	neutral alpha-glucosidase ab	15.49	11.98	185.55
LDEC001619-RA	synaptic vesicle glycoprotein 2b-like	15.80	8.18	129.17
LDEC013137-RA	laminin subunit gamma-1 isoform x2	15.89	5.32	84.60
LDEC015748-RA	chymotrypsin inhibitor-like	15.90	15.64	248.62
LDEC004033-RA	acetyl coenzyme a isoform b	16.12	3.42	55.17
LDEC000706-RA	glucose dehydrogenase	16.18	6.27	101.50
LDEC019636-RA	mesencephalic astrocyte-derived neurotrophic factor homolog	16.28	19.11	311.09
LDEC007780-RA	agap003453-pa-like protein	16.32	3.33	54.31
LDEC008295-RA	pheromone-binding protein 3-like	16.71	2.42	40.51
LDEC014197-RA	larval cuticle protein a2b-like	16.80	17.63	296.29
LDEC005338-RA	isoform a	16.85	2.19	36.85
LDEC021795-RA	---NA---	16.92	20.30	343.35
LDEC001221-RA	glycosyl hydrolase	16.98	3.14	53.27
LDEC005842-RA	juvenile hormone acid o-methyltransferase	17.05	2.95	50.24
LDEC012088-RA	prostatic acid phosphatase	17.30	19.91	344.60
LDEC000238-RA	serine protease	18.25	64.02	1168.59
LDEC022867-RA	venom acid phosphatase acph-1-like	18.50	9.46	174.97
LDEC018931-RA	antichymotrypsin-2-like isoform x4	18.81	40.35	759.01
LDEC020824-RA	endonuclease and reverse transcriptase-like protein	18.92	6.99	132.18
LDEC020340-RA	aldo-keto reductase	19.01	23.57	448.14
LDEC020338-RA	aldo-keto reductase family 4 member c9-like	19.53	4.61	90.03
LDEC015973-RA	cuticle protein 1	19.59	4.71	92.17
LDEC014836-RA	facilitated trehalose transporter tret1	19.64	2.23	43.86
LDEC022836-RA	fatty acyl- reductase cg5065 isoform x2	19.85	3.14	62.28
LDEC000211-RA	glutathione s-transferase epsilon	20.11	6.56	131.92
LDEC011968-RA	kynurenine--oxoglutarate transaminase 3 isoform x2	20.85	21.01	438.00
LDEC000777-RA	craniofacial development protein 2-like	21.81	13.93	303.67
LDEC020810-RA	esterase	21.81	13.69	298.61
LDEC005334-RA	leukocyte elastase inhibitor	22.03	1.62	35.60
LDEC001030-RA	protein disulfide-isomerase a3	22.05	74.00	1631.85

LDEC019922-RA	sodium-coupled monocarboxylate transporter 2-like	22.79	1.43	32.50
LDEC007042-RA	cathepsin l	22.86	11.64	266.24
LDEC022926-RA	b1 protein	22.91	8.89	203.66
LDEC023585-RA	PREDICTED: uncharacterized protein LOC103578083 isoform X1	23.43	5.13	120.28
LDEC005691-RA	cathepsin l-like proteinase	23.45	28.23	661.95
LDEC002556-RA	juvenile hormone partial	23.55	12.22	287.69
LDEC017746-RA	tetratricopeptide repeat protein 39b-like	23.60	3.04	71.78
LDEC017466-RA	protein yellow-like	23.71	2.42	57.47
LDEC003567-RA	beta-galactosidase-1-like protein 2	23.81	1.38	32.83
LDEC002561-RA	chymotrypsin-c-like isoform x1	24.00	6.23	149.43
LDEC002547-RA	---NA---	24.39	5.04	122.87
LDEC003116-RA	esterase	24.62	3.94	97.14
LDEC006945-RA	ccr4-not transcription complex subunit partial	24.64	1.19	29.28
LDEC009736-RA	juvenile hormone binding protein partial	24.74	4.47	110.53
LDEC008133-RA	prostatic acid phosphatase-like	24.79	6.80	168.52
LDEC005388-RA	adult cuticle protein 1-like	24.83	6.04	149.89
LDEC002959-RA	c-type lectin galactose-binding isoform-like	26.27	7.75	203.51
LDEC004031-RA	abp1_ripcl ame: full=probable antibacterial peptide polyprotein	26.57	5.75	152.80
LDEC012089-RA	prostatic acid phosphatase-like	27.25	12.31	335.40
LDEC004210-RA	protein disulfide-isomerase a6	27.80	16.68	463.85
LDEC003042-RA	protein disulfide-isomerase a3	28.07	1.90	53.37
LDEC004852-RA	inorganic phosphate cotransporter	28.08	1.33	37.37
LDEC004176-RA	PREDICTED: uncharacterized protein LOC103312569	28.16	2.95	82.97
LDEC002233-RA	glucose dehydrogenase	30.10	0.95	28.61
LDEC008132-RA	prostatic acid phosphatase	30.31	8.65	262.20
LDEC000024-RA	hypothetical protein YQE_02064, partial [Dendroctonus ponderosae]	30.32	1.47	44.67
LDEC010451-RA	gb12811-like partial	30.35	4.94	150.04
LDEC015349-RA	ubiquitin-like protein partial	30.84	50.38	1553.84
LDEC018796-RA	vanin-like protein 1	30.90	1.05	32.31
LDEC001704-RA	collagen alpha-1 chain-like	30.91	1.28	39.67
LDEC004625-RA	lysozyme precursor	31.68	2.42	76.78
LDEC009269-RA	venom acid phosphatase acph-1	32.09	4.71	151.02
LDEC008874-RA	cec2 protein	32.35	0.95	30.76
LDEC009863-RA	inorganic phosphate cotransporter	33.15	2.23	74.06
LDEC023183-RA	jerky protein homolog-like	33.33	2.09	69.69
LDEC007043-RA	cathepsin l	35.57	3.75	133.54
LDEC002232-RA	glucose dehydrogenase	36.65	3.85	141.09
LDEC015018-RA	chitinase 5 isoform x1	36.67	1.28	47.06
LDEC001398-RA	sortilin-related receptor-like	37.12	3.33	123.50
LDEC012588-RA	AGAP006960-PA	37.22	0.90	33.61
LDEC003840-RA	isoform c	39.39	1.81	71.15

LDEC013186-RA	ejaculatory bulb-specific protein 3	39.67	57.51	2281.73
LDEC018417-RA	odorant binding protein 8	40.33	5.94	239.61
LDEC009115-RA	cathepsin l precursor	40.37	1.57	63.31
LDEC002557-RA	juvenile hormone partial	40.85	7.51	306.75
LDEC023414-RA	agap011197-pa-like protein	40.96	50.81	2080.94
LDEC009913-RA	hypothetical protein L798_04546	41.35	1.00	41.28
LDEC006653-RA	peptidoglycan-recognition protein s2	42.47	1.43	60.55
LDEC005376-RA	serine protease gd-like isoform x2	43.43	1.43	61.93
LDEC015348-RA	agap011225-pa-like protein	43.61	36.22	1579.34
LDEC016584-RA	odorant binding partial	44.85	1.14	51.16
LDEC022969-RA	eukaryotic translation initiation factor 3 subunit a	46.32	1.76	81.46
LDEC009116-RA	cathepsin l	46.44	0.81	37.52
LDEC005692-RA	cathepsin l-like proteinase	46.76	6.27	293.40
LDEC006706-RA	lipase 3	47.82	2.57	122.74
LDEC004092-RA	coleoptericin a	47.98	3.33	159.62
LDEC007327-RA	hypothetical protein D910_06903	49.00	0.81	39.59
LDEC001698-RA	collagen alpha-1 chain-like	50.19	2.33	116.89
LDEC003389-RA	PREDICTED: uncharacterized protein LOC656585	51.55	1.33	68.60
LDEC006942-RA	hypothetical protein EAI_07617	58.80	2.76	162.10
LDEC008092-RA	serine protease	59.47	1.66	98.94
LDEC018149-RA	collagen alpha-1 chain	60.49	1.14	69.00
LDEC009001-RA	hypothetical protein DAPPUDRAFT_105533	61.02	2.90	176.93
LDEC013494-RA	protein takeout-like	62.86	39.69	2494.91
LDEC006895-RA	cuticle protein cp5	63.07	5.70	359.72
LDEC005075-RA	flocculation protein flo11	74.07	0.67	49.29
LDEC011305-RA	bgbp_tenmo ame: full=beta- -glucan-binding protein short=bgbp ame: full=beta- -glucan recognition protein short= flags: precursor	78.77	0.38	29.95
LDEC005723-RA	attacin-like immune protein	78.89	2.19	172.48
LDEC006894-RA	cuticle protein cp5	83.91	3.04	255.25
LDEC006705-RA	lipase 3	84.04	2.95	247.66
LDEC015347-RA	prostaglandin e synthase 3	85.59	3.28	280.71
LDEC016508-RA	skin secretory protein xp2-like	93.85	0.43	40.15
LDEC019184-RA	mast cell protease 6	94.46	1.43	134.69
LDEC009341-RA	microtubule-associated protein futsch-like isoform x12	96.21	0.38	36.58
LDEC023464-RA	agap012703-pa-like protein	98.83	0.67	65.77
LDEC016697-RA	PREDICTED: uncharacterized protein LOC657400 isoform X1	105.63	0.52	55.23
LDEC012045-RA	encapsulation-relating protein	108.78	0.81	87.90
LDEC014079-RA	antifreeze protein maxi	120.91	0.52	63.22
LDEC014481-RA	serine protease	127.56	0.62	78.82
LDEC019869-RA	glucose dehydrogenase	133.33	1.76	234.47
LDEC000705-RA	antifreeze protein maxi-like	144.74	0.52	75.67

LDEC003432-RA	larval cuticle protein 8-like	156.50	0.29	44.63
LDEC020732-RA	zinc finger protein 512b	166.37	0.29	47.45
LDEC005268-RA	fibril-forming collagen alpha chain-like	169.18	3.94	667.39
LDEC023304-RA	zinc carboxypeptidase	172.88	0.24	41.08
LDEC022265-RA	lactase-phlorizin hydrolase	173.77	0.29	49.55
LDEC005685-RA	cathepsin l	180.48	3.37	609.06
LDEC011790-RA	ice-structuring glycoprotein	193.52	0.38	73.58
LDEC003448-RA	larval cuticle protein lcp-17-like	204.71	0.95	194.60
LDEC017176-RA	salivary c-type lectin	212.91	0.48	101.20
LDEC018962-RA	low quality protein: cell wall protein tir4-like	216.50	0.14	30.87
LDEC007343-RA	PREDICTED: LOW QUALITY PROTEIN: uncharacterized protein LOC101888582	218.07	4.33	943.18
LDEC008679-RA	laccase 1 isoform x1	218.57	10.79	2358.17
LDEC009102-RA	cytochrome p450 6k1	222.05	2.90	643.80
LDEC006672-RA	PREDICTED: uncharacterized protein LOC100142033	222.39	7.51	1670.11
LDEC009910-RA	hypothetical protein L798_04546	229.66	0.38	87.32
LDEC006900-RA	larval cuticle protein lcp-30	237.33	1.71	406.09
LDEC002268-RA	PREDICTED: uncharacterized protein LOC655532 isoform X2	250.37	0.71	178.50
LDEC005728-RA	attacin-like immune protein	256.33	3.61	925.92
LDEC009497-RA	hypothetical antimicrobial peptide	268.26	1.00	267.76
LDEC012341-RA	high molecular weight subunit dx5-like	275.87	0.57	157.34
LDEC002958-RA	lectin subunit alpha-like	283.77	0.33	94.41
LDEC013001-RA	cuticular protein	293.91	0.10	27.94
LDEC013822-RA	hypothetical protein D910_11774	3058	0.00	30.58
LDEC013797-RA	cuticular protein cpr2	308.16	0.14	43.94
LDEC004299-RA	solute carrier family 25 member 35	336.35	1.05	351.71
LDEC007681-RA	PREDICTED: uncharacterized protein LOC103314280	341.32	0.24	81.12
LDEC007252-RA	agap006502-pa-like protein	345.23	0.33	114.86
LDEC009342-RA	hormone receptor in 46-like protein	349.01	0.14	49.77
LDEC005727-RA	attacin-like immune protein	349.60	3.71	1296.06
LDEC019613-RA	PREDICTED: uncharacterized protein LOC660742	435.42	0.10	41.39
LDEC022497-RA	esterase	4588	0.00	45.88
LDEC007473-RA	cytochrome p450 307a1	473.59	0.14	67.53
LDEC000781-RA	protein takeout-like	508.67	0.14	72.53
LDEC011289-RA	cuticle protein 65-like	526.14	0.10	50.01
LDEC024311-RA	inactive pancreatic lipase-related protein 1	5277	0.00	52.77
LDEC012734-RA	digestive cysteine proteinase intestain	5569	0.00	55.69
LDEC023221-RA	cytochrome p450	564.44	0.05	26.83
LDEC001911-RA	larval cuticle protein lcp-30	5885	0.00	58.85
LDEC000908-RA	ka261_mesma ame: full=potassium channel blocker alpha-ktx ame: full=neurotoxin 86 flags: precursor	593.13	0.57	338.30
LDEC003783-RA	proclotting enzyme	706.29	0.57	402.84

LDEC003889-RA	chitinase 10 precursor	707.16	0.24	168.06
LDEC003760-RA	apolipoprotein d-like	7632	0.00	76.32
LDEC023170-RA	cytosolic carboxypeptidase 6	783.30	1.24	967.98
LDEC003447-RA	larval cuticle protein lcp-17-like	785.92	2.14	1680.96
LDEC003999-RA	gly-rich protein	8150	0.00	81.50
LDEC005757-RA	diapause-associated transcript-2	8510	0.00	85.10
LDEC012084-RA	prostatic acid phosphatase	912.17	0.19	173.42
LDEC006047-RA	---NA---	951.32	0.48	452.16
LDEC005687-RA	digestive cysteine protease intestain	14021	0.00	140.21
LDEC013553-RA	larval pupal cuticle protein h1c	1432.50	0.48	680.87
LDEC024457-RA	---NA---	1561.88	0.05	74.24
LDEC023632-RA	adfb like protein	1624.51	0.14	231.64
LDEC003996-RA	glycine-rich cell wall structural	1631.60	3.66	5971.33
LDEC006048-RA	hypothetical protein IscW_ISCW024931	1752.08	0.19	333.10
LDEC014792-RA	---NA---	18078	0.00	180.78
LDEC011288-RA	---NA---	2131.84	0.62	1317.24
LDEC006052-RA	hypothetical protein IscW_ISCW024931	2475.29	0.33	823.55
LDEC014795-RA	maltase 2-like	2558.32	0.19	486.38
LDEC022542-RA	plasma membrane calcium-transporting atpase	2573.44	0.05	122.31
LDEC022543-RA	---NA---	2929.57	0.14	417.72
LDEC009004-RA	---NA---	3137.07	0.10	298.21
LDEC013799-RA	endocuticle structural glycoprotein bd-1-like	3189.42	0.95	3031.84
LDEC000795-RA	pro-phenol oxidase subunit 2	32072	0.00	320.72
LDEC017590-RA	serine protease s1a-1	3832.54	0.05	182.16
LDEC021944-RA	cuticle protein	5191.53	0.43	2220.77
LDEC006050-RA	---NA---	5442.40	0.19	1034.70
LDEC012842-RA	b1 protein	74464	0.00	744.64
LDEC013552-RA	larval pupal cuticle protein h1c	7943.67	0.05	377.56
LDEC006045-RA	chorion protein s38	8577.85	0.05	407.70
LDEC011657-RA	cuticular protein 27a	11001.23	0.24	2614.43
LDEC011656-RA	larval cuticle protein 8-like	11755.44	0.10	1117.47
LDEC011658-RA	flexible cuticle protein 12	17032.66	0.24	4047.79
LDEC005725-RA	attacin-like immune protein	19201.39	0.05	912.64
LDEC001912-RA	larval cuticle protein lcp-30	46653.66	0.10	4434.87
LDEC014547-RA	hypothetical protein TcasGA2_TC002836	63004.03	0.29	17967.38
LDEC014544-RA	---NA---	2493196	0.00	24931.96

Table 8S. Significantly differentially expressed genes, after Bonferroni correction, in an adult female and larval *Leptinotarsa decemlineata.*

Feature ID	Gene name	Experiment - Fold Change (normalized values)	Baggerley's test: group 2 vs group 1 Bonferroni adjusted *p*-values	Adult female - Normalized expression values	Larvae (GE) - Normalized expression values
LDEC003837-RA	endonuclease and reverse transcriptase-like protein	-5872.50	0.00	5872.58	0.08
LDEC021419-RA	---NA---	-3877.18	0.00	3877.18	0.00
LDEC006905-RA	endocuticle structural glycoprotein bd-	-1281.24	0.00	1281.28	0.04
LDEC013181-RA	chemosensory protein 8	-537.74	0.00	537.76	0.02
LDEC024230-RA	cuticular protein 13	-1040.48	0.00	1040.52	0.04
LDEC003957-RA	hypothetical protein D910_10187	-1267.44	0.00	1267.52	0.08
LDEC013548-RA	glycine-rich protein	-2026.84	0.00	2027.03	0.19
LDEC006899-RA	cuticular protein isoform a	-903.52	0.00	903.52	0.00
LDEC003437-RA	hypothetical protein YQE_12777, partial	-6318.26	0.00	6319.10	0.84
LDEC010793-RA	mature sequence toxin-like lfec	-4120.68	0.00	4121.31	0.63
LDEC006898-RA	endocuticle structural glycoprotein bd-1	-639.51	0.00	639.51	0.00
LDEC007572-RA	hypothetical protein TcasGA2_TC009650	-1216.52	0.00	1216.73	0.21
LDEC015532-RA	glycine-rich protein	-653.83	0.00	653.95	0.11
LDEC017905-RA	protoheme ix mitochondrial-like	-532.73	0.00	532.73	0.00
LDEC001554-RA	female sterile m3	-62.87	0.00	62.89	0.02
LDEC006427-RA	odorant binding protein 1	-322.05	0.00	322.05	0.00
LDEC005466-RA	protein mesh isoform x3	-59.22	0.00	59.24	0.02
LDEC003962-RA	---NA---	-1124.37	0.00	1124.75	0.38
LDEC002650-RA	---NA---	-218.81	0.00	218.89	0.08
LDEC001840-RA	alpha-tocopherol transfer	-54.20	0.00	54.22	0.02
LDEC017904-RA	---NA---	-287.86	0.00	287.97	0.11
LDEC005689-RA	cathepsin partial	-132.23	0.00	132.29	0.06
LDEC020978-RA	troponin isoform 1-like	-167.60	0.00	167.68	0.08
LDEC003401-RA	isoform b	-325.19	0.00	325.35	0.15
LDEC021138-RA	endonuclease-reverse transcriptase	-223.41	0.00	223.53	0.11
LDEC015974-RA	cuticle protein 1	-4306.71	0.00	4309.20	2.49
LDEC022063-RA	---NA---	-168.78	0.00	168.78	0.00
LDEC006897-RA	endocuticle structural glycoprotein bd-8-like	-89.87	0.00	89.93	0.06
LDEC003960-RA	reverse transcriptase	-2646.85	0.00	2648.60	1.74
LDEC005603-RA	cytochrome p450 partial	-57.68	0.00	57.72	0.04
LDEC012916-RA	cytochrome p450 partial	-28.36	0.00	28.38	0.02
LDEC016351-RA	arginine serine-rich splicing factor	-143.79	0.00	143.79	0.00

LDEC000761-RA	aldose reductase	-54.00	0.00	54.04	0.04
LDEC015624-RA	glutathione s-transferase 1-like	-53.15	0.00	53.19	0.04
LDEC002409-RA	PREDICTED: uncharacterized protein LOC100906494	-147.46	0.00	147.58	0.11
LDEC005602-RA	cytochrome p450	-98.31	0.00	98.39	0.08
LDEC013399-RA	hypothetical protein TcasGA2_TC009650	-47.74	0.00	47.78	0.04
LDEC021664-RA	peroxidase-like isoform 1	-46.71	0.00	46.75	0.04
LDEC019558-RA	fatty acyl- reductase cg5065	-116.34	0.00	116.44	0.10
LDEC017199-RA	---NA---	-44.73	0.00	44.77	0.04
LDEC020977-RA	troponin c type iiia-like protein	-43.07	0.00	43.11	0.04
LDEC006145-RA	hypothetical protein TcasGA2_TC011429	-41.86	0.00	41.90	0.04
LDEC005070-RA	glycine-rich protein	-772.34	0.00	773.11	0.77
LDEC012785-RA	chymotrypsin-like proteinase 6d precursor	-38.57	0.00	38.61	0.04
LDEC018594-RA	pericentriolar material 1 protein isoform x4	-100.69	0.00	100.69	0.00
LDEC000487-RA	g2 mitotic-specific cyclin-b3	-96.90	0.00	96.90	0.00
LDEC003887-RA	agap011183-pa-like protein	-89.94	0.00	90.03	0.10
LDEC001591-RA	minus-c odorant binding protein 3	-214.41	0.00	214.64	0.23
LDEC000621-RA	venom acid phosphatase acph-1-like	-34.36	0.00	34.40	0.04
LDEC004916-RA	spfh domain-containing protein 1	-307.64	0.00	308.00	0.36
LDEC001212-RA	mrna cap-binding protein eif4e	-48.29	0.00	48.34	0.06
LDEC003423-RA	cuticular protein ld-cp2	-534.91	0.00	535.57	0.65
LDEC005843-RA	hypothetical protein TcasGA2_TC009650	-79.24	0.00	79.24	0.00
LDEC017411-RA	uracil dna	-30.19	0.00	30.22	0.04
LDEC017171-RA	galactose-specific c-type lectin	-259.39	0.00	259.73	0.34
LDEC023150-RA	af377988_1trypsin-like serine protease	-39.62	0.00	39.67	0.06
LDEC000175-RA	sterol regulatory element-binding protein cleavage-activating protein	-78.63	0.00	78.74	0.11
LDEC000096-RA	krueppel c2h2-type zinc finger	-68.37	0.00	68.37	0.00
LDEC000212-RA	glutathione s-transferase epsilon	-25.90	0.01	25.94	0.04
LDEC001269-RA	39s ribosomal protein mitochondrial-like	-51.24	0.00	51.32	0.08
LDEC000182-RA	atp-dependent rna	-793.21	0.00	794.41	1.21
LDEC011881-RA	serpin peptidase inhibitor 18	-98.98	0.00	99.13	0.15
LDEC008893-RA	odorant-binding protein 4	-63.24	0.00	63.24	0.00
LDEC004853-RA	tubulin alpha-1 chain	-60.21	0.00	60.31	0.10
LDEC000919-RA	glycosyl hydrolase	-36.04	0.00	36.10	0.06
LDEC019814-RA	---NA---	-92.75	0.00	92.90	0.15
LDEC007624-RA	GJ17783	-181.92	0.00	182.23	0.31
LDEC005608-RA	e3 ubiquitin-protein ligase siah2-like	-51.93	0.00	52.02	0.10
LDEC007217-RA	cytochrome p450 6k1	-215.13	0.00	215.53	0.40
LDEC019151-RA	hypothetical protein D910_11774	-39.84	0.00	39.92	0.08
LDEC016769-RA	cytochrome p450 412a1	-49.83	0.00	49.83	0.00
LDEC004854-RA	tubulin alpha-1 chain-like	-49.09	0.00	49.09	0.00
LDEC000920-RA	glycosyl hydrolase	-48.06	0.00	48.06	0.00
LDEC012788-RA	e3 ubiquitin-protein ligase sina	-47.74	0.00	47.74	0.00

LDEC003954-RA	chromatin assembly factor 1 subunit fas2	-47.35	0.00	47.35	0.00
LDEC005398-RA	zinc finger mym-type protein 4 isoform x1	-26.56	0.01	26.61	0.06
LDEC021554-RA	isoform a	-43.88	0.00	43.88	0.00
LDEC000497-RA	PREDICTED: uncharacterized protein LOC100164320	-1058.97	0.00	1061.40	2.43
LDEC005352-RA	ankyrin repeat and sam domain-containing protein 6-like	-42.75	0.00	42.75	0.00
LDEC002262-RA	udp-glucuronosyltransferase 2b10-like	-41.97	0.00	41.97	0.00
LDEC014904-RA	female sterile nasrat	-79.79	0.00	79.98	0.19
LDEC000914-RA	homeobox protein	-41.44	0.00	41.44	0.00
LDEC007472-RA	alkylated dna repair protein alkb homolog 8	-41.19	0.00	41.19	0.00
LDEC008647-RA	protein c19orf12 homolog	-41.19	0.00	41.19	0.00
LDEC019649-RA	---NA---	-101.75	0.00	102.00	0.25
LDEC000496-RA	hypothetical protein AaeL_AAEL010163	-46.85	0.00	46.96	0.11
LDEC004932-RA	protein bicaudal c	-31.17	0.00	31.25	0.08
LDEC003016-RA	set and mynd domain-containing protein 4	-30.47	0.00	30.54	0.08
LDEC000493-RA	peroxisomal acyl-coenzyme a oxidase 3 isoform x1	-45.43	0.00	45.55	0.11
LDEC005391-RA	three prime repair exonuclease 2-like	-38.93	0.00	38.93	0.00
LDEC005474-RA	rna-binding protein squid	-38.89	0.00	38.89	0.00
LDEC017170-RA	isoform a	-29.44	0.00	29.52	0.08
LDEC003697-RA	zinc finger protein zxdc	-87.68	0.00	87.91	0.23
LDEC018543-RA	fibrous sheath cabyr-binding protein isoform x7	-106.03	0.00	106.31	0.29
LDEC009524-RA	mitogen-activated protein kinase erk-	-36.88	0.00	36.88	0.00
LDEC015562-RA	apoptosis inhibitor 5 homolog	-36.56	0.00	36.56	0.00
LDEC011878-RA	antichymotrypsin-2-like isoform x3	-168.16	0.00	168.64	0.48
LDEC005638-RA	glucose dehydrogenase	-660.49	0.00	662.44	1.95
LDEC004760-RA	alpha-tocopherol transfer	-75.65	0.00	75.88	0.23
LDEC011269-RA	sorting nexin-4-like	-32.88	0.00	32.88	0.00
LDEC010055-RA	glucose dehydrogenase	-75.15	0.00	75.38	0.23
LDEC010565-RA	lys-63-specific deubiquitinase brcc36-like isoform x2	-32.35	0.00	32.35	0.00
LDEC017949-RA	thiolester containing protein iv	-31.32	0.00	31.32	0.00
LDEC008646-RA	exonuclease mut-7 homolog	-30.83	0.00	30.83	0.00
LDEC008890-RA	odorant-binding protein 4	-29.83	0.00	29.83	0.00
LDEC001450-RA	tpa_inf: hypothetical secreted protein 323	-28.95	0.00	28.95	0.00
LDEC024515-RA	centrosomal protein of 162 kda	-28.77	0.00	28.77	0.00
LDEC001555-RA	PREDICTED: uncharacterized protein LOC103315004	-28.42	0.00	28.42	0.00
LDEC006908-RA	nad-dependent protein deacetylase sirt2	-27.22	0.00	27.22	0.00
LDEC003838-RA	---NA---	-61.77	0.00	62.00	0.23
LDEC017951-RA	protein c19orf12 homolog	-26.76	0.01	26.76	0.00
LDEC014107-RA	elongation of very long chain fatty acids protein aael008004	-85.14	0.00	85.47	0.33
LDEC020429-RA	prostatic acid phosphatase	-29.08	0.00	29.20	0.11
LDEC021635-RA	circadian clock-controlled protein	-26.99	0.01	27.11	0.11
LDEC001018-RA	maternal effect protein oskar	-78.68	0.00	79.03	0.34
LDEC013085-RA	PREDICTED: uncharacterized protein LOC103314347 isoform X2	-322.12	0.00	323.54	1.42

LDEC018945-RA	juvenile hormone esterase isoform a	-42.60	0.00	42.79	0.19
LDEC006896-RA	cuticular protein rr-1 motif 32	-66.30	0.00	66.61	0.31
LDEC001794-RA	nose resistant to fluoxetine protein 6	-28.39	0.00	28.52	0.13
LDEC024245-RA	galactose-specific c-type	-37.43	0.00	37.62	0.19
LDEC000793-RA	pro-phenol oxidase subunit 2	-185.48	0.00	186.44	0.96
LDEC005072-RA	glycine-rich protein	-90.26	0.00	90.74	0.48
LDEC012486-RA	c-type lectin mannose-binding isoform	-435.75	0.00	438.24	2.49
LDEC015503-RA	cuticle protein precursor	-39.37	0.00	39.60	0.23
LDEC019556-RA	fatty acyl- reductase 1-like	-229.48	0.00	230.82	1.34
LDEC013180-RA	chemosensory protein 6	-326.21	0.00	328.14	1.94
LDEC010942-RA	soma ferritin	-268.89	0.00	270.56	1.67
LDEC000088-RA	facilitated trehalose transporter tret1	-45.01	0.00	45.30	0.29
LDEC011145-RA	acylphosphatase-2-like isoform x3	-68.69	0.00	69.15	0.46
LDEC003453-RA	endocuticle structural glycoprotein bd-1-like	-71.51	0.00	71.98	0.48
LDEC009916-RA	PREDICTED: uncharacterized protein LOC103312712 isoform X1	-33.89	0.00	34.12	0.23
LDEC011404-RA	circadian clock-controlled	-46.35	0.00	46.68	0.33
LDEC005068-RA	glycine-rich protein	-320.84	0.00	323.12	2.28
LDEC007374-RA	pancreatic lipase-related protein 2-like	-67.08	0.00	67.56	0.48
LDEC017948-RA	---NA---	-31.98	0.00	32.21	0.23
LDEC009915-RA	PREDICTED: uncharacterized protein LOC103312712 isoform X2	-48.94	0.00	49.30	0.36
LDEC013547-RA	cuticle protein 21	-159.20	0.00	160.43	1.23
LDEC001642-RA	PREDICTED: uncharacterized protein LOC103314540	-27.36	0.01	27.57	0.21
LDEC010912-RA	proline-rich protein 4-like	-137.58	0.00	138.70	1.11
LDEC003953-RA	---NA---	-196.21	0.00	197.80	1.59
LDEC003961-RA	PREDICTED: uncharacterized protein LOC655051	-40.60	0.00	40.95	0.34
LDEC001375-RA	retinol dehydrogenase 12	-42.14	0.00	42.54	0.40
LDEC000217-RA	ubiquitin carboxyl-terminal hydrolase 35	-33.44	0.00	33.76	0.33
LDEC003964-RA	cuticle protein 64	-28.10	0.00	28.38	0.29
LDEC016838-RA	myotubularin-related protein 14	-325.17	0.00	328.57	3.39
LDEC005655-RA	peritrophic matrix protein 14 precursor	-34.71	0.00	35.07	0.36
LDEC013604-RA	leucine-rich repeats and immunoglobulin-like domains protein 1 isoform x1	-43.57	0.00	44.03	0.46
LDEC001553-RA	grip and coiled-coil domain-containing protein pfc0235w-like	-53.57	0.00	54.15	0.57
LDEC009134-RA	cyclin partial	-48.90	0.00	49.44	0.54
LDEC018522-RA	lipase 3	-202.74	0.00	205.02	2.28
LDEC022246-RA	alpha-2-macroglobulin-like protein 1 isoform x2	-28.27	0.00	28.60	0.33
LDEC005620-RA	ankyrin-1-like isoform x3	-517.58	0.00	523.75	6.17
LDEC024566-RA	hypothetical protein YQE_00236, partial	-29.84	0.00	30.26	0.42
LDEC023633-RA	hypothetical protein YQE_08895, partial	-127.69	0.00	129.53	1.84
LDEC015659-RA	glycosyl hydrolase	-32.63	0.00	33.13	0.50
LDEC018060-RA	esterase	-51.91	0.00	52.77	0.86
LDEC016827-RA	nose resistant to fluoxetine protein 6-like	-28.79	0.00	29.27	0.48
LDEC001262-RA	transcription factor homolog	-54.64	0.00	55.56	0.92

LDEC006198-RA	hunchback	-136.57	0.00	138.91	2.34
LDEC017615-RA	titin-like isoform x2	-55.72	0.00	56.73	1.02
LDEC003077-RA	odorant-binding protein 4	-39.44	0.00	40.17	0.73
LDEC003924-RA	serine threonine-protein kinase nek8	-40.41	0.00	41.16	0.75
LDEC014638-RA	cytochrome p450 9z4	-773.97	0.00	789.28	15.31
LDEC000495-RA	cytochrome c oxidase subunit partial	-42.03	0.00	42.89	0.86
LDEC006721-RA	isoform b	-91.71	0.00	93.61	1.90
LDEC020979-RA	troponin isoform 1-like	-49.52	0.00	50.57	1.05
LDEC000713-RA	cytosolic carboxypeptidase 2-like isoform x1	-64.64	0.00	66.04	1.40
LDEC004769-RA	alpha-tocopherol transfer	-38.12	0.00	38.97	0.84
LDEC007337-RA	agap000696-pa-like protein	-129.15	0.00	132.04	2.89
LDEC018944-RA	juvenile hormone esterase isoform a	-34.20	0.00	34.97	0.77
LDEC003181-RA	p partial	-27.04	0.01	27.68	0.63
LDEC011974-RA	agap011897-pa-like protein	-27.92	0.01	28.60	0.67
LDEC017271-RA	esterase fe4	-75.26	0.00	77.08	1.82
LDEC012839-RA	odorant-binding protein 56a	-212.40	0.00	217.62	5.21
LDEC015419-RA	palmitoyltransferase zdhhc17-like	-88.31	0.00	90.49	2.18
LDEC021327-RA	dnaj homolog subfamily c member 16-like	-1459.97	0.00	1496.46	36.49
LDEC014642-RA	cytochrome p450 9z4	-157.29	0.00	161.28	3.99
LDEC001996-RA	mushroom body large-type kenyon cell-specific protein 1	-264.81	0.00	271.55	6.75
LDEC006597-RA	protein asteroid	-268.88	0.00	275.80	6.92
LDEC009923-RA	metabotropic glutamate receptor	-27.53	0.01	28.24	0.71
LDEC016554-RA	meiosis arrest female protein 1 isoform x1	-111.56	0.00	114.49	2.93
LDEC017037-RA	myosin light chain smooth muscle	-125.06	0.00	128.40	3.33
LDEC002366-RA	aael014823- partial	-64.14	0.00	65.86	1.72
LDEC004440-RA	eri1 exoribonuclease 2-like isoform x3	-102.46	0.00	105.36	2.89
LDEC007589-RA	formin-like protein 8	-245.77	0.00	252.76	6.99
LDEC014637-RA	PREDICTED: uncharacterized protein K02A2.6-like	-80.64	0.00	82.96	2.32
LDEC002396-RA	fatty acid hydroxylase domain-containing protein 2 isoform x2	-283.11	0.00	291.44	8.34
LDEC000615-RA	protein lethal essential for life	-91.15	0.00	93.86	2.70
LDEC017038-RA	nuclear anchorage protein 1-like	-557.18	0.00	574.14	16.96
LDEC014744-RA	heterotrimeric gtp-binding protein alpha subunit g-alpha-q	-51.41	0.00	52.98	1.57
LDEC003613-RA	werner syndrome helicase	-28.60	0.01	29.52	0.92
LDEC010361-RA	succinyl- synthetase small	-68.93	0.00	71.17	2.24
LDEC006447-RA	aldehyde oxidase	-61.04	0.00	63.03	1.99
LDEC018236-RA	set and mynd domain-containing protein 4-like	-59.82	0.00	61.79	1.97
LDEC006855-RA	colostrum trypsin	-58.88	0.00	60.84	1.95
LDEC007522-RA	leucine-rich repeat protein soc-2 isoform x1	-695.33	0.00	718.78	23.45
LDEC003211-RA	pupal cuticle protein	-35.74	0.00	36.95	1.21
LDEC013734-RA	vacuolar protein sorting-associated protein 27 isoform x2	-384.19	0.00	397.22	13.03
LDEC013928-RA	bifunctional udp-n-acetylglucosamine transferase and deubiquitinase alg13 isoform x1	-32.84	0.00	33.97	1.13
LDEC009154-RA	hypothetical protein YQE_09144, partial	-117.98	0.00	122.10	4.12

LDEC007771-RA	unknown	-90.09	0.00	93.25	3.16
LDEC017298-RA	2 -phosphodiesterase 12-like	-55.08	0.00	57.01	1.94
LDEC022019-RA	tpa: cuticle protein	-145.54	0.00	150.73	5.19
LDEC020419-RA	protein beta isoform-like isoform x18	-59.04	0.00	61.19	2.15
LDEC013747-RA	scavenger receptor class b member 1-like	-37.96	0.00	39.35	1.40
LDEC007553-RA	vitellogenin receptor	-191.96	0.00	199.07	7.11
LDEC000656-RA	agap013007-pa-like protein	-92.07	0.00	95.48	3.41
LDEC006671-RA	serine protease snake-like	-560.42	0.00	581.33	20.91
LDEC005827-RA	PREDICTED: uncharacterized protein LOC103568218 isoform X3	-1821.27	0.00	1889.65	68.37
LDEC004195-RA	cholesterol desaturase daf-36	-43.78	0.00	45.55	1.76
LDEC004859-RA	protein d7	-55.74	0.00	58.01	2.26
LDEC009305-RA	e3 ubiquitin-protein ligase siah1b	-38.24	0.00	39.81	1.57
LDEC021326-RA	defensin precursor	-3275.83	0.00	3411.66	135.82
LDEC016916-RA	15-hydroxyprostaglandin dehydrogenase	-327.83	0.00	341.59	13.76
LDEC016770-RA	cytochrome p450 4c1-like	-64.94	0.00	67.74	2.80
LDEC017225-RA	lysoplasmalogenase-like protein tmem86a	-99.25	0.00	103.62	4.37
LDEC003727-RA	pathogenesis-related protein 5	-28.23	0.01	29.48	1.25
LDEC021910-RA	probable low-specificity l-threonine aldolase 2 isoform x1	-182.86	0.00	191.04	8.18
LDEC011244-RA	cytoplasmic a3	-42.94	0.00	44.88	1.94
LDEC013868-RA	ring finger protein 145	-31.23	0.00	32.67	1.44
LDEC018992-RA	glycine-rich cell wall structural protein	-46.16	0.00	48.31	2.15
LDEC017005-RA	pollen-specific leucine-rich repeat extensin-like protein 1	-4277.20	0.00	4476.24	199.04
LDEC000159-RA	multiple epidermal growth factor-like domains protein 10	-58.02	0.00	60.80	2.78
LDEC022697-RA	vitellogenin receptor	-43.39	0.00	45.48	2.09
LDEC003354-RA	isoform b	-74.35	0.00	77.93	3.58
LDEC015622-RA	glutathione s-transferase	-53.21	0.00	55.78	2.57
LDEC007552-RA	vitellogenin receptor	-69.34	0.00	72.69	3.35
LDEC017272-RA	venom carboxylesterase-6-like	-44.51	0.00	46.68	2.17
LDEC005679-RA	cuticular protein ld-cp1v1	-472.61	0.00	495.68	23.07
LDEC000165-RA	cyclin-dependent kinase inhibitor 1b-like isoform x4	-50.14	0.00	52.59	2.45
LDEC000226-RA	tyrosine hydroxylase	-1924.14	0.00	2019.71	95.56
LDEC022111-RA	hypothetical protein TcasGA2_TC000293	-123.48	0.00	129.64	6.15
LDEC017761-RA	f-box only protein 25	-51.98	0.00	54.61	2.63
LDEC012380-RA	isoform s	-144.15	0.00	151.44	7.28
LDEC016555-RA	meiosis arrest female protein 1 isoform x2	-41.09	0.00	43.18	2.09
LDEC007505-RA	pancreatic triacylglycerol lipase-like isoform x1	-87.00	0.00	91.48	4.48
LDEC005826-RA	PREDICTED: hypothetical protein LOC100748865	-1022.28	0.00	1075.59	53.31
LDEC014525-RA	heat shock protein 70	-80.02	0.00	84.19	4.18
LDEC020501-RA	beta- -glucosyltransferase	-38.07	0.00	40.06	1.99
LDEC015776-RA	headcase protein	-490.76	0.00	516.67	25.91
LDEC002991-RA	PREDICTED: uncharacterized protein LOC103314074	-217.13	0.00	228.62	11.50
LDEC000143-RA	PREDICTED: uncharacterized protein LOC100141808	-96.60	0.00	101.71	5.12

LDEC002282-RA	arrestin domain-containing protein 3	-59.23	0.00	62.43	3.20
LDEC009652-RA	poly rna polymerase gld-2 homolog a-like	-135.71	0.00	143.08	7.38
LDEC007506-RA	pancreatic triacylglycerol lipase-like	-111.14	0.00	117.25	6.11
LDEC014644-RA	cytochrome p450 9z4	-133.04	0.00	140.36	7.32
LDEC000589-RA	protein croquemort-like	-80.67	0.00	85.33	4.66
LDEC011677-RA	equilibrative nucleoside transporter 1	-185.06	0.00	195.89	10.83
LDEC012958-RA	alkylglycerol monooxygenase-like	-195.13	0.00	206.61	11.48
LDEC015650-RA	hypothetical protein YQE_08052, partial	-35.58	0.00	37.69	2.11
LDEC012765-RA	paired box protein pax-5	-36.57	0.00	38.75	2.18
LDEC016941-RA	actin binding protein	-44.63	0.00	47.32	2.68
LDEC006974-RA	low density lipoprotein receptor adapter protein 1	-92.05	0.00	97.61	5.56
LDEC020031-RA	nucleotide excision repair protein	-46.35	0.00	49.23	2.87
LDEC005044-RA	PREDICTED: uncharacterized protein LOC662511 isoform X1	-60.53	0.00	64.30	3.78
LDEC018960-RA	ac transposable element-derived protein partial	-83.91	0.00	89.29	5.38
LDEC008006-RA	n-acetylglucosamine-1-phosphotransferase subunits alpha beta	-39.50	0.00	42.04	2.55
LDEC012294-RA	leucine-rich repeats and immunoglobulin-like domains protein 1	-40.50	0.00	43.14	2.64
LDEC017614-RA	titin-like isoform x1	-1558.62	0.00	1662.86	104.24
LDEC022010-RA	venom carboxylesterase-6-like	-171.96	0.00	183.61	11.65
LDEC002253-RA	protein fam151b isoform x1	-457.03	0.00	488.07	31.04
LDEC005537-RA	toll-like receptor 3	-52.99	0.00	56.59	3.60
LDEC009315-RA	ly-6 neurotoxin-related protein	-386.89	0.00	413.22	26.33
LDEC005298-RA	pr domain zinc finger protein partial	-235.50	0.00	251.56	16.06
LDEC024418-RA	ac transposable element-derived protein partial	-30.58	0.01	32.67	2.09
LDEC011291-RA	---NA---	-57.12	0.00	61.05	3.93
LDEC017273-RA	juvenile hormone esterase	-66.25	0.00	70.85	4.60
LDEC009889-RA	yellow-e precursor	-212.91	0.00	227.99	15.08
LDEC006232-RA	receptor-type tyrosine-protein phosphatase f-like	-226.11	0.00	242.32	16.21
LDEC014643-RA	cytochrome p450 9z4	-33.77	0.00	36.20	2.43
LDEC004707-RA	pdz domain protein	-80.10	0.00	85.93	5.83
LDEC019089-RA	l-allo-threonine aldolase	-213.66	0.00	229.26	15.60
LDEC019378-RA	myosin light chain smooth muscle	-31.34	0.00	33.66	2.32
LDEC009552-RA	ankyrin-3-like isoform x4	-137.88	0.00	148.32	10.44
LDEC018156-RA	zinc finger and btb domain-containing protein 49-like isoform x2	-44.28	0.00	47.67	3.39
LDEC011517-RA	sporulation-specific protein 15	-382.93	0.00	412.69	29.76
LDEC017739-RA	inorganic phosphate cotransporter	-41.34	0.00	44.56	3.22
LDEC002302-RA	zinc finger protein 271-like	-32.56	0.00	35.11	2.55
LDEC006337-RA	protein yellow	-43.29	0.00	46.68	3.39
LDEC008186-RA	PREDICTED: uncharacterized protein LOC103521349, partial	-39.51	0.00	42.61	3.10
LDEC024010-RA	isoform a	-482.75	0.00	521.27	38.52
LDEC018662-RA	inorganic phosphate cotransporter	-45.45	0.00	49.09	3.64
LDEC022959-RA	leukocyte elastase inhibitor-like	-30.37	0.01	32.81	2.43
LDEC019590-RA	aminopeptidase n	-291.19	0.00	314.59	23.40

LDEC021667-RA	p partial	-30.85	0.01	33.34	2.49
LDEC002431-RA	hypothetical protein TcasGA2_TC007080	-177.38	0.00	191.71	14.33
LDEC013514-RA	probable gpi-anchored adhesin-like protein pga55 isoform x2	-35.89	0.00	38.82	2.93
LDEC017558-RA	esterase fe4-like	-48.46	0.00	52.45	3.99
LDEC021362-RA	hypothetical protein D910_11256	-106.44	0.00	115.20	8.76
LDEC001873-RA	isoform b	-34.30	0.00	37.16	2.86
LDEC004501-RA	multidrug resistance-associated protein 4-like	-30.07	0.01	32.59	2.53
LDEC013528-RA	protein tprxl isoform x2	-125.46	0.00	136.04	10.58
LDEC003154-RA	nuclear pore complex protein ddb_g0274915 homolog isoform x1	-77.01	0.00	83.52	6.52
LDEC001049-RA	aminopeptidase n	-1770.58	0.00	1922.06	151.48
LDEC010678-RA	e3 ubiquitin-protein ligase topors	-66.33	0.00	72.02	5.69
LDEC014731-RA	thymidylate kinase	-89.29	0.00	96.97	7.68
LDEC011854-RA	cysteine and histidine-rich protein 1 homolog	-203.40	0.00	221.09	17.69
LDEC016448-RA	PREDICTED: uncharacterized protein LOC103312904	-179.54	0.00	195.18	15.64
LDEC000534-RA	methenyltetrahydrofolate synthase domain-containing protein	-79.68	0.00	86.67	6.99
LDEC015038-RA	toll-like receptor partial	-91.03	0.00	99.09	8.07
LDEC023861-RA	meiosis arrest female protein 1 isoform x2	-46.87	0.00	51.03	4.16
LDEC006490-RA	hypothetical protein	-183.30	0.00	199.78	16.48
LDEC006021-RA	cytochrome p450 6bq10	-136.95	0.00	149.38	12.44
LDEC017119-RA	udp-glucuronosyltransferase 2b17-like	-197.60	0.00	215.85	18.24
LDEC007332-RA	dna-directed rna polymerase i subunit rpa1 isoform x1	-54.79	0.00	59.85	5.06
LDEC006252-RA	phosphatidylinositol 3-kinase catalytic subunit type 3	-101.36	0.00	110.77	9.41
LDEC020670-RA	phosphoenolpyruvate carboxykinase	-1299.88	0.00	1422.14	122.26
LDEC004413-RA	asparagine--trna cytoplasmic	-32.10	0.01	35.14	3.05
LDEC022696-RA	vitellogenin receptor	-34.46	0.00	37.83	3.37
LDEC004855-RA	retrovirus-related pol polyprotein from transposon partial	-58.84	0.00	64.62	5.79
LDEC003028-RA	ubiquitin-conjugating enzyme e2-230k	-45.70	0.00	50.22	4.52
LDEC017560-RA	esterase fe4-like	-256.72	0.00	282.38	25.66
LDEC005680-RA	cuticular protein 100a	-90.25	0.00	99.27	9.03
LDEC020707-RA	major facilitator superfamily protein	-41.65	0.00	45.83	4.18
LDEC000590-RA	protein croquemort-like isoform x3	-72.94	0.00	80.37	7.44
LDEC014696-RA	lipase member i	-48.27	0.00	53.19	4.92
LDEC018663-RA	inorganic phosphate cotransporter	-81.26	0.00	89.57	8.32
LDEC018730-RA	protein smaug homolog 1	-130.17	0.00	143.51	13.34
LDEC012340-RA	trichohyalin isoform x1	-57.74	0.00	63.70	5.96
LDEC006426-RA	PREDICTED: mucin-4	-174.81	0.00	193.13	18.32
LDEC017616-RA	ankyrin-2-like isoform x5	-47.76	0.00	52.80	5.04
LDEC016716-RA	rna-binding protein 40	-95.62	0.00	105.89	10.27
LDEC017269-RA	pickpocket protein 28-like	-37.10	0.00	41.09	3.99
LDEC004287-RA	inositol hexakisphosphate kinase 1 isoform x2	-88.57	0.00	98.21	9.64
LDEC014114-RA	glycogen-binding subunit 76a	-62.28	0.00	69.12	6.84
LDEC020674-RA	zinc finger protein 2 homolog	-56.59	0.00	62.82	6.23

LDEC010330-RA	PREDICTED: uncharacterized protein LOC656470	-218.09	0.00	242.14	24.05
LDEC018465-RA	cathepsin b	-354.81	0.00	394.29	39.47
LDEC001146-RA	protein isoform b-like	-63.53	0.00	70.64	7.11
LDEC011540-RA	zinc finger ccch domain-containing protein 3	-141.69	0.00	157.63	15.94
LDEC021671-RA	cuticle protein 6	-42.16	0.00	46.93	4.77
LDEC008871-RA	e3 ubiquitin-protein ligase siah2-like	-268.50	0.00	298.91	30.41
LDEC003594-RA	tyrosine-protein phosphatase non-receptor	-481.55	0.00	536.66	55.11
LDEC015913-RA	protein toll	-285.73	0.00	318.44	32.71
LDEC003377-RA	protein henna	-144.18	0.00	160.78	16.59
LDEC010733-RA	apolipoprotein d-like	-300.64	0.00	335.61	34.97
LDEC001884-RA	heat shock factor protein isoform x4	-49.81	0.00	55.63	5.83
LDEC013733-RA	tyrosine-protein phosphatase non-receptor type 23	-90.54	0.00	101.22	10.67
LDEC002903-RA	PREDICTED: uncharacterized protein LOC664170 isoform X1	-64.20	0.00	71.77	7.57
LDEC011389-RA	nose resistant to fluoxetine protein 6-like isoform x1	-32.74	0.01	36.63	3.89
LDEC001926-RA	PREDICTED: uncharacterized protein LOC103313770	-98.44	0.00	110.17	11.73
LDEC013907-RA	f-box lrr-repeat protein 20 isoform x3	-35.16	0.00	39.35	4.20
LDEC015737-RA	chitin deacetylase 5 isoform x5	-490.44	0.00	548.98	58.54
LDEC002630-RA	hypothetical protein YQE_07290, partial	-104.37	0.00	116.86	12.49
LDEC012138-RA	PREDICTED: uncharacterized protein LOC655754 isoform X1	-137.01	0.00	153.45	16.44
LDEC013680-RA	nucleoporin-like 2	-118.58	0.00	132.82	14.24
LDEC018567-RA	probable myosin light chain kinase ddb_g0279831	-71.84	0.00	80.48	8.64
LDEC017412-RA	uracil dna	-55.69	0.00	62.39	6.71
LDEC002239-RA	e3 ubiquitin-protein ligase siah1-like	-63.57	0.00	71.28	7.70
LDEC020285-RA	conserved protein	-71.83	0.00	80.55	8.72
LDEC003025-RA	f-box lrr-repeat protein 4	-43.32	0.00	48.59	5.27
LDEC006480-RA	leucine-rich transmembrane	-34.13	0.00	38.33	4.20
LDEC020044-RA	growth factor receptor-bound protein 14-like	-116.99	0.00	131.44	14.45
LDEC009065-RA	PREDICTED: uncharacterized protein LOC100141997	-110.91	0.00	124.65	13.74
LDEC010172-RA	hormone receptor 4 isoform x2	-112.31	0.00	126.38	14.07
LDEC018881-RA	differentially expressed in fdcp 6-like protein	-76.73	0.00	86.39	9.66
LDEC000806-RA	cytoplasmic polyadenylation element-binding protein 1-like	-32.62	0.01	36.74	4.12
LDEC020380-RA	eukaryotic translation initiation factor 4e transporter-like isoform x2	-40.83	0.00	46.01	5.17
LDEC011960-RA	histone-lysine n-methyltransferase 2d isoform x1	-122.52	0.00	138.06	15.54
LDEC021034-RA	ubiquitin carboxyl-terminal hydrolase 2-like isoform x2	-117.17	0.00	132.04	14.87
LDEC005137-RA	reverse transcriptase	-58.07	0.00	65.54	7.47
LDEC005786-RA	nesprin-1 isoform x1	-732.82	0.00	828.21	95.39
LDEC019073-RA	sporulation-specific protein 15	-300.90	0.00	340.10	39.21
LDEC007026-RA	PREDICTED: uncharacterized protein LOC103314916	-52.79	0.00	59.67	6.88
LDEC021802-RA	PREDICTED: uncharacterized protein LOC100574526	-48.35	0.00	54.68	6.32
LDEC003633-RA	sodium-coupled neutral amino acid transporter 9	-41.17	0.00	46.61	5.44
LDEC007955-RA	general receptor for phosphoinositides 1-associated scaffold protein	-73.43	0.00	83.17	9.73
LDEC019373-RA	stretchin- isoform g	-283.03	0.00	320.60	37.58

LDEC013779-RA	protein claret segregational	-93.16	0.00	105.53	12.38
LDEC005054-RA	hypothetical protein EAI_06447	-36.56	0.00	41.44	4.89
LDEC005604-RA	cytochrome p450 6a2	-65.23	0.00	73.97	8.74
LDEC018369-RA	zinc finger protein 271-like	-36.80	0.00	41.76	4.96
LDEC006735-RA	PREDICTED: uncharacterized protein LOC103313913 isoform X1	-156.12	0.00	177.24	21.12
LDEC006286-RA	nuclear factor of activated t-cells 5	-107.57	0.00	122.17	14.60
LDEC016408-RA	sestrin homolog	-497.76	0.00	566.07	68.31
LDEC020382-RA	eukaryotic translation initiation factor 4e transporter-like isoform x4	-185.58	0.00	211.07	25.49
LDEC007954-RA	general receptor for phosphoinositides 1-associated scaffold protein	-114.84	0.00	130.63	15.79
LDEC000682-RA	protein turtle isoform x1	-517.76	0.00	589.29	71.53
LDEC014399-RA	cuticle protein	-72.58	0.00	82.64	10.06
LDEC015889-RA	gametogenetin-binding protein 2-like	-43.44	0.00	49.48	6.04
LDEC003959-RA	glucose dehydrogenase	-50.23	0.00	57.23	6.99
LDEC004535-RA	plk4_droan ame: full=serine threonine-protein kinase plk4 ame: full=polo-like kinase 4 short=plk-4 ame: full=serine threonine-protein kinase sak	-54.97	0.00	62.68	7.70
LDEC013805-RA	cryptochrome 2	-78.03	0.00	88.97	10.94
LDEC001101-RA	agap008807-pa-like protein	-197.53	0.00	225.47	27.94
LDEC000787-RA	arylsulfatase b-like	-38.95	0.00	44.49	5.54
LDEC003056-RA	PREDICTED: uncharacterized protein LOC660988 isoform X2	-119.78	0.00	136.89	17.11
LDEC014029-RA	---NA---	-146.33	0.00	167.29	20.96
LDEC013672-RA	transcriptional-regulating factor 1 isoform x2	-87.58	0.00	100.23	12.65
LDEC009280-RA	---NA---	-144.09	0.00	164.92	20.83
LDEC015817-RA	upf0472 protein c16orf72 homolog	-38.50	0.00	44.10	5.60
LDEC006249-RA	phosphatidylinositol 3-kinase catalytic subunit type 3	-206.10	0.00	236.09	29.99
LDEC008684-RA	probable phosphoserine aminotransferase	-4625.16	0.00	5301.41	676.25
LDEC003710-RA	round spermatid basic protein 1	-68.21	0.00	78.25	10.04
LDEC004632-RA	digestive cysteine protease intestain	-145.13	0.00	166.65	21.52
LDEC020878-RA	hypothetical protein TcasGA2_TC013968	-63.27	0.00	72.66	9.39
LDEC000938-RA	inositol-tetrakisphosphate 1-kinase	-60.12	0.00	69.05	8.93
LDEC021303-RA	thyroid receptor-interacting protein 11-like	-275.92	0.00	317.10	41.18
LDEC019026-RA	zinc finger protein 271-like	-55.48	0.00	63.77	8.30
LDEC005125-RA	calmodulin-binding transcription activator 2-like isoform x3	-47.92	0.00	55.10	7.19
LDEC013049-RA	zinc finger protein 271-like	-43.24	0.00	49.76	6.52
LDEC003774-RA	ferm domain-containing protein 8-like	-36.60	0.00	42.11	5.52
LDEC002545-RA	proactivator polypeptide	-348.22	0.00	400.76	52.54
LDEC005613-RA	PREDICTED: uncharacterized protein LOC100141684	-115.13	0.00	132.89	17.76
LDEC003397-RA	bifunctional purine biosynthesis protein purh	-1560.81	0.00	1801.91	241.10
LDEC017559-RA	esterase	-293.27	0.00	338.72	45.45
LDEC007576-RA	e3 ubiquitin-protein ligase ubr4 isoform x2	-154.31	0.00	178.30	23.99
LDEC013255-RA	protein crebrf homolog	-118.12	0.00	136.50	18.38
LDEC007658-RA	probable gpi-anchored adhesin-like protein pga55	-130.89	0.00	151.26	20.37
LDEC003128-RA	ubiquitin-associated protein 1-like	-53.68	0.00	62.08	8.39

LDEC013607-RA	slit homolog 2 protein	-50.04	0.00	57.90	7.86
LDEC018833-RA	hypothetical protein TcasGA2_TC007902	-196.26	0.00	227.17	30.91
LDEC019798-RA	gastrula zinc finger protein	-35.04	0.01	40.56	5.52
LDEC002657-RA	4-coumarate-- ligase 1-like	-37.71	0.00	43.67	5.96
LDEC003014-RA	cell cycle checkpoint kinase 2	-41.77	0.00	48.38	6.61
LDEC002250-RA	brca1-a complex subunit abraxas-like	-37.45	0.00	43.39	5.94
LDEC020945-RA	ubiquitin-like protein 3	-121.76	0.00	141.14	19.37
LDEC009532-RA	ecdysone-induced protein 74ef isoform a isoform x1	-63.57	0.00	73.68	10.12
LDEC007531-RA	ras-related and estrogen-regulated growth inhibitor isoform x3	-35.87	0.00	41.58	5.71
LDEC014183-RA	rna polymerase ii elongation factor ell-like	-524.35	0.00	608.15	83.80
LDEC018941-RA	alpha partial	-719.83	0.00	835.40	115.57
LDEC005607-RA	nose resistant to fluoxetine protein 6-like	-36.20	0.00	42.08	5.88
LDEC023555-RA	mucin-2-like isoform x1	-67.57	0.00	78.57	11.00
LDEC006388-RA	zinc finger protein 704-like	-392.05	0.00	455.97	63.93
LDEC020702-RA	timeless isoform b	-87.12	0.00	101.43	14.31
LDEC022146-RA	sprt-like domain-containing protein spartan	-42.85	0.00	49.90	7.05
LDEC010167-RA	protein tyrosine non-receptor type nt6	-51.57	0.00	60.09	8.53
LDEC008075-RA	an1-type zinc finger protein 6 isoform x3	-1158.99	0.00	1353.34	194.35
LDEC001166-RA	uncharacterized serine-rich	-60.31	0.00	70.43	10.12
LDEC004770-RA	venom carboxylesterase-6-like	-284.15	0.00	331.96	47.81
LDEC007743-RA	a-kinase anchor protein mitochondrial	-109.78	0.00	128.26	18.47
LDEC001880-RA	protein pat1 homolog 1	-379.79	0.00	443.69	63.91
LDEC004579-RA	serine threonine-protein kinase tricorner isoform x2	-349.06	0.00	408.23	59.17
LDEC004019-RA	circadian protein clock arnt bmal pas	-45.50	0.00	53.23	7.72
LDEC010191-RA	---NA---	-39.02	0.00	45.65	6.63
LDEC000857-RA	poly -binding protein 3 isoform x1	-408.22	0.00	477.63	69.41
LDEC010047-RA	inner centromere protein a-like	-42.83	0.00	50.15	7.32
LDEC009513-RA	pleckstrin-like proteiny domain-containing family m member 2	-136.94	0.00	160.39	23.45
LDEC002380-RA	hermansky-pudlak syndrome 5	-73.39	0.00	86.00	12.61
LDEC015874-RA	rna polymerase ii subunit a c-terminal domain phosphatase	-37.13	0.00	43.53	6.40
LDEC003593-RA	tyrosine-protein phosphatase non-receptor	-44.88	0.00	52.66	7.78
LDEC002706-RA	probable atp-dependent rna helicase dhx35	-391.08	0.00	458.87	67.80
LDEC003983-RA	rwd domain-containing protein 2a	-59.30	0.00	69.61	10.31
LDEC014022-RA	bromodomain-containing protein 4-like	-259.33	0.00	304.82	45.49
LDEC018917-RA	agap001177-pa-like protein	-54.98	0.00	64.69	9.72
LDEC008456-RA	zinc finger protein 271-like	-44.26	0.00	52.09	7.84
LDEC011736-RA	nuclear pore complex protein nup214	-64.03	0.00	75.45	11.42
LDEC010754-RA	hypothetical protein DAPPUDRAFT_15773	-144.05	0.00	169.80	25.75
LDEC005343-RA	xanthine dehydrogenase	-165.87	0.00	195.57	29.70
LDEC009801-RA	titin isoform x1	-2398.70	0.00	2829.34	430.64
LDEC011738-RA	nuclear pore complex protein nup214	-97.03	0.00	114.49	17.46
LDEC000021-RA	transposase [Drosophila buzzatii]	-41.38	0.00	48.84	7.45

LDEC003502-RA	dopamine n-acetyltransferase-like isoform x4	-232.03	0.00	273.99	41.97
LDEC018850-RA	maternal tudor protein	-62.36	0.00	73.65	11.29
LDEC016412-RA	zinc finger protein 260	-74.62	0.00	88.23	13.61
LDEC006124-RA	cadherin 23	-41.66	0.00	49.26	7.61
LDEC019393-RA	vacuolar protein sorting-associated protein	-52.55	0.00	62.15	9.60
LDEC001620-RA	sv2-like protein 1	-196.11	0.00	232.23	36.12
LDEC015770-RA	cytochrome p450 9z4	-101.11	0.00	119.83	18.72
LDEC004550-RA	protein btg1-like	-425.95	0.00	504.85	78.89
LDEC021181-RA	hairy enhancer-of-split related with yrpw motif-like protein	-98.18	0.00	116.36	18.19
LDEC001098-RA	hypothetical protein TcasGA2_TC004643	-81.11	0.00	96.16	15.04
LDEC001747-RA	eye-specific diacylglycerol kinase isoform x1	-66.49	0.00	78.85	12.36
LDEC010877-RA	cathepsin b precursor	-114.95	0.00	136.40	21.44
LDEC010669-RA	phosphatidate phosphatase lpin3	-92.27	0.00	109.53	17.27
LDEC002494-RA	cytochrome p450 6k1	-63.08	0.00	74.92	11.84
LDEC017044-RA	PREDICTED: uncharacterized protein LOC659539	-568.31	0.00	675.71	107.41
LDEC018699-RA	cytochrome p450 9e2	-104.88	0.00	124.72	19.83
LDEC015907-RA	laccase isoform f	-160.61	0.00	191.07	30.47
LDEC022938-RA	nedd8 ultimate buster 1	-44.11	0.00	52.48	8.37
LDEC015569-RA	zinc finger protein 271-like	-38.11	0.00	45.37	7.26
LDEC001823-RA	zinc finger swim domain-containing protein 7-like	-48.89	0.00	58.22	9.33
LDEC004548-RA	PREDICTED: uncharacterized protein LOC661444	-293.75	0.00	349.98	56.22
LDEC014639-RA	cytochrome p450 9z4	-151.25	0.00	180.21	28.95
LDEC006709-RA	5 -3 exoribonuclease 1-like isoform x1	-128.63	0.00	153.31	24.68
LDEC006056-RA	zinc finger protein 260-like isoform x1	-49.58	0.00	59.10	9.52
LDEC005237-RA	eukaryotic translation initiation factor 2-alpha kinase 4	-48.71	0.00	58.08	9.37
LDEC015591-RA	uncharacterized threonine-rich gpi-anchored glyco	-70.70	0.00	84.30	13.61
LDEC002171-RA	afadin isoform x2	-112.25	0.00	133.88	21.63
LDEC015584-RA	diamine acetyltransferase 2	-36.69	0.01	43.78	7.09
LDEC004545-RA	mariner transposase	-354.21	0.00	422.81	68.60
LDEC008089-RA	ring finger protein	-60.90	0.00	72.73	11.82
LDEC011739-RA	ribosome biogenesis protein wdr12 homolog	-270.28	0.00	322.76	52.49
LDEC015881-RA	oxidase peroxidase	-37.61	0.00	44.95	7.34
LDEC012906-RA	PREDICTED: uncharacterized protein LOC103312687	-60.36	0.00	72.16	11.80
LDEC010667-RA	phosphatidate phosphatase lpin3 isoform x3	-48.83	0.00	58.43	9.60
LDEC003628-RA	zinc finger protein partial	-84.88	0.00	101.57	16.69
LDEC002720-RA	zinc finger protein 271-like	-104.75	0.00	125.42	20.68
LDEC007993-RA	low quality protein: mucin-17	-131.61	0.00	157.59	25.98
LDEC007209-RA	nfx1-type zinc finger-containing protein 1-like isoform x1	-105.31	0.00	126.10	20.79
LDEC007274-RA	spermatogenesis-associated protein 13-like isoform x2	-40.56	0.00	48.63	8.07
LDEC017718-RA	low quality protein: probable e3 ubiquitin-protein ligase mycbp2-like	-39.97	0.00	47.92	7.95
LDEC004508-RA	mariner transposase	-38.99	0.00	46.75	7.76
LDEC003632-RA	sodium-coupled neutral amino acid transporter 9	-44.84	0.00	53.83	8.99

LDEC000948-RA	hmg box transcription factor bbx	-159.22	0.00	191.14	31.92
LDEC005238-RA	eukaryotic translation initiation factor 2-alpha kinase 4	-44.13	0.00	52.98	8.85
LDEC019376-RA	myosin light chain	-46.39	0.00	55.70	9.31
LDEC005788-RA	muscle-specific protein 300	-39.11	0.00	46.96	7.86
LDEC019392-RA	vacuolar protein sorting-associated protein 13a-like isoform x1	-65.87	0.00	79.17	13.30
LDEC007934-RA	mob kinase activator-like 3	-58.98	0.00	70.92	11.94
LDEC015435-RA	ca(2+) calmodulin-responsive adenylate cyclase isoform x7	-65.95	0.00	79.31	13.36
LDEC014403-RA	zinc finger protein 502-like isoform x1	-37.52	0.01	45.12	7.61
LDEC001108-RA	hypothetical protein TcasGA2_TC004643	-41.58	0.00	50.01	8.43
LDEC002669-RA	tyrosine-protein phosphatase non-receptor type 1 isoform x1	-118.68	0.00	142.77	24.09
LDEC008211-RA	hypothetical protein X975_24243, partial	-50.72	0.00	61.05	10.33
LDEC014762-RA	probable phospholipid-transporting atpase ia isoform x4	-100.76	0.00	121.32	20.56
LDEC006995-RA	leukocyte receptor tyrosine protein kinase	-59.85	0.00	72.09	12.24
LDEC011079-RA	upf0547 protein c16orf87-like	-65.71	0.00	79.24	13.53
LDEC002306-RA	period isoform x1	-42.17	0.00	50.93	8.76
LDEC011361-RA	superoxide dismutase	-42.36	0.00	51.17	8.81
LDEC005623-RA	isoform b	-93.39	0.00	112.90	19.51
LDEC016130-RA	hypothetical protein D910_10956	-251.49	0.00	304.11	52.62
LDEC010725-RA	15-hydroxyprostaglandin dehydrogenase	-261.33	0.00	316.07	54.75
LDEC004584-RA	cold shock domain-containing protein e1	-1016.95	0.00	1230.53	213.59
LDEC005297-RA	zinc finger protein 251-like	-104.49	0.00	126.45	21.96
LDEC000838-RA	#NAME?	-48.33	0.00	58.50	10.18
LDEC023728-RA	tbc1 domain family member 10a	-99.33	0.00	120.26	20.93
LDEC005755-RA	diapause-associated transcript-2	-162.24	0.00	196.52	34.28
LDEC000150-RA	gem-associated protein 5	-103.30	0.00	125.14	21.85
LDEC007737-RA	peptidyl-prolyl cis-trans isomerase-like 2-like	-199.59	0.00	241.86	42.27
LDEC023114-RA	upf0420 protein c16orf58 homolog	-125.33	0.00	151.93	26.60
LDEC003011-RA	tetratricopeptide repeat protein 36 homolog	-43.20	0.00	52.38	9.18
LDEC009925-RA	lipophorin receptor	-468.44	0.00	568.09	99.65
LDEC019104-RA	ring finger protein unkempt	-39.56	0.00	47.99	8.43
LDEC018540-RA	cd151 antigen-like	-120.60	0.00	146.41	25.81
LDEC008854-RA	molting isoform e	-40.27	0.00	48.91	8.64
LDEC008387-RA	homolog of	-414.44	0.00	503.64	89.20
LDEC004560-RA	persulfide dioxygenase mitochondrial	-260.87	0.00	317.21	56.34
LDEC004273-RA	tripartite motif-containing protein 2 isoform x3	-70.25	0.00	85.50	15.25
LDEC015705-RA	fat-like cadherin-related tumor suppressor homolog isoform x1	-43.24	0.00	52.63	9.39
LDEC019143-RA	zinc transporter 2-like	-47.64	0.00	58.01	10.37
LDEC008551-RA	transcription initiation factor tfiid subunit 5-like	-59.63	0.00	72.66	13.03
LDEC000981-RA	choline-phosphate cytidylyltransferase b-like	-60.50	0.00	73.72	13.22
LDEC013895-RA	acyl- synthetase family member mitochondrial	-56.56	0.00	68.94	12.38
LDEC006276-RA	zinc finger protein 431-like	-81.07	0.00	98.81	17.74
LDEC006966-RA	reverse partial	-549.66	0.00	670.90	121.24

LDEC015367-RA	star-related lipid transfer protein 3	-59.77	0.00	72.98	13.20
LDEC012258-RA	box a-binding factor-like isoform x1	-97.20	0.00	118.70	21.50
LDEC005585-RA	e3 ubiquitin-protein ligase herc2	-75.79	0.00	92.62	16.82
LDEC006546-RA	zinc finger protein 658-like isoform x1	-48.86	0.00	59.70	10.85
LDEC008741-RA	tubby-related protein 1	-496.20	0.00	607.06	110.86
LDEC004680-RA	inosine-5 -monophosphate dehydrogenase	-70.50	0.00	86.25	15.75
LDEC006984-RA	glycine n-methyltransferase	-194.24	0.00	237.65	43.40
LDEC002300-RA	zinc finger protein 2 homolog	-38.81	0.01	47.49	8.68
LDEC015675-RA	leucine-rich repeat-containing protein ddb_g0290503 isoform x2	-47.48	0.00	58.11	10.64
LDEC003930-RA	serine threonine kinase nlk	-125.22	0.00	153.28	28.05
LDEC004536-RA	e3 ubiquitin ligase	-166.10	0.00	203.43	37.33
LDEC007173-RA	---NA---	-147.66	0.00	180.85	33.19
LDEC021977-RA	patched domain-containing protein 3	-104.49	0.00	128.04	23.55
LDEC002249-RA	dna excision repair protein ercc-6-like	-134.13	0.00	164.39	30.26
LDEC006463-RA	protein goliath-like	-95.69	0.00	117.32	21.63
LDEC022169-RA	glutamate ionotropic kainate 3 (glur7)	-51.78	0.00	63.49	11.71
LDEC009691-RA	wd repeat-containing protein 81 isoform x1	-55.08	0.00	67.60	12.51
LDEC001833-RA	zinc finger protein 845-like	-41.33	0.00	50.71	9.39
LDEC016706-RA	oxidative stress-responsive serine-rich protein 1	-60.23	0.00	73.93	13.70
LDEC004771-RA	carboxyl choline esterase	-44.33	0.00	54.43	10.10
LDEC009197-RA	lipopolysaccharide-induced tumor necrosis factor-alpha factor homolog	-73.95	0.00	90.81	16.86
LDEC003747-RA	4-coumarate-- ligase 1-like	-58.54	0.00	71.91	13.38
LDEC007212-RA	nfx1-type zinc finger-containing protein 1-like isoform x1	-71.35	0.00	87.70	16.35
LDEC013100-RA	gem-associated protein 2	-48.01	0.00	59.03	11.02
LDEC015042-RA	glutathione s-transferase theta-1-like isoform x2	-61.85	0.00	76.05	14.20
LDEC020860-RA	nuclear excision repair protein rad23	-117.61	0.00	144.61	27.00
LDEC008000-RA	PREDICTED: uncharacterized protein KIAA1109	-101.52	0.00	124.86	23.34
LDEC002745-RA	ras-interacting protein rip3-like isoform x1	-43.47	0.00	53.48	10.00
LDEC005367-RA	grip and coiled-coil domain-containing protein 2-like	-179.30	0.00	220.70	41.39
LDEC003340-RA	ubiquitin carboxyl-terminal hydrolase 47 isoform x1	-332.28	0.00	409.08	76.80
LDEC021904-RA	transmembrane protein 127-like	-56.25	0.00	69.26	13.01
LDEC019024-RA	zinc finger protein 271-like	-54.35	0.00	66.99	12.65
LDEC002694-RA	wd repeat-containing protein cg11141	-92.61	0.00	114.21	21.60
LDEC021544-RA	serine incorporator	-117.42	0.00	144.82	27.40
LDEC015739-RA	arfaptin-2 isoform x1	-44.56	0.00	54.96	10.41
LDEC022799-RA	reverse partial	-144.89	0.00	178.86	33.98
LDEC018513-RA	protein fam134c-like isoform x1	-121.23	0.00	149.67	28.44
LDEC001810-RA	zinc finger	-50.97	0.00	62.92	11.96
LDEC002109-RA	upf0183 protein cg7083 isoform x1	-65.33	0.00	80.66	15.33
LDEC016864-RA	pleckstrin homology-like domain family b member 1	-59.36	0.00	73.33	13.97
LDEC013716-RA	isocitrate dehydrogenase	-284.68	0.00	351.96	67.28
LDEC015930-RA	protein gdap2 homolog	-406.59	0.00	502.94	96.35

LDEC015039-RA	insulin-like growth factor-binding protein complex acid labile partial	-85.16	0.00	105.36	20.20
LDEC009944-RA	cytochrome b-c1 complex subunit mitochondrial-like	-40.67	0.00	50.33	9.66
LDEC005735-RA	calcium release-activated calcium channel protein 1-like	-113.43	0.00	140.39	26.96
LDEC001179-RA	protocadherin beta-15-like	-248.71	0.00	307.83	59.12
LDEC021825-RA	---NA---	-137.57	0.00	170.34	32.77
LDEC007102-RA	leucine-rich repeat-containing protein 15-like	-54.22	0.00	67.14	12.92
LDEC017884-RA	hypothetical protein G5I_14800	-155.22	0.00	192.28	37.06
LDEC015590-RA	protein cnppd1-like	-122.69	0.00	152.11	29.41
LDEC007081-RA	uba-like domain-containing protein 2	-187.01	0.00	231.95	44.94
LDEC005245-RA	helicase mov-10-	-166.26	0.00	206.26	39.99
LDEC008991-RA	ankyrin repeat and sterile alpha motif domain-containing protein 1b	-57.84	0.00	71.77	13.93
LDEC016723-RA	protein ctla-2-alpha	-528.36	0.00	656.00	127.64
LDEC004637-RA	ribosomal protein s6 kinase alpha-	-123.67	0.00	153.56	29.89
LDEC007904-RA	protein dopey-1 homolog	-85.12	0.00	105.82	20.70
LDEC005500-RA	vacuolar protein sorting-associated protein 13d	-53.64	0.00	66.71	13.07
LDEC003182-RA	e3 ubiquitin-protein ligase rnf8-like	-59.73	0.00	74.36	14.62
LDEC009472-RA	low quality protein: huntingtin-like	-127.51	0.00	158.76	31.25
LDEC008571-RA	ubiquitin-conjugating enzyme e2 w	-77.07	0.00	95.98	18.91
LDEC007664-RA	PREDICTED: uncharacterized protein LOC657045	-392.84	0.00	489.35	96.50
LDEC011561-RA	ectopic p granules protein 5 homolog	-60.91	0.00	75.91	15.00
LDEC010559-RA	rrna-processing protein utp23 homolog	-47.12	0.00	58.75	11.63
LDEC005521-RA	protein takeout-like	-199.44	0.00	248.69	49.25
LDEC010124-RA	factor viii intron 22	-65.12	0.00	81.22	16.10
LDEC019310-RA	yorkie homolog	-174.69	0.00	217.90	43.21
LDEC024116-RA	very long-chain specific acyl- mitochondrial	-56.04	0.00	69.93	13.89
LDEC007992-RA	low quality protein: mucin-17	-148.91	0.00	185.87	36.96
LDEC014976-RA	zinc finger protein 484-like	-49.83	0.00	62.25	12.42
LDEC003202-RA	wash complex subunit fam21a	-62.97	0.00	78.78	15.81
LDEC017254-RA	phosphoglucomutase-2	-44.39	0.00	55.56	11.17
LDEC001178-RA	PREDICTED: uncharacterized protein LOC656855 isoform X3	-1818.08	0.00	2276.22	458.14
LDEC007829-RA	transcriptional protein swt1	-47.52	0.00	59.49	11.98
LDEC007613-RA	transposable element tc3 transposase	-288.89	0.00	361.73	72.84
LDEC001106-RA	hypothetical protein TcasGA2_TC004643	-85.55	0.00	107.13	21.58
LDEC021174-RA	low-density lipoprotein receptor-related protein 6	-114.91	0.00	143.90	28.99
LDEC006797-RA	tbc1 domain family member 16	-59.93	0.00	75.06	15.14
LDEC008866-RA	chromobox protein partial	-55.39	0.00	69.40	14.01
LDEC015914-RA	superkiller viralicidic activity 2-like 2-like isoform x2	-67.32	0.00	84.37	17.05
LDEC007754-RA	PREDICTED: uncharacterized protein LOC100141968	-91.82	0.00	115.13	23.30
LDEC006016-RA	probable basic-leucine zipper transcription factor q-like isoform x4	-46.48	0.00	58.29	11.80
LDEC003977-RA	homeobox protein nk-	-162.03	0.00	203.21	41.18
LDEC010371-RA	vacuolar protein sorting-associated protein 33a	-64.15	0.00	80.48	16.33
LDEC010443-RA	tetraspanin isoform a	-318.29	0.00	399.31	81.02

LDEC008234-RA	three prime repair exonuclease 1	-69.10	0.00	86.71	17.61
LDEC003776-RA	protein szt2-like isoform x1	-176.94	0.00	222.15	45.20
LDEC004155-RA	forkhead box protein p4	-101.98	0.00	128.08	26.10
LDEC010792-RA	facilitated trehalose transporter tret1-2 homolog	-135.78	0.00	170.65	34.88
LDEC024320-RA	lipase 3	-55.74	0.00	70.07	14.33
LDEC003544-RA	was protein family homolog 1	-47.60	0.00	59.85	12.24
LDEC009490-RA	cgmp-dependent protein kinase	-39.91	0.01	50.18	10.27
LDEC005973-RA	exosome component 10	-166.40	0.00	209.26	42.87
LDEC011560-RA	ectopic p granules protein 5 homolog	-44.75	0.00	56.31	11.56
LDEC019475-RA	protein lin-9 homolog isoform x2	-62.24	0.00	78.35	16.12
LDEC015092-RA	protein pbdc1	-57.12	0.00	71.91	14.79
LDEC005612-RA	comm domain-containing protein 3	-51.12	0.00	64.38	13.26
LDEC018260-RA	ubiquitin carboxyl-terminal hydrolase 8	-40.67	0.01	51.25	10.58
LDEC018112-RA	PREDICTED: uncharacterized protein LOC103313387, partial	-69.45	0.00	87.52	18.07
LDEC007932-RA	zinc finger protein 729-like isoform x1	-102.93	0.00	129.74	26.81
LDEC010432-RA	phospholipase d3-like isoform x2	-64.73	0.00	81.61	16.88
LDEC014748-RA	tumor necrosis factor induced protein	-55.96	0.00	70.60	14.64
LDEC009779-RA	isoform a	-200.50	0.00	253.01	52.51
LDEC009713-RA	fh1 fh2 domain-containing protein 3 isoform x4	-58.17	0.00	73.40	15.23
LDEC012691-RA	ddb1- and cul4-associated factor 6- partial	-78.72	0.00	99.34	20.62
LDEC005615-RA	zinc finger fyve domain-containing protein 9	-41.77	0.00	52.73	10.96
LDEC014513-RA	nuclear hormone receptor ftz-	-69.94	0.00	88.30	18.36
LDEC002381-RA	PREDICTED: uncharacterized protein LOC664340	-87.35	0.00	110.35	23.00
LDEC007871-RA	zinc finger protein 277	-45.56	0.00	57.62	12.05
LDEC008448-RA	zinc finger protein 271-like	-39.60	0.01	50.08	10.48
LDEC016586-RA	kynurenine alpha-aminoadipate mitochondrial-like	-568.56	0.00	719.10	150.54
LDEC011513-RA	4-coumarate-- ligase 1-like	-221.63	0.00	280.33	58.69
LDEC003145-RA	androgen-induced protein 1	-98.98	0.00	125.21	26.23
LDEC015381-RA	elongation factor tu gtp-binding domain-containing protein 1	-68.14	0.00	86.25	18.11
LDEC007575-RA	e3 ubiquitin-protein ligase ubr4 isoform x1	-621.48	0.00	786.91	165.43
LDEC003701-RA	retinal protein	-111.76	0.00	141.56	29.80
LDEC000269-RA	spatacsin isoform x1	-64.40	0.00	81.65	17.25
LDEC006753-RA	zinc finger protein 271-like	-89.30	0.00	113.21	23.91
LDEC017229-RA	patatin-like phospholipase domain-containing protein 2-like	-212.71	0.00	269.68	56.97
LDEC020581-RA	m13 peptidase	-54.43	0.00	69.01	14.58
LDEC005947-RA	serine threonine-protein kinase pak 3 isoform x3	-164.51	0.00	208.66	44.15
LDEC022213-RA	g kinase-anchoring protein 1-like	-49.83	0.00	63.21	13.38
LDEC012866-RA	protein pellino isoform x1	-55.10	0.00	69.90	14.79
LDEC014856-RA	---NA---	-361.92	0.00	459.19	97.27
LDEC008073-RA	protein son-like isoform x1	-42.27	0.00	53.65	11.38
LDEC007823-RA	PREDICTED: uncharacterized protein LOC103312333 isoform X2	-296.03	0.00	375.92	79.89
LDEC009473-RA	huntingtin isoform x2	-62.81	0.00	79.81	17.00

LDEC008001-RA	PREDICTED: uncharacterized protein KIAA1109	-104.01	0.00	132.18	28.17
LDEC014895-RA	autophagy-related protein 2 homolog a isoform x2	-54.75	0.00	69.58	14.83
LDEC018997-RA	protein capicua homolog	-41.86	0.00	53.30	11.44
LDEC021725-RA	juvenile hormone esterase	-91.98	0.00	117.14	25.16
LDEC003267-RA	thyrotropin-releasing hormone receptor isoform x3	-77.21	0.00	98.35	21.14
LDEC008992-RA	ankyrin repeat and sam domain-containing protein 1a-like	-68.88	0.00	87.73	18.86
LDEC000584-RA	restin homolog isoform x1	-130.94	0.00	166.83	35.89
LDEC011168-RA	btb poz domain-containing protein 7 isoform x1	-64.09	0.00	81.72	17.63
LDEC021672-RA	sodium- and chloride-dependent gaba transporter ine	-53.77	0.00	68.59	14.81
LDEC007728-RA	pleiotrophic factor-alpha-1	-156.83	0.00	200.13	43.31
LDEC008913-RA	general transcription factor iie subunit 1	-49.94	0.00	63.74	13.80
LDEC001099-RA	PREDICTED: uncharacterized protein LOC660397	-83.23	0.00	106.24	23.01
LDEC011065-RA	protein xmas-2	-155.82	0.00	199.07	43.25
LDEC022492-RA	u2 snrnp-associated surp motif-containing protein	-54.87	0.00	70.11	15.23
LDEC015023-RA	tyrosine-protein phosphatase corkscrew isoform x2	-167.22	0.00	213.65	46.43
LDEC007437-RA	cklf-like marvel transmembrane domain-containing protein 4-like	-122.23	0.00	156.28	34.05
LDEC003612-RA	werner syndrome helicase	-51.72	0.00	66.15	14.43
LDEC020160-RA	histone-lysine n-methyltransferase setmar	-119.97	0.00	153.49	33.52
LDEC005616-RA	zinc finger fyve domain-containing protein 9-like	-55.09	0.00	70.53	15.45
LDEC010245-RA	polynucleotide kinase- 3 -phosphatase	-209.35	0.00	268.30	58.94
LDEC015706-RA	fat-like cadherin-related tumor suppressor homolog	-170.05	0.00	217.97	47.93
LDEC012173-RA	slit-robo rho gtpase-activating protein 1-like isoform x3	-73.08	0.00	93.68	20.60
LDEC010804-RA	proteoglycan 4-like	-125.08	0.00	160.35	35.28
LDEC001566-RA	PREDICTED: uncharacterized protein CG10915 isoform X1	-113.17	0.00	145.21	32.04
LDEC003847-RA	alpha-aminoadipic semialdehyde mitochondrial isoform x1	-199.92	0.00	256.58	56.66
LDEC011365-RA	hypothetical protein YQE_09758, partial	-50.50	0.00	64.84	14.33
LDEC014322-RA	zinc finger protein 271-like	-45.55	0.00	58.50	12.95
LDEC005816-RA	serine threonine-protein phosphatase 4 regulatory subunit 3 isoform x1	-66.35	0.00	85.22	18.88
LDEC006935-RA	integral membrane protein gpr155	-104.82	0.00	134.70	29.87
LDEC008023-RA	hermansky-pudlak syndrome 3 protein	-61.59	0.00	79.20	17.61
LDEC001728-RA	peptidoglycan-recognition protein sc2-like	-52.00	0.00	66.89	14.89
LDEC012626-RA	hypothetical protein TcasGA2_TC005619	-52.19	0.00	67.14	14.95
LDEC020446-RA	lysine-specific demethylase 4b-like isoform x1	-306.26	0.00	394.00	87.75
LDEC007888-RA	vacuolar protein sorting-associated protein 53 homolog	-85.34	0.00	109.85	24.51
LDEC016854-RA	---NA---	-42.56	0.00	54.78	12.23
LDEC007345-RA	low quality protein: leucine-rich repeat-containing protein ddb_g0290503	-163.90	0.00	211.00	47.10
LDEC002399-RA	polyprenol reductase-like isoform x1	-54.40	0.00	70.07	15.67
LDEC017501-RA	tryptophan oxygenase	-165.67	0.00	213.44	47.77
LDEC000634-RA	isoform a	-177.90	0.00	229.26	51.36
LDEC001898-RA	serine threonine-protein kinase wnk1-like isoform x1	-519.44	0.00	669.52	150.08
LDEC001063-RA	protein unc-79 homolog	-117.27	0.00	151.19	33.92
LDEC020748-RA	citron rho-interacting kinase	-103.68	0.00	133.67	29.99

LDEC007820-RA	rna-directed dna polymerase from mobile element jockey-like	-134.18	0.00	173.06	38.88
LDEC019548-RA	trafficking kinesin-binding protein milt-like	-79.28	0.00	102.31	23.03
LDEC000984-RA	leucine-rich repeat-containing protein 58-like	-127.89	0.00	165.06	37.18
LDEC003625-RA	rho gtpase activating protein 15	-85.51	0.00	110.38	24.87
LDEC013919-RA	cyclomaltodextrin partial	-159.54	0.00	206.01	46.47
LDEC001019-RA	e3 ubiquitin-protein ligase rnf220-like isoform x2	-62.03	0.00	80.12	18.09
LDEC006216-RA	venom carboxylesterase-6	-48.14	0.00	62.18	14.05
LDEC023675-RA	cytochrome p450	-126.35	0.00	163.26	36.91
LDEC010296-RA	peptidyl-prolyl cis-trans isomerase g-like	-64.73	0.00	83.66	18.93
LDEC001764-RA	aminopeptidase n	-75.89	0.00	98.10	22.21
LDEC006488-RA	PREDICTED: uncharacterized protein LOC658498	-88.32	0.00	114.17	25.85
LDEC023320-RA	c-1-tetrahydrofolate cytoplasmic isoform x2	-265.40	0.00	343.22	77.82
LDEC007802-RA	apoptosis 1 inhibitor	-728.09	0.00	941.89	213.80
LDEC021144-RA	magnesium transporter nipa2 isoform x2	-45.51	0.00	58.89	13.38
LDEC014841-RA	longitudinals lacking	-166.94	0.00	216.10	49.15
LDEC015401-RA	low quality protein: tuberin-like	-58.33	0.00	75.52	17.19
LDEC009063-RA	zinc finger	-76.98	0.00	99.73	22.75
LDEC004971-RA	enolase-phosphatase e1-like	-48.40	0.00	62.71	14.31
LDEC012500-RA	venom dipeptidyl peptidase 4	-59.02	0.00	76.48	17.46
LDEC014925-RA	polycomb group protein psc-like	-129.12	0.00	167.33	38.21
LDEC001429-RA	protein beta isoform-like isoform x32	-535.69	0.00	694.22	158.53
LDEC018222-RA	cytochrome p450 4c1	-75.64	0.00	98.10	22.46
LDEC003562-RA	kat8 regulatory nsl complex subunit 3 isoform x2	-64.95	0.00	84.27	19.32
LDEC003664-RA	fasciclin-2 isoform x3	-43.96	0.00	57.05	13.09
LDEC010523-RA	extracellular sulfatase sulf-1-like protein	-53.62	0.00	69.58	15.96
LDEC007210-RA	nfx1-type zinc finger-containing protein 1-like isoform x2	-45.15	0.00	58.61	13.45
LDEC007464-RA	rho gtpase-activating protein 18 isoform x2	-80.38	0.00	104.33	23.95
LDEC010340-RA	charged multivesicular body protein 7	-64.47	0.00	83.77	19.30
LDEC008209-RA	PREDICTED: uncharacterized protein LOC103312207	-51.34	0.00	66.75	15.41
LDEC000987-RA	bromo adjacent homology domain containing	-291.38	0.00	379.14	87.76
LDEC001765-RA	aminopeptidase n	-100.22	0.00	130.52	30.30
LDEC012531-RA	general transcription factor 3c polypeptide	-93.50	0.00	121.78	28.28
LDEC006754-RA	beta-hexosaminidase b	-56.33	0.00	73.40	17.07
LDEC008226-RA	cyclic gmp-amp synthase	-79.91	0.00	104.15	24.24
LDEC012581-RA	dna methyltransferase 1	-77.82	0.00	101.46	23.65
LDEC013130-RA	armadillo repeat-containing protein 8	-50.37	0.00	65.68	15.31
LDEC010455-RA	leucine-rich repeat neuronal protein 1-like	-195.16	0.00	254.56	59.40
LDEC002523-RA	transposable element tc3 transposase	-85.22	0.00	111.16	25.95
LDEC020176-RA	cytochrome p450 9z4	-537.79	0.00	702.01	164.22
LDEC021242-RA	immunoglobulin-binding protein 1-like	-109.61	0.00	143.08	33.48
LDEC019100-RA	bifunctional methylenetetrahydrofolate dehydrogenase mitochondrial isoform x3	-104.05	0.00	135.86	31.81
LDEC005787-RA	muscle-specific protein 300	-55.43	0.00	72.44	17.02

LDEC008750-RA	histone deacetylase 7	-63.47	0.00	82.96	19.49
LDEC001097-RA	hypothetical protein YQE_06610, partial	-67.25	0.00	87.91	20.66
LDEC013070-RA	syntaxin-binding protein 5 isoform x3	-148.81	0.00	194.61	45.80
LDEC000677-RA	nuclear rna export factor 2-like	-57.55	0.00	75.31	17.76
LDEC007385-RA	nedd4-binding protein 2	-536.99	0.00	702.86	165.87
LDEC020235-RA	fru-related protein	-41.57	0.01	54.43	12.86
LDEC015194-RA	lysosomal aspartic protease	-267.54	0.00	350.47	82.94
LDEC007776-RA	trna (guanine -n2)-methyltransferase homolog	-44.28	0.00	58.08	13.80
LDEC018934-RA	juvenile hormone esterase isoform a	-132.70	0.00	174.05	41.35
LDEC015271-RA	syntaxin-1a-like isoform x2	-53.92	0.00	70.75	16.82
LDEC008014-RA	rilp-like protein homolog isoform x1	-60.82	0.00	79.81	18.99
LDEC007779-RA	telo2-interacting protein 1 homolog	-49.95	0.00	65.54	15.60
LDEC007905-RA	protein dopey-1 homolog	-64.53	0.00	84.69	20.16
LDEC005617-RA	guanine nucleotide-binding protein subunit beta-like protein 1	-64.20	0.00	84.27	20.06
LDEC006677-RA	membrane magnesium transporter 1	-50.34	0.00	66.07	15.73
LDEC007574-RA	f-box lrr-repeat protein 6 isoform x1	-111.28	0.00	146.06	34.78
LDEC001831-RA	zinc finger protein 845-like	-74.67	0.00	98.03	23.36
LDEC006157-RA	nucleolar complex protein 3 homolog	-318.05	0.00	417.72	99.66
LDEC006105-RA	mitochondrial isoform x3	-57.75	0.00	75.88	18.13
LDEC005598-RA	phosphoribosylaminoimidazole carboxylase	-413.27	0.00	543.25	129.98
LDEC001258-RA	pyruvate dehydrogenase (acetyl-transferring) mitochondrial	-100.73	0.00	132.47	31.73
LDEC016650-RA	probable nucleolar gtp-binding protein 1	-329.57	0.00	433.43	103.86
LDEC003804-RA	atp-binding cassette sub-family g member 1 isoform x2	-139.07	0.00	183.00	43.94
LDEC001062-RA	conserved oligomeric golgi complex subunit 2	-45.42	0.00	59.77	14.35
LDEC012241-RA	endothelin-converting enzyme 1 isoform x2	-123.41	0.00	162.44	39.03
LDEC020227-RA	mediator of rna polymerase ii transcription subunit 1	-115.81	0.00	152.60	36.79
LDEC012541-RA	n-acetylneuraminate lyase-like	-51.91	0.00	68.41	16.50
LDEC001683-RA	ccr4-not transcription complex subunit 6-like isoform x1	-67.70	0.00	89.22	21.52
LDEC007715-RA	rho gtpase-activating protein 21 isoform x7	-87.49	0.00	115.34	27.84
LDEC007466-RA	protein fam192a isoform x2	-52.57	0.00	69.29	16.73
LDEC022662-RA	nadh dehydrogenase subunit 1	-830.99	0.00	1095.59	264.60
LDEC007972-RA	titin isoform x1	-372.79	0.00	491.75	118.96
LDEC000476-RA	inositol-pentakisphosphate 2-kinase isoform x1	-58.00	0.00	76.51	18.51
LDEC013749-RA	scavenger receptor protein	-378.47	0.00	499.29	120.82
LDEC005426-RA	phosphatidylinositol-binding clathrin assembly protein lap-like isoform x2	-387.01	0.00	510.93	123.92
LDEC017480-RA	protein ariadne-2	-92.94	0.00	122.73	29.80
LDEC002960-RA	g2 mitotic-specific cyclin-a	-45.28	0.00	59.81	14.53
LDEC019779-RA	c-1-tetrahydrofolate cytoplasmic isoform x2	-2030.55	0.00	2681.87	651.32
LDEC009118-RA	nuclear factor nf-kappa-b p110 subunit isoform x2	-89.81	0.00	118.63	28.82
LDEC011109-RA	protein argonaute-2 isoform x1	-94.67	0.00	125.14	30.47
LDEC016364-RA	protein ddi1 homolog 2 isoform x1	-233.66	0.00	308.89	75.23
LDEC000815-RA	low quality protein: inhibitor of growth protein 3-like	-53.41	0.00	70.64	17.23

LDEC009122-RA	relish	-76.86	0.00	101.71	24.85
LDEC006020-RA	cytochrome p450 partial	-164.99	0.00	218.40	53.41
LDEC001658-RA	zinc finger protein 271-like	-44.03	0.01	58.32	14.30
LDEC016174-RA	phosphatidylcholine:ceramide cholinephosphotransferase 2 isoform x2	-295.37	0.00	391.31	95.95
LDEC014455-RA	2-hydroxyacylsphingosine 1-beta-galactosyltransferase-like	-86.40	0.00	114.49	28.09
LDEC000177-RA	protein arginine n-methyltransferase 9 isoform x1	-47.43	0.00	62.85	15.43
LDEC003342-RA	nucleolin-like isoform x2	-68.69	0.00	91.10	22.40
LDEC008144-RA	cytochrome p450 6a2	-979.66	0.00	1299.30	319.63
LDEC016290-RA	monocarboxylate transporter 14-like	-93.94	0.00	124.68	30.74
LDEC013830-RA	mediator of rna polymerase ii transcription subunit 29	-66.91	0.00	88.87	21.96
LDEC014285-RA	sh3 domain-containing kinase-binding protein 1 isoform x1	-216.29	0.00	287.51	71.23
LDEC020409-RA	dentin sialophospho	-236.01	0.00	313.84	77.84
LDEC008408-RA	glycoside hydrolase family 1	-76.23	0.00	101.39	25.16
LDEC007979-RA	zinc phosphodiesterase	-57.75	0.00	76.83	19.09
LDEC001355-RA	serine threonine-protein kinase haspin-like protein	-155.09	0.00	206.47	51.37
LDEC022301-RA	upf0505 protein c16orf62 homolog	-129.53	0.00	172.53	43.00
LDEC011811-RA	vacuolar protein sorting-associated protein 13b	-84.30	0.00	112.29	28.00
LDEC004234-RA	hypothetical protein TcasGA2_TC004772	-48.90	0.00	65.15	16.25
LDEC008335-RA	venom dipeptidyl peptidase 4 isoform x1	-161.17	0.00	214.82	53.66
LDEC017890-RA	isocitrate dehydrogenase	-342.85	0.00	457.00	114.15
LDEC001096-RA	hypothetical protein TcasGA2_TC004643	-199.00	0.00	265.29	66.28
LDEC008610-RA	protein phosphatase 1 regulatory subunit 12a	-53.08	0.00	70.78	17.71
LDEC004830-RA	xk-related protein 7-like	-143.19	0.00	191.07	47.89
LDEC002245-RA	atp-binding cassette sub-family a member 3 isoform x1	-124.55	0.00	166.27	41.72
LDEC003698-RA	sex-lethal homolog isoform x5	-165.35	0.00	220.77	55.42
LDEC002775-RA	S-antigen protein, putative	-421.44	0.00	562.82	141.38
LDEC015714-RA	det1- and ddb1-associated protein 1-like	-50.10	0.00	66.92	16.82
LDEC007674-RA	blastoderm-specific protein 25d	-94.91	0.00	126.95	32.04
LDEC006825-RA	muscle m-line assembly protein unc-89-like	-81.24	0.00	108.68	27.44
LDEC007520-RA	atp-dependent helicase brm isoform x1	-59.60	0.00	79.74	20.14
LDEC017722-RA	polyadenylate-binding protein-interacting protein 1	-77.05	0.00	103.09	26.04
LDEC002992-RA	glycoprotein-n-acetylgalactosamine 3-beta-galactosyltransferase 1	-105.54	0.00	141.28	35.74
LDEC015926-RA	n-alpha-acetyltransferase 20	-105.42	0.00	141.14	35.72
LDEC014939-RA	telomere-associated protein rif1-like	-63.28	0.00	84.73	21.44
LDEC000129-RA	facilitated trehalose transporter tret1	-65.83	0.00	88.16	22.32
LDEC002675-RA	serine threonine-protein kinase smg1	-155.88	0.00	208.77	52.89
LDEC010492-RA	serine proteinase	-176.53	0.00	236.62	60.09
LDEC011057-RA	dis3-like exonuclease 2-like isoform x1	-179.65	0.00	240.83	61.19
LDEC012229-RA	hypothetical protein TcasGA2_TC001876	-65.16	0.00	87.45	22.29
LDEC018559-RA	methyltransferase-like protein 6	-89.08	0.00	119.55	30.47
LDEC014818-RA	huntingtin interacting protein	-107.79	0.00	144.71	36.93
LDEC011381-RA	neutral alpha-glucosidase ab	-439.26	0.00	589.86	150.60

LDEC007835-RA	guanine nucleotide-binding 1	-45.49	0.01	61.08	15.60
LDEC017979-RA	phosphofurin acidic cluster sorting protein 2 isoform x3	-98.45	0.00	132.22	33.76
LDEC010670-RA	phosphatidate phosphatase lpin3	-283.36	0.00	380.63	97.27
LDEC016134-RA	phosphoglycolate phosphatase 2-like	-49.48	0.00	66.50	17.02
LDEC024223-RA	c-1-tetrahydrofolate cytoplasmic	-234.89	0.00	315.79	80.90
LDEC000942-RA	serine threonine-protein kinase rio1-like	-107.15	0.00	144.08	36.93
LDEC022326-RA	phosphatidylinositol 5-phosphate 4-kinase type-2 alpha	-46.71	0.00	62.92	16.21
LDEC002736-RA	zinc finger protein 271-like	-319.81	0.00	430.88	111.07
LDEC003738-RA	PREDICTED: uncharacterized protein C16orf52 homolog A	-44.07	0.01	59.39	15.31
LDEC005887-RA	adenylosuccinate lyase	-240.28	0.00	323.82	83.55
LDEC024548-RA	cytochrome p450 6a2	-1547.33	0.00	2085.78	538.45
LDEC010970-RA	max-binding protein mnt	-113.20	0.00	152.64	39.44
LDEC014988-RA	cytosolic carboxypeptidase-like protein 5 isoform x1	-56.05	0.00	75.59	19.55
LDEC013061-RA	fanconi-associated nuclease 1-like	-62.04	0.00	83.73	21.69
LDEC017502-RA	tryptophan oxygenase	-103.36	0.00	139.62	36.26
LDEC005599-RA	amidophosphoribosyltransferase	-323.81	0.00	437.46	113.65
LDEC004369-RA	liprin-beta-1 isoform x1	-191.06	0.00	258.28	67.22
LDEC019041-RA	inositol polyphosphate 5-phosphatase ocrl-1	-83.81	0.00	113.32	29.51
LDEC007277-RA	protein kinase dc2	-125.67	0.00	170.05	44.38
LDEC011745-RA	arginine-glutamic acid dipeptide repeats protein	-107.65	0.00	145.67	38.02
LDEC000224-RA	c19orf22 homolog	-48.38	0.00	65.47	17.09
LDEC001565-RA	major facilitator superfamily domain-containing protein 10	-51.98	0.00	70.36	18.38
LDEC006590-RA	thyroid adenoma-associated protein homolog	-84.49	0.00	114.38	29.89
LDEC001064-RA	ankyrin unc44	-107.25	0.00	145.21	37.96
LDEC010299-RA	arrestin domain-containing protein 3	-67.02	0.00	90.74	23.72
LDEC002679-RA	upf0364 protein c6orf211 homolog	-223.01	0.00	302.27	79.26
LDEC007263-RA	cell differentiation protein rcd1 homolog	-109.84	0.00	148.89	39.05
LDEC011060-RA	peptidyl-prolyl cis-trans mitochondrial	-229.14	0.00	310.66	81.52
LDEC004681-RA	inosine-5 -monophosphate dehydrogenase	-100.41	0.00	136.18	35.78
LDEC019106-RA	exportin domain-containing partial	-49.23	0.00	66.78	17.55
LDEC007388-RA	ammecr1-like protein	-318.80	0.00	432.51	113.71
LDEC013706-RA	low quality protein: membralin	-83.18	0.00	112.86	29.68
LDEC017508-RA	zinc finger protein 264-like isoform x1	-122.19	0.00	165.84	43.65
LDEC001065-RA	transcription initiation factor iia subunit 1	-74.13	0.00	100.65	26.52
LDEC005644-RA	cytochrome p450-like protein	-70.38	0.00	95.55	25.18
LDEC011750-RA	two pore calcium channel protein 1-like	-158.13	0.00	214.71	56.59
LDEC003341-RA	ubiquitin carboxyl-terminal hydrolase 47 isoform x1	-227.50	0.00	308.96	81.46
LDEC007554-RA	hypothetical protein YQE_11513, partial	-107.79	0.00	146.52	38.73
LDEC003293-RA	serine threonine-protein phosphatase 2b catalytic subunit 2-like isoform x1	-423.09	0.00	575.27	152.19
LDEC013455-RA	zinc transporter 1	-55.69	0.00	75.74	20.04
LDEC013822-RA	hypothetical protein D910_11774	-84.83	0.00	115.41	30.58
LDEC005977-RA	dcn1-like protein 1	-54.72	0.00	74.46	19.74

LDEC005912-RA	heparan-alpha-glucosaminide n-acetyltransferase-like isoform x1	-128.64	0.00	175.15	46.51
LDEC003894-RA	ubiquitin thioesterase trabid	-120.60	0.00	164.21	43.61
LDEC000530-RA	caspase nc-like	-86.25	0.00	117.46	31.22
LDEC011699-RA	vacuolar protein sorting-associated protein 11 homolog	-64.36	0.00	87.70	23.34
LDEC002170-RA	zinc finger mym-type protein 1	-59.88	0.00	81.61	21.73
LDEC002132-RA	semaphorin-1a isoform x3	-69.04	0.00	94.14	25.10
LDEC008670-RA	leucine-rich repeat-containing protein 24-like isoform x1	-146.69	0.00	200.17	53.48
LDEC014372-RA	multidrug resistance-associated protein 4-like isoform x1	-51.28	0.00	70.00	18.72
LDEC012781-RA	osteopetrosis-associated transmembrane protein 1	-67.16	0.00	91.77	24.60
LDEC016116-RA	dynactin subunit 3-like	-56.32	0.00	76.97	20.66
LDEC009685-RA	bag domain-containing protein samui isoform x1	-310.81	0.00	425.29	114.48
LDEC000747-RA	unknown	-60.76	0.00	83.20	22.44
LDEC021060-RA	polypeptide n-acetylgalactosaminyltransferase 1	-108.45	0.00	148.50	40.05
LDEC007228-RA	gastrula zinc finger protein	-66.65	0.00	91.27	24.62
LDEC017188-RA	---NA---	-158.83	0.00	217.55	58.71
LDEC012625-RA	PREDICTED: uncharacterized protein LOC100141760 isoform X2	-45.65	0.01	62.54	16.88
LDEC015642-RA	syntenin-1	-279.63	0.00	383.25	103.61
LDEC010549-RA	kruppel-like protein 1	-170.40	0.00	233.76	63.35
LDEC014717-RA	dystroglycan isoform x1	-64.93	0.00	89.08	24.14
LDEC003787-RA	gamma-interferon-inducible lysosomal thiol reductase-like isoform x2	-77.41	0.00	106.35	28.94
LDEC007982-RA	nucleolar complex protein 2 homolog	-159.83	0.00	219.60	59.77
LDEC019268-RA	histone acetyltransferase kat6b isoform x1	-117.53	0.00	161.52	44.00
LDEC023021-RA	protein henna	-59.93	0.00	82.39	22.46
LDEC012277-RA	lian-aa1 retrotransposon protein	-66.05	0.00	90.88	24.83
LDEC000878-RA	ras-related protein rab-39b	-113.24	0.00	155.86	42.62
LDEC015948-RA	mothers against decapentaplegic homolog 4 isoform x3	-120.76	0.00	166.23	45.47
LDEC010491-RA	serine protease s1a- partial	-130.68	0.00	179.93	49.25
LDEC001970-RA	fatty acyl- reductase cg5065 isoform x1	-284.66	0.00	391.95	107.29
LDEC010903-RA	esterase	-98.88	0.00	136.15	37.27
LDEC014020-RA	serine threonine-protein kinase 11-interacting protein isoform x1	-46.49	0.01	64.02	17.53
LDEC002122-RA	ras-related protein rab-2a	-96.71	0.00	133.21	36.50
LDEC004319-RA	mucin-17-like isoform x1	-214.85	0.00	296.01	81.15
LDEC024272-RA	sphingolipid delta -desaturase des1	-75.31	0.00	103.77	28.46
LDEC004130-RA	ileal sodium bile acid cotransporter-like isoform x1	-48.31	0.00	66.57	18.26
LDEC018560-RA	polynucleotide 5 -hydroxyl-kinase nol9	-64.92	0.00	89.47	24.55
LDEC005482-RA	autophagy-related protein 13 homolog isoform x1	-170.52	0.00	235.10	64.58
LDEC014763-RA	probable phospholipid-transporting atpase ia isoform x1	-50.85	0.00	70.14	19.30
LDEC017470-RA	protein dennd6a isoform x1	-78.88	0.00	108.83	29.95
LDEC011071-RA	exonuclease gor-like protein	-113.16	0.00	156.14	42.98
LDEC013023-RA	ctd small phosphatase-like protein 2	-85.35	0.00	117.82	32.46
LDEC002019-RA	protein lsm14 homolog b isoform x4	-68.50	0.00	94.63	26.14
LDEC006953-RA	ubiquitin conjugation factor e4 b	-62.25	0.00	86.07	23.82

LDEC005488-RA	hermansky-pudlak syndrome 4 isoform b	-51.59	0.00	71.35	19.76
LDEC004172-RA	cell cycle control protein 50a	-80.71	0.00	111.69	30.99
LDEC017926-RA	protein winged eye isoform x1	-72.18	0.00	99.91	27.73
LDEC018606-RA	zinc finger protein 433	-50.64	0.00	70.11	19.47
LDEC016579-RA	zinc finger ccch domain-containing protein 13	-311.17	0.00	430.88	119.71
LDEC004182-RA	short gastrulation precursor	-90.82	0.00	125.85	35.03
LDEC011813-RA	negative elongation factor a	-51.41	0.00	71.24	19.83
LDEC016836-RA	polya-binding protein interacting	-135.16	0.00	187.32	52.16
LDEC005817-RA	serine threonine-protein phosphatase 4 regulatory subunit 3	-50.54	0.00	70.11	19.56
LDEC009947-RA	protein diaphanous isoform x3	-116.16	0.00	161.31	45.15
LDEC002144-RA	microtubule-associated protein futsch isoform x1	-140.01	0.00	194.68	54.67
LDEC001130-RA	monocarboxylate transporter 5	-47.28	0.01	65.79	18.51
LDEC003512-RA	flj37770-like protein	-52.66	0.00	73.29	20.64
LDEC008105-RA	protein painting of fourth isoform x1	-55.37	0.00	77.08	21.71
LDEC004450-RA	phospholipase a2 inhibitor-like	-81.55	0.00	113.53	31.98
LDEC001539-RA	PREDICTED: uncharacterized protein LOC103314900	-48.56	0.00	67.67	19.11
LDEC017391-RA	cullin-2	-190.28	0.00	265.15	74.87
LDEC010405-RA	maternal protein pumilio isoform x5	-206.76	0.00	288.26	81.50
LDEC002757-RA	striatin-interacting protein 1 isoform x1	-56.24	0.00	78.43	22.19
LDEC000850-RA	fatty acid-biding protein	-121.69	0.00	169.80	48.12
LDEC000519-RA	ubiquitin carboxyl-terminal hydrolase 2-like isoform x2	-100.61	0.00	140.47	39.86
LDEC022062-RA	protein fam107b isoform x2	-67.92	0.00	94.85	26.92
LDEC016648-RA	rna-binding protein pno1	-123.25	0.00	172.21	48.96
LDEC000580-RA	agap007663-pa-like protein	-270.95	0.00	378.68	107.73
LDEC001335-RA	PREDICTED: uncharacterized protein LOC663962	-79.25	0.00	110.77	31.52
LDEC007973-RA	smoothelin-like protein 1 isoform x6	-196.56	0.00	274.88	78.32
LDEC018052-RA	nuclear pore complex protein nup50	-46.33	0.01	64.80	18.47
LDEC004154-RA	27 kda hemolymph	-107.90	0.00	150.98	43.08
LDEC021950-RA	zinc transporter 9 isoform x2	-49.24	0.00	68.94	19.70
LDEC010493-RA	serine protease s1a- partial	-123.57	0.00	173.02	49.46
LDEC003009-RA	suz domain-containing protein 1	-65.17	0.00	91.31	26.14
LDEC004484-RA	probable ribosome production factor 1	-46.92	0.01	65.76	18.84
LDEC022355-RA	traf2 and nck interacting tnik	-149.75	0.00	209.94	60.19
LDEC015081-RA	ornithine mitochondrial	-49.33	0.00	69.26	19.93
LDEC004616-RA	amino acid transporter	-104.78	0.00	147.22	42.45
LDEC009800-RA	tumor susceptibility gene 101 protein	-47.85	0.01	67.24	19.39
LDEC015375-RA	ras-related protein rab-1a	-80.27	0.00	112.83	32.56
LDEC018431-RA	rab3 gtpase-activating protein non-catalytic subunit	-151.93	0.00	213.62	61.68
LDEC016738-RA	seven in	-102.76	0.00	144.75	41.99
LDEC014668-RA	creb-regulated transcription coactivator 1-like isoform x1	-132.75	0.00	187.00	54.25
LDEC000770-RA	monocarboxylate transporter	-176.87	0.00	249.15	72.28
LDEC010458-RA	low-density lipoprotein receptor class a domain-containing protein 3	-82.77	0.00	116.61	33.84

LDEC007938-RA	leucine-rich repeat-containing protein 47-like	-93.40	0.00	131.72	38.33
LDEC000053-RA	tubulin-specific chaperone c	-260.00	0.00	366.72	106.72
LDEC012240-RA	fatty acyl- reductase cg5065-like	-100.14	0.00	141.24	41.10
LDEC009952-RA	suppression of tumorigenicity 1	-521.23	0.00	735.31	214.08
LDEC017324-RA	flj37770-like protein	-70.20	0.00	99.09	28.90
LDEC006694-RA	large proline-rich protein bag6	-51.58	0.00	72.83	21.25
LDEC005801-RA	multiple pdz domain protein	-103.61	0.00	146.30	42.69
LDEC013408-RA	transmembrane protein 70 mitochondrial	-53.31	0.00	75.31	22.00
LDEC020383-RA	rna-directed dna polymerase from mobile element jockey-like	-133.26	0.00	188.31	55.05
LDEC018746-RA	hypothetical protein YQE_08038, partial	-108.31	0.00	153.14	44.82
LDEC016498-RA	probable serine threonine-protein kinase ddb_g0282963	-131.54	0.00	186.19	54.65
LDEC007843-RA	cadherin 1	-118.46	0.00	167.79	49.32
LDEC019564-RA	e3 ubiquitin-protein ligase kcmf1-like	-58.16	0.00	82.42	24.26
LDEC005625-RA	rhomboid-related protein 3-like isoform 2	-57.13	0.00	81.04	23.91
LDEC013344-RA	ccr4-not transcription complex subunit 4	-164.82	0.00	233.83	69.00
LDEC024410-RA	calcium-independent phospholipase a2-gamma-like	-57.73	0.00	82.11	24.37
LDEC001543-RA	upf0518 protein agap011705	-85.24	0.00	121.25	36.01
LDEC016470-RA	repressor of rna polymerase iii transcription maf1 homolog	-202.75	0.00	288.50	85.75
LDEC007662-RA	3-ketodihydrosphingosine reductase	-57.11	0.00	81.33	24.22
LDEC001534-RA	cytokine receptor	-284.79	0.00	405.61	120.82
LDEC002599-RA	endophilin-b1 isoform x2	-54.02	0.00	76.94	22.92
LDEC008291-RA	acidic fibroblast growth factor intracellular-binding protein	-207.82	0.00	296.01	88.19
LDEC000642-RA	probable protein phosphatase 2c	-51.05	0.00	72.73	21.67
LDEC016193-RA	supervillin-like isoform x1	-123.63	0.00	176.46	52.83
LDEC015586-RA	selenoprotein m-like	-70.10	0.00	100.08	29.99
LDEC004812-RA	rna-directed dna polymerase from mobile element jockey-like	-68.28	0.00	97.50	29.22
LDEC002993-RA	protein hid1-like isoform x2	-62.49	0.00	89.26	26.77
LDEC010226-RA	phytanoyl- dioxygenase domain-containing protein 1 homolog	-59.62	0.00	85.19	25.56
LDEC013763-RA	agap005178-pa-like protein	-59.19	0.00	84.58	25.39
LDEC004252-RA	cationic amino acid transporter 2 isoform x2	-140.80	0.00	201.20	60.40
LDEC001075-RA	zinc-type alcohol dehydrogenase-like protein	-239.76	0.00	342.79	103.04
LDEC000178-RA	reverse transcriptase	-48.27	0.01	69.05	20.77
LDEC004693-RA	phospholipid scramblase 1-like isoform x1	-83.56	0.00	119.62	36.06
LDEC011140-RA	phosphoglucomutase-2	-146.37	0.00	209.55	63.18
LDEC010384-RA	mediator of rna polymerase ii transcription subunit 15-like isoform x4	-50.62	0.00	72.48	21.86
LDEC001389-RA	gaba-gated ion channel	-155.46	0.00	222.64	67.18
LDEC003266-RA	proteoglycan 4-like isoform x1	-583.02	0.00	835.01	251.99
LDEC015196-RA	lysosomal aspartic protease	-104.87	0.00	150.34	45.47
LDEC019790-RA	pyrroline-5-carboxylate reductase 3	-49.27	0.01	70.64	21.37
LDEC005932-RA	dna polymerase eta	-80.99	0.00	116.12	35.12
LDEC019651-RA	golgin subfamily a member 4-like	-115.19	0.00	165.20	50.01
LDEC011221-RA	centrosomal protein of 290 kda-like isoform x1	-63.60	0.00	91.24	27.63

LDEC001405-RA	sortilin-related receptor-like	-353.55	0.00	507.25	153.70
LDEC007731-RA	cd63 antigen-like	-105.25	0.00	151.01	45.76
LDEC010122-RA	protein gawky isoform x1	-109.67	0.00	157.56	47.89
LDEC006957-RA	small subunit processome component 20 homolog	-75.15	0.00	107.98	32.83
LDEC007840-RA	cadherin-like protein	-82.91	0.00	119.12	36.22
LDEC000643-RA	probable protein phosphatase 2c	-257.16	0.00	369.55	112.39
LDEC016219-RA	protein fam46c isoform x3	-104.35	0.00	150.06	45.70
LDEC006600-RA	uncharacterized family 31 glucosidase kiaa1161 isoform x2	-146.64	0.00	211.11	64.46
LDEC007533-RA	major facilitator superfamily domain-containing protein 9-like	-148.66	0.00	214.04	65.38
LDEC012321-RA	ran-binding protein 9-like	-65.94	0.00	94.95	29.01
LDEC009920-RA	sodium potassium-transporting atpase subunit beta-1-interacting protein isoform x1	-59.98	0.00	86.39	26.41
LDEC002701-RA	probable atp-dependent rna helicase ddx17-like	-1125.59	0.00	1622.16	496.58
LDEC000572-RA	adp-ribosylation factor-like protein 8b-a	-73.91	0.00	106.60	32.69
LDEC019359-RA	fasciculation and elongation protein zeta-2	-121.48	0.00	175.33	53.85
LDEC005802-RA	protein ltv1 homolog	-171.34	0.00	247.38	76.04
LDEC002998-RA	low quality protein: sialin	-103.73	0.00	149.77	46.05
LDEC018182-RA	neutral and basic amino acid transport protein rbat-like isoform x2	-121.33	0.00	175.33	54.00
LDEC011950-RA	cyclin-g2 isoform x1	-394.89	0.00	570.78	175.89
LDEC003376-RA	protein henna	-107.66	0.00	155.68	48.02
LDEC009271-RA	PREDICTED: uncharacterized protein LOC655336	-113.50	0.00	164.14	50.65
LDEC016863-RA	pleckstrin homology-like domain family b member 1 isoform x2	-89.82	0.00	129.99	40.16
LDEC006522-RA	protein phosphatase 1l	-222.11	0.00	321.49	99.38
LDEC021145-RA	corepressor interacting with rbpj 1-like	-65.50	0.00	94.95	29.45
LDEC014031-RA	poly -binding-splicing factor half pint isoform x4	-124.57	0.00	180.60	56.03
LDEC005223-RA	tyrosine-protein kinase hopscotch	-82.45	0.00	119.58	37.14
LDEC015382-RA	dihydroxyacetone phosphate acyltransferase	-110.69	0.00	160.81	50.13
LDEC004485-RA	retinoblastoma-binding protein 5	-53.01	0.00	77.08	24.07
LDEC015738-RA	calcium uptake protein mitochondrial isoform x3	-84.47	0.00	122.88	38.40
LDEC003007-RA	isoform b	-590.67	0.00	859.43	268.76
LDEC016591-RA	hypothetical protein YQE_11786, partial	-67.69	0.00	98.53	30.83
LDEC024633-RA	nadh dehydrogenase subunit 4	-1337.72	0.00	1947.51	609.79
LDEC007736-RA	ubiquitin-conjugating enzyme e2-24 kda	-179.52	0.00	261.36	81.84
LDEC005576-RA	ras gtpase-activating protein 1	-83.78	0.00	121.99	38.21
LDEC000245-RA	tyrosine phosphatase	-124.78	0.00	181.77	56.99
LDEC004673-RA	major facilitator superfamily domain-containing protein 6-like	-64.76	0.00	94.39	29.63
LDEC014539-RA	zinc finger protein 106-like isoform x1	-64.91	0.00	94.63	29.72
LDEC013643-RA	facilitated trehalose transporter tret1	-146.02	0.00	212.87	66.86
LDEC013725-RA	and pleckstrin domain-containing protein 2-like	-52.38	0.00	76.37	23.99
LDEC007908-RA	cdk5 regulatory subunit-associated protein 3	-116.74	0.00	170.30	53.56
LDEC002774-RA	mediator of rna polymerase ii transcription subunit 15-like	-451.71	0.00	658.97	207.26
LDEC002018-RA	protein lsm14 homolog b isoform x3	-158.19	0.00	230.78	72.59
LDEC011548-RA	vacuolar fusion protein ccz1 homolog	-158.18	0.00	230.78	72.61

LDEC010612-RA	probable rna-binding protein 19-like isoform x2	-53.95	0.00	78.74	24.80
LDEC007842-RA	cadherin-like protein	-127.50	0.00	186.15	58.66
LDEC014406-RA	huntingtin-interacting protein 1	-60.42	0.00	88.23	27.80
LDEC009248-RA	protein arginine n-methyltransferase 5	-81.55	0.00	119.09	37.54
LDEC002165-RA	casp8-associated protein 2-like	-53.22	0.00	77.82	24.60
LDEC013104-RA	serine threonine-protein phosphatase 2a 55 kda regulatory subunit b alpha isoform isoform x1	-150.07	0.00	219.46	69.39
LDEC003406-RA	protein wings apart-like isoform x1	-92.54	0.00	135.58	43.04
LDEC012114-RA	protein toll	-88.44	0.00	129.60	41.16
LDEC015308-RA	gdp-l-fucose synthase	-61.82	0.00	90.60	28.78
LDEC010230-RA	protein sda1 homolog	-116.13	0.00	170.30	54.17
LDEC009226-RA	mucin-22 isoform x1	-73.16	0.00	107.30	34.15
LDEC010688-RA	enhancer of polycomb homolog 1	-81.15	0.00	119.05	37.90
LDEC020645-RA	tho complex subunit 2	-144.65	0.00	212.24	67.59
LDEC000956-RA	agap010793-pa-like protein	-146.41	0.00	215.03	68.62
LDEC014400-RA	cuticular protein 92f	-336.90	0.00	494.80	157.90
LDEC024339-RA	protein ssxt isoform x1	-73.25	0.00	107.59	34.34
LDEC021173-RA	low-density lipoprotein receptor-related protein 6	-52.54	0.00	77.22	24.68
LDEC000083-RA	poly rna polymerase protein cid1	-139.15	0.00	204.91	65.77
LDEC003896-RA	peroxidasin homolog isoform x1	-54.37	0.00	80.09	25.72
LDEC019778-RA	c-1-tetrahydrofolate cytoplasmic	-294.02	0.00	433.22	139.20
LDEC005419-RA	caskin-1 isoform x5	-152.71	0.00	225.01	72.30
LDEC018936-RA	isoform b	-74.76	0.00	110.17	35.41
LDEC000445-RA	longitudinals lacking isoform 4	-853.88	0.00	1258.38	404.50
LDEC009163-RA	neurofibromin isoform x1	-123.92	0.00	183.04	59.12
LDEC005785-RA	muscle-specific protein 300	-1026.06	0.00	1516.06	490.01
LDEC008570-RA	pab-dependent poly -specific ribonuclease subunit pan3	-109.51	0.00	161.84	52.33
LDEC010098-RA	ww domain-binding protein 2	-285.42	0.00	422.10	136.69
LDEC008584-RA	protein zer-1 homolog	-61.47	0.00	90.92	29.45
LDEC020084-RA	feline leukemia virus subgroup c receptor-related protein 2-like	-101.93	0.00	150.91	48.98
LDEC017678-RA	multidrug resistance-associated protein 4	-78.90	0.00	116.82	37.92
LDEC017992-RA	PREDICTED: uncharacterized protein LOC100141567 isoform X3	-325.40	0.00	482.13	156.73
LDEC019753-RA	protein beta isoform-like isoform x1	-82.27	0.00	121.92	39.65
LDEC017204-RA	tumor protein p53-inducible nuclear protein 2-like	-57.95	0.00	85.89	27.94
LDEC013178-RA	bromodomain-containing protein 8	-88.77	0.00	131.62	42.85
LDEC021274-RA	ubiquitin carboxyl-terminal hydrolase 24-like isoform x1	-152.39	0.00	226.32	73.93
LDEC005950-RA	wash complex subunit 7	-72.22	0.00	107.27	35.05
LDEC007273-RA	maguk p55 subfamily member 2	-104.29	0.00	154.94	50.65
LDEC016853-RA	transcriptional regulator atrx homolog isoform x2	-80.61	0.00	119.76	39.15
LDEC012008-RA	zinc finger mym-type protein 1-like	-81.53	0.00	121.18	39.65
LDEC004260-RA	high affinity cationic amino acid transporter 1-like	-77.38	0.00	115.05	37.67
LDEC012508-RA	rb1-inducible coiled-coil protein 1 isoform x2	-61.24	0.00	91.10	29.86
LDEC021430-RA	serine proteinase	-190.49	0.00	283.41	92.92

LDEC002196-RA	ring finger protein nhl-1-like	-57.31	0.00	85.29	27.98
LDEC003658-RA	phosphoribosylformylglycinamidine synthase-like	-272.24	0.00	405.58	133.33
LDEC019652-RA	hypothetical protein D910_08010	-86.83	0.00	129.39	42.56
LDEC006166-RA	bis(5 -nucleosyl)-tetraphosphatase	-67.48	0.00	100.62	33.13
LDEC011419-RA	dnaj homolog subfamily c member 7	-543.26	0.00	810.41	267.15
LDEC015368-RA	5 -amp-activated protein kinase catalytic subunit alpha-2	-76.31	0.00	113.89	37.58
LDEC014261-RA	protein tssc1	-61.54	0.00	91.87	30.33
LDEC014503-RA	calcium uptake protein 1 mitochondrial isoform x2	-123.16	0.00	183.89	60.73
LDEC000123-RA	tyrosine-protein kinase btk29a isoform x3	-53.35	0.01	79.66	26.31
LDEC021714-RA	extended synaptotagmin-2-b isoform x3	-110.04	0.00	164.39	54.35
LDEC015374-RA	ras-related protein rab-1a	-545.48	0.00	814.90	269.43
LDEC011020-RA	lim and sh3 domain protein lasp	-324.04	0.00	484.57	160.52
LDEC008009-RA	map microtubule affinity-regulating kinase 3	-79.94	0.00	119.58	39.65
LDEC022660-RA	selenium-binding protein 1	-107.12	0.00	160.39	53.27
LDEC005491-RA	ribose-phosphate pyrophosphokinase 1 isoform x2	-373.67	0.00	559.77	186.11
LDEC019936-RA	carnitine o-palmitoyltransferase mitochondrial	-108.82	0.00	163.19	54.36
LDEC015101-RA	serine threonine-protein kinase mark2 isoform x1	-248.67	0.00	373.02	124.35
LDEC010542-RA	mlx-interacting protein isoform x3	-77.95	0.00	116.97	39.01
LDEC005040-RA	n-myc protein	-140.68	0.00	211.14	70.46
LDEC020769-RA	glutathione s-transferase theta-1-like isoform x2	-57.96	0.00	86.99	29.03
LDEC003731-RA	leucine-rich repeat-containing protein 16a	-63.97	0.00	96.05	32.08
LDEC000075-RA	interference hedgehog-like isoform x1	-99.98	0.00	150.16	50.19
LDEC000223-RA	protein jumonji	-66.20	0.00	99.48	33.29
LDEC011732-RA	major facilitator superfamily domain-containing protein 1-like	-87.34	0.00	131.26	43.92
LDEC017833-RA	protein tyrosine phosphatase type iva 1	-642.77	0.00	966.06	323.29
LDEC005924-RA	unhealthy ribosome biogenesis protein 2-like protein	-79.65	0.00	119.80	40.15
LDEC002970-RA	serine threonine-protein kinase rio3	-75.10	0.00	112.97	37.87
LDEC007271-RA	ubiquitin-protein ligase e3c-like	-73.11	0.00	109.99	36.89
LDEC009300-RA	yellow-1 precursor	-205.13	0.00	308.82	103.69
LDEC007491-RA	microtubule-associated protein futsch isoform x1	-85.99	0.00	129.74	43.75
LDEC015900-RA	rps6-p70-protein partial	-234.88	0.00	354.51	119.63
LDEC009984-RA	sh3 and multiple ankyrin repeat domains protein 3 isoform x1	-60.95	0.00	92.02	31.06
LDEC020111-RA	rna exonuclease nef-sp	-84.01	0.00	126.84	42.83
LDEC011344-RA	vesicle transport protein sft2b	-105.31	0.00	159.05	53.73
LDEC014179-RA	histone-lysine n-methyltransferase setmar-like	-74.73	0.00	112.86	38.13
LDEC022787-RA	poly -specific ribonuclease parn	-54.36	0.01	82.14	27.79
LDEC018629-RA	ribosomal l1 domain-containing protein cg13096-like	-86.88	0.00	131.30	44.42
LDEC003223-RA	polycomb protein asx isoform x1	-58.71	0.00	88.80	30.09
LDEC000778-RA	monocarboxylate transporter 14 isoform x2	-155.28	0.00	234.92	79.64
LDEC010796-RA	tbc1 domain family member 14	-52.82	0.01	79.91	27.10
LDEC001806-RA	prickle-like protein 2	-61.28	0.00	92.72	31.45
LDEC011351-RA	protein crooked neck	-67.74	0.00	102.63	34.90

LDEC012820-RA	unknown	-117.58	0.00	178.19	60.61
LDEC015184-RA	abhydrolase domain-containing protein 4-like isoform x2	-98.55	0.00	149.38	50.84
LDEC002685-RA	atp-binding cassette sub-family e member 1	-386.89	0.00	586.56	199.67
LDEC011475-RA	microtubule-associated serine threonine-protein kinase 3 isoform x4	-64.19	0.00	97.32	33.13
LDEC008586-RA	probable deoxyhypusine synthase	-111.70	0.00	169.38	57.68
LDEC001860-RA	protein suppressor of sable isoform x2	-98.39	0.00	149.21	50.82
LDEC021826-RA	---NA---	-137.04	0.00	207.85	70.81
LDEC008516-RA	ufm1-specific protease 2	-58.78	0.00	89.15	30.37
LDEC020164-RA	facilitated trehalose transporter tret1	-73.40	0.00	111.34	37.94
LDEC016248-RA	tetraspanins-like protein-8	-353.78	0.00	536.66	182.89
LDEC013559-RA	probable gpi-anchored adhesin-like protein pga55	-102.83	0.00	156.00	53.18
LDEC009638-RA	guanine nucleotide-releasing factor 2 isoform x5	-81.89	0.00	124.29	42.41
LDEC000059-RA	zinc finger ccch domain-containing protein 11a-like isoform x2	-63.88	0.00	96.97	33.09
LDEC012586-RA	e3 ubiquitin-protein ligase huwe1	-158.80	0.00	241.22	82.42
LDEC013926-RA	e3 ubiquitin-protein ligase mdm2-like	-65.21	0.00	99.13	33.92
LDEC005745-RA	ankyrin repeat and mynd domain-containing protein 2	-79.78	0.00	121.28	41.51
LDEC004745-RA	myb-binding protein 1a	-109.92	0.00	167.47	57.55
LDEC018364-RA	chitin synthase 2	-84.48	0.00	128.79	44.30
LDEC004694-RA	kinesin-like protein kif13a	-151.93	0.00	231.74	79.81
LDEC008970-RA	enolase-phosphatase e1-like	-124.79	0.00	190.37	65.57
LDEC004105-RA	chitin synthase	-168.30	0.00	257.01	88.70
LDEC010179-RA	kazal-type proteinase inhibitor-like protein	-86.84	0.00	132.71	45.88
LDEC001939-RA	rna-directed dna polymerase from mobile element jockey-like	-64.54	0.00	98.67	34.13
LDEC008574-RA	large proline-rich protein bag6-like	-187.51	0.00	286.73	99.22
LDEC004144-RA	4-hydroxyphenylpyruvate dioxygenase	-801.64	0.00	1225.86	424.22
LDEC002585-RA	gtp-binding nuclear protein	-70.57	0.00	107.98	37.41
LDEC004177-RA	rho guanine nucleotide exchange factor 10 isoform x1	-58.82	0.00	90.03	31.22
LDEC005499-RA	vacuolar protein sorting-associated protein 13d	-61.75	0.00	94.60	32.84
LDEC013918-RA	maltase 2	-220.23	0.00	337.45	117.22
LDEC016594-RA	ac transposable element-derived protein partial	-124.14	0.00	190.37	66.23
LDEC006673-RA	lian-aa1-like retrotransposon protein	-69.72	0.00	106.95	37.23
LDEC015187-RA	synaptic vesicle membrane protein vat-1 homolog-like	-124.02	0.00	190.40	66.38
LDEC002118-RA	microtubule-associated protein futsch-like isoform x1	-63.30	0.00	97.22	33.92
LDEC004876-RA	cuticular protein analogous to peritrophins 3-d1	-64.40	0.00	98.92	34.51
LDEC016503-RA	mitochondrial import inner membrane translocase subunit tim17-b-like	-129.71	0.00	199.64	69.92
LDEC021712-RA	extended synaptotagmin-like protein 2a	-80.59	0.00	124.22	43.63
LDEC007839-RA	cadherin-like protein	-110.20	0.00	169.87	59.67
LDEC003224-RA	cyclin-y-like protein 1	-114.52	0.00	176.53	62.01
LDEC000817-RA	dentin sialophosphoprotein isoform x3	-55.44	0.01	85.50	30.07
LDEC018764-RA	PREDICTED: uncharacterized protein LOC661670 isoform X2	-174.06	0.00	268.69	94.62
LDEC024050-RA	ras-related protein rab-7a	-95.98	0.00	148.18	52.20
LDEC016710-RA	golgi-specific brefeldin a-resistance guanine nucleotide	-78.74	0.00	121.60	42.87

	exchange factor 1				
LDEC011864-RA	glutamine:fructose-6-phosphate aminotransferase partial	-73.44	0.00	113.60	40.16
LDEC007777-RA	nuclear export mediator factor nemf homolog	-74.63	0.00	115.44	40.82
LDEC010139-RA	cysteine--trna cytoplasmic-like	-93.66	0.00	144.92	51.26
LDEC014703-RA	tbc1 domain family member 9	-58.34	0.00	90.28	31.94
LDEC021491-RA	neurofilament heavy polypeptide-like isoform x2	-86.00	0.00	133.10	47.10
LDEC006839-RA	tudor domain-containing protein 3	-59.62	0.00	92.33	32.71
LDEC012306-RA	15-hydroxyprostaglandin dehydrogenase	-378.90	0.00	586.78	207.88
LDEC014505-RA	protein argonaute-2-like	-132.33	0.00	205.05	72.72
LDEC003226-RA	nuclear receptor coactivator 7 isoform x5	-170.74	0.00	264.86	94.13
LDEC009141-RA	makorin isoform a	-67.23	0.00	104.33	37.10
LDEC016034-RA	acyl- delta desaturase	-103.00	0.00	159.86	56.86
LDEC003886-RA	PREDICTED: uncharacterized protein LOC103313983	-57.85	0.00	89.89	32.04
LDEC007077-RA	tom1-like protein 2 isoform x1	-122.77	0.00	190.90	68.12
LDEC024631-RA	nadh dehydrogenase subunit 5	-1272.32	0.00	1979.65	707.33
LDEC005957-RA	autophagy-related protein 9a isoform x3	-97.71	0.00	152.07	54.36
LDEC001038-RA	e3 ubiquitin-protein ligase siah1-like	-99.15	0.00	154.62	55.48
LDEC010436-RA	docking protein 2	-85.04	0.00	132.64	47.60
LDEC001714-RA	protein roadkill isoform x4	-62.53	0.00	97.54	35.01
LDEC002287-RA	arrestin domain-containing protein 3	-93.20	0.00	145.49	52.29
LDEC005739-RA	zinc finger cchc domain-containing protein 8 homolog	-65.18	0.00	101.78	36.60
LDEC002385-RA	transmembrane protein 131 isoform x1	-88.54	0.00	138.27	49.73
LDEC005083-RA	probable 39s ribosomal protein mitochondrial	-61.92	0.00	96.72	34.80
LDEC012585-RA	e3 ubiquitin-protein ligase huwe1	-165.00	0.00	257.82	92.82
LDEC000144-RA	dynein light	-60.61	0.00	94.74	34.13
LDEC020170-RA	cgmp-dependent protein isozyme 2 forms cd5 t2 isoform x1	-111.44	0.00	174.33	62.89
LDEC002485-RA	histone-lysine n-methyltransferase setmar	-103.62	0.00	162.16	58.54
LDEC015830-RA	poly polymerase type 3	-70.19	0.00	109.85	39.67
LDEC015022-RA	isoform a	-63.00	0.00	98.78	35.78
LDEC017891-RA	isocitrate dehydrogenase	-328.04	0.00	515.22	187.18
LDEC007655-RA	zinc finger protein 271-like	-73.77	0.00	115.87	42.10
LDEC001488-RA	serine threonine-protein kinase	-94.74	0.00	148.82	54.08
LDEC015006-RA	muscle m-line assembly protein unc-89-like	-68.39	0.00	107.48	39.09
LDEC019673-RA	ubiquitin carboxyl-terminal hydrolase 32 isoform x2	-63.05	0.00	99.09	36.04
LDEC002629-RA	rna-binding protein nob1	-102.13	0.00	160.64	58.50
LDEC022838-RA	serine protease	-82.26	0.00	129.42	47.16
LDEC013583-RA	glutathione s-transferase omega-1	-65.34	0.00	102.88	37.54
LDEC009353-RA	tubulin polyglutamylase ttll5	-110.12	0.00	173.91	63.79
LDEC020680-RA	dnaj homolog subfamily c member 2	-173.59	0.00	274.35	100.76
LDEC015551-RA	serine threonine-protein kinase pelle-like isoform x1	-75.21	0.00	119.09	43.88
LDEC002269-RA	calcium calmodulin-dependent protein kinase kinase	-86.69	0.00	137.35	50.67
LDEC010666-RA	mrg morf4l-binding protein	-64.39	0.00	102.39	38.00
LDEC015856-RA	ubiquitin carboxyl-terminal	-90.52	0.00	144.04	53.52

LDEC005266-RA	PREDICTED: uncharacterized protein LOC105199588	-82.64	0.00	131.58	48.94
LDEC007489-RA	cyclic amp-responsive element-binding protein 1 isoform x1	-169.66	0.00	270.42	100.76
LDEC014726-RA	sorting nexin-2	-127.04	0.00	202.51	75.46
LDEC014477-RA	brahma-associated protein of 60 kda isoform x2	-74.55	0.00	118.88	44.32
LDEC010238-RA	casein kinase i isoform alpha isoform x1	-151.05	0.00	241.12	90.06
LDEC002429-RA	membrane-associated progesterone receptor component 1-like	-394.75	0.00	630.70	235.95
LDEC001868-RA	traf-type zinc finger domain-containing protein 1-like isoform x2	-77.71	0.00	124.26	46.55
LDEC001352-RA	xk-related protein 6-like	-104.95	0.00	167.82	62.87
LDEC007638-RA	ecdysone 20-monooxygenase isoform x2	-139.26	0.00	222.71	83.45
LDEC003559-RA	neuropathy target esterase sws isoform x1	-65.61	0.00	104.97	39.36
LDEC016747-RA	hypothetical protein YQE_01873, partial	-71.87	0.00	114.98	43.12
LDEC001731-RA	acidic leucine-rich nuclear phosphoprotein 32 family member a	-164.00	0.00	262.46	98.46
LDEC000658-RA	pecanex-like protein 1 isoform x2	-58.43	0.01	93.54	35.11
LDEC001126-RA	carboxy-terminal domain rna polymerase ii polypeptide a small phosphatase 1	-106.53	0.00	170.69	64.16
LDEC002288-RA	dehydrogenase reductase sdr family member 11-like	-101.15	0.00	162.30	61.15
LDEC014265-RA	zinc finger protein partial	-77.98	0.00	125.14	47.16
LDEC017776-RA	low quality protein: midasin-like	-75.09	0.00	120.54	45.45
LDEC004071-RA	calcium-independent phospholipase a2-gamma	-112.71	0.00	180.99	68.28
LDEC021177-RA	PREDICTED: uncharacterized protein LOC105252462	-88.60	0.00	142.27	53.67
LDEC019072-RA	dnaj homolog subfamily b member 12-like	-85.58	0.00	137.49	51.91
LDEC007667-RA	protein flightless-1	-76.42	0.00	122.95	46.53
LDEC022674-RA	cytochrome p450 9z4	-77.10	0.00	124.04	46.95
LDEC019699-RA	branched-chain-amino-acid cytosolic	-114.69	0.00	184.60	69.91
LDEC006324-RA	beta nu integrin subunit	-60.57	0.00	97.50	36.93
LDEC000767-RA	liprin-alpha-1 isoform x12	-71.95	0.00	115.83	43.88
LDEC016286-RA	coiled-coil and c2 domain-containing protein 1-like isoform x2	-59.48	0.01	95.87	36.39
LDEC013334-RA	symplekin	-85.83	0.00	138.38	52.54
LDEC011693-RA	tubulin polyglutamylase ttll4-like	-80.28	0.00	129.49	49.21
LDEC006924-RA	bromodomain-containing protein 7 isoform x3	-67.51	0.00	108.90	41.39
LDEC003795-RA	fatty acid synthase	-324.87	0.00	524.24	199.37
LDEC002041-RA	mkl myocardin-like protein 2 isoform x3	-82.50	0.00	133.28	50.78
LDEC002962-RA	e3 ubiquitin-protein ligase rbbp6	-67.44	0.00	109.00	41.56
LDEC007677-RA	transport and golgi organization protein 1 isoform x1	-226.93	0.00	366.97	140.04
LDEC018368-RA	btb poz domain-containing protein kctd10	-68.52	0.00	110.81	42.29
LDEC007591-RA	agap005575-pa-like protein	-202.65	0.00	327.86	125.21
LDEC005886-RA	agap010471-pa-like protein	-66.86	0.00	108.37	41.51
LDEC011110-RA	sphingomyelin phosphodiesterase isoform x2	-70.96	0.00	115.05	44.09
LDEC011343-RA	h(+) cl(-) exchange transporter 7	-62.25	0.00	101.08	38.82
LDEC007233-RA	suppressor of presenilin protein 4 isoform x4	-135.54	0.00	220.38	84.83
LDEC020451-RA	thyroid receptor-interacting protein 11	-92.16	0.00	149.88	57.72
LDEC006472-RA	exosome complex component rrp45	-61.29	0.00	99.73	38.44

LDEC005529-RA	leucine-rich repeat-containing protein ddb_g0290503 isoform x4	-162.18	0.00	264.05	101.87
LDEC020216-RA	serine hydroxymethyltransferase	-590.89	0.00	963.12	372.23
LDEC001862-RA	ras-related protein rab-7a	-58.86	0.01	95.98	37.12
LDEC021631-RA	dual 3 -cyclic-amp and -gmp phosphodiesterase 11 isoform x3	-74.54	0.00	121.60	47.06
LDEC010246-RA	class b secretin-like g-protein coupled receptor	-96.48	0.00	157.45	60.98
LDEC009573-RA	ring finger and transmembrane domain-containing protein 2-like	-76.74	0.00	125.28	48.54
LDEC010472-RA	chondroitin sulfate synthase 2	-90.04	0.00	147.22	57.18
LDEC019318-RA	rho guanine nucleotide exchange factor 12 isoform x3	-85.40	0.00	139.72	54.33
LDEC020033-RA	nad-dependent deacetylase sirtuin-2	-67.44	0.00	110.35	42.90
LDEC010363-RA	neurogenic protein mastermind- partial	-109.19	0.00	178.86	69.68
LDEC013135-RA	sphingosine kinase 2	-80.73	0.00	132.25	51.53
LDEC015640-RA	activating signal cointegrator 1 complex subunit 3	-88.87	0.00	145.88	57.01
LDEC012664-RA	60s acidic ribosomal protein p0	-76.98	0.00	126.42	49.44
LDEC015757-RA	modular serine protease zymogen	-70.94	0.00	116.51	45.57
LDEC012660-RA	protein prenyltransferase alpha subunit repeat-containing protein 1-b	-81.88	0.00	134.56	52.68
LDEC020999-RA	PREDICTED: uncharacterized protein LOC662064 isoform X3	-99.70	0.00	163.93	64.23
LDEC000637-RA	mitochondrial nadh-ubiquinone oxidoreductase 9 kda subunit-like protein	-67.01	0.00	110.28	43.27
LDEC003678-RA	hypothetical protein YQE_05934, partial	-126.10	0.00	207.60	81.50
LDEC018653-RA	atp-dependent rna helicase me31b	-301.70	0.00	497.24	195.53
LDEC009543-RA	conserved oligomeric golgi complex subunit 3	-63.15	0.00	104.08	40.93
LDEC007592-RA	---NA---	-2664.89	0.00	4393.18	1728.29
LDEC005630-RA	myotubularin-related protein 3	-109.92	0.00	181.20	71.28
LDEC013772-RA	probable elongation factor 1-delta isoform x3	-505.57	0.00	833.48	327.91
LDEC007866-RA	vinculin isoform x1	-231.94	0.00	382.78	150.85
LDEC013339-RA	isoform a	-340.86	0.00	562.60	221.75
LDEC004393-RA	nuclear factor related to kappa-b-binding protein isoform x1	-213.77	0.00	353.02	139.25
LDEC005098-RA	oxidoreductase glyr1 homolog	-157.76	0.00	260.76	103.00
LDEC011062-RA	PREDICTED: uncharacterized protein LOC659663	-83.46	0.00	138.13	54.67
LDEC003908-RA	cap-gly domain-containing linker protein 1	-88.58	0.00	146.62	58.04
LDEC023469-RA	homogentisate -dioxygenase	-89.52	0.00	148.22	58.69
LDEC010341-RA	eukaryotic peptide chain release factor subunit 1 isoform x1	-147.49	0.00	244.37	96.89
LDEC002222-RA	multidrug resistance-associated protein 4-like isoform x1	-78.79	0.00	130.66	51.87
LDEC018557-RA	protein fam102b-like isoform x7	-79.13	0.00	131.23	52.10
LDEC013040-RA	adenomatous polyposis coli isoform x3	-71.52	0.00	118.66	47.14
LDEC007084-RA	n-alpha-acetyltransferase 60 isoform x2	-103.69	0.00	172.14	68.45
LDEC001807-RA	atp-binding cassette sub-family d member 2	-109.97	0.00	182.58	72.61
LDEC001136-RA	protein slowmo	-221.11	0.00	367.25	146.13
LDEC001662-RA	apoptotic protease-activating factor 1	-61.27	0.01	101.78	40.51
LDEC019031-RA	probable e3 ubiquitin-protein ligase herc4-like isoform x1	-93.22	0.00	154.87	61.65
LDEC018500-RA	selenoprotein s b-like	-129.17	0.00	214.79	85.62
LDEC013327-RA	endonuclease exonuclease phosphatase family domain-containing protein 1-like	-89.77	0.00	149.31	59.54

LDEC007931-RA	exonuclease 3 -5 domain-like-containing protein 1	-193.54	0.00	321.91	128.37
LDEC022507-RA	ccr4-not transcription	-79.07	0.00	131.62	52.54
LDEC012288-RA	death-associated protein kinase related	-74.60	0.00	124.29	49.69
LDEC010425-RA	gut-specific chitinase	-114.46	0.00	190.83	76.36
LDEC005960-RA	cgmp-dependent 3 -cyclic phosphodiesterase-like	-60.15	0.01	100.30	40.15
LDEC008040-RA	PREDICTED: uncharacterized protein LOC103314979	-521.41	0.00	869.69	348.28
LDEC004821-RA	c1 family cathepsin f10	-122.69	0.00	205.37	82.69
LDEC005910-RA	sensitized chromosome inheritance modifier 19	-60.71	0.01	101.64	40.93
LDEC013823-RA	hypothetical protein D910_11256	-73.98	0.00	123.94	49.96
LDEC003174-RA	e3 ubiquitin-protein ligase rnf181	-66.93	0.00	112.33	45.40
LDEC008460-RA	zinc finger protein 271-like	-143.24	0.00	240.41	97.17
LDEC015782-RA	PREDICTED: fibrillin-2	-61.59	0.01	103.55	41.97
LDEC000839-RA	cell division cycle and apoptosis regulator protein 1-like	-125.04	0.00	210.75	85.71
LDEC003739-RA	ubiquinone biosynthesis protein mitochondrial	-100.34	0.00	169.17	68.83
LDEC019000-RA	xaa-pro aminopeptidase 1	-72.31	0.00	121.92	49.61
LDEC004820-RA	rna-binding protein nova-1 isoform x2	-234.10	0.00	395.07	160.97
LDEC005077-RA	cytochrome b5-related	-282.03	0.00	476.39	194.37
LDEC006581-RA	nascent polypeptide-associated complex subunit muscle-specific form-like	-133.03	0.00	224.84	91.81
LDEC007944-RA	probable serine threonine-protein kinase ddb_g0282963 isoform x1	-120.12	0.00	203.32	83.20
LDEC002090-RA	beta-arrestin-1 isoform x3	-103.82	0.00	175.89	72.07
LDEC006078-RA	n-alpha-acetyltransferase auxiliary subunit isoform x1	-70.75	0.00	119.97	49.23
LDEC020030-RA	dna repair protein xp-c	-75.83	0.00	128.72	52.89
LDEC020093-RA	hypothetical protein D910_03631, partial	-67.94	0.00	115.48	47.54
LDEC020095-RA	nuclear pore complex protein nup153	-103.93	0.00	176.67	72.74
LDEC002337-RA	rhomboid-related protein 3-like isoform 2	-94.68	0.00	161.13	66.46
LDEC000922-RA	glycosyl hydrolase	-138.02	0.00	235.42	97.40
LDEC009346-RA	wd sam and u-box domain-containing protein 1-like	-77.59	0.00	132.36	54.77
LDEC002750-RA	sodium potassium-transporting atpase subunit beta-1	-105.09	0.00	179.29	74.20
LDEC008710-RA	serine protease p146	-116.37	0.00	198.61	82.25
LDEC018338-RA	myosin 1a isoform x1	-112.49	0.00	192.38	79.89
LDEC014221-RA	ataxin-1	-72.07	0.00	123.37	51.30
LDEC010108-RA	ribosome biogenesis regulatory protein homolog	-109.69	0.00	187.89	78.20
LDEC002382-RA	nucleosome assembly protein 1-like 1	-886.79	0.00	1519.04	632.25
LDEC014553-RA	mitogen-activated protein kinase 1	-160.77	0.00	275.76	114.99
LDEC011702-RA	ubiquitin carboxyl-terminal hydrolase 45	-121.06	0.00	207.81	86.75
LDEC017046-RA	v-type proton atpase 116 kda subunit a isoform 1	-135.62	0.00	233.05	97.42
LDEC015124-RA	ist1 homolog isoform x5	-126.81	0.00	218.50	91.69
LDEC007630-RA	protein trc8 homolog isoform x1	-93.62	0.00	161.42	67.80
LDEC004521-RA	de cadherin-like protein	-104.51	0.00	180.81	76.31
LDEC012518-RA	retinol dehydrogenase 11	-87.25	0.00	151.12	63.87
LDEC018589-RA	leucine-rich repeat-containing protein ddb_g0290503 isoform x1	-211.25	0.00	366.22	154.97
LDEC001071-RA	n-alpha-acetyltransferase auxiliary subunit	-74.12	0.00	128.54	54.42

LDEC018745-RA	major facilitator superfamily domain-containing protein 6 isoform x2	-183.81	0.00	319.54	135.73
LDEC003012-RA	trifunctional purine biosynthetic protein adenosine-3	-170.62	0.00	296.79	126.17
LDEC005417-RA	PREDICTED: uncharacterized protein LOC659233	-71.38	0.00	124.29	52.91
LDEC009045-RA	protein mo25	-129.72	0.00	225.90	96.18
LDEC007215-RA	insulin-like growth factor-binding protein complex acid labile subunit	-96.38	0.00	168.35	71.97
LDEC010351-RA	tropomodulin isoform x1	-305.96	0.00	534.68	228.72
LDEC011549-RA	hypothetical protein TcasGA2_TC003147	-224.27	0.00	392.27	168.00
LDEC002294-RA	isoform a	-95.45	0.00	167.11	71.67
LDEC018424-RA	ribonuclease x25	-376.34	0.00	658.97	282.63
LDEC012338-RA	cytochrome p450-like protein	-124.31	0.00	217.90	93.59
LDEC011355-RA	tetratricopeptide repeat protein 17-like isoform x1	-75.20	0.00	131.83	56.63
LDEC019611-RA	cap-gly domain-containing linker protein 1 isoform x4	-134.87	0.00	236.55	101.68
LDEC005425-RA	glycoside hydrolase family 28 protein	-67.72	0.01	118.84	51.13
LDEC015595-RA	serine threonine protein kinase	-100.13	0.00	175.79	75.65
LDEC000157-RA	---NA---	-74.76	0.00	131.37	56.61
LDEC014493-RA	rho gtpase-activating protein 5	-86.29	0.00	151.79	65.50
LDEC000801-RA	juvenile hormone-inducible protein	-79.75	0.00	140.36	60.61
LDEC002088-RA	oxidoreductase glyr1 homolog	-90.55	0.00	159.79	69.23
LDEC016561-RA	autophagy-related protein 101	-71.68	0.00	126.49	54.80
LDEC021067-RA	protein tprxl isoform x2	-72.16	0.00	127.65	55.49
LDEC014615-RA	microsomal glutathione s-transferase 1	-183.41	0.00	324.60	141.19
LDEC016007-RA	poly -specific endoribonuclease homolog	-96.52	0.00	171.08	74.56
LDEC002400-RA	cubilin	-171.88	0.00	304.68	132.80
LDEC007044-RA	molybdenum cofactor biosynthesis protein 1	-71.22	0.00	126.27	55.05
LDEC005504-RA	PREDICTED: sideroflexin-3	-107.63	0.00	190.93	83.30
LDEC001871-RA	mitogen-activated protein kinase-binding protein 1 isoform x2	-74.57	0.00	133.53	58.96
LDEC019483-RA	gamma-interferon-inducible lysosomal thiol reductase-like	-544.15	0.00	975.08	430.93
LDEC000858-RA	fatty acid synthase	-453.44	0.00	813.95	360.51
LDEC010954-RA	intracellular protein transport protein uso1-like isoform x1	-114.18	0.00	205.05	90.87
LDEC009421-RA	peritrophic matrix protein 2-b precursor	-79.18	0.00	142.41	63.24
LDEC022378-RA	eukaryotic translation initiation factor 3 subunit m	-174.05	0.00	313.60	139.54
LDEC008777-RA	serine threonine-protein phosphatase 4 regulatory subunit 1-like isoform x10	-130.94	0.00	236.16	105.22
LDEC019265-RA	adenosine kinase	-68.04	0.01	122.77	54.73
LDEC019900-RA	probable serine hydrolase	-70.75	0.00	127.80	57.05
LDEC014909-RA	tppp family protein cg45057	-121.16	0.00	219.17	98.02
LDEC014018-RA	f-box only protein 28	-142.27	0.00	257.40	115.13
LDEC020333-RA	prostaglandin f synthase-like	-97.15	0.00	175.89	78.74
LDEC003608-RA	synaptotagmin-7 isoform x4	-72.47	0.00	131.26	58.79
LDEC009333-RA	amp deaminase 2 isoform x6	-118.91	0.00	215.56	96.66
LDEC003369-RA	mog interacting and ectopic p-granules protein 1 isoform x2	-127.90	0.00	232.16	104.26
LDEC001941-RA	hypothetical protein TcasGA2_TC015166	-123.09	0.00	223.63	100.55
LDEC006511-RA	d-3-phosphoglycerate dehydrogenase	-257.50	0.00	469.03	211.54

LDEC017723-RA	tgf-beta receptor type-1 isoform x2	-83.83	0.00	152.78	68.95
LDEC020757-RA	cytochrome p450	-69.65	0.01	127.65	58.01
LDEC003356-RA	mitochondrial carrier homolog 2-like isoform x1	-72.52	0.00	132.96	60.44
LDEC016551-RA	bone morphogenetic protein receptor type-1b isoform x4	-86.89	0.00	159.36	72.47
LDEC005952-RA	rna-binding protein 39	-167.44	0.00	307.44	140.00
LDEC018195-RA	atp-dependent rna helicase belle	-149.84	0.00	275.59	125.74
LDEC014999-RA	protein kibra-like isoform x3	-74.97	0.00	138.38	63.41
LDEC007937-RA	protein strawberry notch	-84.17	0.00	155.47	71.30
LDEC004321-RA	low quality protein: plexin-b-like	-82.74	0.00	152.85	70.12
LDEC012029-RA	probable cytochrome p450 mitochondrial	-74.23	0.00	137.14	62.91
LDEC016789-RA	myeloid leukemia factor isoform x4	-166.39	0.00	307.58	141.19
LDEC005965-RA	tyrosine aminotransferase	-421.92	0.00	783.94	362.02
LDEC006435-RA	rna-binding protein fusilli isoform x2	-98.27	0.00	183.50	85.24
LDEC021987-RA	folate carrier protein	-159.93	0.00	298.84	138.91
LDEC002321-RA	low-density lipoprotein	-114.85	0.00	215.07	100.22
LDEC012729-RA	ets dna-binding protein pokkuri	-131.28	0.00	246.85	115.57
LDEC002916-RA	prominin-like protein isoform x1	-97.50	0.00	183.75	86.25
LDEC018190-RA	u5 small nuclear ribonucleoprotein 200 kda helicase	-153.30	0.00	289.07	135.77
LDEC016847-RA	neuroplastin isoform x1	-493.40	0.00	932.22	438.82
LDEC009435-RA	aldose reductase	-78.78	0.00	148.85	70.08
LDEC014766-RA	facilitated trehalose transporter tret1	-119.35	0.00	225.72	106.37
LDEC022506-RA	ccr4-not transcription complex subunit 1 isoform 1	-216.23	0.00	409.01	192.78
LDEC012185-RA	renin receptor	-137.71	0.00	261.54	123.83
LDEC005023-RA	ww domain-binding protein 11	-78.75	0.00	149.60	70.84
LDEC014857-RA	ww domain-containing adapter protein with coiled-coil-like isoform x3	-97.59	0.00	185.84	88.24
LDEC007747-RA	ctl-like protein 1	-206.70	0.00	393.86	187.16
LDEC004665-RA	udp-glucuronosyltransferase 2b7	-80.54	0.00	153.49	72.95
LDEC004797-RA	cystathionine beta-synthase isoform x2	-170.05	0.00	324.46	154.41
LDEC000855-RA	fatty acid-binding protein	-115.23	0.00	220.59	105.36
LDEC014777-RA	protein angel isoform x2	-171.33	0.00	328.39	157.06
LDEC015682-RA	acylamino-acid-releasing enzyme	-78.34	0.00	150.16	71.82
LDEC001388-RA	poly	-77.75	0.00	149.95	72.20
LDEC001072-RA	eh domain-containing protein 3	-162.68	0.00	314.48	151.81
LDEC014153-RA	serine threonine-protein kinase n isoform x4	-89.34	0.00	172.99	83.64
LDEC009642-RA	programmed cell death protein 4	-158.02	0.00	306.20	148.18
LDEC007303-RA	protein preli-like	-106.58	0.00	206.82	100.24
LDEC005597-RA	purine biosynthesis protein pur6	-103.17	0.00	200.20	97.04
LDEC017506-RA	importin-5	-476.49	0.00	927.31	450.82
LDEC005244-RA	serine arginine repetitive matrix protein 1 isoform x1	-89.48	0.00	174.16	84.68
LDEC011015-RA	maternal protein exuperantia-like	-224.35	0.00	437.04	212.69
LDEC008160-RA	lysosomal aspartic protease	-1002.26	0.00	1954.23	951.98
LDEC015758-RA	acidic mammalian chitinase-like	-130.12	0.00	253.86	123.73

LDEC015589-RA	sh3 domain-binding protein 5 homolog	-96.14	0.00	187.82	91.67
LDEC012212-RA	muscleblind-like protein 2	-169.95	0.00	332.32	162.36
LDEC006054-RA	protein lap4-like isoform 1	-108.40	0.00	212.70	104.30
LDEC011073-RA	low quality protein: trithorax group protein osa	-87.99	0.00	172.71	84.72
LDEC002457-RA	26s proteasome non-atpase regulatory subunit 12	-134.60	0.00	265.00	130.40
LDEC001742-RA	nicotinate phosphoribosyltransferase isoform x2	-80.87	0.00	159.40	78.53
LDEC007663-RA	helicase domino	-220.98	0.00	435.69	214.72
LDEC003414-RA	ankyrin repeat domain-containing protein 17	-211.44	0.00	417.64	206.21
LDEC015029-RA	cdc42 homolog	-161.38	0.00	319.19	157.80
LDEC013900-RA	dynein regulatory complex protein 1	-106.80	0.00	211.57	104.76
LDEC016796-RA	esterase b1-like	-77.57	0.01	153.70	76.13
LDEC007487-RA	u-box domain containing 5	-175.29	0.00	348.17	172.88
LDEC020453-RA	ryanodine receptor	-138.91	0.00	276.15	137.24
LDEC022003-RA	lysosomal aspartic protease-like	-447.67	0.00	892.41	444.74
LDEC013712-RA	ubiquitin carboxyl-terminal hydrolase 2 isoform x2	-122.18	0.00	243.84	121.66
LDEC010895-RA	sodium hydrogen exchanger 8	-112.23	0.00	224.52	112.29
LDEC024629-RA	cytochrome c oxidase subunit iii	-3764.68	0.00	7551.51	3786.83
LDEC014283-RA	zinc finger protein	-98.48	0.00	197.76	99.28
LDEC020629-RA	PREDICTED: prosaposin	-339.73	0.00	682.26	342.53
LDEC000532-RA	vesicular integral-membrane protein vip36	-124.41	0.00	249.96	125.55
LDEC016282-RA	charged multivesicular body protein 1b	-119.82	0.00	241.05	121.22
LDEC009330-RA	purine nucleoside phosphorylase-like isoform x2	-212.04	0.00	426.95	214.91
LDEC011294-RA	tripeptidyl-peptidase 2 isoform x2	-99.99	0.00	201.58	101.60
LDEC011341-RA	isoform p	-455.98	0.00	922.70	466.72
LDEC006827-RA	lim and calponin homology domains-containing protein 1 isoform x2	-100.44	0.00	203.67	103.23
LDEC002389-RA	cwf19-like protein 2 homolog	-99.70	0.00	202.26	102.56
LDEC005983-RA	filamin-c isoform x1	-111.57	0.00	226.36	114.78
LDEC004461-RA	luciferase homolog	-248.94	0.00	505.20	256.26
LDEC004605-RA	mariner transposase	-117.48	0.00	238.60	121.13
LDEC009411-RA	serrate rna effector molecule homolog isoform x2	-153.71	0.00	312.78	159.07
LDEC010083-RA	protein split ends	-190.82	0.00	388.94	198.12
LDEC007679-RA	xanthine dehydrogenase isoform x1	-270.96	0.00	553.05	282.09
LDEC017911-RA	flavin-containing monooxygenase fmo gs-ox-like 3-like	-85.81	0.00	175.82	90.01
LDEC011512-RA	atp-binding cassette sub-family a member 3-like	-118.28	0.00	242.53	124.25
LDEC001412-RA	leucine--trna cytoplasmic	-215.65	0.00	442.60	226.94
LDEC007757-RA	pyridoxal kinase	-101.19	0.00	208.27	107.08
LDEC004927-RA	atpase family aaa domain-containing protein 1-a-like	-87.86	0.00	180.95	93.09
LDEC019372-RA	protein ssxt isoform x1	-103.79	0.00	213.97	110.18
LDEC010050-RA	proton-coupled amino acid transporter 4 isoform x3	-251.45	0.00	519.78	268.33
LDEC001811-RA	transcriptional activator protein pur-alpha isoform x1	-143.95	0.00	297.71	153.76
LDEC001809-RA	cysteine string protein	-84.97	0.00	176.03	91.06
LDEC011215-RA	protein spinster isoform x2	-93.77	0.00	195.71	101.94

LDEC005395-RA	atp synthase subunit mitochondrial	-121.63	0.00	254.39	132.76
LDEC006607-RA	myosin heavy chain 95f isoform x1	-375.92	0.00	787.05	411.13
LDEC001865-RA	protein-methionine sulfoxide oxidase mical3 isoform x8	-187.01	0.00	392.13	205.12
LDEC007239-RA	dnaj homolog subfamily a member 2	-224.18	0.00	470.80	246.62
LDEC003353-RA	PREDICTED: uncharacterized protein LOC103314894	-94.27	0.00	197.98	103.71
LDEC005971-RA	atp-binding cassette sub-family f member 2	-124.75	0.00	262.07	137.32
LDEC011559-RA	n-acetylneuraminate lyase-like	-195.91	0.00	412.37	216.46
LDEC011511-RA	atp-binding cassette sub-family a member 3-like isoform x4	-101.18	0.00	213.05	111.87
LDEC016955-RA	tether containing ubx domain for glut4-like	-95.02	0.00	200.20	105.18
LDEC003125-RA	tubulin-specific chaperone a	-130.49	0.00	275.06	144.56
LDEC007734-RA	cd63 antigen	-118.44	0.00	250.99	132.55
LDEC000050-RA	mitochondrial import receptor subunit tom70-like	-95.47	0.00	202.47	107.00
LDEC003767-RA	isoform b	-91.04	0.00	193.41	102.37
LDEC024632-RA	nadh dehydrogenase subunit 4	-298.08	0.00	635.19	337.11
LDEC017398-RA	dentin sialophospho	-113.59	0.00	242.07	128.49
LDEC011496-RA	leucine-rich repeat-containing protein ddb_g0290503	-118.09	0.00	254.56	136.48
LDEC007282-RA	juvenile hormone acid methyltransferase	-91.39	0.00	198.90	107.50
LDEC007863-RA	oxysterol-binding protein 1	-98.76	0.00	215.21	116.45
LDEC006826-RA	lim and calponin homology domains-containing protein 1 isoform x2	-99.43	0.00	219.78	120.34
LDEC012575-RA	semaphorin-1a-like isoform x1	-104.30	0.00	230.92	126.63
LDEC001481-RA	next to brca1 gene 1 protein	-140.40	0.00	312.32	171.93
LDEC024057-RA	eukaryotic translation initiation factor 3 subunit e-like	-113.58	0.00	252.72	139.14
LDEC004640-RA	protein cepu-1	-147.95	0.00	330.48	182.52
LDEC004244-RA	translational activator gcn1	-160.88	0.00	359.92	199.04
LDEC003442-RA	hypothetical protein YQE_03563, partial	-310.03	0.00	695.39	385.36
LDEC013287-RA	cytochrome p450 9z4	-333.65	0.00	750.46	416.81
LDEC022187-RA	cathepsin b	-101.88	0.00	231.38	129.50
LDEC020608-RA	heterogeneous nuclear ribonucleoprotein q-like isoform x1	-149.37	0.00	340.25	190.88
LDEC016249-RA	23 kda integral membrane	-143.25	0.00	326.48	183.23
LDEC001692-RA	fatty acid synthase-like isoform x3	-452.23	0.00	1033.55	581.32
LDEC019788-RA	cysteine proteinase	-402.02	0.00	920.90	518.88
LDEC003276-RA	28 kda heat- and acid-stable phosphoprotein	-96.52	0.01	223.70	127.18
LDEC019896-RA	dnaj homolog subfamily b member 6 isoform x2	-94.16	0.01	218.89	124.73
LDEC003053-RA	translationally-controlled tumor protein homolog	-493.36	0.00	1152.28	658.92
LDEC012940-RA	PREDICTED: LOW QUALITY PROTEIN: uncharacterized protein LOC658528	-259.45	0.00	608.44	348.99
LDEC010782-RA	dimethyladenosine transferase mitochondrial	-100.94	0.01	242.04	141.09
LDEC016402-RA	glycogenin-2-like isoform x3	-120.07	0.00	289.42	169.36
LDEC020891-RA	1-acyl-sn-glycerol-3-phosphate acyltransferase gamma-like isoform x1	-108.98	0.00	263.48	154.51
LDEC014667-RA	microtubule-associated protein futsch isoform x1	-130.08	0.00	315.58	185.49
LDEC017469-RA	cullin-associated nedd8-dissociated protein 1	-122.84	0.00	299.90	177.06
LDEC001822-RA	serine threonine-protein phosphatase alpha-2 isoform	-107.76	0.00	265.61	157.84
LDEC011832-RA	protein jim lovell isoform x4	-109.70	0.00	270.63	160.93

LDEC014164-RA	importin subunit alpha	-107.62	0.00	265.54	157.92
LDEC016119-RA	protein tis11	-145.50	0.00	366.29	220.79
LDEC010566-RA	serine threonine-protein phosphatase 2a catalytic subunit alpha isoform	-176.44	0.00	446.77	270.33
LDEC016510-RA	fatty acid synthase	-219.52	0.00	558.22	338.70
LDEC017562-RA	venom carboxylesterase-6-like	-153.03	0.00	389.69	236.66
LDEC018739-RA	chloride intracellular channel exl-	-111.96	0.00	285.99	174.03
LDEC012360-RA	titin isoform x2	-185.68	0.00	482.87	297.19
LDEC003986-RA	nadph--cytochrome p450 reductase isoform x2	-440.72	0.00	1156.42	715.70
LDEC002968-RA	niemann-pick c1 isoform x3	-113.85	0.01	300.36	186.51
LDEC013421-RA	eukaryotic translation initiation factor 3 subunit i	-119.49	0.00	317.17	197.68
LDEC008099-RA	four and a half lim domains	-311.03	0.00	836.81	525.78
LDEC011313-RA	facilitated trehalose transporter tret1-like	-840.26	0.00	2278.41	1438.15
LDEC013069-RA	h+ transporting atp synthase o subunit isoform 1	-204.08	0.00	554.89	350.81
LDEC019777-RA	glycosyl hydrolase	-151.56	0.00	420.16	268.60
LDEC000985-RA	glycogen synthase kinase-3 beta isoform x3	-121.98	0.01	338.90	216.92
LDEC006133-RA	glycerol-3-phosphate dehydrogenase	-219.65	0.00	636.78	417.13
LDEC018238-RA	agap004396-pa-like protein	-265.21	0.00	773.07	507.87
LDEC018262-RA	synaptic vesicle glycoprotein 2b	-613.30	0.00	1791.47	1178.17
LDEC009803-RA	muscle m-line assembly protein unc- partial	-264.98	0.00	789.42	524.44
LDEC017156-RA	apolipophorin- partial	-499.31	0.00	1545.90	1046.58
LDEC003530-RA	filamin-a isoform x1	-146.12	0.00	465.39	319.27
LDEC006258-RA	spectrin alpha chain	-214.78	0.00	720.73	505.95
LDEC022004-RA	lysosomal aspartic protease	-585.74	0.00	2025.87	1440.12
LDEC021171-RA	ribosomal protein l5	-592.42	0.00	2061.54	1469.12
LDEC009012-RA	microtubule-actin cross-linking factor 1 isoform x9	-179.37	0.00	625.92	446.55
LDEC012939-RA	vigilin	-214.26	0.00	827.26	612.99
LDEC021542-RA	chitin deacetylase 1	-386.80	0.00	2278.34	1891.54
LDEC017569-RA	adenine nucleotide translocase	-1103.46	0.00	6808.02	5704.56
LDEC004552-RA	translation elongation factor 2	-938.83	0.00	6757.63	5818.79
LDEC005654-RA	chitin-binding protein	-399.88	0.00	3157.37	2757.49
LDEC012644-RA	s-adenosylmethionine synthetase	406.69	0.00	1708.34	2115.03
LDEC000809-RA	peptidyl-prolyl cis-trans isomerase	350.13	0.00	1330.37	1680.50
LDEC011577-RA	ribosomal protein l7ae	250.31	0.00	873.02	1123.33
LDEC018225-RA	myrosinase 1-like	204.29	0.01	696.24	900.53
LDEC005085-RA	40s ribosomal protein s10	248.85	0.00	844.10	1092.96
LDEC014063-RA	ferritin 2	209.80	0.00	676.35	886.15
LDEC003616-RA	apolipoprotein li-ii precursor	263.80	0.00	818.94	1082.74
LDEC001326-RA	ornithine decarboxylase antizyme partial	233.76	0.00	705.55	939.31
LDEC003545-RA	isoform c	197.95	0.00	575.03	772.98
LDEC004253-RA	ribosomal protein l27e	218.62	0.00	625.28	843.90
LDEC024338-RA	long form-like	194.88	0.00	556.94	751.82
LDEC007644-RA	60s ribosomal protein l18a	257.52	0.00	704.98	962.50

LDEC001536-RA	elongation factor 1-alpha isoform x1	2689.10	0.00	7334.25	10023.35
LDEC013855-RA	60s ribosomal protein l18	278.63	0.00	716.94	995.57
LDEC009934-RA	40s ribosomal protein s3a	460.80	0.00	1178.86	1639.66
LDEC001798-RA	ribosomal protein l19e	323.23	0.00	803.51	1126.74
LDEC005287-RA	ferritin 2	239.87	0.00	583.17	823.03
LDEC010962-RA	fructose -bisphosphate aldolase	235.30	0.00	550.29	785.59
LDEC003536-RA	polyadenylate-binding protein 1-like isoform 1	1042.56	0.00	2430.77	3473.33
LDEC018144-RA	myosin heavy chain	3258.86	0.00	7579.47	10838.33
LDEC015684-RA	ribosomal protein l10	196.19	0.00	454.91	651.11
LDEC000772-RA	sparc	191.28	0.00	437.43	628.70
LDEC018973-RA	ribosomal protein s26	174.34	0.00	390.50	564.84
LDEC017095-RA	atp synthase subunit mitochondrial	144.26	0.01	316.68	460.94
LDEC024126-RA	60s ribosomal protein l7a-like	172.15	0.00	377.58	549.74
LDEC017981-RA	atp-dependent rna helicase p62	173.99	0.00	380.59	554.58
LDEC005914-RA	ribosomal protein l7e	392.92	0.00	858.26	1251.18
LDEC005034-RA	alpha- partial	221.53	0.00	479.01	700.55
LDEC010190-RA	60s ribosomal protein l13a	329.44	0.00	688.60	1018.03
LDEC005653-RA	mucin- partial	154.02	0.00	319.01	473.03
LDEC021000-RA	peroxisomal acyl-coenzyme a oxidase 3-like	203.45	0.00	417.40	620.85
LDEC016361-RA	aspartate mitochondrial	212.98	0.00	435.76	648.75
LDEC003355-RA	agap006602-pa-like protein	146.80	0.00	299.90	446.70
LDEC010949-RA	isoform b	204.10	0.00	406.78	610.88
LDEC015117-RA	chromobox protein homolog 5	156.92	0.00	271.94	428.86
LDEC014944-RA	adp-ribosylation factor 1	115.74	0.01	199.67	315.42
LDEC005064-RA	juvenile hormone epoxide hydrolase-like protein 3	120.46	0.00	206.93	327.39
LDEC009709-RA	long form	121.78	0.00	206.19	327.97
LDEC000701-RA	cytochrome p450	2783.04	0.00	4665.55	7448.59
LDEC000562-RA	translocator protein	174.32	0.00	288.68	463.01
LDEC005045-RA	activating transcription factor of chaperone-like	721.74	0.00	1187.21	1908.96
LDEC018073-RA	ribosomal protein l17	400.09	0.00	653.20	1053.29
LDEC000058-RA	glycogen phosphorylase	177.54	0.00	287.90	465.44
LDEC011542-RA	ribosomal protein l14	348.67	0.00	565.19	913.86
LDEC002424-RA	40s ribosomal protein s19a-like	303.88	0.00	489.70	793.58
LDEC017373-RA	serine arginine repetitive matrix protein 1	140.33	0.00	226.00	366.33
LDEC023007-RA	60s ribosomal protein l6	464.86	0.00	748.19	1213.05
LDEC005976-RA	imaginal disc growth factor 2 precursor	160.18	0.00	252.09	412.26
LDEC021641-RA	elongation factor 1 beta	236.31	0.00	371.57	607.88
LDEC002683-RA	annexin isoform c	310.23	0.00	487.75	797.99
LDEC013717-RA	hydroxyacid oxidase 1	142.67	0.00	223.31	365.99
LDEC003531-RA	filamin-a isoform x3	193.79	0.00	301.32	495.10
LDEC008701-RA	protein l 37cc	120.14	0.00	185.94	306.08
LDEC008691-RA	pyruvate kinase	221.37	0.00	342.58	563.95
LDEC010207-RA	thioredoxin reductase mitochondrial isoform x5	265.95	0.00	411.56	677.51

LDEC014178-RA	atp synthase lipid-binding mitochondrial	1087.89	0.00	1683.00	2770.89
LDEC001515-RA	phosphoglyceromutase	106.73	0.01	164.96	271.69
LDEC008084-RA	40s ribosomal protein s16	388.93	0.00	599.73	988.65
LDEC015377-RA	40s ribosomal protein s25	196.72	0.00	301.99	498.71
LDEC015091-RA	ribosomal protein l26e	233.96	0.00	355.46	589.42
LDEC016969-RA	translocation associated membrane protein	186.77	0.00	278.84	465.61
LDEC014486-RA	2-oxoglutarate mitochondrial-like isoform x1	118.10	0.00	173.34	291.44
LDEC006314-RA	60s ribosomal protein l30	254.38	0.00	368.98	623.36
LDEC017598-RA	eukaryotic translation initiation factor 5a	112.87	0.00	163.43	276.31
LDEC000760-RA	aldose reductase	470.34	0.00	672.88	1143.22
LDEC003113-RA	inorganic phosphate cotransporter	128.24	0.00	181.87	310.11
LDEC000762-RA	aldose reductase	378.45	0.00	531.60	910.05
LDEC004943-RA	fatbody protein 3rev-g1	124.56	0.00	174.72	299.28
LDEC005749-RA	dihydrolipoamide dehydrogenase e3 subunit	170.69	0.00	238.78	409.47
LDEC015280-RA	troponin c ia	177.86	0.00	247.66	425.52
LDEC018924-RA	v-type proton atpase subunit c	99.60	0.01	138.59	238.19
LDEC002964-RA	dihydrolipoamide acetyltransferase component of pyruvate dehydrogenase	191.65	0.00	266.56	458.22
LDEC010111-RA	rna-binding protein lark isoform x4	136.34	0.00	189.48	325.82
LDEC002667-RA	unc93-like protein	110.00	0.00	150.98	260.98
LDEC012375-RA	heat shock 70 kda protein cognate 5	177.22	0.00	242.74	419.97
LDEC017303-RA	---NA---	184.06	0.00	251.20	435.26
LDEC003866-RA	interferon-related developmental regulator 1	135.66	0.00	184.99	320.65
LDEC010164-RA	hairy cell leukemia	161.02	0.00	219.32	380.34
LDEC008164-RA	cytoplasmic a3	1899.37	0.00	2573.25	4472.62
LDEC010126-RA	40s ribosomal protein sa	1266.88	0.00	1705.86	2972.75
LDEC019737-RA	glutamate mitochondrial-like	105.25	0.00	140.61	245.86
LDEC008057-RA	ribosomal protein s6	655.00	0.00	871.99	1526.99
LDEC004599-RA	transketolase-like protein 2 isoform x1	231.19	0.00	306.59	537.78
LDEC021581-RA	cyc_sarpe ame: full=cytochrome c	201.93	0.00	265.25	467.18
LDEC009322-RA	60s ribosomal protein l3	701.26	0.00	916.65	1617.92
LDEC012533-RA	malate mitochondrial	295.18	0.00	385.44	680.62
LDEC015841-RA	probable methylmalonate-semialdehyde dehydrogenase	110.26	0.00	142.52	252.77
LDEC006842-RA	high mobility group protein	221.20	0.00	285.92	507.12
LDEC006099-RA	v-type proton atpase catalytic subunit a	519.86	0.00	667.43	1187.29
LDEC006988-RA	rna-binding protein squid isoform x3	275.86	0.00	352.88	628.74
LDEC009203-RA	atpase inhibitor mai- mitochondrial	92.68	0.01	118.42	211.09
LDEC005337-RA	60s acidic ribosomal protein p0	1209.94	0.00	1542.57	2752.51
LDEC007070-RA	aldehyde mitochondrial	371.87	0.00	470.94	842.81
LDEC019738-RA	glutamate mitochondrial	180.48	0.00	228.23	408.72
LDEC005894-RA	40s ribosomal protein s5	574.85	0.00	722.04	1296.88
LDEC003855-RA	nucleolar protein 56	101.41	0.00	127.30	228.71
LDEC018896-RA	myelin expression factor 2	180.65	0.00	226.08	406.73

LDEC015135-RA	retinaldehyde-binding protein 1	109.19	0.00	136.40	245.59
LDEC010880-RA	cathepsin b	263.16	0.00	321.74	584.90
LDEC014011-RA	cg9135-pa	108.22	0.00	131.87	240.09
LDEC008349-RA	gtp-binding nuclear protein ran	127.67	0.00	155.44	283.11
LDEC014462-RA	probable salivary secreted peptide	241.80	0.00	293.85	535.65
LDEC018920-RA	transitional endoplasmic reticulum atpase ter94	281.46	0.00	338.58	620.04
LDEC002143-RA	40s ribosomal protein s24	347.41	0.00	417.75	765.16
LDEC007868-RA	lamin dm0-like isoform x1	123.82	0.00	148.29	272.11
LDEC021922-RA	low quality protein: sarcalumenin-like	87.36	0.01	103.73	191.09
LDEC009502-RA	midline fasciclin	153.68	0.00	181.41	335.10
LDEC009874-RA	utp-glucose-1-phosphate uridylyltransferase 2	117.82	0.00	137.63	255.46
LDEC014489-RA	isovaleryl coenzyme a dehydrogenase	109.79	0.00	127.62	237.41
LDEC003148-RA	glucosidase 2 subunit beta	158.30	0.00	183.50	341.80
LDEC018192-RA	ribosomal protein l8e	219.85	0.00	253.04	472.89
LDEC016690-RA	surfeit locus protein 4 homolog	129.55	0.00	148.50	278.05
LDEC018261-RA	plasminogen activator inhibitor 1 rna-binding partial	104.58	0.00	119.66	224.24
LDEC007894-RA	atp-citrate synthase	89.65	0.00	102.49	192.14
LDEC013113-RA	ribosomal protein l37e	160.57	0.00	182.62	343.18
LDEC004009-RA	o-linked n-acetylglucosamine ogt	119.53	0.00	135.79	255.32
LDEC000125-RA	hydroxysteroid dehydrogenase-like protein 2	97.78	0.00	110.63	208.41
LDEC000771-RA	uracil-dna degrading isoform b	102.53	0.00	114.95	217.48
LDEC022001-RA	---NA---	588.81	0.00	657.24	1246.05
LDEC010650-RA	phosphoglucose isomerase	182.46	0.00	201.73	384.19
LDEC006115-RA	zinc finger rna-binding protein	153.18	0.00	169.31	322.49
LDEC006658-RA	nadh-cytochrome b5 reductase 2 isoform x1	85.19	0.01	93.96	179.15
LDEC005884-RA	probable aconitate mitochondrial	423.66	0.00	465.85	889.51
LDEC006160-RA	far upstream element-binding protein 1	151.64	0.00	166.69	318.33
LDEC004397-RA	t-complex protein 1 subunit delta	102.91	0.00	112.40	215.31
LDEC007471-RA	translocon-associated protein subunit delta	93.68	0.00	102.07	195.75
LDEC018983-RA	60s ribosomal protein l35a	256.40	0.00	278.74	535.13
LDEC006360-RA	60s ribosomal protein l27a	157.41	0.00	170.48	327.89
LDEC005882-RA	probable aconitate mitochondrial	514.69	0.00	554.85	1069.54
LDEC003786-RA	gamma-interferon-inducible lysosomal thiol reductase-like	310.32	0.00	333.52	643.84
LDEC001334-RA	zinc finger protein	81.85	0.01	87.70	169.55
LDEC005270-RA	atp synthase subunit mitochondrial	153.11	0.00	163.36	316.47
LDEC016959-RA	nadh dehydrogenase	111.81	0.00	117.32	229.13
LDEC005975-RA	imaginal disc growth factor 4 precursor	328.12	0.00	343.01	671.13
LDEC007118-RA	60s ribosomal protein l9	461.39	0.00	481.56	942.95
LDEC005806-RA	serine--trna cytoplasmic	84.66	0.00	86.85	171.51
LDEC006479-RA	muscle lim protein mlp84b-like isoform x1	1011.77	0.00	1034.29	2046.06
LDEC014796-RA	---NA---	280.88	0.00	286.49	567.37
LDEC016962-RA	cofilin actin-depolymerizing factor homolog	122.72	0.00	124.61	247.33
LDEC004861-RA	non-specific lipid-transfer protein	224.54	0.00	227.74	452.28

LDEC001762-RA	alcohol dehydrogenase class-3-like	236.16	0.00	239.21	475.37
LDEC012713-RA	steroid dehydrogenase	92.83	0.00	93.64	186.47
LDEC018534-RA	calnexin	183.73	0.00	184.92	368.65
LDEC011895-RA	adipocyte plasma membrane-associated	261.36	0.00	260.97	522.33
LDEC012590-RA	4-coumarate-- ligase 1-like	84.17	0.00	83.70	167.86
LDEC000193-RA	60s ribosomal protein l21	232.83	0.00	230.18	463.01
LDEC012374-RA	heat shock 70 kda protein cognate 5	77.50	0.01	76.37	153.88
LDEC015177-RA	small heat shock protein	115.98	0.00	113.99	229.97
LDEC008668-RA	spondin-1	99.46	0.00	96.69	196.15
LDEC002231-RA	ribosomal protein l22	317.42	0.00	304.04	621.46
LDEC001198-RA	heat shock protein 90	1333.45	0.00	1275.55	2609.00
LDEC006785-RA	probable multidrug resistance-associated protein lethal 03659	165.41	0.00	156.89	322.30
LDEC018230-RA	thioredoxin-dependent peroxide mitochondrial	85.40	0.00	80.80	166.20
LDEC000955-RA	nadh-ubiquinone oxidoreductase 39 kda subunit	107.97	0.00	102.07	210.04
LDEC019751-RA	afg3-like protein 2	92.52	0.00	87.38	179.90
LDEC005871-RA	60s ribosomal protein l37a	229.41	0.00	214.43	443.84
LDEC002421-RA	malate cytoplasmic	122.41	0.00	114.35	236.75
LDEC007608-RA	ribosomal protein l35	236.39	0.00	220.27	456.66
LDEC010053-RA	glucose dehydrogenase	144.02	0.00	134.17	278.18
LDEC008098-RA	four and a half lim domains protein 2 isoform x5	134.19	0.00	124.93	259.12
LDEC014600-RA	xanthine dehydrogenase oxidase	77.89	0.00	72.52	150.41
LDEC016748-RA	hypothetical protein D910_08753	187.14	0.00	173.56	360.70
LDEC000045-RA	ribosomal protein L39 [Danaus plexippus]	99.71	0.00	91.87	191.59
LDEC014165-RA	ribosomal protein s3	712.11	0.00	655.54	1367.65
LDEC009033-RA	long-chain-fatty-acid-- ligase 4 isoform x10	470.87	0.00	429.89	900.76
LDEC000780-RA	protein takeout	1785.65	0.00	1615.51	3401.16
LDEC006128-RA	rna-binding protein squid isoform x3	325.15	0.00	293.00	618.15
LDEC019895-RA	acyl- -binding protein	115.40	0.00	103.55	218.95
LDEC005396-RA	tubulin alpha-1 chain	3005.53	0.00	2696.91	5702.44
LDEC009165-RA	polya-binding protein	484.10	0.00	433.46	917.56
LDEC017679-RA	probable multidrug resistance-associated protein lethal 03659	82.15	0.00	73.05	155.20
LDEC006322-RA	short-chain dehydrogenase	73.81	0.01	65.61	139.43
LDEC009474-RA	chromodomain-helicase-dna-binding protein mi-2 homolog isoform x1	153.78	0.00	136.64	290.43
LDEC014126-RA	translocon-associated protein subunit alpha	228.80	0.00	202.15	430.95
LDEC003142-RA	retinoid-inducible serine carboxypeptidase-like	175.71	0.00	155.22	330.94
LDEC002053-RA	digestive cysteine protease intestain	402.93	0.00	354.72	757.65
LDEC019904-RA	isoform a	99.42	0.00	87.41	186.84
LDEC005452-RA	adenosylhomocysteinase b	98.52	0.00	86.00	184.52
LDEC001686-RA	moesin ezrin radixin	133.48	0.00	115.94	249.42
LDEC013922-RA	heterogeneous nuclear ribonucleoprotein a2 b1 homolog isoform x1	125.45	0.00	108.12	233.57
LDEC014488-RA	pdz and lim domain protein 1 isoform x2	109.97	0.00	94.67	204.64
LDEC016599-RA	ubiquitin carboxyl-terminal hydrolase 2 isoform x2	105.39	0.00	90.10	195.50

LDEC010916-RA	von willebrand factor d and egf domain-containing protein	239.79	0.00	202.75	442.54
LDEC000525-RA	serine threonine-protein phosphatase 2a 65 kda regulatory subunit a alpha isoform	128.56	0.00	108.40	236.96
LDEC018015-RA	probable aconitate mitochondrial	86.06	0.00	72.13	158.19
LDEC010780-RA	chitooligosaccharidolytic beta-n-acetylglucosaminidase	88.17	0.00	73.51	161.67
LDEC013915-RA	elongation of very long chain fatty acids protein 1-like	208.20	0.00	173.56	381.76
LDEC023381-RA	---NA---	251.95	0.00	209.90	461.86
LDEC005049-RA	pyruvate dehydrogenase e1 component subunit mitochondrial	211.32	0.00	175.82	387.14
LDEC022719-RA	adenylate kinase	82.25	0.00	67.95	150.20
LDEC003241-RA	3-hydroxyacyl-coa dehyrogenase	142.41	0.00	116.19	258.60
LDEC012448-RA	40s ribosomal protein s20	312.14	0.00	253.75	565.89
LDEC016483-RA	innexin 2	111.65	0.00	90.74	202.40
LDEC024177-RA	mitochondrial enolase superfamily member 1-like isoform x2	211.83	0.00	172.07	383.90
LDEC017885-RA	ribosomal protein l4e	98.47	0.00	79.70	178.17
LDEC024395-RA	ribosomal protein s20	115.87	0.00	93.75	209.62
LDEC020565-RA	choline-phosphate cytidylyltransferase a-like isoform x2	171.79	0.00	138.91	310.70
LDEC020740-RA	atp synthase subunit mitochondrial-like	1814.11	0.00	1457.42	3271.53
LDEC003144-RA	glutamate--cysteine ligase catalytic subunit	97.75	0.00	78.11	175.85
LDEC009090-RA	pancreatic triacylglycerol lipase-like	98.36	0.00	78.57	176.93
LDEC008159-RA	lysosomal aspartic protease	960.82	0.00	759.48	1720.30
LDEC007413-RA	heat shock protein 70	3905.80	0.00	3086.42	6992.21
LDEC010905-RA	esterase	74.43	0.00	58.78	133.22
LDEC009204-RA	methyltransferase-like protein 23	152.11	0.00	119.94	272.05
LDEC010935-RA	#NAME?	66.87	0.01	52.63	119.50
LDEC006619-RA	protein disulfide-isomerase	130.90	0.00	102.63	233.53
LDEC017313-RA	transformer-2 protein homolog beta isoform x2	74.29	0.00	57.76	132.05
LDEC007395-RA	#NAME?	81.27	0.00	63.07	144.33
LDEC016520-RA	dnaj homolog subfamily b member 11	71.15	0.00	55.17	126.32
LDEC000085-RA	spectrin beta chain isoform x4	720.41	0.00	558.00	1278.41
LDEC016496-RA	cytochrome c oxidase subunit 6c	86.04	0.00	66.36	152.40
LDEC017247-RA	glutamine synthetase 2	93.96	0.00	72.44	166.41
LDEC008853-RA	elongation factor 1 gamma	406.06	0.00	310.94	717.01
LDEC003947-RA	very long-chain-fatty-acid-- ligase bubblegum isoform x1	77.85	0.00	59.10	136.95
LDEC000846-RA	fatty acid-binding protein 12-like	484.50	0.00	364.38	848.88
LDEC005909-RA	ubiquitin-like modifier-activating enzyme 1-like	240.56	0.00	180.46	421.02
LDEC011628-RA	restin homolog	90.92	0.00	67.63	158.55
LDEC000084-RA	40s ribosomal protein sa	68.37	0.00	50.79	119.15
LDEC014808-RA	dna topoisomerase mitochondrial isoform x3	164.35	0.00	121.46	285.81
LDEC002501-RA	60s ribosomal protein l6	179.17	0.00	132.40	311.56
LDEC007028-RA	probable multidrug resistance-associated protein lethal 03659	64.99	0.01	47.21	112.20
LDEC001224-RA	glycosyl hydrolase	105.89	0.00	76.48	182.37
LDEC003400-RA	myosin light chain alkali isoform x1	821.46	0.00	592.79	1414.26
LDEC002557-RA	juvenile hormone partial	178.68	0.00	128.08	306.75

LDEC004703-RA	swi snf complex subunit smarcc2	69.08	0.00	49.16	118.23
LDEC006513-RA	10 kda heat shock mitochondrial	87.75	0.00	62.18	149.93
LDEC019597-RA	t-complex protein 1 subunit alpha	85.69	0.00	60.52	146.21
LDEC004427-RA	serine-arginine protein 55-like isoform x2	131.53	0.00	92.44	223.97
LDEC003275-RA	splicing arginine serine-rich 2	96.86	0.00	67.45	164.32
LDEC015088-RA	acyl- dehydrogenase	116.47	0.00	81.08	197.55
LDEC006357-RA	glycoside hydrolase family protein 48	2013.54	0.00	1396.66	3410.19
LDEC014876-RA	abc transporter g family member 23-like isoform x2	136.89	0.00	94.85	231.73
LDEC000483-RA	calcium-transporting atpase sarcoplasmic endoplasmic reticulum type isoform x1	1162.25	0.00	804.29	1966.54
LDEC010852-RA	tubulin beta-1 chain	2528.03	0.00	1748.83	4276.86
LDEC004990-RA	glutathione s-transferase	400.63	0.00	277.11	677.74
LDEC015453-RA	atp synthase b mitochondrial	173.24	0.00	118.74	291.98
LDEC013633-RA	mitochondrial enolase superfamily member 1-like isoform x2	343.53	0.00	235.35	578.88
LDEC017682-RA	probable multidrug resistance-associated protein lethal 03659	161.75	0.00	110.49	272.24
LDEC008607-RA	enolase	543.19	0.00	370.08	913.27
LDEC010269-RA	alanine aminotransferase 2 isoform x2	129.32	0.00	87.70	217.02
LDEC010407-RA	juvenile hormone epoxide hydrolase-like protein 2	129.66	0.00	87.87	217.53
LDEC018298-RA	chymotrypsin inhibitor-like	175.52	0.00	118.66	294.18
LDEC021246-RA	nadh dehydrogenase	215.99	0.00	145.67	361.66
LDEC009655-RA	chondroitin proteoglycan-2	131.44	0.00	88.58	220.02
LDEC011582-RA	tropomyosin 1	543.40	0.00	365.94	909.34
LDEC016646-RA	protein ergic-53	111.93	0.00	75.35	187.28
LDEC000979-RA	60s ribosomal protein l31	413.76	0.00	277.29	691.04
LDEC004603-RA	peroxiredoxin prdx5	125.19	0.00	83.42	208.60
LDEC017475-RA	arginine kinase	2265.51	0.00	1505.87	3771.39
LDEC002247-RA	eukaryotic translation initiation factor 2 subunit 1	61.34	0.01	40.45	101.79
LDEC019168-RA	protein anoxia up-regulated isoform x4	127.61	0.00	83.91	211.52
LDEC012234-RA	cellular fabp-like protein isoform 1 precursor	143.76	0.00	94.28	238.04
LDEC019869-RA	glucose dehydrogenase	141.79	0.00	92.69	234.47
LDEC013825-RA	cytosolic non-specific dipeptidase	107.89	0.00	70.32	178.21
LDEC015672-RA	troponin c	139.89	0.00	89.72	229.61
LDEC013728-RA	basement membrane-specific heparan sulfate proteoglycan core protein isoform x8	298.62	0.00	191.43	490.04
LDEC004781-RA	cytochrome c oxidase polypeptide iv	474.04	0.00	297.14	771.18
LDEC007953-RA	protein d2-like isoform x2	182.10	0.00	114.10	296.20
LDEC011303-RA	beta- -glucanase	139.20	0.00	86.42	225.62
LDEC014332-RA	muscle-specific protein 20-like	91.75	0.00	56.62	148.38
LDEC014278-RA	beta-galactosidase-1-like protein 2	89.40	0.00	54.61	144.01
LDEC010502-RA	esterase	80.54	0.00	49.19	129.73
LDEC004810-RA	galectin-4-like isoform x2	71.65	0.00	43.71	115.36
LDEC000239-RA	serine protease	219.94	0.00	132.61	352.55
LDEC021660-RA	gamma-glutamyltranspeptidase 1 isoform x2	61.00	0.00	36.77	97.77
LDEC008881-RA	acetyl- mitochondrial	115.11	0.00	69.12	184.23

LDEC005684-RA	cathepsin l precursor	98.93	0.00	59.31	158.24
LDEC018966-RA	glyoxylate reductase hydroxypyruvate reductase-like	134.14	0.00	80.27	214.41
LDEC003973-RA	hypothetical protein YQE_00903, partial	364.91	0.00	217.65	582.56
LDEC005731-RA	thioredoxin domain-containing protein 17-like	135.14	0.00	80.23	215.37
LDEC013324-RA	60s acidic ribosomal protein p1	976.30	0.00	574.18	1550.48
LDEC018229-RA	chitinase 1	313.14	0.00	182.86	496.00
LDEC006014-RA	ribosomal protein l10ae	699.03	0.00	408.09	1107.12
LDEC000077-RA	voltage-dependent anion channel	212.19	0.00	123.69	335.88
LDEC008467-RA	60s ribosomal protein l44	379.87	0.00	219.85	599.71
LDEC010338-RA	pantothenate kinase mitochondrial-like	74.56	0.00	43.14	117.70
LDEC009139-RA	digestive cysteine proteinase intestain	1520.60	0.00	878.61	2399.21
LDEC002639-RA	immediate early response gene 5-like protein	56.08	0.01	31.75	87.82
LDEC021669-RA	40s ribosomal protein s2	1009.92	0.00	568.94	1578.86
LDEC006952-RA	translocon-associated protein subunit gamma	139.59	0.00	78.46	218.05
LDEC002529-RA	acetyl-coa carboxylase	200.17	0.00	112.12	312.29
LDEC003498-RA	beta-amyloid-like partial	59.31	0.00	33.13	92.44
LDEC018545-RA	troponin i	995.84	0.00	552.13	1547.97
LDEC011920-RA	tho complex subunit 4	96.56	0.00	53.37	149.93
LDEC023006-RA	ribosomal protein s27e	239.16	0.00	132.11	371.27
LDEC001282-RA	protein arginine n-methyltransferase 1 isoform x1	126.86	0.00	69.90	196.76
LDEC014599-RA	indole-3-acetaldehyde oxidase-like	92.77	0.00	50.93	143.70
LDEC011014-RA	peroxisomal multifunctional enzyme type 2	296.58	0.00	162.23	458.81
LDEC005652-RA	mucin- partial	200.77	0.00	109.18	309.95
LDEC002666-RA	unc93-like protein	68.09	0.00	36.98	105.07
LDEC014453-RA	udp-glucuronosyltransferase 2b16	58.69	0.00	31.82	90.50
LDEC007893-RA	atp-citrate synthase isoform x1	706.53	0.00	382.08	1088.61
LDEC004900-RA	superoxide dismutase	135.64	0.00	73.33	208.97
LDEC007807-RA	la-related protein 6	428.62	0.00	231.21	659.82
LDEC000699-RA	lipid storage droplets surface-binding protein 1 isoform x1	350.85	0.00	188.88	539.73
LDEC004433-RA	oligosaccharyl transferase	176.33	0.00	94.67	271.00
LDEC010980-RA	guanine nucleotide-binding protein g g g subunit beta-1	60.12	0.00	32.17	92.29
LDEC020917-RA	6-phosphogluconate decarboxylating	81.24	0.00	43.32	124.56
LDEC018480-RA	probable enoyl- mitochondrial-like	624.93	0.00	332.81	957.75
LDEC016118-RA	hypoxia up-regulated protein 1 isoform x1	196.71	0.00	104.76	301.47
LDEC014917-RA	ribosomal protein l36e	259.59	0.00	138.13	397.72
LDEC003254-RA	glycoside hydrolase family 28	957.76	0.00	508.35	1466.11
LDEC017886-RA	ribosomal protein l4	1338.35	0.00	704.84	2043.19
LDEC021639-RA	nadh-ubiquinone oxidoreductase 75 kda subunit	128.76	0.00	67.60	196.36
LDEC013727-RA	basement membrane-specific heparan sulfate proteoglycan core protein isoform x3	68.38	0.00	35.67	104.05
LDEC010356-RA	synaptic vesicle glycoprotein 2c-like isoform x1	112.25	0.00	58.39	170.64
LDEC014837-RA	uncharacterized threonine-rich gpi-anchored glyco	179.23	0.00	93.18	272.42
LDEC004242-RA	PREDICTED: uncharacterized protein LOC103314372	238.71	0.00	124.08	362.79

LDEC010761-RA	6-phosphogluconate decarboxylating	491.09	0.00	251.84	742.93
LDEC014467-RA	hypothetical protein D910_05825	161.77	0.00	82.71	244.48
LDEC020611-RA	laminin subunit beta-1	280.16	0.00	143.08	423.24
LDEC004140-RA	cyclin-dependent kinase 12 isoform x3	77.99	0.00	39.71	117.70
LDEC011752-RA	hypothetical protein TcasGA2_TC015315	96.19	0.00	48.70	144.89
LDEC013437-RA	isoform a	67.99	0.00	34.40	102.39
LDEC024513-RA	ribosomal protein s8e	930.74	0.00	470.87	1401.61
LDEC009495-RA	probable adenylate kinase isoenzyme	80.64	0.00	40.70	121.34
LDEC021225-RA	phosphatidylserine decarboxylase	57.44	0.00	28.95	86.38
LDEC011802-RA	polypyrimidine tract-binding protein 1 isoform x3	70.47	0.00	35.50	105.97
LDEC019996-RA	cartilage oligomeric matrix protein	172.91	0.00	87.03	259.94
LDEC007477-RA	lipoyltransferase mitochondrial	157.55	0.00	78.53	236.08
LDEC002531-RA	acetyl- carboxylase isoform x2	63.64	0.00	31.67	95.31
LDEC014750-RA	probable isocitrate dehydrogenase	150.70	0.00	74.74	225.45
LDEC010875-RA	cathepsin b	74.72	0.00	37.05	111.78
LDEC020664-RA	serine protease snake-like	80.55	0.00	39.92	120.48
LDEC003818-RA	alpha-n-acetylgalactosaminidase-like isoform x1	67.74	0.00	33.51	101.26
LDEC016736-RA	hypothetical protein KGM_03566	312.84	0.00	154.55	467.39
LDEC002142-RA	y-box factor homolog isoform x1	1011.09	0.00	497.80	1508.90
LDEC018191-RA	60s ribosomal protein l8	688.99	0.00	338.97	1027.96
LDEC000730-RA	mitochondrial amidoxime reducing component 2	60.84	0.00	29.83	90.68
LDEC015475-RA	sodium-coupled monocarboxylate transporter 1	176.23	0.00	86.14	262.37
LDEC009415-RA	probable medium-chain specific acyl- mitochondrial	247.29	0.00	119.73	367.02
LDEC007078-RA	protein disulfide isomerase	733.94	0.00	354.26	1088.20
LDEC001114-RA	5-aminolevulinate mitochondrial	102.83	0.00	49.23	152.06
LDEC000581-RA	nadh dehydrogenase	50.18	0.01	23.75	73.93
LDEC002528-RA	acetyl- carboxylase isoform x3	89.68	0.00	42.29	131.97
LDEC017194-RA	procollagen- -oxoglutarate 5-dioxygenase 3-like	54.18	0.00	25.48	79.66
LDEC002048-RA	tp53-regulating kinase	83.38	0.00	39.14	122.53
LDEC001027-RA	translocon-associated protein subunit beta	114.75	0.00	53.26	168.02
LDEC002633-RA	glypican-6 isoform x1	49.76	0.01	23.07	72.84
LDEC008711-RA	mfs-type transporter c6orf192 homolog	56.20	0.00	26.05	82.25
LDEC010888-RA	cathepsin b	79.38	0.00	36.56	115.93
LDEC009998-RA	mdl2	85.76	0.00	39.39	125.15
LDEC016681-RA	triosephosphate isomerase	207.76	0.00	95.20	302.96
LDEC019925-RA	cg1561	79.74	0.00	36.52	116.26
LDEC005003-RA	zinc transporter zip1	86.48	0.00	39.46	125.94
LDEC000877-RA	nadp-dependent malic enzyme isoform x2	207.52	0.00	94.35	301.87
LDEC004606-RA	ribonucleoside-diphosphate reductase large subunit	71.64	0.00	32.52	104.17
LDEC007619-RA	heat shock protein 90	436.43	0.00	197.44	633.88
LDEC008944-RA	aromatic-l-amino-acid decarboxylase-like	62.72	0.00	28.21	90.93
LDEC018333-RA	serine protease	117.75	0.00	52.66	170.41
LDEC010233-RA	acyl- synthetase short-chain family member mitochondrial	170.15	0.00	76.09	246.24

LDEC002595-RA	acyl-coa thioesterase	57.38	0.00	25.55	82.94
LDEC011579-RA	tropomyosin 1	909.71	0.00	404.27	1313.98
LDEC007378-RA	short-chain dehydrogenease reductase	126.44	0.00	56.16	182.60
LDEC001419-RA	na+ + atpase alpha-subunit partial	64.52	0.00	28.63	93.15
LDEC010881-RA	cathepsin b	233.91	0.00	103.73	337.64
LDEC015119-RA	regucalcin-like isoform x1	182.51	0.00	80.76	263.27
LDEC014931-RA	cytosolic 10-formyltetrahydrofolate dehydrogenase	67.47	0.00	29.83	97.31
LDEC011415-RA	methylcrotonoyl- carboxylase subunit mitochondrial isoform x2	64.88	0.00	28.56	93.44
LDEC009853-RA	glyceraldehyde-3-phosphate dehydrogenase	976.54	0.00	429.85	1406.40
LDEC020299-RA	peptidyl-prolyl cis-trans isomerase 5	230.27	0.00	100.97	331.24
LDEC002665-RA	unc93-like protein	117.55	0.00	51.35	168.90
LDEC005923-RA	farnesoic acid o-methyltransferase-like protein	480.45	0.00	208.27	688.72
LDEC017593-RA	voltage-dependent anion channel	493.13	0.00	213.51	706.64
LDEC009454-RA	glycosyl hydrolase	83.38	0.00	36.06	119.44
LDEC016638-RA	t-complex protein 1 subunit gamma	192.41	0.00	82.92	275.33
LDEC013730-RA	basement membrane-specific heparan sulfate proteoglycan core protein isoform x14	307.81	0.00	131.09	438.90
LDEC004918-RA	succinate dehydrogenase	67.65	0.00	28.70	96.35
LDEC019657-RA	sterol o-acyltransferase 1-like	51.34	0.00	21.77	73.11
LDEC014169-RA	ribosomal protein s23	679.28	0.00	287.05	966.33
LDEC007396-RA	serine protease h3	132.17	0.00	55.56	187.74
LDEC000757-RA	endo-beta- -glucanase	352.20	0.00	147.54	499.74
LDEC009388-RA	eukaryotic translation initiation factor 4 gamma 3-like isoform x3	486.30	0.00	203.28	689.58
LDEC009032-RA	protein transport protein sec61 subunit beta	142.82	0.00	59.63	202.45
LDEC004844-RA	glycoside hydrolase family protein 48	183.47	0.00	76.41	259.88
LDEC011408-RA	udp-glucose:glycoprotein glucosyltransferase	58.65	0.00	24.38	83.03
LDEC017203-RA	aarf domain containing kinase 5	73.04	0.00	30.19	103.23
LDEC001223-RA	glycosyl hydrolase	308.89	0.00	127.26	436.16
LDEC012345-RA	dolichyl-diphosphooligosaccharide--protein glycosyltransferase subunit 2	304.71	0.00	124.11	428.82
LDEC008120-RA	endopolygalacturonase	130.95	0.00	52.84	183.79
LDEC002556-RA	juvenile hormone partial	205.51	0.00	82.18	287.69
LDEC021576-RA	multidrug resistance protein 1a	54.28	0.00	21.41	75.69
LDEC004666-RA	2-hydroxyacylsphingosine 1-beta-galactosyltransferase-like	128.79	0.00	50.79	179.57
LDEC015597-RA	serine-arginine protein 55 isoform x7	203.43	0.00	79.98	283.41
LDEC001118-RA	transketolase-like protein 2 isoform x1	876.83	0.00	344.03	1220.87
LDEC014671-RA	40s ribosomal protein s11-like	346.15	0.00	134.94	481.10
LDEC015972-RA	myosin-2 essential light chain isoform x2	107.72	0.00	41.97	149.70
LDEC008468-RA	---NA---	692.40	0.00	268.01	960.41
LDEC015100-RA	rrna 2 -o-methyltransferase fibrillarin	67.01	0.00	25.87	92.88
LDEC013118-RA	protein disulfide-isomerase	395.01	0.00	151.54	546.55
LDEC003256-RA	glycoside hydrolase family 28	333.68	0.00	126.95	460.63
LDEC016660-RA	60s ribosomal protein l34	147.72	0.00	56.13	203.85
LDEC005707-RA	ribosomal protein l27ae	73.91	0.00	27.96	101.87

LDEC012445-RA	low quality protein: water dikinase	228.68	0.00	85.61	314.29
LDEC001619-RA	synaptic vesicle glycoprotein 2b-like	94.00	0.00	35.18	129.17
LDEC019169-RA	tyrosine 3-monooxygenase	50.23	0.00	18.76	68.99
LDEC017279-RA	salicyl alcohol oxidase paralog 1	57.66	0.00	21.45	79.10
LDEC007478-RA	PREDICTED: uncharacterized protein LOC102674856	68.75	0.00	25.34	94.09
LDEC013444-RA	inorganic phosphate cotransporter isoform x1	289.25	0.00	104.61	393.87
LDEC002057-RA	digestive cysteine protease intestain	1668.31	0.00	600.79	2269.10
LDEC009005-RA	ruvb-like 2	48.47	0.00	17.45	65.92
LDEC003946-RA	very long-chain-fatty-acid-- ligase bubblegum isoform x1	232.89	0.00	83.77	316.66
LDEC007709-RA	esterase b1-like	61.89	0.00	22.15	84.05
LDEC002622-RA	elongation factor mitochondrial	62.18	0.00	22.12	84.30
LDEC013729-RA	basement membrane-specific heparan sulfate proteoglycan core protein isoform x6	319.39	0.00	112.65	432.04
LDEC003115-RA	esterase	56.90	0.00	19.96	76.86
LDEC010878-RA	cathepsin b precursor	54.70	0.00	19.04	73.74
LDEC008426-RA	transmembrane protein 53	86.04	0.00	29.80	115.84
LDEC021583-RA	ump-cmp kinase	87.66	0.00	30.12	117.77
LDEC001815-RA	cuticular protein rr-1 family	62.32	0.00	21.31	83.63
LDEC023680-RA	aconitate mitochondrial-like isoform x2	92.74	0.00	31.60	124.35
LDEC006148-RA	angiotensin-converting enzyme-like	77.91	0.00	26.51	104.42
LDEC022336-RA	60 kda heat shock mitochondrial-like	273.25	0.00	91.87	365.12
LDEC006127-RA	---NA---	54.11	0.00	18.01	72.13
LDEC016525-RA	paxillin isoform x5	199.81	0.00	66.36	266.17
LDEC013286-RA	alpha- partial	104.76	0.00	34.65	139.41
LDEC000982-RA	40s ribosomal protein s2	1804.92	0.00	594.77	2399.69
LDEC009942-RA	phosphoglycerate kinase	99.88	0.00	32.59	132.47
LDEC009157-RA	heat shock cognate 70	762.89	0.00	247.35	1010.23
LDEC015842-RA	probable methylmalonate-semialdehyde dehydrogenase	95.80	0.00	31.04	126.84
LDEC007616-RA	atp-binding cassette sub-family d member 3	147.03	0.00	47.32	194.35
LDEC019595-RA	neurexin-4 isoform x1	52.45	0.00	16.67	69.12
LDEC009387-RA	eukaryotic translation initiation factor 4 gamma 3-like isoform x3	103.17	0.00	32.63	135.81
LDEC017672-RA	probable multidrug resistance-associated protein lethal 03659	47.37	0.00	14.97	62.34
LDEC009679-RA	orcokinin precursor isoform b	47.66	0.00	15.01	62.66
LDEC011312-RA	hypothetical protein YQE_05172, partial	7459.72	0.00	2344.95	9804.66
LDEC022234-RA	serine protease easter	89.08	0.00	27.89	116.97
LDEC002361-RA	agap002583-pa-like protein	234.69	0.00	73.05	307.73
LDEC017189-RA	serine threonine-protein kinase polo	50.80	0.00	15.75	66.55
LDEC008648-RA	agap005332-pc-like protein	170.59	0.00	52.52	223.11
LDEC018713-RA	protein transport protein sec61 subunit gamma	55.45	0.00	16.99	72.43
LDEC013647-RA	facilitated trehalose transporter tret1-2 homolog	191.19	0.00	58.57	249.76
LDEC008758-RA	scavenger receptor class b member 1 isoform x2	63.57	0.00	19.46	83.03
LDEC023974-RA	muscular protein 20	207.84	0.00	63.63	271.48
LDEC009536-RA	protein ovo isoform x2	75.58	0.00	23.11	98.69

LDEC015417-RA	pupal cuticle protein 36-like isoform x2	270.52	0.00	82.57	353.09
LDEC001589-RA	odorant binding protein	43.03	0.01	13.09	56.13
LDEC020000-RA	12 kda fk506-binding protein	207.65	0.00	63.17	270.82
LDEC016089-RA	collagen alpha- chain	1955.29	0.00	592.33	2547.63
LDEC013405-RA	fibrillin-2 isoform x2	51.77	0.00	15.68	67.45
LDEC013817-RA	venom carboxylesterase-6	72.47	0.00	21.94	94.41
LDEC019971-RA	gamma-interferon-inducible lysosomal thiol reductase-like	145.42	0.00	43.81	189.23
LDEC005079-RA	inorganic phosphate cotransporter	103.38	0.00	30.90	134.27
LDEC019515-RA	serine protease p98	49.79	0.00	14.86	64.65
LDEC019589-RA	troponin t isoform x2	3785.18	0.00	1127.01	4912.19
LDEC012332-RA	cytochrome p450-like protein	87.16	0.00	25.76	112.93
LDEC020558-RA	glutathione s transferase e6	197.43	0.00	58.25	255.69
LDEC003450-RA	endocuticle structural glycoprotein bd-8-like	46.18	0.00	13.59	59.77
LDEC014104-RA	elongation of very long chain fatty acids protein aael008004	41.97	0.01	12.35	54.33
LDEC010381-RA	heat shock protein 90	80.98	0.00	23.82	104.80
LDEC024655-RA	adipocyte plasma membrane-associated	44.17	0.00	12.99	57.16
LDEC007394-RA	mast cell protease 6	175.44	0.00	51.56	227.00
LDEC006874-RA	inter-alpha-trypsin inhibitor heavy chain h4-like isoform x2	160.67	0.00	47.07	207.74
LDEC018788-RA	alanine--trna cytoplasmic	101.87	0.00	29.83	131.70
LDEC020822-RA	muscle actin	15669.61	0.00	4582.48	20252.10
LDEC001608-RA	scavenger receptor class b member 1	126.07	0.00	36.70	162.77
LDEC006875-RA	inter-alpha-trypsin inhibitor heavy chain h4-like isoform x1	51.58	0.00	15.01	66.59
LDEC001987-RA	upstream activation factor subunit spp27	464.47	0.00	133.88	598.35
LDEC011092-RA	udp-glucuronosyltransferase 2b7	64.56	0.00	18.54	83.11
LDEC008865-RA	cuticular protein analogous to peritrophins 1-c precursor	106.15	0.00	30.12	136.27
LDEC011583-RA	tropomyosin- partial	673.30	0.00	190.97	864.27
LDEC019801-RA	d-beta-hydroxybutyrate mitochondrial	62.58	0.00	17.73	80.31
LDEC016324-RA	PREDICTED: uncharacterized protein LOC657683	94.91	0.00	26.83	121.74
LDEC024625-RA	alcohol dehydrogenase	40.29	0.01	11.25	51.55
LDEC023039-RA	esterase	63.89	0.00	17.84	81.73
LDEC023888-RA	acyl-coa thioesterase	48.92	0.00	13.59	62.51
LDEC014890-RA	tubulointerstitial nephritis	108.28	0.00	29.73	138.01
LDEC005648-RA	glucose dehydrogenase	72.89	0.00	20.00	92.88
LDEC003945-RA	very long-chain-fatty-acid-- ligase bubblegum isoform x1	395.12	0.00	108.22	503.34
LDEC006352-RA	alpha- sarcomeric	309.94	0.00	84.62	394.56
LDEC000731-RA	tyrosine-protein phosphatase 10d isoform x1	941.57	0.00	256.97	1198.54
LDEC023585-RA	PREDICTED: uncharacterized protein LOC103578083 isoform X1	94.59	0.00	25.69	120.28
LDEC009538-RA	calumenin	300.45	0.00	81.15	381.60
LDEC016968-RA	type i cytoskeletal 9 isoform x2	63.33	0.00	17.02	80.35
LDEC024072-RA	glycoside hydrolase family 45 protein	226.41	0.00	60.84	287.25
LDEC020286-RA	glycoside hydrolase family 45 protein	173.44	0.00	46.04	219.49
LDEC012841-RA	odorant-binding protein 17	433.58	0.00	114.74	548.32

LDEC003829-RA	protein transport protein sec61 subunit alpha isoform 2	857.18	0.00	225.33	1082.51
LDEC006147-RA	angiotensin-converting enzyme-like	82.12	0.00	21.38	103.50
LDEC017157-RA	hypothetical protein TcasGA2_TC015372	263.94	0.00	68.37	332.32
LDEC016658-RA	nadh dehydrogenase	91.71	0.00	23.61	115.32
LDEC014088-RA	udp-glucuronosyltransferase 2b9	196.54	0.00	50.43	246.97
LDEC007780-RA	agap003453-pa-like protein	43.23	0.00	11.08	54.31
LDEC011968-RA	kynurenine--oxoglutarate transaminase 3 isoform x2	348.71	0.00	89.29	438.00
LDEC003785-RA	gamma-interferon-inducible lysosomal thiol reductase-like	1110.04	0.00	282.52	1392.56
LDEC021798-RA	gamma-interferon-inducible lysosomal thiol reductase-like	836.85	0.00	212.91	1049.76
LDEC009339-RA	PREDICTED: uncharacterized protein LOC663405 isoform X2	38.89	0.01	9.80	48.69
LDEC018086-RA	collagen alpha-2 chain-like	611.46	0.00	153.28	764.74
LDEC007262-RA	insulin-like growth factor 2 mrna-binding protein 1 isoform x4	81.93	0.00	20.49	102.42
LDEC006878-RA	inter-alpha-trypsin inhibitor heavy chain h4-like isoform x1	308.66	0.00	77.01	385.67
LDEC015188-RA	1-acylglycerol-3-phosphate o-acyltransferase abhd5-like isoform x2	41.05	0.00	10.16	51.20
LDEC014168-RA	aminopeptidase n	112.69	0.00	27.85	140.54
LDEC009158-RA	heat shock protein 70	1008.42	0.00	247.17	1255.59
LDEC022329-RA	cytochrome p450	78.73	0.00	19.29	98.02
LDEC003910-RA	peroxisomal acyl-coenzyme a oxidase 3	124.40	0.00	30.44	154.83
LDEC014331-RA	PREDICTED: myophilin	213.55	0.00	51.81	265.36
LDEC005442-RA	unknown	210.97	0.00	51.14	262.11
LDEC010000-RA	PREDICTED: uncharacterized protein LOC655909 isoform X2	38.52	0.01	9.31	47.83
LDEC010503-RA	juvenile hormone esterase isoform a	124.23	0.00	29.62	153.86
LDEC022012-RA	40s ribosomal protein sa	214.59	0.00	50.68	265.27
LDEC003972-RA	myofilin variant a	1084.45	0.00	252.34	1336.78
LDEC010948-RA	hypothetical protein D910_00165	306.03	0.00	70.25	376.28
LDEC002153-RA	glycerol-3-phosphate mitochondrial isoform x1	285.53	0.00	65.22	350.75
LDEC011066-RA	RT01145p	77.42	0.00	17.59	95.01
LDEC012851-RA	hydroxyacyl-coenzyme a mitochondrial	245.43	0.00	55.60	301.02
LDEC000451-RA	ethanolamine-phosphate cytidylyltransferase	90.22	0.00	20.17	110.40
LDEC017984-RA	basement membrane-specific heparan sulfate proteoglycan core protein	58.56	0.00	12.95	71.51
LDEC001999-RA	isoform a	173.86	0.00	38.40	212.26
LDEC024311-RA	inactive pancreatic lipase-related protein 1	43.25	0.00	9.52	52.77
LDEC006881-RA	inter-alpha-trypsin inhibitor heavy chain h4-like isoform x1	50.57	0.00	11.04	61.61
LDEC012651-RA	PREDICTED: uncharacterized protein LOC103312147	68.58	0.00	14.97	83.55
LDEC000758-RA	endo-beta- -glucanase	923.05	0.00	200.45	1123.50
LDEC016367-RA	agap004199-pa-like protein	52.80	0.00	11.47	64.27
LDEC000535-RA	lysyl oxidase homolog 3	39.86	0.00	8.60	48.46
LDEC016867-RA	af117576_1kazal-type proteinase inhibitor	67.50	0.00	14.47	81.98
LDEC010734-RA	apolipoprotein d-like	358.61	0.00	75.88	434.49
LDEC014789-RA	endo-beta- -glucanase	1227.37	0.00	258.46	1485.83
LDEC013889-RA	inactive pancreatic lipase-related protein 1	133.10	0.00	27.92	161.02
LDEC015477-RA	sodium-coupled monocarboxylate transporter 1	167.62	0.00	35.14	202.76

LDEC009735-RA	juvenile hormone binding protein partial	2794.19	0.00	585.43	3379.63
LDEC016254-RA	myosin light chain 2	3556.23	0.00	737.51	4293.74
LDEC006412-RA	prostaglandin reductase 1	60.94	0.00	12.56	73.51
LDEC009180-RA	lysozyme i-2	155.83	0.00	31.92	187.75
LDEC011160-RA	juvenile hormone binding protein partial	3087.34	0.00	625.60	3712.94
LDEC011117-RA	actin	669.92	0.00	133.53	803.45
LDEC010420-RA	24-dehydrocholesterol reductase	49.60	0.00	9.80	59.40
LDEC004034-RA	acetyl-coenzyme a synthetase	84.67	0.00	16.70	101.37
LDEC018087-RA	collagen alpha-5 chain	487.70	0.00	94.95	582.66
LDEC014790-RA	glycoside hydrolase family 45 protein	117.88	0.00	22.79	140.67
LDEC020469-RA	peptidyl-prolyl cis-trans isomerase fkbp14- partial	300.96	0.00	57.90	358.86
LDEC007862-RA	14-3-3 epsilon protein	143.71	0.00	27.57	171.28
LDEC001225-RA	ornithine decarboxylase 1-like	383.82	0.00	72.69	456.51
LDEC012711-RA	fk506-binding protein 2 isoform x1	80.76	0.00	15.15	95.91
LDEC013137-RA	laminin subunit gamma-1 isoform x2	71.40	0.00	13.20	84.60
LDEC009332-RA	probable cytochrome p450 mitochondrial isoform x1	35.24	0.01	6.48	41.72
LDEC000777-RA	craniofacial development protein 2-like	256.53	0.00	47.14	303.67
LDEC003087-RA	citrate synthase	2313.14	0.00	422.49	2735.63
LDEC015759-RA	chitinase 5 precursor	319.16	0.00	58.29	377.45
LDEC015476-RA	sodium-coupled monocarboxylate transporter 1	85.90	0.00	15.68	101.58
LDEC020880-RA	protein yellow-like isoform x2	525.28	0.00	95.63	620.91
LDEC015496-RA	integument esterase	85.33	0.00	15.50	100.83
LDEC014889-RA	uncharacterized peptidase c1-like protein	409.82	0.00	73.79	483.61
LDEC007614-RA	low quality protein: calreticulin-like	3284.19	0.00	580.19	3864.38
LDEC019877-RA	aldo-keto reductase	87.19	0.00	15.29	102.48
LDEC020078-RA	venom serine carboxypeptidase	199.80	0.00	35.00	234.80
LDEC000262-RA	udp-glucuronosyltransferase 2c1-like	93.45	0.00	16.28	109.72
LDEC010984-RA	trypsin 7	287.64	0.00	50.04	337.68
LDEC012853-RA	midgut chitinase	76.13	0.00	13.17	89.30
LDEC011144-RA	PREDICTED: uncharacterized protein LOC662719	61.02	0.00	10.51	71.53
LDEC006909-RA	endocuticle structural glycoprotein bd-2-like	179.94	0.00	30.51	210.44
LDEC014791-RA	glycoside hydrolase family 45 protein	366.90	0.00	60.77	427.67
LDEC000773-RA	histone	136.76	0.00	22.61	159.38
LDEC021311-RA	pacifastin-like protease inhibitor cvp4	109.57	0.00	17.98	127.55
LDEC016722-RA	protein ctla-2-alpha	184.68	0.00	30.19	214.87
LDEC021797-RA	gamma-interferon-inducible lysosomal thiol reductase-like	451.70	0.00	73.44	525.13
LDEC016032-RA	cytochrome p450 9z4	339.77	0.00	54.25	394.02
LDEC012761-RA	seminal fluid partial	86.64	0.00	13.73	100.37
LDEC012334-RA	cytochrome p450 4c3-like	76.53	0.00	12.00	88.53
LDEC024465-RA	myosin regulatory light chain 2	551.35	0.00	86.03	637.39
LDEC018935-RA	heat shock 70 kda protein cognate 3 isoform x1	2235.67	0.00	347.29	2582.96
LDEC019995-RA	cartilage oligomeric matrix protein	211.89	0.00	32.67	244.55
LDEC022867-RA	venom acid phosphatase acph-1-like	151.62	0.00	23.36	174.97

LDEC017589-RA	93 kda serpin	40.45	0.00	6.23	46.68
LDEC023294-RA	sphingomyelin phosphodiesterase-like	43.77	0.00	6.72	50.49
LDEC017191-RA	procollagen- -oxoglutarate 5-dioxygenase 3	141.95	0.00	21.66	163.61
LDEC021558-RA	venom acid phosphatase acph-1-like	74.70	0.00	11.15	85.85
LDEC002263-RA	ef-hand calcium-binding domain-containing protein 1	872.75	0.00	129.53	1002.28
LDEC012852-RA	probable chitinase 2 isoform x2	131.80	0.00	19.22	151.02
LDEC004919-RA	serine protease persephone-like	66.78	0.00	9.70	76.48
LDEC007822-RA	---NA---	152.47	0.00	22.12	174.59
LDEC020510-RA	histone h2a-like	92.10	0.00	13.24	105.34
LDEC016473-RA	cuticular protein analogous to peritrophins 3-e	237.59	0.00	33.90	271.50
LDEC006832-RA	cell wall protein dan4	72.20	0.00	10.30	82.49
LDEC005376-RA	serine protease gd-like isoform x2	54.29	0.00	7.64	61.93
LDEC007021-RA	agap006497-pa-like protein	916.68	0.00	128.75	1045.43
LDEC017855-RA	alpha-n-acetylgalactosaminidase-like isoform x2	131.22	0.00	18.19	149.41
LDEC006784-RA	zinc carboxypeptidase	68.69	0.00	9.38	78.07
LDEC008947-RA	aromatic-l-amino-acid decarboxylase-like	33.51	0.01	4.57	38.08
LDEC005692-RA	cathepsin l-like proteinase	258.33	0.00	35.07	293.40
LDEC018831-RA	odorant binding protein	63.79	0.00	8.56	72.36
LDEC004033-RA	acetyl coenzyme a isoform b	48.69	0.00	6.48	55.17
LDEC014836-RA	facilitated trehalose transporter tret1	38.73	0.00	5.13	43.86
LDEC005370-RA	mucin 12ea	536.37	0.00	71.06	607.43
LDEC007593-RA	blackjack	657.18	0.00	86.60	743.78
LDEC018549-RA	hexosaminidase 1 isoform x1	112.66	0.00	14.83	127.49
LDEC003042-RA	protein disulfide-isomerase a3	47.25	0.00	6.12	53.37
LDEC008919-RA	ribonucleoside-diphosphate reductase subunit m2 b	74.57	0.00	9.56	84.12
LDEC001337-RA	probable serine threonine-protein kinase nek3 isoform x1	52.76	0.00	6.76	59.52
LDEC019760-RA	membrane-bound alkaline phosphatase	71.84	0.00	9.06	80.90
LDEC017853-RA	neutral alpha-glucosidase ab	164.81	0.00	20.74	185.55
LDEC001030-RA	protein disulfide-isomerase a3	1453.09	0.00	178.76	1631.85
LDEC000908-RA	ka261_mesma ame: full=potassium channel blocker alpha-ktx ame: full=neurotoxin 86 flags: precursor	301.35	0.00	36.95	338.30
LDEC000238-RA	serine protease	1042.57	0.00	126.03	1168.59
LDEC009737-RA	juvenile hormone binding protein partial	85.43	0.00	10.26	95.70
LDEC013013-RA	venom metalloproteinase 3 isoform x2	49.70	0.00	5.95	55.65
LDEC001398-RA	sortilin-related receptor-like	110.34	0.00	13.17	123.50
LDEC000706-RA	glucose dehydrogenase	90.82	0.00	10.69	101.50
LDEC002059-RA	digestive cysteine proteinase intestain	13466.07	0.00	1565.93	15032.00
LDEC008876-RA	antichymotrypsin-2-like isoform x3	53.55	0.00	6.16	59.71
LDEC009230-RA	polyadenylate-binding protein 1-like isoform 3	50.59	0.00	5.80	56.40
LDEC012058-RA	udp-glucuronosyltransferase 2b20-like	102.63	0.00	11.68	114.30
LDEC018548-RA	hexosaminidase 1 isoform x1	89.76	0.00	10.19	99.95
LDEC018931-RA	antichymotrypsin-2-like isoform x4	681.86	0.00	77.15	759.01
LDEC001143-RA	protein takeout-like	149.98	0.00	16.85	166.83

LDEC019636-RA	mesencephalic astrocyte-derived neurotrophic factor homolog	279.94	0.00	31.14	311.09
LDEC012233-RA	neo-calmodulin-like isoform x2	35.31	0.00	3.86	39.17
LDEC004210-RA	protein disulfide-isomerase a6	418.34	0.00	45.51	463.85
LDEC005903-RA	fatty acyl- reductase cg5065-like	167.08	0.00	18.08	185.17
LDEC003331-RA	l-lactate dehydrogenase isoform x2	645.56	0.00	68.52	714.07
LDEC020428-RA	prostatic acid phosphatase-like	299.87	0.00	31.78	331.65
LDEC005371-RA	serine protease h164	597.37	0.00	62.61	659.98
LDEC020338-RA	aldo-keto reductase family 4 member c9-like	81.53	0.00	8.49	90.03
LDEC005713-RA	93 kda serpin	130.05	0.00	13.38	143.43
LDEC004852-RA	inorganic phosphate cotransporter	33.90	0.00	3.47	37.37
LDEC002055-RA	digestive cysteine protease intestain	1217.65	0.00	120.65	1338.29
LDEC009663-RA	heat shock protein 70	183.25	0.00	17.98	201.23
LDEC002232-RA	glucose dehydrogenase	128.49	0.00	12.60	141.09
LDEC005691-RA	cathepsin l-like proteinase	604.19	0.00	57.76	661.95
LDEC009738-RA	juvenile hormone binding protein partial	50.18	0.00	4.74	54.92
LDEC007042-RA	cathepsin l	243.31	0.00	22.93	266.24
LDEC003840-RA	isoform c	65.06	0.00	6.09	71.15
LDEC018247-RA	probable c-5 sterol desaturase	47.02	0.00	4.32	51.34
LDEC022926-RA	b1 protein	186.74	0.00	16.92	203.66
LDEC014146-RA	z9 acyl- desaturase b	618.26	0.00	55.46	673.72
LDEC015748-RA	chymotrypsin inhibitor-like	228.41	0.00	20.21	248.62
LDEC003725-RA	pathogenesis-related protein 5	202.08	0.00	17.73	219.81
LDEC010285-RA	fatty acid synthase	1304.89	0.00	109.71	1414.60
LDEC013653-RA	alanine--glyoxylate aminotransferase 2-like	441.49	0.00	36.81	478.30
LDEC010015-RA	counting factor associated protein d-like	581.04	0.00	47.92	628.95
LDEC003222-RA	c-type lectin 5	590.46	0.00	48.38	638.84
LDEC007249-RA	agap006502-pa-like protein	34.17	0.00	2.76	36.93
LDEC021796-RA	gamma-interferon-inducible lysosomal thiol reductase-like	1300.11	0.00	104.54	1404.65
LDEC016562-RA	---NA---	1648.25	0.00	132.47	1780.72
LDEC020810-RA	esterase	276.49	0.00	22.12	298.61
LDEC006944-RA	---NA---	205.26	0.00	16.10	221.37
LDEC004176-RA	PREDICTED: uncharacterized protein LOC103312569	77.31	0.00	5.66	82.97
LDEC002056-RA	digestive cysteine protease intestain	36.07	0.00	2.62	38.69
LDEC004093-RA	coleoptericin a	225.88	0.00	16.35	242.23
LDEC017746-RA	tetratricopeptide repeat protein 39b-like	67.04	0.00	4.74	71.78
LDEC000931-RA	lipase 3	31.47	0.00	2.12	33.59
LDEC020736-RA	40s ribosomal protein s2	36.81	0.00	2.48	39.28
LDEC012287-RA	pathogenesis-related protein 5	48.02	0.00	3.22	51.24
LDEC009269-RA	venom acid phosphatase acph-1	141.57	0.00	9.45	151.02
LDEC007043-RA	cathepsin l	125.40	0.00	8.14	133.54
LDEC020340-RA	aldo-keto reductase	420.96	0.00	27.18	448.14
LDEC022268-RA	probable multidrug resistance-associated protein lethal 03659 isoform x1	74.32	0.00	4.67	78.99

LDEC018796-RA	vanin-like protein 1	30.43	0.00	1.88	32.31
LDEC018417-RA	odorant binding protein 8	225.77	0.00	13.84	239.61
LDEC002547-RA	---NA---	115.86	0.00	7.01	122.87
LDEC014788-RA	endo-beta- -glucanase	82.90	0.00	4.88	87.78
LDEC005334-RA	leukocyte elastase inhibitor	33.66	0.00	1.95	35.60
LDEC006706-RA	lipase 3	116.12	0.00	6.62	122.74
LDEC000211-RA	glutathione s-transferase epsilon	124.98	0.00	6.94	131.92
LDEC013494-RA	protein takeout-like	2364.42	0.00	130.49	2494.91
LDEC015349-RA	ubiquitin-like protein partial	1474.21	0.00	79.63	1553.84
LDEC003431-RA	flexible cuticle protein 12-like	61.48	0.00	3.26	64.73
LDEC017176-RA	salivary c-type lectin	96.21	0.00	4.99	101.20
LDEC006705-RA	lipase 3	235.70	0.00	11.96	247.66
LDEC007347-RA	protein takeout	79.01	0.00	3.93	82.94
LDEC017590-RA	serine protease s1a-1	173.59	0.00	8.56	182.16
LDEC001339-RA	agap009769-pa-like protein	28.30	0.01	1.34	29.64
LDEC015973-RA	cuticle protein 1	88.00	0.00	4.18	92.17
LDEC022969-RA	eukaryotic translation initiation factor 3 subunit a	77.78	0.00	3.68	81.46
LDEC004576-RA	ecdysone-induced protein 78c-like	28.61	0.01	1.34	29.95
LDEC002959-RA	c-type lectin galactose-binding isoform-like	194.38	0.00	9.13	203.51
LDEC022836-RA	fatty acyl- reductase cg5065 isoform x2	59.62	0.00	2.65	62.28
LDEC003766-RA	gustatory receptor candidate 59	44.79	0.00	1.98	46.78
LDEC009006-RA	protein takeout	582.96	0.00	25.66	608.62
LDEC017805-RA	chorion peroxidase-like isoform x2	29.46	0.00	1.27	30.74
LDEC022883-RA	digestive cysteine protease intestain	648.47	0.00	27.57	676.04
LDEC012588-RA	AGAP006960-PA	32.34	0.00	1.27	33.61
LDEC006945-RA	ccr4-not transcription complex subunit partial	28.18	0.01	1.10	29.28
LDEC013203-RA	collagen alpha-1 chain	219.83	0.00	8.46	228.28
LDEC006653-RA	peptidoglycan-recognition protein s2	58.32	0.00	2.23	60.55
LDEC020824-RA	endonuclease and reverse transcriptase-like protein	127.51	0.00	4.67	132.18
LDEC017376-RA	PREDICTED: uncharacterized protein LOC100142362	29.68	0.00	1.06	30.74
LDEC019184-RA	mast cell protease 6	130.13	0.00	4.57	134.69
LDEC012816-RA	arylphorin-like hexameric storage protein	58.63	0.00	2.02	60.65
LDEC007343-RA	PREDICTED: LOW QUALITY PROTEIN: uncharacterized protein LOC101888582	912.29	0.00	30.90	943.18
LDEC001221-RA	glycosyl hydrolase	51.54	0.00	1.73	53.27
LDEC023414-RA	agap011197-pa-like protein	2013.56	0.00	67.38	2080.94
LDEC018962-RA	low quality protein: cell wall protein tir4-like	29.92	0.00	0.96	30.87
LDEC008295-RA	pheromone-binding protein 3-like	39.31	0.00	1.20	40.51
LDEC009115-RA	cathepsin l precursor	61.44	0.00	1.88	63.31
LDEC003389-RA	PREDICTED: uncharacterized protein LOC656585	66.58	0.00	2.02	68.60
LDEC007681-RA	PREDICTED: uncharacterized protein LOC103314280	78.74	0.00	2.37	81.12
LDEC002561-RA	chymotrypsin-c-like isoform x1	145.15	0.00	4.28	149.43
LDEC008679-RA	laccase 1 isoform x1	2291.67	0.00	66.50	2358.17

LDEC004031-RA	abp1_ripcl ame: full=probable antibacterial peptide polyprotein	148.52	0.00	4.28	152.80
LDEC000024-RA	hypothetical protein YQE_02064, partial [Dendroctonus ponderosae]	43.43	0.00	1.24	44.67
LDEC001704-RA	collagen alpha-1 chain-like	38.57	0.00	1.10	39.67
LDEC015348-RA	agap011225-pa-like protein	1537.33	0.00	42.01	1579.34
LDEC021795-RA	---NA---	334.26	0.00	9.10	343.35
LDEC004625-RA	lysozyme precursor	74.77	0.00	2.02	76.78
LDEC015606-RA	cystine knot toxin	68.17	0.00	1.77	69.94
LDEC006895-RA	cuticle protein cp5	350.91	0.00	8.81	359.72
LDEC004092-RA	coleoptericin a	155.73	0.00	3.89	159.62
LDEC005728-RA	attacin-like immune protein	903.62	0.00	22.30	925.92
LDEC000795-RA	pro-phenol oxidase subunit 2	313.19	0.00	7.54	320.72
LDEC009497-RA	hypothetical antimicrobial peptide	261.67	0.00	6.09	267.76
LDEC011289-RA	cuticle protein 65-like	48.92	0.00	1.10	50.01
LDEC013281-RA	odorant-binding protein 5	247.12	0.00	5.49	252.60
LDEC006942-RA	hypothetical protein EAI_07617	158.66	0.00	3.43	162.10
LDEC013186-RA	ejaculatory bulb-specific protein 3	2233.49	0.00	48.24	2281.73
LDEC005075-RA	flocculation protein flo11	48.26	0.00	1.03	49.29
LDEC009001-RA	hypothetical protein DAPPUDRAFT_105533	173.25	0.00	3.68	176.93
LDEC003963-RA	atp-binding cassette sub-family b member mitochondrial	584.33	0.00	12.28	596.61
LDEC010451-RA	gb12811-like partial	146.96	0.00	3.08	150.04
LDEC023464-RA	agap012703-pa-like protein	64.42	0.00	1.34	65.77
LDEC009341-RA	microtubule-associated protein futsch-like isoform x12	35.84	0.00	0.74	36.58
LDEC009736-RA	juvenile hormone binding protein partial	108.30	0.00	2.23	110.53
LDEC023183-RA	jerky protein homolog-like	68.31	0.00	1.38	69.69
LDEC005685-RA	cathepsin l	597.31	0.00	11.75	609.06
LDEC005338-RA	isoform a	36.14	0.00	0.71	36.85
LDEC002286-RA	peroxidasin homolog	151.57	0.00	2.90	154.47
LDEC005723-RA	attacin-like immune protein	169.26	0.00	3.22	172.48
LDEC012734-RA	digestive cysteine proteinase intestain	54.66	0.00	1.03	55.69
LDEC005388-RA	adult cuticle protein 1-like	147.16	0.00	2.73	149.89
LDEC003116-RA	esterase	95.40	0.00	1.73	97.14
LDEC014481-RA	serine protease	77.61	0.00	1.20	78.82
LDEC008130-RA	protein rmd5 homolog a isoform x2	29.95	0.00	0.46	30.41
LDEC012084-RA	prostatic acid phosphatase	170.84	0.00	2.58	173.42
LDEC023304-RA	zinc carboxypeptidase	40.48	0.00	0.60	41.08
LDEC015347-RA	prostaglandin e synthase 3	276.64	0.00	4.07	280.71
LDEC000704-RA	antifreeze protein maxi	49.73	0.00	0.71	50.44
LDEC018149-RA	collagen alpha-1 chain	68.08	0.00	0.92	69.00
LDEC001698-RA	collagen alpha-1 chain-like	115.37	0.00	1.52	116.89
LDEC005757-RA	diapause-associated transcript-2	84.04	0.00	1.06	85.10
LDEC009116-RA	cathepsin l	37.06	0.00	0.46	37.52
LDEC000781-RA	protein takeout-like	71.72	0.00	0.81	72.53

LDEC009913-RA	hypothetical protein L798_04546	40.82	0.00	0.46	41.28
LDEC012045-RA	encapsulation-relating protein	86.94	0.00	0.96	87.90
LDEC007327-RA	hypothetical protein D910_06903	39.17	0.00	0.42	39.59
LDEC006894-RA	cuticle protein cp5	252.63	0.00	2.62	255.25
LDEC022265-RA	lactase-phlorizin hydrolase	49.06	0.00	0.50	49.55
LDEC003449-RA	larval cuticle protein lcp-17-like	35.48	0.00	0.35	35.83
LDEC003432-RA	larval cuticle protein 8-like	44.20	0.00	0.42	44.63
LDEC009342-RA	hormone receptor in 46-like protein	49.31	0.00	0.46	49.77
LDEC004299-RA	solute carrier family 25 member 35	348.52	0.00	3.19	351.71
LDEC014197-RA	larval cuticle protein a2b-like	293.81	0.00	2.48	296.29
LDEC005268-RA	fibril-forming collagen alpha chain-like	661.84	0.00	5.56	667.39
LDEC016508-RA	skin secretory protein xp2-like	39.83	0.00	0.32	40.15
LDEC016697-RA	PREDICTED: uncharacterized protein LOC657400 isoform X1	54.80	0.00	0.42	55.23
LDEC008874-RA	cec2 protein	30.54	0.00	0.21	30.76
LDEC017994-RA	pupal cuticle protein 20	103.86	0.00	0.71	104.57
LDEC012341-RA	high molecular weight subunit dx5-like	156.28	0.00	1.06	157.34
LDEC007252-RA	agap006502-pa-like protein	114.12	0.00	0.74	114.86
LDEC005727-RA	attacin-like immune protein	1288.66	0.00	7.40	1296.06
LDEC003448-RA	larval cuticle protein lcp-17-like	193.50	0.00	1.10	194.60
LDEC002958-RA	lectin subunit alpha-like	93.88	0.00	0.53	94.41
LDEC012089-RA	prostatic acid phosphatase-like	333.63	0.00	1.77	335.40
LDEC008092-RA	serine protease	98.44	0.00	0.50	98.94
LDEC006672-RA	PREDICTED: uncharacterized protein LOC100142033	1661.90	0.00	8.21	1670.11
LDEC009863-RA	inorganic phosphate cotransporter	73.71	0.00	0.35	74.06
LDEC003889-RA	chitinase 10 precursor	167.28	0.00	0.78	168.06
LDEC012088-RA	prostatic acid phosphatase	343.29	0.00	1.31	344.60
LDEC014800-RA	endopolygalacturonase	26.50	0.01	0.00	26.50
LDEC008133-RA	prostatic acid phosphatase-like	167.95	0.00	0.57	168.52
LDEC003783-RA	proclotting enzyme	401.56	0.00	1.27	402.84
LDEC017993-RA	pupal cuticle protein 20	33.87	0.00	0.11	33.98
LDEC003996-RA	glycine-rich cell wall structural	5953.13	0.00	18.19	5971.33
LDEC005687-RA	digestive cysteine protease intestain	139.82	0.00	0.39	140.21
LDEC019613-RA	PREDICTED: uncharacterized protein LOC660742	41.28	0.00	0.11	41.39
LDEC013001-RA	cuticular protein	27.87	0.00	0.07	27.94
LDEC008132-RA	prostatic acid phosphatase	261.56	0.00	0.64	262.20
LDEC024197-RA	quaking related	43.74	0.00	0.11	43.84
LDEC014079-RA	antifreeze protein maxi	63.08	0.00	0.14	63.22
LDEC020732-RA	zinc finger protein 512b	47.34	0.00	0.11	47.45
LDEC009102-RA	cytochrome p450 6k1	642.39	0.00	1.42	643.80
LDEC007473-RA	cytochrome p450 307a1	67.39	0.00	0.14	67.53
LDEC023170-RA	cytosolic carboxypeptidase 6	965.96	0.00	2.02	967.98
LDEC011790-RA	ice-structuring glycoprotein	73.44	0.00	0.14	73.58

LDEC002268-RA	PREDICTED: uncharacterized protein LOC655532 isoform X2	178.18	0.00	0.32	178.50
LDEC001911-RA	larval cuticle protein lcp-30	58.85	0.00	0.00	58.85
LDEC013797-RA	cuticular protein cpr2	43.87	0.00	0.07	43.94
LDEC016584-RA	odorant binding partial	51.09	0.00	0.07	51.16
LDEC009910-RA	hypothetical protein L798_04546	87.22	0.00	0.11	87.32
LDEC013553-RA	larval pupal cuticle protein h1c	680.05	0.00	0.81	680.87
LDEC000705-RA	antifreeze protein maxi-like	75.60	0.00	0.07	75.67
LDEC003760-RA	apolipoprotein d-like	76.25	0.00	0.07	76.32
LDEC022542-RA	plasma membrane calcium-transporting atpase	122.31	0.00	0.00	122.31
LDEC011656-RA	larval cuticle protein 8-like	1116.58	0.00	0.88	1117.47
LDEC022497-RA	esterase	45.84	0.00	0.04	45.88
LDEC006048-RA	hypothetical protein IscW_ISCW024931	332.89	0.00	0.21	333.10
LDEC003447-RA	larval cuticle protein lcp-17-like	1680.11	0.00	0.85	1680.96
LDEC024457-RA	---NA---	74.20	0.00	0.04	74.24
LDEC003999-RA	gly-rich protein	81.46	0.00	0.04	81.50
LDEC014792-RA	---NA---	180.71	0.00	0.07	180.78
LDEC013799-RA	endocuticle structural glycoprotein bd-1-like	3030.71	0.00	1.13	3031.84
LDEC006900-RA	larval cuticle protein lcp-30	405.95	0.00	0.14	406.09
LDEC006045-RA	chorion protein s38	407.56	0.00	0.14	407.70
LDEC009004-RA	---NA---	298.21	0.00	0.00	298.21
LDEC011658-RA	flexible cuticle protein 12	4046.44	0.00	1.34	4047.79
LDEC006047-RA	---NA---	452.02	0.00	0.14	452.16
LDEC005725-RA	attacin-like immune protein	912.35	0.00	0.28	912.64
LDEC011288-RA	---NA---	1316.92	0.00	0.32	1317.24
LDEC006052-RA	hypothetical protein IscW_ISCW024931	823.37	0.00	0.18	823.55
LDEC013552-RA	larval pupal cuticle protein h1c	377.49	0.00	0.07	377.56
LDEC021944-RA	cuticle protein	2220.42	0.00	0.35	2220.77
LDEC023632-RA	adfb like protein	231.60	0.00	0.04	231.64
LDEC011657-RA	cuticular protein 27a	2614.07	0.00	0.35	2614.43
LDEC012842-RA	b1 protein	744.64	0.00	0.00	744.64
LDEC006050-RA	---NA---	1034.70	0.00	0.00	1034.70
LDEC022543-RA	---NA---	417.69	0.00	0.04	417.72
LDEC014795-RA	maltase 2-like	486.35	0.00	0.04	486.38
LDEC001912-RA	larval cuticle protein lcp-30	4434.76	0.00	0.11	4434.87
LDEC014547-RA	hypothetical protein TcasGA2_TC002836	17966.99	0.00	0.39	17967.38
LDEC014544-RA	---NA---	24931.89	0.00	0.07	24931.96

Table 9S. Transposable elements found in the genome of *Lepinotarsa decemlineata*.

Class	No. of elements	Length occupied	Percentage of sequence
**DNA Transposon**	214017	65814287	5.68%
**Crypton**	9036	2041448	0.18%
**Maverick**	7633	3543185	0.30%
**Merlin**	118	19518	0.00%
**P**	196	80876	0.01%
**PIF-Harbinger**	312	108920	0.01%
**PiggyBac**	1659	653738	0.06%
**TcMar-Fot1**	1242	477036	0.04%
**TcMar-Mariner**	71714	19958085	1.72%
**TcMar-Tc1**	74528	25433624	2.19%
**TcMar-Tigger**	535	97062	0.01%
**TcMar-m44**	31600	7175489	0.62%
**hAT-Charlie**	5773	2280352	0.20%
**hAT-Tip100**	4168	1356066	0.12%
**Unassigned**	5503	2588888	0.22%
**LINE**	390083	118755191	10.23%
**CR1**	12688	3948478	0.34%
**CR1-Zenon**	43947	15864600	1.37%
**Dong-R4**	22668	9277919	0.80%
**I**	2988	1078040	0.09%
**I-Nimb**	180	65650	0.01%
**Jockey**	13981	6953377	0.60%
**L2**	59917	18867997	1.62%
**LOA**	45947	15341550	1.32%
**Penelope**	167040	36080823	3.10%
**R1**	2505	1733373	0.15%
**RTE-BovB**	119	60060	0.01%
**Tad1**	215	71126	0.01%
**Unassigned**	17888	9412198	0.81%
**LTR**	12466	6764044	0.59%
**Copia**	1276	537363	0.05%
**Gypsy**	6363	3057954	0.26%
**Gypsy-Cigr**	1986	1809823	0.16%
**Pao**	2841	1358904	0.12%
**RC**	2664	944631	0.08%

**Helitron**	2664	944631	0.08%
**Total interspersed**	619230	192278153	16.55%
**Low complexity**	14018	679438	0.06%
**Satellites**	560	288317	0.02%
**Simple repeats**	83727	3531098	0.30%
**Total**	717535	196777006	16.93%

Table 10S. List of genes in *Leptinotarsa decemlineata* genome with transposable elements in flanking regions.

**Gene Annotation**
5-aminoimidazole-4-carboxamide ribonucleotide formyltransferase/IMP cyclohydrolase
69 copies of cytochromes p450
ADP ribosylation factor like 1
aquaporin%2C partial
argonaute 2a
aspartic protease 2
aspartic protease 3
ATP Synthase/ATPase
Auxiliary RISC-associated or regulatory factors
beta-mannosidase-like
Cadherin 23
Cadherin 96C
Cadherin-like protein
Cadherin-N
CaM Kinase
cAMP-dependent protein kinase C1
cathepsin B-like proteinase
cathepsin L-like 1
chitin binding-like
Chitin binding Peritrophin-A domain containing protein
Chymotrypsin
Clathrin hc
Cyclin-dependent kinase 5 homolog Partial
DEAD RNA helicase domain proteins
defensin-like 1
defensin-like 3
diapause-associated transcript-2
digestive cysteine proteinase
DnaJ and jiv90 domain containing protein
DnaJ and sec63 domain containing protein
DnaJ and TPR domain containing protein
DnaJ doamin containing protein
dsRNA Binding
easter-like
Egghead
eIF2B-gamma
eIF4AII
eIF4AIII
Epsin-like (rsd-3)
fatty acid-biding protein
forkhead box protein O isoform A%2C partial
forkhead box protein O isoform B%2C partial
GDP-fucose transmembrane transporter
GDP-L-fucose synthase
GDP-mannose 4%2C6-dehydratase
Glutathion S trasferase 2
Glycoside hydrolase family 1

Glycoside hydrolase family 16
Glycoside hydrolase family 28
Glycoside hydrolase family 45
Glycoside hydrolase family 48 protein 1
Glycoside hydrolase family 48 protein 2
Glycoside hydrolase family 48 protein 3
Glycosyltransferase family 49
Gustatory receptor 33
Histone deacetylase 3 like
IAP1
IAP2
IKK-beta
intestain A1
intestain B1
intestain C4_A
intestain D1
intestain D2
intestain D3
intestain E1
intestain E2
intestain E3
Isocitrate dehydrogenase
JHBP-like 1
JHBP-like 3
JHBP-like 4
KIF18A-like
Glycoside hydrolase family 28
Glycoside hydrolase family 1
Light, Lysosomal Transport
lipase-like
liquid facets (Epn-1)
loquacious
lysosomal thiol reductase IP30-1
lysosomal thiol reductase IP30-2
lysosomal thiol reductase IP30-3
MAPKK4 incomplete
methoprene tolerant
Multidrug resistance-associated protein
Multi drug resistance cassette transporter
Phosphatidylinositol 3-kinase
putative nuclease
raspartic protease 1
Ribosome-biogenesis C-terminal fragment
rintestain C6
RNA-Pol subunit RPABC2
scavenger receptor-C2 Parital (1/3)
scavenger receptor-CI
SDN-like (III)
serine protease
serine protease%2C proclotting-like 2 (partial)
serine protease chymotrypsin
serine protease-like
serine protease-like 1
serine protease-like 2
Serine/threonine p21-activated kinase (PAK) mbt like protein
Shrub/Sfn7 gene
Sid1-A
SID1-C
Signal recognition particle protein 72
Similar to Xenopus ADAM10
Superoxide Dismutase
TC016254 Homolg Partial (2/2)
trypsin-like
Trypsin-like serine protease
Tudor-SN
U1A snRNP, RNA processing module
Vasa Intronic Gene (VIG) Partial, Auxiliary RISC-associated or regulatory factor
Vitellogenin

Table 11S. Average pairwise genetic divergence (FST) estimated for Leptinotarsa decemlineata populations. Population codes: NJ- New Jersey lab strain, WIs- imidacloprid susceptible population from Arlington, Wisconsin, WIr- imidacloprid resistant population from Hancock, Wisconsin, MI- imidacloprid resistant population from Michigan, and EU- European samples combined from Italy and Russia.

	**WIs**	**WIr**	**MI**	**NJ**	**EU**
**WIs**	0	0.035	0.070	0.088	0.182
**WIr**		0	0.071	0.095	0.15
**MI**			0	0.089	0.164
**NJ**				0	0.179
**EU**					0

Table 12S. Demographic parameters estimated from the allele frequency spectrum for *Leptinotarsa decemlineata* populations.

	Arlington,Wisconsin	Michigan	Europe
**N projected**	20	20	300
**Constant size**			
Likelihood	-42243.39896	-14449.8006	-115.679928
θ_0	83011.02	39296.62	12.49805553
**Two epoch**			
Likelihood	-42243.39602	-14449.7986	-115.679928
θ_0	16376.89	21411.59167	3.272435331
N_1/N_0	5.069	1.835	3.819
T	151.25	44.69	286
**Bottle growth**			
Likelihood	-14644.782866	-66.733882	-106.798686
θ_0	12595.8	93956.8915	3.06883311
N_b/N_0	243.233	10.369	985.465
N_1/N_0	3.308	0.066	2.119
T	18.04	0.329	21
**Three epoch**			
Likelihood	-41795.10345	-15675.8233	-115.679928
θ_0	8246.73	4798.185014	11.66530265
N_b/N_0	21.516	10.518	16.737
N_1/N_0	10.012	7.74	1.071
τ_b	14.41	16	6.7
τ_g	26.41	325	398

θ_0: ancestral theta, or effective population size*mutation rate
N_1/N_0: Ratio of contemporary to ancient population size
N_b/N_0: Ratio of population size after instantanous change to ancient population size
τ: Time in the past at which instantaneous change happened in units of 2*Na generations
τ_b: Length of bottleneck in units of 2*Na generations
τ_g: Time since bottleneck recovery in units of 2*Na generations

Table 13S. The genes encoding odorant binding proteins (OBP) in *Leptinotarsa decemlineata*. The genomic locations (columns ’Scaffold’, ’Coordinates’ [start-end position of cDNA] and ’Strand’), number of introns and splicing phases, number of amino acids (‘AAs’), OBP classification, and correspondence to OBP sequences identified by Liu et al. [34] from antennal transcriptome of *L. decemlineata* are shown.

Gene name°	Scaffold	Coordinates	Strand	Introns|phases	AAs	OBP class	Number in Liu et al. (2015)	Transcript differences compared to genomic protein model
LdecOBP1	768	30534-35796	minus	1|0	134	Minus C	OBP15	3 amino acid substitutions
LdecOBP2	158	231811-233138	plus	1|0	143	Minus C	NA	
LdecOBP3	2165	27877-31057	plus	1|0	117	Minus C	NA	
LdecOBP4FIX	2165	42673-43020	plus	1|0	142	Minus C	NA	
LdecOBP5	5	1098601-1107706	minus	1|0	120	Minus C	OBP25	3 amino acid substitutions
LdecOBP6	5	1141751-1144600	minus	1|0	114	Minus C	NA	
LdecOBP7FIX	5	1172871-1179892	minus	1|0	117	Minus C	NA	
LdecOBP8	5	1189332-1195173	minus	1|0	146	Minus C	NA	
LdecOBP9	942	255047-257458	minus	1|0	117	Minus C	NA	
LdecOBP10PSE	942	320694-320996	plus	1|0	101	Minus C	NA	
LdecOBP11	942	331690-337793	minus	1|0	112	Minus C	NA	
LdecOBP12	506	73884-80443	plus	2|01	135	Minus C	NA	
LdecOBP13	506	82214-86962	plus	2|01	132	Minus C	OBP18	Identical
LdecOBP14	506	92067-93122	plus	1|0	135	Minus C	OBP14	1 amino acid substitution
LdecOBP15	506	101495-106511	plus	1|0	132	Minus C	OBP19	1 amino acid substitution
LdecOBP16	506	113523-117470	plus	1|0	139	Minus C	NA	
LdecOBP17	506	123762-128941	plus	3|000	256	Dimer	NA	
LdecOBP18FIX	4872	10168-10464	minus	1|0	130	Minus C	OBP21	3 amino acid substitutions
LdecOBP19	506	131055-134183	minus	1|0	133	Minus C	OBP17	2 amino acid substitutions
LdecOBP20	4052	10265-25636	plus	1|0	140	Minus C	NA	
LdecOBP21FIX	128	574213-580730	plus	1|0	140	Minus C	NA	
LdecOBP22	1299	57363-60569	minus	1|0	128	Classic	OBP22	Identical
LdecOBP23	1299	67333-74378	minus	1|0	125	Classic	OBP23	1 amino acid substitution
LdecOBP24	208	1957692-1961993	minus	1|0	114	Classic	NA	
LdecOBP25FIX	47	59788-60087	minus	1|0	122	Classic	OBP24	Identical
LdecOBP26FIX	116	551879-557535	minus	2|10	155	Classic	OBP26	Lacks N terminal half
LdecOBP27FIX	874	149244-1500033	minus	1|1	153	Classic	OBP16	”EN” before start ”M” and lacks C-terminal
LdecOBP28	1983	64852-96520	plus	5|01001	177	Classic	OBP4	2 amino acid substitutions and C terminal amino acid missing
LdecOBP29FIX	817	25093-35844	plus	3|100	255	Plus C	OBP1	5 amino acid substitutions
LdecOBP30	31	1270048-1278759	minus	5|01010	136	Classic	OBP13	2 amino acid substitutions
LdecOBP31	31	1280360-1290725	minus	5|01010	131	Classic	OBP20	Identical
LdecOBP32FIX	817	70671-80539	minus	6|010010	139	Classic	OBP12	Identical
LdecOBP33	57	1641209-1644774	plus	5|01000	142	Classic	OBP10	”MM” start and 1 amino acid substitution
LdecOBP34	44	2817409-2833602	plus	5|01010	159	Classic	OBP5	1 amino acid substitution
LdecOBP35FIX	913	152773-163008	minus	4|0101	248	Classic	OBP2	2 amino acid substitutions
LdecOBP36	946	103681-115474	plus	6|010010	143	Classic	OBP9	1 amino acid substitution
LdecOBP37	130	703036-712245	plus	5|00010	143	Minus C	OBP8	1 amino acid substitution
LdecOBP38CTE	1347	66819-80346	minus	>3|000	79	Minus C	NA	
LdecOBP39FIX	32	899856-917341	minus	5|00010	142	Minus C	OBP11	3 amino acid substitutions
LdecOBP40	101	70403-80371	minus	5|00010	139	Minus C	NA	
LdecOBP41	44	1402321-1410418	minus	5|00010	141	Minus C	NA	

<table>
<tr><th></th><th></th><th></th><th></th><th></th><th></th><th></th><th></th><th></th></tr>
<tr><td>LdecOBP42</td><td>44</td><td>1382816-1392434</td><td>minus</td><td>5|00010</td><td>142</td><td>Minus C</td><td>NA</td><td></td></tr>
<tr><td>LdecOBP43</td><td>44</td><td>1367643-1375622</td><td>minus</td><td>5|00010</td><td>141</td><td>Minus C</td><td>NA</td><td></td></tr>
<tr><td>LdecOBP44FJ</td><td>3735/855</td><td>30806-30934/129968-134028</td><td>minus/minus</td><td>>4|0001?</td><td>141</td><td>Minus C</td><td>NA</td><td></td></tr>
<tr><td>LdecOBP45</td><td>160</td><td>528844-531777</td><td>minus</td><td>5|00010</td><td>144</td><td>Minus C</td><td>OBP7</td><td>Identical</td></tr>
<tr><td>LdecOBP46</td><td>160</td><td>514046-521438</td><td>minus</td><td>5|00010</td><td>144</td><td>Minus C</td><td>NA</td><td></td></tr>
<tr><td>LdecOBP47</td><td>160</td><td>499716-508498</td><td>minus</td><td>5|00010</td><td>143</td><td>Minus C</td><td>NA</td><td></td></tr>
<tr><td>LdecOBP48</td><td>160</td><td>378210-388841</td><td>plus</td><td>5|00010</td><td>145</td><td>Minus C</td><td>NA</td><td></td></tr>
<tr><td>LdecOBP49</td><td>160</td><td>395662-406820</td><td>plus</td><td>5|00010</td><td>143</td><td>Minus C</td><td>NA</td><td></td></tr>
<tr><td>LdecOBP50FIX</td><td>160</td><td>416936-423233</td><td>plus</td><td>>3|??010</td><td>147</td><td>Minus C</td><td>NA</td><td></td></tr>
<tr><td>LdecOBP51FIX</td><td>160</td><td>434479-441351</td><td>plus</td><td>5|00010</td><td>146</td><td>Minus C</td><td>NA</td><td></td></tr>
<tr><td>LdecOBP52</td><td>160</td><td>447688-455945</td><td>plus</td><td>5|00010</td><td>142</td><td>Minus C</td><td>NA</td><td></td></tr>
<tr><td>LdecOBP53FIX</td><td>160</td><td>462998-475040</td><td>plus</td><td>5|00010</td><td>145</td><td>Minus C</td><td>NA</td><td></td></tr>
<tr><td>LdecOBP54</td><td>160</td><td>483402-489922</td><td>plus</td><td>5|00010</td><td>145</td><td>Minus C</td><td>NA</td><td></td></tr>
<tr><td>LdecOBP55FJ</td><td>1347/1506</td><td>88574-94882/111453-111529</td><td>plus/minus</td><td>5|00010</td><td>149</td><td>Minus C</td><td>OBP3</td><td>N-terminal wrong and 1 amino acid substitution</td></tr>
<tr><td>LdecOBP56FJ</td><td>1347/1506</td><td>116718-119402/88643-88786</td><td>plus/minus</td><td>3|010</td><td>149</td><td>Minus C</td><td>OBP6</td><td>2 amino acid substitutions</td></tr>
<tr><td>LdecOBP57</td><td>811</td><td>220020-224657</td><td>minus</td><td>5|00010</td><td>144</td><td>Minus C</td><td>NA</td><td></td></tr>
<tr><td>LdecOBP58</td><td>811</td><td>227939-229849</td><td>minus</td><td>5|00010</td><td>142</td><td>Minus C</td><td>NA</td><td></td></tr>
<tr><td>LdecOBP59</td><td>811</td><td>235404-237298</td><td>minus</td><td>5|00010</td><td>142</td><td>Minus C</td><td>NA</td><td></td></tr>
</table>

° *Suffixes to gene names:* CTE = C-terminal missing; JOI = exons from two scaffolds joined into one gene model; FIX = model completed manually using raw reads; PSE = pseudogene; 1-letter abbreviations for genes with multiple suffixes: FJ = FIX + JOI.

Table 14S. Details of the genes encoding odorant receptors (ORs) in *Leptinotarsa decemlineata*. Included are the genomic locations ('Scaffold'), number of amino acids ('AAs'), correspondence to OR sequences identified by Liu et al. [34] from an antennal transcriptome of *L. decemlineata*, and notes concerning the structure of pseudogenes and incomplete genes.

Gene name	Scaffold	AAs	Number in Liu et al. (2015)	Notes
Ldec\Orco	1110	479	NA	Complete
LdecOR1	1126	405	NA	Complete
LdecOR2PSE	3952	106	NA	3 fragments near N-terminus
LdecOR3CTE	1126	397	NA	Missing few AAs near C-terminus
LdecOR4PSE	1126	179	NA	2 fragments spanning first exon of Ldec3
LdecOR5IC	821	331	NA	Missing small second and terminal exons
LdecOR6IC	870	331	NA	Missing small second and terminal exons
LdecOR7	1462	406	33	Complete
LdecOR8CTE	1462	198	NA	Missing 50% of gene, estimated five terminal exons
LdecOR9	181	390	26	Complete
LdecOR10	561	396	NA	Complete
LdecOR11	561	395	6	Complete
LdecOR12NI	65	205	NA	Missing half of first exon, and penultimate exon
LdecOR13	65	371	NA	Complete
LdecOR14	1467	369	NA	Complete
LdecOR15NTE	650	384	NA	Missing small first exon
LdecOR16NTE	82	383	24, 36	Missing small first exon
LdecOR17NTE	51	388	NA	Missing small first exon
LdecOR18NC	58	371	NA	Missing small first and terminal exons
LdecOR19	237	417	8	Complete
LdecOR20IC	150	327	2	Missing third exon and terminal exon
LdecOR21	618	422	7, 11	Complete
LdecOR22CTE	120	403	NA	Missing small terminal exon
LdecOR23FIX	608	417	3	Missing fragment of exon 3, and exon 4; fixed by Liu et al. 2015
LdecOR24FIX	618, 4138	423	NA	Missing small fragment of exon 6; fixed by Liu et al. 2015
LdecOR25	136, 3465	404	NA	Complete
LdecOR26	821	403	NA	Complete
LdecOR27	173	397	NA	Complete
LdecOR28PSE	341	356	NA	Stop codon in penultimate exon, no terminal exon
LdecOR29NI	1097	206	29	Missing fragment of exon 1, and exons 2 and 4
LdecOR30	1260	390	35	Complete
LdecOR31NC	1063	175	NA	Missing >50% of gene; only includes three middle exons
LdecOR32NTE	670	329	4	Missing first exon
LdecOR33NTE	274	323	NA	Missing first exon
LdecOR34	1368	384	1	Complete
LdecOR35	1368	384	NA	Complete
LdecOR36CTE	325	287	NA	Missing final three exons

LdecOR37NTE	1267	326	NA	Missing first exon
LdecOR38INT	1554, 1171	331	20, 32	Missing third exon
LdecOR39	12	385	34	Complete
LdecOR40	302	386	9, 22	Complete
LdecOR41	302	387	12, 31	Complete
LdecOR42CTE	334	343	NA	Missing half of penultimate exon and small terminal exon
LdecOR43	59, 5353, 16827	406	13, 25	Complete
LdecOR44	334	390	17, 19	Complete
LdecOR45	52	347	16, 30	Complete
LdecOR46	21	373	5	Complete
LdecOR47	2571	381	14, 23	Complete
LdecOR48INT	2571	214	NA	Missing second exon
LdecOR49	52	381	NA	Complete
LdecOR50NTE	321	376	NA	Missing small first exon
LdecOR51	1233	372	21	Complete
LdecOR52PSE	1370	130	NA	Two fragments homologous to second exon of OR53
LdecOR53NTE	34	286	NA	Missing first exon
LdecOR54FIX	693	286	15, 18	Missing fragment of exon 2 and terminal exon; fixed by Liu et al. 2015
LdecOR55NTE	34	288	NA	Missing first exon
LdecOR56NTE	96	321	10	Missing first two exons
LdecOR57	277	402	NA	Complete
LdecOR58NTE	1284	307	NA	Missing first two exons
LdecOR59NTE	2	307	NA	Missing first two exons
LdecOR60NTE	894	310	NA	Missing first two exons
LdecOR61NTE	894	311	NA	Missing first two exons
LdecOR62NC	1059	290	NA	Missing first two exons and terminal two exons
LdecOR63NIC	894	253	NA	Missing first two exons, exon 5, and terminal exon
LdecOR64CTE	425	355	NA	Missing small terminal exon
LdecOR65NI	425	239	NA	Missing first two exons and exons 4 and 5
LdecOR66NI	425	121	NA	Missing >50% of gene; fragment of third exon and two terminal exons
LdecOR67NTE	621	364	NA	Missing small first exon
LdecOR68IC	276	220	NA	Missing first two exons, exons 4 and 5, and terminal exon
LdecOR69NI	276	269	NA	Missing first two exons and exon 4
LdecOR70NTE	415	305	NA	Missing first two exons
LdecOR71	64	385	NA	Complete
LdecOR72INT	64	181	NA	Missing >50% of gene; missing large third exon and exon 5
LdecOR73	64	384	NA	Complete
LdecOR74	64	376	NA	Complete
LdecOR75NI	64	331	NA	Missing small first exon and exon 5
LdecOR76NTE	58	371	NA	Missing small first exon
LdecOR77INT	1640	310	NA	Missing exons 5 and 6
LdecOR78NC	1391	276	27	Missing first exon (possibly two) and terminal exon
LdecOR79INT	732	453	NA	Missing 3-4 AAs of Exon 4

Table 15S. Details on the *L. decemlineata* gustatory receptors (GR) gene family. Columns include the gene name (suffixes are INT = internal sequence missing, NTE = N terminus missing, CTE = C terminus missing, JOI = join models across scaffold, PSE = pseudogene), genomic locations, number of introns and splicing phases, number of amino acids ('AAs') and annotation comments.

No.	Gene name	Suffix	Scaffold	Coordinates	Strand	Introns|phases	AAs	Comments
1	LdecGR1		65	616054-647521	plus	5|00110	443	Replace LdecTmpM006445-RA
2	LdecGR2	JOI	643/10963/ 643	51356-61122/2712-3160/32595-32921	minus	N/A	394	Gene model has 3 parts - merging across scaffolds, part1 (replace LdecTmpM014761-RA) part2 (replace LdecTmpS024642-RA) and part3, part1and3 scaffold643, part2 scaffold10963
3	LdecGR3	INT	489	442492-458677	plus	N/A	348	Gene model has 2 parts - missing internal sequence, Assembly gap
4	LdecGR4	INT	1635	21651-23523/20230-20361/ 12166-12249	minus	N/A	271	Gene model has 3 parts - missing internal sequence, Assembly gap
5	LdecGR5		1635	53376-60712	plus	7|1100200	411	Replace LdecTmpM022080-RA (add 2 exons at 3' end)
6	LdecGR6		1635	79770-87417	plus	7|1000200	387	Replace LdecTmpM022081-RA (add 2 internal exons, extend first exon)
7	LdecGR7	INT	1635	89347-90302/91350-91499/96033-97978	plus	N/A	249	Gene model has 3 parts - missing internal sequence, Assembly gap
8	LdecGR8	NTE	330	36262-42857	plus	5|10000	333	New gene model, partial gene, missing N terminus
9	LdecGR9	IC	330	19783-20004/23283-23824	plus	N/A	231	Gene model has 2 parts - missing internal and C terminus sequence, Assembly gap
10	LdecGR10	NIC	8	1600488-1616108/1585523-1588602	minus	N/A	319	New gene model (part 1 and 2), Partial gene - N, C and Internal seq missing
11	LdecGR11	INT	77	155820-156698/150953-152338	minus	N/A	364	New gene model (Part 1 and 2), Partial gene - Internal seq missing, assembly gap
12	LdecGR12		278	581444-583563	minus	1|0	379	New gene model
13	LdecGR13	CTE	2114	43701-59954	plus	1|0	292	New gene model, Partial gene, missing C terminus - Assembly gap
14	LdecGR14	NTE	2114	66122-72255	plus	1|0	347	New gene model, Partial gene, missing N terminus - Assembly gap
15	LdecGR15		980	29138-36168	plus	1|0	351	Replace LdecTmpM018846-RA
16	LdecGR16	NI	2890	31026-31186/26464-26856/18489-18545	minus	N/A	202	New gene model (part 1, 2 and 3), Partial gene - N and Internal seq missing
17	LdecGR17		154	46389-53506	plus	2|20	375	New gene model
18	LdecGR18		154	57960-67571	plus	2|20	385	New gene model
19	LdecGR19		17	479900-484661	minus	1|0	379	Modify old gene model (LdecTmpA003558-RA) (split exon1 and exon2)
20	LdecGR20a	INT	61	558058-558849/564772-565041	plus	1|0	353	New gene model, alternative splicing, Internal sequence missing (part 1 and 2), assembly gap at 3' end of 1st exon
21	LdecGR20b		61	561245-565041	plus	1|0	364	New gene model, alternative splicing
22	LdecGR21		557	816868-820770	plus	1|0	381	Replace LdecTmpS008149-RA
23	LdecGR22	CTE	3631	15577-16455	minus	0	293	New gene model, Partial gene - C terminus missing, End of scaffold
24	LdecGR23		3631	27792-32644	minus	1|0	377	New gene model
25	LdecGR24		3631	37815-46259	minus	1|0	372	New gene model

26	LdecGR25	INT	272	482687-488461	minus	N/A	289	New gene model, Partial gene - internal sequence missing (model contain 2 parts), Assembly gap
27	LdecGR26	CTE	272	503400-504194	minus	0	265	New gene model, Partial gene - C terminus missing, Assembly gap
28	LdecGR27		717	170906-187445	minus	1|0	369	New gene model
29	LdecGR28	CTE	717	193095-193898	minus	0	268	New gene model, Partial gene - C terminus missing, Assembly gap
30	LdecGR29	CTE	1166	38437-60819	minus	1|0	346	New gene model, partial gene model, C terminus missing, assembly gap
31	LdecGR30		754	50102-59991	plus	1|0	384	New gene model
32	LdecGR31		1166	6890-17503	minus	1|0	358	New gene model
33	LdecGR32		1673	319495-349259	minus	1|0	362	New gene model
34	LdecGR33		1673	281546-293623	minus	1|0	363	New gene model
35	LdecGR34		347	187770-193865	plus	1|0	372	New gene model
36	LdecGR35		33	1205093-1212481	plus	1|0	405	New gene model
37	LdecGR36	CTE	29	1989528-1990439	minus	0	304	New gene model, Partial gene -C terminus missing
38	LdecGR37	CTE	29	1997069-1997974	minus	0	302	New gene model, Partial gene -C terminus missing
39	LdecGR38		29	2003083-2006661	minus	1|0	409	Replace LdecTmpM003032-RA
40	LdecGR39	CTE	29	2016773-2017675	minus	0	301	New gene model, Partial gene - C terminus missing
41	LdecGR40		29	2027309-2034614	minus	1|0	402	New gene model
42	LdecGR41a		334	255167-266245	plus	1|0	370	New gene model, alternative splicing
43	LdecGR41b		334	262284-266245	plus	1|0	367	New gene model, alternative splicing
44	LdecGR42		334	270303-279198	plus	1|0	371	New gene model
45	LdecGR43		1263	123075-129898	plus	1|0	374	Replace LdecTmpS019980-RA
46	LdecGR44		1263	139586-141462	plus	1|0	374	New gene model
47	LdecGR45		1263	147984-150183	plus	1|0	351	Replace LdecTmpS019980-RA
48	LdecGR46		1263	157413-166310	plus	1|0	345	Replace LdecTmpS019980-RA
49	LdecGR47	PSE	1263	170864-171588	plus	0	241	Pseudogene, stop codon in first exon, 2nd exon missing
50	LdecGR48a		122	729231-810264	minus	1|0	356	Replace LdecTmpS007797-RA, alternative splicing
51	LdecGR48b		122	729231-805628	minus	1|0	359	Replace LdecTmpS007797-RA, alternative splicing
52	LdecGR48c		122	729231-798094	minus	1|0	359	Replace LdecTmpS007797-RA, alternative splicing
53	LdecGR48d		122	729231-792138	minus	1|0	378	Replace LdecTmpS007797-RA, alternative splicing
54	LdecGR48e		122	729231-785022	minus	1|0	373	Replace LdecTmpS007797-RA, alternative splicing
55	LdecGR48f		122	729231-778588	minus	1|0	376	Replace LdecTmpS007797-RA, alternative splicing

56	LdecGR48g		122	729231-768849	minus	1|0	373	Replace LdecTmpS007797-RA, alternative splicing
57	LdecGR48h		122	729231-761642	minus	1|0	373	Replace LdecTmpS007797-RA, alternative splicing
58	LdecGR48i		122	729231-754429	minus	1|0	368	Replace LdecTmpS007797-RA, alternative splicing
59	LdecGR48j		122	729231-750998	minus	1|0	376	Replace LdecTmpS007797-RA, alternative splicing
60	LdecGR48k		122	729231-744869	minus	1|0	376	Replace LdecTmpS007797-RA, alternative splicing
61	LdecGR48l		122	729231-737276	minus	1|0	367	Replace LdecTmpS007797-RA, alternative splicing
62	LdecGR48m		122	729231-733454	minus	1|0	367	Replace LdecTmpS007797-RA, alternative splicing
63	LdecGR49		474	453364-459933	plus	1|0	369	New gene model
64	LdecGR50a	CTE	109	230155-231003	plus	0	283	Partial gene, missing C terminus (2nd exon) - Assembly gap, alternative splicing
65	LdecGR50b	CTE	109	235997-236854	plus	0	286	Partial gene, missing C terminus (2nd exon) - Assembly gap, alternative splicing
66	LdecGR50c	NC	109	242812-243603	plus	0	264	Partial gene, missing C terminus (2nd exon) - Assembly gap, alternative splicing
67	LdecGR50d	CTE	109	248589-249428	plus	0	280	Partial gene, missing C terminus (2nd exon) - Assembly gap, alternative splicing
68	LdecGR50e	CTE	109	257726-258568	plus	0	281	Partial gene, missing C terminus (2nd exon) - Assembly gap, alternative splicing
69	LdecGR50f	CTE	109	264444-265280	plus	0	279	Partial gene, missing C terminus (2nd exon) - Assembly gap, alternative splicing
70	LdecGR50g	CTE	109	271861-272694	plus	0	278	Partial gene, missing C terminus (2nd exon) - Assembly gap, alternative splicing
71	LdecGR51a		208	264127-289151	plus	1|0	362	New gene model, alternative splicing
72	LdecGR51b		208	272381-289151	plus	1|0	362	New gene model, alternative splicing
73	LdecGR51c		208	283303-289151	plus	1|0	356	New gene model, alternative splicing
74	LdecGR52a		751	111283-139204	plus	1|0	370	Replace LdecTmpS017030-RA, alternative splicing
75	LdecGR52b		751	120322-139204	plus	1|0	371	Replace LdecTmpS017030-RA, alternative splicing
76	LdecGR53a		40	1748771-1819677	minus	1|0	360	New gene model, alternative splicing
77	LdecGR53b		40	1748771-1808790	minus	1|0	358	New gene model, alternative splicing
78	LdecGR54a	INT	75	1156671-1157531/1138585-1138845	minus	1|0	373	New gene model, alternative splicing, partial gene missing internal sequence - assembly gap at 3' end of 1st exon, model has 2 parts
79	LdecGR54b		75	1138585-1152847	minus	1|0	375	New gene model, alternative splicing
80	LdecGR54c		75	1138585-1148819	minus	1|0	369	New gene model, alternative splicing
81	LdecGR54d		75	1138585-1143561	minus	1|0	380	New gene model, alternative splicing
82	LdecGR55		217	730526-734697	plus	1|0	377	New gene model
83	LdecGR56a	PSE	244	568373-582747	plus	N/A	376	New gene model, alternative splicing, Pseudogene
84	LdecGR56b		244	575427-582747	plus	1|0	373	New gene model, alternative splicing
85	LdecGR56c		244	580190-582747	plus	1|0	376	New gene model, alternative splicing

86	LdecGR57		1524	178941-199752	minus	1|0	375	New gene model
87	LdecGR58		1135	20763-26120	minus	1|0	376	Replace LdecTmpS020356-RA
88	LdecGR59		88	939560-944820	plus	1|0	377	New gene model
89	LdecGR60		10	1505521-1508518	plus	1|0	375	New gene model
90	LdecGR61	JOI	10//6674	1250603-1251436//8331-8591	minus/plus	N/A	376	Replace LdecTmpS002504-RA,merging two gene models across scaffolds (Part 1 - Scaffold 10, Part 2 - Scaffold 6674)
91	LdecGR62		336	338550-348303	minus	1|0	370	New gene model
92	LdecGR63		19	2103384-2106008	minus	1|0	364	New gene model
93	LdecGR64a		1034	31381-49514	plus	1|0	393	Replace LdecTmpS018864-RA, alternative splicing
94	LdecGR64b		1034	35010-49514	plus	1|0	393	Replace LdecTmpS018864-RA, alternative splicing
95	LdecGR64c		1034	40146-49514	plus	1|0	392	Replace LdecTmpS018864-RA, alternative splicing
96	LdecGR65a	CTE	1034	57545-95429	plus	1|0	382	Partial gene, missing C terminus - Assembly gap, alternative splicing
97	LdecGR65b	CTE	1034	68508-95429	plus	1|0	381	Partial gene - missing C terminus - Assembly gap, alternative splicing
98	LdecGR65c	NC	1034	81532-95429	plus	1|0	239	Partial gene - missing N and C terminus - Assembly gap, alternative splicing
99	LdecGR66		1034	101458-106891	plus	1|0	382	New gene model
100	LdecGR67a		1034	126779-197718	plus	1|0	377	New gene model, Alternative splicing
101	LdecGR67b		1034	139198-197718	plus	1|0	378	New gene model, Alternative splicing
102	LdecGR67c		1034	141315-197718	plus	1|0	377	New gene model, Alternative splicing
103	LdecGR67d		1034	163919-197718	plus	1|0	365	New gene model, Alternative splicing
104	LdecGR67e		1034	184250-197718	plus	1|0	374	New gene model, Alternative splicing
105	LdecGR68	INT	5699	25834-26388/11522-11806	minus	1|0	279	Internal sequence missing, Assembly gap
106	LdecGR69	CTE	3705	9216-10103	plus	0	296	Partial gene, missing C terminus (2nd exon), Assembly gap
107	LdecGR70a		820	134377-145279	plus	1|0	399	New gene model, Alternative splicing
108	LdecGR70b		820	138420-145279	plus	1|0	399	New gene model, Alternative splicing
109	LdecGR71		820	157491-170094	plus	1|0	397	New gene model
110	LdecGR72		773	86846-94951	minus	1|0	391	Replace LdecTmpM016780-RA
111	LdecGR73a	NTE	328	449754-461447	minus	1|0	385	New gene model, Alternative splicing, Partial model N terminus missing - Assembly gap
112	LdecGR73b		328	449754-457184	minus	1|0	395	New gene model, Alternative splicing
113	LdecGR74		689	329894-333293	minus	1|0	402	Replace LdecTmpM016031-RA
114	LdecGR75	CTE	456	43204-44031	minus	0	276	Partial gene model, C terminus missing (2nd exon), Assembly gap
115	LdecGR76		423	250930-275649	plus	0	364	New gene model

116	LdecGR77	NTE	336	352358-363072	minus	1|0	265	Partial gene model, N terminus missing, Assembly gap
117	LdecGR78		239	501157-509796	minus	1|0	400	New gene model
118	LdecGR79		18	1120018-1127838	plus	1|0	375	New gene model
119	LdecGR80a	INT	112	832032-832889/823281-824135	minus	N/A	328	New gene model (Part 1 and 2), Partial gene - Internal seq missing, 5' terminus of 2nd exon missing, assembly gap
120	LdecGR80b	INT	112	827316-828182/823281-824135	minus	N/A	331	New gene model (Part 1 and 2), Partial gene - Internal seq missing, 5' terminus of 2nd exon missing, assembly gap
121	LdecGR80c	INT	112	823281-824135/823281-824135	minus	N/A	327	New gene model (Part 1 and 2), Partial gene - Internal seq missing, 5' terminus of 2nd exon missing, assembly gap
122	LdecGR81		112	837105-838516	minus	1|0	380	New gene model
123	LdecGR82	CTE	4662	30529-31380	plus	0	284	New gene model, Partial gene, missing C terminus - Assembly gap
124	LdecGR83a	NC	349	150681-151301	minus	0	207	New gene model, Partial gene - missing C terminus (2nd exon) and N terminus, alternative splicing
125	LdecGR83b	CTE	349	149326-150198	minus	0	291	New gene model, Partial gene - missing C terminus (2nd exon), alternative splicing
126	LdecGR84a	NTE	1636	59258-115256	plus	3|000	245	New gene model, Alternative splicing, Assembly gaps
127	LdecGR84b	INT	1636	69800-111526	plus	N/A	211	New gene model, Alternative splicing, Assembly gaps (2 parts)
128	LdecGR84c		1636	83967-111526	plus	3|000	386	New gene model, Alternative splicing
129	LdecGR84d		1636	88866-111526	plus	3|000	396	New gene model, Alternative splicing
130	LdecGR84e		1636	92528-111526	plus	3|000	394	New gene model, Alternative splicing
131	LdecGR84f	PSE	1636	98078-111526	plus	N/A	235	Pseudogene, truncation at the N terminus
132	LdecGR84g		1636	103506-111526	plus	3|000	394	New gene model, Alternative splicing
133	LdecGR85		1636	119409-132448	plus	3|000	393	New gene model
134	LdecGR86		1636	143163-154539	plus	2|00	428	New gene model
135	LdecGR87		1086	78645-93447	minus	3|000	398	Replace LdecTmpM018818-RA
136	LdecGR88a		1086	111275-176195	minus	2|00	385	Replace LdecTmpS018819-RA, alternative splicing
137	LdecGR88b		1086	111275-168167	minus	2|00	386	Replace LdecTmpS018819-RA, alternative splicing
138	LdecGR88c		1086	111275-158733	minus	2|00	379	Replace LdecTmpS018819-RA, alternative splicing
139	LdecGR88d	NTE	1086	111275-120572	minus	2|00	425	Replace LdecTmpS018819-RA, alternative splicing
140	LdecGR89a		211	464159-495078	plus	3|000	392	New gene model, Alternative splicing
141	LdecGR89b	INT	211	474356-475005/491025-495078	plus	N/A	290	New gene model, Alternative splicing, Missing internal sequence (3' end of 1st exon - assembly gap), Models has 2 parts (part 1 and 2)
142	LdecGR89c		211	487188-495078	plus	3|000	395	New gene model, Alternative splicing
143	LdecGR90		91	509692-512308	plus	1|0	375	New gene model
144	LdecGR91a		904	221166-228880	plus	1|0	367	New gene model, alternative splicing

145	LdecGR91b		904	221166-231917	plus	1|0	367	New gene model, alternative splicing
146	LdecGR92	JOI	1167//569	190718-191551/17351-17617	plus	1|0	366	New gene model, result of merging models across scaffolds, part 1 scaffold1167 + part2 scaffold569
147	LdecGR93		825	235673-244887	plus	1|0	362	New gene model

Table 16S. Details on the *L. decemlineata* ionotropic receptor (IR) gene family. Columns include the gene name (suffixes are CTE – C-terminus unidentified; FIX – assembly was repaired; INT – internal regions missing; JOI – gene model crosses two scaffolds; NTE – N-terminus unidentified; multiple suffixes are abbreviated to single letters), the official gene number in OGS_v0.5.3 (prefix is LdecTmpB), the presence of a partial gene model from AUGUSTUS, the genome assembly scaffold ID, the scaffold coordinates from the first position of the start codon to the last position of the stop codon, the +/- (forward/reverse) strand, the number of introns, the number of encoded amino acids (AAs), and any comments.

Gene	OGS	AUG	Scaffold	Coordinates	Strand	Introns	AAs	Comments
Ir25aFIX	8041	yes	104	185086-204625	+	11	921	Fix assembly
Ir8aJOI	018065/6	yes	893	<1-146430	-	13	877	Join across scaffolds
	16856	yes	1100	<1-43957	+			
Ir21aJIC	4186	yes	25	<1-3863	-	>7	593	Join across scaffolds
	15742	yes	662	<424977->439062	-			2 exons missing
Ir40aNJF	-	no	637	<367286->379960	+	13	708	Join across scaffolds
	-	no	1467	<56957->68387	-			Fix assembly
Ir41a	10367	yes	415	735442-739232	+	6	601	Add 1 exon
Ir68aNJF	-	no	1446	<74948->103949	+	8	720	Join across scaffolds
	20499	yes	1194	9332->40161	-			Fix assembly
Ir75aNTE	6338	yes	75	<55171-66090	+	>5	584	N-terminus missing
Ir75b	017661/2	no	867	78631-106333	-	8	583	Merge/fix models
Ir75c	14737	no	520	351878-391144	+	9	661	Add 2 exons
Ir75d	1507	yes	4	740524-769235	+	8	598	Add 5 exons
Ir75e	18658	no	1065	128744-142003	-	9	561	Add 6 exons
Ir75f	11282	no	254	126921-159481	+	7	618	Multiple changes
Ir76bINT	19191	yes	1041	82844-98654	+	6	506	1 exon missing
Ir93aJF	1737	no	13	40870-75015	+	13	893	Join across scaffolds
	24011	no	6315	<1->17744	-			Fix assembly
Ir100a	-	yes	600	503039-504892	+	0	617	New gene model
Ir101	-	yes	140	1068417-1070366	-	0	649	New gene model
Ir102	7040	yes	82	145788-147644	-	0	618	Split off from model
Ir103	-	yes	1871	25247-27100	+	0	617	New gene model
Ir104	-	yes	761	56201-58093	+	0	630	New gene model
Ir105	-	yes	761	63556-65403	+	0	615	New gene model
Ir106NTE	-	yes	2245	<32801-34087	+	0	428	N-terminus unclear
Ir107NTE	-	yes	2	6282019->6293049	-	2	496	N-terminus unclear
Ir108	-	yes	496	277847-279595	+	0	582	New gene model
Ir109	4653	yes	56	1281808-1284225	-	1	605	Small modifications

Ir110FIX	-	yes	42	1213802->1214740	-	1	581	Fix assembly
Ir111FIX	-	no	23	966565-968039	-	1	475	Fix assembly
Ir112	-	yes	23	1360428-1372830	+	1	554	New gene model

Table 17S. Gene annotations related to diapause and environmental stress in *Leptinotarsa. decemlineata*.

Gene ID	Scaffold	Start Position	End Position	Gene Name
LDEC003084-RA	Scaffold43	1167070	1167700	bric-a-brac 1
LDEC019265-RA	Scaffold1120	213092	213784	dichaete
LDEC003913-RA	Scaffold57	2192436	2200132	DnaJ and jiv90 domain containing protein
LDEC017329-RA	Scaffold834	228547	230811	DnaJ and SANT domain containing protein
LDEC010647-RA	Scaffold285	528018	537055	DnaJ and TPR domain containing protein
LDEC007149-RA	Scaffold149	316924	318611	DnaJ doamin containing protein
LDEC024548-RA	Scaffold13267	4009	4904	DnaJ domain containing protein
LDEC022521-RA	Scaffold2146	66416	79190	DnaJ domain containing protein
LDEC021056-RA	Scaffold1545	78906	82889	DnaJ domain containing protein
LDEC000650-RA	Scaffold6	1669491	1671354	DnaJ domain containing protein
LDEC006429-RA	Scaffold122	95226	98458	DnaJ domain containing protein
LDEC007985-RA	Scaffold175	704059	706339	DnaJ domain containing protein
LDEC002209-RA	Scaffold27	651115	653628	DnaJ domain containing protein
LDEC019115-RA	Scaffold1096	497666	502072	DnaJ domain containing protein
LDEC008386-RB	Scaffold192	524400	542731	DnaJ Domain containing protein
LDEC020375-RA	Scaffold1361	39526	62988	DnaJ domain containing protein
LDEC014729-RA	Scaffold576	139794	140840	DnaJ domain containing protein
LDEC008386-RA	Scaffold192	531396	542731	DnaJ domain containing protein
LDEC019149-RA	Scaffold1101	74390	104752	DnaJ domain containing protein
LDEC011829-RA	Scaffold355	656964	686449	DnaJ domain containing protein
LDEC000893-RA	Scaffold9	313303	336353	DnaJ domain containing protein
LDEC003497-RA	Scaffold49	497969	508973	DnaJ domain containing protein
LDEC020072-RA	Scaffold1292	136606	137947	DnaJ domain containing protein
LDEC002386-RA	Scaffold30	254702	255652	DnaJ domain containing protein
LDEC016188-RA	Scaffold721	329871	339867	DnaJ Domain containing protein
LDEC009406-RA	Scaffold231	282675	288041	DnaJ domain containing protein
LDEC001676-RA	Scaffold18	291789	293676	DnaJ domain containing protein
LDEC019656-RA	Scaffold1198	210413	211089	DnaJ domain containing protein
LDEC003497-RB	Scaffold49	497870	499240	DnaJ domain contining protein
LDEC014517-RA	Scaffold557	685166	689287	DnaJ domin containing protein
LDEC011408-RA	Scaffold331	391865	396687	DnaJ-like protein
LDEC019872-RA	Scaffold1252	121816	122539	doublesex
LDEC009502-RA	Scaffold237	120853	614211	ecdysone receptor isoform A
LDEC009268-RA	Scaffold224	712433	721328	ecdysone-induced protein 74EF
LDEC018838-RA	Scaffold1050	40825	44678	Gamma interferon inducible lysosomal thiol reductase
LDEC012199-RA	Scaffold382	61068	63448	Glycine-rich protein
LDEC010403-RA	Scaffold278	193550	195652	Glycine-rich protein

LDEC010404-RA	Scaffold278	197109	197685	Glycine-rich protein
LDEC021900-RA	Scaffold1831	68817	69965	Heat Shock 60 Protein
LDEC002760-RA	Scaffold34	1214934	1232179	Heat Shock Protein -10
LDEC008523-RA	Scaffold196	558587	559554	Heat shock protein 70
LDEC001138-RA	Scaffold12	1343370	1353199	Heat shock protein 70 -2
LDEC001140-RA	Scaffold12	1359841	1361800	Heat Shock Protein 70 -3
LDEC001137-RA	Scaffold12	1337216	1339281	Heat Shock Protein 70 -4
LDEC016280-RA	Scaffold725	274798	277042	Heat Shock Protein 70 -5
LDEC000250-RA	Scaffold2	4595862	4605333	Heat Shock Protein 70 -6
LDEC018987-RA	Scaffold1075	207433	211998	Heat Shock Protein 70 -7
LDEC010172-RA	Scaffold270	139718	140416	Heat Shock Protein 70 -8
LDEC007545-RA	Scaffold159	991361	994892	Heat Shock Protein -9
LDEC000387-RA	Scaffold3	3423742	3427229	heat shock protein 90
LDEC004651-RA	Scaffold70	655331	656407	Insulin-like peptide
LDEC006817-RA	Scaffold134	1070321	1169129	Myc, diminutive
LDEC012832-RA	Scaffold422	146876	149047	myc-associated factor X
LDEC000329-RA	Scaffold3	520279	520815	small heat shock protein
LDEC007977-RA	Scaffold175	496415	497139	small heat shock protein
LDEC003106-RA	Scaffold44	406164	406986	small heat shock protein
LDEC007984-RA	Scaffold175	695270	695960	small heat shock protein
LDEC022122-RA	Scaffold1922	369336	371977	small heat shock protein
LDEC003107-RA	Scaffold44	410321	411058	small heat shock protein
LDEC007978-RA	Scaffold175	501123	501589	small heat shock protein
LDEC007976-RA	Scaffold175	476884	478051	small heat shock protein
LDEC003108-RA	Scaffold44	418999	419732	small heat shock protein
LDEC008130-RA	Scaffold181	451524	452539	small heat shock protein
LDEC000330-RA	Scaffold3	523395	524370	small heat shock protein
LDEC007975-RA	Scaffold175	473963	474701	small heat shock protein
LDEC011463-RA	Scaffold335	221556	239704	small heat shock protein

Table 18S. Cysteine peptidases of *Leptinotarsa decemlineata*. Peptidase classification described in the Supplementary Methods.

Peptidase	C1 Peptidase Family	Functional Group	Intestain Group	NCBI ID	UniProt ID
CatL	L	L		-	-
CatF	L	F		-	-
CatO	L	O		-	-
CatI	L	I		-	-
CatLl.1	L	Ll		-	-
CatLl.2	L	Ll		-	-
CatLc	L	Lc		-	-
Cat.ss.uL1.1	L	ss.uL1		-	-
Cat.ss.uL1.2	L	ss.uL1		-	-
Cat.ss.uL1.3	L	ss.uL1	C	-	Q8I880
Cat.ss.uL1.4	L	ss.uL1		-	-
Cat.ss.uL1.5	L	ss.uL1		-	-
Cat.ss.uL1.6	L	ss.uL1	C	AAN77416	Q8I879<br>Q8I881<br>Q8I882<br>Q6QRP5
Cat.ss.uL1.7	L	ss.uL1		-	-
Cat.ss.uL1.8	L	ss.uL1		-	-
Cat.ss.uL2.1	L	ss.uL2		-	-
Cat.ss.uL2.2	L	ss.uL2		-	-
Cat.ss.uL2.3	L	ss.uL2		-	-
Cat.ss.uL2.4	L	ss.uL2		-	-
Cat.ss.uL2.5	L	ss.uL2		-	-
Cat.ss.uL2.6	L	ss.uL2		-	-
Cat.ss.uL2.7	L	ss.uL2	A	AAN77408<br>AAS20589<br>AAS20588<br>AAS20590	Q6QRP9<br>Q6QRQ0<br>Q6QRP8<br>Q8I888<br>Q8I887
Cat.ss.uL2.8	L	ss.uL2		-	-
Cat.ss.uL2.9	L	ss.uL2	D	ABM55489	-
Cat.ss.uL2.10	L	ss.uL2	D	ABM55487<br>ABM55490	A2I7Q1<br>A2I7Q2
Cat.ss.uL2.11	L	ss.uL2		-	-
Cat.ss.uL2.12	L	ss.uL2	E	ABM55481<br>ABM55480	A2I7N8<br>A2I7N9
Cat.ss.uL2.13	L	ss.uL2	E	ABM55477	-
Cat.ss.uL2.14	L	ss.uL2	B	AAN77412<br>AAS20592<br>AAS20591	Q6QRP6<br>Q6QRP7<br>Q8I885<br>Q8I884<br>Q8I883
Cat.ss.uL3.1	L	ss.uL3		-	-
Cat.ss.uL4.1	L	ss.uL4		-	-
CatB	B	B		-	-
Cat.ss.uB1.1	B	ss.uB1		-	-
Cat.ss.uB1.2	B	ss.uB1		-	-
Cat.ss.uB1.3	B	ss.uB1		-	-
Cat.ss.uB1.4	B	ss.uB1		-	-
Cat.ss.uB1.5	B	ss.uB1		-	-
Cat.ss.uB1.6	B	ss.uB1		-	-
Cat.ss.uB1.7	B	ss.uB1		-	-
Cat.ss.uB1.8	B	ss.uB1		-	-
Cat.ss.uB1.9	B	ss.uB1		-	-
Cat.ss.uB1.10	B	ss.uB1		-	-
Cat.ss.uB1.11	B	ss.uB1		-	-
Cat.ss.uB1.12	B	ss.uB1		-	-
Cat.ss.uB1.13	B	ss.uB1		-	-
Cat.ss.uB1.14	B	ss.uB1		-	-
CatBSer	TINAL	TINAL-like		AGX25160	V9PBG4

Table 19S. Serine peptidases of *Leptinotarsa decemlineata.*

**Genome position**	**Name**	**Description**	**Symbol**
Scaffold2:3144960..3164282	chymotrypsin-like 1	serine peptidase chymotrypsin-like	CTR1
Scaffold37:1258337..1276789	chymotrypsin-like 2	serine peptidase chymotrypsin-like	CTR2
Scaffold220:681841..702323	chymotrypsin-like 3 (partial)	serine peptidase chymotrypsin-like	CTR3
Scaffold333:88603..95293	chymotrypsin-like 4	serine peptidase chymotrypsin-like	CTR4
Scaffold355:106567..137196	chymotrypsin-like 5 (partial)	serine peptidase chymotrypsin-like	CTR5
Scaffold355:176564..214045	chymotrypsin-like 6	serine peptidase chymotrypsin-like	CTR6
Scaffold355:253512..268845	chymotrypsin-like 7 (partial)	serine peptidase chymotrypsin-like	CTR7
Scaffold495:126587..145129	chymotrypsin-like 8	serine peptidase chymotrypsin-like	CTR8
Scaffold543:220754..226799	chymotrypsin-like 9	serine peptidase chymotrypsin-like	CTR9
Scaffold732:39458..48849	chymotrypsin-like 10	serine peptidase chymotrypsin-like	CTR10
Scaffold761:289769..301320	chymotrypsin-like 11	serine peptidase chymotrypsin-like	CTR11
Scaffold1404:18032-80703_A	chymotrypsin-like 12	serine peptidase chymotrypsin-like	CTR12
Scaffold1404:18032-80703_B	chymotrypsin-like 13	serine peptidase chymotrypsin-like	CTR13
Scaffold2238:32691..50979	chymotrypsin-like 14	serine peptidase chymotrypsin-like	CTR14
Scaffold5219:1415..9598	chymotrypsin-like 15 (partial)	serine peptidase chymotrypsin-like	CTR15
Scaffold3:1837754..1841781	trypsin-like 1	serine peptidase trypsin-like	TRY1
Scaffold18:1724283..1749300	trypsin-like 2	serine peptidase trypsin-like	TRY2
Scaffold107:802377..808411	trypsin-like 3	serine peptidase trypsin-like	TRY3
Scaffold107:845267..861201	trypsin-like 4 (partial)	serine peptidase trypsin-like	TRY4
Scaffold132:836684..844985	trypsin-like 5	serine peptidase trypsin-like	TRY5
Scaffold138:993736..1011540	trypsin-like 6	serine peptidase trypsin-like	TRY6
Scaffold138:1011997..1028911	trypsin-like 7	serine peptidase trypsin-like	TRY7
Scaffold303:44823..47028	trypsin-like 8	serine peptidase trypsin-like	TRY8
Scaffold1075:257861..261430	trypsin-like 9	serine peptidase trypsin-like	TRY9
Scaffold1538:37269..48673	trypsin-like 10	serine peptidase trypsin-like	TRY10
Scaffold1615:103103..110212_A	trypsin-like 11	serine peptidase trypsin-like	TRY11
Scaffold1615:103103..110212_B	trypsin-like 12	serine peptidase trypsin-like	TRY12
Scaffold1615:103103..110212_C	trypsin-like 13	serine peptidase trypsin-like	TRY13
Scaffold1775:13820..27225	trypsin-like 14	serine peptidase trypsin-like	TRY14
Scaffold2630:36557..42503	trypsin-like 15	serine peptidase trypsin-like	TRY15
Scaffold2630:53494..62845	trypsin-like 16	serine peptidase trypsin-like	TRY16

Table 20S. Summary of the number of *Leptinotarsa decemlineata* protein models assigned to families of the CAZy database and comparison to other insects.

**GH family**	1	2	9	13	16	18	20	22	24	27	28	29	30	31	32	35	37	38	39	43	45	47	48	56	63	65	79	84	85	89	99
Leptinotarsa decemlineata	49	1	0	6	7	20	11	7	0	3	15	2	1	11	0	8	3	9	0	0	13	7	2	1	1	0	0	1	1	1	0
Tribolium castaneum	15	8	2	15	4	39	16	9	0	6	0	7	5	14	0	4	10	12	1	0	0	5	0	1	1	0	2	2	2	2	1
Danaus plexippus	29	2	0	15	6	21	9	13	0	5	0	2	6	9	4	1	3	11	0	0	0	5	0	2	1	1	2	1	1	1	0
Zootermopsis nevadensis	9	4	7	11	6	18	9	7	0	2	0	2	2	6	0	1	3	3	2	0	0	5	0	1	1	0	2	1	1	1	1
Acyrthosiphum pisum	14	3	1	29	4	13	8	3	1	6	0	1	1	8	0	5	15	4	0	1	0	6	0	1	1	0	1	1	2	0	0
Drosophila melanogaster	1	4	0	16	3	22	4	19	0	2	0	1	2	3	0	2	2	8	1	0	0	6	0	0	1	1	0	1	1	1	1

**GH family cont.**	116	133	Total	**GT family**	1	2	3	4	7	8	10	11	13	14	16	17	20	21	22	23	24	25	27	29	31	32	33	35
Leptinotarsa decemlineata	1	1	182		57	9	1	4	6	6	5	1	2	1	2	1	1	2	4	1	1	1	12	1	18	1	1	1
Tribolium castaneum	1	1	185		50	7	2	4	10	6	10	1	2	1	3	1	1	1	4	1	2	1	11	1	24	2	1	1
Danaus plexippus	1	1	152		47	7	1	4	8	3	11	0	1	1	6	0	2	1	6	1	2	1	12	1	12	2	1	1
Zootermopsis nevadensis	0	1	106		28	6	1	4	6	4	4	2	5	1	1	1	2	1	4	1	1	1	9	1	15	7	1	1
Acyrthosiphum pisum	1	1	131		75	4	3	3	7	6	7	0	4	1	3	0	4	1	5	10	1	1	9	1	9	2	1	2
Drosophila melanogaster	1	1	104		37	5	1	4	5	3	5	0	1	1	1	1	1	1	4	1	1	1	15	1	23	2	1	1

**GT family continued**	39	41	43	47	49	50	54	57	58	59	61	64	65	66	68	76	90	92	Total
Leptinotarsa decemlineata	5	1	4	4	5	0	2	3	1	1	1	3	1	3	1	1	2	4	181
Tribolium castaneum	2	2	6	3	11	1	1	2	1	1	1	3	1	2	1	5	2	8	200
Danaus plexippus	2	2	2	3	4	1	3	2	1	1	2	4	1	2	1	1	3	3	169
Zootermopsis nevadensis	2	1	3	5	6	1	1	2	1	1	1	3	1	2	1	1	2	3	144
Acyrthosiphum pisum	3	7	4	2	3	0	3	0	0	0	1	2	1	4	1	1	2	3	196
Drosophila melanogaster	2	1	3	3	4	1	2	2	1	1	1	3	1	2	1	1	2	4	151

CBM family	13	14	18	20	21	39	47	48	50	57	Total	CE family	4	9	13	Total
Leptinotarsa decemlineata	7	81	0	1	2	4	1	1	1	1	99		0	1	1	2
Tribolium castaneum	22	165	0	2	3	4	2	2	1	1	202		0	1	1	2
Danaus plexippus	8	77	0	1	2	5	2	2	1	1	99		0	1	0	1
Zootermopsis nevadensis	12	53	0	1	2	2	2	2	0	1	75		0	1	0	1
Acyrthosiphum pisum	10	57	3	0	1	0	1	2	1	1	76		2	1	0	3
Drosophila melanogaster	14	246	0	5	2	6	2	2	1	1	279		0	1	0	1

Table 21S. Carbohydrate active enzymes and gene family assignments in the *Leptinotarsa decemlineata* genome.

Protein ID	Description (family-subfamily)	Definition	Notes
LDEC000058_PA	GT35	Glycosyltransferase Family 35 protein	splicing problem;
LDEC000098_PA	GT1-GT1	Glycosyltransferase Family 1 protein	
LDEC000146_PA	GT27-CBM13	Glycosyltransferase Family 27 / Carbohydrate-Binding Module Family 13 protein	
LDEC000249_PA	GT22	Glycosyltransferase Family 22 protein	splicing problem;
LDEC000262_PA	GT1	Glycosyltransferase Family 1 protein	splicing problem;
LDEC000757_PA	GH45	Glycoside Hydrolase Family 45 protein	
LDEC000758_PA	GH45-GH45-GH45-GH45-GH45	Glycoside Hydrolase Family 45 protein	
LDEC000918_PA	GH1	Glycoside Hydrolase Family 1 protein	
LDEC000919_PA	GH1	Glycoside Hydrolase Family 1 protein	fragment N-term;
LDEC000920_PA	GH1	Glycoside Hydrolase Family 1 protein	fragment C-term;
LDEC000922_PA	GH1	Glycoside Hydrolase Family 1 protein	
LDEC001117_PA	GH37	Glycoside Hydrolase Family 37 protein	
LDEC001221_PA	GH1	Glycoside Hydrolase Family 1 protein	splicing problem;
LDEC001222_PA	GH1	Glycoside Hydrolase Family 1 protein	fragment N-term;
LDEC001223_PA	GH1-GH1	Glycoside Hydrolase Family 1 protein	splicing problem;
LDEC001224_PA	GH1	Glycoside Hydrolase Family 1 protein	fragment N-term;
LDEC001284_PA	GH18	Glycoside Hydrolase Family 18 protein	
LDEC001563_PA	GT16	Glycosyltransferase Family 16 protein	splicing problem;
LDEC001677_PA	GH28	Glycoside Hydrolase Family 28 protein	fragment N-term;
LDEC001678_PA	GH28	Glycoside Hydrolase Family 28 protein	fragment C-term;
LDEC001954_PA	CBM14-CBM14	Carbohydrate-Binding Module Family 14 protein	
LDEC002020_PA	GH31	Glycoside Hydrolase Family 31 protein	
LDEC002040_PA	GT76	Glycosyltransferase Family 76 protein	
LDEC002215_PA	CBM50	Carbohydrate-Binding Module Family 50 protein	
LDEC002262_PA	GT1	Glycosyltransferase Family 1 protein	fragment C-term;
LDEC002295_PA	GT27-CBM13	Glycosyltransferase Family 27 / Carbohydrate-Binding Module Family 13 protein	
LDEC002383_PA	GT10	Glycosyltransferase Family 10 protein	
LDEC002539_PA	GT92	Glycosyltransferase Family 92 protein	
LDEC002637_PA	GT65	Glycosyltransferase Family 65 protein	
LDEC002670_PA	GT33	Glycosyltransferase Family 33 protein	
LDEC002758_PA	CBM14	Carbohydrate-Binding Module Family 14 protein	

LDEC002760_PA	CBM14	Carbohydrate-Binding Module Family 14 protein	fragment N-term;
LDEC002972_PA	GT27-CBM13	Glycosyltransferase Family 27 / Carbohydrate-Binding Module Family 13 protein	splicing problem;
LDEC002992_PA	GT31	Glycosyltransferase Family 31 protein	fragment C-term;
LDEC003178_PA	CBM14	Carbohydrate-Binding Module Family 14 protein	
LDEC003218_PA	GT4	Glycosyltransferase Family 4 protein	
LDEC003254_PA	GH28	Glycoside Hydrolase Family 28 protein	
LDEC003256_PA	GH28-GH28	Glycoside Hydrolase Family 28 protein	fragment N-term / C-term;
LDEC003388_PA	GT27	Glycosyltransferase Family 27 protein	
LDEC003493_PA	CBM39	Carbohydrate-Binding Module Family 39 protein	
LDEC003566_PA	GH35	Glycoside Hydrolase Family 35 protein	fragment C-term;
LDEC003567_PA	GH35	Glycoside Hydrolase Family 35 protein	fragment N-term;
LDEC003681_PA	GH31	Glycoside Hydrolase Family 31 protein	fragment N-term;
LDEC003812_PA	GT27-CBM13	Glycosyltransferase Family 27 / Carbohydrate-Binding Module Family 13 protein	splicing problem;
LDEC003817_PA	GH27	Glycoside Hydrolase Family 27 protein	fragment N-term;
LDEC003818_PA	GH27	Glycoside Hydrolase Family 27 protein	fragment C-term;
LDEC003862_PA	GH13_25-GH133	Glycoside Hydrolase Family 13 / Glycoside Hydrolase Family 133 protein	fragment N-term;
LDEC003867_PA	GT7	Glycosyltransferase Family 7 protein	
LDEC003889_PA	GH18-CBM14	Glycoside Hydrolase Family 18 / Carbohydrate-Binding Module Family 14 protein	
LDEC004009_PA	GT41	Glycosyltransferase Family 41 protein	
LDEC004057_PA	GH20	Glycoside Hydrolase Family 20 protein	
LDEC004105_PA	GT2	Glycosyltransferase Family 2 protein	
LDEC004433_PA	GT66	Glycosyltransferase Family 66 protein	fragment C-term;
LDEC004443_PA	GH18	Glycoside Hydrolase Family 18 protein	splicing problem;
LDEC004625_PA	GH22-GH22	Glycoside Hydrolase Family 22 protein	
LDEC004665_PA	GT1-GT1	Glycosyltransferase Family 1 protein	splicing problem;
LDEC004666_PA	GT1	Glycosyltransferase Family 1 protein	fragment C-term;
LDEC004775_PA	GT1	Glycosyltransferase Family 1 protein	fragment N-term;
LDEC004778_PA	GT1	Glycosyltransferase Family 1 protein	fragment N-term / C-term;
LDEC004785_PA	GT2	Glycosyltransferase Family 2 protein	
LDEC004844_PA	GH48	Glycoside Hydrolase Family 48 protein	
LDEC004876_PA	CBM14-CBM14-CBM14	Carbohydrate-Binding Module Family 14 protein	
LDEC004878_PA	CBM14-CBM14	Carbohydrate-Binding Module Family 14 protein	
LDEC004947_PA	GT16	Glycosyltransferase Family 16 protein	fragment C-term;
LDEC005220_PA	GH2	Glycoside Hydrolase Family 2 protein	fragment C-term;
LDEC005328_PA	GT7	Glycosyltransferase Family 7 protein	fragment C-term;

LDEC005362_PA	GT31	Glycosyltransferase Family 31 protein	
LDEC005425_PA	GH28	Glycoside Hydrolase Family 28 protein	
LDEC005440_PA	GT14	Glycosyltransferase Family 14 protein	splicing problem;
LDEC005483_PA	GH47	Glycoside Hydrolase Family 47 protein	fragment N-term;
LDEC005484_PA	GH47	Glycoside Hydrolase Family 47 protein	fragment C-term;
LDEC005654_PA	CBM14-CBM14	Carbohydrate-Binding Module Family 14 protein	
LDEC005655_PA	CBM14-CBM14	Carbohydrate-Binding Module Family 14 protein	
LDEC005681_PA	GH20	Glycoside Hydrolase Family 20 protein	
LDEC005769_PA	GT8	Glycosyltransferase Family 8 protein	fragment N-term / C-term;
LDEC005885_PA	CBM14	Carbohydrate-Binding Module Family 14 protein	
LDEC005975_PA	GH18	Glycoside Hydrolase Family 18 protein	splicing problem;
LDEC005976_PA	GH18	Glycoside Hydrolase Family 18 protein	splicing problem;
LDEC005991_PA	GH1	Glycoside Hydrolase Family 1 protein	fragment N-term;
LDEC005992_PA	GH1	Glycoside Hydrolase Family 1 protein	fragment C-term;
LDEC006132_PA	GT57	Glycosyltransferase Family 57 protein	
LDEC006175_PA	GH30_1	Glycoside Hydrolase Family 30 protein	splicing problem;
LDEC006184_PA	GH38	Glycoside Hydrolase Family 38 protein	fragment N-term;
LDEC006194_PA	GT31	Glycosyltransferase Family 31 protein	
LDEC006201_PA	GT90	Glycosyltransferase Family 90 protein	
LDEC006204_PA	GT29	Glycosyltransferase Family 29 protein	fragment N-term;
LDEC006296_PA	GT32	Glycosyltransferase Family 32 protein	
LDEC006328_PA	GH18	Glycoside Hydrolase Family 18 protein	
LDEC006357_PA	GH48	Glycoside Hydrolase Family 48 protein	
LDEC006491_PA	GT1	Glycosyltransferase Family 1 protein	fragment N-term;
LDEC006564_PA	GH28	Glycoside Hydrolase Family 28 protein	fragment C-term;
LDEC006565_PA	GH28	Glycoside Hydrolase Family 28 protein	splicing problem;
LDEC006600_PA	GH31	Glycoside Hydrolase Family 31 protein	
LDEC006669_PA	GH1	Glycoside Hydrolase Family 1 protein	fragment N-term;
LDEC006726_PA	GT31	Glycosyltransferase Family 31 protein	fragment C-term;
LDEC006728_PA	GT31	Glycosyltransferase Family 31 protein	fragment N-term;
LDEC006732_PA	GT31	Glycosyltransferase Family 31 protein	splicing problem;
LDEC006754_PA	GH20	Glycoside Hydrolase Family 20 protein	
LDEC006840_PA	CE9	Carbohydrate Esterase Family 9 protein	fragment N-term;
LDEC006921_PA	GH18	Glycoside Hydrolase Family 18 protein	splicing problem;
LDEC006925_PA	GT3	Glycosyltransferase Family 3 protein	fragment C-term;

LDEC006973_PA	GT7	Glycosyltransferase Family 7 protein	
LDEC007036_PA	CBM14	Carbohydrate-Binding Module Family 14 protein	
LDEC007099_PA	GT1	Glycosyltransferase Family 1 protein	fragment N-term;
LDEC007424_PA	CBM14	Carbohydrate-Binding Module Family 14 protein	
LDEC007645_PA	GT47-GT64	Glycosyltransferase Family 47 / Glycosyltransferase Family 64 protein	fragment N-term / C-term;
LDEC007753_PA	GT22	Glycosyltransferase Family 22 protein	
LDEC007872_PA	GT58	Glycosyltransferase Family 58 protein	fragment N-term;
LDEC007964_PA	GH47	Glycoside Hydrolase Family 47 protein	fragment C-term;
LDEC008012_PA	GT61	Glycosyltransferase Family 61 protein	
LDEC008120_PA	GH28-GH28	Glycoside Hydrolase Family 28 protein	
LDEC008121_PA	GH28	Glycoside Hydrolase Family 28 protein	
LDEC008122_PA	GH28	Glycoside Hydrolase Family 28 protein	fragment N-term;
LDEC008123_PA	GH28	Glycoside Hydrolase Family 28 protein	
LDEC008408_PA	GH1	Glycoside Hydrolase Family 1 protein	fragment N-term / C-term;
LDEC008409_PA	GH1	Glycoside Hydrolase Family 1 protein	fragment N-term / C-term;
LDEC008413_PA	GH1	Glycoside Hydrolase Family 1 protein	fragment N-term / C-term;
LDEC008483_PA	GT31	Glycosyltransferase Family 31 protein	
LDEC008496_PA	GT2	Glycosyltransferase Family 2 protein	
LDEC008523_PA	GH1	Glycoside Hydrolase Family 1 protein	fragment N-term;
LDEC008524_PA	GH1	Glycoside Hydrolase Family 1 protein	fragment C-term;
LDEC008797_PA	CBM48-GH13_8	Carbohydrate-Binding Module Family 48 / Glycoside Hydrolase Family 13 protein	splicing problem;
LDEC008865_PA	CBM14	Carbohydrate-Binding Module Family 14 protein	fragment N-term;
LDEC008885_PA	CE13	Carbohydrate Esterase Family 13 protein	
LDEC008907_PA	GT90	Glycosyltransferase Family 90 protein	
LDEC008993_PA	GT1	Glycosyltransferase Family 1 protein	fragment N-term;
LDEC009094_PA	GT49	Glycosyltransferase Family 49 protein	
LDEC009104_PA	GH28	Glycoside Hydrolase Family 28 protein	
LDEC009178_PA	GT1	Glycosyltransferase Family 1 protein	
LDEC009179_PA	GT1	Glycosyltransferase Family 1 protein	
LDEC009180_PA	GH22	Glycoside Hydrolase Family 22 protein	
LDEC009181_PA	GT25	Glycosyltransferase Family 25 protein	fragment N-term;
LDEC009222_PA	GH18	Glycoside Hydrolase Family 18 protein	fragment C-term;
LDEC009223_PA	GH18	Glycoside Hydrolase Family 18 protein	fragment N-term;
LDEC009225_PA	CBM14	Carbohydrate-Binding Module Family 14 protein	fragment N-term;
LDEC009259_PA	GT27	Glycosyltransferase Family 27 protein	fragment N-term / C-term;

LDEC009260_PA	GT27	Glycosyltransferase Family 27 protein	fragment N-term / C-term;
LDEC009304_PA	GT10	Glycosyltransferase Family 10 protein	splicing problem;
LDEC009421_PA	CBM14	Carbohydrate-Binding Module Family 14 protein	
LDEC009423_PA	CBM14-CBM14-CBM14	Carbohydrate-Binding Module Family 14 protein	
LDEC009452_PA	GH1	Glycoside Hydrolase Family 1 protein	fragment C-term;
LDEC009453_PA	GH1	Glycoside Hydrolase Family 1 protein	fragment N-term;
LDEC009454_PA	GH1	Glycoside Hydrolase Family 1 protein	fragment C-term;
LDEC009568_PA	GH35	Glycoside Hydrolase Family 35 protein	fragment N-term;
LDEC009637_PA	GT49	Glycosyltransferase Family 49 protein	
LDEC009655_PA	CBM14-CBM14-CBM14	Carbohydrate-Binding Module Family 14 protein	fragment N-term;
LDEC009837_PA	GH18-GH18	Glycoside Hydrolase Family 18 protein	fragment N-term;
LDEC009893_PA	GT8-GT49	Glycosyltransferase Family 8 / Glycosyltransferase Family 49 protein	splicing problem;
LDEC010009_PA	GT10	Glycosyltransferase Family 10 protein	
LDEC010013_PA	GT68	Glycosyltransferase Family 68 protein	
LDEC010251_PA	GT31	Glycosyltransferase Family 31 protein	
LDEC010425_PA	GH18	Glycoside Hydrolase Family 18 protein	
LDEC010472_PA	GT31-GT7	Glycosyltransferase Family 31 / Glycosyltransferase Family 7 protein	splicing problem;
LDEC010495_PA	GT43	Glycosyltransferase Family 43 protein	fragment N-term;
LDEC010496_PA	GT43	Glycosyltransferase Family 43 protein	fragment C-term;
LDEC010572_PA	CBM14-CBM14-CBM14	Carbohydrate-Binding Module Family 14 protein	
LDEC010655_PA	GT39	Glycosyltransferase Family 39 protein	fragment N-term / C-term;
LDEC010658_PA	GT39	Glycosyltransferase Family 39 protein	fragment N-term / C-term;
LDEC010751_PA	GT4	Glycosyltransferase Family 4 protein	fragment C-term;
LDEC010780_PA	GH20	Glycoside Hydrolase Family 20 protein	fragment C-term;
LDEC010867_PA	GT54	Glycosyltransferase Family 54 protein	fragment N-term;
LDEC010935_PA	GT20	Glycosyltransferase Family 20 protein	
LDEC011081_PA	GT47-GT64	Glycosyltransferase Family 47 / Glycosyltransferase Family 64 protein	fragment N-term;
LDEC011082_PA	GT47	Glycosyltransferase Family 47 protein	fragment C-term;
LDEC011092_PA	GT1	Glycosyltransferase Family 1 protein	fragment C-term;
LDEC011093_PA	GT1	Glycosyltransferase Family 1 protein	fragment N-term;
LDEC011094_PA	GT1	Glycosyltransferase Family 1 protein	
LDEC011095_PA	GT1	Glycosyltransferase Family 1 protein	splicing problem;
LDEC011104_PA	GT1	Glycosyltransferase Family 1 protein	
LDEC011303_PA	GH16	Glycoside Hydrolase Family 16 protein	
LDEC011304_PA	GH16	Glycoside Hydrolase Family 16 protein	splicing problem;

LDEC011305_PA	GH16	Glycoside Hydrolase Family 16 protein	
LDEC011307_PA	CBM39	Carbohydrate-Binding Module Family 39 protein	fragment C-term;
LDEC011309_PA	CBM39-GH16	Carbohydrate-Binding Module Family 39 / Glycoside Hydrolase Family 16 protein	fragment C-term;
LDEC011316_PA	CBM39-GH16	Carbohydrate-Binding Module Family 39 / Glycoside Hydrolase Family 16 protein	fragment C-term;
LDEC011381_PA	GH31	Glycoside Hydrolase Family 31 protein	
LDEC011383_PA	GH31	Glycoside Hydrolase Family 31 protein	fragment N-term;
LDEC011387_PA	GH31	Glycoside Hydrolase Family 31 protein	
LDEC011408_PA	GT24	Glycosyltransferase Family 24 protein	fragment N-term;
LDEC012050_PA	GT1	Glycosyltransferase Family 1 protein	fragment N-term;
LDEC012051_PA	GT1	Glycosyltransferase Family 1 protein	fragment C-term;
LDEC012054_PA	GT1	Glycosyltransferase Family 1 protein	
LDEC012055_PA	GT1	Glycosyltransferase Family 1 protein	splicing problem;
LDEC012057_PA	GT1	Glycosyltransferase Family 1 protein	fragment C-term;
LDEC012058_PA	GT1	Glycosyltransferase Family 1 protein	splicing problem;
LDEC012142_PA	GT31	Glycosyltransferase Family 31 protein	fragment C-term;
LDEC012144_PA	GT31	Glycosyltransferase Family 31 protein	fragment N-term / C-term;
LDEC012213_PA	GT57	Glycosyltransferase Family 57 protein	fragment N-term;
LDEC012235_PA	GT22	Glycosyltransferase Family 22 protein	
LDEC012346_PA	GT39	Glycosyltransferase Family 39 protein	fragment C-term;
LDEC012369_PA	GT11	Glycosyltransferase Family 11 protein	splicing problem;
LDEC012670_PA	GT31-GT7	Glycosyltransferase Family 31 / Glycosyltransferase Family 7 protein	fragment N-term;
LDEC012672_PA	GT31	Glycosyltransferase Family 31 protein	fragment C-term;
LDEC012833_PA	CBM47	Carbohydrate-Binding Module Family 47 protein	
LDEC012852_PA	GH18-CBM14	Glycoside Hydrolase Family 18 / Carbohydrate-Binding Module Family 14 protein	fragment N-term;
LDEC012853_PA	GH18	Glycoside Hydrolase Family 18 protein	fragment N-term / C-term;
LDEC013009_PA	CBM14	Carbohydrate-Binding Module Family 14 protein	fragment C-term;
LDEC013020_PA	GT13	Glycosyltransferase Family 13 protein	fragment C-term;
LDEC013021_PA	GT13	Glycosyltransferase Family 13 protein	fragment N-term;
LDEC013356_PA	GH56	Glycoside Hydrolase Family 56 protein	fragment N-term / C-term;
LDEC013419_PA	GH47	Glycoside Hydrolase Family 47 protein	
LDEC013485_PA	GH63	Glycoside Hydrolase Family 63 protein	fragment N-term;
LDEC013542_PA	CBM14	Carbohydrate-Binding Module Family 14 protein	
LDEC013574_PA	GH38	Glycoside Hydrolase Family 38 protein	fragment N-term / C-term;
LDEC013575_PA	GH38	Glycoside Hydrolase Family 38 protein	fragment C-term;
LDEC013663_PA	CBM21	Carbohydrate-Binding Module Family 21 protein	

LDEC013918_PA	GH13_17	Glycoside Hydrolase Family 13 protein	fragment N-term;
LDEC013919_PA	GH13_17	Glycoside Hydrolase Family 13 protein	fragment C-term;
LDEC014083_PA	GT1	Glycosyltransferase Family 1 protein	fragment C-term;
LDEC014085_PA	GT1	Glycosyltransferase Family 1 protein	fragment C-term;
LDEC014086_PA	GT1	Glycosyltransferase Family 1 protein	fragment N-term / C-term;
LDEC014088_PA	GT1	Glycosyltransferase Family 1 protein	
LDEC014089_PA	GT1	Glycosyltransferase Family 1 protein	splicing problem;
LDEC014114_PA	CBM21	Carbohydrate-Binding Module Family 21 protein	
LDEC014138_PA	GH1	Glycoside Hydrolase Family 1 protein	fragment N-term;
LDEC014139_PA	GH1	Glycoside Hydrolase Family 1 protein	fragment N-term;
LDEC014140_PA	GH1	Glycoside Hydrolase Family 1 protein	fragment C-term;
LDEC014141_PA	GH1	Glycoside Hydrolase Family 1 protein	fragment N-term / C-term;
LDEC014142_PA	GH1	Glycoside Hydrolase Family 1 protein	fragment N-term;
LDEC014276_PA	GH35	Glycoside Hydrolase Family 35 protein	fragment C-term;
LDEC014277_PA	GH35	Glycoside Hydrolase Family 35 protein	
LDEC014278_PA	GH35-GH35	Glycoside Hydrolase Family 35 protein	
LDEC014296_PA	GH1	Glycoside Hydrolase Family 1 protein	fragment C-term;
LDEC014321_PA	CBM14	Carbohydrate-Binding Module Family 14 protein	
LDEC014453_PA	GT1	Glycosyltransferase Family 1 protein	fragment C-term;
LDEC014454_PA	GT1-GT1	Glycosyltransferase Family 1 protein	splicing problem;
LDEC014455_PA	GT1	Glycosyltransferase Family 1 protein	
LDEC014554_PA	CBM57	Carbohydrate-Binding Module Family 57 protein	
LDEC014572_PA	GT8	Glycosyltransferase Family 8 protein	fragment C-term;
LDEC014573_PA	GT10	Glycosyltransferase Family 10 protein	splicing problem;
LDEC014575_PA	GT10	Glycosyltransferase Family 10 protein	fragment C-term;
LDEC014707_PA	GT31	Glycosyltransferase Family 31 protein	
LDEC014767_PA	GH31	Glycoside Hydrolase Family 31 protein	fragment N-term;
LDEC014768_PA	GH31	Glycoside Hydrolase Family 31 protein	fragment C-term;
LDEC014788_PA	GH45	Glycoside Hydrolase Family 45 protein	
LDEC014789_PA	GH45	Glycoside Hydrolase Family 45 protein	
LDEC014790_PA	GH45	Glycoside Hydrolase Family 45 protein	
LDEC014791_PA	GH45	Glycoside Hydrolase Family 45 protein	
LDEC014800_PA	GH28	Glycoside Hydrolase Family 28 protein	
LDEC015018_PA	GH18	Glycoside Hydrolase Family 18 protein	splicing problem;
LDEC015059_PA	GT92	Glycosyltransferase Family 92 protein	fragment N-term;
LDEC015060_PA	GT92	Glycosyltransferase Family 92 protein	fragment N-term / C-term;
LDEC015115_PA	GT47-GT64	Glycosyltransferase Family 47 / Glycosyltransferase Family 64	

		protein	
LDEC015144_PA	GH89	Glycoside Hydrolase Family 89 protein	fragment C-term;
LDEC015162_PA	CBM20	Carbohydrate-Binding Module Family 20 protein	
LDEC015309_PA	CBM14-CBM14	Carbohydrate-Binding Module Family 14 protein	splicing problem;
LDEC015422_PA	GT1	Glycosyltransferase Family 1 protein	fragment C-term;
LDEC015423_PA	GT1	Glycosyltransferase Family 1 protein	fragment N-term;
LDEC015531_PA	GH22	Glycoside Hydrolase Family 22 protein	
LDEC015659_PA	GH1	Glycoside Hydrolase Family 1 protein	
LDEC015668_PA	GT27	Glycosyltransferase Family 27 protein	fragment C-term;
LDEC015669_PA	GT27	Glycosyltransferase Family 27 protein	fragment N-term / C-term;
LDEC015670_PA	GT27-CBM13	Glycosyltransferase Family 27 / Carbohydrate-Binding Module Family 13 protein	fragment N-term;
LDEC015685_PA	GT1	Glycosyltransferase Family 1 protein	
LDEC015701_PA	CBM14	Carbohydrate-Binding Module Family 14 protein	
LDEC015757_PA	GH18	Glycoside Hydrolase Family 18 protein	fragment C-term;
LDEC015758_PA	GH18	Glycoside Hydrolase Family 18 protein	
LDEC015759_PA	GH18	Glycoside Hydrolase Family 18 protein	
LDEC015793_PA	CBM14-CBM14-CBM14	Carbohydrate-Binding Module Family 14 protein	
LDEC015864_PA	CBM14	Carbohydrate-Binding Module Family 14 protein	splicing problem;
LDEC016139_PA	GH1	Glycoside Hydrolase Family 1 protein	fragment C-term;
LDEC016141_PA	GH1	Glycoside Hydrolase Family 1 protein	fragment N-term;
LDEC016380_PA	GH38	Glycoside Hydrolase Family 38 protein	
LDEC016402_PA	GT8	Glycosyltransferase Family 8 protein	fragment N-term;
LDEC016403_PA	GT8	Glycosyltransferase Family 8 protein	fragment N-term / C-term;
LDEC016472_PA	GT49	Glycosyltransferase Family 49 protein	
LDEC016473_PA	CBM14-CBM14	Carbohydrate-Binding Module Family 14 protein	fragment N-term;
LDEC016508_PA	CBM14-CBM14-CBM14	Carbohydrate-Binding Module Family 14 protein	
LDEC016567_PA	GH37	Glycoside Hydrolase Family 37 protein	
LDEC016579_PA	CBM14	Carbohydrate-Binding Module Family 14 protein	
LDEC016666_PA	CBM14	Carbohydrate-Binding Module Family 14 protein	splicing problem;
LDEC016674_PA	GT2	Glycosyltransferase Family 2 protein	fragment N-term;
LDEC016675_PA	GT2	Glycosyltransferase Family 2 protein	fragment C-term;
LDEC016751_PA	GH1	Glycoside Hydrolase Family 1 protein	fragment N-term;
LDEC016877_PA	GT1	Glycosyltransferase Family 1 protein	fragment N-term;
LDEC016973_PA	GT2	Glycosyltransferase Family 2 protein	splicing problem;
LDEC017054_PA	GT43	Glycosyltransferase Family 43 protein	fragment N-term;
LDEC017056_PA	GT43	Glycosyltransferase Family 43 protein	fragment C-term;
LDEC017119_PA	GT1	Glycosyltransferase Family 1 protein	fragment N-term;

LDEC017152_PA	GH1	Glycoside Hydrolase Family 1 protein	fragment C-term;
LDEC017307_PA	GH16	Glycoside Hydrolase Family 16 protein	
LDEC017478_PA	GT31	Glycosyltransferase Family 31 protein	
LDEC017486_PA	GT1	Glycosyltransferase Family 1 protein	splicing problem;
LDEC017584_PA	CBM14	Carbohydrate-Binding Module Family 14 protein	
LDEC017660_PA	GH1	Glycoside Hydrolase Family 1 protein	
LDEC017733_PA	GH35	Glycoside Hydrolase Family 35 protein	
LDEC017832_PA	CBM14	Carbohydrate-Binding Module Family 14 protein	
LDEC017840_PA	CBM14	Carbohydrate-Binding Module Family 14 protein	
LDEC017853_PA	GH31	Glycoside Hydrolase Family 31 protein	fragment N-term;
LDEC017855_PA	GH27	Glycoside Hydrolase Family 27 protein	fragment N-term;
LDEC017972_PA	CBM14-CBM14	Carbohydrate-Binding Module Family 14 protein	
LDEC018149_PA	CBM14-CBM14-CBM14-CBM14-CBM14-CBM14-CBM14-CBM14-CBM14-CBM14-CBM14-CBM14	Carbohydrate-Binding Module Family 14 protein	
LDEC018187_PA	GH84	Glycoside Hydrolase Family 84 protein	fragment C-term;
LDEC018223_PA	GH1	Glycoside Hydrolase Family 1 protein	fragment N-term;
LDEC018224_PA	GH1	Glycoside Hydrolase Family 1 protein	fragment C-term;
LDEC018226_PA	GH1	Glycoside Hydrolase Family 1 protein	fragment C-term;
LDEC018227_PA	GH1	Glycoside Hydrolase Family 1 protein	fragment N-term;
LDEC018229_PA	GH18	Glycoside Hydrolase Family 18 protein	
LDEC018304_PA	GH1	Glycoside Hydrolase Family 1 protein	fragment N-term;
LDEC018308_PA	GT1	Glycosyltransferase Family 1 protein	fragment N-term;
LDEC018364_PA	GT2	Glycosyltransferase Family 2 protein	fragment C-term;
LDEC018365_PA	GT2	Glycosyltransferase Family 2 protein	fragment N-term / C-term;
LDEC018366_PA	GT2	Glycosyltransferase Family 2 protein	fragment N-term;
LDEC018409_PA	CBM14-CBM14-CBM14-CBM14-CBM14-CBM14-CBM14-CBM14	Carbohydrate-Binding Module Family 14 protein	
LDEC018494_PA	GH85	Glycoside Hydrolase Family 85 protein	fragment C-term;
LDEC018548_PA	GH20	Glycoside Hydrolase Family 20 protein	fragment N-term;
LDEC018549_PA	GH20	Glycoside Hydrolase Family 20 protein	fragment C-term;
LDEC018618_PA	GH1	Glycoside Hydrolase Family 1 protein	fragment N-term;
LDEC018620_PA	GH1	Glycoside Hydrolase Family 1 protein	fragment C-term;
LDEC018639_PA	GT1	Glycosyltransferase Family 1 protein	splicing problem;
LDEC018640_PA	GT1	Glycosyltransferase Family 1 protein	fragment N-term;
LDEC018642_PA	GT1	Glycosyltransferase Family 1 protein	fragment N-term / C-term;
LDEC018643_PA	GT1	Glycosyltransferase Family 1 protein	fragment N-term;
LDEC018644_PA	GT1	Glycosyltransferase Family 1 protein	fragment C-term;
LDEC018771_PA	GT1	Glycosyltransferase Family 1 protein	fragment N-term;

LDEC018925_PA	GH116	Glycoside Hydrolase Family 116 protein	fragment N-term;
LDEC018930_PA	GT22	Glycosyltransferase Family 22 protein	fragment C-term;
LDEC018941_PA	GH13_15	Glycoside Hydrolase Family 13 protein	
LDEC018942_PA	GT49	Glycosyltransferase Family 49 protein	
LDEC019016_PA	GH29	Glycoside Hydrolase Family 29 protein	splicing problem;
LDEC019066_PA	GH38	Glycoside Hydrolase Family 38 protein	fragment C-term;
LDEC019069_PA	GH38	Glycoside Hydrolase Family 38 protein	fragment N-term / C-term;
LDEC019070_PA	GH38	Glycoside Hydrolase Family 38 protein	fragment N-term / C-term;
LDEC019097_PA	GT59	Glycosyltransferase Family 59 protein	fragment N-term;
LDEC019148_PA	GH1	Glycoside Hydrolase Family 1 protein	fragment C-term;
LDEC019149_PA	GH1	Glycoside Hydrolase Family 1 protein	fragment N-term;
LDEC019187_PA	GT1	Glycosyltransferase Family 1 protein	fragment N-term;
LDEC019195_PA	GT23	Glycosyltransferase Family 23 protein	
LDEC019233_PA	GH20	Glycoside Hydrolase Family 20 protein	
LDEC019301_PA	GT31	Glycosyltransferase Family 31 protein	
LDEC019539_PA	CBM14	Carbohydrate-Binding Module Family 14 protein	
LDEC019629_PA	GT57	Glycosyltransferase Family 57 protein	fragment N-term / C-term;
LDEC019638_PA	GT4	Glycosyltransferase Family 4 protein	fragment N-term;
LDEC019765_PA	GT1	Glycosyltransferase Family 1 protein	fragment N-term;
LDEC019766_PA	GT1	Glycosyltransferase Family 1 protein	fragment C-term;
LDEC019777_PA	GH1	Glycoside Hydrolase Family 1 protein	fragment N-term;
LDEC019988_PA	GH47	Glycoside Hydrolase Family 47 protein	fragment C-term;
LDEC019989_PA	GH47	Glycoside Hydrolase Family 47 protein	fragment N-term / C-term;
LDEC019990_PA	GH47	Glycoside Hydrolase Family 47 protein	fragment N-term;
LDEC020286_PA	GH45	Glycoside Hydrolase Family 45 protein	
LDEC020378_PA	GT4	Glycosyltransferase Family 4 protein	fragment C-term;
LDEC020391_PA	GH22	Glycoside Hydrolase Family 22 protein	fragment N-term / C-term;
LDEC020501_PA	GT31	Glycosyltransferase Family 31 protein	fragment C-term;
LDEC020713_PA	GT66	Glycosyltransferase Family 66 protein	fragment N-term;
LDEC020714_PA	GT66	Glycosyltransferase Family 66 protein	fragment C-term;
LDEC020814_PA	GT92	Glycosyltransferase Family 92 protein	fragment C-term;
LDEC020847_PA	GH1	Glycoside Hydrolase Family 1 protein	
LDEC020998_PA	GH20	Glycoside Hydrolase Family 20 protein	
LDEC021060_PA	GT27-CBM13	Glycosyltransferase Family 27 / Carbohydrate-Binding Module Family 13 protein	splicing problem;
LDEC021068_PA	GH1	Glycoside Hydrolase Family 1 protein	fragment C-term;
LDEC021129_PA	GT21	Glycosyltransferase Family 21	fragment N-term;

		protein	
LDEC021347_PA	GT27-CBM13	Glycosyltransferase Family 27 / Carbohydrate-Binding Module Family 13 protein	splicing problem;
LDEC021369_PA	GH1	Glycoside Hydrolase Family 1 protein	fragment N-term / C-term;
LDEC021704_PA	GH22	Glycoside Hydrolase Family 22 protein	
LDEC021748_PA	GT21	Glycosyltransferase Family 21 protein	fragment C-term;
LDEC021794_PA	GT7	Glycosyltransferase Family 7 protein	splicing problem;
LDEC022117_PA	GT1	Glycosyltransferase Family 1 protein	fragment N-term;
LDEC022154_PA	GH20	Glycoside Hydrolase Family 20 protein	splicing problem;
LDEC022264_PA	GH1	Glycoside Hydrolase Family 1 protein	fragment N-term / C-term;
LDEC022265_PA	GH1	Glycoside Hydrolase Family 1 protein	fragment C-term;
LDEC022352_PA	GH29	Glycoside Hydrolase Family 29 protein	fragment C-term;
LDEC022592_PA	GT54	Glycosyltransferase Family 54 protein	fragment N-term / C-term;
LDEC022794_PA	GH22	Glycoside Hydrolase Family 22 protein	fragment N-term;
LDEC022798_PA	CBM14-CBM14-CBM14	Carbohydrate-Binding Module Family 14 protein	
LDEC022800_PA	GH31	Glycoside Hydrolase Family 31 protein	fragment N-term / C-term;
LDEC022910_PA	CBM14	Carbohydrate-Binding Module Family 14 protein	
LDEC022929_PA	GH31	Glycoside Hydrolase Family 31 protein	fragment N-term / C-term;
LDEC023093_PA	GT31	Glycosyltransferase Family 31 protein	
LDEC023131_PA	GH38	Glycoside Hydrolase Family 38 protein	fragment N-term / C-term;
LDEC023147_PA	GT1	Glycosyltransferase Family 1 protein	fragment N-term / C-term;
LDEC023197_PA	GH20	Glycoside Hydrolase Family 20 protein	fragment N-term / C-term;
LDEC023222_PA	GH37	Glycoside Hydrolase Family 37 protein	fragment C-term;
LDEC023267_PA	GT1	Glycosyltransferase Family 1 protein	
LDEC023422_PA	GT1	Glycosyltransferase Family 1 protein	fragment N-term;
LDEC023432_PA	GT1	Glycosyltransferase Family 1 protein	fragment N-term / C-term;
LDEC023442_PA	GH18	Glycoside Hydrolase Family 18 protein	fragment C-term;
LDEC023514_PA	GT17	Glycosyltransferase Family 17 protein	
LDEC023578_PA	GH20	Glycoside Hydrolase Family 20 protein	fragment N-term / C-term;
LDEC023654_PA	GT1	Glycosyltransferase Family 1 protein	fragment N-term / C-term;
LDEC023678_PA	GT8	Glycosyltransferase Family 8 protein	fragment C-term;
LDEC023743_PA	GH1	Glycoside Hydrolase Family 1 protein	fragment N-term;
LDEC023934_PA	GT39	Glycosyltransferase Family 39 protein	fragment N-term;
LDEC023994_PA	GT1	Glycosyltransferase Family 1 protein	fragment N-term / C-term;
LDEC024072_PA	GH45-GH45	Glycoside Hydrolase Family 45 protein	
LDEC024184_PA	GH38	Glycoside Hydrolase Family 38 protein	fragment N-term / C-term;
LDEC024202_PA	CBM14-CBM14-CBM14	Carbohydrate-Binding Module Family 14 protein	

LDEC024277_PA	GH16	Glycoside Hydrolase Family 16 protein	fragment N-term / C-term;
LDEC024494_PA	GT39	Glycosyltransferase Family 39 protein	fragment N-term / C-term;
LDEC024574_PA	GH13_25	Glycoside Hydrolase Family 13 protein	fragment N-term;
LDEC024614_PA	GH1	Glycoside Hydrolase Family 1 protein	fragment N-term / C-term;

Table 22S. Clusters of genes encoding cuticle proteins in the genome of *Leptinotarsa decemlineata*.

	Scaffold #	# Genes	Family	Length (Kbp)	Density (Kbp/gene)
1	305	20	CPR RR-2	220	11.0
2	20	18	CPR RR-1/CPR Uncl	610	33.9
3	10	17	CPR RR-2/CPR Uncl	348	20.5
4	117	11	CPR RR-1	306	27.8
5	47	7	CPR RR-2/CPR Uncl	478	68.3
6	64	6	TWDL	164	27.2
7	297	4	CPR RR-2	55	13.6
8	295	3	CPR RR-1	70	23.3
9	964	3	CPR RR-2/CPR Uncl	97	32.4

Table 23S. Genes associated with RNA interference in *Leptinotarsa decemlineata.*

Category	Gene Name	Location	Tribolium Homolog
Dicer Family	Dicer-1	Scaffold4:1953612..1966951	TC001750
	Dicer-2a	Scaffold557:657805..683537	TC001108
	Dicer-2b	Scaffold723:316634..344171	TC001108
	Drosha	Scaffold440:17396..101377	TC016208
Argonautes	Ago-1	Scaffold255:189144..247144	TC005857
	Ago-2a	Scaffold740:337679..351812	TC011525
	Ago-2b	Scaffold740:322668..330275; Scaffold19765:3658..4119	TC013762
	Aubergine/Piwi	Scaffold492:128965..183831	TC008711
	Ago-3	Scaffold52:1144302..1167444	TC008511
dsRNA Binding	R2D2	Scaffold27:1222406..1225477	TC008716
	loquacious	Scaffold898:221935..260468	TC011666
	pasha	Scaffold21:1391464..1392897;Scaffold8844:3674..12696	TC015332
Systemic RNAi – dsRNA uptake	SID1-related A	Scaffold278:602740..622120	TC011760
	SID1-related C	Scaffold281:261440..284078	TC015033
Systemic RNAi – dsRNA export	Epsin-like (rsd-3)	Scaffold278:345374..358991	TC012168
	liquid facets (Epn-1)	Scaffold134:491488..504533	TC005393
RNA-dependent RNA polymerase	Elp-1	Scaffold161:465307..472844	TC015781
Endosome Transport	Arf72A	Scaffold93:424612..42744	TC008443
Clathrin Endocytosis	AP 50	Scaffold272:726268-729030;Scaffold1101:183745-188337	TC011923
	Clathrin hc	Scaffold2037:260184..283654	TC015014
Exocytosis	IdICP	Scaffold1:1790498..1794021	TC010886
Lysosomal Transport	Light	Scaffold2260:28538..36890	TC015204
Rhodopsin-mediated signaling	Nina C	Scaffold460:475139..539606	TC014087
ATP Synthase/ATPase	Vha16	Scaffold248:544081..575090	TC011025
	VhaSFD	Scaffold40:320894..345317	TC006281
Auxiliary RISC-	Translin	Scaffold396:331037..333677	TC009885

associated or regulatory factors	Trax-B	Scaffold7483:9..3072; Scaffold1468:111798..148472	XP_008195924
	Hen1	Scaffold845:164503..192398	TC004824
	Gawky	Scaffold220:551316..586481	TC006679
	FXMR	Scaffold706:173027..268200	TC003774
	Tudor-SN	Scaffold1292:145408..158021	TC002936
	Belle	Scaffold644:77897..129634	TC013328
	p68 RNA helicase	Scaffold16:1266030..1272240	
	Armitage	Scaffold842:239770..258458	TC010546
	putative RNA helicase (armitage-like)	Scaffold509:162029..225517	
	Staufen	Scaffold45:332077..342844	TC004615
	Neuron-Specific Staufen	Scaffold7252:12722..18330;Scaffold21:976658..979896	TC008157
	Maelstrom	Scaffold44:1098636..1102196	TC008172
	PRMT5	Scaffold649:780257..782508	TC003689
	Clp1 kinase	Scaffold249:356809..359396	TC009961
DsRNA uptake	Hermansky-Pudlak Syndrome 4 (HPS4)	Scaffold123:307738..0321303	TC002372
	scavenger receptor-C 2 (SR-C) Partial	Scaffold215:443302..452628;Scaffold14935:3694..4145	TC015640
	FBX011	Scaffold35:293128..326905	TC010102
Nucleases	SDN-like (I)	Scaffold1200:128266..142769	TC003027
	Nibbler	Scaffold349:15850..25334;Scaffold2142:59856..60785	TC002596
	SDN-like (III)	Scaffold799:123632..127302	TC002980
Antiviral RNAi	Ars2	Scaffold190:107540..119936	
	Egghead	Scaffold642:26552...71965	TC008154
	CG4572	Scaffold216:182107..184431	TC02692

Table 24S. Genes associated with venom in *Leptinotarsa decemlineata.*

LDEC Gene ID	Annotation
LDEC000596-RA	venom carboxylesterase-6-like
LDEC001753-RA	venom carboxylesterase-6-like
LDEC002822-RA	venom carboxylesterase-6-like
LDEC003521-RA	venom carboxylesterase-6-like
LDEC005267-RA	venom carboxylesterase-6-like
LDEC005268-RA	venom carboxylesterase-6-like
LDEC006843-RA	venom carboxylesterase-6-like
LDEC009957-RA	venom carboxylesterase-6-like
LDEC012343-RA	venom carboxylesterase-6-like
LDEC012572-RA	venom carboxylesterase-6-like
LDEC018985-RA	venom carboxylesterase-6-like
LDEC018985-RA	venom carboxylesterase-6-like
LDEC019608-RA	venom carboxylesterase-6-like
LDEC021156-RA	venom carboxylesterase-6-like
LDEC021157-RA	venom carboxylesterase-6-like

LDEC022145-RA	venom carboxylesterase-6-like
LDEC022567-RA	venom carboxylesterase-6-like
LDEC022654-RA	venom carboxylesterase-6-like
LDEC023739-RA	venom carboxylesterase-6-like
LDEC024628-RA	venom carboxylesterase-6-like
LDEC000039-RA	aminopeptidase N
LDEC001405-RA	aminopeptidase N
LDEC001406-RA	aminopeptidase N
LDEC001410-RA	aminopeptidase N
LDEC001411-RA	aminopeptidase N
LDEC001412-RA	aminopeptidase N
LDEC001413-RA	aminopeptidase N
LDEC001414-RA	aminopeptidase N
LDEC020617-RA	aminopeptidase N
LDEC020618-RA	aminopeptidase N
LDEC013430-RA	hyaluronidase-like
LDEC007712-RA	venom acid phosphatase Acph-1-like
LDEC007713-RA	venom acid phosphatase Acph-1-like
LDEC007860-RA	venom acid phosphatase Acph-1-like
LDEC021275-RA	venom acid phosphatase Acph-1-like
LDEC021275-RA	venom acid phosphatase Acph-1-like
LDEC003829-RA	venom allergen
LDEC003531-RA	venom allergen 3-like
LDEC003531-RA	venom allergen 3-like
LDEC013326-RA	venom allergen 3-like
LDEC013326-RA	venom allergen 3-like
LDEC015068-RA	venom allergen 3-like
LDEC015068-RA	venom allergen 3-like
LDEC021266-RA	venom carboxylesterase-6-like isoform X2
LDEC011393-RA	venom dipeptidyl peptidase 4
LDEC006659-RA	venom dipeptidyl peptidase 4 isoform X5
LDEC006659-RA	venom dipeptidyl peptidase 4 isoform X5
LDEC001897-RA	venom protease-like
LDEC001898-RA	venom protease-like
LDEC019017-RA	venom protease-like
LDEC020482-RA	venom protease-like
LDEC020483-RA	venom protease-like
LDEC020485-RA	venom protease-like isoform X1
LDEC013552-RA	venom protein r-like partial
LDEC013553-RA	venom protein r-like partial
LDEC013554-RA	venom protein r-like partial
LDEC004488-RA	venom prothrombin activator nigrarin-d-like
LDEC005299-RA	venom prothrombin activator nigrarin-d-like
LDEC008843-RA	venom prothrombin activator nigrarin-d-like
LDEC012024-RA	venom prothrombin activator nigrarin-d-like
LDEC018395-RA	venom prothrombin activator nigrarin-d-like
LDEC009056-RA	venom serine carboxypeptidase
LDEC009057-RA	venom serine carboxypeptidase

LDEC019866-RA	venom serine carboxypeptidase
LDEC006748-RA	venom serine protease 34-like
LDEC017399-RA	venom serine protease 34-like

Table 25S. The Cadherin genes, putative Bt receptors, annotated in *Leptinotarsa decemlineata*.

Gene ID	Scaffold	Start Position	End Position	Gene Name
LDEC000281-RA	Scaffold2	5817105	5822291	Cadherin
LDEC006970-RA	Scaffold141	285861	390558	Cadherin 23
LDEC004644-RA	Scaffold70	495520	536485	Cadherin 23
LDEC004288-RA	Scaffold63	916751	959927	Cadherin 96Ca
LDEC000283-RA	Scaffold2	6154395	6182206	Cadherin-like
LDEC003675-RA	Scaffold53	730343	753322	Cadherin-like
LDEC015036-RA	Scaffold605	326047	358498	Cadherin-like
LDEC000368-RA	Scaffold3	2060012	2166586	Cadherin-like
LDEC006967-RA	Scaffold141	49907	180049	Cadherin-like
LDEC010197-RA	Scaffold271	463411	481151	Cadherin-N
LDEC006991-RA	Scaffold143	343713	346234	DN Cadherin-like
LDEC010196-RA	Scaffold271	450072	457395	DN-Cadherin
LDEC010195-RA	Scaffold271	425770	428669	DN-Cadherin

Supplementary Figures

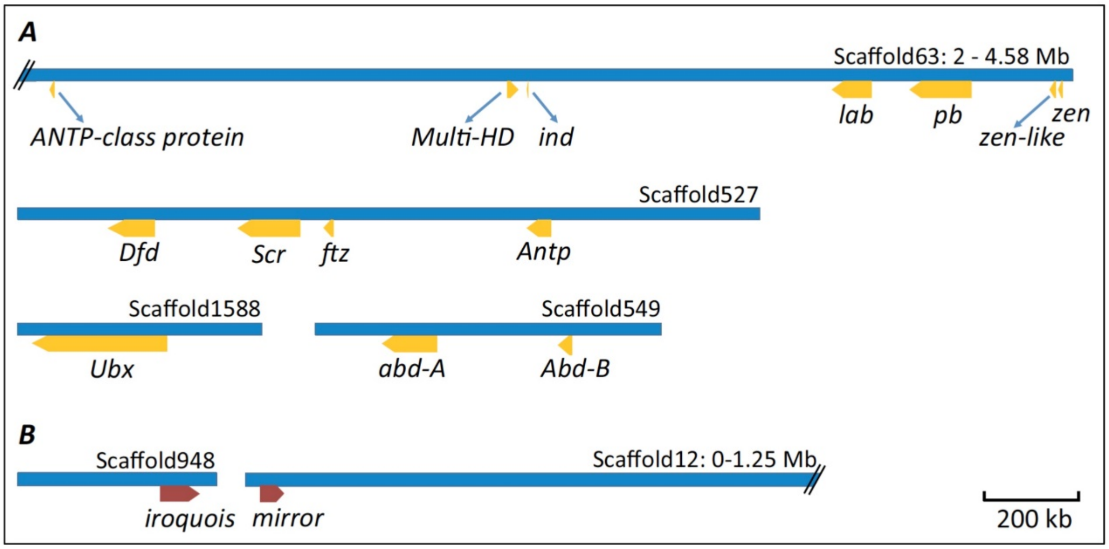

Figure 1S. Organization of the Hox (A) and Iro-C clusters (B). Shown to scale, with transcriptional orientation indicated and scaffolds ordered based on expected cluster organization. For full gene names, see **Supplementary Table 1S**.

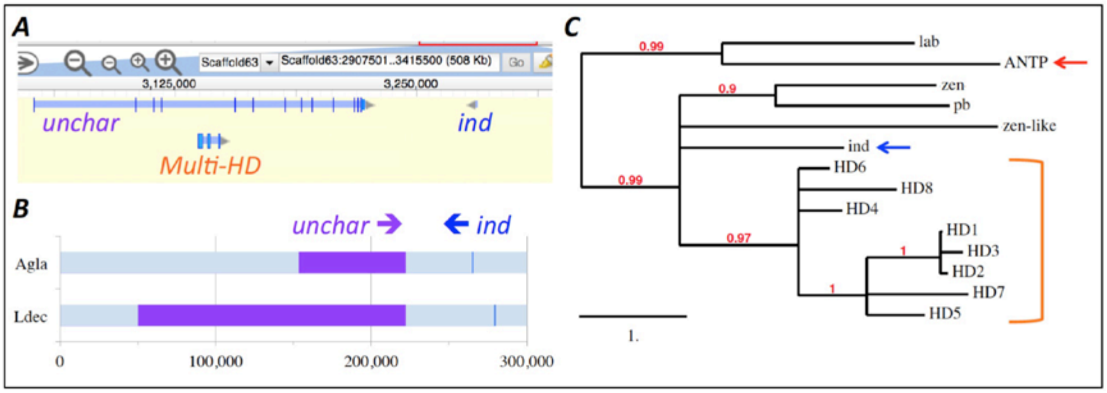

Figure 2S. Organization, synteny, and phylogeny of non-canonical homeodomain genes on Scaffold 63. **A.** Screenshot of the region including the *uncharacterized protein* (“*unchar*”, purple), *Multi-homeodomain protein* (“*Multi-HD*”, orange), and *intermediate neuroblasts defective* (“*ind*”, blue). **B.** The *unchar* and *ind* genes have conserved synteny between *L. decemlineata* (“Ldec”) and *A. glabripennis* (“Agla”), but there is no *Multi-HD* ortholog in the latter. **C.** Maximum likelihood phylogeny of the complete proteins of all *L. decemlineata* homeodomain encoding genes on Scaffold 63. The *ANTP-class homeodomain protein* (*“ANTP”*, red) is most closely related to *labial*, while *ind* (blue) falls within the more general anterior Hox clade. Here, the *Multi-HD* sequence was partitioned into eight homeodomain (“HD”, orange) containing sequence fragments, all of which are more closely related to one another than to other anterior Hox genes. Note that *ind* is a highly conserved homeodomain encoding gene in insects, but is considered non-canonical in this context due to its unexpected linkage to the Hox cluster proper.

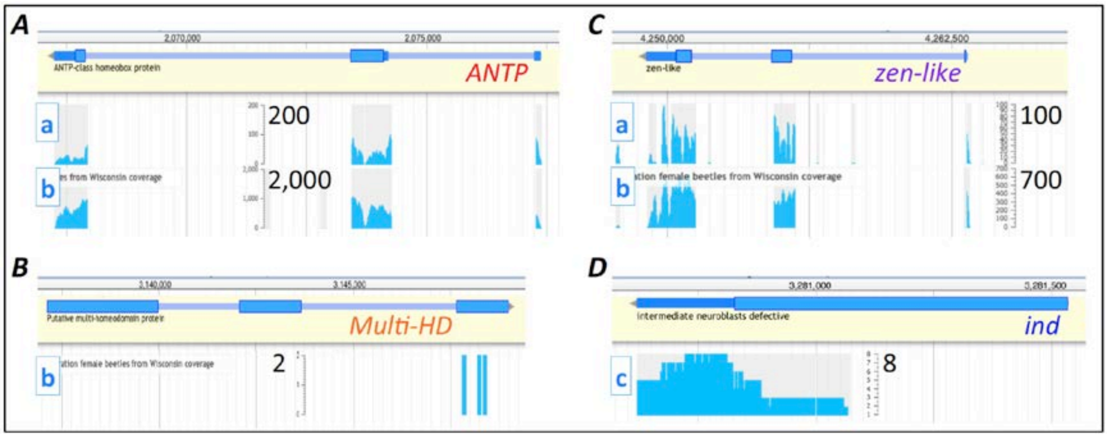

Figure 3S. Expression support for non-canonical homeodomain genes on Scaffold 63. **A.** *ANTP-class homeodomain protein*, **B.** *Multi-homeodomain protein*, **C.** *zen-like*, **D.** *ind*. Screenshots from Apollo show the gene models and relevant RNAseq coverage histograms from the following samples: (a) "Female RNAseq", (b) "Pooled data from 60 adult 1st generation female beetles from Wisconsin", (c) "23 full adult CPB from 4 European populations". Additional RNAseq evidence tracks from other tissue sources did not provide (appreciable) expression support. Histogram numbers indicate the maximum value for each track's y-axis (number of raw RNAseq reads mapped). Both the *ANTP* and *zen-like* genes have strong expression support, and *ind* has moderate support, but *Multi-HD* effectively has no support (only three pairs of individual raw RNAseq reads mapped to this locus).

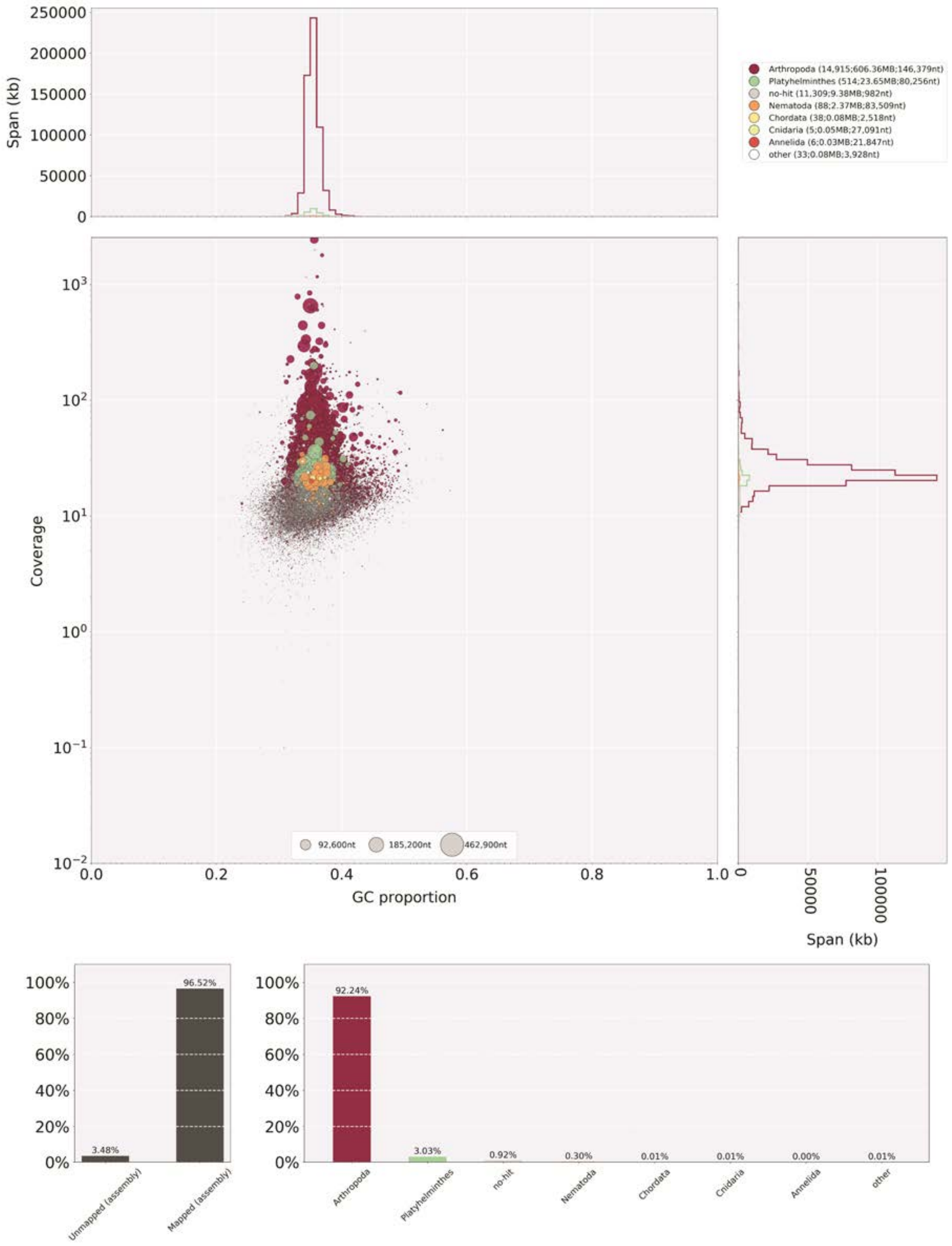

Figure 4S. Screen for possible contamination using BlobTools. A) The taxon-annotated GC-coverage plot. B) A summary of reads mapped to taxonomic groups as putative contaminants.

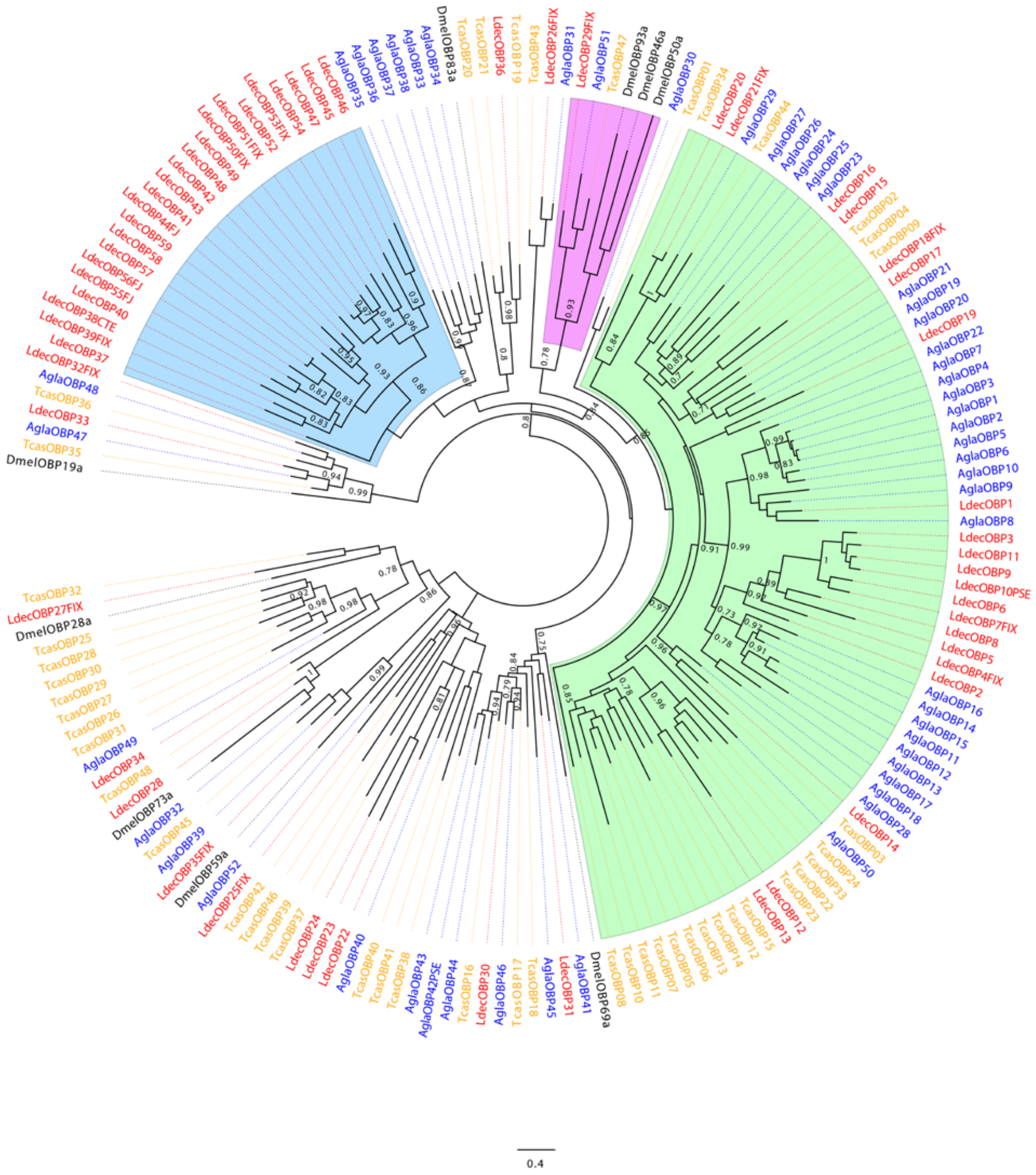

Figure 5S. Approximate maximum-likelihood phylogeny of odorant binding proteins (OBP). Based on MUSCLE alignment of amino acid sequences and FastTree 2.1 algorithm. Species are abbreviated as: *L. decemlineata* (**Ldec**), *A. glabripennis* (**Agla**), *T. castaneum* (**Tcas**), and *D. melanogaster* (**Dmel**). Suffixes to gene names given in Supplementary Table 13S. The "traditional" Minus-C class is indicated in green, the Ldec-specific expansion of additional unrelated Minus-C proteins in blue, and the Plus-C class in purple.

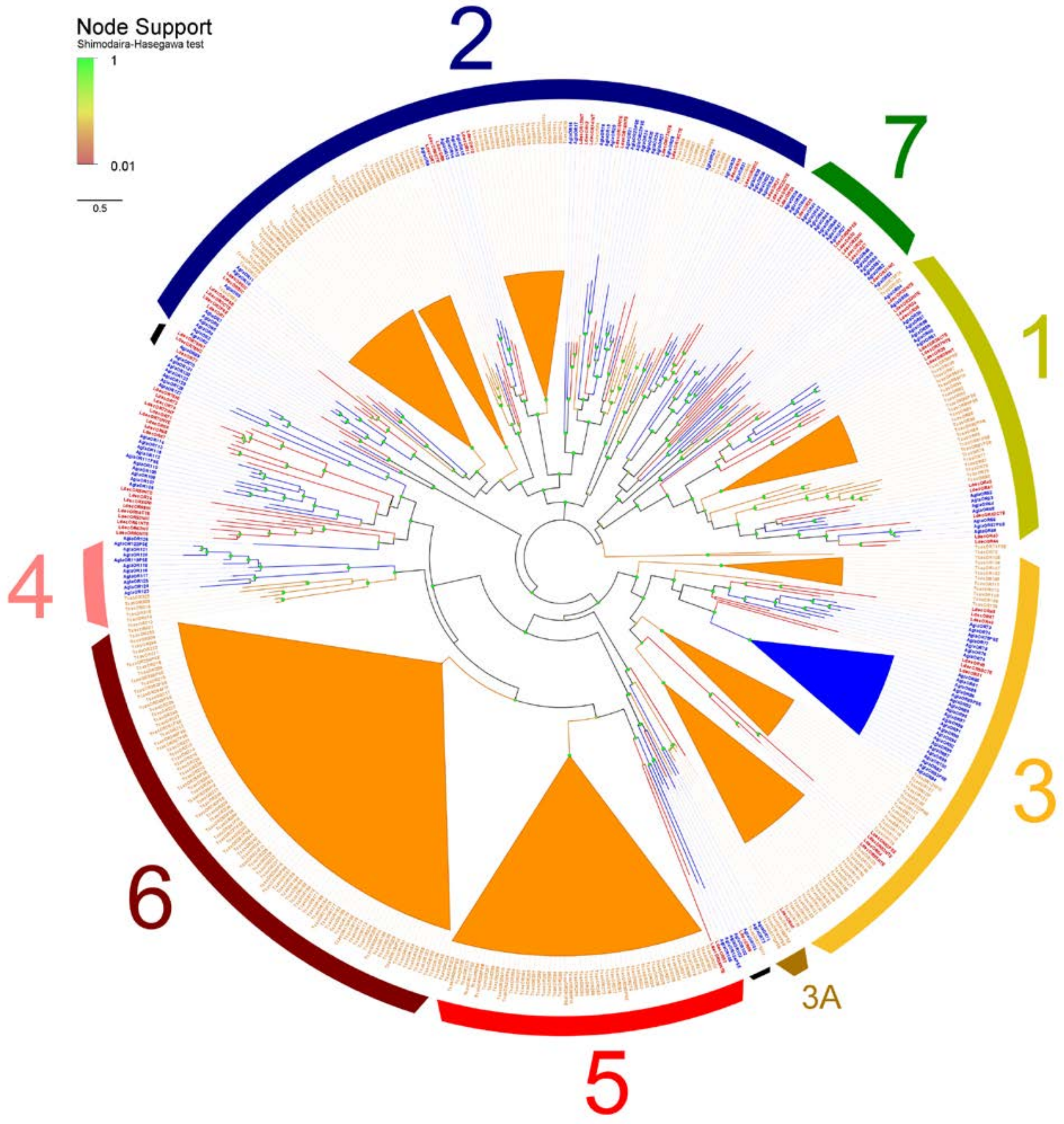

Figure 6S. Unrooted phylogram illustrating the OR gene families of *Leptinotarsa decemlineata*. Species include *L. decemlineata* (**Ldec**), *A. glabripennis* (**Agla**), *T. castaneum* (**Tcas**). Large expansions of AglaORs and TcasORs have been collapsed to improve clarity, but remain color-coded by species. Pseudogenes are indicated by the suffix PSE, and other suffixes indicate missing exons (see supplemental text). Colored arcs indicate the seven described groups of coleopteran ORs. Thin, black arcs indicate smaller radiations of note: lower right, a highly conserved OR clade that appears to be the outgroup to Groups 4, 5, and 6; upper left, a small lineage of apparently chrysomeloid-specific ORs. Colored circles on nodes indicate Shimodaira-Hasegawa support values, with high support indicated both by green color and larger size.

A.

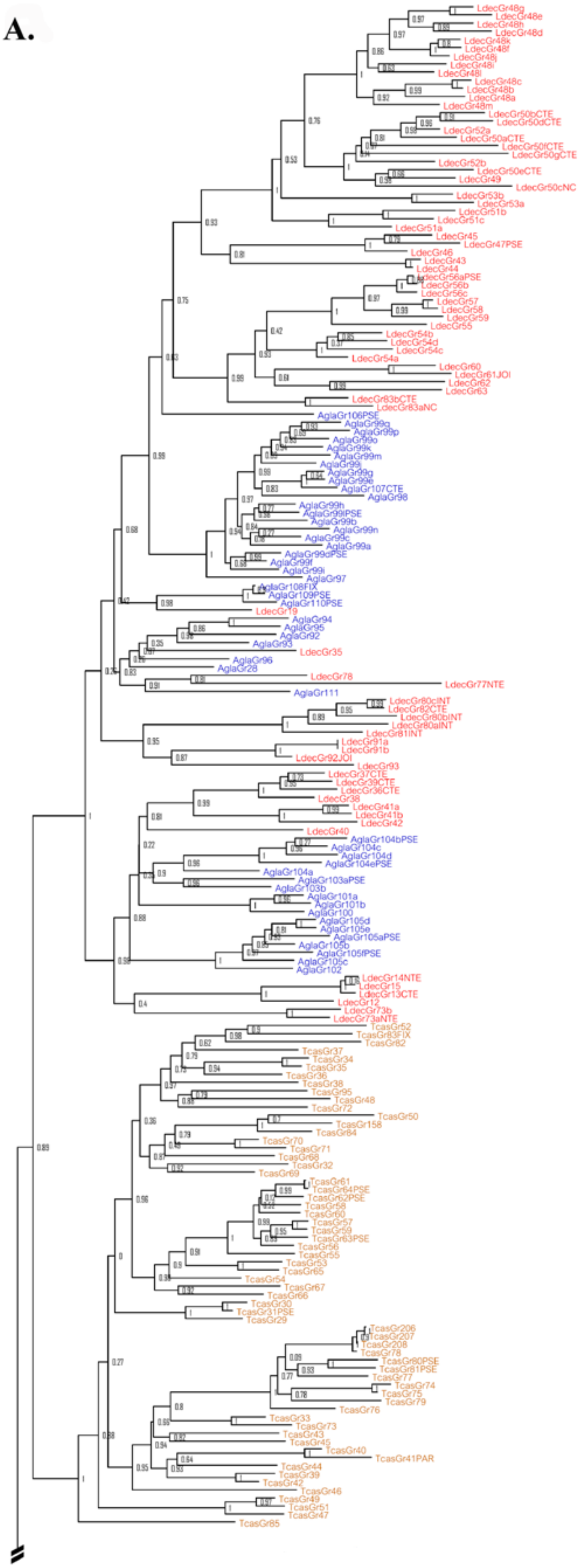

B.

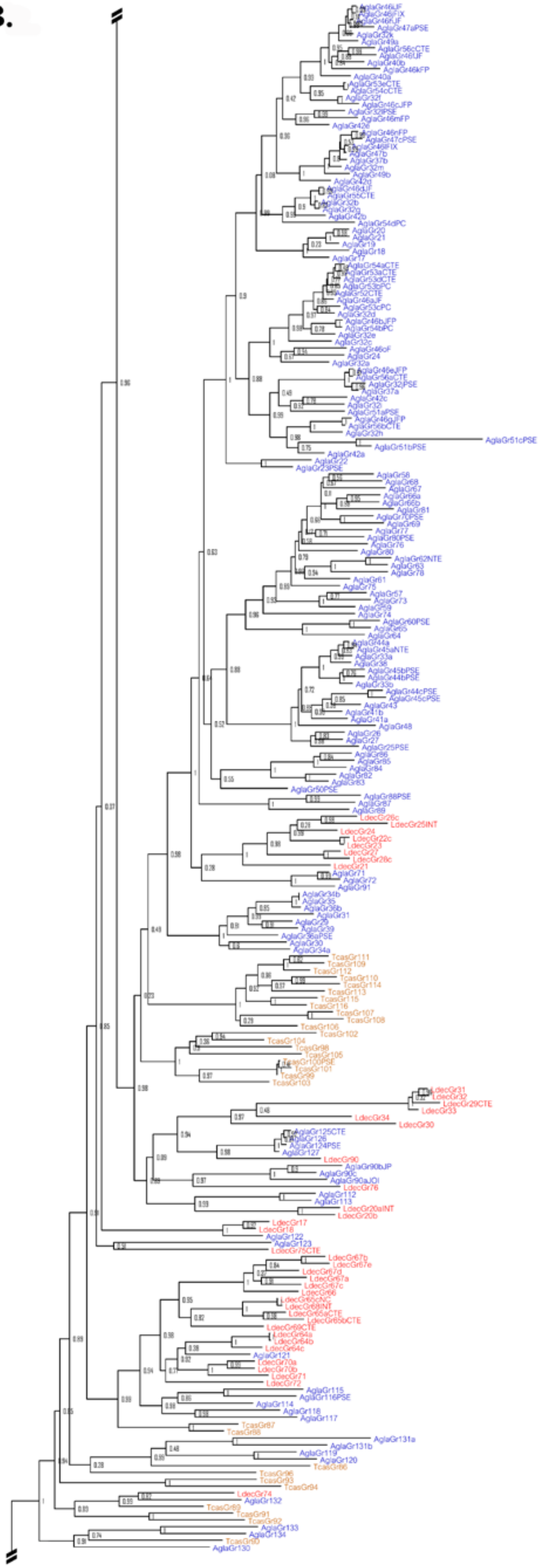

C.

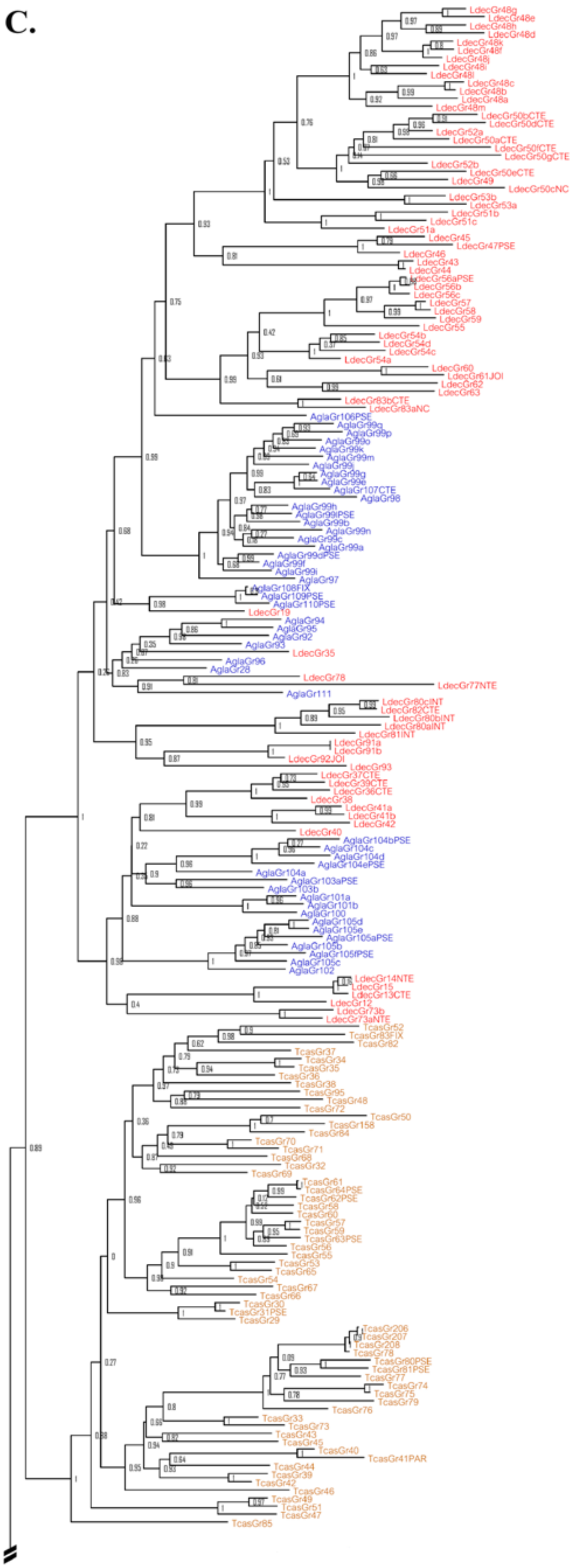
LdecGr48g
LdecGr48e
LdecGr48h
LdecGr48d
LdecGr48k
LdecGr48f
LdecGr48j
LdecGr48i
LdecGr48l
LdecGr48c
LdecGr48b
LdecGr48a
LdecGr48m
LdecGr50bCTE
LdecGr50dCTE
LdecGr52a
LdecGr50aCTE
LdecGr50fCTE
LdecGr50gCTE
LdecGr52b
LdecGr50eCTE
LdecGr49
LdecGr50cNC
LdecGr53b
LdecGr53a
LdecGr51b
LdecGr51c
LdecGr51a
LdecGr45
LdecGr47PSE
LdecGr46
LdecGr43
LdecGr44
LdecGr56aPSE
LdecGr56b
LdecGr56c
LdecGr57
LdecGr58
LdecGr59
LdecGr55
LdecGr54b
LdecGr54d
LdecGr54c
LdecGr54a
LdecGr60
LdecGr61JOI
LdecGr62
LdecGr63
LdecGr83bCTE
LdecGr83aNC
AglaGr106PSE
AglaGr99q
AglaGr99p
AglaGr99o
AglaGr99k
AglaGr99m
AglaGr99j
AglaGr99g
AglaGr99e
AglaGr107CTE
AglaGr98
AglaGr99h
AglaGr99lPSE
AglaGr99b
AglaGr99n
AglaGr99c
AglaGr99a
AglaGr99dPSE
AglaGr99f
AglaGr99i
AglaGr97
AglaGr108FIX
AglaGr109PSE
AglaGr110PSE
LdecGr19
AglaGr94
AglaGr95
AglaGr92
AglaGr93
LdecGr35
AglaGr96
AglaGr28
LdecGr78
LdecGr77NTE
AglaGr111
LdecGr80cINT
LdecGr82CTE
LdecGr80bINT
LdecGr80aINT
LdecGr81INT
LdecGr91a
LdecGr91b
LdecGr92JOI
LdecGr93
LdecGr37CTE
LdecGr39CTE
LdecGr36CTE
LdecGr38
LdecGr41a
LdecGr41b
LdecGr42
LdecGr40
AglaGr104bPSE
AglaGr104c
AglaGr104d
AglaGr104ePSE
AglaGr104a
AglaGr103aPSE
AglaGr103b
AglaGr101a
AglaGr101b
AglaGr100
AglaGr105d
AglaGr105e
AglaGr105aPSE
AglaGr105b
AglaGr105fPSE
AglaGr105c
AglaGr102
LdecGr14NTE
LdecGr15
LdecGr13CTE
LdecGr12
LdecGr73b
LdecGr73aNTE
TcasGr52
TcasGr83FIX
TcasGr82
TcasGr37
TcasGr34
TcasGr35
TcasGr36
TcasGr38
TcasGr95
TcasGr48
TcasGr72
TcasGr50
TcasGr158
TcasGr84
TcasGr70
TcasGr71
TcasGr68
TcasGr32
TcasGr69
TcasGr61
TcasGr64PSE
TcasGr62PSE
TcasGr58
TcasGr60
TcasGr57
TcasGr59
TcasGr63PSE
TcasGr56
TcasGr55
TcasGr53
TcasGr65
TcasGr54
TcasGr67
TcasGr66
TcasGr30
TcasGr31PSE
TcasGr29
TcasGr206
TcasGr207
TcasGr208
TcasGr78
TcasGr80PSE
TcasGr81PSE
TcasGr77
TcasGr74
TcasGr75
TcasGr79
TcasGr76
TcasGr33
TcasGr73
TcasGr43
TcasGr45
TcasGr40
TcasGr41PAR
TcasGr44
TcasGr39
TcasGr42
TcasGr46
TcasGr49
TcasGr51
TcasGr47
TcasGr85

Figure 7S. Approximate maximum-likelihood phylogeny of the gustatory receptor proteins (GRs). Based on MUSCLE alignment of amino acid sequences and the FastTree 2.1 algorithm. Species are abbreviated as: *L. decemlineata* (**Ldec**), *A. glabripennis* (**Agla**), *T. castaneum* (**Tcas**), and *D. melanogaster* (**Dmel**). Due to its large size, tree was divided into three parts (A-Bottom, B-Middle and C-Top).

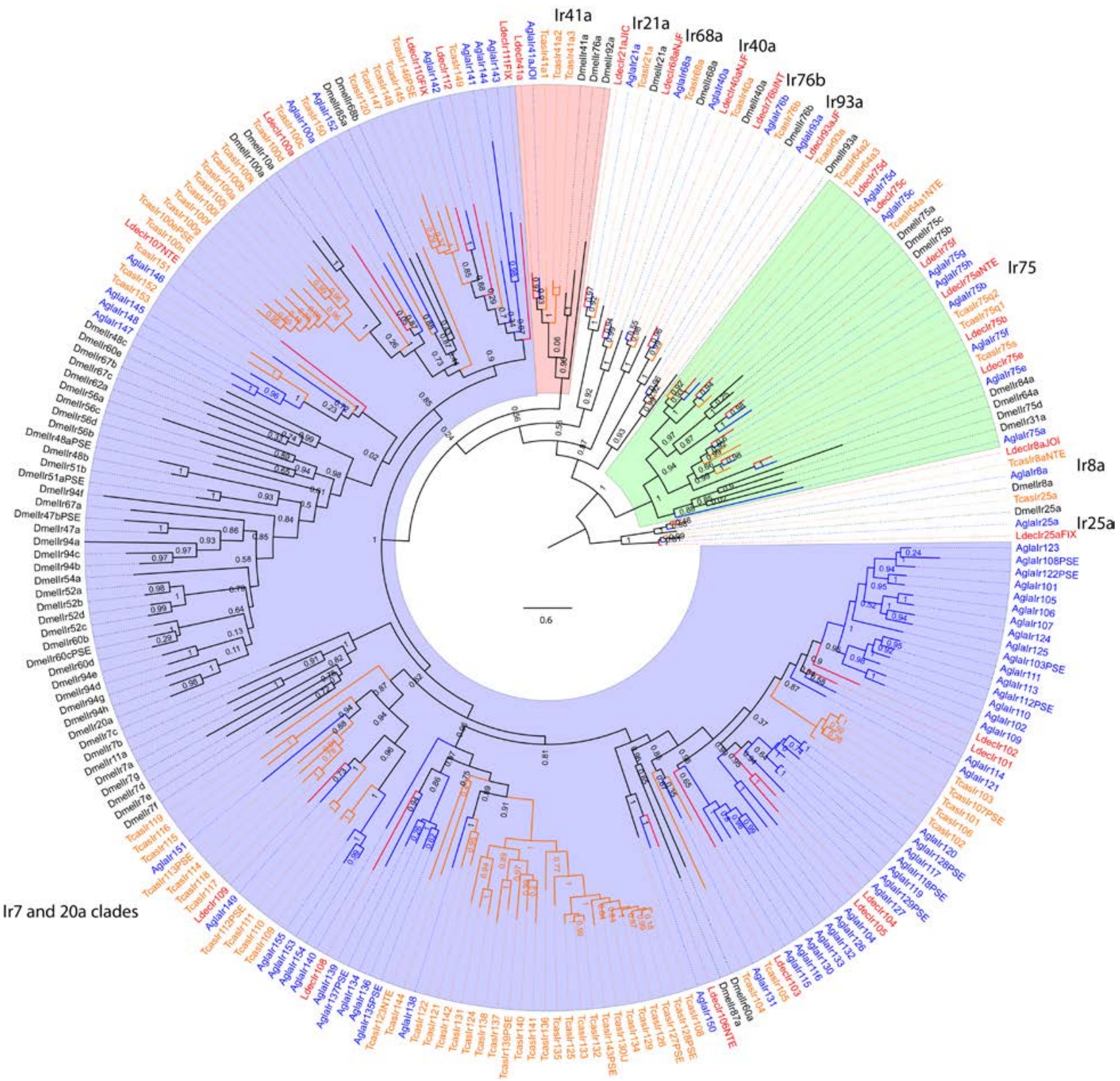

Figure 8S. Approximate maximum-likelihood phylogeny of the ionotropic receptor (IR) proteins. Based on ClustalX alignment of amino acid sequences and the PHYML algorithm, where support values at nodes are approximate Likelihood-Ratio Tests. Species are abbreviated as: *L. decemlineata* (Ldec), *A. glabripennis* (Agla), *T. castaneum* (Tcas), and *D. melanogaster* (Dmel). The tree was rooted by declaring the conserved and basal Ir8a and 25a proteins as the outgroup. Highly conserved simple orthologs are indicated by name outside the circle, while three clades are highlighted, the Ir41a clade in pink, the Ir75 clade in green, and the divergent proteins related to the Ir7 and 20a clades in *D. melnaogaster* in blue.

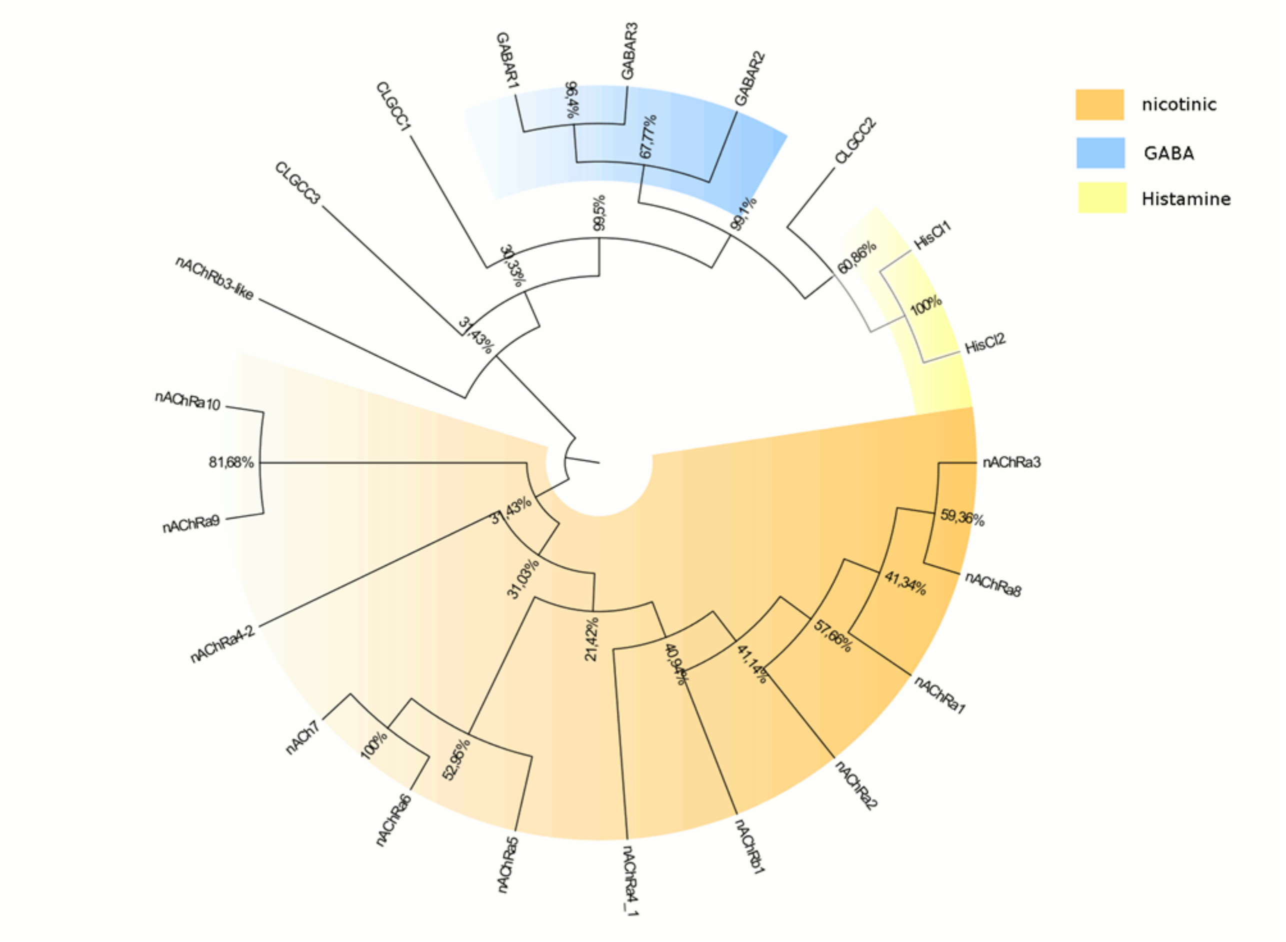

Figure 9S. Phylogenetic tree of cys-loop ligand-gated ion channels in *Leptinotarsa decemlineata*. The tree was constructed using the Neighbor-joining method in MEGA6, using Poisson correction and 1,000 replicates bootstrap replicates.

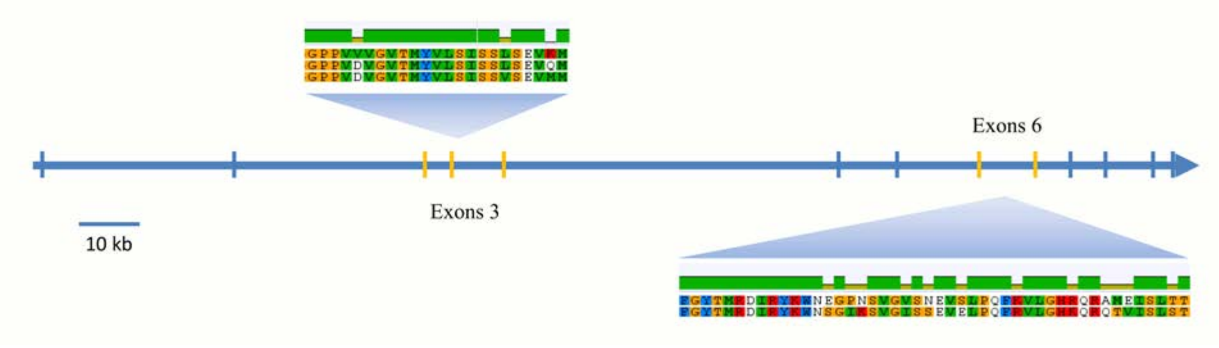

Figure 10S. Representation of the exon structure of GABAR1 and the alternative versions of exon 3 and 6.

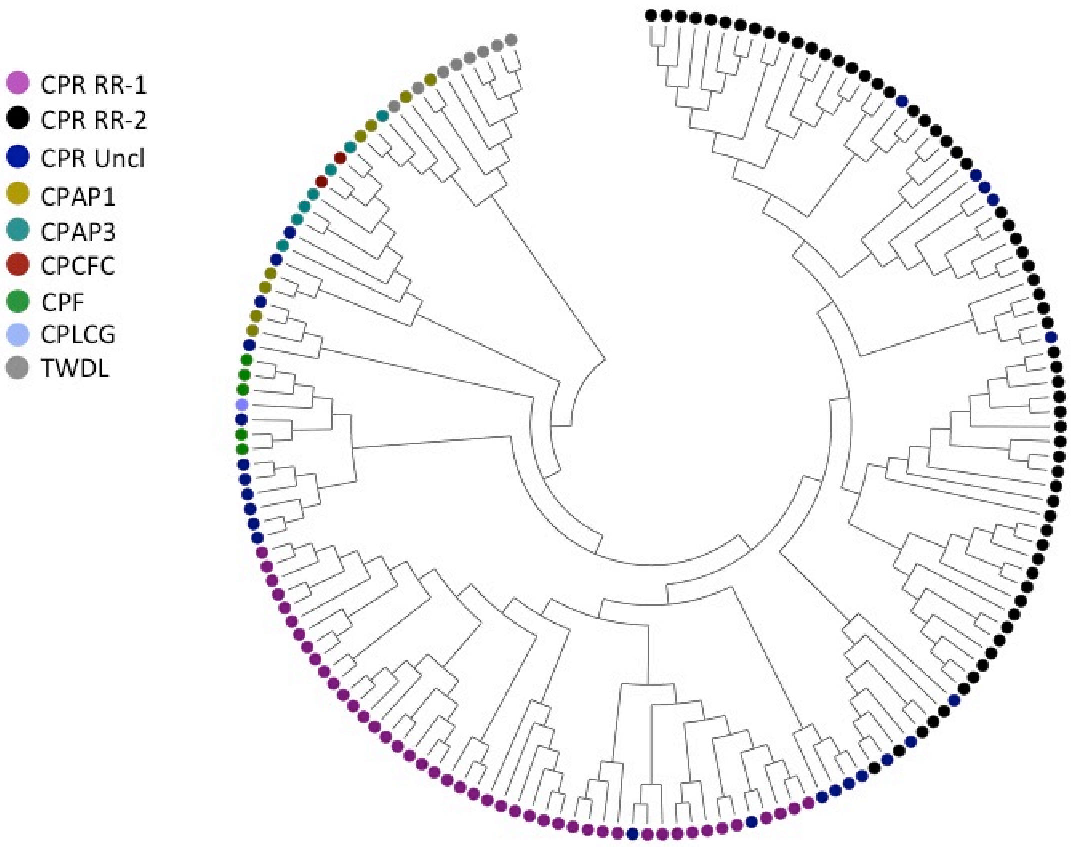

Figure 11S. Phylogenetic tree of cuticle proteins from *Leptinotarsa decemlineata*. The tree was constructed using the Neighbor-joining method in MEGA6, using Poisson correction and 1,000 replicates bootstrap replicates.

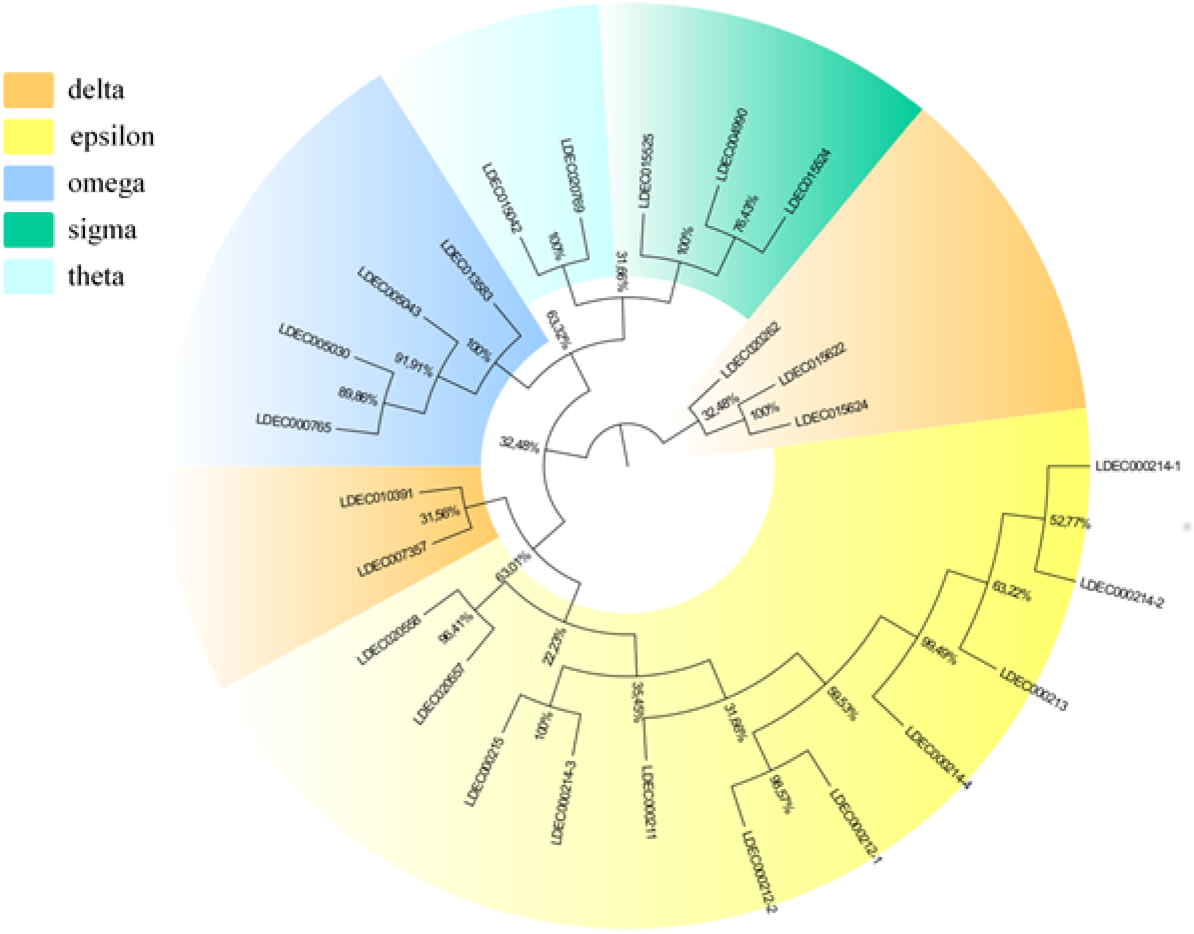

Figure 12S. Phylogenetic tree of cytosolic GST proteins from *Leptinotarsa decemlineata*. The tree was constructed using the Neighbor-joining method in MEGA6, using Poisson correction and 1,000 replicates bootstrap replicates.
